# Structure–activity relationships of fenarimol analogues with potent *in vitro* and *in vivo* activity against *Madurella mycetomatis*, the main causative agent of mycetoma

**DOI:** 10.1039/d5md00427f

**Published:** 2025-09-18

**Authors:** Hung Phat Duong, Dmitrij Melechov, Wilson Lim, Jingyi Ma, Kymberley R. Scroggie, Luxsika Rajendra, Benjamin Perry, Luiza R. Cruz, Rahman Shah Zaib Saleem, Peter J. Rutledge, Alice Motion, Wendy W. J. van de Sande, Matthew H. Todd

**Affiliations:** a School of Chemistry, Faculty of Science, The University of Sydney NSW 2006 Australia alice.motion@sydney.edu.au; b UCL School of Pharmacy, University College London 29-39 Brunswick Square London WC1N 1AX UK matthew.todd@ucl.ac.uk; c Structural Genomics Consortium, University College London 29-39 Brunswick Square London WC1N 1AX UK; d Department of Medical Microbiology & Infectious Diseases, Erasmus MC, University Medical Center Rotterdam Dr. Molewaterplein 40 3015 GD Rotterdam The Netherlands w.vandesande@erasmusmc.nl; e The Drugs for Neglected Diseases Initiative 15 Chemin Camille-Vidart 1202 Geneva Switzerland; f Department of Chemistry and Chemical Engineering, Syed Babar Ali School of Science and Engineering, Lahore University of Management Sciences Lahore 54792 Pakistan

## Abstract

The fenarimol analogue EPL-BS1246 was previously discovered to be potent against *Madurella mycetomatis*, the causative agent of the neglected tropical disease mycetoma. Further evaluation of a small set of fenarimol analogues *in vivo* revealed a correlation between efficacy and the lipophilicity (log *D*) of the analogues. To explore both this correlation and the series structure–activity relationship (SAR), we have evaluated a total of 185 fenarimol analogues derived from five different daughter chemotypes. Potent (MIC_50_ < 9 μM) *in vitro* activity was found for 22 analogues, five of which gave promising results in an *in vivo* larval survival assay. Again, a trend towards prolonged larval survival (better *in vivo* activity) was noted in analogues with log *D* values <2.5. Insights into the SAR could be gleaned that suggested optimal substituents for the rings forming the fenarimol core.

## Abstract 2

From 185 fenarimol analogues evaluated against *M. mycetomatis*, 22 were found to be potent *in vitro*, with 5 showing promise in an *in vivo* model; low predicted log *D* (<2.5) correlated with better *in vivo* efficacy, providing insights for future design.

## Graphical abstract

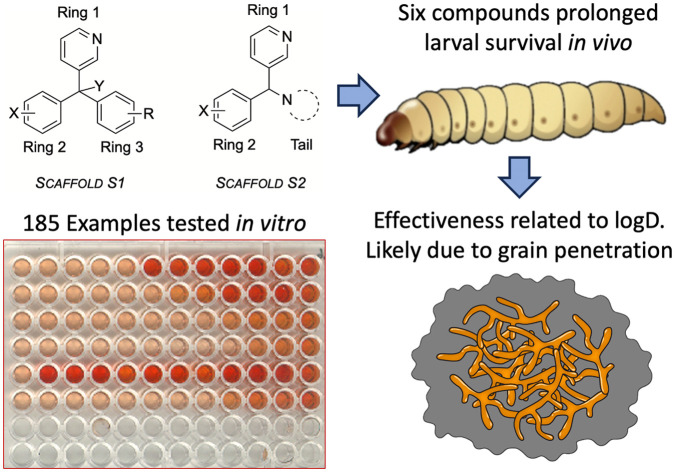

## Introduction

Mycetoma is a neglected tropical disease characterized by tumorous swellings of the subcutaneous tissues.^1^ This disease can be caused by more than 90 different agents of either bacterial or fungal origin, the most common of which is the fungus *Madurella mycetomatis*.^2^ Characteristic of mycetoma is that the causative agents organize themselves in the form of grains which protect them from stress, host responses and antimicrobials.^3–5^ Bacterial mycetoma is treated with a combination of antibiotics and often with a good cure rate. However, to treat fungal mycetoma, a combination of prolonged medication and surgery is needed. Typically, this therapy consists of 6 months of 400 mg itraconazole daily followed by surgery and another 6 to 12 months of 400 mg itraconazole daily.^6^ Surgical treatments range from excision of the lesion to amputation of the infected limb.^7^ Even after prolonged treatment, the causative agent often is viable at the time of surgery and recurrences are common.^8,9^ Other azoles have been used to treat fungal mycetoma with different degrees of success, but there are concerns over the implementation of the newer generation azoles due to low accessibility and affordability in endemic regions.^10^ Therefore, there is an urgent need to find an effective, safe and affordable drug to treat fungal mycetoma (hereafter, just “mycetoma”).

For neglected tropical diseases that lack prioritization and support in drug discovery programs, drug repurposing or repositioning studies have proven to be quite effective, due to a lowered risk and the reduced costs in discovery and development. Open source drug-repurposing studies were previously performed to discover new compounds with activity against *M. mycetomatis*.^1,11,12^ In those studies, a total of 1200 compounds from the Pathogen Box, Stasis Box and Pandemic Response Box obtained from Medicines for Malaria Venture (MMV) were first tested *in vitro*.^1,11,12^ Promising compounds from *in vitro* assessment were then tested *in vivo* for their efficacy in our *M. mycetomatis Galleria mellonella* larval model.^1,11,12^ Fenarimol analogue MMV698244, also known as EPL-BS1246, was amongst the most potent hits identified *in vitro*, with an IC_50_ of 1.35 μM. The molecule was previously identified as a potent inhibitor of the Chagas disease-causing *Trypanosoma cruzi* and it shows druglike properties suitable for preclinical development.^13–15^ Fenarimol is a fungicide that is commonly used to control powdery mildew. It acts as a potent inhibitor of ergosterol biosynthesis by interfering with the oxidative demethylation of lanosterol.^16,17^

EPL-BS1246's potency against *M. mycetomatis* led to the evaluation of another 35 fenarimol analogues, chosen from a library of 800 molecules originally developed to identify new drugs for Chagas disease;^13–15^ these were tested *in vitro* and *in vivo* for activity and efficacy against *M. mycetomatis*.^1^ Out of five fenarimol analogues tested in the *in vivo G. mellonella* model, only three were able to prolong larval survival. A correlation was observed between survival and the calculated lipophilicity (log *D* value at pH 7.4) of the fenarimol analogues and other compounds previously evaluated in this model.

Such a correlation would be useful for the future design of compounds that should exhibit better *in vivo* performance, so to explore this further we have utilized the Open Source Mycetoma project (MycetOS, https://github.com/OpenSourceMycetoma) to pinpoint the physiochemical properties needed for activity against *M. mycetomatis*. Additional analogues were designed and synthesized based on the structure and log *D* of previously tested fenarimol analogues that showed potency. Further analogues were obtained from the fenarimol library previously exploited in the original screening campaign.^1^ We report here the *in vitro* activity and *in vivo* efficacy of all the newly-obtained compounds. In line with the open source rules of MycetOS, all data and ideas have been shared publicly as the research was proceeding and anyone could participate.^18^ The project remains live online for future contributors.

## Compound numbering

In this paper, the numbering of the compounds retains the numbering used in the online MycetOS, in order to maintain the connection between this paper and the live research project. Molecules in MycetOS are numbered according to a convention described online (https://github.com/opensourceantibiotics/OSA_Tech_Ops/wiki/Molecule-Numbering-Convention). In brief, a compound's identifier takes the form of MYOS_[integer identifier including prefix zeroes]_[XX]_[YY] where XX refers to the salt form and YY refers to the batch. Codes used in this paper are the simplest integer identifier. For example, MYOS_00012_00_01 becomes 12.

## Results and discussion

In total, 185 fenarimol analogues were evaluated in this, and our previous, study. The compounds included 108 fenarimol analogues obtained from the Epichem library (of which 35 were screened in our previous study^1^ and 73 that were screened during this study) and a further 77 analogues that were synthesized for this study. To determine their potential to inhibit *M. mycetomatis* growth *in vitro*, these analogues were tested at concentrations of 100 μM and 25 μM. Of those tested, 76 analogues were able to inhibit metabolic activity of *M. mycetomatis* at 100 μM and 41 at 25 μM (SI, Table S1). To determine at which concentration 50% of the *M. mycetomatis* cells were inhibited, the IC_50_ values were determined for these 41 analogues against *M. mycetomatis* isolate mm55. Those compounds with values of 9 μM or lower were considered potent *in vitro* inhibitors and were further evaluated on a panel of nine other *M. mycetomatis* isolates with a different genetic background or geographical origin. To determine the concentrations at which 50% of these isolates were completely inhibited in growth, the MIC_50_ values were determined and these ranged from 0.25 μM to >16 μM with a median of 4 μM (SI Table S1). In total, 22 analogues exhibited potent *in vitro* activity with MIC_50_ values <9 μM; 9 and 12 were the most potent, both with a MIC_50_ of 0.25 μM.

**Fig. 1:**
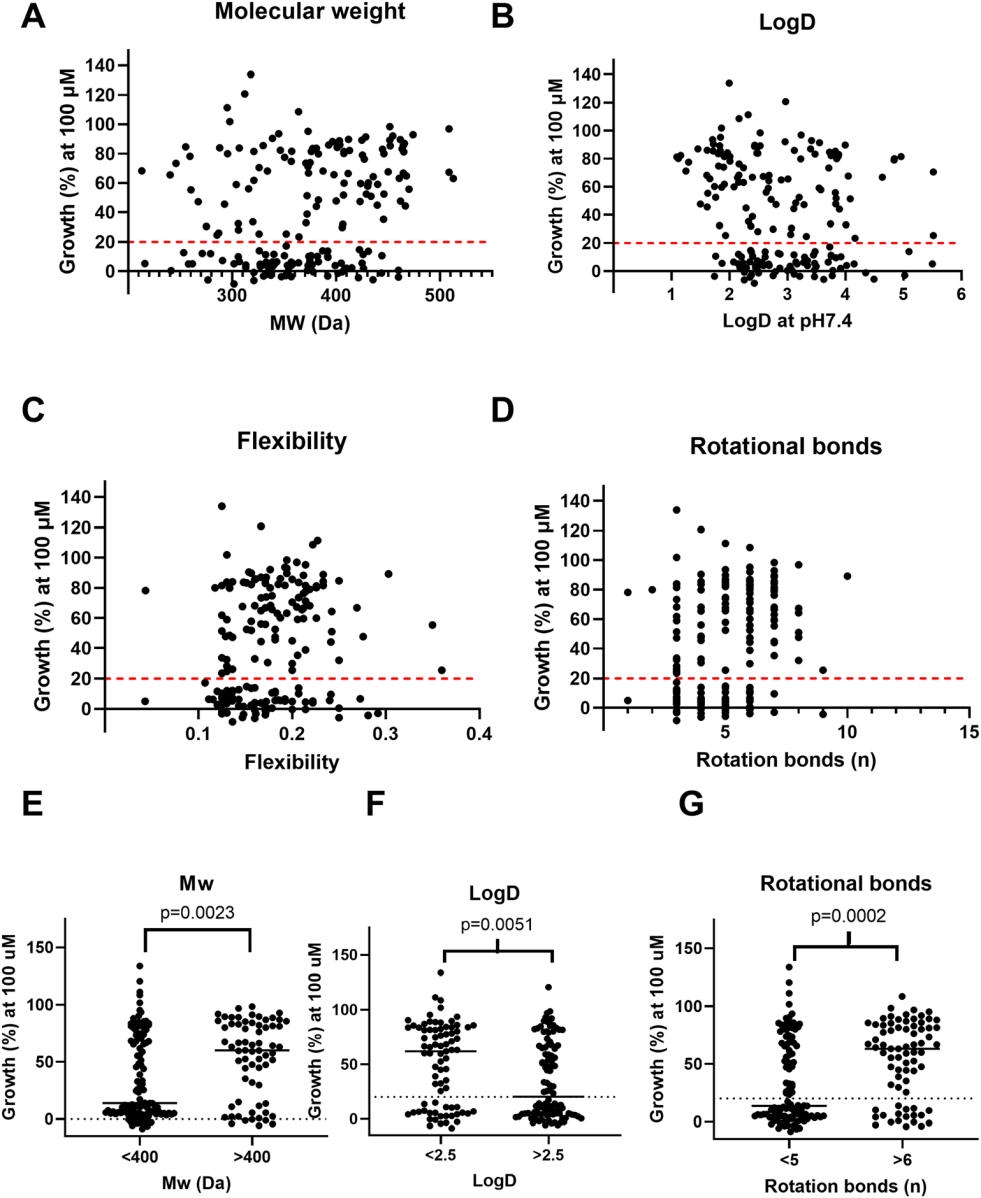
Correlation between fungal growth and physicochemical properties of the molecules tested. Percentage growth at 100 μM *vs.* molecular weight (panel A), log *D* at pH 7.4 (panel B), the flexibility of the molecule (panel C) and the number of rotational bonds (panel D). Each black dot represents a different fenarimol. In panel E, the percentage growth is plotted for compounds with molecular weight of <400 Da *versus* compounds with a molecular weight of >400. A lower percentage growth is observed when fungi are exposed to fenarimols with a lower molecular weight (Mann–Whitney, *p* = 0.0023). In panel F, the percentage growth is plotted for compounds with a log *D* value <2.5 *versus* compounds with a log *D* value >2.5. At higher log *D* values a lower percentage growth is observed (Mann–Whitney, *p* = 0.0051). In panel G, the percentage growth is plotted for compounds with 5 or less rotational bonds *versus* compounds with 6 or more rotational bonds. A lower percentage growth is noted for compounds with 5 or fewer rotational bonds (Mann–Whitney, *p* = 0.0006).

To determine if there was an association between certain physiochemical properties and the analogues' performance *in vitro*, their molecular weight (Fig. 1A), log *D* (at pH 7.4) (Fig. 1B and E), flexibility (Fig. 1C) and number of rotational bonds (Fig. 1D) were compared to the resulting percentage growth *in vitro*. Analogues with a lower molecular weight were associated with lower growth percentages (*i.e.*, more potent compounds) at 100 μM (Fig. 1E, Mann–Whitney, *p* = 0.0023). Compounds with a log *D* value >2.5 (Fig. 1F, Mann–Whitney, *p* = 0.0051) or <5 rotational bonds (Fig. 1G, Mann–Whitney, *p* = 0.0006) were associated with lower growth percentages at 100 μM.

### *In vivo* efficacy

**Fig. 2:**
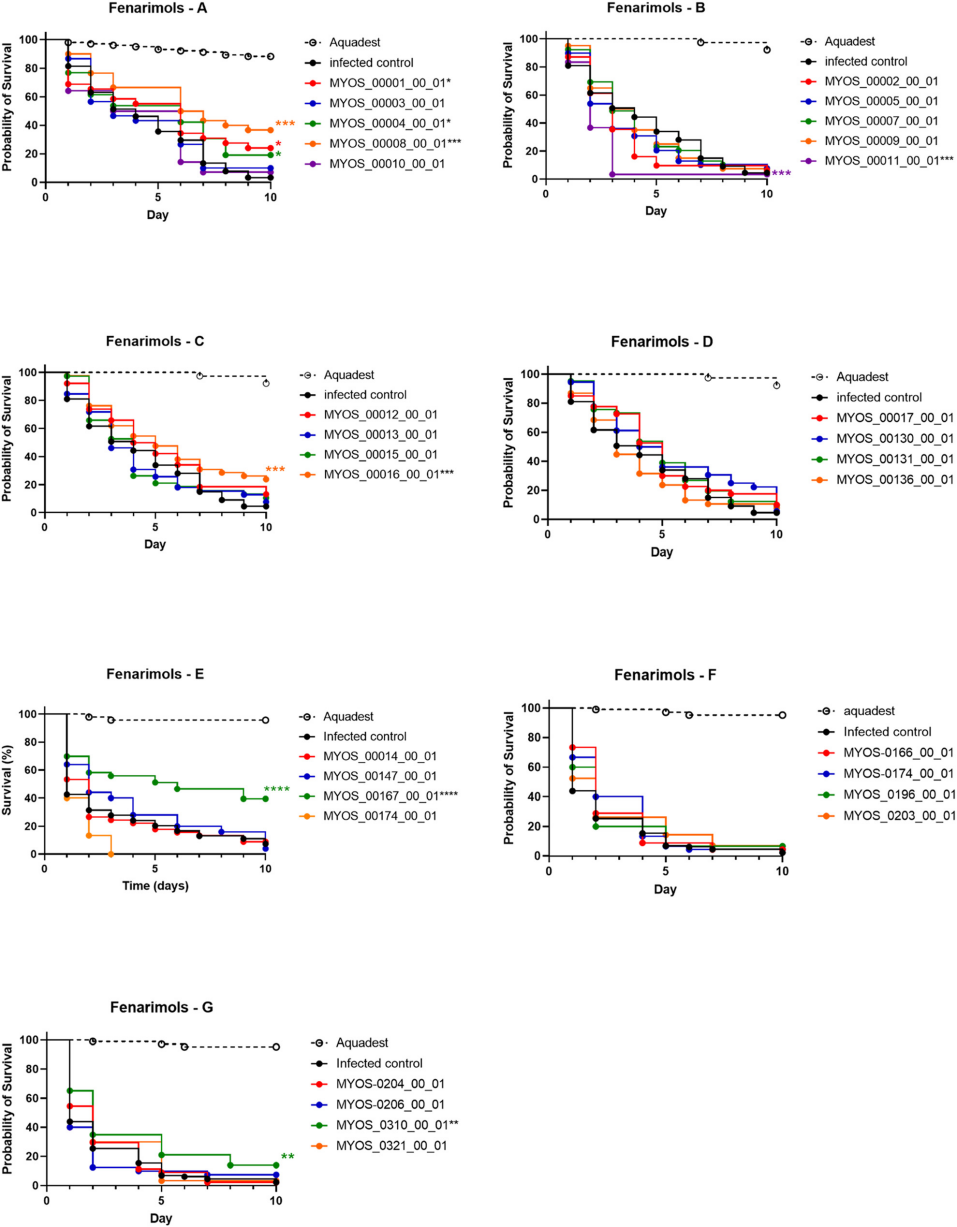
Survival of infected *Galleria mellonella* larvae treated with the fenarimol analogues. Panel A display the results obtained in our previous study,^1^ while panels B–G each display molecules selected or synthesized during this study. In each panel, the dotted line represents healthy uninfected larvae treated with solvent. The black line represents *M. mycetomatis* infected larvae treated with distilled water. The colored lines represent *M. mycetomatis* infected larvae treated with the different compounds during the first three days of infection. Survival was compared to the infected control line (black line) with the log-rank test. Compounds which significantly prolonged or decreased larval survival were indicated with an *. A single * represents a *p*-value between 0.01 and 0.05, ** *p*-value ≤ 0.01, *** *p*-value ≤ 0.001.

In total, 30 potent analogues (*in vitro* MIC_50_ values <9 μM) were evaluated in the *G. mellonella* larvae model for their *in vivo* activity. In this model *M. mycetomatis* produces grains similar to those found in human mycetoma.^19^ Furthermore, this *G. mellonella* model could predict the therapeutic outcome of itraconazole and amphotericin B in a *M. mycetomatis* grain model in mice.^20,21^ Of the 30 fenarimol analogues evaluated in this model, five had already been evaluated in a previous study^1^ and the data were used in subsequent analyses. The remaining 25 analogues were obtained, synthesized and evaluated in this study. None of the analogues displayed any toxicity at a concentration of 20 μM. In total, six of the analogues prolonged larvae survival, 23 had no significant effect and l increased the death rate (Fig. 2). The analogues that increased larvae survival were 1, 4, 8, 16, 167 and 310 (log-rank, *p* = 0.020, *p* < 0.0001, *p* = 0.044, *p* = 0.0004, *p* < 0.0001 and *p* = 0.0024, respectively). We resynthesized 1 and confirmed that it does indeed significantly prolong larval survival (log-rank, *p* = 0.015) (Fig. 2A). For 174, a trend towards shortened larval survival was noted (log-rank, *p* = 0.07) (Fig. 2E).

### Dependency of potency on physicochemical properties

**Fig. 3:**
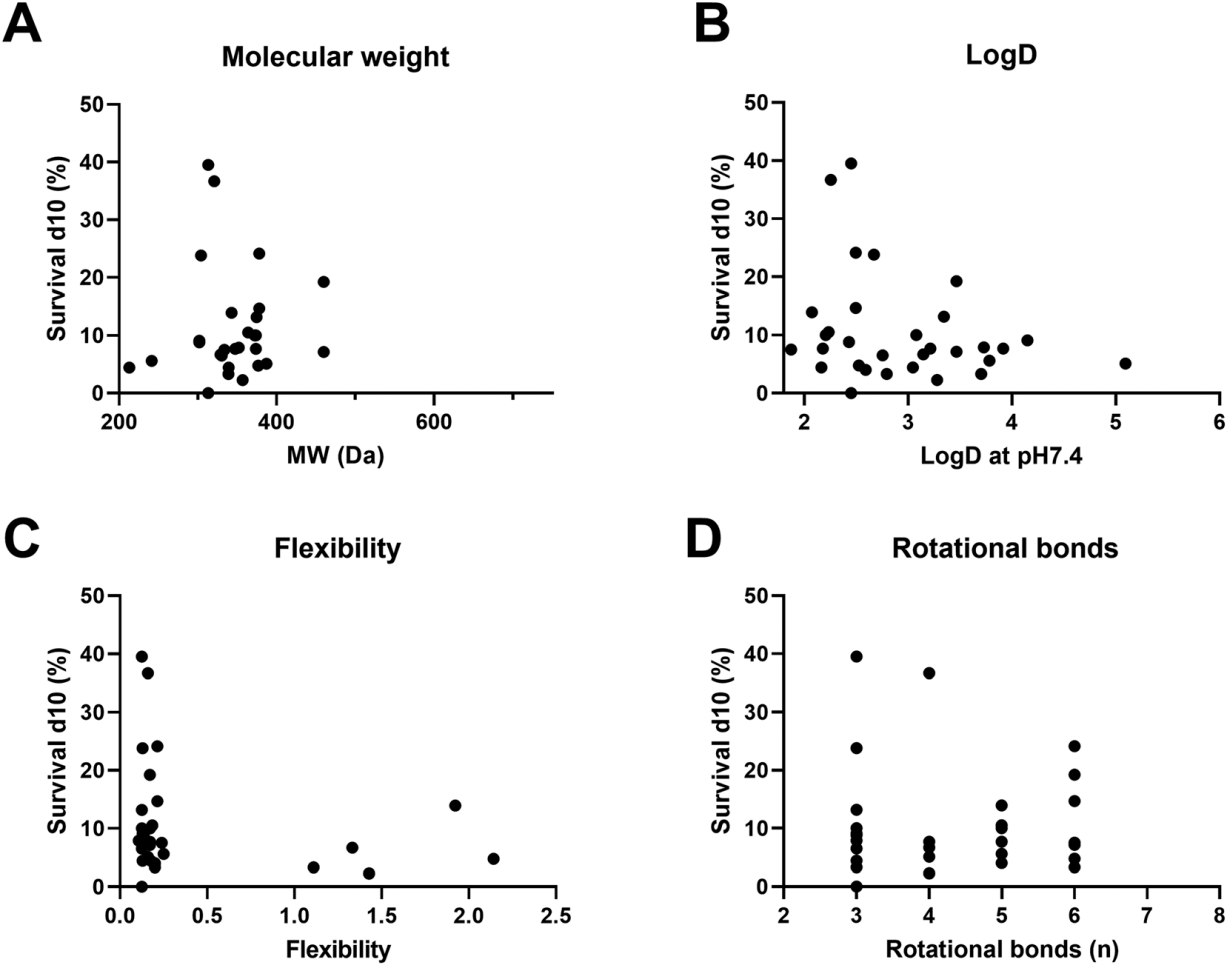
Relationship between physiochemical properties of the analogues and their efficacy *in vivo* (percentage larval survival at day 10) *vs.* panel A: molecular weight, panel B: log *D*, panel C: flexibility, panel D: number of rotational bonds.

**Fig. 4:**
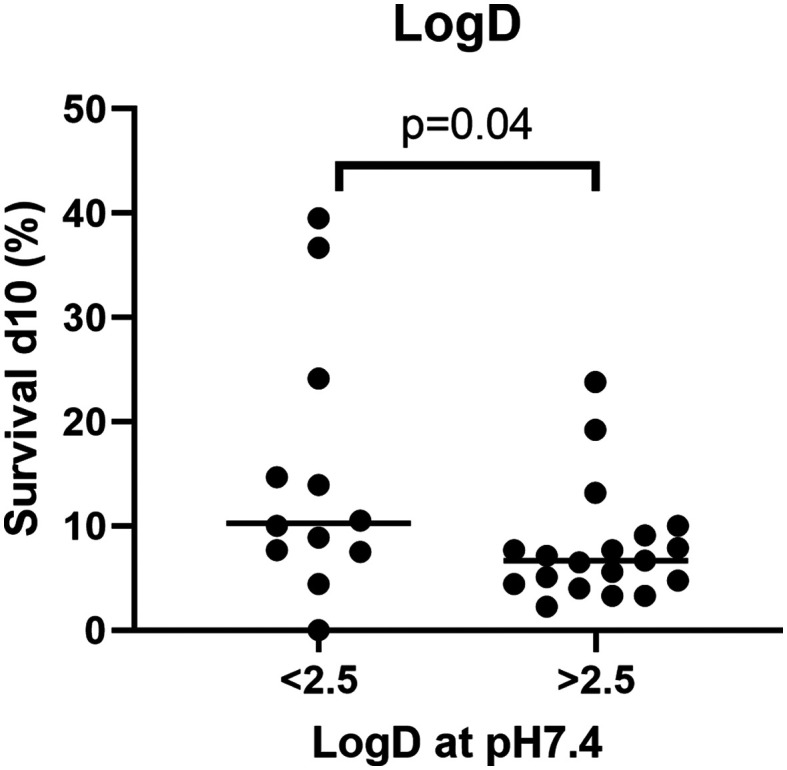
Compound log *D* < 2.5 correlated with higher percentage survival. In this graph the larval survival percentages were grouped according to their calculated log *D*. In the left column the obtained survival percentages for compounds with a log *D* < 2.5 are plotted, in the right column the obtained survival percentages for compounds with a log *D* > 2.5. Significance is determined using the Mann–Whitney *U*-test.

When comparing different physiochemical properties of the analogues to their efficacy *in vivo*, it was discovered that lower log *D* values (<2.5) were correlated with higher survival percentages in the *G. mellonella* larvae (Mann–Whitney) (Fig. 3 and 4). This suggests that compounds with a lower log *D* value are more likely to penetrate *M. mycetomatis* grains, inhibit growth and subsequently increase larval survival. Lipophilicity, here approximated by calculated log *D*, is a physiochemical parameter of a compound that affects solubility, membrane permeability, distribution and elimination of a compound or drug in the body. The higher log *D* value of a compound, the more lipophilic it is, the more likely they are to encounter off target binding to lipophilic pockets such as non-polar active sites of metabolic CYP enzymes.^22,23^ The fact that in general more lipophilic compounds were able to inhibit the metabolic activity in *M. mycetomatis* hyphae *in vitro*, but less lipophilic compounds showed a trend to prolonged larval survival demonstrates that a balance of chemical properties is likely to be needed to penetrate the mycetoma grain and kill the pathogen. This will be an important criterion for future rounds of analogue design.

### Structure–activity relationships

**Fig. 5:**
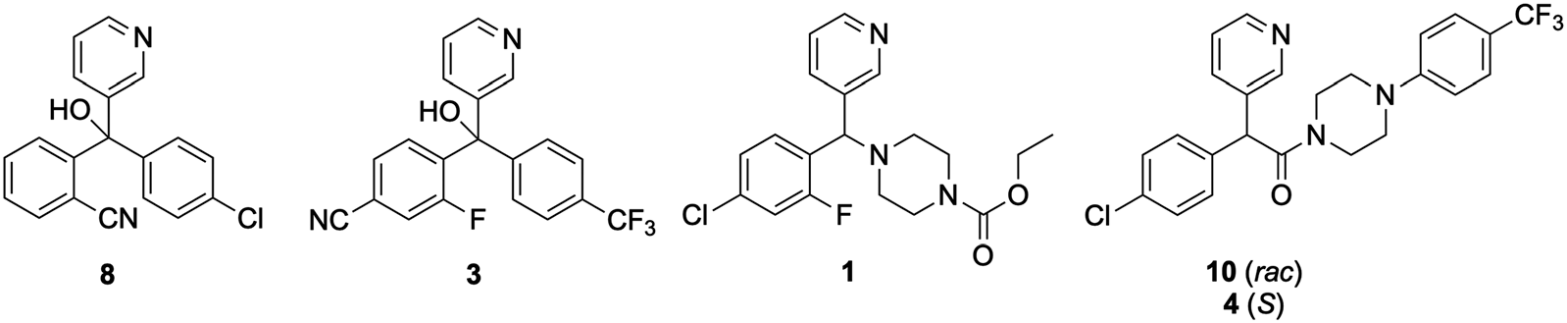
The best-performing compounds in the fenarimol series (“series 1”) previously identified.

A previous study found the potent inhibition of *M. mycetomatis* by the fenarimol analogue 10 and further screening of 35 other fenarimol analogues revealed four other potent leads (Fig. 5) showing diverse chemical groups that could contribute to potency.^1^ Analysis of activity for these 35 compounds did not produce conclusive SAR observations, which was attributed to the diversity of structures screened.^1^

The present study expanded on these preliminary findings by conducting an SAR analysis on 196 series 1 fenarimol analogues biologically evaluated in the Open Source Mycetoma project, and which were contributed from various sources (details of the contributions are available in SI Table S1 and the online Master List of compounds (which is updated in real time)).

**Fig. 6:**
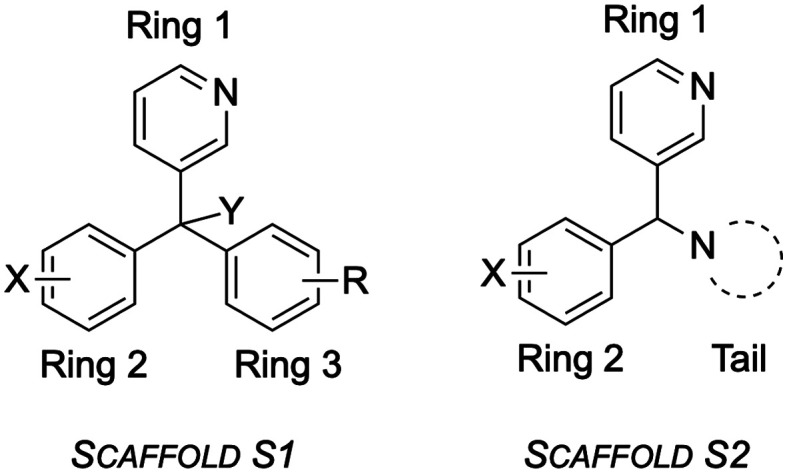
The fenarimol analogues may be grouped as two scaffolds, S1 and S2.

The compounds were generalized into two scaffolds (S1 and S2, Fig. 6). Scaffold S1 consisted of three aromatic rings along with an additional functional group (Y) giving a quaternary carbon. Of the three rings, one (ring 1) was typically pyridyl or pyridyl-like, one (ring 2) was typically substituted with a halogen and one (ring 3) featured a lower variety of substitution and may be aliphatic. The second scaffold, S2, featured similar groups for rings 1 and 2, but the third substituent, the tail, consisted of more saturated groups usually connected to the core, a tertiary carbon, *via* a nitrogen atom. Analogues were grouped into S1 or S2 and the analysis focussed on identifying matched pairs of analogues where single changes resulted in different potencies. The compounds analysed included changes that had previously been proposed,^1^ including modification of the pyridyl group (ring 1), modification of the core (Y), variation of substitution patterns (rings 2 and 3), variation of ring aromaticity, variation of the ring 3 tail and conformational restriction through ring-locking. Values for the *in vitro* and *in vivo* potencies for all compounds are provided in SI Table S1 and in the online Master List^24^ for the Open Source Mycetoma Consortium.

Compounds have been evaluated for *in vitro* efficacy as judged by observed fungal growth at 100 μM and 25 μM dosages, half-maximal inhibitory concentration (IC_50_), and minimum inhibitory concentration (MIC_50_). The observed fungal growth at 25 μM and MIC_50_ were selected to explore *in vitro* SAR trends as these were the threshold values determining whether an analogue would be selected for further evaluations. At 25 μM, a value of ≤20% observed fungal growth was defined as no observed growth (complete inhibition) and >20% was defined as observable growth (incomplete inhibition). All analogues which resulted in no observed growth at 25 μM were selected for further *in vitro* evaluation (IC_50_ and MIC_50_). Additionally, all analogues with MIC_50_ ≤ 8.0 μM were selected for *in vivo* evaluation.

Of the examined analogues, using the above criteria, fifty completely inhibited fungal growth and 145 compounds did not inhibit fungal growth. Of the 61 analogues evaluated for MIC_50_, 38 compounds had MIC_50_ values ≤8.0 μM and 23 compounds had MIC_50_ values >8.0 μM. To facilitate evaluation of the SAR, a traffic light visualization was implemented where **green** denoted compounds with “excellent potency” (≤20% growth at 25 μM and MIC_50_ ≤ 8.0 μM), **amber** denoted compounds with “moderate potency” (≤20% growth at 25 μM but MIC_50_ > 8.0 μM) and **red** denoted compounds with “poor potency” (>20% growth at 25 μM and MIC_50_ > 8.0 μM). This classification returned **35 compounds** with excellent potency, **12 compounds** with moderate potency, and **145 compounds** with poor potency. The following subsections explored the SAR of chemical space within the S1 and S2 scaffolds, including ring 1, core Y, ring 2 and ring 3/tail positions.

## Scaffold S1 *in vitro* results

### SAR exploration of ring 1 and core Y

**Fig. 7:**
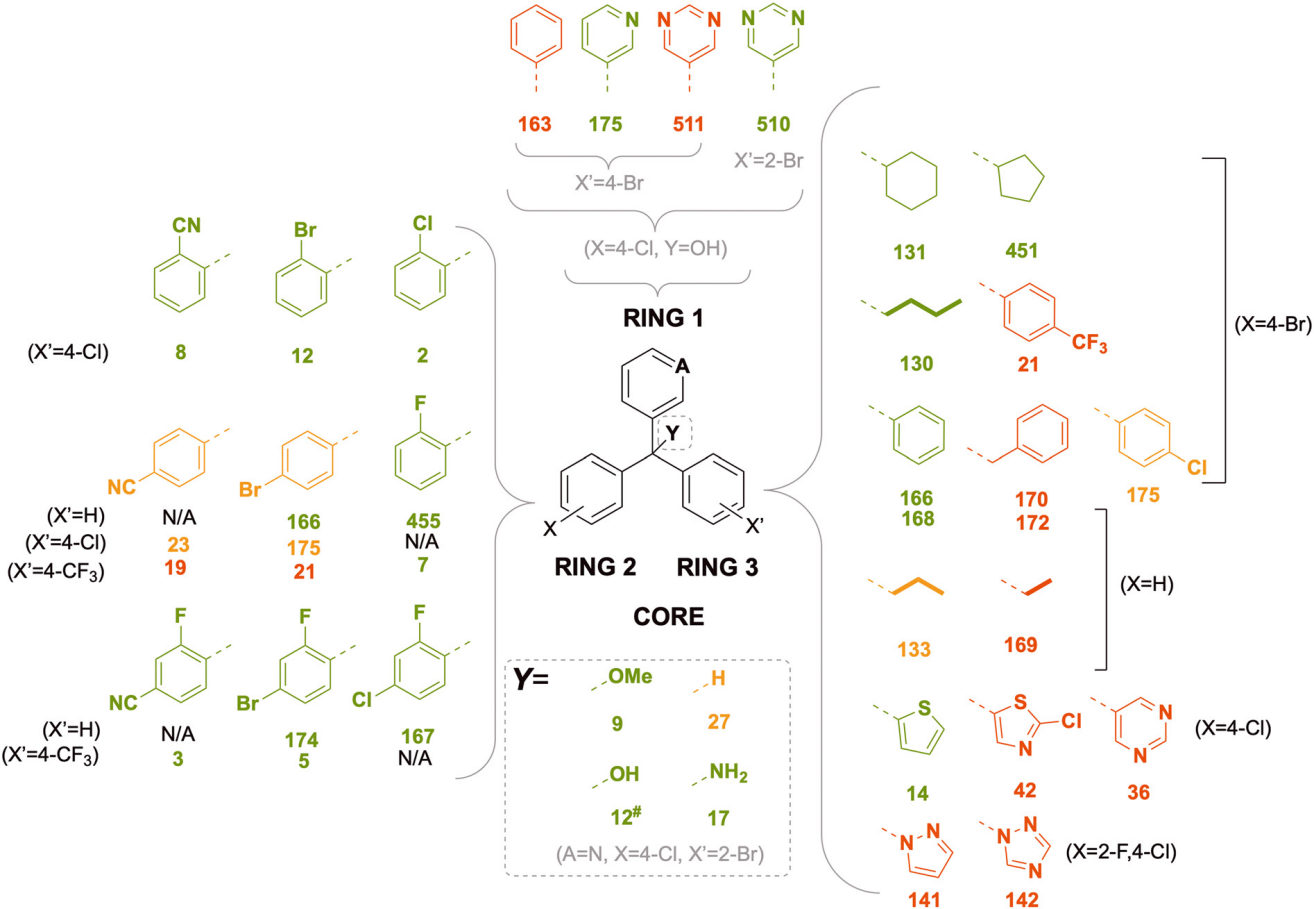
Scaffold S1 variants and the corresponding *in vitro* inhibition of *M. mycetomatis* growth. High (green) is defined as complete inhibition (≤20% fungal growth) at 25 μM and MIC ≤ 8.0, moderate (amber) as complete inhibition at 25 μM and MIC > 8.0, low (red) as incomplete inhibition (>20% fungal growth) at 25 μM and MIC > 8.0. For matched pairs from rings 2 and 3, ring 1 is kept constant as 3-pyridyl and the core is kept constant as Y = OH. Parentheses denote the substitution patterns that were unchanged when a single SAR change was explored.

For ring 1, the 3-pyridyl group (175) displayed better potency than the phenyl (163) or 5-pyrimidyl (511) groups when ring 2 was kept as 4-chlorophenyl, ring 3 as 4-bromophenyl and the core as Y = OH (Fig. 7, ring 1); moving the bromine substituent to the 2- (rather than the 4-) position reclaimed potency for the pyrimidine substituent (510). Varying the core's quaternary centre (keeping ring 1 as 3-pyridyl, ring 2 as 4-chlorophenyl and ring 3 as 2-bromophenyl) revealed that diverse functional groups able to undergo hydrogen bonding (12 (Y = OH), 17 (Y = NH_2_), 9 (Y = OMe)) produced better activity than the analogue featuring only a hydrogen atom at this position (27). With these results in mind, variations in ring 2 and ring 3 were explored while keeping ring 1 as 3-pyridyl and the core as Y = OH.

### SAR exploration of ring 2

Various substituents at the 2-position on ring 2 (8, 2-CN; 12, 2-Br; 2, 2-Cl; Fig. 7, ring 2) gave excellent activity while 4-monosubstitutions of the same groups reduced potency (23, 4-CN; 175, 4-Br) (ring 3 was kept constant as 4-chlorophenyl). 2-Fluorination (455, 2-F) or where this fluorine is combined with other substituents in the 4-position on ring 2 (3, 2-F, 4-NC, 5, 2-F, 4-Br) led to better potency than those compounds lacking the 2-F substituent (19, 4-NC, 21, 4-Br). Overall, the SAR for ring 2 suggests strong potency for 2- or 2,4-substituted phenyl groups at this position, while showing lower potency for mono 4-substitution. The effect of 3-substitution was not explored in this work but could be addressed in future.

### SAR exploration of ring 3

When ring 2 was 4-bromophenyl, a phenyl group at ring 3 gave excellent activity (166), with potency maintained when the aromatic group was changed to alicyclic (131, 451) or aliphatic (130) groups (Fig. 7, ring 3). The potency was lost when ring 3 was changed from phenyl to benzyl (166*vs.*170). 4-Substitution patterns on ring 3 also resulted in moderate (175, 4-Cl) to complete (21, 4-CF_3_) loss of activity. This result, combined with SAR on ring 2, suggests poor tolerance of 4-trifluoromethyl (4-CF_3_) phenyl group, as well as the combination of 4-substituted phenyls on both rings 2 and 3 being deleterious.

When ring 2 was an unsubstituted phenyl group, a phenyl group on ring 3 still resulted in excellent potency while a change to benzyl again caused a complete loss of activity (168*vs.*172). This time, changes from aromatic ring 3 to aliphatic groups were not tolerated (168*vs.*133, 169). When ring 2 was 4-chlorophenyl, a thiophenyl group at ring 3 (14) showed superior potency while other heteroaromatic groups were not tolerated (42, 36, 141, 142).

### Refinement of SAR for rings 2 and 3

**Fig. 8:**
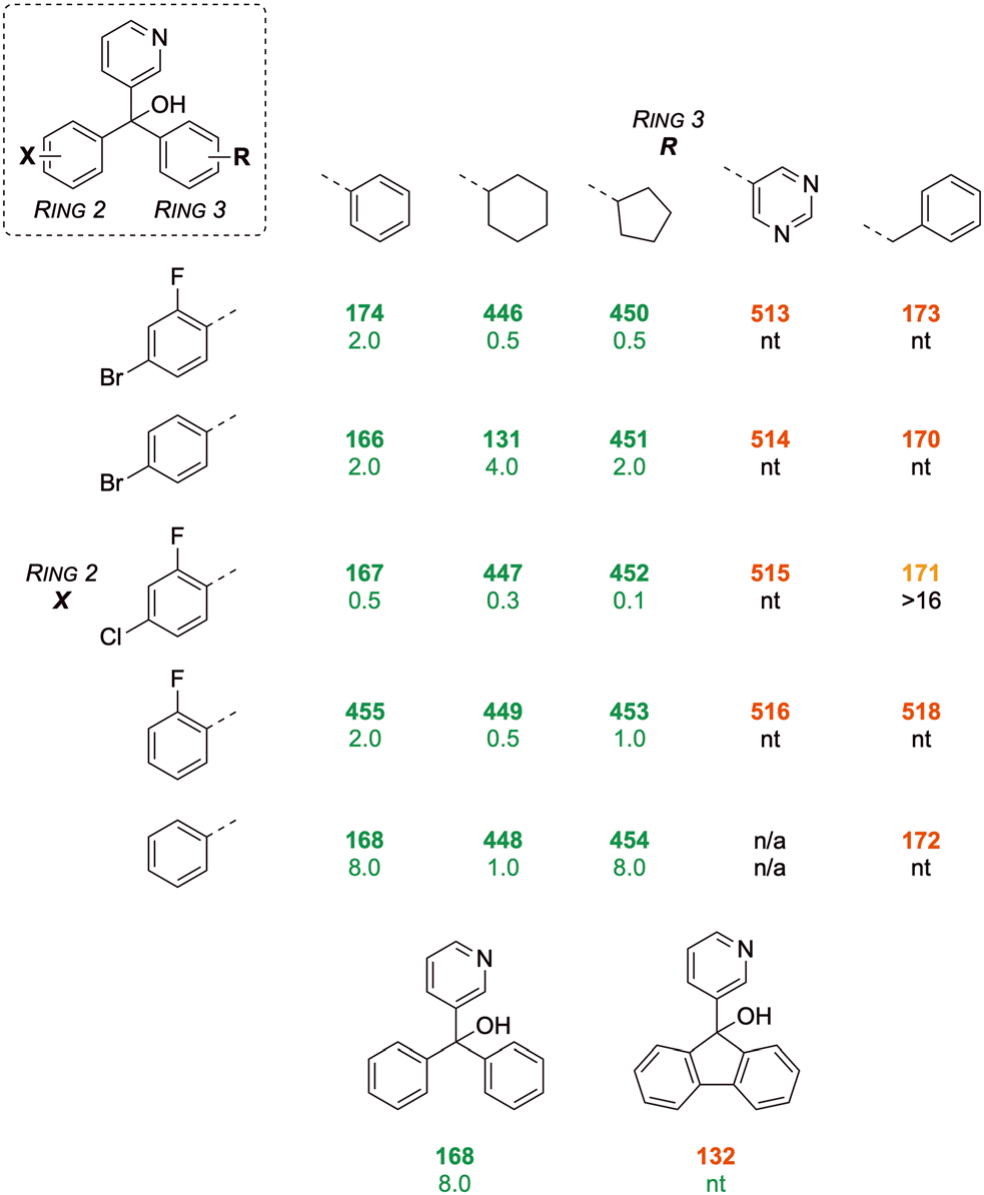
Focussed matched pairs of ring 2 and ring 3 variations. Ring 1 was maintained as 3-pyridyl and core Y was maintained as hydroxyl. The numbers shown are compound codes (bold; colouration as for Fig. 7) and MIC_50_ values (nt = not tested). Also shown are compounds 168 and 132 indicating that ring-locking caused loss of *in vitro* activity. n/a = compound not made. nt = not tested.

Since some of the early SAR data were acquired with suboptimal substituents (*e.g.*, ring 3, 4-Br), further analogues were synthesized to clarify the trends (Fig. 8) clearly showing the preference for ring 2 2-substitution and for the direct attachment of non-polar rings to the core as ring 3. The 2-fluoro 4-chloro substitution on ring 2 yielded the most potent MIC_50_ values for the phenyl (455, MIC_50_ = 0.5 μM), cyclohexyl (449, MIC_50_ = 0.3 μM) and cyclopentyl (452, MIC_50_ = 0.1 μM) ring 3 variants. Locking of ring 2 and ring 3 resulted in inferior potency (168*vs.*132, Fig. 8).

## Scaffold S2 *in vitro* results

**Fig. 9:**
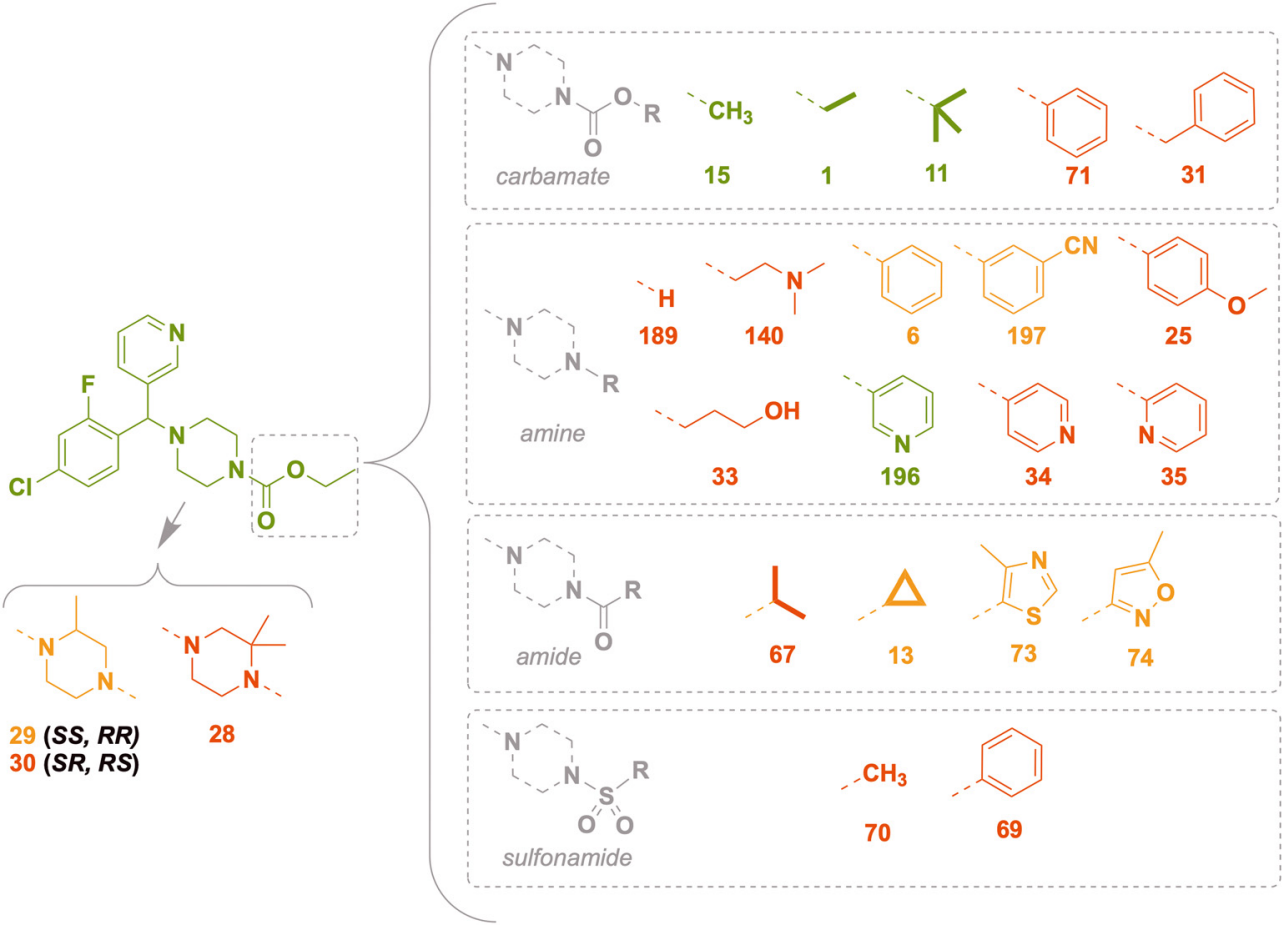
Scaffold S2 piperazyl variants and the corresponding *in vitro* inhibition of *M. mycetomatis* growth. Colouration used is as for Fig. 7.

Compounds represented by scaffold 2 feature similar groups to scaffold S1 for rings 1 and 2, but the third substituent consists of more saturated groups connected to the central carbon *via* a nitrogen atom (Fig. 9). Structures were explored with variation in the third substituent while keeping groups at ring 1 as 3-pyridyl and ring 2 as 4-chloro-2-fluorophenyl, both of which demonstrated superior activity for scaffold 1 (the full set of screened compounds is shown in SI Table S1 and in the online Master List^24^).

### Scaffold 2 – ring 3 piperazyl SAR

Keeping the ethyl-carbamate group constant (1), the piperazyl group showed excellent potency which was lost upon ring mono- (29, 30) and di- (28) methylation (Fig. 9). Compound 29 (*SS*, *RR* enantiomers) was more potent than 30 (*SR*, *RS*), revealing a differential impact of stereoisomeric forms on the activity. When the un-methylated piperazyl group was kept constant, alkyl **R** groups on the carbamate had superior activity (15, methyl; 1, ethyl, 11, *tert*-butyl) to aromatic groups such as phenyl (71) or benzyl (31). Removing the ester moiety of the carbamate caused a complete loss of activity (189, Fig. 9, amine box) and indeed all other changes to the amine–R group led to inferior activity, including polar aliphatic (140, 33) and aromatic groups (6, phenyl; 25, 197 substituted phenyl; and heteroaromatic 34, 35) with the exception of the 3-pyridyl group (196). A smaller subset of compounds that replaced the carbamate with amide or sulfonamide linkers also yielded medium to poor activity.

### Scaffold 2 – ring 3 piperidyl SAR

**Fig. 10:**
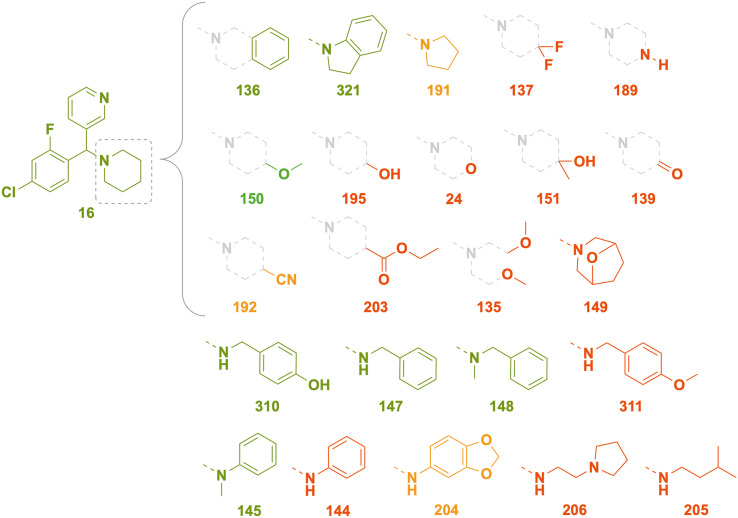
Scaffold S2 piperidyl variants and the corresponding *in vitro* inhibition of *M. mycetomatis* growth. Colouration used is as for Fig. 7.

At the third substituent position of scaffold S2, changing the piperazyl (189) to a piperidyl group (16) maintained excellent activity (Fig. 10). Further expanding the piperidyl ring to a tetrahydroisoquinoline (136) or indolinyl (321) moiety maintained potency but the smaller pyrrolidyl ring (191) and the larger bridged bicyclic substituent (149) yielded lower activity. Variants of 4-substitution on the piperidyl ring generally resulted in inferior activity, such as cyano- (192), difluoro (137), hydroxy (151, 195) and carbonyl (139); 4-methoxy (150) was an outlier, with good potency. Changing the 4-carbon to heteroatoms such as nitrogen (189) or oxygen (24) was not tolerated. The “open ring” equivalents of 136 and 321 (*i.e.*, 148 and 145) maintained potency but several simple variants of these (*e.g.*, anilines 144, 204) did not; benzylamines 147 and 310 were potent, while the orthologous 4-methoxy substituted ether 311 was not. Conversion of the carbamate 1 into the ester (203) reduced potency. Acyclic aliphatic amine substituents (205, 206) were not tolerated.

### SAR exploration of ring 2 extension

**Fig. 11:**
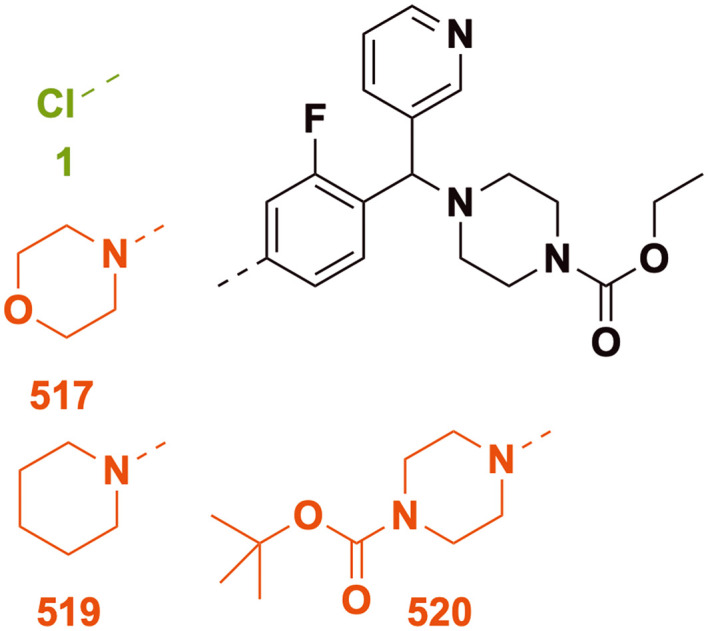
Scaffold S2 ring 2 extension and the corresponding *in vitro* inhibition of *M. mycetomatis* growth. Colouration used is as for Fig. 7.

A small set of variants of the 4-chloro substituent on ring 2 was explored (Fig. 11). While the 4-chloro variant (1) had excellent potency, the three variations (morpholinyl (517), piperidyl (519) and *tert*-butyl piperazine-1-carboxylate (520)) caused loss of activity.

## Scaffold S1 *in vivo* results

**Fig. 12:**
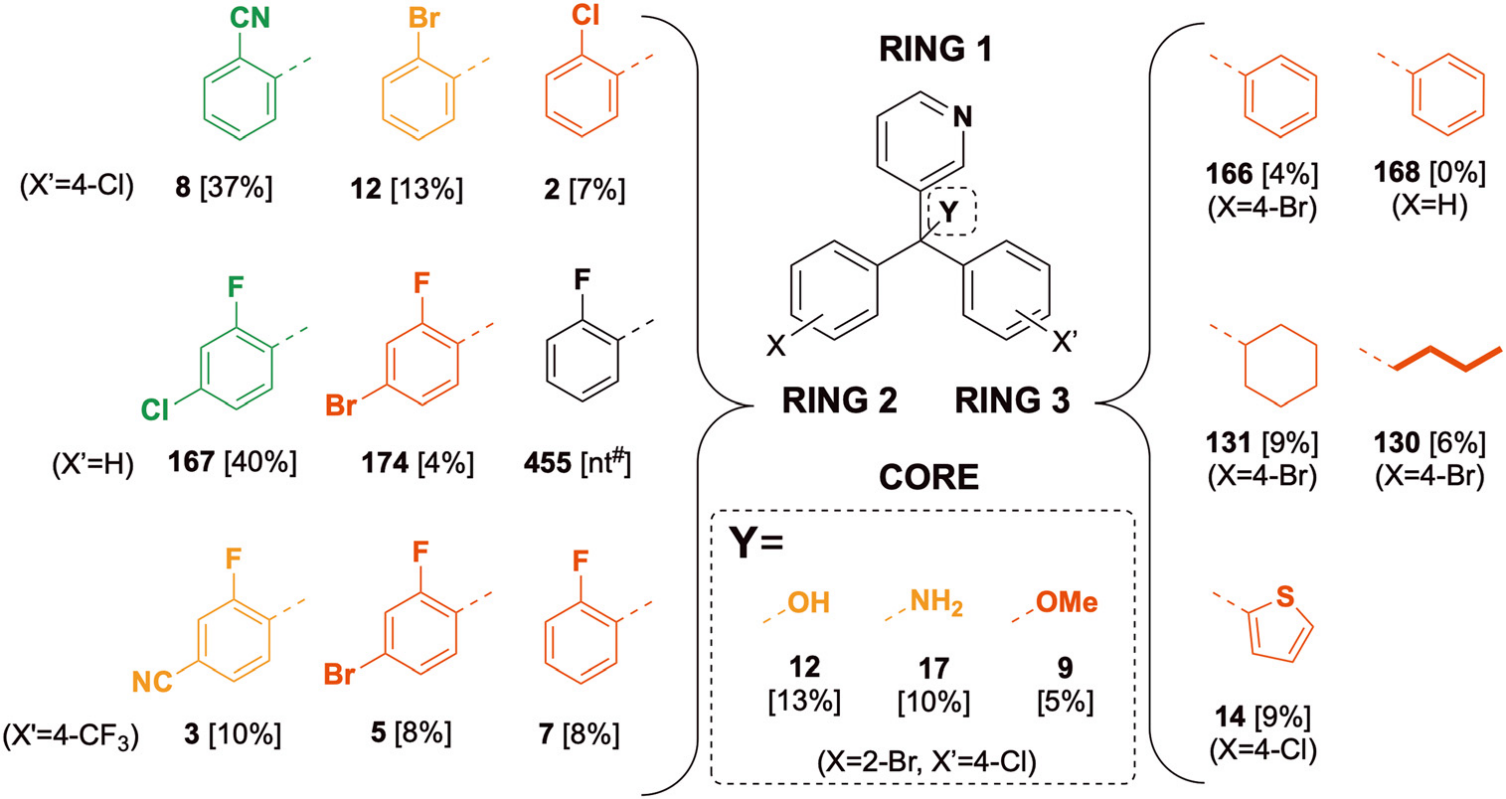
Scaffold S1 *in vivo* activity, showing larval survival rate (%) at day 10. ^#^Compound 455 showed undesirable increased *in vitro* fungal at higher dosage and was excluded from *in vivo* screening. Parentheses denote the substitution patterns that were unchanged when a single SAR change was explored. Compounds leading to survival rates >20% are shown in **green**, 10–20% **amber** and <10% in **red**.

Compounds that yielded excellent *in vitro* activity (complete inhibition of *in vitro* fungal growth at 25 μM, with a minimum inhibitory concentration less than 8.0 μM) were progressed to *in vivo* evaluation in the *G. mellonella* larvae model described above. All such analogues contained the 3-pyridyl group at ring 1 (Fig. 12). At the core, the hydroxy group (12, Y = OH) had the highest activity, which was slightly reduced when changed to an amino group (17, Y = NH_2_) and further reduced when changed to a methoxy group (9, Y = OMe) (ring 2 fixed as 2-bromophenyl, ring 3 as 4-cholorophenyl). The hydroxy group at the core was maintained when exploring the remaining SAR for this section.

For ring 2, the 2-cyanophenyl group (8) showed superior activity to 2-bromo or 2-chlorophenyl groups (12, 2), when ring 3 had a 4-chloro substitution. Disubstituted 4-chloro-2-fluorophenyl group (167) was more potent than 4-bromo-2-fluorophenyl group (174) when ring 3 was an unsubstituted phenyl group. Compound 455 was not tested *in vivo* because the *in vitro* results showed an undesirable increase in fungal growth at higher concentration. When ring 3 was changed to 4-trifluoromethylphenyl, the disubstituted 4-cyano-2-fluorophenyl group (3) at ring 2 had a slightly higher activity than 4-bromo-2-fluorophenyl (5) and losing 4-substition here did not reduce activity any further (7).

For ring 3, the unsubstituted phenyl group (166) showed inferior activity to a cyclohexyl group (131) and a straight-chain butyl group (130), when ring 2 was a 4-bromophenyl. Compound 131 showed the strongest activity amongst the three compounds. Removing all substitutions on phenyl rings 2 and 3 caused a complete loss of activity (168*vs.*166). When ring 2 became 4-chlorophenyl, having a thiophene on ring 3 (14) did not yield superior activity.

## Scaffold S2 *in vivo* results

**Fig. 13:**
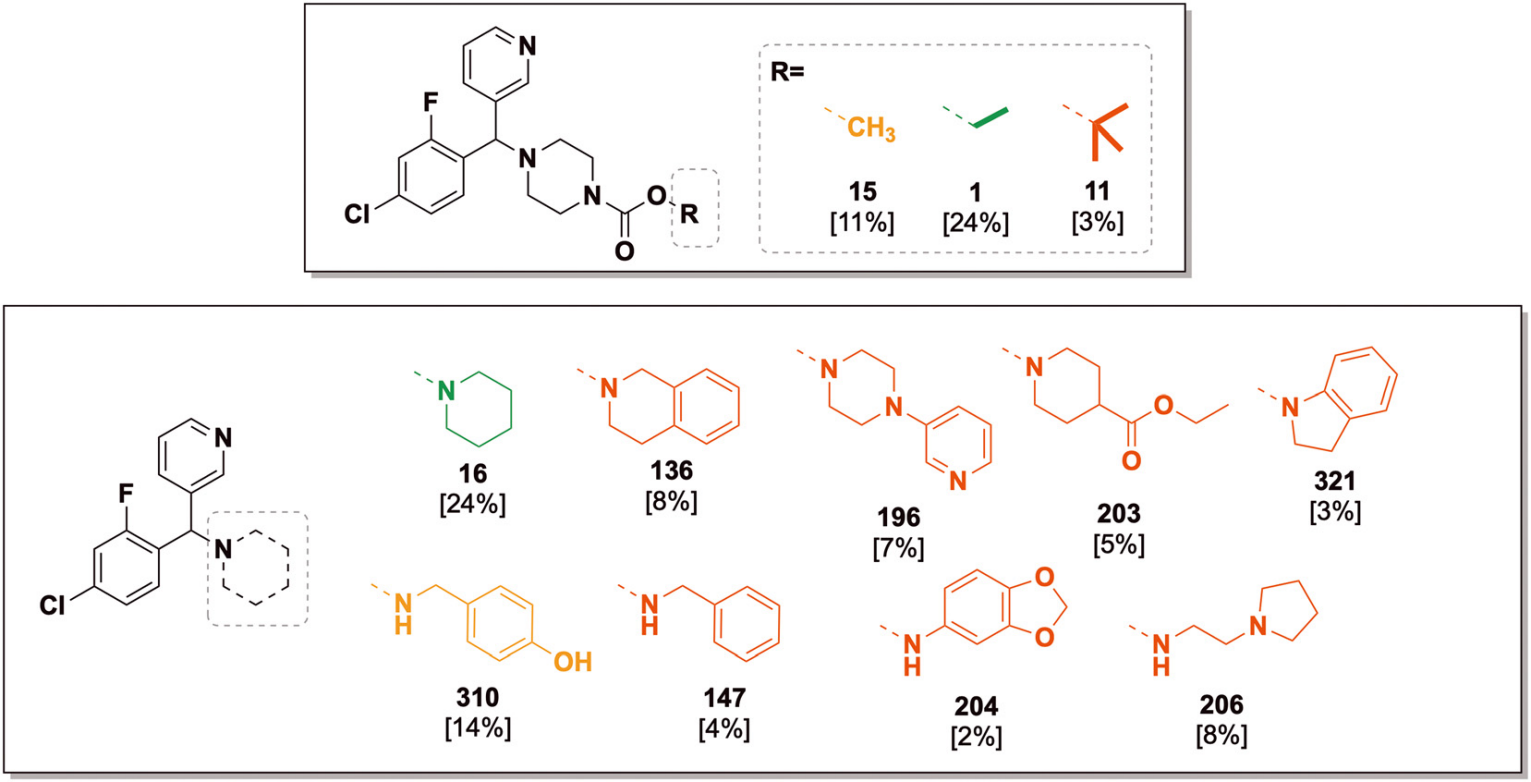
Scaffold S2 *in vivo* activity, showing larval survival rate (%) at day 10.

For scaffold S2 with a piperazyl moiety at ring 3, the carbamate R-group with highest activity was an ethyl group (1), with activity reduced when the R-group became shorter (15, R = methyl) or more bulky (11, R = *tert*-butyl) (Fig. 13, top box). The plain piperidyl moiety at ring 3 had comparable activity to compound 1 which was reduced when there was an attached aromatic ring (136, tetrahydroisoquinoline); the open ring analogue (310) performed reasonably well, while similar variants (147, 148) without a hydroxy group had significantly reduced *in vivo* activity. The best activities observed are comparable to the best previously seen for compound 4 (Fig. 5, 19%), which was more potent than the corresponding racemate (7%), implying that it may be productive to evaluate the activities of enantioenriched 1 and 16.

### Summary of key SAR findings and gaps

The SAR analysis showed high *in vitro* potency for 3-pyridyl group at the ring 1 position (scaffolds S1 and S2) and 2,4-disubstituted phenyl group at ring 2 with the 2-fluoro-4-chloro pattern most frequently present in potent analogues (S1 and S2). Potency tolerated diverse motifs at ring 3/tail positions (S1 and S2) including phenyl, thiophenyl, alicyclic (cyclohexyl and cyclopentyl) and aliphatic (butyl), piperazyl and piperidyl groups. Furthermore, *in vivo* potent analogues typically had a 3-pyridyl group at ring 1 (S1 and S2), a hydroxyl core Y (S1) and 2,4-disubstituted phenyl groups at ring 2 (S1 and S2). Monosubstituted phenyl group at ring 2 showed inferior *in vivo* potency compared to their disubstituted matched pairs (S1). For scaffold S2, the ethyl carbamate piperazyl group and the piperidyl group at the ring 3 tail position showed potent *in vivo* activity. Two other S2 analogues with potency *in vivo* (4 (Fig. 5) and 310 (Fig. 13)) without a piperazyl or piperidyl motif were found, but matched pairs to these analogues were not explored during the present campaign. Compounds that can be explored in future include 3-monosubstituted and 3,5-disubstituted phenyl ring 2 (S1 and S2), and disubstitution patterns simultaneously present on both ring 2 and ring 3 (S1). Matched pairs would be beneficial to further elaboration of SAR for ring 3/tail positions (S1 and S2).

## Concluding remarks and future directions

From the 196 fenarimol analogues evaluated *vs. M. mycetomatis*, five analogues exhibited good *in vivo* activity in the *G. mellonella* model (8 and 167 from S1 and 1, 16 and 4 from S2), suggesting they may have promise as starting points for optimization projects aimed at the development of new drug leads for the treatment of mycetoma. Importantly, our results point to a delicate balance needed in the log *D* of any drug candidate. While compounds with log *D* values greater than 2.5 tended to perform better in the *in vitro* hyphae assay, compounds with log *D* values lower than 2.5 appeared to be associated with improved larval survival in the *in vivo* assay. This correlation in based on calculated log *D* values; it will be important to validate the correlation through the measurement of log *D* for selected compounds.

The pharmacokinetic properties of scaffold S1^14^ and scaffold 2^15^ compounds were evaluated in the manuscripts originally reporting their activity *vs. T. cruzi*, so this has not been pursued again as part of this study, but it was found that kinetic solubility was low to moderate (range of *ca.* 1 to 100 μg mL^−1^ at pH 6.5) and the compounds exhibited medium to high levels of intrinsic clearance in human and rat liver microsomal assays. A good *in vitro*–*in vivo* correlation was observed, with selected members of the original library exhibiting good plasma exposures in mice following oral administration. It was reported that, perhaps unsurprisingly, there was no correlation between potency *vs.* the pathogen and *in vivo* efficacy, but these data provide supporting evidence for the overall potential of these scaffolds as the basis for new treatments for mycetoma.

Should molecules in this series be identified with suitable *in vitro* potency as well as potency in the *in vivo* model, progression would depend upon acceptable selectivity over host cells, good *in vitro* clearance and good solubility data; these would in turn allow reasonable free exposure in an *in vivo* PK study in mouse or rat.

### CYP51 inhibition as a probable mechanism of action

The SAR findings presented in this study support the idea that sterol 14α-demethylase (CYP51) inhibition is the likely mechanism of action for fenarimol analogues against *M. mycetomatis*; in the absence of a crystal structure of this protein bound to a fenarimol it is possible to form a preliminary conclusion based on the available literature on CYP51 inhibition by azole and fenarimol analogues for other pathogens, helping future compound design.

CYP51 is a cytochrome P450 (CYP) enzyme that catalyses the removal of 14α-methyl group from sterol precursors.^25,26^ Found in all biological kingdoms, CYP51 is essential for the biosynthesis of sterols, such as cholesterol in humans and ergosterol in fungi. CYP51-mediated sterol production plays an important role in maintaining cell membrane integrity and signalling pathways, making it a major drug target for antifungal treatment by azoles,^27–29^ one of the largest classes of antifungal drugs in clinical use.^30,31^ Azoles inhibit CYP51 through coordination of the heme group in the active site pocket, lowering the reduction potential of CYP51 and preventing cleavage of 14α-methyl from the sterol precursors.^32,33^ Additionally, azoles form non-coordinating interactions with amino acid residues in the active site and around the access channel into the active site, thus competing with the sterol substrates.

**Fig. 14:**
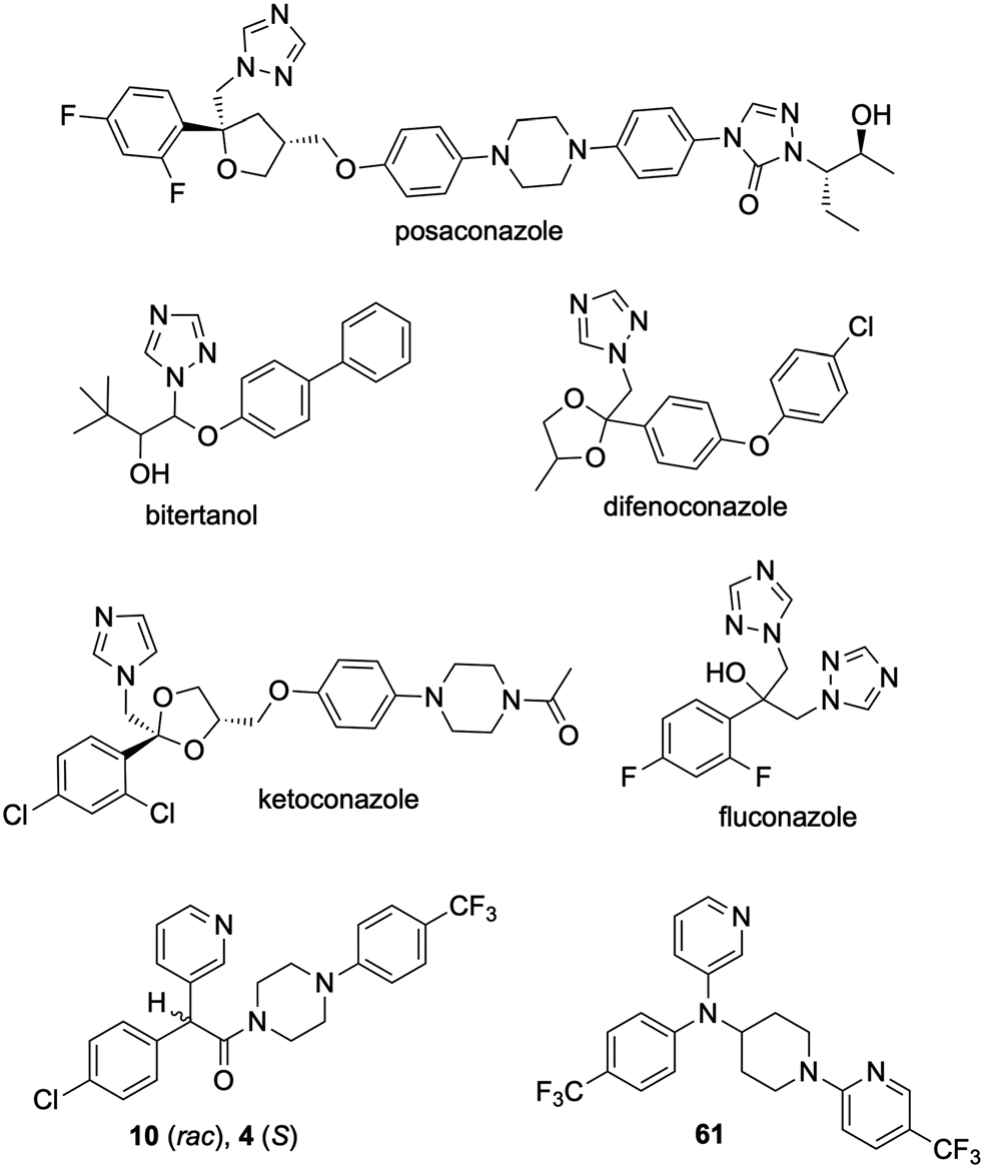
Examples of azoles (posaconazole, ketoconazole and fluconazole) and fenarimol analogues (4, 10 and 61) with known CYP51-bound complexes.

Several azoles and fenarimol analogues are known to inhibit CYP51 (Fig. 14). Our initial fenarimol analogue drug discovery study identified 13 compounds with potent activity against mycetoma, seven of which had known mechanisms of action for other pathogens and four were from the CYP51 inhibitor class.^1^ Three of the CYP51 inhibitors were the azole antifungals posaconazole, bitertanol and difenoconazole, and one was the antiprotozoal EPL-BS1246, the lead fenarimol analogue (10) target for the present project. Posaconazole was shown to bind to CYP51 of *T. cruzi* (protozoan)^34^ and *C. albicans* (fungus),^32^ fluconazole was shown to bind to CYP51 of *M. tuberculosis* (bacterium)^35^ and *T. cruzi*^34^ and, in a homology model study, ketoconazole was shown to bind to *A. fumigatus* (fungus) CYP51.^36^ These CYP51 inhibitors feature common structural motifs such as an aromatic ring (5-membered or 6-membered) with one or more nitrogen atoms, a halogenated phenyl ring and a longer “tail”, features that map well to the active core of the fenarimols in the present study.

Studies on posaconazole bound to CYP51 of *T. cruzi* (protozoan) and *C. albicans* (fungus) showed the active site fully encapsulating the triazole ring, the dihalogenated phenyl ring and the proximal portion of the tail, while the access channel into the active site accommodated the middle and distal portions of the tail.^32,34^*C. albicans* CYP51 bound with posaconazole showed the triazole nitrogen coordinating to the heme group in the active site and the additional non-coordinating interactions facilitating the closed state of entry into the site.^32^ Similarly, triazole nitrogens were shown to coordinate to the heme group when a CYP51 homology model was derived of ketoconazole bound to *A. fumigatus* (fungus) through comparison with the known structure of fluconazole bound to the protein in *M. tuberculosis*.^35,36^ From studies such as these, clues may be gleaned about the role of dihalogenated phenyl rings and the hydroxyl core in the inhibition of CYP51. Fluconazole, being smaller than posaconazole, was shown to be fully buried in the active site when bound to *T. cruzi* and *M. tuberculosis* CYP51.^31,36^ In the *T. cruzi* CYP51–fluconazole complex, the fluorinated aromatic ring disrupted hydrogen-bonding of tyrosine side chain residues that support the heme rings, to which was attributed fluconazole's selective inhibition of *T. cruzi* CYP51 despite its smaller size.^11^ For the *M. tuberculosis* CYP51–fluconazole complex, the hydroxyl core of fluconazole was found to be hydrogen-bonded to the CYP51 heme group.^36^

Fenarimol analogues 4 and 61 were shown to bind to *T. cruzi* CYP51 in a similar fashion to posaconazole.^33^ The pyridine nitrogen was shown to coordinate to the heme iron, forming iron–nitrogen bonds longer (2.31 Å for 4 and 2.34 Å for 61) than those in CYP51–azole complexes (2.07–2.15 Å). The longer (weaker) coordination of pyridine nitrogen with the heme iron was suggested as a beneficial trait for fenarimol analogues as 4 and 61 showed weaker influence on human CYP51 and a higher selectivity for *T. cruzi* CYP51. Moreover, the trifluoromethyl group (CF_3_) in the 4 tail also disrupted tyrosine H-bonding to the heme group, further contributing to potency. Compounds 4 and 61 also formed more non-coordinating contacts with amino acid residues in the active site *vs.* posaconazole.

The SAR data in the present study supports CYP51 inhibition as a probable mechanism of action, when compared to the literature evidence described above. Firstly, the pyridyl group (ring 1) showed superior potency and the removal of the nitrogen atom on this ring caused loss of activity, similar to related literature studies.^32,34–36^ Secondly, the hydroxyl core (core Y) showed stronger potency compared to a matched analogue without this motif and compounds with halogenated phenyl groups (ring 2) showed higher potency than unsubstituted ones. Literature evidence showed the hydroxyl core and halogenated phenyl group of fluconazole disrupting the tyrosine H-bonding supporting the CYP51 heme group.^34,36^ Thirdly, SAR showed potency-tolerating variations in the ring 3 and tail positions in scaffolds S1 and S2 for a wide range of motifs, consistent with the diverse range of tail lengths and motifs reported in the literature, which were shown to offer additional non-coordinating interaction with residues around the entrance to the CYP51 active site, facilitating a closed-state of entry.^32,34^

**Fig. 15:**
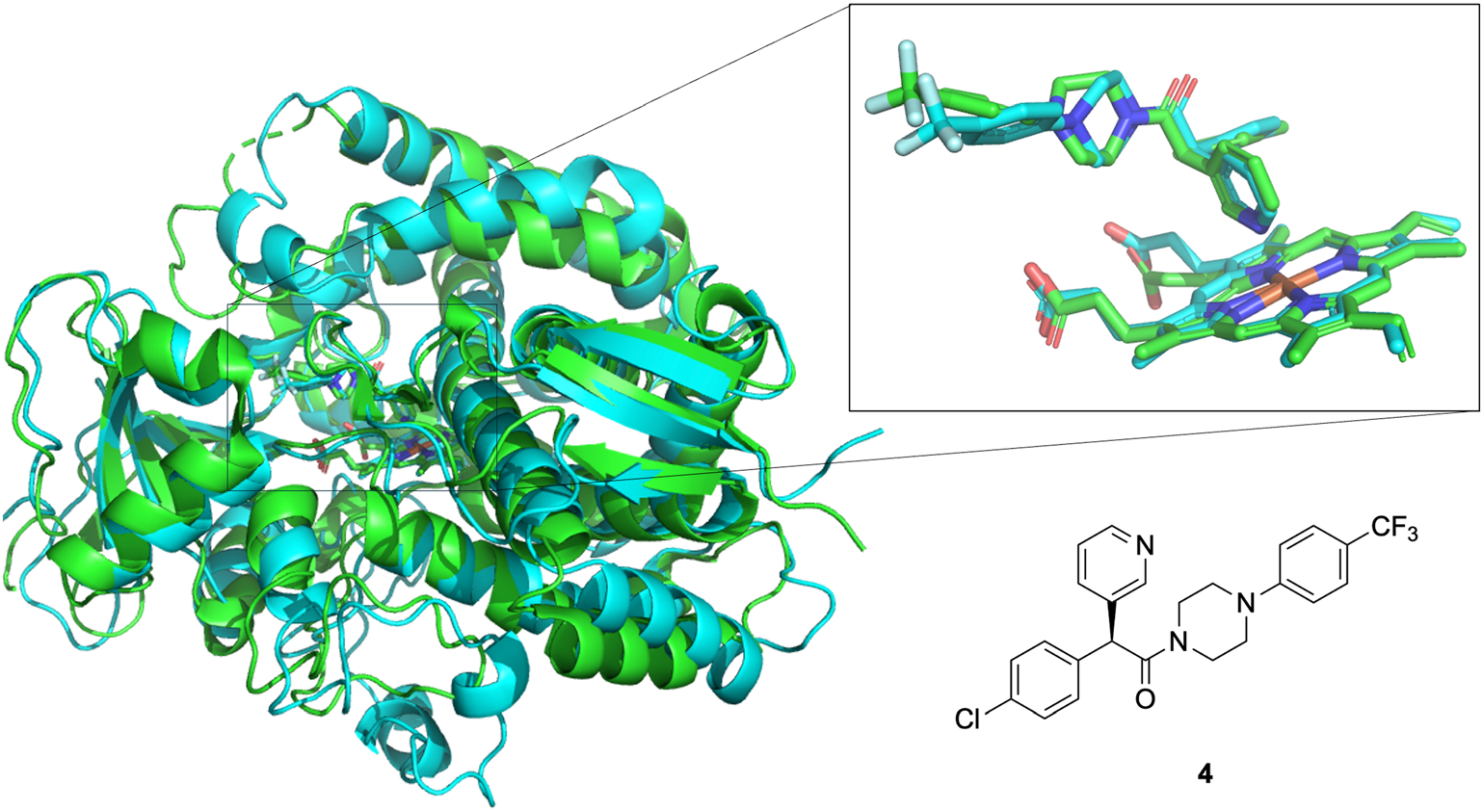
Overlap of *T. cruzi* sterol 14-alpha demethylase (Cyp51) in complex with compound 4 (green, from PDB 3ZG2) and predicted binding (Protenix) of compound 4 with the orthologous *M. mycetomatis* eburicol 14-alpha-demethylase (blue); inset: coordination with haem. Images rendered in PyMol, see SI for more details.

Overall, comparison of SAR findings with evidence from the literature suggests inhibition of CYP51 as a probable mechanism of action for fenarimol analogues against *M. mycetomatis*. Recently, the *M. mycetomatis* CYP51 gene was sequenced, and the homology 3D model of the *M. mycetomatis* CYP51 protein was produced, allowing the modelling of itraconazole and ravuconazole bound to CYP51.^37^ In support of this hypothesis, a comparison was made between an experimentally-determined structure of compound 4 bound to the *T. cruzi* Cyp51 and a calculated structure for the orthologous *M. mycetomatis* protein eburicol 14-alpha-demethylase bound to the same molecule (Fig. 15). There is good overlap, clearly indicating the expected coordination of the pyridine ring to the haem iron atom and extension of the hydrophobic portion of the molecule into the hydrophobic tunnel that accommodates a wide range of azole “tail” motifs.

While docking remains an ongoing part of Open Source Mycetoma^38^ direct elucidation of the interaction (including through recombinant expression of the protein and an experimental evaluation of protein binding or inhibition) could assist the future rational development of improved fenarimol analogues.

## Conflicts of interest

There is no conflict of interest to declare.

## Supplementary Material

MD-016-D5MD00427F-s001

MD-016-D5MD00427F-s002

MD-016-D5MD00427F-s003

MD-016-D5MD00427F-s004

## Data Availability

Supplementary information is available. See DOI: https://doi.org/10.1039/D5MD00427F. This article arises from a project carried out by an open source research organisation, Open Source Mycetoma (MycetOS). The guiding principles of MycetOS are that all data and ideas are freely shared, anyone may participate and no patents will be taken. Accordingly all data are publicly available through the project website, including: 1) Discussions and updates at https://github.com/OpenSourceMycetoma. 2) Electronic laboratory notebooks at: https://au-mynotebook.labarchives.com/share/Hung%2520Duong%2520-%2520PhD/Ni41fDY4MTUwLzUvVHJlZU5vZGUvNDI4NzgyODc5N3wxNi41, https://tinyurl.com/MyOS-HungELN, and https://tinyurl.com/MycetosDmitrij, with all links provided on the MycetOS Github repository at https://github.com/OpenSourceMycetoma/Series-1-Fenarimols/wiki/Sources-of-Data. 3) Online spreadsheet of all molecules evaluated along with primary data at http://tinyurl.com/MycetomaMols. In case the online providers becomes obsolete, offline snapshots of the ELNs have been uploaded to university repositories, for example for Hung Phat Duong at The University of Sydney eRepository at https://ses.library.usyd.edu.au/handle/2123/30280 (https://hdl.handle.net/2123/30280) and Dmitrij Melechov at University College London (https://doi.org/10.5522/04/28360370.v1). Data on all compounds evaluated have also been provided as SI. Monthly meetings are recorded and placed on YouTube, and may be found by searching “open source mycetoma” on that site. This paper was deposited on ChemRxiv (DOI: https://doi.org/10.26434/chemrxiv-2025-kj5x1) and underwent minor revisions between that deposition and the current manuscript.

## References

[cit1] Lim W., Melse Y., Konings M., Phat Duong H., Eadie K., Laleu B., Perry B., Todd M. H., Ioset J. R., van de Sande W. W. J. (2018). Addressing the most neglected diseases through an open research model: The discovery of fenarimols as novel drug candidates for eumycetoma. PLoS Neglected Trop. Dis..

[cit2] van de Sande W. W. J., Fahal A. H. (2024). An updated list of eumycetoma causative agents and their differences in grain formation and treatment response. Clin. Microbiol. Rev..

[cit3] Sheehan G., Konings M., Lim W., Fahal A., Kavanagh K., van de Sande W. W. J. (2020). Proteomic analysis of the processes leading to Madurella mycetomatis grain formation in Galleria mellonella larvae. PLoS Neglected Trop. Dis..

[cit4] van de Sande W. W. J. (2021). In vitro susceptibility testing for black grain eumycetoma causative agents. Trans. R. Soc. Trop. Med. Hyg..

[cit5] Ibrahim A. I., El Hassan A. M., Fahal A., van de Sande W. W. (2013). A histopathological exploration of the Madurella mycetomatis grain. PLoS One.

[cit6] Welsh O., Al-Abdely H. M., Salinas-Carmona M. C., Fahal A. H. (2014). Mycetoma medical therapy. PLoS Neglected Trop. Dis..

[cit7] Suleiman S. H., Wadaella el S., Fahal A. H. (2016). The Surgical Treatment of Mycetoma. PLoS Neglected Trop. Dis..

[cit8] Zein H. A., Fahal A. H., Mahgoub el S., El Hassan T. A., Abdel-Rahman M. E. (2012). Predictors of cure, amputation and follow-up dropout among patients with mycetoma seen at the Mycetoma Research Centre, University of Khartoum, Sudan. Trans. R. Soc. Trop. Med. Hyg..

[cit9] Wadal A., Elhassan T. A., Zein H. A., Abdel-Rahman M. E., Fahal A. H. (2016). Predictors of Post-operative Mycetoma Recurrence Using Machine-Learning Algorithms: The Mycetoma Research Center Experience. PLoS Neglected Trop. Dis..

[cit10] Crabol Y., Poiree S., Bougnoux M. E., Maunoury C., Barete S., Zeller V., Arvieux C., Pineau S., Amazzough K., Lecuit M. (2014). *et al.*, Last generation triazoles for imported eumycetoma in eleven consecutive adults. PLoS Neglected Trop. Dis..

[cit11] Lim W., Verbon A., van de Sande W. (2022). Identifying novel drugs with new modes of action for neglected tropical fungal skin diseases (fungal skinNTDs) using an Open Source Drug discovery approach. Expert Opin. Drug Discovery.

[cit12] Lim W., Nyuykonge B., Eadie K., Konings M., Smeets J., Fahal A., Bonifaz A., Todd M., Perry B., Samby K. (2022). *et al.*, Screening the pandemic response box identified benzimidazole carbamates, Olorofim and ravuconazole as promising drug candidates for the treatment of eumycetoma. PLoS Neglected Trop. Dis..

[cit13] Keenan M., Chaplin J. H., Alexander P. W., Abbott M. J., Best W. M., Khong A., Botero A., Perez C., Cornwall S., Thompson R. A. (2013). *et al.*, Two analogues of fenarimol show curative activity in an experimental model of Chagas disease. J. Med. Chem..

[cit14] Keenan M., Abbott M. J., Alexander P. W., Armstrong T., Best W. M., Berven B., Botero A., Chaplin J. H., Charman S. A., Chatelain E. (2012). *et al.*, Analogues of fenarimol are potent inhibitors of Trypanosoma cruzi and are efficacious in a murine model of Chagas disease. J. Med. Chem..

[cit15] Keenan M., Alexander P. W., Diao H., Best W. M., Khong A., Kerfoot M., Thompson R. C., White K. L., Shackleford D. M., Ryan E. (2013). *et al.*, Design, structure-activity relationship and in vivo efficacy of piperazine analogues of fenarimol as inhibitors of Trypanosoma cruzi. Bioorg. Med. Chem..

[cit16] Oh K., Matsumoto T., Yamagami A., Hoshi T., Nakano T., Yoshizawa Y. (2015). Fenarimol, a Pyrimidine-Type Fungicide, Inhibits Brassinosteroid Biosynthesis. Int. J. Mol. Sci..

[cit17] Burden R. S., Cooke D. T., Carter G. A. (1989). Inhibitors of Sterol Biosynthesis and Growth in Plants and Fungi. Phytochemistry.

[cit18] Todd M. H. (2019). Six Laws of Open Source Drug Discovery. ChemMedChem.

[cit19] Kloezen W., van Helvert-van Poppel M., Fahal A. H., van de Sande W. W. (2015). A Madurella mycetomatis Grain Model in Galleria mellonella Larvae. PLoS Neglected Trop. Dis..

[cit20] Kloezen W., Parel F., Bruggemann R., Asouit K., Helvert-van Poppel M., Fahal A., Mouton J., van de Sande W. (2018). Amphotericin B and terbinafine but not the azoles prolong survival in Galleria mellonella larvae infected with Madurella mycetomatis. Med. Mycol..

[cit21] van de Sande W. W., van Vianen W., ten Kate M., Fahal A., Bakker-Woudenberg I. (2015). Amphotericin B but not itraconazole is able to prevent grain formation in experimental Madurella mycetomatis mycetoma in mice. Br. J. Dermatol..

[cit22] Lewis D. F., Dickins M. (2003). Baseline lipophilicity relationships in human cytochromes P450 associated with drug metabolism. Drug Metab. Rev..

[cit23] Lewis D. F., Ito Y., Lake B. G. (2010). Quantitative structure-activity relationships (QSARs) for inhibitors and substrates of CYP2B enzymes: importance of compound lipophilicity in explanation of potency differences. J. Enzyme Inhib. Med. Chem..

[cit24] MycetOS , What's Open Source Mycetoma (MycetOS)? github, 2024, https://github.com/OpenSourceMycetoma/General-Start-Here, (accessed 2024 04-06-2024)

[cit25] Fischer R. T., Stam S. H., Johnson P. R., Ko S. S., Magolda R. L., Gaylor J. L., Trzaskos J. M. (1989). Mechanistic studies of lanosterol 14 alpha-methyl demethylase: substrate requirements for the component reactions catalyzed by a single cytochrome P-450 isozyme. J. Lipid Res..

[cit26] Trzaskos J. M., Bowen W. D., Shafiee A., Fischer R. T., Gaylor J. L. (1984). Cytochrome P-450-dependent oxidation of lanosterol in cholesterol biosynthesis. Microsomal electron transport and C-32 demethylation. J. Biol. Chem..

[cit27] Bennett J. E. (1974). Chemotherapy of systemic mycoses (first of two parts). N. Engl. J. Med..

[cit28] Latge J. P. (1999). Aspergillus fumigatus and aspergillosis. Clin. Microbiol. Rev..

[cit29] Maertens J. A. (2004). History of the development of azole derivatives. Clin. Microbiol. Infect..

[cit30] Pappas P. G., Kauffman C. A., Andes D. R., Clancy C. J., Marr K. A., Ostrosky-Zeichner L., Reboli A. C., Schuster M. G., Vazquez J. A., Walsh T. J. (2016). *et al.*, Clinical Practice Guideline for the Management of Candidiasis: 2016 Update by the Infectious Diseases Society of America. Clin. Infect. Dis..

[cit31] Lass-Florl C. (2011). Triazole antifungal agents in invasive fungal infections: a comparative review. Drugs.

[cit32] Hargrove T. Y., Friggeri L., Wawrzak Z., Qi A., Hoekstra W. J., Schotzinger R. J., York J. D., Guengerich F. P., Lepesheva G. I. (2017). Structural analyses of Candida albicans sterol 14alpha-demethylase complexed with azole drugs address the molecular basis of azole-mediated inhibition of fungal sterol biosynthesis. J. Biol. Chem..

[cit33] Hargrove T. Y., Wawrzak Z., Alexander P. W., Chaplin J. H., Keenan M., Charman S. A., Perez C. J., Waterman M. R., Chatelain E., Lepesheva G. I. (2013). Complexes of Trypanosoma cruzi sterol 14alpha-demethylase (CYP51) with two pyridine-based drug candidates for Chagas disease: structural basis for pathogen selectivity. J. Biol. Chem..

[cit34] Lepesheva G. I., Hargrove T. Y., Anderson S., Kleshchenko Y., Furtak V., Wawrzak Z., Villalta F., Waterman M. R. (2010). Structural insights into inhibition of sterol 14alpha-demethylase in the human pathogen Trypanosoma cruzi. J. Biol. Chem..

[cit35] Podust L. M., Poulos T. L., Waterman M. R. (2001). Crystal structure of cytochrome P450 14alpha-sterol demethylase (CYP51) from Mycobacterium tuberculosis in complex with azole inhibitors. Proc. Natl. Acad. Sci. U. S. A..

[cit36] Snelders E., Camps S. M., Karawajczyk A., Schaftenaar G., Kema G. H., van der Lee H. A., Klaassen C. H., Melchers W. J., Verweij P. E. (2012). Triazole fungicides can induce cross-resistance to medical triazoles in Aspergillus fumigatus. PLoS One.

[cit37] Nyuykonge B., Siddig E. E., Mhmoud N. A., Nyaoke B. A., Zijlstra E. E., Verbon A., Bakhiet S., Fahal A. H., van de Sande W. W. J. (2022). Epidemiological cut-off values for itraconazole and ravuconazole for Madurella mycetomatis, the most common causative agent of mycetoma. Mycoses.

[cit38] MycetOS , Crystal structures of azole-bound CYP51 enzymes from various organisms (mammals, bacteria, fungi and protozoan) #68. github, 2024, https://github.com/OpenSourceMycetoma/Series-1-Fenarimols/issues/68, (accessed 2024 04-06-2024)

