# Supplementary material for: Structure–activity relationships of fenarimol analogues with potent in vitro and in vivo activity against Madurella mycetomatis, the main causative agent of mycetoma
Source: RSC Med Chem. 2025 Sep 18;16(12):6094–108. doi: 10.1039/d5md00427f (PMC12529582; doi:10.1039/d5md00427f)
Supplement: MD-016-D5MD00427F-s004 [file MD-016-D5MD00427F-s004.pdf]

Supplementary Information (SI) for RSC Medicinal Chemistry.
This journal is © The Royal Society of Chemistry 2025

**Supporting Information for**

# Structure–activity relationships of fenarimol analogues with potent *in vitro* and *in vivo* activity against *Madurella mycetomatis,* the main causative agent of eumycetoma.

Hung Phat Duong[1,#], Dmitrij Melechov[2,3,#], Wilson Lim[4,#], Jingyi Ma[4,#], Kymberley R. Scroggie[1], Luxsika Rajendra,[2] Benjamin Perry[5], Luiza Cruz[5], Rahman Shah Zaib Saleem,[2,3,6] Peter J. Rutledge[1], Alice Motion[1*], Wendy W.J. van de Sande[4,*] and Matthew H. Todd[2,3*]

Affiliations:

[1] School of Chemistry, Faculty of Science, The University of Sydney, NSW 2006, Australia

[2] UCL School of Pharmacy, University College London, 29-39 Brunswick Square London WC1N 1AX, United Kingdom

[3] Structural Genomics Consortium, University College London, 29-39 Brunswick Square London WC1N 1AX, United Kingdom

[4] Department of Medical Microbiology & Infectious Diseases, Erasmus MC, University Medical Center Rotterdam, Dr. Molewaterplein 40, 3015 GD Rotterdam, The Netherlands

[5] The Drugs for Neglected Diseases Initiative, 15 Chemin Camille-Vidart, 1202 Geneva, Switzerland

[6] Department of Chemistry and Chemical Engineering, Syed Babar Ali School of Science and Engineering, Lahore University of Management Sciences, Lahore, 54792, Pakistan

[#] These authors contributed equally

* To whom correspondence should be addressed:

Alice Motion, alice.motion@sydney.edu.au

Wendy W. J. van de Sande, w.vandesande@erasmusmc.nl

Matthew H. Todd, matthew.todd@ucl.ac.uk

**Contents**


| | |
|---|---|
| Table S1: *In vitro* raw data for all compounds | S3 |
| Table S2: *In vivo* raw data for all compounds | S13 |
| Materials and Methods | S16 |
| NMR Data for Novel Compounds | S81 |
| Compounds Contributed from IMSA (Illinois Mathematics and Science Academy) | S168 |
| Large Scale Synthesis of **Int1** | S178 |
| References | S187 |

**Table S1: *In vitro* raw data for all compounds**

Individual compound contributions are provided. The full, live dataset can be found on the Open Source Mycetoma Master List at http://tinyurl.com/MycetomaMols (https://docs.google.com/spreadsheets/d/1YhK-3i2KwuVabo1GbZSgVjAUbavEICCMKi5v-EhNq80/edit?usp=sharing)

| MYOS Codes[#] | Other ID | Origin/ Synthesized by | 25 µM mean | 25 µM stdev | $MIC_{50}$ | Potency |
|---|---|---|---|---|---|---|
| MYOS_00001 | HPD14_1 | Duong USYD* | 20 | 11 | 0.5 | Excellent |
| MYOS_00002 | EPL-BS0038 | Epichem | -3 | 0 | 1 | Excellent |
| MYOS_00003 | EPL-BS0800 | Epichem* | -2 | 6 | 4 | Excellent |
| MYOS_00004 | EPL-BS1025 | Epichem* | -2 | 6 | 4 | Excellent |
| MYOS_00005 | HPD9-1 | Duong USYD* | 51 | 12 | 1 | Poor |
| MYOS_00006 | EPL-BS0237 | Epichem | 10 | 2 | >16 | Moderate |
| MYOS_00007 | EPL-BS0240 | Epichem | -5 | 1 | 4 | Excellent |
| MYOS_00008 | EPL-BS0178 | Epichem* | 20 | 39 | 8 | Excellent |
| MYOS_00009 | HPD20_1 | Duong USYD | 7 | 1 | 0.3 | Excellent |
| MYOS_00010 | EPL-BS1246 | Epichem* | 1 | 11 | 4 | Excellent |
| MYOS_00011 | HPD12_1 | Duong USYD* | 49 | 25 | 2 | Poor |
| MYOS_00012 | HPD17_2F3 | Duong USYD | 11 | 6 | 0.3 | Excellent |
| MYOS_00013 | EPL-BS0549 | Epichem | 17 | 14 | >16 | Moderate |
| MYOS_00014 | EPL-BS0115 | Epichem | -4 | 0 | 8 | Excellent |
| MYOS_00015 | HPD30_1 | Duong USYD | 10 | 7 | 2 | Excellent |

| | | | | | | |
|---|---|---|---|---|---|---|
| MYOS_00016 | DM7-1 | Melechov UCL | 18 | | 8 | Excellent |
| MYOS_00017 | HPD25_1 | Duong USYD | 6 | 2 | 2 | Excellent |
| MYOS_00018 | EPL-BS0132 | Epichem | -5 | 0 | >16 | Moderate |
| MYOS_00019 | HPD8-1 | Duong USYD | 59 | 5 | 16 | Poor |
| MYOS_00021 | HPD3-4 | Duong USYD | 42 | 2 | 16 | Poor |
| MYOS_00022 | EPL-BS0447 | Epichem | 22 | 29 | >16 | Poor |
| MYOS_00023 | EPL-BS0271 | Epichem | 4 | 12 | >16 | Moderate |
| MYOS_00024_00_01 | DM6-1 | Melechov UCL | 47 | | | Poor |
| MYOS_00024_00_02 | P4_C_001 | IMSA | 116 | | | Poor |
| MYOS_00025 | P4_B_013 LR8-1 | Rajendra UCL | 76 | | | Poor |
| MYOS_00026 | P4_C_009 LR10-1 | Rajendra UCL | 66 | | | Poor |
| MYOS_00027 | HPD26_1 | Duong USYD | 28 | 33 | 16 | Poor |
| MYOS_00028 | HPD27_2 | Duong USYD | 58 | 7 | 16 | Poor |
| MYOS_00029 | HPD28_1F2(SS,RR) | Duong USYD | 16 | 18 | 8 | Excellent |
| MYOS_00030 | HPD28_1F3(SR,RS) | Duong USYD | 45 | 6 | 16 | Poor |
| MYOS_00031 | HPD29_1 | Duong USYD | 20 | 3 | >16 | Poor |
| MYOS_00032 | EPL-BS0114 | Epichem | -5 | 1 | >16 | Moderate |

| | | | | | | |
|---|---|---|---|---|---|---|
| MYOS_00033 | P4_A_002<br>LR5-1 | Rajendra UCL | 103 | | | Poor |
| MYOS_00034 | P4_B_001<br>LR7-1 | Rajendra UCL | 95 | | | Poor |
| MYOS_00035 | P4_B_002<br>LR9-1 | Rajendra UCL | 71 | | | Poor |
| MYOS_00036 | EPL-BS0083 | Epichem* | 86 | 8 | | Poor |
| MYOS_00037 | EPL-BS0118 | Epichem* | 46 | 40 | >16 | Poor |
| MYOS_00038 | EPL-BS0167 | Epichem* | 97 | 12 | | Poor |
| MYOS_00039 | EPL-BS0282 | Epichem* | 57 | 58 | | Poor |
| MYOS_00040 | EPL-BS0290 | Epichem* | 97 | 23 | | Poor |
| MYOS_00041 | EPL-BS0309 | Epichem* | 91 | 6 | | Poor |
| MYOS_00042 | EPL-BS0374 | Epichem* | 98 | 22 | | Poor |
| MYOS_00043 | EPL-BS0400 | Epichem* | 93 | 16 | | Poor |
| MYOS_00044 | EPL-BS0407 | Epichem* | 69 | 36 | | Poor |
| MYOS_00045 | EPL-BS0425 | Epichem* | 66 | 21 | | Poor |
| MYOS_00046 | EPL-BS0435 | Epichem* | 86 | 21 | | Poor |
| MYOS_00047 | EPL-BS0480 | Epichem* | 85 | 19 | | Poor |
| MYOS_00048 | EPL-BS0506 | Epichem* | 99 | 19 | | Poor |
| MYOS_00049 | EPL-BS0512 | Epichem* | 72 | 20 | | Poor |
| MYOS_00050 | EPL-BS0516 | Epichem* | 82 | 16 | | Poor |

| | | | | | | |
|---|---|---|---|---|---|---|
| MYOS_00051 | EPL-BS0566 | Epichem* | 81 | 26 | | Poor |
| MYOS_00052 | EPL-BS0593 | Epichem* | 83 | 19 | | Poor |
| MYOS_00053 | EPL-BS0633 | Epichem* | 86 | 25 | | Poor |
| MYOS_00054 | EPL-BS0654 | Epichem* | 93 | 24 | | Poor |
| MYOS_00055 | EPL-BS0690 | Epichem* | 92 | 16 | | Poor |
| MYOS_00056 | EPL-BS0713 | Epichem* | 86 | 13 | | Poor |
| MYOS_00057 | EPL-BS0729 | Epichem* | 48 | 32 | | Poor |
| MYOS_00058 | EPL-BS0793 | Epichem* | 88 | 8 | | Poor |
| MYOS_00059 | EPL-BS0831 | Epichem* | 71 | 5 | | Poor |
| MYOS_00060 | EPL-BS0836 | Epichem* | 88 | 16 | | Poor |
| MYOS_00061 | EPL-BS0967 | Epichem* | 46 | 38 | | Poor |
| MYOS_00062 | EPL-BS1007 | Epichem* | 97 | 13 | | Poor |
| MYOS_00063 | EPL-BS1071 | Epichem* | 73 | 2 | | Poor |
| MYOS_00064 | EPL-BS1296 | Epichem* | 80 | 16 | | Poor |
| MYOS_00065 | EPL-BS1338 | Epichem* | 87 | 22 | | Poor |
| MYOS_00066 | EPL-BS1365 | Epichem* | 70 | 5 | | Poor |
| MYOS_00067 | EPL-BS0248 | Epichem | 53 | 50 | | Poor |
| MYOS_00068 | EPL-BS 250 | Epichem | 31 | 6 | | Poor |
| MYOS_00069 | EPL-BS 256 | Epichem | 52 | 24 | | Poor |
| MYOS_00070 | EPL-BS0322 | Epichem | 78 | 15 | | Poor |

| | | | | | | |
|---|---|---|---|---|---|---|
| MYOS_00071 | EPL-BS0494 | Epichem | 30 | 33 | | Poor |
| MYOS_00072 | EPL-BS0572 | Epichem | 87 | 53 | | Poor |
| MYOS_00073 | EPL-BS0595 | Epichem | 54 | 53 | | Poor |
| MYOS_00074 | EPL-BS0609 | Epichem | 27 | 26 | | Poor |
| MYOS_00076 | EPL-BS0150 | Epichem | 96 | 10 | | Poor |
| MYOS_00077 | EPL-BS0170 | Epichem | 106 | 19 | | Poor |
| MYOS_00078 | EPL-BS0193 | Epichem | 99 | 8 | | Poor |
| MYOS_00079 | EPL-BS0196 | Epichem | 87 | 1 | | Poor |
| MYOS_00080 | EPL-BS207 | Epichem | 105 | 7 | | Poor |
| MYOS_00081 | EPL-BS0269 | Epichem | 53 | 5 | | Poor |
| MYOS_00082 | EPL-BS0277 | Epichem | 85 | 3 | | Poor |
| MYOS_00083 | EPL-BS0 | Epichem | 81 | 2 | | Poor |
| MYOS_00084 | EPL-BS0310 | Epichem | 85 | 2 | | Poor |
| MYOS_00085 | EPL-BS0317 | Epichem | 81 | 3 | | Poor |
| MYOS_00086 | EPL-BS0348 | Epichem | 83 | 7 | | Poor |
| MYOS_00087 | EPL-BS0356 | Epichem | 101 | 10 | | Poor |
| MYOS_00088 | EPL-BS0358 | Epichem | 91 | 5 | | Poor |
| MYOS_00089 | EPL-BS0361 | Epichem | 69 | 2 | | Poor |
| MYOS_00090 | EPL-BS0362 | Epichem | 86 | 2 | | Poor |
| MYOS_00091 | EPL-BS0388 | Epichem | 87 | 0 | | Poor |

| | | | | | | |
|---|---|---|---|---|---|---|
| MYOS_00092 | EPL-BS0431 | Epichem | 82 | 2 | | Poor |
| MYOS_00093 | EPL-BS0449 | Epichem | 80 | 3 | | Poor |
| MYOS_00094 | EPL-BS0476 | Epichem | 88 | 0 | | Poor |
| MYOS_00096 | EPL-BS0499 | Epichem | 105 | 8 | | Poor |
| MYOS_00098 | EPL-BS0523 | Epichem | 84 | 3 | | Poor |
| MYOS_00099 | EPL-BS0528 | Epichem | 84 | 6 | | Poor |
| MYOS_00100 | EPL-BS0535 | Epichem | 77 | 3 | | Poor |
| MYOS_00101 | EPL-BS0558 | Epichem | 72 | 1 | | Poor |
| MYOS_00102 | EPL-BS0561 | Epichem | 86 | 3 | | Poor |
| MYOS_00103 | EPL-BS0575 | Epichem | 84 | 5 | | Poor |
| MYOS_00104 | EPL-BS0587 | Epichem | 88 | 2 | | Poor |
| MYOS_00105 | EPL-BS0603 | Epichem | 89 | 1 | | Poor |
| MYOS_00106 | EPL-BS0613 | Epichem | 85 | 5 | | Poor |
| MYOS_00107 | EPL-BS0642 | Epichem | 93 | 5 | | Poor |
| MYOS_00108 | EPL-BS0756 | Epichem | 83 | 4 | | Poor |
| MYOS_00109 | EPL-BS0761 | Epichem | 75 | 3 | | Poor |
| MYOS_00110 | EPL-BS0810 | Epichem | 89 | 1 | | Poor |
| MYOS_00111 | EPL-BS0839 | Epichem | 70 | 13 | | Poor |
| MYOS_00112 | EPL-BS0863 | Epichem | 87 | 3 | | Poor |
| MYOS_00113 | EPL-BS0881 | Epichem | 55 | 1 | | Poor |

| | | | | | | |
|---|---|---|---|---|---|---|
| MYOS_00114 | EPL-BS0922 | Epichem | 47 | 6 | | Poor |
| MYOS_00115 | EPL-BS0930 | Epichem | 81 | 1 | | Poor |
| MYOS_00116 | EPL-BS1008 | Epichem | 82 | 3 | | Poor |
| MYOS_00118 | EPL-BS1044 | Epichem | 86 | 1 | | Poor |
| MYOS_00119 | EPL-BS1072 | Epichem | 85 | 4 | | Poor |
| MYOS_00120 | EPL-BS1080 | Epichem | 39 | 8 | | Poor |
| MYOS_00122 | EPL-BS1387 | Epichem | 75 | 1 | | Poor |
| MYOS_00123 | EPL-BS1392 | Epichem | 54 | 37 | | Poor |
| MYOS_00124 | EPL-BS1439 | Epichem | 88 | 9 | | Poor |
| MYOS_00125 | EPL-BS1440 | Epichem | 71 | 1 | | Poor |
| MYOS_00126 | EPL-BS1482 | Epichem | 93 | 5 | | Poor |
| MYOS_00127 | EPL-BS1483 | Epichem | 97 | 1 | | Poor |
| MYOS_00128 | EPL-BS751 | Epichem | 52 | 7 | | Poor |
| MYOS_00129 | EPL-BS333 | Epichem | 81 | 7 | | Poor |
| MYOS_00130 | HPD033-2P4 | Duong USYD | 3 | | 2 | Excellent |
| MYOS_00131 | HPD033-3P3 | Duong USYD | 1 | | 4 | Excellent |
| MYOS_00132 | HPD035-1P6 | Duong USYD | 79 | | | Poor |
| MYOS_00133 | HPD037-1 | Duong USYD | -3 | | >16 | Moderate |
| MYOS_00134 | HPD040-1 | Duong USYD | 69 | | | Poor |
| MYOS_00135 | DM8-2 | Melechov UCL | 67 | | | Poor |

| | | | | | | |
|---|---|---|---|---|---|---|
| MYOS_00136 | DM10-2 | Melechov UCL | 11 | | 2 | Excellent |
| MYOS_00137 | DM11-2 | Melechov UCL | 123 | | | Poor |
| MYOS_00139 | DM17-3 | Melechov UCL | 79 | | | Poor |
| MYOS_00140 | DM18-2 | Melechov UCL | 92 | | | Poor |
| MYOS_00141 | DM19-3 | Melechov UCL | 64 | | | Poor |
| MYOS_00142 | DM22-1 | Melechov UCL | 112 | | | Poor |
| MYOS_00143 | DM23-6 | Melechov UCL | 74 | | | Poor |
| MYOS_00144 | DM24-1 | Melechov UCL | 95 | | | Poor |
| MYOS_00145 | DM25-1 | Melechov UCL | 8 | | 8 | Excellent |
| MYOS_00147 | DM27-1 | Melechov UCL | 8 | 4 | 8 | Excellent |
| MYOS_00148 | DM28-1 | Melechov UCL | 6 | 3 | 8 | Excellent |
| MYOS_00149 | DM29-1 | Melechov UCL | 52 | 22 | | Poor |
| MYOS_00150 | DM30-1 | Melechov UCL | 10 | 9 | | Excellent |
| MYOS_00151 | DM31-1 | Melechov UCL | 78 | 4 | | Poor |
| MYOS_00163 | HPD041-3P2 | Duong USYD | 74 | 38 | | Poor |
| MYOS_00164 | HPD049-1P2 | Duong USYD | 68 | 10 | | Poor |
| MYOS_00165 | HPD050-1P | Duong USYD | 83 | 17 | | Poor |
| MYOS_00166 | HPD045-1P3 | Duong USYD | 5 | 3 | 2 | Excellent |
| MYOS_00167 | HPD046-1P3 | Duong USYD | 5 | 4 | 0.5 | Excellent |
| MYOS_00168 | HPD047-1P3 | Duong USYD | 5 | 2 | 8 | Excellent |

| | | | | | | |
|---|---|---|---|---|---|---|
| MYOS_00169 | HPD052-1P4 | Duong USYD | 43 | 5 | | Poor |
| MYOS_00170 | HPD054-1P5.3 | Duong USYD | 38 | 26 | | Poor |
| MYOS_00171 | HPD055-1P3 | Duong USYD | 19 | 23 | >16 | Moderate |
| MYOS_00172 | HPD056-1P4 | Duong USYD | 83 | 6 | | Poor |
| MYOS_00173 | HPD053-1 | Duong USYD | 67 | 3 | | Poor |
| MYOS_00174 | HPD044-1 | Duong USYD | 8 | 6 | 2 | Excellent |
| MYOS_00175 | HPD043-1 | Duong USYD | 16 | 1 | >16 | Moderate |
| MYOS_00189 | KRS_011_002 | Scroggie USYD | 54 | 13 | | Poor |
| MYOS_00190 | DM36-1 | Melechov UCL | 61 | 22 | | Poor |
| MYOS_00191 | DM37-1 | Melechov UCL | 6 | 3 | 16 | Moderate |
| MYOS_00192 | DM38-1 | Melechov UCL | 11 | 2 | 16 | Moderate |
| MYOS_00195 | DM40-1 | Melechov UCL | 63 | 17 | | Poor |
| MYOS_00196 | DM41-2 | Melechov UCL | 7 | 1 | 8 | Excellent |
| MYOS_00197 | DM42-1 | Melechov UCL | 7 | 3 | >16 | Moderate |
| MYOS_00203 | DM53-1 | Melechov UCL | 82 | 11 | >16 | Poor |
| MYOS_00204 | DM54-1 | Melechov UCL | 11 | 6 | >16 | Moderate |
| MYOS_00205 | DM55-1 | Melechov UCL | 33 | 34 | >16 | Poor |
| MYOS_00206 | DM56-1 | Melechov UCL | 24 | 26 | >16 | Poor |
| MYOS_00310 | DM44-1 | Melechov UCL | 5 | 3 | | Excellent |
| MYOS_00311 | DM45-1 | Melechov UCL | 41 | 11 | | Poor |

| | | | | | | |
|---|---|---|---|---|---|---|
| MYOS_00312 | P4_A_001 | IMSA | 77 | 31 | | Poor |
| MYOS_00313 | P4_B_004 | IMSA | 38 | 23 | | Poor |
| MYOS_00314 | P4_B_005 | IMSA | 70 | 19 | | Poor |
| MYOS_00321 | DM61-1 | Melechov UCL | 5 | 5 | 8 | Excellent |
| MYOS_00446 | HPD057-2P4 | Duong USYD | 11 | 1 | 0.5 | Excellent |
| MYOS_00447 | HPD058-2P6 | Duong USYD | 10 | 2 | 0.3 | Excellent |
| MYOS_00448 | HPD059A-2P3 | Duong USYD | 11 | 1 | 1 | Excellent |
| MYOS_00449 | HPD059B-2P2.3 | Duong USYD | 11 | 2 | 0.5 | Excellent |
| MYOS_00450 | HPD060-2P1.3 | Duong USYD | 10 | 3 | 0.5 | Excellent |
| MYOS_00451 | HPD061-1R2P3.4 | Duong USYD | 11 | 2 | 2 | Excellent |
| MYOS_00452 | HPD062A-1P4 | Duong USYD | 13 | 4 | 0.1 | Excellent |
| MYOS_00453 | HPD062B-3P1.2 | Duong USYD | 12 | 4 | 1 | Excellent |
| MYOS_00454 | HPD063-1R2P2.3 | Duong USYD | 13 | 2 | 8 | Excellent |
| MYOS_00455 | HPD074-1P3.2 | Duong USYD | 12 | 5 | 2 | Excellent |
| MYOS_00510 | HPD080-3P4 | Duong USYD | 3 | 20 | | Excellent |
| MYOS_00511 | HPD081-1FP3.2 | Duong USYD | 194 | 21 | | Poor |
| MYOS_00512 | HPD082-1P2R2(P4) | Duong USYD | 141 | 42 | | Poor |
| MYOS_00513 | HPD083-1P4 | Duong USYD | 197 | 8 | | Poor |
| MYOS_00514 | HPD084-1P2.2 | Duong USYD | 123 | 63 | | Poor |
| MYOS_00515 | HPD085-1P3 | Duong USYD | 211 | 18 | | Poor |

| | | | | | | |
|---|---|---|---|---|---|---|
| MYOS_00516 | HPD086-1P4 | Duong USYD | 111 | 22 | | Poor |
| MYOS_00517 | HPD087-1P2 | Duong USYD | 91 | 33 | | Poor |
| MYOS_00518 | HPD088-1P5 | Duong USYD | 37 | 82 | | Poor |
| MYOS_00519 | HPD092-1P2 | Duong USYD | 119 | 20 | | Poor |
| MYOS_00520 | HPD093-1P2 | Duong USYD | 81 | 21 | | Poor |

* Screened in previous study[1]

# Where salt codes are abbreviated, all compounds were obtained as a free base (salt code: 00). Where batch codes are abbreviated, all compounds were obtained as a first batch (batch code: 01).

**Table S2: *In vivo* raw data for all compounds**

| MYOS Codes# | Other ID | Origin/ Synthesized by | d10 survival % | d10 *p*-value (Log-Rank) | Significance |
|---|---|---|---|---|---|
| MYOS_00001 | HPD14_1 | Duong USYD | 14.7 | 0.015 | yes * |
| MYOS_00002 | EPL-BS0038 | Epichem | 6.5 | 0.189 | no |
| MYOS_00003 | EPL-BS0800 | Epichem | 10 | 0.74 | no |
| MYOS_00004 | EPL-BS1025 | Epichem | 19.2 | 0.044 | yes * |
| MYOS_00005 | HPD9-1 | Duong USYD | 7.7 | 0.412 | no |
| MYOS_00007 | EPL-BS0240 | Epichem | 7.7 | 0.932 | no |
| MYOS_00008 | EPL-BS0178 | Epichem | 36.7 | <0.0001 | yes *** |
| MYOS_00009 | HPD20_1 | Duong USYD | 5.1 | 0.585 | no |

| | | | | | |
|---|---|---|---|---|---|
| MYOS_00010 | EPL-BS1246 | Epichem | 7.14 | 0.61 | no |
| MYOS_00011 | HPD12_1 | Duong USYD | 3.3 | 0.0004 | yes *** (increased death) |
| MYOS_00012 | HPD17_2F3 | Duong USYD | 13.2 | 0.064 | no |
| MYOS_00013 | EPL-BS0549 | Epichem | 7.7 | 0.983 | no |
| MYOS_00014 | EPL-BS0115 | Epichem | 8.88 | 0.82 | no |
| MYOS_00015 | HPD30_1 | Duong USYD | 10.5 | 0.762 | no |
| MYOS_00017 | HPD25_1 | Duong USYD | 10 | 0.225 | no |
| MYOS_00130 | HPD033-2P4 | Duong USYD | 5.6 | 0.064 | no |
| MYOS_00131 | HPD033-3P3 | Duong USYD | 9.1 | 0.179 | no |
| MYOS_00136 | DM10-2 | Melechov UCL | 7.9 | 0.626 | no |
| MYOS_00147 | DM27-1 | Melechov UCL | 4 | 0.522 | no |
| MYOS_00166 | HPD045-1P3 | Duong USYD | 4.44 | 0.2027 | no |
| MYOS_00167 | HPD046-1P3 | Duong USYD | 39.5 | <0.0001 | yes **** |
| MYOS_00168 | HPD047-1P3 | Duong USYD | 0 | 0.668 | no |
| MYOS_00174 | HPD044-1 | Duong USYD | 4.44 | 0.1805 | no |
| MYOS_00196 | DM41-2 | Melechov UCL | 6.67 | 0.3448 | no |
| MYOS_00203 | DM53-1 | Melechov UCL | 4.76 | 0.2038 | no |
| MYOS_00204 | DM54-1 | Melechov UCL | 2.27 | 0.6934 | no |
| MYOS_00206 | DM56-1 | Melechov UCL | 7.5 | 0.8885 | no |

| | | | | | |
|---|---|---|---|---|---|
| MYOS_00310 | DM44-1 | Melechov UCL | 14 | 0.0024 | yes ** |
| MYOS_00321 | DM61-1 | Melechov UCL | 3.33 | 0.5256 | no |

# Where salt codes are abbreviated, all compounds were obtained as a free base (salt code: 00). Where batch codes are abbreviated, all compounds were obtained as a first batch (batch code: 01).

## Materials and Methods

*General Experimental Details*

A fenarimol library containing 73 analogues was kindly provided by the Drugs for Neglected Diseases *initiative* (DND*i*) *via* Epichem (Perth, Australia). The compounds were dissolved in DMSO to 10 mM. For the synthesis of new fenarimols, all chemical reagents were purchased from Sigma Aldrich, Fisher Scientific, Merck and Fluorochem. Unless specified in experimental protocols, reagents were used as received without further purification. Analytical Thin Layer Chromatography (TLC) was performed on TLC Silica gel 60 $F_{254,}$ 20 x 20 cm aluminium sheets (Merck) and visualized under UV light (254 nm). Compound purification was performed on Biotage Selekt flash chromatography system. Normal phase purification was performed on Biotage Sfar Silica 60 µm 5 g/10 g/25 g/50 g columns. Reverse phase chromatography was performed on Biotage Sfar C18 Duo 100 Å 30 µm, 30 g columns. Acidity measurements were evaluated with pH-Fix 0–14 colour fixed indicator sticks (Fisherbrand). Reactions using air sensitive reagents were conducted under argon or nitrogen atmosphere with anhydrous solvents and oven dried glass vessels. All reported yields refer to chromatographically and spectroscopically pure products. Calculated logD, flexibility and number of rotational bonds were calculated using StarDrop™ ADME QSAR and Simple Properties models.

*General Spectroscopic Analysis*

$^{1}H$ and $^{13}C$ NMR spectra were obtained on either Bruker Advance DPX200 (200 MHz and 50 MHz respectively), DPX400 (400 MHz and 100 MHz respectively), DPX500 (500 MHz and 126 MHz respectively) or DPX600 (600 MHz and 150 MHz respectively) instruments. Deuterated chloroform ($CDCl_3$), methanol-*d*$_4$ ($CD_3OD$), acetone-*d*$_6$ $(CD_3)_2CO$ and dimethylsulfoxide-*d*$_6$ ($(CD_3)_2SO$) were used as solvents and for internal locking. Chemical shifts were measured in parts per million (ppm) and coupling constants (*J*) calculated in Hertz (Hz). $^{1}H$ NMR chemical shifts were referenced to residual solvent signals δ 7.26 ppm ($CDCl_3$), δ 4.79 ppm ($CD_3OD$) and δ 2.05 ppm ($(CD_3)_2CO$). $^{1}H$ NMR splitting patterns have the following assigned definitions: s (singlet), d (doublet), t (triplet), q (quartet), m (multiplet), ds (doublet of singlets), dd (doublet of doublets), dt (doublet of triplets), ddd (doublet of doublet of doublets), tt (triplet of triplets) and app (apparent). $^{13}C$ NMR chemical shifts were referenced to residual solvent signals δ 77.16 ppm ($CDCl_3$) and δ 49.00 ppm ($CD_3OD$), δ 29.84 and 206.26 ppm ($(CH_3)_2CO$), and δ 39.52 ppm ($(CD_3)_2SO$). Integrals are relative. Low resolution mass

spectrometry (m/z) was carried out on a Bruker amaZon SL quadrupole ion trap mass spectrometer using electrospray ionization (ESI) or atmospheric-pressure chemical ionization (APCI). Liquid Chromatography Mass Spectrometry (LCMS) data were acquired on Agilent 1260 Infinity II system. High Resolution Mass Spectrometry (HRMS) analysis was performed on Bruker 7T FT-ICR (ESI & ACPI), or Waters Q-ToF Premier Tandem Mass Spectrometer with papaverine $[M+H]^+$ = 340.1549 or reserpine $[M+H]^+$ = 609.2812 used for calibration. Infrared Spectra (IR) were recorded on Agilent Cary 630 FTIR or Bruker Platinum Alpha-E FTIR spectrometers. Samples were analysed neat. IR spectra are reported with frequency of maximum absorbance $\nu_{max}$ ($cm^{-1}$). Elemental analyses were carried out by Dr Christopher McRae at the Chemical Analysis Facility, Macquarie University on a Carlo Erba EA 1108 Elemental Analyzer. Melting points (m.p.) were recorded on a Stanford Research Systems OptiMelt at 2°C per min (capillaries ø = 1.5–1.6 mm, 90 mm).

As an open science project, the laboratory notebooks are openly available, for example at https://au-mynotebook.labarchives.com/share/Hung%2520Duong%2520-%2520PhD/Ni41fDY4MTUwLzUvVHJlZU5vZGUvNDI4NzgyODc5N3wxNi41, https://tinyurl.com/MyOS-HungELN and https://tinyurl.com/MycetosDmitrij, with all links provided on the MycetOS Github repository (https://github.com/OpenSourceMycetoma/Series-1-Fenarimols/wiki/Sources-of-Data). In case the ELN software provider becomes obsolete we have also uploaded offline snapshots of the ELNs, for example for Hung Phat Duong at The University of Sydney eRepository at https://ses.library.usyd.edu.au/handle/2123/30280 (https://hdl.handle.net/2123/30280) and Dmitrij Melechov at University College London (https://doi.org/10.5522/04/28360370.v1).

## Experimental Procedures

*General Synthetic Procedure A: Lithiation*

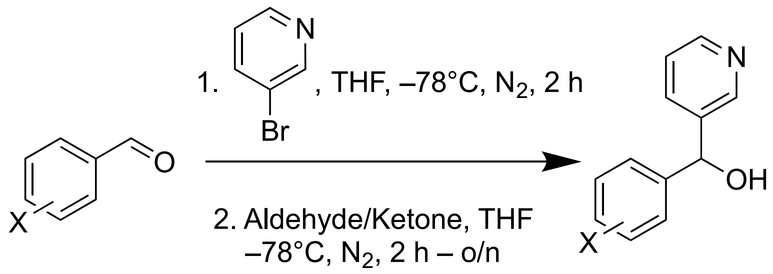

*n*-Butyllithium (2.0 equiv., 1.6 M solution in hexanes) was added dropwise to a solution of the bromo-substituted compound (2.0 equiv.) in dried diethyl ether at –78°C. The reaction mixture was stirred for 2 h. A solution of the ketone or aldehyde (1.0 equiv.) in dried diethyl ether was added dropwise at –78°C. The reaction was allowed to warm to room temperature (rt) overnight, quenched with water and extracted with ethyl acetate (× 3). The organic phases were combined, dried ($MgSO_4$) and concentrated under reduced pressure, and used without further purification.

*General Synthetic Procedure B: Nucleophilic Substitution*

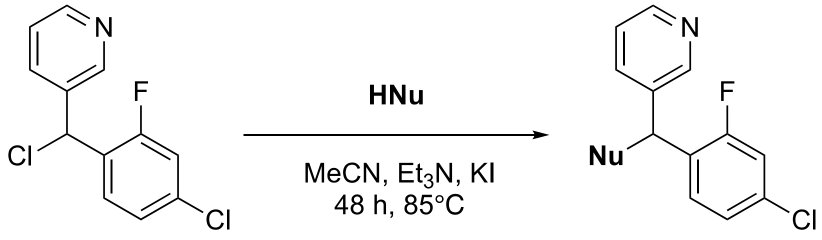


A 5 mL microwave vial was charged with aryl halide (1.0 equiv.) dissolved in anhydrous MeCN (2.0 equiv.), followed by the addition of the desired nucleophile (HNu, 1.5 equiv.), anhydrous $Et_3N$ (2.0 equiv.) and dry KI (0.05–0.10 equiv.). The reaction mixture was purged with argon for 10 min and then heated under reflux at 85°C for 48 h. Reaction completion was monitored by TLC. The stirring mixture was allowed to cool to rt and the residue was partitioned between $CH_2Cl_2$ and sat. $Na_2CO_3$ solution. The aqueous phase was extracted twice with $CH_2Cl_2$, and the combined organic phases were dried over $Na_2SO_4$, filtered and concentrated under reduced pressure to give the crude fenarimol analogue which was purified by flash chromatography to afford the desired product.

*General Synthetic Procedure C: Grignard Reaction*

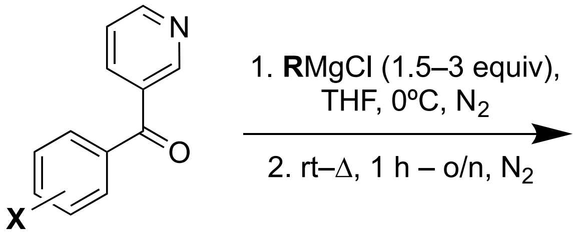

The Grignard reagent (**R**MgCl, 1.5–4.0 equiv.) of known concentration in dry THF (determined by titration)[2] was added to a solution of the ketone (1.0 equiv.) in dry THF at 0°C. The progress of the reaction was monitored by TLC as the reaction was allowed to warm to rt and then, if necessary, the reaction was heated at reflux (0.25–6 h). The reaction was quenched with distilled water and concentrated to remove THF. The crude reaction was extracted with ethyl acetate (× 3). The organic phases were combined, dried ($MgSO_4$) and concentrated under reduced pressure.

*General Synthetic Procedure D: Diarylalcohol Oxidation*

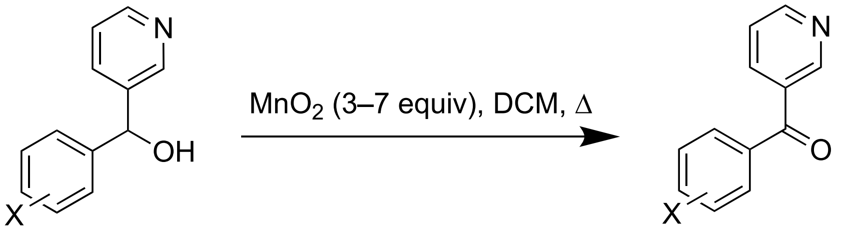


Activated manganese(IV) oxide (3.0–7.0 equiv.) was added to a solution of diarylalcohol (1.0 equiv.) in $CH_2Cl_2$. After being heated at reflux for 4 h, the reaction mixture was allowed to cool and filtered through celite. The filter cake was washed with $CH_2Cl_2$. The filtrate was concentrated under reduced pressure.

*General Synthetic Procedure E: Buchwald-Hartwig Amination*

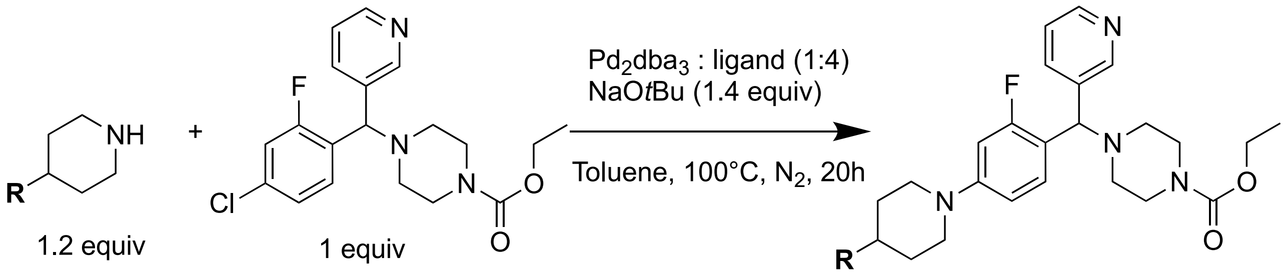


A mixture of the heteroaryl chloride (1 equiv., 0.1–0.5 mmol), amine (1.2 equiv.), $Pd_2dba_3$ (4% mol equiv.), ligand (16% mol equiv.) and NaO*t*Bu (1.4 equiv.) were suspended in dried toluene under nitrogen. The reaction was heated to and maintained at 100°C until the reaction was completed as judged by TLC visualization. The cooled reaction mixture was filtered through celite and washed with ethyl acetate (× 3). The solvent was evaporated to obtain the crude product.

*General Synthetic Procedure F: Friedel-Crafts Acylation*

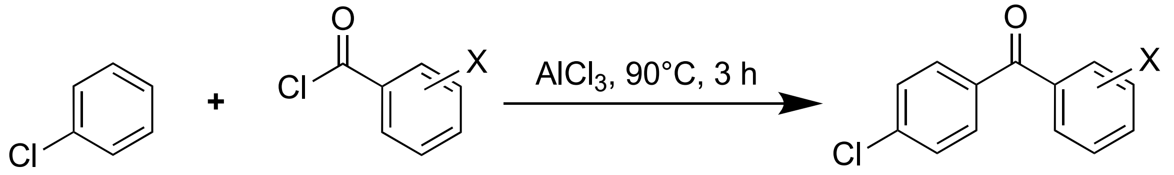


Benzoyl chloride (1 equiv.) was cautiously added to aluminium trichloride (1.5 equiv.) suspended in chlorobenzene, and the reaction mixture was slowly warmed to 90 °C. The reaction temperature was maintained until no more hydrogen chloride gas was evolved (about 3 h minimum). The reaction was cooled and poured over a mixture of crushed ice (150 mL) and 1 M hydrochloric acid (100 mL). The mixture was extracted with $CH_2Cl_2$ (2 × 150 mL), then washed with 2 M NaOH (2 × 100 mL) and water (75 mL). The organic phase was dried, and the solvent was evaporated.

*General Synthetic Procedure G: Acylation*

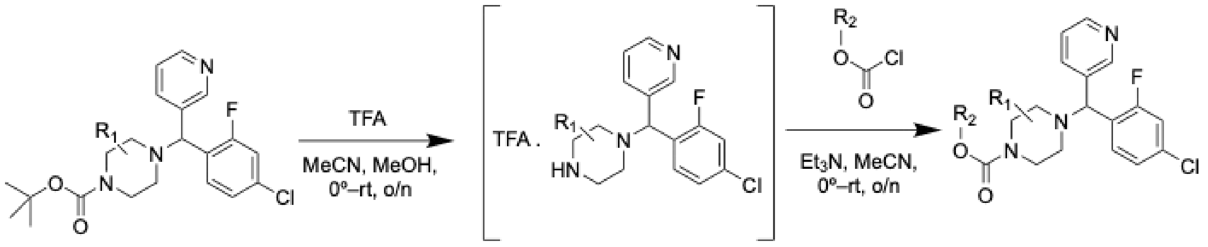


TFA (10 equiv) was added to a solution of the Boc-piperazine compound (**MYOS_00011**, 1 equiv) in MeCN at 0°C. The reaction mixture was allowed to warm to rt overnight, concentrated under reduced pressure to give the crude TFA salt intermediate (**Int15**), used without further purification. Anhydrous triethylamine (25 equiv) and alkyl chloroformate (0.9 – 1 equiv) were added to a solution of the TFA salt intermediate in MeCN at 0°C. The reaction mixture was allowed to warm to rt overnight, quenched with a saturated solution of ammonium chloride and extracted with MeCN. The organic layers were combined, dried ($MgSO_4$) and concentrated under reduced pressure.

For simplicity, compounds are listed in order of the MyOS codes used in this paper and the online project, with synthetic intermediates **Int1**–**Int17** listed first.

(4-Chloro-2-fluorophenyl)(pyridin-3-yl)methanol (Intermediate **Int1**)

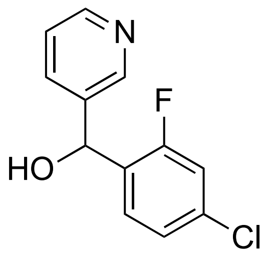

Prepared according to *General Synthetic Procedure A*, using 4-chloro-2-fluorobenzaldehyde (4.4 g, 27 mmol) to give *the title compound* as a reddish orange waxy solid (5.6 g, 88%), used without further purification. $^{1}$H NMR (500 MHz, Acetone-$d_6$) δ 8.62 (d, $J$ = 2.4 Hz, 1H), 8.46 (dd, $J$ = 4.7, 1.7 Hz, 1H), 7.92 – 7.57 (m, 2H), 7.51 – 7.26 (m, 2H), 7.20 (m, 1H), 6.14 (s, 1H); $^{13}$C NMR (126 MHz, Acetone-$d_6$) δ 160.3 (d, $^{1}J_{CF}$ = 249 Hz), 149.6, 149.2 (d, $^{4}J_{CF}$ = 1.6 Hz), 139.9, 134.6, 134.3 (d, $^{3}J_{CF}$ = 10.5 Hz), 131.7 (d, $^{2}J_{CF}$ = 13.7 Hz), 129.9 (d, $^{3}J_{CF}$ = 5.2 Hz), 125.7 (d, $^{4}J_{CF}$ = 3.6 Hz), 124.2, 116.6 (d, $^{2}J_{CF}$ = 25.4 Hz), 67.6; $^{19}$F NMR (471 MHz, Acetone-$d_6$) δ –116.82; LRMS *m/z* (ESI) 296 (27%), 294 (100%), 240 ([M+H]$^+$, 2%), 238 ([M+H]$^+$, 8%); HRMS (ESI) calcd. for [$C_{12}H_{10}{}^{37}ClFNO$]$^+$ 240.04054 and [$C_{12}H_{10}{}^{35}ClFNO$]$^+$ 238.04350 ([M+H]$^+$), found 240.04000, 238.04295; IR (film): $\nu_{max}$ 3113 (br), 2929, 1609, 1578, 1482, 1426, 1402 cm$^{-1}$. Spectroscopic data matched those in the literature.[1]

A protocol and data for the large-scale synthesis of **Int1**, performed by a contract research organization, supported by the Drugs for Neglected Diseases *initiative* and contributed to MycetOS, may be found later in this Supporting Information file.

3-(Chloro(4-chloro-2-fluorophenyl)methyl)pyridine (Intermediate **Int2**)

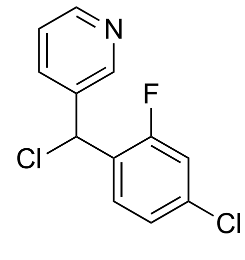

(4-Chloro-2-fluorophenyl)(pyridin-3-yl)methanol **Int1** (1.1 g, 4.8 mmol, 1.0 equiv.) was dissolved in $CH_2Cl_2$ (15 mL) and the mixture was cooled to 0°C in an ice bath. Thionyl chloride (0.70 mL, 9.5 mmol, 2.0 equiv.) was added and the reaction was allowed to warm to rt over ~2 h. The reaction mixture was partitioned between $Na_2CO_3$ (sat.) and $CH_2Cl_2$ and the aqueous phase was washed with $CH_2Cl_2$ (3 × 10 mL). The combined organic phases were washed with brine (2 × 7.5 mL), dried with $MgSO_4$, filtered and concentrated under reduced pressure to afford *the title compound* as a light brown oil (1.1 g, 90%). The halogenated

intermediate was carried forward without further purification. $^{1}$H NMR (400 MHz, Chloroform-*d*) δ 8.68 (d, *J* = 2.4 Hz, 1H), 8.60 (d, *J* = 4.9 Hz, 1H), 7.80 (dt, *J* = 8.0, 2.0 Hz, 1H), 7.51 (app t, *J* = 8.2 Hz, 1H), 7.36 (dd, *J* = 8.0, 4.8 Hz, 1H), 7.23 (dd, *J* = 8.5, 1.4 Hz, 1H), 7.14 (dd, *J* = 9.9, 2.1 Hz, 1H), 6.39 (s, 1H). LRMS *m/z* (ESI) 255.9 ([M+H]$^{+}$, 100%). IR (film): $\nu_{max}$ 3034, 1684, 1610, 1576, 1483, 1408, 1200, 1073, 902, 600 cm$^{-1}$. Spectroscopic data matched those in the literature.[3]

2-(4-Chlorophenyl)-2-(pyridin-3-yl)acetonitrile (Intermediate **Int3**)

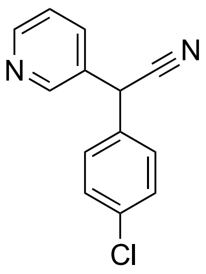


4-Chlorophenylacetonitrile (7.3 g, 48 mmol, 2.0 equiv.) and 3-fluoropyridine (2.4 g, 24 mmol, 1.0 equiv.) were dissolved in *N*-methyl-2-pyrrolidone (21 mL). The solution was placed in a water bath at 0°C and stirred for 5 min. Potassium *tert*-butoxide (8.1 g, 73 mmol, 3.0 equiv.) was added in aliquots until the mixture changed in colour from yellow/orange to dark green-brown. The reaction flask was fitted with a reflux condenser and the mixture heated at 85°C overnight. The vessel was cooled to 0°C and the reaction was quenched with 1 M HCl (aq) until pH 6–7, followed by dilution with EtOAc. White solid present in the mixture was removed by filtration through a sinter funnel with subsequent separation of the phases. The organic layer was washed with $H_2O$ (2 × 15 mL), dried with $MgSO_4$, filtered and concentrated under reduced pressure to give an oily residue. The product was purified by Biotage Selekt column chromatography (10–30% EtOAc in hexanes) to give a material that was judged to be semi-pure by TLC analysis (70% EtOAc in hexanes). This product was partitioned into 2 M HCl (aq) and $Et_2O$, and the phases were separated. The aqueous layer was repeatedly washed with $Et_2O$ (2 × 15 mL), treated with 2 M NaOH (aq) until approximately pH 8 and extracted with EtOAc. The combined EtOAc extracts were washed with brine (15 mL), dried with $MgSO_4$, filtered and concentrated to give a crude product. The sample was repurified by the same chromatography method (10–30% EtOAc in hexanes) to give *the title compound* as an amber oil (2.6 g, 50%). $^{1}$H NMR (500 MHz, Chloroform–*d*) δ 8.67 (d, *J* = 2.4 Hz, 1H), 8.64 (dd, *J* = 4.9, 1.5 Hz, 1H), 7.81 (app dt, *J* = 8.0, 2.0 Hz, 1H), 7.40 (d, *J* = 8.5 Hz, 2H), 7.37–7.33 (m, 1H), 7.30 (d, *J* = 8.6 Hz, 2H), 5.24 (s, 1H). LRMS *m/z* (ESI+) 229.0 ([M+H]$^{+}$, 100%). IR (film): $\nu_{max}$ 2200, 1636, 1580, 1500, 1423, 1092, 782, 708 cm$^{-1}$. Spectroscopic data matched those in the literature.[4]

2-(4-Chlorophenyl)-2-(pyridin-3-yl)acetic acid (Intermediate **Int4**)

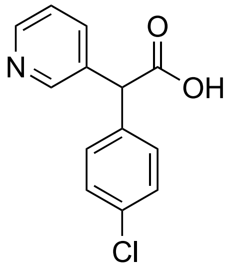

Nitrile intermediate **Int3** (248 mg, 1.1 mmol, 1.0 equiv.) was dissolved in 2 M NaOH (aq) (6.0 mL, 1.1 mmol, 1.0 equiv.) and the suspension was purged with Ar for 5 min. The stoppered reaction flask was fitted with an Ar balloon and the solution was heated overnight at 110°C. The mixture was allowed to cool to rt and was treated with $Et_2O$ (10 mL), followed by the separation of the phases. The aqueous phase was adjusted to pH 5 by the addition of 4 M HCl (aq). The resulting solution was extracted with EtOAc (2 × 10 mL). The organic phases were combined, dried with $MgSO_4$, filtered and concentrated under reduced pressure to give *the title compound* as a pale-yellow solid (131 mg, 49%) that was used in the next synthetic step without further purification. $^1$H NMR (500 MHz, Chloroform-d) δ 12.43 (s, 1H), 8.73 (d, *J* = 2.2 Hz, 1H), 8.55 – 8.48 (m, 1H), 7.79 (dd, *J* = 8.8, 2.2 Hz, 1H), 7.69 (app dt, *J* = 8.0, 2.0 Hz, 1H), 7.57 – 7.48 (m, 1H), 7.36 – 7.32 (m, 2H), 7.30 (d, *J* = 2.4 Hz, 1H), 5.05 (s, 1H). LRMS *m/z* (ESI+) 248.1 ([M+H]$^+$, 100%). IR (film): $\nu_{max}$ 2900, 1718, 1651, 1584, 1490, 1278, 1162, 1088, 1043, 924, 700 cm$^{-1}$. Spectroscopic data matched those in the literature.[4]

*tert*-Butyl 4-(4-(trifluoromethyl)phenyl)piperazine-1-carboxylate (Intermediate **Int5**)

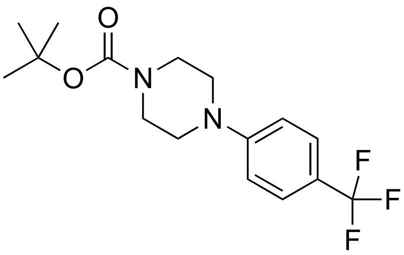

A 10 mL round-bottomed flask was charged with 1-Boc-piperazine (500 mg, 2.7 mmol, 1.0 equiv.), 4-bromobenzotrifluoride (0.40 mL, 2.8 mmol, 1.1 equiv.), palladium acetate (13 mg, 54 µmol, 0.02 equiv.) and BINAP (84 mg, 134 µmol, 0.05 equiv.). The reagents were dissolved in a 1:1 mixture of EtOAc and 1,4-dioxane (5.4 mL) with subsequent addition of $Cs_2CO_3$ (1.8 g, 5.4 mmol, 2.0 equiv.). The flask was fitted with a sealed reflux condenser and degassed with $N_2$ for 5 min. The stirring mixture was heated under reflux for 1 h at 85°C until precipitation of palladium black, indicating consumption of piperazine reagent. After an hour, the reaction mixture was allowed to cool to rt and quenched with aqueous 10% $NH_4Cl$ (aq) (3 mL) solution. The crude product was transferred to a silica gel plug in a sintered funnel and washed with $CH_2Cl_2$. The obtained filtrate was concentrated under reduced pressure and the residue purified by Biotage Selekt chromatography (eluent $CH_2Cl_2$) to afford *the title compound* as a white

solid (773 mg, 87%). m.p. 128–129°C (no lit. m.p.). $^1$H NMR (500 MHz, Chloroform-*d*) δ 7.52 (d, *J* = 8.6 Hz, 2H), 7.00 (d, *J* = 8.4 Hz, 2H), 3.63 (t, *J* = 5.2 Hz, 4H), 3.26 (t, *J* = 5.2 Hz, 4H), 1.49 (s, 9H). IR (film): $\nu_{max}$ 3000, 1673, 1613, 1420, 1326, 1233, 1159, 1069, 831 cm$^{-1}$. Spectroscopic data matched those in the literature.[4]

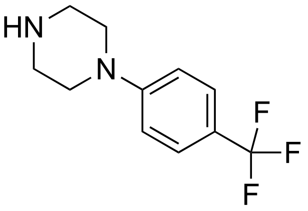

1-(4-(Trifluoromethyl)phenyl)piperazine (Intermediate **Int6**)

The Boc-protected piperazine intermediate **Int5** (569 mg, 1.7 mmol, 1.0 equiv.) was dissolved in $CH_2Cl_2$ (2.4 mL) and MeOH (0.14 mL). The reaction mixture was treated with TFA (0.40 mL, 5.2 mmol, 3.0 equiv.) and was left stirring overnight at rt. The solvents were removed under vacuum, 2 M NaOH (aq) was added and the solution was washed with EtOAc (3 × 8 mL). The phases were separated, the organic fractions combined, dried with $MgSO_4$, filtered and concentrated under reduced pressure to afford *the title compound* as a tan solid (278 mg, 70%) that was carried forward to the next synthetic step without further purification. m.p. 97–99°C (no lit. m.p.). $^1$H NMR (400 MHz, Chloroform–*d*) δ 7.49 (d, *J* = 8.7 Hz, 2H), 6.93 (d, *J* = 8.7 Hz, 2H), 3.25 (t, *J* = 5.2 Hz, 4H), 3.06 (t, *J* = 5.2 Hz, 4H). 2.01 (s, 1H). LRMS *m/z* (ESI+) 231.1 ([M+H]$^+$, 100%). IR (film): $\nu_{max}$ 3300, 2850, 1669, 1613, 1520, 1326, 1241, 1103, 1069, 909, 827 cm$^{-1}$. Spectroscopic data matched those in the literature.[4]

(4-Bromophenyl)(pyridin-3-yl)methanol (Intermediate **Int7**)

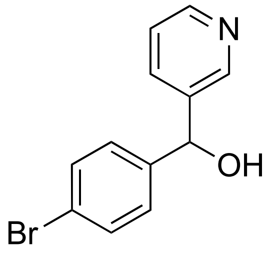

Prepared according to *General Synthetic Procedure A*, using 4-bromobenzaldehyde (10 g, 0.054 mol) to give *the title compound* as an orange solid (13 g, 95%), used without further purification. $^1$H NMR (500 MHz, Acetone-$d_6$) δ 8.63 (d, *J* = 2.3 Hz, 1H), 8.44 (dd, *J* = 4.7, 1.7 Hz, 1H), 7.77 – 7.69 (m, 1H), 7.55 – 7.47 (m, 2H), 7.44 – 7.36 (m, 2H), 7.30 (ddd, *J* = 7.8, 4.8, 0.9 Hz, 1H), 5.92 (s, 1H); $^{13}$C NMR (126 MHz, Acetone-$d_6$) δ 148.5, 148.3, 144.2, 140.2, 133.7, 131.3, 128.4, 123.2, 120.5, 72.5; LRMS *m/z* (ESI) 322 (100%), 320 (95%), 266 ([M+H]$^+$, 28%), 264 ([M+H]$^+$, 27%); HRMS (ESI) calcd. for $C_{12}H_{10}{}^{81}BrNONa^+$ 287.98230, $C_{12}H_{10}{}^{79}BrNONa^+$ 285.98435 ([M+Na]$^+$), found 287.98208, 285.98414; IR (film): $\nu_{max}$ 3149 (br), 2856, 1588, 1578, 1486, 1474, 1424, 1396 cm$^{-1}$.[1]

(4-Bromophenyl)(pyridin-3-yl)methanone (Intermediate **Int8**)

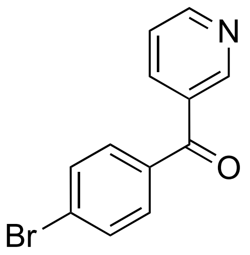

Prepared according to *General Synthetic Procedure D*, using activated manganese dioxide (21 g, 0.24 mol) and a solution of **Int7** (12 g, 0.045 mol) in DCM (75 mL). The crude mixture was purified by column chromatography to give *the title compound* as a cream solid (7.8 g, 69 %) and recovered **Int7** as an orange solid (1.4 g, 12%). $^1$H NMR (500 MHz, Acetone-$d_6$) δ 8.94 (dd, $J$ = 2.3, 0.9 Hz, 1H), 8.83 (dd, $J$ = 4.9, 1.7 Hz, 1H), 8.15 (dt, $J$ = 7.9, 2.0 Hz, 1H), 7.86 – 7.69 (m, 4H), 7.58 (ddd, $J$ = 7.9, 4.8, 0.9 Hz, 1H); $^{13}$C NMR (126 MHz, Acetone-$d_6$) δ 193.5, 153.0, 150.4, 136.8, 136.0, 132.8, 131.9, 131.6, 127.4, 123.4; LRMS *m/z* (ESI) 286 ([M+Na]$^+$, 100%), 284 ([M+Na]$^+$, 79%), 264 ([M+H]$^+$, 45%), 262 ([M+H]$^+$, 44%); HRMS (ESI) calcd. for $C_{12}H_8{}^{81}BrNONa^+$ 285.96665 and $C_{12}H_8{}^{79}BrNONa^+$ 283.96870 ([M+Na]$^+$), found 285.96649, 283.96851; IR (film): $\nu_{max}$ 1650 (s), 1581, 1479, 1414, 1393, 1337 cm$^{-1}$.[1]

(4-Bromo-2-fluorophenyl)(pyridin-3-yl)methanol (Intermediate **Int9**)

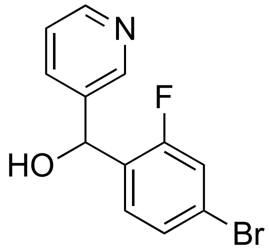

Prepared according to *General Synthetic Procedure A*, using 4-bromo-2-fluorobenzaldehyde (5.6 g, 27 mmol) in tetrahydrofuran (20 mL) to give *the title compound* as a reddish brown waxy solid (7.3 g, 96%), used without further purification. $^1$H NMR (500 MHz, Acetone-$d_6$) δ 8.62 (d, $J$ = 2.4 Hz, 1H), 8.46 (dd, $J$ = 4.8, 1.7 Hz, 1H), 7.74 (app dt, $J$ = 7.9, 2.5, 1.3 Hz, 1H), 7.70 – 7.59 (m, 1H), 7.44 (dd, $J$ = 8.4, 1.9 Hz, 1H), 7.37 – 7.24 (m, 2H), 6.13 (s, 1H); $^{13}$C NMR (126 MHz, Acetone-$d_6$) δ 160.3 (d, $^1J_{CF}$ = 250 Hz), 149.6, 149.2 (d, $^4J_{CF}$ = 1.6 Hz), 139.8, 134.6, 132.2 (d, $^2J_{CF}$ = 13.5 Hz), 130.2 (d, $^3J_{CF}$ = 4.9 Hz), 128.7 (d, $^4J_{CF}$ = 3.6 Hz), 124.2, 121.7 (d, $^3J_{CF}$ = 9.7 Hz), 119.5 (d, $^2J_{CF}$ = 25.1 Hz), 67.7; $^{19}$F NMR (471 MHz, Acetone-$d_6$) δ –116.62; LRMS *m/z* (ESI) 340 (87%), 338 (100%), 284 ([M+H]$^+$, 20%), 282 ([M+H]$^+$, 18%); HRMS (ESI) calcd. for $[C_{12}H_{10}{}^{81}BrFNO]^+$ 283.99093 and $[C_{12}H_{10}{}^{79}BrFNO]^+$ 281.99298 ([M+H]$^+$), found 283.99035, 281.99242; IR (film): $\nu_{max}$ 3123 (br), 2923, 2853, 1603, 1574, 1479, 1425, 1398 cm$^{-1}$. Spectroscopic data matched those in the literature.[1]

(4-Bromo-2-fluorophenyl)(pyridin-3-yl)methanone (Intermediate **Int10**)

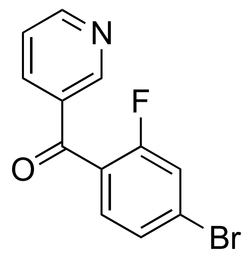

Prepared according to *General Synthetic Procedure D*, using activated manganese dioxide (5.8 g, 25 mmol) and a solution of **Int9** (6.7 g, 76 mmol) in $CH_2Cl_2$ (55 mL). The crude mixture was purified by column chromatography (hexane/ethyl acetate) to give *the title compound* as a cream solid (0.83 g, 12%) and recovered **Int9** as a reddish brown waxy solid (3.4 g, 50%). $^1$H NMR (500 MHz, Acetone-$d_6$) δ 8.98 – 8.93 (m, 1H), 8.84 (dd, $J$ = 4.8, 1.7 Hz, 1H), 8.19 (app dt, $J$ = 8.0, 2.7, 1.4 Hz, 1H), 7.74 – 7.45 (m, 4H); $^{13}$C NMR (126 MHz, Acetone-$d_6$) δ 190.6, 160.0 (d, $^1J_{CF}$ = 256 Hz), 153.8, 150.4 (d, $^4J_{CF}$ = 1.7 Hz), 136.5, 132.8, 132.3 (d, $^3J_{CF}$ = 3.3 Hz), 128.3 (d, $^4J_{CF}$ = 3.7 Hz), 126.4 (d, $^3J_{CF}$ = 9.6 Hz), 125.3 (d, $^2J_{CF}$ = 14.5 Hz), 123.7, 120.0 (d, $^2J_{CF}$ = 25.2 Hz); $^{19}$F NMR (471 MHz, Acetone-$d_6$) δ –109.99; LRMS *m/z* (ESI) 340 (87%), 338 (100%), 304 ([M+Na]$^+$, 27%), 302 ([M+Na]$^+$, 21%), 282 ([M+H]$^+$, 27%), 280 ([M+H]$^+$, 23%); HRMS (ESI) calcd. for $[C_{12}H_8{}^{81}BrFNO]^+$ 281.97528 ([M+H]$^+$) and $[C_{12}H_8{}^{79}BrFNO]^+$ 279.97733, found 281.97476, 279.97677; IR (film): $\nu_{max}$ 3007, 1654 (s), 1584, 1417, 1395 cm$^{-1}$. Spectroscopic data matched those in the literature.[1]

(4-Chloro-2-fluorophenyl)(pyridin-3-yl)methanone **(**Intermediate **Int11)**

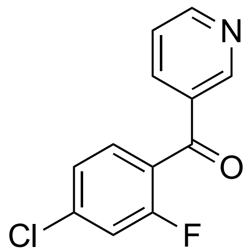

Prepared according to *General Synthetic Procedure D*, using **Int1** (310 mg, 1.3 mmol), to obtain *the title compound* as a light straw-coloured solid (290 mg, 95%), used without further purification. $^1$H NMR (300 MHz, Acetone-$d_6$) δ 9.0 (s, 1H), 8.8 (dd, $J$ = 4.8, 1.7 Hz, 1H), 8.2 (dd, $J$ = 8.0, 1.7 Hz, 1H), 7.7 (app t, $J$ = 8.1 Hz, 1H), 7.6 (dd, $J$ = 8.0, 4.8 Hz, 1H), 7.5 – 7.4 (m, 2H); $^{13}$C NMR (75 MHz, Acetone-$d_6$) δ 191.4, 161.1 (d, $^1J_{CF}$ = 254.9 Hz), 154.7, 151.3 (d, $^4J_{CF}$ = 1.7 Hz), 139.5 (d, $^3J_{CF}$ = 10.5 Hz), 137.4 (d, $^4J_{CF}$ = 1.3 Hz), 133.7, 133.1 (d, $^3J_{CF}$ = 3.6 Hz), 126.2 (d, $^4J_{CF}$ = 3.6 Hz), 125.8 (d, $^2J_{CF}$ = 14.4 Hz), 124.5, 118.0 (d, $^2J_{CF}$ = 25.6 Hz); LRMS *m/z* (ESI) 238 ([M+H]$^+$, 40%) and 236 ([M+H]$^+$, 100%); HRMS (ESI) calcd. for $[C_{12}H_7{}^{37}ClFNONa]^+$ 260.00467 and $[C_{12}H_7{}^{35}ClFNONa]^+$ 258.00762, found 260.00648 and 258.00942. IR (film): $\nu_{max}$ 3105, 1655, 1602, 1401, 1295, 937, 749 cm$^{-1}$.

(2-Bromophenyl)(4-chlorophenyl)methanone (Intermediate **Int12**)

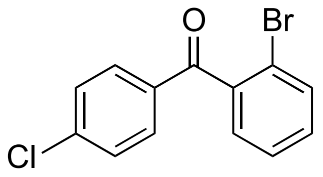

Prepared according to *General Synthetic Procedure F*, using 2-bromobenzoyl chloride (21 g, 96 mmol). The crude mixture concentrated under reduced pressure to give *the title compound* as a brown oil, used without further purification (28 g, 99%). $^{1}$H NMR (500 MHz, Chloroform-*d*) δ 7.82 – 7.72 (m, 2H), 7.65 (d, *J* = 7.9, 1.1 Hz, 1H), 7.51 – 7.31 (m, 5H); $^{13}$C NMR (126 MHz, $CDCl_3$) δ 194.79, 140.46, 140.37, 134.67, 133.42, 131.66, 131.53, 129.18, 129.10, 127.49, 119.61, 77.16; LRMS *m/z* (ESI) 619 ([2M+Na]$^+$, 3%), 617 ([2M+Na]$^+$, 31%), 615 ([2M+Na]$^+$, 65%), 613 ([2M+Na]$^+$, 53%), 611 ([2M+Na]$^+$, 26%), 321 ([M+Na]$^+$, 22%), 319 ([M+Na]$^+$, 100%), 317 ([M+Na]$^+$, 81%); HRMS (ESI) calcd. for $[C_{13}H_8{}^{81}Br^{37}ClONa]^+$ 320.92948, $[C_{13}H_8{}^{81}Br^{35}ClONa]^+$ 318.93243, $[C_{13}H_8{}^{79}Br^{37}ClONa]^+$ 318.93152, $[C_{13}H_8{}^{79}Br^{35}ClONa]^+$ 316.93447 ([M+H]$^+$) found 320.92942, 318.93250, 316.93451; IR (film): $\nu_{max}$ 3086, 3053, 1662 (s), 1583, 1570, 1486, 1463 cm$^{-1}$. Spectroscopic data matched those in the literature.[5]

(4-Chlorophenyl)(p-tolyl)methanone (Intermediate **Int13**)

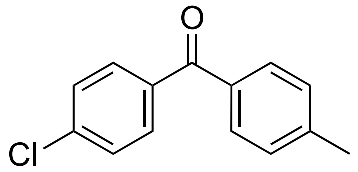

Prepared according to *General Synthetic Procedure F* using 4-methylbenzoyl chloride (3.4 mL, 25 mmol). The crude mixture concentrated under reduced pressure to give *the title compound* as a brown oil, used without further purification (0.98 g, 17%). $^{1}$H NMR (500 MHz, Acetone-$d_6$) δ 7.83 – 7.74 (m, 2H), 7.72 – 7.66 (m, 2H), 7.62 – 7.55 (m, 2H), 7.37 (app dt, *J* = 7.8, 0.8 Hz, 2H), 2.43 (s, 3H); $^{13}$C NMR (126 MHz, Acetone) δ 195.1, 144.3, 138.7, 137.5, 135.5, 132.2, 130.8, 130.0, 129.4, 21.6; LRMS *m/z* (ESI) 255 ([M+Na]$^+$, 40%), 253 ([M+Na]$^+$, 100%); HRMS (ESI) calcd. for $[C_{14}H_{11}{}^{37}ClNaO]^+$ 255.03611 and $[C_{14}H_{11}{}^{35}ClNaO]^+$ 253.03906 ([M+Na]$^+$), found 255.03635 and 253.03923. Spectroscopic data matched those in the literature.[5]

(2-Fluorophenyl)(pyridin-3-yl)methanone (Intermediate **Int14**)

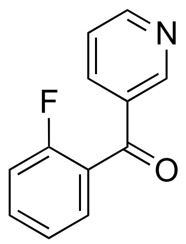

Prepared according to *General Synthetic Procedure D*, using (2-fluorophenyl)(pyridin-3-yl)methanol (5.0 g, 25 mmol). The crude mixture was purified by column chromatography (hexane/ethyl acetate) to give *the title compound* as a straw-coloured solid (1.9 g, 39%). $^1$H NMR (300 MHz, Chloroform-*d*) δ 8.98 (d, $J$ = 1.2 Hz, 1H), 8.80 (dd, $J$ = 4.9, 1.7 Hz, 1H), 8.22 – 8.03 (m, 1H), 7.59 (app dtd, $J$ = 14.0, 7.3, 1.8 Hz, 2H), 7.44 (ddd, $J$ = 8.0, 4.9, 1.0 Hz, 1H), 7.31 (app td, $J$ = 7.6, 1.1 Hz, 1H), 7.18 (ddd, $J$ = 9.7, 8.3, 1.0 Hz, 1H); $^{13}$C NMR (75 MHz, Chloroform-*d*) δ 191.7, 160.2 (d, $^1J_{FC}$ = 253.3 Hz), 153.4, 150.7 (d, $^5J_{FC}$ = 2.1 Hz), 136.7 (d, $^4J_{FC}$ = 1.3 Hz), 134.1 (d, $^3J_{FC}$ = 8.6 Hz), 133.0 (d, $^4J_{FC}$ = 1.0 Hz), $^4J_{FC}$ (d, $J$ = 2.5 Hz), 125.8 (d, $^2J_{FC}$ = 14.0 Hz), 124.6 (d, $^3J_{FC}$ = 3.5 Hz), 123.4, 116.4 (d, $^2J_{FC}$ = 21.7 Hz); LRMS *m/z* (ESI) 202 ((M+H)$^+$); HRMS (ESI) calcd. for $[C_{12}H_8FNNaO]^+$ 224.04821 ([M+Na]$^+$), found 224.04831; IR (film): $\nu_{max}$ 2970, 1666 (s), 1610, 1584, 1541, 1298, 928 and 750 cm$^{-1}$. Literature contains only incomplete data.[6]

1–((4–Chloro–2–fluorophenyl)(pyridin–3–yl)methyl)piperazine **(**Intermediate **Int15)**

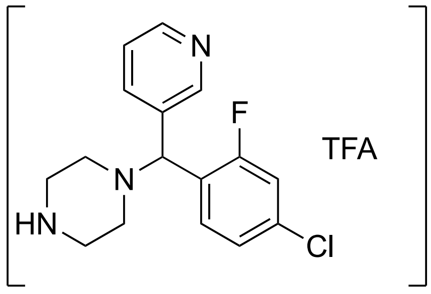


The Boc–protected precursor (*tert*-butyl 4-((4-chloro-2-fluorophenyl)(pyridin-3-yl)methyl)piperazine-1-carboxylate, **MYOS_00011**,[1] 810 mg, 2.0 mmol, 1.0 equiv**.**) dissolved in a mixture of DCM (9 mL) and MeOH (3 drops) was cooled to –5°C and TFA (1.5 mL, 20 mmol, 10 equiv.) was added dropwise. The mixture was stirred for 1 h at –5°C, left overnight to warm to rt, azeotroped with DCM (3 × 10 mL) and concentrated under reduced pressure to give *the title compound* as an orange oil (497 mg, 82%). The crude TFA salt was carried forward to the next synthetic step without further purification. $^1$H NMR (500 MHz, Chloroform–*d*) δ 9.12 (d, $J$ = 2.0 Hz, 1H), 8.79–8.73 (m, 1H), 8.29 (d, $J$ = 8.1 Hz, 1H), 7.84 (dd, $J$ = 8.1, 5.5 Hz, 1H), 7.31 (app t, $J$ = 7.9 Hz, 1H), 7.23 (dd, $J$ = 8.5, 2.1 Hz, 1H), 7.16 (dd, $J$ = 10.0, 2.0 Hz, 1H), 5.09 (s, 1H), 3.37–3.33 (m, 4H), 2.85–2.66 (m, 4H), 1.25 (s, 1H). LRMS *m/z* (ESI+) 306.0 ([M+H]$^+$, 100%). IR (film): $\nu_{max}$ 2988, 1685, 1500, 1405, 1200, 1130, 910, 850, 710 cm$^{-1}$. Spectroscopic data matched those in the literature.[3]

The neutral compound (**MYOS_00189**) could be isolated using sodium hydrogen carbonate solution and extraction into ethyl acetate. $^{1}$H NMR (300 MHz, Chloroform-d) δ 8.61 (d, *J* = 2.2 Hz, 1H), 8.43 (dt, *J* = 4.8, 1.3 Hz, 1H), 7.72 – 7.62 (m, 1H), 7.50 (t, *J* = 8.0 Hz, 1H), 7.19 (dd, *J* = 7.9, 4.8 Hz, 1H), 7.10 (dd, *J* = 8.4, 2.0 Hz, 1H), 7.00 (dt, *J* = 9.9, 1.4 Hz, 1H), 4.65 (s, 1H), 2.86 (t, *J* = 4.9 Hz, 4H), 2.44 – 2.24 (m, 4H).

*tert*-Butyl 4-((4-chloro-2-fluorophenyl)(pyridin-3-yl)methyl)-2,2-dimethylpiperazine-1-carboxylate (Intermediate **Int16**)

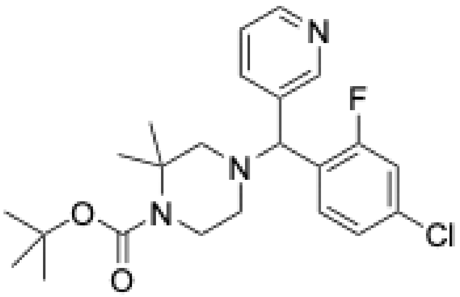

Prepared according *General Synthetic Procedure B* using anhydrous triethylamine (0.7 mL, 5.1 mmol), dried potassium iodide (catalytic amount) and a solution of **Int2** (0.66 g) and *tert*-butyl 2,2-dimethylpiperazine-1-carboxylate (3.6 g, 4.3 mmol) in anhydrous acetonitrile (4.5 mL) to give a crude orange oil that was purified with column chromatography (ethyl acetate/hexane) to give *the title compound* as an amber resin (0.70 g, 64%). **$^{1}$H NMR** (500 MHz, Chloroform-*d*) δ 8.64 (d, *J* = 2.2 Hz, 1H), 8.46 (dd, *J* = 4.8, 1.7 Hz, 1H), 7.70 (dt, *J* = 7.9, 2.0 Hz, 1H), 7.54 (t, *J* = 8.0 Hz, 1H), 7.22 (ddd, *J* = 7.9, 4.7, 0.8 Hz, 1H), 7.15 – 7.08 (m, 1H), 7.02 (dd, *J* = 9.9, 2.1 Hz, 1H), 4.58 (s, 1H), 3.64 – 3.15 (m, 2H), 2.55 – 2.35 (m, 2H), 2.24 – 2.08 (m, 2H), 1.43 (s, 9H), 1.37 (d, *J* = 1.8 Hz, 6H). **$^{13}$C NMR** (126 MHz, Chloroform-*d*) δ 160.4 (d, *J* = 249.9 Hz), 156.4, 149.7, 149.1, 136.6, 135.4, 133.9 (d, *J* = 10.5 Hz), 129.3 (d, *J* = 4.7 Hz), 127.3 (d, *J* = 12.7 Hz), 123.8, 116.7 (d, *J* = 25.9 Hz), 79.9, 65.0, 64.1 (d, *J* = 1.6 Hz), 54.9, 51.7, 43.0, 28.6, 25.3, 25.0. **LRMS** *m/z* (APCI) 436 ($[M+H]^+$, 25%), 434 ($[M+H]^+$, 66%), 380 ($[M–(C_4H_9)^{+}+2H^{+}]^{+}$, 47%), 378 ($[M–(C_4H_9)^{+}+2H^{+}]^{+}$, 100%), 336 ($[M–(C_5H_9O_2)^{+}+2H^{+}]^{+}$, 12%), 334 ($[M–(C_5H_9O_2)^{+}+2H^{+}]^{+}$, 38%), 222 ($[M–(C_{11}H_{21}N_2O_2)^{-}]^{+}$, 10%), 220 ($[M–( C_{11}H_{21}N_2O_2)^{-}]^{+}$, 17%). **HRMS** (ESI) calcd. for $C_{23}H_{29}{}^{37}ClFN_3O_2Na^+$ 458.18005 and $C_{23}H_{29}{}^{35}ClFN_3O_2Na^+$ 456.18300 ($[M+Na]^+$), found 458.17948, 456.18244. **IR** (film): $\nu_{max}$ 2972, 1701 (s), 1481, 1343 cm$^{-1}$. This compound, and several others below, were previously reported in a thesis[7] but the data are reproduced here in full.

*tert*-Butyl 4-((4-chloro-2-fluorophenyl)(pyridin-3-yl)methyl)-3-methylpiperazine-1-carboxylate (Intermediate **Int17**).

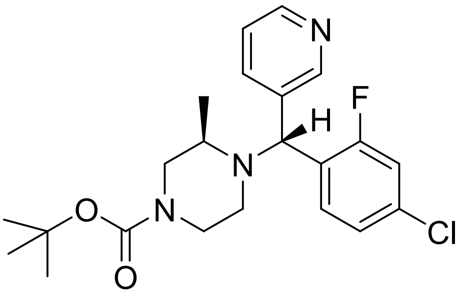

Prepared according to *General Synthetic Procedure B*, using **Int2** (0.15 g, 0.6 mmol) and thionyl chloride (0.35 mL, 4.7 mmol), anhydrous triethylamine (0.7 mL, 5.1 mmol), dried potassium iodide (catalytic amount) and *tert*-butyl 3-methylpiperazine-1-carboxylate (0.19 g, 1.0 mmol) in anhydrous acetonitrile (4.5 mL) to give a crude orange oil. The crude oil was purified with column chromatography (ethyl acetate/hexane) to give two diastereomers of *the title compound* (**Int17a** (*syn, or (R,R) and (S,S)*) and **Int17b** (*anti, or (R,S) and (S,R)*), and recovered **Int2** as a waxy brown solid (86 mg, 57%).

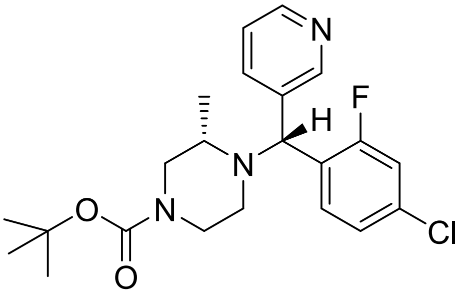

**Isomer Int17a** (57 mg, 22%) as a brown oil. **$^1$H NMR** (400 MHz, Chloroform-*d*) δ 8.66 (d, *J* = 2.1 Hz, 1H), 8.55 – 8.07 (m, 1H), 7.73 (dt, *J* = 8.0, 1.9 Hz, 1H), 7.38 (t, *J* = 8.0 Hz, 1H), 7.22 (dd, *J* = 7.9, 4.8 Hz, 1H), 7.09 (m, 2H), 5.16 (s, 1H), 3.70 (s, 1H), 3.58 – 3.44 (m, 1H), 3.30 (m, 1H), 3.15 (s, 1H), 2.86 (s, 1H), 2.52 (m, 1H), 2.26 (s, 1H), 1.43 (s, 9H), 1.02 (d, *J* = 6.4 Hz, 3H). **$^{13}$C NMR** (75 MHz, Chloroform-*d*) δ 160.4 (d, *J* = 249.5 Hz), 155.1, 149.6, 148.8, 137.4, 135.4, 134.0 (d, *J* = 10.6 Hz), 130.7 (d, *J* = 4.8 Hz), 125.8 (d, *J* = 13.9 Hz), 125.1 (d, *J* = 3.5 Hz), 123.7, 116.8 (d, *J* = 26.5 Hz), 79.7, 59.1, 50.2, 44.3, 28.5, 10.1 (2 obscured signals). **LRMS** *m/z* (ESI) 444 ([M+Na]$^+$, 36%), 442 ([M+Na]$^+$, 100%), 422 ([M+H]$^+$, 7%), 420 ([M+H]$^+$, 16%). **HRMS** (ESI) calcd. for $C_{22}H_{27}{}^{37}ClFN_3O_2Na^+$ 444.16440 and $C_{22}H_{27}{}^{35}ClFN_3O_2Na^+$ 442.16735 ([M+Na]$^+$), found 444.16379, 442.16670. **IR** (film): $\nu_{max}$ 2974, 1689 (s), 1603, 1574, 1478, 1423, 1379 cm$^{-1}$.

**Isomer Int17b** (61 mg, 23%) as a brown oil. **$^1$H NMR** (400 MHz, Chloroform-*d*) δ 8.64 (d, *J* = 2.1 Hz, 1H), 8.47 (dd, *J* = 4.8, 1.6 Hz, 1H), 7.92 – 7.56 (m, 2H), 7.23 (dd, *J* = 7.9, 4.8 Hz, 1H), 7.17 – 7.10 (m, 1H), 6.99 (dd, *J* = 10.0, 2.1 Hz, 1H), 4.96 (s, 1H), 4.33 – 3.46 (m, 2H), 3.41 – 2.75 (m, 3H), 2.77 – 2.29 (m, 2H), 1.43 (s, 9H), 0.94 (d, *J* = 6.5 Hz, 3H). **$^{13}$C NMR** (75 MHz, Chloroform-*d*) δ 160.4 (d, *J* = 250.0 Hz), 155.2, 149.8, 149.0, 135.9, 133.7 (d, *J* = 10.6 Hz), 129.0, 128.3 (d, *J* = 12.0 Hz), 125.2 (d, *J* = 3.6 Hz), 123.7, 116.6 (d, *J* = 25.8 Hz), 79.7, 60.1, 49.5, 43.5, 28.5, 8.4 (3 obscured signals). **LRMS** *m/z* (ESI) 444 ([M+Na]$^+$, 37%), 442 ([M+Na]$^+$, 100%), 422 ([M+H]$^+$, 3%), 420 ([M+H]$^+$, 12%). **HRMS** (ESI) calcd. for

$C_{22}H_{27}{}^{37}ClFN_3O_2Na^+$ 444.16440 and $C_{22}H_{27}{}^{35}ClFN_3O_2Na^+$ 442.16735 ($[M+Na]^+$), found 444.16445, 442.16736. **IR** (film): $\nu_{max}$ 2967, 1692 (s), 1605, 1577, 1473, 1420, 1422, 1379 $cm^{-1}$.

3-((2-Bromophenyl)(4-chlorophenyl)(methoxy)methyl)pyridine, **MYOS_00009**

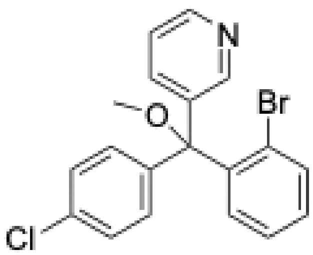

Compound **MYOS_00012** (100 mg, 0.31 mmol) in THF (2 mL) was slowly added to a suspension of sodium hydride (60% w/w in mineral oil, 23 mg, 0.935 mmol) in THF (5 mL) at 0°C. After 1 h at 0°C, iodomethane (0.03 mL, 0.47 mmol) was added and the mixture was allowed to warm to rt overnight. The mixture was quenched with water and extracted with diethyl ether. The organic layers were combined, dried ($MgSO_4$) and concentrated under reduced pressure. The crude product was purified with column chromatography (ethyl acetate/hexane) to give *the title compound* as a pale straw-coloured oil (34 mg, 33%). **$^1$H NMR** (500 MHz, Acetone-$d_6$) δ 8.76 – 8.57 (m, 1H), 8.46 (dd, $J$ = 4.7, 1.5 Hz, 1H), 7.80 (m, 2H), 7.68 (dd, $J$ = 7.9, 1.3 Hz, 1H), 7.57 – 7.09 (m, 7H), 3.11 (s, 3H). **$^{13}$C NMR** (126 MHz, Acetone-$d_6$) δ 150.4, 149.0, 141.9, 140.7, 139.1, 136.7, 136.2, 133.5, 132.7, 131.0, 130.8, 128.9, 128.3, 124.5, 123.6, 87.0, 53.0. **LRMS** *m/z* (ESI) 414 ($[M+Na]^+$, 6%), 412 ($[M+Na]^+$, 32%), 410 ($[M+Na]^+$, 29%), 392 ($[M+H]^+$, 29%), 390 ($[M+H]^+$, 100%), 388 ($[M+H]^+$, 70%). **HRMS** (ESI) calcd. for $C_{19}H_{15}{}^{81}Br^{37}ClNONa^+$ 413.98733, $C_{19}H_{15}{}^{81}Br^{35}ClNONa^+$ 411.98937, $C_{19}H_{15}{}^{79}Br^{37}ClNONa^+$ 411.99028 and $C_{19}H_{15}{}^{79}Br^{35}ClNONa^+$ 409.99232 ($[M+Na]^+$), found 413.98710, 411.99012, 409.99208. **IR** (film): $\nu_{max}$ 3057, 2937, 2827, 1702, 1488, 1417 $cm^{-1}$.

2-(4-Chlorophenyl)-2-(pyridin-3-yl)-1-(4-(4-(trifluoromethyl)phenyl)piperazin-1-yl)ethan-1-one, **MYOS_00010**

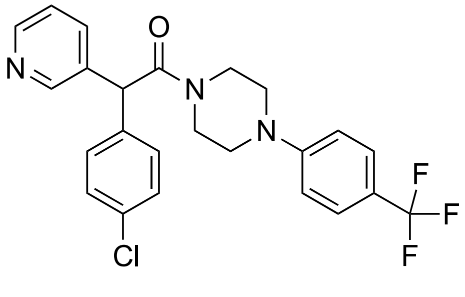

Carboxylic acid **Int4** (88 mg, 355 µmol, 1.0 equiv.), HBTU (136 mg, 355 µmol, 1.0 equiv.) and piperazine substrate **Int6** (82 mg, 355 µmol, 1.0 equiv.) were dissolved in *N,N*-dimethylformamide (1 mL) and then treated with diisopropylethylamine (0.10 mL, 533 µmol, 1.5 equiv.). The reaction mixture was stirred for 3 h at rt. The crude product was partitioned between EtOAc and $H_2O$. The two layers were separated and the organic phase was washed

with $H_2O$ (3 × 5 mL), brine (1 × 5 mL), dried with $MgSO_4$, filtered and concentrated under reduced pressure. The crude residue was purified by Biotage Selekt chromatography (reverse phase 30 g Sfar column, 5–95%, MeOH in $H_2O$) to afford *the title compound* as a white foam (36 mg, 22%). m.p. 143–147°C (no lit. m.p.). $^{1}$H NMR (500 MHz, Chloroform–*d*): δ ppm 8.52 (dd, *J* = 4.8, 1.6 Hz, 1H), 8.50 (d, *J* = 2.1 Hz, 1H), 7.59 (app dt, *J* = 8.0, 2.0 Hz, 1H), 7.52–7.49 (m, 2H), 7.37–7.35 (m, 2H), 7.24–7.22 (m, 2H), 6.89 (d, *J* = 8.7 Hz, 2H), 5.23 (s, 1H), 3.97–3.93 (m, 1H), 3.84–3.79 (m, 1H), 3.69–3.62 (m, 2H), 3.36–3.24 (m, 2H), 3.17–3.13 (m, 1H), 3.02–2.97 (m, 1H). LRMS *m/z* (ESI+) 460.0 ($[M+H]^+$, 100%). IR: $\nu_{max}$ 1640, 1613, 1524, 1427, 1326, 1226, 1155, 1103, 1069, 790, 708 $cm^{-1}$. Spectroscopic data matched those in the literature.[4]

(2-Bromophenyl)(4-chlorophenyl)(pyridin-3-yl)methanol, **MYOS_00012**

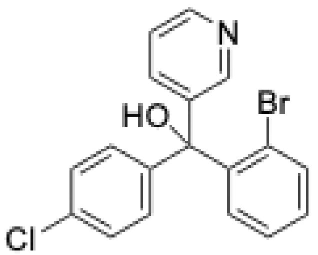


Prepared according to *General Synthetic Procedure A*, using bromopyridine (2.95 mL, 30 mmol), *n*-butyllithium (12.5 mL, 20 mmol) and **Int12** (3.0 g, 10 mmol). The reaction was allowed to proceed for 2 h at –78°C then quenched with water at –78°C. The crude product was purified by column chromatography (ethyl acetate/hexane) to give *the title compound* as a pale straw-coloured fine solid (2.3 g, 62%) and recovered **9** as a brown oil (0.34 g, 11%). **m.p.** 64.7–65.8°C. **$^{1}$H NMR** (500 MHz, Acetone-$d_6$) δ 8.86 – 8.19 (m, 2H), 7.83 – 7.55 (m, 2H), 7.48 – 7.24 (m, 7H), 7.12 (dd, *J* = 7.8, 1.9 Hz, 1H). **$^{13}$C NMR** (126 MHz, Acetone-$d_6$) δ 150.2, 149.1, 145.1, 145.0, 141.9, 136.3, 136.1, 133.6, 131.9, 130.7, 130.6, 128.9, 128.0, 124.0, 123.6, 81.6. **LRMS** *m/z* (ESI) 511 (29%), 509 (100%), 453 (24%), 451 (58%), 378 ($[M+H]^+$, 19%), 376 ($[M+H]^+$, 87%), 374 ($[M+H]^+$, 69%). **HRMS** (ESI) calcd. for $C_{18}H_{14}{}^{81}Br^{37}ClNO^+$ 377.98973, $C_{18}H_{14}{}^{81}Br^{35}ClNO^+$ 375.99268, $C_{18}H_{14}{}^{79}Br^{37}ClNO^+$ 375.99178 and $C_{18}H_{14}{}^{79}Br^{35}ClNO^+$ 373.99473 ($[M+H]^+$), found 377.98918, 375.99205, 373.99418. **IR** (film): $\nu_{max}$ 3057 (br), 2775, 1734, 1586, 1487, 1462, 1419, 1399 $cm^{-1}$.[5]

Methyl 4-((4-chloro-2-fluorophenyl)(pyridin-3-yl)methyl)piperazine-1-carboxylate, **MYOS_00015**

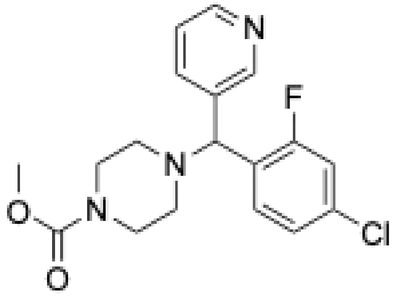

Prepared according to *General Synthetic Procedure B*, using TFA (0.9 mL, 12 mmol) and **Int15** (0.36 g, 0.9 mmol) in DCM (5 mL), anhydrous triethylamine (2.5 mL, 18 mmol), methyl chloroformate (0.060 mL 0.77 mmol). The crude product was purified with column chromatography (ethyl acetate/hexane) to give *the title compound* as an amber resin (0.17 g, 61%). **$^1$H NMR** (600 MHz, Chloroform-*d*) δ 8.62 (d, *J* = 2.1 Hz, 1H), 8.45 (dd, *J* = 5.0, 1.6 Hz, 1H), 7.67 (dd, *J* = 8.0, 2.0 Hz, 1H), 7.48 (t, *J* = 8.0 Hz, 1H), 7.21 (ddd, *J* = 7.9, 4.9, 0.8 Hz, 1H), 7.15 – 7.08 (m, 1H), 7.01 (dd, *J* = 9.9, 2.1 Hz, 1H), 4.68 (s, 1H), 3.65 (s, 3H), 3.46 (s, 4H), 2.69 – 2.05 (m, 4H). **$^{13}$C NMR** (151 MHz, Chloroform-*d*) δ 160.4 (d, *J* = 250.0 Hz), 155.8, 149.6, 148.9, 136.3, 135.6, 134.0 (d, *J* = 10.5 Hz), 129.5 (d, *J* = 4.8 Hz), 126.7 (d, *J* = 12.8 Hz), 125.2 (d, *J* = 3.6 Hz), 123.8, 116.7 (d, *J* = 26.0 Hz), 64.4, 52.7, 51.4, 43.8. **LRMS** *m/z* (ESI) 388 ([M+Na]$^+$, 36%), 386 ([M+Na]$^+$, 100%), 366 ([M+H]$^+$, 12%), 364 ([M+H]$^+$, 32%). **HRMS** (ESI) calcd. for $C_{18}H_{19}{}^{37}ClFN_3O_2Na^+$ 388.10180 and $C_{18}H_{19}{}^{35}ClFN_3O_2Na^+$ 386.10475 ([M+Na]$^+$), found 388.10185, 386.10483. **IR** (film): $\nu_{max}$ 2958, 2816, 1697 (s), 1607, 1578, 1472, 1444, 1408 cm$^{-1}$.

3-((4-Chloro-2-fluorophenyl)(piperidin-1-yl)methyl)pyridine, **MYOS_00016**

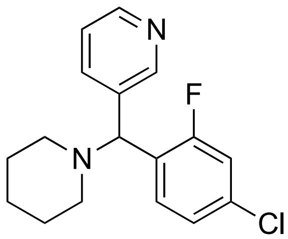

Prepared according to *General Synthetic Procedure* B using **Int2** (231 mg, 902 μmol), piperidine (0.12 mL, 1.4 mmol), anhydrous $Et_3N$ (0.25 mL, 1.8 mmol) and KI (catalytic amount) to give the crude product (159 mg) which was purified by manual column chromatography on silica (25–75%, EtOAc in hexanes) to afford *the title compound* as a light-yellow waxy liquid (92.0 mg, 34%). $^1$H NMR (400 MHz, Chloroform-*d*) δ 8.62 (d, *J* = 2.2 Hz, 1H), 8.45 (dd, *J* = 4.8, 1.7 Hz, 1H), 7.68 (app dt, *J* = 7.9, 2.1 Hz, 1H), 7.51 (app t, *J* = 8.1 Hz, 1H), 7.21 (dd, *J* = 7.9, 4.8 Hz, 1H), 7.12 (dd, *J* = 8.5, 2.1 Hz, 1H), 7.02 (dd, *J* = 9.9, 2.1 Hz, 1H), 4.67 (s, 1H), 2.72 – 2.04 (m, 4H), 1.67 – 1.52 (m, 4H), 1.50 – 1.39 (m, 2H). $^{13}$C NMR (126 MHz, Chloroform–*d*) δ 160.5 (d, $^1J_{CF}$ = 249.4 Hz), 149.8, 148.7, 137.0, 135.8, 133.7, 129.9 (d, $^3J_{CF}$ = 5.0 Hz), 127.4, 125.1, 123.8, 116.5 (d, $^2J_{CF}$ = 25.9 Hz), 65.0, 53.0, 26.0, 24.4. LRMS *m/z* (ESI) 305.2 ([M+H]$^+$, 100%). HRMS (ESI) calcd. for $[C_{17}H_{19}{}^{35}ClFN_2]^+$

305.1215, found 305.1220 [M+H]$^+$. IR (film): $\nu_{max}$ 2929, 2758, 1580, 1472, 1408, 1304, 1211, 1095, 1025, 894, 808 cm$^{-1}$.

(2-Bromophenyl)(4-chlorophenyl)(pyridin-3-yl)methanamine, **MYOS_00017**

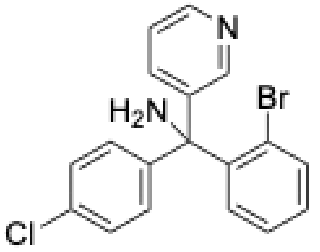


Thionyl chloride (0.15 ml, 1.5 mmol) was added to a solution of **MYOS_00012** (98 mg, 0.30 mmol) in dry DCM (10 ml) at 0°C. The mixture was allowed to warm to rt overnight then concentrated under reduced pressure. Ammonia (7 N in MeOH) (0.35 mL, 2.3 mmol) was added to the residue in DCM (10 mL) at 0°C and the mixture was allowed to warm to rt for 48 h. The reaction was quenched with water and extracted with DCM. The organic layers were combined, dried ($MgSO_4$) and concentrated under reduced pressure. The crude product was purified with column chromatography (ethyl acetate/hexane) to give *the title compound* as a pale straw-coloured oil (33 mg, 34%). **$^1$H NMR** (500 MHz, Acetone-$d_6$) δ 8.67 – 8.38 (m, 2H), 7.89 – 7.60 (m, 2H), 7.43 – 7.13 (m, 7H), 7.04 – 6.63 (m, 1H), 3.19 (s, 2H). **$^{13}$C NMR** (126 MHz, Acetone-$d_6$) δ 150.5, 148.7, 147.1, 146.3, 136.5, 136.1, 133.2, 131.8, 130.7, 130.0, 128.9, 128.1, 123.9, 67.0 (2 obscured signals). **LRMS** *m/z* (ESI) 399 ([M+Na]$^+$, 12%), 397 ([M+Na]$^+$, 42%), 395 ([M+Na]$^+$, 33%), 377 ([M+H]$^+$, 27%), 375 ([M+H]$^+$, 100%), 373 ([M+H]$^+$, 77%). **HRMS** (ESI) calcd. for $C_{18}H_{15}{}^{81}Br^{37}ClN_2^+$ 377.00572, $C_{18}H_{15}{}^{81}Br^{35}ClN_2^+$ 375.00867, $C_{18}H_{15}{}^{79}Br^{37}ClN_2^+$ 375.00776, $C_{18}H_{15}{}^{79}Br^{35}ClN_2^+$ 373.01071 ([M+H]$^+$), found 377.00517, 375.00804, 373.01017. **IR** (film): $\nu_{max}$ 3058, 2937, 2828, 1701, 1584, 1572, 1487, 1416 cm$^{-1}$.

4-((4-Chloro-2-fluorophenyl)(pyridin-3-yl)methyl)morpholine, **MYOS_00024**

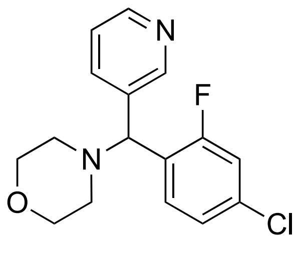


Prepared according to *General Synthetic Procedure B* using **Int2** (231 mg, 902 μmol), morpholine (0.12 mL, 1.4 mmol), anhydrous $Et_3N$ (0.25 mL, 1.8 mmol) and KI (15 mg, 10 mol%) to give the crude product (240 mg) which was purified by flash chromatography (25–75%, EtOAc in hexanes) to afford *the title compound* as an orange oil (123 mg, 45%).$^1$H NMR (400 MHz, Chloroform-*d*) δ 8.66 (d, *J* = 2.3 Hz, 1H), 8.48 (dd, *J* = 4.7, 1.7 Hz, 1H), 7.70 (app dt, *J* = 7.9, 2.1 Hz, 1H), 7.55 (app t, *J* = 8.1 Hz, 1H), 7.23 (dd, *J* = 7.9, 4.7 Hz, 1H), 7.14 (dd, *J* = 8.1, 2.0 Hz, 1H), 7.04 (dd, *J* = 9.9, 2.1 Hz, 1H), 4.66 (s, 1H), 3.72 (t, *J* = 4.7 Hz, 4H), 2.54 – 2.32 (m, 4H). $^{13}$C NMR (126 MHz, Chloroform–*d*) δ 160.4 (d,

${}^{1}J_{CF}$ = 250.0 Hz), 149.7, 149.0, 136.3, 135.6, 133.9 (d, ${}^{3}J_{CF}$ = 10.8 Hz), 129.5 (d, ${}^{3}J_{CF}$ = 4.6 Hz), 126.9 (d, ${}^{2}J_{CF}$ = 12.7 Hz), 125.1 (d, ${}^{4}J_{CF}$ = 3.6 Hz), 123.7, 116.6 (d, ${}^{2}J_{CF}$ = 25.7 Hz), 67.0, 64.8, 52.3. LRMS *m/z* (ESI) 307.2 ($[M+H]^+$, 100%). HRMS (ESI) calcd. for $[C_{16}H_{17}{}^{35}ClFN_2O]^+$ 307.1008, found 307.1012 $[M+H]^+$. IR (film): $\nu_{max}$ 2959, 2851, 1613, 1576, 1476, 1412, 1215, 1107, 1006, 898, 872, 704 $cm^{-1}$.

1-((4-Chloro-2-fluorophenyl)(pyridine-3-yl)methyl)-4-(4-methoxyphenyl)piperazine, **MYOS_00025**

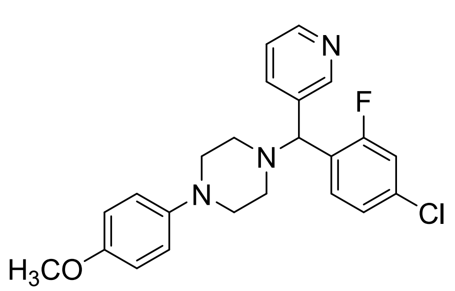


Prepared according to *General Synthetic Procedure B* to give the title compound in a 11% yield as an orange viscous oil. **IR (film)/cm$^{-1}$** 1500 (C=C), 2955 (C-H), 1036 (C-O). **$^{1}$H NMR** (400 MHz, $CDCl_3$) δ 8.57 (d, *J* = 1.8 Hz, 1H), 8.37 (dd, *J* = 4.8, 1.5 Hz, 1H), 7.65 (d, *J* = 7.9 Hz, 1H), 7.48 (t, *J* = 8.0 Hz, 1H), 7.19 – 7.13 (m, 1H), 7.06 (dd, *J* = 8.4, 1.7 Hz, 1H), 6.96 (dd, *J* = 6.4, 3.4 Hz, 1H), 6.81 – 6.72 (m, 5H), 4.65 (s, 1H), 3.67 (s, 4H), 2.60 – 2.39 (m, 5H). **$^{13}$C NMR** (101 MHz, $CDCl_3$) δ 160.36 (d, *J* = 249.8 Hz), 153.87, 149.58, 148.74, 133.79 (d, *J* = 10.5 Hz), 129.57 (d, *J* = 4.7 Hz), 125.08 (d, *J* = 3.4 Hz), 123.72, 118.07, 116.57 (d, *J* = 24.1 Hz), 114.47, 62.41 (d, *J* = 402.7 Hz), 55.55 (s), 51.19 (d, *J* = 114.8 Hz). **$^{19}$F NMR** (659 MHz, $CDCl_3$) δ -115.32 (t, *J* = 8.6 Hz). **HRMS** (ESI) *m/z* Calcd. for $C_{23}H_{24}{}^{35}ClFN_3O$ $[M+H]^+$: 412.1586; Found: 412.1583.

3-((2-Bromophenyl)(4-chlorophenyl)methyl)pyridine, **MYOS_00027**

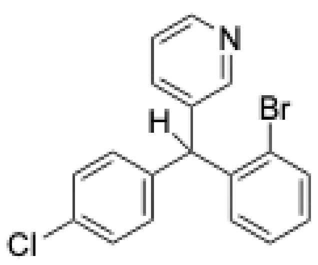


Sodium borohydride (0.53 g, 14 mmol) was cautiously added to TFA (10 mL) at 0°C. After 1.5 h at 0°C, a solution of **MYOS_00012** (81 mg, 0.25 mmol) in DCM (10 mL) was added portionwise. After 30 min at 0°C, the reaction was poured onto 25% aqueous sodium hydroxide/ice (35 mL) and extracted with diethyl ether (2 × 25 mL). The organic layers were combined, dried ($MgSO_4$) and concentrated under reduced pressure. The crude product was purified with column chromatography (ethyl acetate/hexane) to give *the title compound* as a pale straw-coloured oil (49 mg, 63%). **$^{1}$H NMR** (500 MHz, Acetone-$d_6$) δ 8.48 (dd, *J* = 4.7, 1.6 Hz, 1H), 8.39 (dt, *J* = 2.4, 0.8 Hz, 1H), 7.67 (dd, *J* = 8.0, 1.3 Hz, 1H), 7.48 – 7.42 (m, 1H), 7.41 – 7.30 (m, 4H), 7.27 – 7.21 (m, 1H), 7.18 – 7.09 (m, 2H), 7.05 – 6.53

(m, 1H), 6.02 (s, 1H). **$^{13}$C NMR** (126 MHz, Acetone-*$d_6$*) δ 151.7, 148.9, 142.7, 141.6, 138.5, 137.3, 134.2, 133.1, 132.0, 129.8, 129.5, 128.7, 125.7, 124.3, 53.8. **LRMS** *m/z* (ESI) 447 (25%), 446 (100%), 362 ($[M+H]^+$, 6%), 360 ($[M+H]^+$, 27%), 358 ($[M+H]^+$, 21%). **HRMS** (ESI) calcd. for $C_{18}H_{13}{}^{81}Br^{37}ClNNa^+$ 383.97676, $C_{18}H_{13}{}^{81}Br^{35}ClNNa^+$ 381.97971, $C_{18}H_{13}{}^{79}Br^{37}ClNNa^+$ 381.97881, $C_{18}H_{13}{}^{79}Br^{35}ClNNa^+$ 379.98176, ($[M+Na]^+$), found 383.97684, 381.97973, 379.98176. **IR** (film): $\nu_{max}$ 3057, 3029, 2923, 2853, 1701, 1574, 1489, 1465, 1421 cm$^{-1}$.

Ethyl 4-((4-chloro-2-fluorophenyl)(pyridin-3-yl)methyl)-2,2-dimethylpiperazine-1-carboxylate, **MYOS_00028**

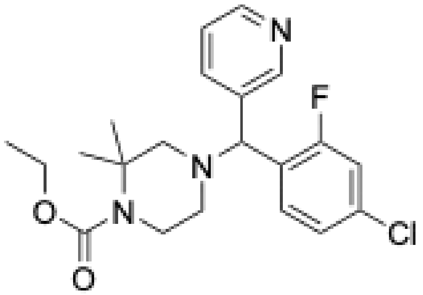


Prepared according to *General Synthetic Procedure B*, using TFA (2 mL, 27 mmol) and **Int16** (0.27 g, 0.66 mmol) in DCM (5 mL), anhydrous triethylamine (1.5 mL, 1.1 mmol), ethyl chloroformate (0.07 mL, 0.75 mmol). The crude product was purified with column chromatography (ethyl acetate/hexane) to give *the title compound* as an amber resin (40 mg, 15%). **$^1$H NMR** (600 MHz, Chloroform-*d*) δ 8.57 (m, 2H), 7.71 (dt, *J* = 8.0, 1.8 Hz, 1H), 7.54 (t, *J* = 8.0 Hz, 1H), 7.25 – 7.19 (m, 1H), 7.13 (dt, *J* = 8.4, 1.3 Hz, 1H), 7.02 (dd, *J* = 9.9, 2.1 Hz, 1H), 4.60 (s, 1H), 4.08 (q, *J* = 7.1 Hz, 2H), 3.65 – 3.24 (m, 2H), 2.78 – 2.29 (m, 2H), 2.27 – 1.92 (m, 2H), 1.39 (d, *J* = 2.1 Hz, 6H), 1.22 (t, *J* = 7.1 Hz, 3H). **$^{13}$C NMR** (151 MHz, Chloroform-*d*) δ 160.4 (d, *J* = 250.1 Hz), 157.0, 149.5, 149.0, 136.7, 135.5, 134.0 (d, *J* = 10.6 Hz), 129.3 (d, *J* = 4.6 Hz), 127.2 (d, *J* = 12.6 Hz), 125.3 (d, *J* = 3.4 Hz), 124.0, 116.7 (d, *J* = 25.9 Hz), 64.9, 64.1, 61.0, 55.2, 51.6, 42.8, 25.2, 24.8, 14.7. **LRMS** *m/z* (ESI) 430 ($[M+Na]^+$, 36%), 428 ($[M+Na]^+$, 100%), 408 ($[M+H]^+$, 7%), 406 ($[M+H]^+$, 16%). **HRMS** (ESI) calcd. for $C_{21}H_{25}{}^{37}ClFN_3O_2Na^+$ 430.14875 and $C_{21}H_{25}{}^{35}ClFN_3O_2Na^+$ 428.15170 ($[M+Na]^+$), found 430.14879, 428.15177. **IR** (film): $\nu_{max}$ 2974, 1701 (s), 1607, 1578, 1482, 1405, 1370, 1334 cm$^{-1}$.

Ethyl 4-((4-chloro-2-fluorophenyl)(pyridin-3-yl)methyl)-3-methylpiperazine-1-carboxylate, **MYOS_00029** ((*R*,*R*) and (*S*,*S*))

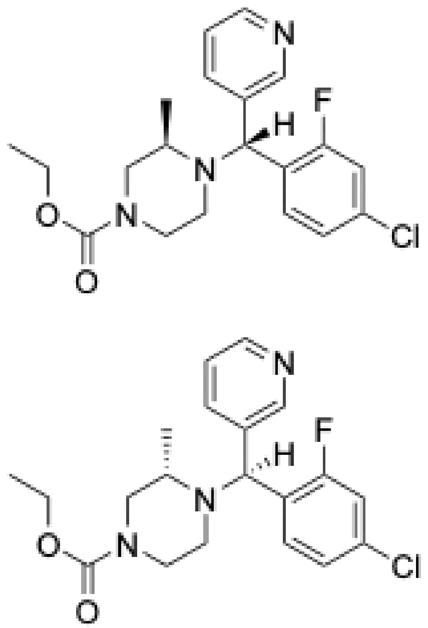

Prepared according to *General Synthetic Procedure B*, using TFA (1.5 mL, 20 mmol) and **Int17a** (52 mg, 0.12 mmol) in DCM (5 mL), anhydrous triethylamine (2 mL, 1.5 mmol), ethyl chloroformate (0.011 mL, 0.12 mmol). The crude product was purified with column chromatography (ethyl acetate/hexane) to give *the title compound* as an amber resin (11 mg, 25%). **$^{1}$H NMR** (400 MHz, Chloroform-*d*) δ 8.93 – 8.25 (m, 2H), 7.82 – 7.72 (m, 1H), 7.28 (d, *J* = 6.0 Hz, 2H), 7.17 – 6.97 (m, 2H), 5.20 (s, 1H), 4.44 – 4.00 (m, 2H), 3.84 – 3.06 (m, 3H), 3.01 – 1.83 (m, 4H), 1.23 (t, *J* = 7.1 Hz, 3H), 1.09 – 0.67 (m, 3H). **$^{13}$C NMR** (101 MHz, Chloroform-*d*) δ 160.5 (d, *J* = 250.1 Hz), 155.9, 153.9, 149.2, 148.5, 135.9, 130.8 (d, *J* = 4.8 Hz), 125.2, 124.0, 117.0 (d, *J* = 26.5 Hz), 61.5, 59.2, 50.3, 49.8, 44.4, 43.9, 14.8, 10.5 (2 obscured signals). **LRMS** *m/z* (ESI) 416 ([M+Na]$^+$, 41%), 414 ([M+Na]$^+$, 100%), 394 ([M+H]$^+$, 6%), 392 ([M+H]$^+$, 19%). **HRMS** (ESI) calcd. for $C_{20}H_{23}{}^{37}ClFN_3O_2Na^+$ 416.13310 and $C_{20}H_{23}{}^{35}ClFN_3O_2Na^+$ 414.13605 ([M+Na]$^+$), found 416.13322, 414.13608. **IR** (film): $\nu_{max}$ 2976, 1693 (s), 1606, 1577, 1481, 1429, 1382 cm$^{-1}$.

Ethyl 4-((4-chloro-2-fluorophenyl)(pyridin-3-yl)methyl)-3-methylpiperazine-1-carboxylate, **MYOS_00030** ((*S*,*R*) and (*R*,*S*))

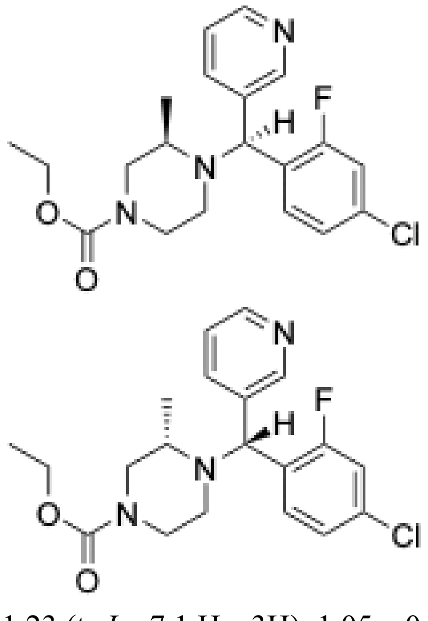

Prepared according to *General Synthetic Procedure B*, using TFA (1.5 mL, 20 mmol) and **Int17b** (56 mg, 0.13 mmol) in DCM (5 mL), anhydrous triethylamine (2 mL, 1.5 mmol), ethyl chloroformate (0.013 mL, 0.13 mmol). The crude product was purified with column chromatography (ethyl acetate/hexane) to give *the title compound* as an amber resin (17 mg, 34%). **$^{1}$H NMR** (400 MHz, Chloroform-*d*) δ 8.57 (m, 2H), 7.69 (m, 2H), 7.25 (dd, *J* = 7.6, 5.1 Hz, 1H), 7.15 (dd, *J* = 8.5, 2.1 Hz, 1H), 7.00 (dd, *J* = 10.0, 2.1 Hz, 1H), 4.98 (s, 1H), 4.12 (qq, *J* = 7.0, 3.6 Hz, 1H), 3.85 (m, 2H), 3.39 – 2.78 (m, 2H), 2.68 – 1.74 (m, 3H), 1.23 (t, *J* = 7.1 Hz, 3H), 1.05 – 0.89 (m, 3H). **$^{13}$C NMR** (101 MHz, Chloroform-*d*) δ 160.5 (d, *J* = 250.0 Hz), 156.1, 153.9, 149.7, 148.9, 136.1, 129.1, 125.4, 123.9, 116.7 (d, *J* = 25.8 Hz),

61.5, 60.2, 49.6, 43.8, 43.5, 14.8, 8.6 (3 obscured signals). **LRMS** *m/z* (ESI) 416 ([M+Na]$^+$, 38%), 414 ([M+Na]$^+$, 100%), 394 ([M+H]$^+$, 6%), 392 ([M+H]$^+$, 15%). **HRMS** (ESI) calcd. for $C_{20}H_{23}{}^{37}ClFN_3O_2Na^+$ 416.13310 and $C_{20}H_{23}{}^{35}ClFN_3O_2Na^+$ 414.13605 ([M+Na]$^+$), found 416.13320, 414.13611. **IR** (film): $\nu_{max}$ 2976, 1693 (s), 1606, 1578, 1480, 1427, 1382 cm$^{-1}$.

Benzyl 4-((4-chloro-2-fluorophenyl)(pyridin-3-yl)methyl)piperazine-1-carboxylate, **MYOS_00031**

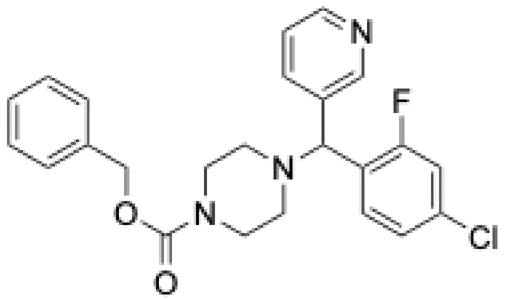

Prepared according to *General Synthetic Procedure B*, using TFA (0.9 mL, 12 mmol) and **Int15** (0.36 g, 0.9 mmol) in DCM (5 mL), anhydrous triethylamine (2.5 mL, 18 mmol), benzyl chloroformate (0.11 mL 0.78 mmol). The crude product was purified with column chromatography (ethyl acetate/hexane) to give *the title compound* as an amber resin (0.22 g, 63%). **$^1$H NMR** (600 MHz, Chloroform-*d*) δ 8.56 (s, 1H), 8.46 – 8.17 (m, 1H), 7.60 (d, *J* = 8.0 Hz, 1H), 7.41 (t, *J* = 8.0 Hz, 1H), 7.28 – 7.17 (m, 4H), 7.13 (dd, *J* = 7.9, 4.7 Hz, 1H), 7.04 (dd, *J* = 8.5, 2.0 Hz, 1H), 6.93 (dd, *J* = 9.9, 2.1 Hz, 1H), 5.03 (s, 2H), 4.61 (s, 1H), 3.73 – 3.01 (m, 4H), 2.67 – 1.93 (m, 4H). **$^{13}$C NMR** (151 MHz, Chloroform-*d*) δ 160.3 (d, *J* = 250.1 Hz), 155.1, 149.5, 148.9, 136.6, 136.3, 135.5, 133.9 (d, *J* = 10.5 Hz), 129.5 (d, *J* = 4.5 Hz), 128.5, 128.0, 127.8, 126.6 (d, *J* = 12.5 Hz), 125.1 (d, *J* = 3.5 Hz), 123.7, 116.6 (d, *J* = 26.0 Hz), 67.1, 64.3 (d, *J* = 1.6 Hz), 51.3, 43.8. **LRMS** *m/z* (ESI) 464 ([M+Na]$^+$, 39%), 462 ([M+Na]$^+$, 100%), 442 ([M+H]$^+$, 12%), 440 ([M+H]$^+$, 28%). **HRMS** (ESI) calcd. for $C_{24}H_{23}{}^{37}ClFN_3O_2Na^+$ 464.13310 and $C_{24}H_{23}{}^{35}ClFN_3O_2Na^+$ 462.13605 ([M+Na]$^+$), found 464.13334, 462.13624. **IR** (film): $\nu_{max}$ 2817, 1695 (s), 1607, 1577, 1481, 1426 cm$^{-1}$.

(3-(4-((4-Chloro-2-fluorophenyl)(pyridin-3-yl)methyl)piperazin-1-yl)propan-1-ol, **MYOS_00033**

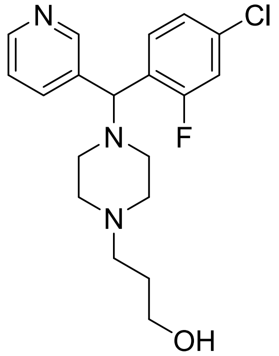

Prepared according to *General Synthetic Procedure B* to give the title compound in a 39% yield as a white viscous oil. **IR (film)/cm$^{-1}$** 3306

(C-OH), 2929 (C-H), 1427 (C-F). **$^1$H NMR** (400 MHz, $CDCl_3$) δ 8.56 (d, *J* = 1.9 Hz, 1H), 8.39 (dd, *J* = 4.8, 1.5 Hz, 1H), 7.59 (d, *J* = 7.9 Hz, 1H), 7.42 (t, *J* = 8.1 Hz, 1H), 7.13 (dt, *J* = 10.1, 5.0 Hz, 1H), 7.04 (dd, *J* = 8.4, 1.7 Hz, 1H), 6.95 (dd, *J* = 9.9, 2.0 Hz, 1H), 4.60 (s, 1H), 3.71 – 3.68 (m, 2H), 2.58 – 2.29 (m, 10H), 1.62 (dt, *J* = 10.8, 5.4 Hz, 2H), 1.19 (d, *J* = 14.4 Hz, 1H). **$^{13}$C NMR** (101 MHz, $CDCl_3$) δ 161.70 – 161.48 (m), 159.35 – 159.30 (m), 149.64, 148.82, 136.03 (d, *J* = 103.0 Hz), 133.91 – 133.60 (m), 129.50 (d, *J* = 4.6 Hz), 127.24 – 126.80 (m), 125.03, 123.60, 116.58 (d, *J* = 26.1 Hz), 64.53, 64.36, 58.64, 53.34, 51.56, 27.05. **$^{19}$F NMR** (659 MHz, $CDCl_3$) δ -115.33 (t, *J* = 8.6 Hz). **HRMS** (ESI) *m/z* Calcd. for $C_{19}H_{24}{}^{35}ClFN_3O$ $[M+H]^+$: 364.1586; Found: 364.1583.

1-((4-Chloro-2-fluorophenyl)(pyridin-3-yl)methyl)-4-(pyridin-4-yl)piperzine, **MYOS_00034**

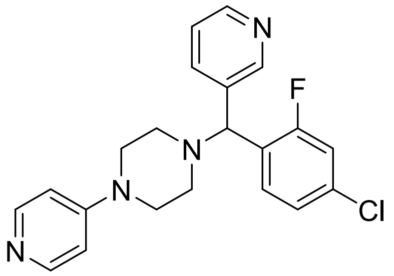


Prepared according to *General Synthetic Procedure B* to give the title compound in a 23% yield as a white viscous oil. **IR (film)/cm$^{-1}$** 1595 (C=C), 1248 (C-N). **$^1$H NMR** (500 MHz, $CDCl_3$) δ 8.68 (d, *J* = 1.9 Hz, 1H), 8.51 (dd, *J* = 4.8, 1.6 Hz, 1H), 8.26 (s, 2H), 7.73 (dt, *J* = 7.9, 1.8 Hz, 1H), 7.56 (t, *J* = 8.0 Hz, 1H), 7.16 (dd, *J* = 8.4, 1.8 Hz, 1H), 7.06 (dd, *J* = 9.9, 2.1 Hz, 1H), 6.63 (d, *J* = 6.0 Hz, 2H), 4.73 (s, 1H), 2.61 – 2.49 (m, 4H). **$^{13}$C NMR** (126 MHz, $CDCl_3$) δ 161.3, 159.35, 154.92, 149.90, 149.62, 149.09, 135.86 (d, *J* = 94.3 Hz), 134.06 (d, *J* = 10.6 Hz), 129.36 (d, *J* = 4.6 Hz), 126.74 (d, *J* = 12.7 Hz), 125.22 (d, *J* = 3.5 Hz), 123.78, 116.69 (d, *J* = 25.8 Hz), 108.31, 64.33, 48.55 (d, *J* = 650.1 Hz). **$^{19}$F NMR** (659 MHz, $CDCl_3$) δ -115.37 (t, *J* = 8.7 Hz). **HRMS** (ESI) *m/z* Calcd. for $C_{21}H_{21}{}^{35}ClFN_4$ $[M+H]^+$: 383.1433; Found: 383.1448.

1-4((4-Chloro-2-fluorophenyl)(pyridine-3-yl)methyl)-4-(pyridine-2-yl)piperazine, **MYOS_00035**

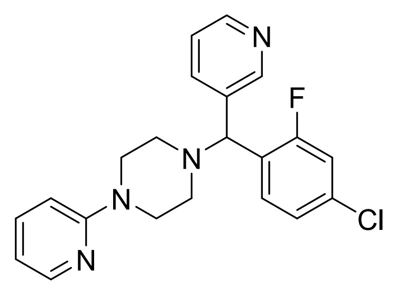


Prepared according to *General Synthetic Procedure B* to give the title compound in a 61% yield as an orange viscous oil. **IR (film)/cm$^{-1}$** 1595 (C=C), 1244 (C-N). **$^1$H NMR** (500 MHz, $CDCl_3$) δ 8.68 (d, *J* = 2.0 Hz, 1H), 8.49 (dd, *J* = 4.8, 1.6 Hz,

1H), 8.18 (ddd, $J = 4.9, 1.9, 0.8$ Hz, 1H), 7.73 (dt, $J = 7.9, 1.9$ Hz, 1H), 7.59 (t, $J = 8.1$ Hz, 1H), 7.49 – 7.45 (m, 1H), 7.25 – 7.23 (m, 1H), 4.72 (s, 1H), 2.59 – 2.44 (m, 4H). **$^{13}$C NMR** (126 MHz, $CDCl_3$) δ 161.34, 159.40 (d, $J = 11.5$ Hz), 149.71, 148.94, 147.98, 137.02 (d, $J = 117.8$ Hz), 136.03 (d, $J = 130.2$ Hz), 127.11 (d, $J = 12.5$ Hz), 125.12 (d, $J = 3.3$ Hz), 123.70, 116.58 (d, $J = 25.9$ Hz), 114.03 – 106.48 (m), 64.44, 51.51. **$^{19}$F NMR** (659 MHz, $CDCl_3$) δ -115.47 (t, $J = 8.7$ Hz). **HRMS** (ESI) *m/z* Calcd. for $C_{21}H_{21}{}^{35}ClFN_4$ $[M+H]^+$: 383.1433; Found: 383.1430.

1-(4-Bromophenyl)-1-(pyridin-3-yl)pentan-1-ol, **MYOS_00130**

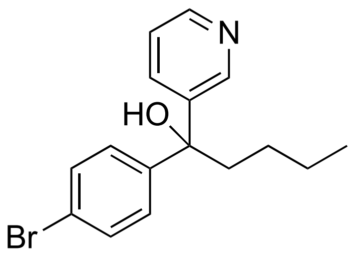


Prepared according to *General Synthetic Procedure A* using intermediate **Int8** (0.20 g, 0.75 mmol). The crude mixture was purified by column chromatography (hexane/ethyl acetate) to give *the title compound* as a transparent, straw-coloured gel (0.48 g, 65%). $^1$H NMR (500 MHz, Acetone-$d_6$) $\delta$ 8.68 (d, $J = 1.8$ Hz, 1H), 8.37 (dd, $J = 4.7, 1.6$ Hz, 1H), 7.85 (ddd, $J = 8.0, 2.4, 1.6$ Hz, 1H), 7.54 – 7.43 (m, 4H), 7.27 (ddd, $J = 8.1, 4.7, 0.9$ Hz, 1H), 5.06 (s, 1H), 2.38 – 2.32 (m, 2H), 1.39 – 1.24 (m, 4H), 0.85 (t, $J = 7.0$ Hz, 3H); $^{13}$C NMR (126 MHz, Acetone-$d_6$) $\delta$ 148.6, 148.4, 148.0, 144.0, 134.3, 131.8, 129.0, 123.7, 120.9, 76.7, 41.8, 26.5, 23.6, 14.3; LRMS *m/z* (ESI) 322 ($[M+H]^+$, 99%), 320 ($[M+H]^+$, 98%); HRMS (ESI) calcd for $[C_{16}H_{19}{}^{81}BrNO]^+$ 322.06241 and $[C_{16}H_{19}{}^{79}BrNO]^+$ 320.06445 ($[M+H]^+$), found 322.06269 and 320.06475; IR (film) $\nu_{max}$ 3165 (br), 1485, 1141, 1072, 739 and 666 cm$^{-1}$.

(4-Bromophenyl)(cyclohexyl)(pyridine-3-yl)methanol, **MYOS_00131**

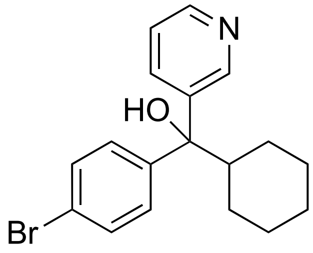


Prepared according to *General Synthetic Procedure C*, using cyclohexyl magnesium bromide (1 M in THF, 1.5 mL, 1.5 mmol) and intermediate **Int8** (0.25 g, 1.0 mmol). The crude product was purified by recrystallization in ethyl acetate to give *the title compound* as an opaque lustrous white crystalline solid (0.21 g,

64%). m.p. 109–111°C (no lit. m.p.) $^{1}$H NMR (400 MHz, Chloroform-*d*) $\delta$ 8.67 (d, $J$ = 2.4 Hz, 1H), 8.40 (dd, $J$ = 4.8, 1.6 Hz, 1H), 7.76 (dt, $J$ = 8.1, 2.0 Hz, 1H), 7.45 – 7.39 (m, 2H), 7.36 – 7.31 (m, 2H), 7.21 (dd, $J$ = 8.0, 4.8 Hz, 1H), 2.48 (s, 1H), 2.38 (tt, $J$ = 11.8, 2.9 Hz, 1H), 1.82 – 1.63 (m, 3H), 1.60 – 1.46 (m, 2H), 1.39 – 1.21 (m, 2H), 1.19 – 0.81 (m, 3H); $^{13}$C NMR (101 MHz, Chloroform-*d*) $\delta$ 147.8, 147.6, 144.8, 141.5, 133.9, 131.6, 127.8, 123.3, 121.0, 79.3, 45.6, 27.1, 26.6, 26.4; LRMS *m/z* (ESI) 348 ([M+H]$^+$, 96%) and 346 ([M+H]$^+$, 100%); 346; HRMS (ESI) calcd. for $[C_{18}H_{20}{}^{81}BrNONa]^+$ 370.06000 and $[C_{18}H_{20}{}^{79}BrNONa]^+$ 368.06205 ([M+Na]$^+$), found 370.06005 and 368.06207; IR (film) $\nu_{max}$ 3138 (br), 2931, 1480, 1073, 1007, 806 and 711 cm$^{-1}$.

9-(Pyridin-3-yl)-9H-fluoren-9-ol, **MYOS_00132**

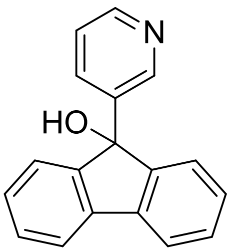


Prepared according to *General Synthetic Procedure A* using 3-bromopyridine (2.0 mL, 20. mmol) and 9*H*-fluoren-9-one (1.8 g, 10 mmol). The crude mixture was purified by recrystallization in ethyl acetate to give *the title compound* as a white lustrous, transparent, crystalline solid (1.5 g, 58 %). m.p. 147–148°C (no lit. m.p.). $^{1}$H NMR (400 MHz, Acetone-$d_6$) $\delta$ 8.52 (dd, $J$ = 2.3, 0.9 Hz, 1H), 8.40 (dd, $J$ = 4.7, 1.6 Hz, 1H), 7.80 (app dt, $J$ = 7.6, 0.9 Hz, 2H), 7.68 (ddd, $J$ = 8.0, 2.4, 1.7 Hz, 1H), 7.40 (app td, $J$ = 7.3, 1.6 Hz, 2H), 7.35 – 7.19 (m, 5H), 5.44 (s, 1H); $^{13}$C NMR (101 MHz, Acetone-$d_6$) $\delta$ 151.6, 149.0, 148.1, 141.3, 140.5, 133.7, 129.9, 129.2, 125.6, 123.8, 121.0, 82.8; LRMS *m/z* (ESI) 282 ([M+Na]$^+$, 39%), 260 ([M+H]$^+$, 100%); HRMS (ESI) calcd. for $[C_{18}H_{13}NONa]^+$ 282.08893 ([M+Na]$^+$), found 282.08877; IR (film): $\nu_{max}$ 3140 (br), 3056, 1448, 1358, 1178, 1057, 928 and 731 cm$^{-1}$.

1-Phenyl-1-(pyridine-3-yl)butan-1-ol, **MYOS_00133**

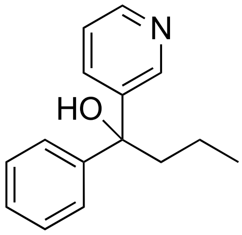


Prepared according to *General Synthetic Procedure A* using 3-bromopyridine (2.0 mL, 20 mmol) and 1-phenylbutan-1-one (1.5 g, 10. mmol). The crude mixture was purified by recrystallization in ethyl acetate to give *the title compound* as a straw-coloured semi-lustrous translucent coarsely crystalline solid (1.3 g, 56%). m.p. 83–85°C (no lit. m.p.). $^{1}$H NMR (500 MHz, Acetone-$d_6$) $\delta$ 8.68 (dd, $J$ = 2.4, 0.9 Hz, 1H), 8.35 (dd, $J$ = 4.7, 1.6

Hz, 1H), 7.85 (ddd, $J$ = 8.0, 2.3, 1.6 Hz, 1H), 7.56 – 7.49 (m, 2H), 7.34 – 7.27 (m, 2H), 7.25 (ddd, $J$ = 8.0, 4.7, 0.9 Hz, 1H), 7.23 – 7.15 (m, 1H), 4.94 (s, 1H), 2.33 (dd, $J$ = 8.9, 7.3 Hz, 2H), 1.55 – 1.20 (m, 2H), 0.91 (t, $J$ = 7.4 Hz, 3H); $^{13}$C NMR (126 MHz, Acetone-$d_6$) $\delta$ 148.7, 148.5, 148.2, 144.6, 134.3, 128.8, 127.3, 126.8, 123.6, 77.0, 44.6, 17.6, 14.65; LRMS *m/z* (ESI) 228 ([M+H]$^+$, 100%); HRMS (ESI) calcd. for [$C_{15}H_{17}NONa$]$^+$ 250.12023 ([M+Na]$^+$), found 250.12027; IR (film) $\nu_{max}$ 3123 (br), 2960, 1595, 1419, 1134, 771, 711 and 698 cm$^{-1}$.

(4-Bromophenyl)(4-chlorophenyl)methanone, **MYOS_00134**

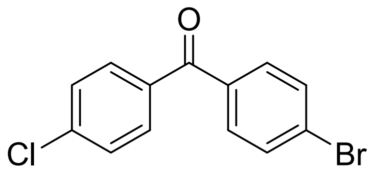


Prepared according to *General Synthetic Procedure F,* using 4-bromobenzoyl chloride (2.9 mL, 23 mmol). The crude mixture was recrystallized in ethyl acetate to give *the title compound* as golden lustrous opaque long needles (5.1 g, 76%). $^1$H NMR (500 MHz, Acetone-$d_6$) $\delta$ 7.86 – 7.78 (m, 2H), 7.79 – 7.69 (m, 4H), 7.64 – 7.56 (m, 2H); $^{13}$C NMR (126 MHz, Acetone-$d_6$) $\delta$ 194.5, 139.3, 137.2, 136.7, 132.6, 132.4, 132.3, 129.6, 127.9; LRMS *m/z* (ESI) 321([M+Na]$^+$, 25%), 319 ([M+Na]$^+$, 100%), 317 ([M+Na]$^+$, 73%); HRMS (ESI) calcd. for [$C_{13}H_8{}^{81}Br^{37}ClONa$]$^+$ 320.92893, [$C_{13}H_8{}^{79}Br^{37}ClONa$]$^+$ and [$C_{13}H_8{}^{81}Br^{35}ClONa$]$^+$ 318.93188, [$C_{13}H_8{}^{79}Br^{35}ClONa$]$^+$ 316.93393 ([M+Na]$^+$), found 320.92970, 318.93185, 316.93414; IR (film) $\nu_{max}$ 1644 (s), 1581, 1281, 852, 748 and 665 cm$^{-1}$. Spectroscopic data matched those in the literature.[5]

*N*-((4-Chloro-2-fluorophenyl)(pyridin-3-yl)methyl)-2-methoxy-*N*-(2-methoxyethyl)ethan-1-amine, **MYOS_00135**

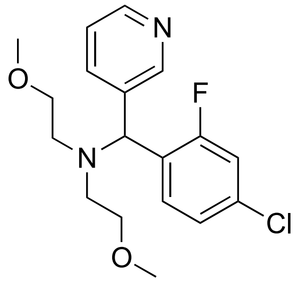


Prepared according to *General Synthetic Procedure B* using **Int2** (300 mg, 1.2 mmol), *bis*(2-methoxyethyl)amine (0.26 mL, 1.8 mmol), anhydrous $Et_3N$ (0.33 mL, 2.3 mmol) and KI (catalytic amount) to give the crude product (318 mg) which was purified by column chromatography (40–70%, EtOAc in hexanes) to afford *the title*

*compound* as an orange oil (151 mg, 37%). $^{1}$H NMR (500 MHz, Chloroform-*d*) δ 8.62 (d, *J* = 2.2 Hz, 1H), 8.48 (dd, *J* = 4.7, 1.7 Hz, 1H), 7.70 (dt, *J* = 7.9, 2.0 Hz, 1H), 7.46 (app t, *J* = 8.1 Hz, 1H), 7.25 – 7.18 (m, 1H), 7.13 (dt, *J* = 8.5, 1.4 Hz, 1H), 7.05 (dd, *J* = 9.9, 2.1 Hz, 1H), 5.34 (s, 1H), 3.63 – 3.33 (m, 4H), 3.29 (s, 6H), 2.96 – 2.51 (m, 4H). $^{13}$C NMR (126 MHz, Chloroform–*d*) δ 160.6 (d, $^{1}J_{CF}$ = 250.1 Hz), 149.9, 148.6, 137.4, 136.1, 133.9 (d, $^{3}J_{CF}$ = 10.8 Hz), 130.5 (d, $^{3}J_{CF}$ = 4.7 Hz), 127.1 (d, $^{2}J_{CF}$ = 13.3 Hz), 124.9 (d, $^{4}J_{CF}$ = 3.6 Hz), 123.4, 116.6 (d, $^{2}J_{CF}$ = 26.5 Hz), 71.2, 61.3, 59.0, 50.7. LRMS *m/z* (ESI) 353.2 ([M+H]$^{+}$, 100%). HRMS (ESI) calcd. For $[C_{18}H_{23}{}^{35}ClFN_2O_2]^{+}$ 353.1427, found 353.1429 [M+H]$^{+}$. IR (film): $\nu_{max}$ 2929, 1621, 1569, 1476, 1408, 1114, 1021, 801, 708 cm$^{-1}$.

2-((4-Chloro-2-fluorophenyl)(pyridin-3-yl)methyl)-1,2,3,4-tetrahydroisoquinoline, **MYOS_00136**

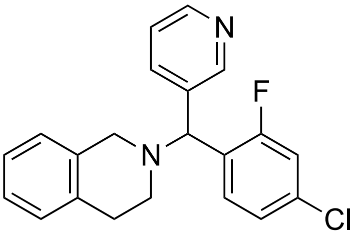


Prepared according to *General Synthetic Procedure B* using **Int2** (200 mg, 781 μmol), 1,2,3,4-tetrahydroisoquinoline (0.15 mL, 1.2 mmol), anhydrous $Et_3N$ (0.22 mL, 1.6 mmol) and KI (catalytic amount) to give the crude product (233 mg) which was purified by Biotage Selekt chromatography (40–70%, EtOAc in hexanes) to afford *the title compound* as a waxy yellow solid (115 mg, 42%). $^{1}$H NMR (400 MHz, Chloroform-*d*) δ 8.70 (d, *J* = 2.3 Hz, 1H), 8.51 (dd, *J* = 4.8, 1.7 Hz, 1H), 7.76 (app dt, *J* = 7.9, 1.9 Hz, 1H), 7.63 (app t, *J* = 8.1 Hz, 1H), 7.27 – 7.21 (m, 1H), 7.18 – 7.07 (m, 4H), 7.06 (dd, *J* = 9.9, 2.1 Hz, 1H), 6.90 (d, *J* = 7.5 Hz, 1H), 4.87 (s, 1H), 3.58 (s, 2H), 2.96 – 2.85 (m, 2H), 2.84 – 2.64 (m, 2H). $^{13}$C NMR (126 MHz, Chloroform–*d*) δ 160.4 (d, $^{1}J_{CF}$ = 250.0 Hz), 149.7, 149.1, 137.1, 135.6, 134.5 (d, *J* = 16.4 Hz), 133.9 (d, $^{3}J_{CF}$ = 10.4 Hz), 129.5 (d, $^{3}J_{CF}$ = 4.7 Hz), 128.8, 127.7 (d, $^{2}J_{CF}$ = 12.7 Hz), 126.9, 126.5, 125.9, 125.3 (d, $^{4}J_{CF}$ = 3.5 Hz), 123.9, 116.7 (d, $^{2}J_{CF}$ = 25.8 Hz), 64.2, 55.0, 49.3, 29.3. LRMS *m/z* (ESI+) 353.3 ([M+H]$^{+}$, 100%). HRMS (ESI+) calcd. for $[C_{21}H_{19}{}^{35}ClFN_2]^{+}$ 353.1215, found 353.1224 [M+H]$^{+}$. IR (film): $\nu_{max}$ 3026, 2922, 1736, 1576, 1479, 1408, 1237, 1129, 1080, 898, 790, 745, 711 cm$^{-1}$.

3-((4-Chloro-2-fluorophenyl)(4,4-difluoropiperidin-1-yl)methyl)pyridine, **MYOS_00137**

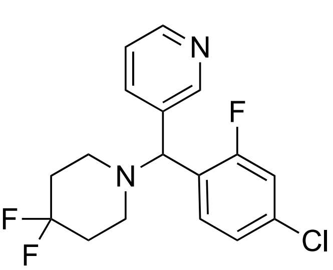

Prepared according to *General Synthetic Procedure B* using **Int2** (100 mg, 390 µmol), 4,4-difluoropiperidine hydrochloride (94 mg, 586 µmol), anhydrous $Et_3N$ (0.11 mL, 781 µmol) and KI (catalytic amount) to give the crude product (37 mg) which was purified by Biotage Selekt chromatography (5–75%, EtOAc in hexanes) to afford *the title compound* as a white solid (21 mg, 16%). $^1H$ NMR (500 MHz, Chloroform-*d*) δ 8.58 (d, *J* = 2.3 Hz, 1H), 8.43 (dd, *J* = 4.7, 1.7 Hz, 1H), 7.62 (app dt, *J* = 8.0, 2.0 Hz, 1H), 7.42 (app t, *J* = 8.1 Hz, 1H), 7.19 – 7.11 (m, 1H), 7.08 (dd, *J* = 8.9, 1.9 Hz, 1H), 6.98 (dd, *J* = 9.9, 2.1 Hz, 1H), 4.73 (s, 1H), 2.55 – 2.35 (m, 4H), 2.04 – 1.81 (m, 4H). $^{13}C$ NMR (126 MHz, Methanol–$d_4$) δ 162.7 (d, $^1J_{CF}$ = 251.7 Hz), 149.7, 149.4, 138.9, 137.7, 135.3 (d, $^3J_{CF}$ = 10.4 Hz), 131.1 (d, $^3J_{CF}$ = 4.7 Hz), 128.5 (d, $^2J_{CF}$ = 12.9 Hz), 126.4 (d, $^4J_{CF}$ = 3.6 Hz), 125.6, 117.5 (d, $^2J_{CF}$ = 26.3 Hz), 115.4, 74.6, 64.9, 35.1 (t, *J* = 23.2 Hz). LRMS *m/z* (ESI+) 341.2 ($[M+H]^+$, 100%). HRMS (ESI+) calcd. for $[C_{17}H_{17}{}^{35}ClF_3N_2]^+$ 341.1027, found 341.1024 $[M+H]^+$. IR (film): $\nu_{max}$ 2970, 2832, 1576, 1464, 1360, 1230, 1080, 1021, 950, 898, 708 $cm^{-1}$.

1-((4-Chloro-2-fluorophenyl)(pyridin-3-yl)methyl)piperidin-4-one, **MYOS_00139**

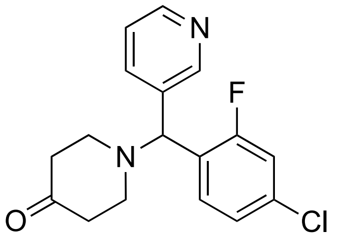


Prepared according to *General Synthetic Procedure B* using **Int2** (308 mg, 1.2 mmol), piperidin-4-one (179 mg, 1.8 mmol), anhydrous $Et_3N$ (0.34 mL, 2.4 mmol) and KI (10 mg, 60 µmol). Piperidin-4-one reagent was prepared by treating 4-piperidone monohydrate hydrochloride with 1 M NaOH (aq) to pH 10. The aqueous solution was treated with EtOAc, layers separated, organic phases combined and concentrated under reduced pressure to afford the pure substrate for the reaction. The reaction mixture was heated under reflux at 85ºC for 48 h. The crude product (442 mg) was purified by Biotage Selekt chromatography (20–85%, EtOAc in hexanes) to give a semi-pure material which was repurified by reverse phase chromatography (Biotage Sfar C18 30 g column, 0–100%, MeOH in $H_2O$) to afford *the title compound* as a viscous light-yellow liquid (143 mg, 37%). $^1H$ NMR (400 MHz, Chloroform–*d*) δ 8.71 (d, *J* = 2.3 Hz, 1H), 8.51 (dd, *J* = 4.8, 1.6 Hz, 1H), 7.74 (app dt, *J* = 7.9 Hz, 1H), 7.57 (app t, 1H, *J* = 8.05 Hz), 7.28 (s, 1H), 7.20 – 7.12 (m, 1H), 7.07 (dd, *J* = 9.9, 2.1 Hz, 1H), 4.89 (s, 1H), 2.87 – 2.57 (m, 4H), 2.62 – 2.34 (m, 4H). $^{13}C$ NMR (126 MHz, Chloroform–*d*) δ 208.3 (C=O), 160.3 (d, $^1J_{CF}$ = 250.2 Hz), 148.8, 148.6, 137.0, 135.9, 134.5 (d, $^3J_{CF}$ = 10.4 Hz), 129.4 (d, $^3J_{CF}$ = 4.5 Hz), 126.6 (d, $^2J_{CF}$ = 12.1 Hz), 125.5 (d, $^4J_{CF}$ = 3.5 Hz), 124.1, 116.9 (d, $^2J_{CF}$ = 25.7 Hz), 63.6, 51.4, 41.2. LRMS *m/z* (ESI+) 319.2 ($[M+H]^+$,

100%). HRMS (ESI+) calcd. for $[C_{17}H_{17}{}^{35}ClFN_2O]^+$ 319.1008, found 319.1020 $[M+H]^+$. IR (film): $\nu_{max}$ 2967, 2810, 1714, 1576, 1479, 1211, 1077, 898, 711 $cm^{-1}$.

2-(4-((4-Chloro-2-fluorophenyl)(pyridin-3-yl)methyl)piperazin-1-yl)-*N,N*-dimethylethan-1-amine, **MYOS_00140**

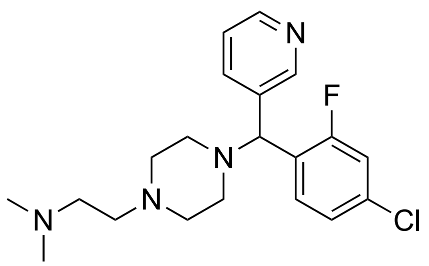

Prepared according to *General Synthetic Procedure B* using **Int2** (248 mg, 969 µmol), *N,N*-dimethyl-2-(piperazin-1-yl)ethan-1-amine (0.25 mL, 1.5 mmol), anhydrous $Et_3N$ (0.27 mL, 1.9 mmol) and KI (catalytic amount). The reaction mixture was heated under reflux at 85°C for 48 h to give the crude product (192 mg) which was purified by Biotage Selekt reverse phase chromatography (Biotage Sfar C18 30 g column, 0–100%, MeOH in $H_2O$) to afford *the title compound* as a viscous light-orange liquid (67 mg, 18%). $^1$H NMR (500 MHz, Chloroform–*d*) δ 8.62 (d, *J* = 2.2 Hz, 1H), 8.46 (dd, *J* = 4.8, 1.6 Hz, 1H), 7.69 (app dt, *J* = 7.9 Hz, 1H), 7.51 (app t, *J* = 8.1 Hz, 1H), 7.25 – 7.18 (m, 1H), 7.12 (dd, *J* = 8.6, 2.1 Hz, 1H), 7.02 (dd, *J* = 9.8, 2.1 Hz, 1H), 4.67 (s, 1H), 3.53 – 3.41 (m, 8H), 2.51 – 2.38 (m, 4H), 2.23 (s, 6H). $^{13}$C NMR (126 MHz, Methanol–$d_4$) δ 161.8 (d, $^1J_{CF}$ = 249.2 Hz), 149.9, 149.3, 138.8, 137.9, 135.2 (d, $^3J_{CF}$ = 10.8 Hz), 131.2 (d, $^3J_{CF}$ = 4.6 Hz), 128.4 (d, $^2J_{CF}$ = 12.3 Hz), 126.3 (d, $^4J_{CF}$ = 3.5 Hz), 125.5, 117.4 (d, $^2J_{CF}$ = 26.5 Hz), 65.8, 57.1, 56.8, 54.6, 52.3, 45.8. LRMS *m/z* (ESI+) 377.1 ($[M+H]^+$, 100%). HRMS (ESI+) calcd. for $[C_{20}H_{27}{}^{35}ClFN_4]^+$ 377.1903, found 377.1907 $[M+H]^+$. IR (film): $\nu_{max}$ 2944, 2810, 1606, 1576, 1479, 1334, 1285, 1133, 1006, 898, 846, 711 $cm^{-1}$.

3-((4-Chloro-2-fluorophenyl)(1H-pyrazol-1-yl)methyl)pyridine, **MYOS_00141**

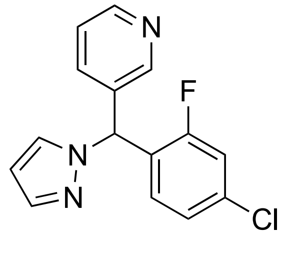

Prepared according to *General Synthetic Procedure B* using **Int2** (307 mg, 1.2 mmol), pyrazole (123 mg, 1.8 mmol), anhydrous $Et_3N$ (0.34 mL, 2.4 mmol) and KI (10 mg, 60 µmol). The reaction mixture was heated under reflux at 85°C for 48 h to give the crude product (358 mg) which was purified by Biotage Selekt reverse phase chromatography (Biotage Sfar C18 30 g column, 0–91%, MeOH in $H_2O$) to afford *the title*

*compound* as a light-yellow liquid (14 mg, 4%). $^{1}$H NMR (400 MHz, Chloroform-*d*) δ 8.62 (d, *J* = 4.1 Hz, 1H), 8.44 (d, *J* = 2.4 Hz, 1H), 7.65 (d, *J* = 1.8 Hz, 1H), 7.44 (app dt, obscured peak, 1H), 7.40 (d, *J* = 2.4 Hz, 1H), 7.32 (dd, *J* = 7.9, 4.8 Hz, 1H), 7.21 – 7.15 (m, 1H), 7.18 – 7.12 (m, 1H), 6.99 (s, 1H), 6.95 (app t, *J* = 8.3 Hz, 1H), 6.36 (app t, *J* = 2.1 Hz, 1H). $^{13}$C NMR (126 MHz, Chloroform–*d*) δ 159.8 (d, $^{1}J_{CF}$ = 252.1 Hz), 148.0, 147.8, 141.0, 137.4, 136.1 (d, $^{3}J_{CF}$ = 10.1 Hz), 135.2, 130.4 (d, $^{3}J_{CF}$ = 3.7 Hz), 130.0, 125.4 (d, $^{4}J_{CF}$ = 3.7 Hz), 124.6 (d, $^{2}J_{CF}$ = 13.6 Hz), 124.4, 116.9 (d, $^{2}J_{CF}$ = 24.7 Hz), 106.6, 60.3 (d, *J* = 3.6 Hz). LRMS *m/z* (ESI+) 288.0 ([M+H]$^{+}$, 100%). HRMS (ESI+) calcd. for $[C_{15}H_{12}{}^{35}ClFN_3]^+$ 288.0698, found 288.0703 [M+H]$^{+}$. IR (film): $\nu_{max}$ 2926, 1610, 1580, 1483, 1408, 1289, 1230, 1047, 965, 902, 752, 711 cm$^{-1}$.

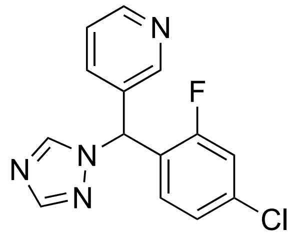


3-((4-Chloro-2-fluorophenyl)(1H-1,2,4-triazol-1-yl)methyl)pyridine, **MYOS_00142**

Prepared according to *General Synthetic Procedure B* using **Int2** (200 mg, 781 μmol), 1,2,4-triazole (81 mg, 1.2 mmol), anhydrous $Et_3N$ (0.22 mL, 1.6 mmol) and KI (7.0 mg, 39 μmol). The reaction mixture was heated under reflux at 85°C for 48 h to give the crude product (241 mg) which was purified by Biotage Selekt reverse phase chromatography (MeOH in $H_2O$, 0–100%) to obtain a semi-pure product (49 mg). Repurification of the impure mixture by normal phase column (25–100%, EtOAc in hexanes) gave *the title compound* as a yellow solid (25 mg, 11%). $^{1}$H NMR (400 MHz, Chloroform-*d*) δ 8.67 (dd, *J* = 4.9, 1.5 Hz, 1H), 8.51 (d, *J* = 2.4 Hz, 1H), 8.12 (s, 1H), 8.07 (s, 1H), 7.53 (app dt, *J* = 8.1, 1.8 Hz, 1H), 7.37 (dd, *J* = 8.0, 4.8 Hz, 1H), 7.23 – 7.21 (m, 1H), 7.22 – 7.17 (m, 1H), 7.08 (app t, *J* = 8.0 Hz, 1H), 7.02 (s, 1H). $^{13}$C NMR (126 MHz, Chloroform–*d*) δ 159.8 (d, $^{1}J_{CF}$ = 252.8 Hz), 153.0, 149.7, 148.7, 143.8, 136.5 (d, $^{3}J_{CF}$ = 10.2 Hz), 136.2, 132.9, 130.3 (d, $^{3}J_{CF}$ = 3.6 Hz), 125.5 (d, $^{4}J_{CF}$ = 3.6 Hz), 124.1, 123.4 (d, $^{2}J_{CF}$ = 13.6 Hz), 117.1 (d, $^{2}J_{CF}$ = 24.6 Hz), 58.6 (d, *J* = 4.2 Hz). LRMS *m/z* (ESI+) 289.0 ([M+H]$^{+}$, 100%). HRMS (ESI+) calcd. for $[C_{14}H_{11}{}^{35}ClFN_4]^+$ 289.0651, found 289.0659 [M+H]$^{+}$. IR (film): $\nu_{max}$ 1610, 1580, 1483, 1408, 1274, 1136, 902, 779, 708 cm$^{-1}$.

*tert*-Butyl 3-((4-chloro-2-fluorophenyl)(pyridin-3-yl)methoxy)azetidine-1-carboxylate, **MYOS_00143**

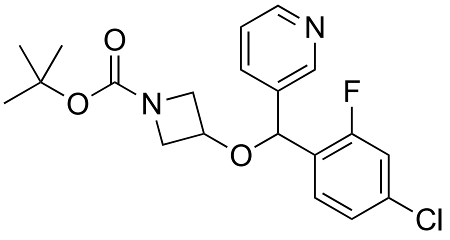

1-Boc-3-hydroxyazetidine (54 mg, 310 μmol, 1.5 equiv.) dissolved in anhydrous MeCN (0.50 mL) was mixed with NaH (13 mg, 310 μmol, 1.5 equiv., 60% dispersion in mineral oil) and the mixture was stirred for 30 min in a 5 mL microwave vial. Aryl halide (53 mg, 207 μmol, 1.0 equiv.) in anhydrous MeCN (0.50 mL) and DMAP (2.5 mg, 21 μmol, 0.10 equiv.) were added to the reaction mixture which was purged with Ar and left stirring at 45°C for 72 h. The reaction was quenched with a saturated solution of $NH_4Cl$ until no further effervescence was observed. The product mixture was extracted with EtOAc (3 × 5 mL), the organic phases combined, dried with $MgSO_4$, filtered and concentrated under reduced pressure to afford the crude product which was purified by Biotage Selekt chromatography on a self-packed celite column (10–100%, EtOAc in hexanes) to obtain semi-pure material. The collected product was repurified by reverse phase chromatography (Biotage Sfar C18 30 g column, 40–60%, MeCN in $H_2O$) to afford *the title compound* as a white solid (24 mg, 30%). $^1$H NMR (400 MHz, Chloroform-*d*) δ 8.61 (d, obscured peak, 1H), 8.57 (dd, $J$ = 4.5, 1.5 Hz, 1H), 7.66 (app dt, $J$ = 8.0, 1.7 Hz, 1H), 7.42 (app t, $J$ = 8.1 Hz, 1H), 7.31 (d, $J$ = 4.9 Hz, 1H), 7.20 (dd, $J$ = 8.2, 2.0 Hz, 1H), 7.11 (dd, $J$ = 9.8, 2.0 Hz, 1H), 5.67 (s, 1H), 4.44 – 4.26 (m, 1H), 4.16 – 3.99 (m, 2H), 4.02 – 3.82 (m, 2H), 1.45 (s, 9H). $^{13}$C NMR (126 MHz, Chloroform–*d*) δ 159.8 (d, $^1J_{CF}$ = 250.3 Hz), 156.3, 149.3, 148.2, 135.9, 135.2 (d, $^3J_{CF}$ = 10.1 Hz), 128.9 (d, $^3J_{CF}$ = 4.5 Hz), 126.5 (d, $^2J_{CF}$ = 13.0 Hz), 125.3 (d, $^4J_{CF}$ = 3.6 Hz), 123.8, 116.6 (d, $^2J_{CF}$ = 25.4 Hz), 79.9, 73.2 (d, $J$ = 2.5 Hz), 66.8, 53.6, 28.4. LRMS *m/z* (ESI+) 393.1 ($[M+H]^+$, 100%), 415.0 ($[M+Na]^+$, 21%). HRMS (ESI+) calcd. for $[C_{20}H_{23}{}^{35}ClFN_2O_3]^+$ 393.1376, found 393.1392 $[M+H]^+$. IR (film): $\nu_{max}$ 2974, 2881, 1695, 1479, 1401, 1155, 1069, 902, 857, 715, 711 cm$^{-1}$.

*N*-((4-Chloro-2-fluorophenyl)(pyridin-3-yl)methyl)aniline, **MYOS_00144**

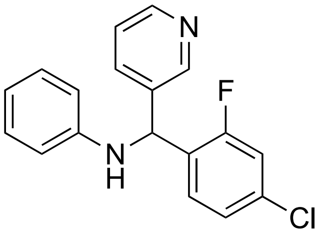

Prepared according to *General Synthetic Procedure B* using **Int2** (246 mg, 961 μmol), aniline (0.13 mL, 1.4 mmol), $Et_3N$ (0.27 mL, 1.9 mmol) and KI (8.0 mg, 48 μmol). The reaction mixture was heated under reflux at 85°C for 48 h to give the crude product (675 mg) which was purified by Biotage Selekt chromatography (20–100%, EtOAc in hexanes) to obtain a semi-pure material (456 mg). The product mixture was repurified by reverse phase chromatography (Biotage Sfar C18 30 g column, 5–100%, MeOH

in $H_2O$) to afford *the title compound* as yellow flakes (162 mg, 54%). m.p. 127–128°C. $^1$H NMR (400 MHz, Chloroform-*d*) δ 8.62 (d, *J* = 2.3 Hz, 1H), 8.55 (dd, *J* = 4.9, 1.6 Hz, 1H), 7.63 (app dt, *J* = 7.7, 1.6 Hz, 1H), 7.41 (app t, *J* = 8.0 Hz, 1H), 7.28 (d, *J* = 4.8 Hz, 1H), 7.18 – 7.08 (m, 4H), 6.76 (app t, *J* = 7.4 Hz, 1H), 6.55 (d, *J* = 7.7 Hz, 2H), 5.80 (d, *J* = 4.4 Hz, 1H), 4.12 (d, *J* = 4.6 Hz, 1H). $^{13}$C NMR (126 MHz, Chloroform–*d*) δ 160.1 (d, $^1J_{CF}$ = 251.0 Hz), 148.4 (d, *J* = 4.6 Hz), 146.3, 137.6, 136.0, 134.8 (d, $^3J_{CF}$ = 10.7 Hz), 129.5, 129.3 (d, $^3J_{CF}$ = 4.6 Hz), 127.2 (d, $^2J_{CF}$ = 13.2 Hz), 125.3 (d, $^4J_{CF}$ = 3.5 Hz), 124.2, 119.0, 117.0 (d, $^2J_{CF}$ = 24.9 Hz), 113.8, 54.3 (d, *J* = 2.7 Hz). LRMS *m/z* (ESI+) 313.1 ([M+H]$^+$, 100%). HRMS (ESI+) calcd. for $[C_{18}H_{15}{}^{35}ClFN_2]^+$ 313.0902, found 313.0904 [M+H]$^+$.

*N*-((4-Chloro-2-fluorophenyl)(pyridin-3-yl)methyl)-*N*-methylaniline, **MYOS_00145**

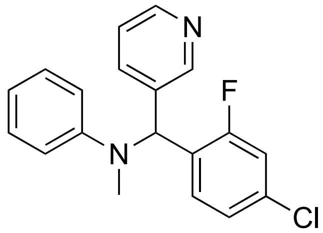


Prepared according to *General Synthetic Procedure B* using **Int2** (93 mg, 363 μmol), *N*-methylaniline (0.06 mL, *ca*. 545 μmol), $Et_3N$ (0.10 mL, 726 μmol) and KI (3.0 mg, 18 μmol). The reaction mixture was heated under reflux at 85°C for 48 h to give the crude product (208 mg) which was purified by Biotage Selekt chromatography (10–95%, EtOAc in hexanes) to afford *the title compound* as a yellow solid (62 mg, 52%). $^1$H NMR (400 MHz, Chloroform-*d*) δ 8.56 (dd, *J* = 4.8, 1.6 Hz, 1H), 8.46 (d, *J* = 2.4 Hz, 1H), 7.49 (app dt, obscured peak, *J* = 8.0 Hz, 1H), 7.31 – 7.27 (m, 1H), 7.25 – 7.20 (m, 2H), 7.15 – 7.07 (m, 3H), 6.85 – 6.70 (m, 3H), 6.31 (s, 1H), 2.76 (s, 3H). $^{13}$C NMR (126 MHz, Chloroform–*d*) δ 160.8 (d, $^1J_{CF}$ = 251.4 Hz), 149.9, 149.6, 149.1, 135.9, 135.3, 134.7 (d, $^3J_{CF}$ = 10.1 Hz), 130.5 (d, $^3J_{CF}$ = 4.6 Hz), 129.4, 125.7 (d, $^2J_{CF}$ = 14.5 Hz), 124.9 (d, $^4J_{CF}$ = 3.7 Hz), 123.6, 118.3, 116.8 (d, $^2J_{CF}$ = 24.9 Hz), 113.8, 59.6, 35.2. LRMS *m/z* (ESI+) 327.1 ([M+H]$^+$, 100%). HRMS (ESI+) calcd. for 327.1059 $[C_{19}H_{17}{}^{35}ClFN_2]^+$, found 327.1067 [M+H]$^+$. IR (film): $\nu_{max}$ 3030, 2817, 1595, 1502, 1405, 1367, 1222, 1099, 898, 745, 693 cm$^{-1}$.

*N*-Benzyl-1-(4-chloro-2-fluorophenyl)-1-(pyridin-3-yl)methanamine, **MYOS_00147**

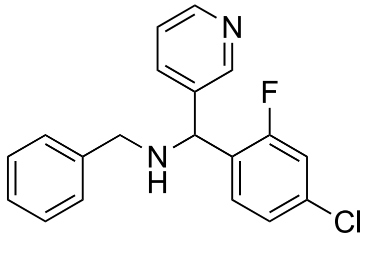


Prepared according to *General Synthetic Procedure B* using **Int2** (250 mg, 976 μmol, 1.0 equiv.), benzylamine (0.16 mL, 1.5 mmol, 1.5 equiv.), DMAP (12 mg, 98 μmol, 0.10 equiv.), anhydrous $Et_3N$ (0.27 mL, 2.0 mmol, 2.0 equiv.) and KI (16 mg, 98 μmol, 0.10 equiv.) to give the crude product (424 mg) which

was purified by Biotage Selekt chromatography (10–85%, EtOAc in hexanes) to afford *the title compound* as a light-yellow viscous liquid (168 mg, 53%). $^{1}$H NMR (500 MHz, Chloroform–*d*) δ 8.64 (d, *J* = 2.2 Hz, 1H), 8.55 (dd, *J* = 4.9, 1.5 Hz, 1H), 8.13 (d, *J* = 8.0 Hz, 1H), 8.03 – 7.80 (m, 1H), 7.64 (app t, *J* = 8.0 Hz, 1H), 7.42 – 7.33 (m, 5H), 7.22 (dd, *J* = 8.4, 2.2 Hz, 1H), 7.09 (dd, *J* = 9.8, 2.1 Hz, 1H), 5.25 (s, 1H), 4.12 (q, *J* = 7.1 Hz, 1H), 3.87 (d, obscured peak, 2H). $^{13}$C NMR (126 MHz, Methanol–$d_4$) δ 161.6 (d, $^{1}J_{CF}$ = 248.5 Hz), 149.5, 149.0, 140.7, 140.2, 137.4, 135.1 (d, $^{3}J_{CF}$ = 10.2 Hz), 130.5 (d, $^{3}J_{CF}$ = 4.7 Hz), 130.0 (d, $^{2}J_{CF}$ = 13.0 Hz), 129.5, 128.2, 126.1 (d, $^{4}J_{CF}$ = 3.5 Hz), 125.4, 117.2 (d, $^{2}J_{CF}$ = 25.8 Hz), 57.2 (d, *J* = 2.5 Hz), 52.4. LRMS *m/z* (ESI+) 327.1 ([M+H]$^+$, 100%). HRMS (ESI+) calcd. for $[C_{19}H_{17}{}^{35}ClFN_2]^+$ 327.1059, found 327.1067 [M+H]$^+$. IR (film): $\nu_{max}$ 1736, 1606, 1576, 1483, 1241,1095, 1043, 894, 857, 812, 700 cm$^{-1}$.

*N*-Benzyl-1-(4-chloro-2-fluorophenyl)-*N*-methyl-1-(pyridin-3-yl)methanamine, **MYOS_00148**

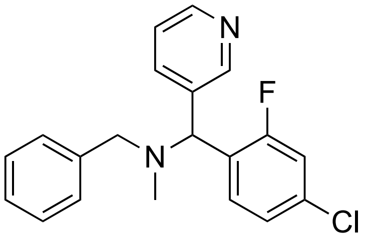


Prepared according to *General Synthetic Procedure B* using **Int2** (142 mg, 554 µmol, 1.0 equiv.), *N*-benzylmethylamine (0.11 mL, 832 µmol, 1.5 equiv.), DMAP (7.0 mg, 55 µmol, 0.10 equiv.), anhydrous $Et_3N$ (0.16 mL, 1.1 mmol, 2.0 equiv.) and KI (9.0 mg, 55 µmol, 0.10 equiv.) to give the crude product (251 mg) which was purified by reversed phase chromatography (Biotage Sfar C18 30 g column, 5–100%, MeOH in $H_2O$) to afford *the title compound* as a yellow viscous liquid (113 mg, 60%). $^{1}$H NMR (500 MHz, Methanol-$d_4$) δ 8.64 (d, *J* = 2.2 Hz, 1H), 8.41 (dd, *J* = 4.9, 1.6 Hz, 1H), 7.98 (app dt, *J* = 8.0, 2.0 Hz, 1H), 7.76 (app t, *J* = 8.1 Hz, 1H), 7.41 (dd, *J* = 8.0, 4.9 Hz, 1H), 7.38 – 7.34 (m, 2H), 7.34 – 7.22 (m, 4H), 7.19 (dd, *J* = 10.2, 2.1 Hz, 1H), 4.95 (s, 1H), 3.57 – 3.44 (m, 2H), 2.07 (s, 3H). $^{13}$C NMR (126 MHz, Methanol–$d_4$) δ 161.8 (d, $^{1}J_{CF}$ = 249.2 Hz), 149.8, 149.2, 140.1, 139.6, 137.9, 135.2 (d, $^{3}J_{CF}$ = 10.3 Hz), 131.5 (d, $^{3}J_{CF}$ = 4.6 Hz), 129.7, 129.4, 128.8 (d, $^{2}J_{CF}$ = 12.8 Hz), 128.2, 126.3 (d, $^{4}J_{CF}$ = 3.6 Hz), 125.5, 117.4 (d, $^{2}J_{CF}$ = 26.5 Hz), 65.5, 60.6, 40.4. LRMS *m/z* (ESI+) 341.1 ([M+H]$^+$, 100%).HRMS (ESI+) calcd. for $[C_{20}H_{19}{}^{35}ClFN_2]^+$ 341.1215, found 341.1212 [M+H]$^+$. IR (film): $\nu_{max}$ 3010, 2840, 2791, 1606, 1576, 1479, 1408, 1215, 1073, 1017, 902, 857, 730, 697 cm$^{-1}$.

3–((4–Chloro–2–fluorophenyl)(pyridin–3–yl)methyl)–8–oxa–3–azabicyclo[3.2.1]octane, **MYOS_00149**

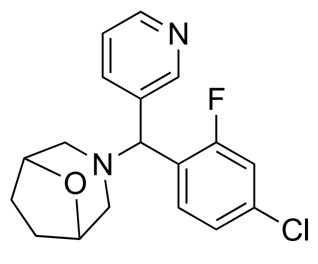

Prepared according to *General Synthetic Procedure B* using 3–(chloro(4–chloro–2–fluorophenyl)methyl)pyridine (**Int2**, 100 mg, 1.0 equiv.), 8–oxa–3–azabicyclo[3.2.1]octane hydrochloride (88 mg, 1.5 equiv.), DMAP (5 mg, 10 mol%), anhydrous $Et_3N$ (0.11 mL, 2.0 equiv.) and a KI catalyst (7 mg, 10 mol%) to give the crude product (142 mg) which was purified by Biotage Selekt chromatography (15–80%, eluent EtOAc in hexanes) to afford *the title compound* as a white liquid (78 mg, 60%). $^1$H NMR (400 MHz, Chloroform–*d*) δ 8.68 (d, obscured peak, 1H), 8.50 (d, $J$ = 4.6 Hz, 1H), 7.76 (app dt, obscured peak, $J$ = 7.9 Hz, 1H), 7.48 (app t, $J$ = 8.0 Hz, 1H), 7.29 (dd, $J$ = 7.9, 4.9 Hz, 1H), 7.14 (dd, $J$ = 8.5, 2.1 Hz, 1H), 7.04 (dd, $J$ = 9.9, 2.1 Hz, 1H), 4.69 (s, 1H), 4.27 (p, $J$ = 5.6, 1.9 Hz, 2H), 2.62 – 2.45 (m, 2H), 2.32 – 2.21 (m, 2H), 2.07 – 2.04 (m, 2H), 1.95 – 1.89 (m, 2H). $^{13}$C NMR (101 MHz, Chloroform–*d*) δ 160.5 (d, $^1J_{CF}$ = 250.1 Hz), 149.8, 149.0, 136.5, 135.6, 133.9 (d, $^3J_{CF}$ = 10.6 Hz), 129.6 (d, $^3J_{CF}$ = 4.9 Hz), 127.0 (d, $^2J_{CF}$ = 12.7 Hz), 125.2 (d, $^4J_{CF}$ = 3.6 Hz), 123.8, 116.8 (d, $^2J_{CF}$ = 26.0 Hz), 74.8 (d, $J$ = 2.0 Hz), 64.7 (d, $J$ = 1.6 Hz), 57.3 (d, $J$ = 70.7 Hz), 28.7 (d, $J$ = 1.8 Hz). LRMS *m/z* (ESI+) 333.0 ([M+H]$^+$, 100%). HRMS (ESI) calcd. for 333.1164 [$C_{18}H_{19}{}^{35}ClFN_2O$]$^+$, found 333.1167. IR (film): $\nu_{max}$ 2950, 1610, 1483,1427, 1215, 1159, 1069, 1025, 995, 745, 667 cm$^{-1}$.

3–((4–Chloro–2–fluorophenyl)(4–methoxypiperidin–1–yl)methyl)pyridine, **MYOS_00150**

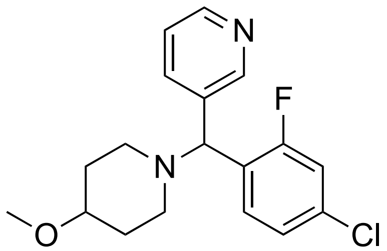

Prepared according to *General Synthetic Procedure B* using 3–(chloro(4–chloro–2–fluorophenyl)methyl)pyridine (**Int2**, 124 mg, 1.0 equiv.), 4–methoxypiperidine hydrochloride (110 mg, 1.5 equiv.), DMAP (6 mg, 10 mol%), anhydrous $Et_3N$ (0.14 mL, 2.0 equiv.) and a KI catalyst (8 mg, 10 mol%) to give the crude product (160 mg) which was purified by Biotage Selekt chromatography (15–90%, eluent EtOAc in hexanes) to afford *the title compound* as a light yellow oil (49 mg, 30%). $^1$H NMR (400 MHz, Chloroform-*d*) δ 8.62 (d, $J$ = 2.3 Hz, 1H), 8.46 (dd, $J$ = 4.8, 1.6 Hz, 1H), 7.68 (d, $J$ = 7.9 Hz, 1H), 7.51 (app t, $J$ = 8.0 Hz, 1H), 7.21 (dd, $J$ = 7.9, 4.8 Hz, 1H), 7.12 (dd, $J$ = 8.4, 1.5 Hz, 1H), 7.02 (dd, $J$ = 9.9, 2.1 Hz, 1H), 4.70 (s, 1H), 3.31 (s, 3H), 3.29 – 3.17 (m,

1H), 2.77 – 2.60 (m, 2H), 2.18 – 2.02 (m, 2H), 1.93 – 1.83 (m, 2H), 1.66 – 1.53 (m, 2H). $^{13}$C NMR (101 MHz, Chloroform–*d*) δ 159.2, 149.8, 148.9, 137.3, 135.5, 133.8, 129.8 (d, $^{3}J_{CF}$ = 4.3 Hz), 127.6 (d, $^{2}J_{CF}$ = 13.0 Hz), 125.1 (d, $^{4}J_{CF}$ = 2.6 Hz), 123.7, 116.6 (d, $^{2}J_{CF}$ = 25.9 Hz), 76.3, 64.4, 55.7, 49.3, 31.1. LRMS *m/z* (ESI+) 335.1 ([M+H]$^{+}$, 100%). HRMS (ESI) calcd. for 335.1321 [$C_{18}H_{21}{}^{35}ClFN_2O$]$^{+}$, found 335.1328. IR (film): $\nu_{max}$ 2839, 1483, 1215, 1088, 898, 749, 667 cm$^{-1}$.

1–((4–Chloro–2–fluorophenyl)(pyridin–3–yl)methyl)–4–methylpiperidin–4–ol, **MYOS_00151**

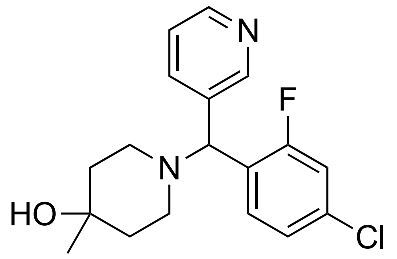


Prepared according to *General Synthetic Procedure B* using 3–(chloro(4–chloro–2–fluorophenyl)methyl)pyridine (**Int2**, 100 mg, 1.0 equiv.), 4–methylpiperidin–4–ol (68 mg, 1.5 equiv.), DMAP (5 mg, 10 mol%), anhydrous $Et_3N$ (0.11 mL, 2.0 equiv.) and a KI catalyst (7 mg, 10 mol%) to give the crude product (113 mg) which was purified by Biotage Selekt chromatography (5–90%, EtOAc in hexanes) to afford *the title compound* as a yellow oil (65 mg, 50%). $^{1}$H NMR (400 MHz, DMSO–$d_6$) δ 8.55 (d, $J$ = 2.3 Hz, 1H), 8.43 (dd, $J$ = 4.7, 1.6 Hz, 1H), 7.72 (app dt, $J$ = 7.9, 2.0 Hz, 1H), 7.62 (app t, $J$ = 8.2 Hz, 1H), 7.51 – 7.22 (m, 3H), 4.74 (s, 1H), 4.11 (s, 1H), 2.44 – 1.90 (m, 4H), 1.63 – 1.33 (m, 4H), 1.09 (s, 3H). $^{13}$C NMR (101 MHz, DMSO–$d_6$) δ 159.8 (d, $^{1}J_{CF}$ = 248.6 Hz), 149.0, 148.5, 136.8, 135.2, 132.4 (d, $^{3}J_{CF}$ = 10.7 Hz), 130.3 (d, $^{3}J_{CF}$ = 5.2 Hz), 127.9 (d, $^{2}J_{CF}$ = 12.9 Hz), 125.1 (d, $^{4}J_{CF}$ = 3.4 Hz), 123.8, 116.2 (d, $^{2}J_{CF}$ = 26.3 Hz), 79.2, 65.6, 64.0, 47.8, 47.4, 38.5 (d, $J$ = 4.4 Hz). LRMS *m/z* (ESI+) 335.0 ([M+H]$^{+}$, 100%). HRMS (ESI) calcd. for 335.1321 [$C_{18}H_{21}{}^{35}ClFN_2O$]$^{+}$, found 335.1320. IR (film): $\nu_{max}$ 3300 (O–H), 2959, 2821, 1580, 1483, 1408, 1215, 1088, 898, 752 cm$^{-1}$.

(4-Bromophenyl)(4-chlorophenyl)(phenyl)methanol, **MYOS_00163**

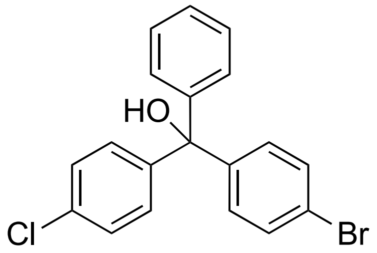


Prepared according to *General Synthetic Procedure C,* using phenylmagnesium bromide (1 M in THF, 1.0 mL, 1.0 mmol) and intermediate **MYOS_00134** (15 mg, 0.50 mmol). The crude mixture was purified by column chromatography (hexane/ethyl acetate) to give *the title compound* as a light straw-coloured gel (24 mg, 13 %). $^{1}$H NMR (500 MHz, Acetone-$d_6$) $\delta$ 7.50 (d, $J$ = 2.0 Hz, 1H),

7.49 (d, $J$ = 2.1 Hz, 1H), 7.38 – 7.17 (m, 11H), 5.60 (s, 1H); $^{13}$C NMR (126 MHz, Acetone-$d_6$) $\delta$ 147.6, 147.7, 147.2, 133.3, 131.6, 131.0, 130.9, 130.6, 128.7, 128.1, 121.5, 81.5; LRMS *m/z* (ESI) 375 ([M–H]$^-$, 16%), 373 ([M–H]$^-$, 100%), 371 ([M–H]$^-$, 80%); HRMS (ESI) calcd. for $[C_{19}H_{13}{}^{81}Br^{37}ClO]^-$ 374.97939, $[C_{19}H_{13}{}^{79}Br^{37}ClO]^-$ and $[C_{19}H_{13}{}^{81}Br^{35}ClO]^-$ 372.98211, $[C_{19}H_{13}{}^{79}Br^{35}ClO]^-$ 370.98438 ([M–H]$^-$), found 374.97920, 372.98179, 370.98416; IR (film) $\nu_{max}$ 3059 (br), 2929, 1671, 1586, 1303, 1011, 927 and 747 cm$^{-1}$.

1,1-Diphenylpropan-1-ol, **MYOS_00164**

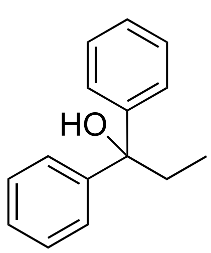


Prepared according to *General Synthetic Procedure C,* using phenylmagnesium bromide (1 M in THF, 1.2 mL, 1.2 mmol) and propiophenone (100 mg, 0.75 mmol). The crude mixture was purified by column chromatography (hexane/ethyl acetate) to give *the title compound* as a viscous transparent colourless gel (330 mg, 69%). $^1$H NMR (400 MHz, Acetone-d$_6$) $\delta$ 7.50 (dd, $J$ = 8.4, 1.4 Hz, 3H), 7.27 (app t, $J$ = 7.7 Hz, 3H), 7.16 (app t, $J$ = 7.3 Hz, 1H), 4.43 (s, 1H), 2.35 (q, $J$ = 7.3 Hz, 2H), 0.86 (t, $J$ = 7.3 Hz, 3H); $^{13}$C NMR (101 MHz, Acetone-d$_6$) $\delta$ 149.1, 128.5, 126.9, 78.2, 35.0, 8.5; LRMS *m/z* (ESI) 235 ([M+Na]$^+$, 34%); HRMS (ESI) calcd. for $[C_{15}H_{16}ONa]^+$ 235.10934 ([M+Na]$^+$), found 235.10935; IR (film) $\nu_{max}$ 3459 (br), 3058, 3025, 2971, 2936, 1599, 1447, 1001, 752, 696 and 637 cm$^{-1}$. Consistent with literature spectroscopic data.[8]

9-Phenyl-9H-fluoren-9-ol, **MYOS_00165**

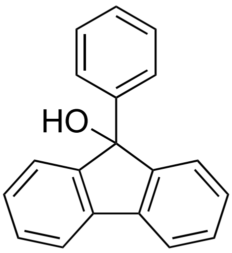


Prepared according to *General Synthetic Procedure C*, using phenylmagnesium bromide (1 M in THF, 1.2 mL, 1.2 mmol) and 9*H*-fluoren-9-one (140 mg, 0.75 mmol). The crude mixture was purified by column chromatography (hexane/ethyl acetate) to give *the title compound* as a translucent semi-lustrous white crystalline solid (120 mg, 64%). m.p. 138–140°C (no lit. m.p.). $^1$H NMR (400 MHz, Acetone-d$_6$) $\delta$ 7.78 (d, $J$ = 7.5 Hz, 1H), 7.41 – 7.32 (m, 3H), 7.31 – 7.11 (m, 4H), 5.19 (s, 1H); $^{13}$C NMR (101 MHz, Acetone-d$_6$) $\delta$ 152.5, 145.9, 140.6, 129.5, 128.9, 128.8, 127.6, 126.2, 125.6, 120.8, 84.0; LRMS *m/z* (ESI) 281 ([M+Na]$^+$, 49%); HRMS (ESI) calcd. for $[C_{19}H_{14}ONa]^+$ 281.09369 ([M+Na]$^+$), found 281.09361; IR (film) $\nu_{max}$ 3334 (br), 3052, 1740, 1489, 1170, 917 and 732 cm$^{-1}$. Consistent with literature spectroscopic data.[9]

(4-Bromophenyl)(phenyl)(pyridine-3-yl)methanol, **MYOS_00166**

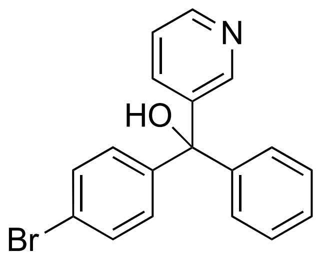

Prepared according to *General Synthetic Procedure C,* using phenylmagnesium bromide (1 M in THF, 0.60 mL, 0.60 mmol) and intermediate **Int8** (110 mg, 0.40 mmol). The crude mixture was purified by column chromatography (hexane/ethyl acetate) to give *the title compound* as a dull opaque coarse white powder (78 mg, 57%). m.p. 124–126°C (no lit. m.p.). $^{1}$H NMR (500 MHz, Acetone-$d_6$) $\delta$ 8.50 (dd, $J$ = 2.4, 0.9 Hz, 1H), 8.46 (dd, $J$ = 4.7, 1.7 Hz, 1H), 7.67 (ddd, $J$ = 8.0, 2.4, 1.6 Hz, 1H), 7.56 – 7.48 (m, 2H), 7.40 – 7.23 (m, 9H), 5.81 (s, 1H); $^{13}$C NMR (126 MHz, Acetone-$d_6$) $\delta$ 150.2, 149.1, 147.4, 147.3, 143.5, 136.0, 131.7, 130.9, 128.9, 128.7, 128.2, 123.5, 121.7, 80.6; LRMS *m/z* (ESI) 342 ([M+H]$^+$, 100%) and 340 ([M+H]$^+$, 94%); HRMS calcd. for [$C_{18}H_{15}{}^{81}BrNO$]$^+$ 342.03111 and [$C_{18}H_{15}{}^{79}BrNO$]$^+$ 340.03315 ([M+H]$^+$), found 342.03103 and 340.03315; IR (film) $\nu_{max}$ 3152 (br), 3058, 1591, 1482, 1421, 1075, 803, 703 and 644 cm$^{-1}$.

(4-Chloro-2-fluorophenyl)(phenyl)(pyridine-3-yl)methanol, **MYOS_00167**

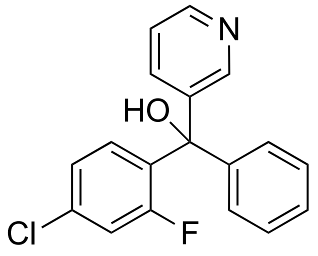

Prepared according to *General Synthetic Procedure C* using phenylmagnesium bromide (1 M in THF, 0.60 mL, 0.60 mmol) and intermediate **Int11** (95 mg, 0.40 mmol). The crude mixture was purified by column chromatography (hexane/ethyl acetate) to give *the title compound* as a dull opaque coarse white powder (80 mg, 53%). m.p. 131–132°C (no lit. m.p.). $^{1}$H NMR (500 MHz, Acetone-$d_6$) $\delta$ 8.52 (d, $J$ = 2.7 Hz, 1H), 8.45 (dd, $J$ = 4.7, 1.7 Hz, 1H), 7.73 – 7.68 (m, 1H), 7.49 (app t, $J$ = 8.6 Hz, 1H), 7.39 – 7.30 (m, 6H), 7.27 (ddd, $J$ = 8.5, 2.2, 0.7 Hz, 1H), 7.21 (dd, $J$ = 11.1, 2.1 Hz, 1H), 5.98 (s, 1H); $^{13}$C NMR (126 MHz, Acetone-$d_6$) $\delta$ 160.4 (d, $^{1}J_{CF}$ = 251.5 Hz), 149.5 (d, $^{5}J_{CF}$ = 2.2 Hz), 148.8, 145.6, 142.1, 135.5 (d, $^{5}J_{CF}$ = 2.0 Hz), 134.8 (d, $^{3}J_{CF}$ = 10.3 Hz), 133.7 (d, $^{2}J_{CF}$ = 11.5 Hz), 131.0 (d, $^{3}J_{CF}$ = 4.1 Hz), 128.7, 128.2, 128.0 (d, $^{5}J_{CF}$ = 1.3 Hz), 124.9 (d, $^{4}J_{CF}$ = 3.5 Hz), 123.4,

117.3 (d, $^{2}J_{CF}$ = 26.6 Hz), 78.6 (d, $^{3}J_{CF}$ = 1.6 Hz); LRMS *m/z* (ESI) 316 ([M+H]$^+$, 35%) and 314 ([M+H]$^+$, 100%); HRMS calcd. for $[C_{18}H_{14}{}^{37}ClFNO]^+$ 316.07130 and $[C_{18}H_{14}{}^{35}ClFNO]^+$ 314.07425 ([M+H]$^+$), found 316.07122 and 314.07418; IR (film) $\nu_{max}$ 3094 (br), 2791, 1736, 1480, 1242, 1161, 866 and 699 cm$^{-1}$.

Diphenyl(pyridine-3-yl)methanol, **MYOS_00168**

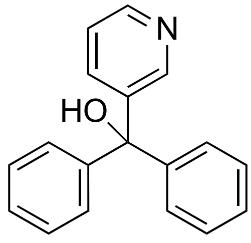


Prepared according to *General Synthetic Procedure C*, using phenylmagnesium bromide (1 M in THF, 0.75 mL, 0.75 mmol) and phenyl(pyridin-3-yl)methanone (95 mg, 0.50 mmol). The crude mixture was purified by column chromatography (hexane/ethyl acetate) to give *the title compound* as a light straw-coloured gel (58 mg, 54%). $^{1}$H NMR (500 MHz, Acetone-*d$_6$*) $\delta$ 8.50 (d, *J* = 2.4 Hz, 1H), 8.44 (dd, *J* = 4.8, 1.6 Hz, 1H), 7.72 – 7.61 (m, 1H), 7.45 – 7.15 (m, 13H), 5.72 (s, 1H); $^{13}$C NMR (126 MHz, Acetone-*d$_6$*) $\delta$ 150.3, 148.8, 147.9, 144.1, 136.1, 128.7, 128.7, 128.0, 123.4, 80.9; LRMS *m/z* (ESI) 284 ([M+HNa]$^+$, 22%) and 262 ([M+H]$^+$, 100%); HRMS (ESI) calcd. for $[C_{18}H_{16}NO]^+$ 262.12264 ([M+H]$^+$), found 262.12270; IR (film) $\nu_{max}$ 3052 (br), 2769, 1593, 1477, 1022, 759, 700 and 635 cm$^{-1}$. Consistent with literature spectroscopic data.[10]

1-Phenyl-1-(pyridine-3-yl)propan-1-ol, **MYOS_00169**

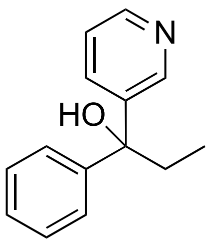


Prepared according to *General Synthetic Procedure C*, using ethylmagnesium bromide (3 M in THF, 0.5 mL, 1.5 mmol) and phenyl(pyridin-3-yl)methanone (180 mg, 1.0 mmol). The crude mixture was purified by column chromatography (hexane 20–40% in ethyl acetate) to give three fractions. Fraction A gave the starting ketone (8 mg, 4%, $R_f$ = 0.5), fraction B gave *the title compound* as an opaque dull straw-coloured powder (37 mg, 17%, $R_f$ = 0.3) and fraction C gave the starting ketone reduced to alcohol (80 mg, 44%, $R_f$ = 0.1). m.p. 98.6–99.3°C (no lit. m.p.). $^{1}$H NMR (500 MHz, Chloroform-*d*) $\delta$ 8.54 (s, 1H), 8.30 (d, *J* = 4.5 Hz, 1H), 7.73 (app dt, *J* = 9.7, 2.0 Hz, 1H), 7.42 – 7.38 (m, 1H), 7.33 – 7.28 (m, 1H), 7.25 – 7.19 (m, 1H), 7.17 (dd, *J* = 8.1, 4.7 Hz, 1H), 3.70 (s, 1H), 2.29 (qd, *J* = 7.1, 5.5 Hz, 2H), 0.87 (t, *J* = 7.3 Hz, 3H); $^{13}$C NMR (126 MHz, Chloroform-*d*) $\delta$ 147.7, 147.5, 146.3, 142.8, 134.3,

128.4, 127.2, 126.2, 123.1, 77.1, 34.4, 8.1; LRMS *m/z* (ESI) 214 ([M+H]$^+$, 100%); HRMS (ESI) calcd. for $[C_{14}H_{16}NO]^+$ 214.12264 ([M+H]$^+$), found 214.12260; IR (film) $\nu_{max}$ 3162 (br), 2970, 1588, 1475, 1448, 1026, 810, 754 and 699 cm$^{-1}$.

1-(4-Bromophenyl)-2-phenyl-1-(pyridine-3-yl)ethan-1-ol, **MYOS_00170**

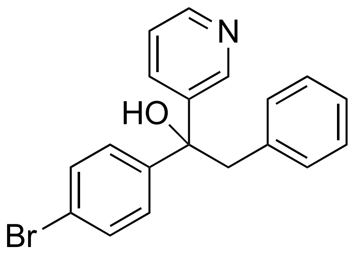


Prepared according to *General Synthetic Procedure C*, using benzylmagnesium chloride (1 M in THF, 0.60 mL, 0.60 mmol) and **Int8** (110mg, 0.40 mmol). The crude mixture was purified by column chromatography (hexane/ethyl acetate) to give *the title compound* as a light straw-coloured oil (75 mg, 55%). $^1$H NMR (500 MHz, Acetone-$d_6$) $\delta$ 8.65 (s, 1H), 8.40 – 8.30 (m, 1H), 7.79 (ddd, $J$ = 8.1, 2.4, 1.7 Hz, 1H), 7.50 – 7.41 (m, 4H), 7.24 (dd, $J$ = 7.9, 4.5 Hz, 1H), 7.13 – 7.07 (m, 3H), 7.05 – 7.00 (m, 2H), 5.17 (s, 1H), 3.70 (s, 2H); $^{13}$C NMR (126 MHz, Acetone-$d_6$) $\delta$ 148.9, 148.5, 147.4, 143.5, 137.5, 134.7, 131.9, 131.7, 129.5, 128.2, 126.9, 123.5, 121.1, 77.2, 47.7; LRMS *m/z* (ESI) 378 ([M+HNa]$^+$, 22%), 376 ([M+HNa]$^+$, 21%), 356 ([M+H]$^+$, 99%) and 354 ([M+H]$^+$, 100%); HRMS (ESI) calcd. for $[C_{19}H_{17}{}^{81}BrNO]^+$ 356.04676 and $[C_{19}H_{17}{}^{79}BrNO]^+$ 354.04880, found 356.04661 and 354.04869; IR (film) $\nu_{max}$ 3153 (br), 2937, 1586, 1452, 1110, 821 and 700 cm$^{-1}$.

1-(4-Chloro-2-fluorophenyl)-2-phenyl-1-(pyridine-3-yl)ethan-1-ol, **MYOS_00171**

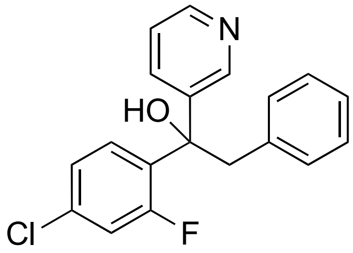


Prepared according to *General Synthetic Procedure C*, using benzylmagnesium chloride (1 M in THF, 0.60 mL, 0.60 mmol) and **Int11** (89 mg, 0.40 mmol). The crude mixture was purified by column chromatography (hexane/ethyl acetate) to give *the title compound* as a light straw-coloured oil (55 mg, 44%). $^1$H NMR (500 MHz, DMSO-$d_6$) $\delta$ 8.56 (d, $J$ = 2.4 Hz, 1H), 8.43 (dd, $J$ = 4.9, 1.6 Hz, 1H), 7.75 (app dt, $J$ = 8.1, 2.0 Hz, 1H), 7.48 (app t, $J$ = 8.6 Hz, 1H), 7.34 (dd, $J$ = 8.0, 4.7 Hz, 1H), 7.26 (dd, $J$ = 11.4, 2.2 Hz, 1H), 7.16 (dd, $J$ = 8.5, 2.2 Hz, 1H), 7.11 – 7.06 (m, 3H), 6.98 – 6.94 (m, 2H), 6.28 (s, 1H), 3.74 – 3.56 (m, 2H); $^{13}$C NMR (126 MHz, DMSO-$d_6$) $\delta$ 159.0 (d, $^1J_{CF}$ = 249.6 Hz), 148.3, 148.0, 142.6, 137.0, 134.3, 133.0 (d, $^3J_{CF}$ = 10.6 Hz), 132.5 (d, $^2J_{CF}$ = 11.3 Hz), 130.9, 129.9 (d, $^3J_{CF}$ = 4.8 Hz), 127.8, 126.5, 124.5, 123.5, 116.5 (d, $^2J_{CF}$ = 26.6 Hz), 74.5,

44.4; LRMS *m/z* (ESI) 330 ([M+H]$^+$, 31%) and 328 ([M+H]$^+$, 100%); HRMS (ESI) calcd. for [$C_{19}H_{16}{}^{37}ClFNO$]$^+$ 330.08695 and [$C_{19}H_{16}{}^{35}ClFNO$]$^+$ 328.08990 ([M+H]$^+$), found 330.08692 and 328.08992; IR (film) $\nu_{max}$ 3130 (br), 2778, 1606, 1494, 1111, 876 and 701 cm$^{-1}$.

1,2-Diphenyl-1-(pyridin-3-yl)ethan-1-ol, **MYOS_00172**

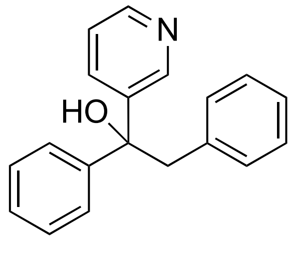


Prepared according to *General Synthetic Procedure C*, using benzylmagnesium chloride (1 M in THF, 0.60 mL, 0.60 mmol) and phenyl(pyridin-3-yl)methanone (94 mg, 0.40 mmol). The crude mixture was purified by column chromatography (hexane/ethyl acetate) to give *the title compound* as a light straw-coloured oil (64 mg, 48%). $^1$H NMR (500 MHz, Chloroform-*d*) $\delta$ 8.60 (dd, $J$ = 2.4, 0.8 Hz, 1H), 8.38 (dd, $J$ = 4.8, 1.6 Hz, 1H), 7.68 (ddd, $J$ = 8.0, 2.4, 1.6 Hz, 1H), 7.45 – 7.40 (m, 2H), 7.35 – 7.28 (m, 2H), 7.29 – 7.21 (m, 1H), 7.18 – 7.13 (m, 4H), 6.92 – 6.87 (m, 2H), 3.69 – 3.56 (m, 2H), 3.02 (s, 1H); $^{13}$C NMR (126 MHz, Chloroform-*d*) $\delta$ 148.0, 147.9, 145.9, 142.2, 135.3, 134.2, 131.0, 128.5, 128.3, 127.4, 127.1, 126.3, 123.0, 76.9, 47.9; LRMS *m/z* (ESI) 276 ([M+H]$^+$, 100%); HRMS (ESI) calcd. for [$C_{19}H_{18}NO$]$^+$ 276.13829 ([M+H]$^+$), found 276.13831; IR (film) $\nu_{max}$ 3207 (br), 2924, 1573, 1493, 1450, 1224, 1046, 818 and 700 cm$^{-1}$.

1-(4-Bromo-2-fluorophenyl)-2-phenyl-1-(pyridin-3-yl)ethan-1-ol, **MYOS_00173**

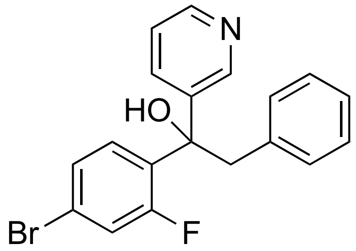


Prepared according to *General Synthetic Procedure C*, using benzylmagnesium chloride (1 M in THF, 0.60 mL, 0.60 mmol) and intermediate **Int10** (110 mg, 0.4 mmol). The crude mixture was purified by column chromatography (hexane/ethyl acetate) to give *the title compound* as an opaque dull straw-coloured powder (65 mg, 46%). m.p. 115–116°C (no lit. m.p.). $^1$H NMR (500 MHz, DMSO-$d_6$) $\delta$ 8.58 (s, 1H), 8.44 (s, 1H), 7.81 – 7.68 (m, 1H), 7.42 (app t, $J$ = 8.5 Hz, 1H), 7.37 (dd, $J$ = 11.1, 2.0 Hz, 1H), 7.32 (dd, $J$ = 8.0, 4.7 Hz, 1H), 7.28 (dd, $J$ = 8.5, 2.0 Hz, 1H), 7.12 – 7.05 (m, 4H), 6.96 (dd, $J$ = 7.2, 2.3 Hz, 2H), 6.26 (s, 1H), 3.75 – 3.54 (m, 2H); $^{13}$C NMR (126 MHz, DMSO-$d_6$) $\delta$ 158.6 (d, $^1J_{CF}$ = 250.6 Hz), 147.9, 147.7, 142.1, 136.6, 133.8, 132.6 (d, $^3J_{CF}$ = 11.2 Hz), 130.5, 129.9 (d, $^3J_{CF}$ = 4.6 Hz), 127.3, 127.0 (d, $^4J_{CF}$ = 3.0 Hz), 126.1, 123.0, 120.5

(d, $^2J_{CF}$ = 9.9 Hz), 118.8 (d, $^2J_{CF}$ = 26.2 Hz), 74.2, 44.0; LRMS *m/z* (ESI) 374 ([M+H]$^+$, 99%), 372 ([M+H]$^+$, 99%); HRMS (ESI) calcd. for [$C_{19}H_{16}{}^{81}BrFNO$]$^+$ 374.03733 and [$C_{19}H_{16}{}^{79}BrFNO$]$^+$ 372.03938 ([M+H]$^+$), found 374.03728 and 372.03934; IR (film) $\nu_{max}$ 3121 (br), 2780, 1599, 1493, 1453, 1077, 852 and 701 cm$^{-1}$.

(4-Bromo-2-fluorophenyl)(phenyl)(pyridin-3-yl)methanol, **MYOS_00174**

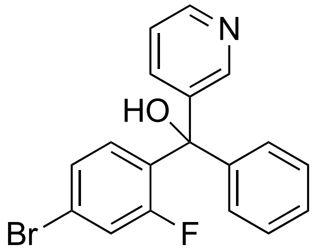

Prepared according to *General Synthetic Procedure C,* using phenylmagnesium bromide (1 M in THF, 0.60 mL, 0.60 mmol) and intermediate **Int10** (110 mg, 0.40 mmol). The crude mixture was purified by column chromatography (hexane/ethyl acetate) to give *the title compound* as a dull opaque coarse white powder (100 mg, 75%). m.p. 149–150°C (no lit. m.p.). $^1$H NMR (500 MHz, Chloroform-*d*) $\delta$ 8.51 – 8.34 (m, 2H), 7.63 (app dt, $J$ = 8.1, 2.0 Hz, 1H), 7.36 – 7.29 (m, 3H), 7.27 – 7.18 (m, 5H), 6.90 (app t, $J$ = 8.7 Hz, 1H), 4.25 (s, 1H); $^{13}$C NMR (126 MHz, Chloroform-*d*) $\delta$ 160.3 (d, $^1J_{CF}$ = 251.3 Hz), 149.0, 148.7, 144.2, 140.9, 135.3, 132.7 (d, $^3J_{CF}$ = 10.5 Hz), 130.8 (d, $^3J_{CF}$ = 3.7 Hz), 128.5, 128.2, 127.4, 127.4, 123.1, 122.6 (d, $^2J_{CF}$ = 10.0 Hz), 120.1 (d, $^2J_{CF}$ = 26.1 Hz), 79.2; LRMS *m/z* (ESI) 360 ([M+H]$^+$, 100%), 358 ([M+H]$^+$, 98%); HRMS (ESI) calcd. for [$C_{18}H_{14}{}^{81}BrFNO$]$^+$ 360.02168 and [$C_{18}H_{14}{}^{79}BrFNO$]$^+$ 358.02373 ([M+H]$^+$), found 360.02162 and 358.02374; IR (film) $\nu_{max}$ 3095 (br), 2789, 1736, 1600, 1448, 1073, 760 and 648 cm$^{-1}$.

(4-Bromophenyl)(4-chlorophenyl)(pyridin-3-yl)methanol, **MYOS_00175**

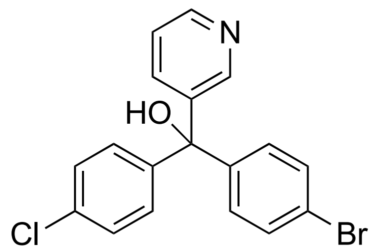

Prepared according to *General Synthetic Procedure A* using 3-bromopyridine (2.5 mL, 0.90 mmol) and intermediate **MYOS_00134** (260 mg, 0.40 mmol). The crude mixture was purified by column chromatography (hexane/ethyl acetate) to give *the title compound* as a dull, opaque, coarse, white powder (120 mg, 38%). m.p. 106–108°C (no lit. m.p.). $^1$H NMR (500 MHz, DMSO-$d_6$) $\delta$ 8.48 (dd, $J$ = 4.7, 1.6 Hz, 1H), 8.40 (dd, $J$ = 2.4, 0.9 Hz, 1H), 7.59 – 7.50 (m, 3H), 7.43 – 7.38 (m, 2H), 7.36 (ddd, J = 8.0, 4.8, 0.9 Hz, 1H), 7.27 – 7.20 (m, 2H), 7.19 – 7.07 (m, 2H), 6.89 (s, 1H); $^{13}$C NMR (126 MHz, DMSO-$d_6$) $\delta$ 148.8, 148.2, 145.9, 145.4, 142.1, 135.1, 132.0, 130.9, 129.8, 129.5, 127.9, 123.0, 120.6, 78.8; LRMS *m/z* (ESI) 378 ([M+H]$^+$, 27%), 376 ([M+H]$^+$, 97%) and 374 ([M+H]$^+$, 71%); HRMS (ESI) calcd. for [$C_{18}H_{14}{}^{81}Br^{37}ClNO$]$^+$ 377.98918,

$[C_{18}H_{14}{}^{79}Br^{37}ClNO]^+$ and $[C_{18}H_{14}{}^{81}Br^{35}ClNO]^+$ 375.99191, and $[C_{18}H_{14}{}^{79}Br^{35}ClNO]^+$ 373.99418 ($[M+H]^+$), found 377.98911, 375.99217 and 373.99402; IR (film) $\nu_{max}$ 3047 (br), 2788, 1481, 1415, 1164, 1009, 816 and 709 $cm^{-1}$. Consistent with incomplete literature spectroscopic data.[5]

3–((Azetidin–3–yloxy)(4–chloro–2–fluorophenyl)methyl)pyridine, **MYOS_00190**[11]

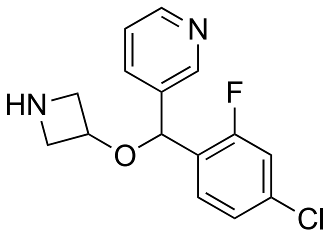

In a 5 mL round–bottomed flask equipped with a magnetic stirrer was dissolved *tert*–butyl 3–((4–chloro–2–fluorophenyl)(pyridin–3–yl)methoxy)azetidine–1–carboxylate (**MYOS_00143**, 45 mg, 1.0 equiv.) in 1,4–dioxane (1.5 mL). The solution was dropwise charged with 4 M $HCl_{(aq)}$ (0.26 mL, 9.0 equiv.) and the mixture was left to stir for 3 h at rt. After reaction completion, the mixture was concentrated under high pressure and the residue partitioned between $Et_2O$ and $H_2O$, followed by basification with $NaHCO_{3(sat)}$. The aq phase was extracted with $Et_2O$ (3 × 4 mL), the organic extracts combined, dried over $MgSO_4$ and concentrated *in vacuo* to afford the *title* compound as a colourless liquid (31 mg, 92%). $^1$H NMR (400 MHz, Methanol-$d_4$) δ 8.89 (br s, 1H), 8.62 (d, $J$ = 2.3 Hz, 1H), 8.53 (dd, $J$ = 4.9, 1.6 Hz, 1H), 7.86 (app dt, $J$ = 8.0, 2.0 Hz, 1H), 7.61 – 7.55 (m, 1H), 7.47 (dd, $J$ = 8.0, 4.9 Hz, 1H), 7.32 (dd, $J$ = 8.3, 2.1 Hz, 1H), 7.28 (dd, $J$ = 10.2, 2.1 Hz, 1H), 5.95 (s, 1H), 4.65 – 4.58 (m, 1H), 4.29 – 4.21 (m, 2H), 4.13 – 4.03 (m, 2H). $^{13}$C NMR (126 MHz, Chloroform–$d$) δ 159.7 (d, $^1J_{CF}$ = 251.1 Hz), 148.5, 147.0, 135.9 (d, $^3J_{CF}$ = 10.4 Hz), 135.7, 135.6, 128.8 (d, $^3J_{CF}$ = 3.9 Hz), 125.5 (d, $^4J_{CF}$ = 3.7 Hz), 125.3 (d, $^2J_{CF}$ = 12.9 Hz), 124.2, 116.8 (d, $^2J_{CF}$ = 24.8 Hz), 74.3, 68.4, 53.6. LRMS *m/z* (ESI+) 293.0 ($[M+H]^+$, 70%, 147.0 ($[M/2+H]^+$, 100% ). HRMS (ESI+) calcd. for $[C_{15}H_{15}{}^{35}ClFN_2O]^+$ 293.0851, found 293.0858 $[M+H]^+$. IR (film): $\nu_{max}$ 1677 (N–H), 1200, 1133, 905, 723 $cm^{-1}$.

3–((4–Chloro–2–fluorophenyl)(pyrrolidin–1–yl)methyl)pyridine, **MYOS_00191**

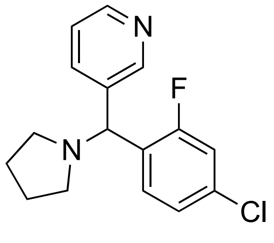

Prepared according to *General Synthetic Procedure B* using **Int2 (**200 mg, 1.0 equiv.), pyrrolidine (0.10 mL, 1.5 equiv.), anhydrous $Et_3N$ (0.22 mL, 2.0 equiv.) and a KI catalyst (13 mg, 10 mol%) to give the crude product (228 mg) which was purified by flash chromatography (5–90%, EtOAc in hexanes) to obtain a semi–pure material (185 mg). The product mixture was repurified by reverse phase chromatography (Biotage Sfar C18 30 g

column, 5–100%, MeOH in $H_2O$) to afford *the title compound* as a yellow viscous liquid (148 mg, 65%). $^1$H NMR (500 MHz, DMSO–$d_6$) δ 8.60 (d, $J$ = 2.2 Hz, 1H), 8.44 (dd, $J$ = 4.7, 1.6 Hz, 1H), 7.78 (app dt, $J$ = 7.9, 2.0 Hz, 1H), 7.74 (app t, $J$ = 8.2 Hz, 1H), 7.37 (d, $J$ = 2.1 Hz, 1H), 7.37 – 7.31 (m, 2H), 7.31 (dd, $J$ = 8.5, 2.0 Hz, 1H), 4.63 (s, 1H), 2.45 – 2.39 (m, 2H), 2.33 – 2.25 (m, 2H), 1.76 – 1.69 (m, 4H). $^{13}$C NMR (126 MHz, DMSO–$d_6$) δ 159.2 (d, $^1J_{CF}$ = 248.4 Hz), 148.8, 148.7, 137.7, 135.1, 132.4 (d, $^3J_{CF}$ = 10.9 Hz), 130.1 (d, $^3J_{CF}$ = 5.3 Hz), 129.2 (d, $^2J_{CF}$ = 12.6 Hz), 125.2 (d, $^4J_{CF}$ = 3.3 Hz), 123.9, 116.2 (d, $^2J_{CF}$ = 26.3 Hz), 63.9, 52.7, 23.2. LRMS *m/z* (ESI+) 291.0 ([M+H]$^+$, 100%). HRMS (ESI) calcd. for 291.1059 [$C_{16}H_{17}{}^{35}ClFN_2$]$^+$, found 291.1064. IR (film): $\nu_{max}$ 2967, 2791, 1580, 1479, 1423, 1222, 1125, 1099, 1025, 898, 711 cm$^{-1}$.

1–((4–Chloro–2–fluorophenyl)(pyridin–3–yl)methyl)piperidine–4–carbonitrile, **MYOS_00192**

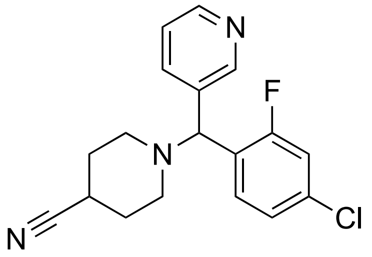

Prepared according to *General Synthetic Procedure B* using **Int2** (360 mg, 1.0 equiv.), piperidine–4–carbonitrile (0.20 mL, 1.3 equiv.), anhydrous $Et_3N$ (0.40 mL, 2.0 equiv.) and a KI catalyst (24 mg, 10 mol%) to give the crude product (534 mg) which was purified by normal phase flash chromatography (5–100%, EtOAc in hexanes) to obtain a semi–pure material (273 mg). The product mixture was repurified twice by reverse phase chromatography (Biotage Sfar C18 30 g column, 5–100%, MeOH in $H_2O$) to afford *the title compound* as a transparent oil (103 mg, 49%). $^1$H NMR (400 MHz, Chloroform–$d$) δ 8.62 (d, $J$ = 2.3 Hz, 1H), 8.48 (dd, $J$ = 4.8, 1.6 Hz, 1H), 7.66 (app dt, $J$ = 7.9, 2.0 Hz, 1H), 7.46 (app t, $J$ = 8.0 Hz, 1H), 7.23 (dd, $J$ = 7.9, 4.8 Hz, 1H), 7.13 (dd, $J$ = 8.5, 2.0 Hz, 1H), 7.04 (dd, $J$ = 9.9, 2.1 Hz, 1H), 4.73 (s, 1H), 2.85 – 2.51 (m, 3H), 2.47 – 2.16 (m, 2H), 2.10 – 1.80 (m, 4H). $^{13}$C NMR (101 MHz, Chloroform–$d$) δ 160.5 (d, $^1J_{CF}$ = 250.0 Hz), 149.7, 149.1, 136.5, 135.4, 134.1 (d, $^3J_{CF}$ = 10.6 Hz), 129.5 (d, $^3J_{CF}$ = 4.7 Hz), 126.9 (d, $^2J_{CF}$ = 12.8 Hz), 125.3 (d, $^4J_{CF}$ = 3.6 Hz), 123.8, 121.7, 116.8 (d, $^2J_{CF}$ = 26.0 Hz), 64.5, 49.9, 29.1, 26.4. LRMS *m/z* (ESI+) 330.1 ([M+H]$^+$, 100%). HRMS (ESI) calcd. for 330.1170 [$C_{18}H_{18}{}^{35}ClFN_3$]$^+$, found 330.1168. IR (film): $\nu_{max}$ 2959, 2810, 2765, 2240 (CN), 1576, 1479, 1338, 1222, 1092, 894, 711 cm$^{-1}$.

1–((4–Chloro–2–fluorophenyl)(pyridin–3–yl)methyl)piperidin–4–ol, **MYOS_00195**

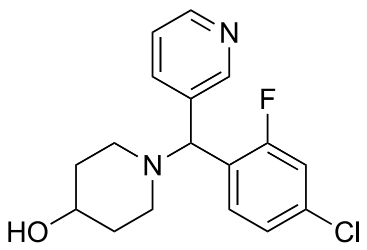

Prepared according to *General Synthetic Procedure B* using **Int2** (260 mg, 1.0 equiv.), piperidin–4–ol (154 mg, 1.5 equiv.), DMAP (13 mg, 10 mol%), anhydrous $Et_3N$ (0.28 mL, 2.0 equiv.) and a KI catalyst (17 mg, 10 mol%) to give the crude product (328 mg) which was purified by flash chromatography (5–85%, EtOAc in hexanes) to afford *the title compound* as a yellow oil (154 mg, 47%). $^1$H NMR (400 MHz, DMSO–$d_6$) δ 8.54 (d, obscured peak, 1H), 8.44 (d, $J$ = 4.8 Hz, 1H), 7.72 (app dt, obscured peak, $J$ = 8.0 Hz, 1H), 7.62 (app t, $J$ = 8.2 Hz, 1H), 7.46 – 7.23 (m, 3H), 4.72 (s, 1H), 4.56 (d, $J$ = 4.0 Hz, 1H), 3.53 – 3.39 (m, 1H), 2.74 – 2.52 (m, 2H), 2.20 – 1.85 (m, 2H), 1.79 – 1.56 (m, 2H), 1.59 – 1.16 (m, 2H). $^{13}$C NMR (101 MHz, DMSO–$d_6$) δ 159.8 (d, $^1J_{CF}$ = 248.7 Hz), 149.0, 148.6, 136.7, 135.2, 132.4 (d, $^3J_{CF}$ = 11.0 Hz), 130.3 (d, $^3J_{CF}$ = 5.0 Hz), 127.8 (d, $^2J_{CF}$ = 12.9 Hz), 125.1 (d, $^4J_{CF}$ = 3.4 Hz), 123.8, 116.2 (d, $^2J_{CF}$ = 26.2 Hz), 66.0, 63.9, 48.6, 34.3. LRMS *m/z* (ESI+) 321.1 ([M+H]$^+$, 100%). HRMS (ESI) calcd. for $[C_{17}H_{19}{}^{35}ClFN_2O]^+$ 321.1164, found 321.1167 [M+H]$^+$. IR (film): $\nu_{max}$ 3300 (O–H), 2980, 2800, 1500, 1100, 1040, 725 cm$^{-1}$.

1–((4–Chloro–2–fluorophenyl)(pyridin–3–yl)methyl)–4–(pyridin–3–yl)piperazine,

**MYOS_00196**[12]

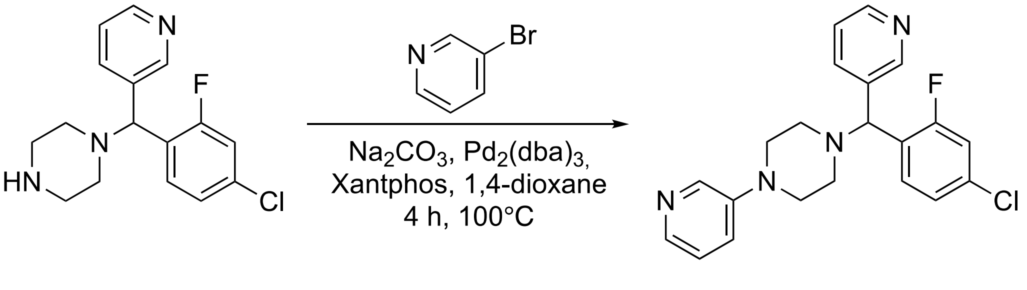


3–Bromopyridine (0.71 mL, 1.0 equiv.) was dissolved in 1,4–dioxane (6 mL, 122 mM) in a 25 mL round–bottomed flask. The solution was charged with amine **Int15** (269 mg, 1.2 equiv.), $Na_2CO_3$ (155 mg, 2.0 equiv.) and purged with Ar for 10 minutes. $Pd_2(dba)_3$ (34 mg, 0.05 equiv.) and Xantphos (43 mg, 0.10 equiv.) were added, the flask was fitted with a sealed reflux condenser, purged for additional 5 min with Ar and the stirring mixture was heated under reflux

in an oil bath for 4 h at 100°C. After completion (as indicated by TLC), the reaction was allowed to cool down to rt, quenched with water (8 mL) and the product was extracted with EtOAc (3 × 7 mL). The combined organic phases were washed with brine solution, dried with $MgSO_4$ and concentrated under reduced pressure to afford the crude material (270 mg) which was purified using Biotage Selekt column chromatography (1–15%, eluent DCM in MeOH) to give a pure fenarimol analogue as an orange oil (197 mg, 70%). $^1H$ NMR (400 MHz, Chloroform–*d*) δ 8.68 (d, $J$ = 2.2 Hz, 1H), 8.50 (dd, $J$ = 4.8, 1.6 Hz, 1H), 8.28 (app t, $J$ = 1.9 Hz, 1H), 8.10 (app t, $J$ = 3.0 Hz, 1H), 7.73 (app dt, $J$ = 8.0, 2.0 Hz, 1H), 7.56 (app t, $J$ = 8.0 Hz, 1H), 7.24 (d, $J$ = 5.0 Hz, 1H), 7.21 – 7.10 (m, 3H), 7.06 (dd, $J$ = 9.9, 2.1 Hz, 1H), 4.75 (s, 1H), 3.24 (t, $J$ = 5.0 Hz, 4H), 3.09 – 2.31 (m, 4H). $^{13}C$ NMR (101 MHz) δ 160.5 (d, $^1J_{CF}$ = 249.9 Hz), 149.9, 149.2, 146.9, 140.9, 138.5, 136.5, 135.6, 134.1 (d, $^3J_{CF}$ = 10.7 Hz), 129.6 (d, $^3J_{CF}$ = 4.7 Hz), 127.1 (d, $^2J_{CF}$ = 12.8 Hz), 125.3 (d, $^4J_{CF}$ = 3.6 Hz), 123.8, 123.6, 122.4, 116.8 (d, $^2J_{CF}$ = 25.9 Hz), 77.4, 51.5, 48.6. LRMS *m/z* (ESI+) 383.1 ($[M+H]^+$, 40%, 192.1 ($[M/2+H]^+$, 100% ). HRMS (ESI) calcd. for $[C_{21}H_{21}{}^{35}ClFN_4]^+$ 383.1433, found 383.1436 $[M+H]^+$. IR (film): $\nu_{max}$ 2828, 1733, 1595, 1483, 1427, 1244, 1107, 995, 898, 730, 682 $cm^{-1}$.

3–(4–((4–Chloro–2–fluorophenyl)(pyridin–3–yl)methyl)piperazin–1–yl)benzonitrile, **MYOS_00197**[13]

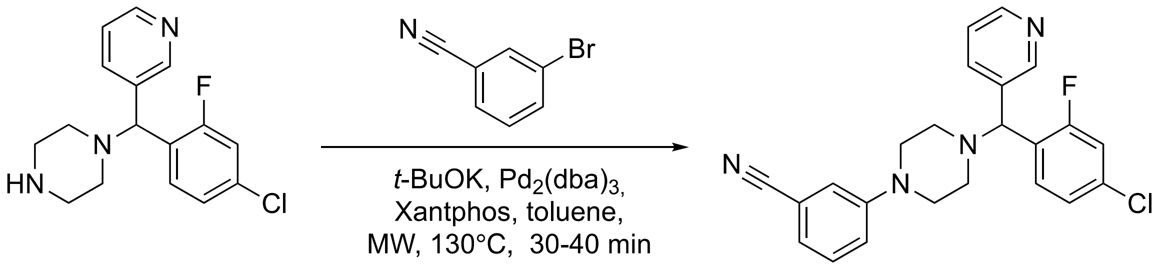


**Int15** (157 mg, 1.0 equiv.) and 3–bromobenzonitrile (94 mg, 1.0 equiv.) dissolved in anhydrous toluene (3.5 mL, 145 mM) were added to a 20 ml microwave vial. The mixture was charged with *t*–BuOK (87 mg, 1.5 equiv.), $Pd_2(dba)_3$ (10 mg, 0.020 equiv.) and Xantphos (18 mg, 0.060 equiv.). Reaction vial closed with a crimp seal was purged with Ar for 15 minutes and heated under microwave irradiation at 130ºC for 40 min. The mixture was cooled to rt and filtered through celite with EtOAc (8 mL). The extract was washed with brine solution, dried over

$MgSO_4$ and concentrated under reduced pressure. The crude material (203 mg) was purified with Biotage Selekt normal phase chromatography (1–10%, DCM in MeOH) to afford the pure product as a yellow resin (105 mg, 50%). $^1$H NMR (400 MHz, Chloroform–*d*) δ 8.61 (d, $J$ = 2.3 Hz, 1H), 8.43 (dd, $J$ = 4.8, 1.7 Hz, 1H), 7.66 (app dt, $J$ = 8.0, 2.0 Hz, 1H), 7.49 (app t, $J$ = 8.0 Hz, 1H), 7.28 – 7.20 (m, 1H), 7.18 (dd, $J$ = 4.8, 0.8 Hz, 1H), 7.11 – 7.07 (m, 1H), 7.05 – 6.98 (m, 3H), 6.98 (d, $J$ = 2.0 Hz, 1H), 4.67 (s, 1H), 3.15 (t, $J$ = 5.1 Hz, 4H), 2.61 – 2.40 (m, 4H). $^{13}$C NMR (101 MHz, Chloroform–*d*) δ 160.5 (d, $^1J_{CF}$ = 250.5 Hz), 151.3, 149.8, 149.2, 136.5, 135.6, 134.2 (d, $^3J_{CF}$ = 11.0 Hz), 130.0, 129.6 (d, $^3J_{CF}$ = 4.8 Hz), 125.3 (d, $^4J_{CF}$ = 3.4 Hz), 123.9, 122.8, 119.9, 119.4, 118.4, 116.8 (d, $^2J_{CF}$ = 26.1 Hz), 113.2, 64.5, 51.5, 48.5. LRMS *m/z* (ESI+) 407.0 ([M+H]$^+$, 100%). HRMS (ESI) calcd. for 407.1433 $[C_{23}H_{21}{}^{35}ClFN_4]^+$, found 407.1732. IR (film): $\nu_{max}$ 2825, 2228 (CN), 1733, 1595, 1483, 1244, 1107, 991, 898, 782, 711, 678 cm$^{-1}$.

Ethyl 1–((4–chloro–2–fluorophenyl)(pyridin–3–yl)methyl)piperidine–4–carboxylate, **MYOS_00203**

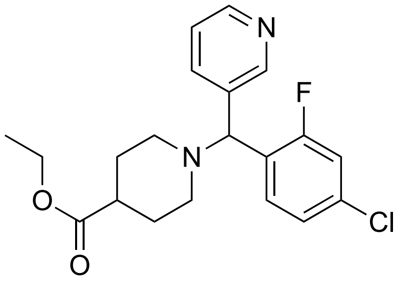


Prepared according to *General Synthetic Procedure B* using 3–(chloro(4–chloro–2–fluorophenyl)methyl)pyridine (**Int2**, 108 mg, 1.0 equiv.), ethyl piperidine–4–carboxylate (0.10 mL, 1.5 equiv.), $Et_3N$ (0.12 mL, 2.0 equiv.) and KI catalyst (4.0 mg, 0.05 equiv.) to give the crude product (437 mg) which was purified by flash chromatography (0–100%, EtOAc in hexanes) to afford *the title compound* as an amber oil (116 mg, 73%). $^1$H NMR (400 MHz, Chloroform–*d*) δ 8.60 (d, $J$ = 2.2 Hz, 1H), 8.46 (dd, $J$ = 4.8, 1.6 Hz, 1H), 7.68 (app dt, obscured peak, $J$ = 7.9 Hz, 1H), 7.50 (app t, $J$ = 8.0 Hz, 1H), 7.21 (dd, $J$ = 7.9, 4.8 Hz, 1H), 7.12 (dd, $J$ = 8.6, 2.1 Hz, 1H), 7.01 (dd, $J$ = 9.9, 2.1 Hz, 1H), 4.68 (s, 1H), 4.12 (q, $J$ = 7.1 Hz, 2H), 2.94 – 2.67 (m, 2H), 2.39 – 2.19 (m, 1H), 2.10 – 1.70 (m, 6H), 1.23 (t, $J$ = 7.1 Hz, 3H). $^{13}$C NMR (101 MHz, Chloroform–*d*) δ 175.1, 160.4 (d, $^1J_{CF}$ = 249.8 Hz), 149.8, 148.9, 137.0, 135.5, 133.7 (d, $^3J_{CF}$ = 10.7 Hz), 129.7 (d, $^3J_{CF}$ = 4.8 Hz), 127.5 (d, $^2J_{CF}$ = 13.0 Hz), 125.1 (d,

$^4J_{CF}$ = 3.6 Hz), 123.7, 116.6 (d, $^2J_{CF}$ = 25.9 Hz), 64.5, 60.5, 51.7, 51.1, 41.2, 28.5 (d, *J* = 9.4 Hz), 14.3. LRMS *m/z* (ESI+) 377.1 ([M+H]$^+$, 100%). HRMS (ESI+) calcd. for 377.1427 [$C_{20}H_{23}{}^{35}ClFN_2O_2$]$^+$, found 377.1427 [M+H]$^+$. IR (film): $\nu_{max}$ 2959, 2803, 1729 (C=O), 1580, 1483, 1170, 1047, 898, 715 cm$^{-1}$.

*N*–((4–Chloro–2–fluorophenyl)(pyridin–3–yl)methyl)benzo[*d*][1,3]dioxol–5–amine, **MYOS_00204**

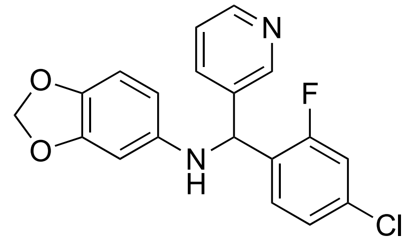


Prepared according to *General Synthetic Procedure B* using 3–(chloro(4–chloro–2–fluorophenyl)methyl)pyridine (**Int2**, 200 mg, 1.0 equiv.), 3,4–(methylenedioxy)aniline (161 mg, 1.5 equiv.), $Et_3N$ (0.22 mL, 2.0 equiv.) and KI catalyst (7.0 mg, 0.05 equiv.) to give the crude product (289 mg) which was purified by Biotage Selekt chromatography (0–85%, EtOAc in hexanes) to afford *the title compound* as an orange resin (223 mg, 80%). $^1$H NMR (400 MHz, DMSO–$d_6$) δ 8.58 (d, *J* = 2.3 Hz, 1H), 8.48 (dd, *J* = 4.8, 1.5 Hz, 1H), 7.71 (app dt, *J* = 7.9, 2.4 Hz, 1H), 7.48 (app t, *J* = 8.3 Hz, 1H), 7.44 – 7.35 (m, 2H), 7.31 (dd, *J* = 8.4, 2.1 Hz, 1H), 6.62 (d, *J* = 8.3 Hz, 1H), 6.39 (d, *J* = 2.2 Hz, 1H), 6.28 (d, *J* = 7.7 Hz, 1H), 6.03 (dd, *J* = 8.4, 2.2 Hz, 1H), 5.84 (d, *J* = 7.6 Hz, 1H), 5.82 (s, 2H). $^{13}$C NMR (101 MHz, DMSO–$d_6$) δ 158.6, 148.9, 148.5, 147.7, 142.7, 138.8, 136.9, 135.0, 132.7 (d, $^3J_{CF}$ = 10.6 Hz), 129.8 (d, $^3J_{CF}$ = 5.0 Hz), 128.4 (d, $^2J_{CF}$ = 14.0 Hz), 124.9 (d, $^4J_{CF}$ = 3.1 Hz), 123.6, 116.2 (d, $^2J_{CF}$ = 25.3 Hz), 108.4, 104.6, 100.1, 96.3, 52.9. LRMS *m/z* (ESI+) 357.0 ([M+H]$^+$, 100%). HRMS (ESI+) calcd. for 357.0801 [$C_{19}H_{15}{}^{35}ClFN_2O_2$]$^+$, found 357.0805 [M+H]$^+$. IR (film): $\nu_{max}$ 3265 (N–H), 2881, 1580, 1483, 1423, 1200, 1036, 894, 812, 708 cm$^{-1}$.

*N*–((4–Chloro–2–fluorophenyl)(pyridin–3–yl)methyl)–3–methylbutan–1–amine, **MYOS_00205**

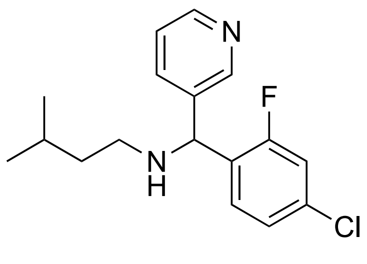


Prepared according to *General Synthetic Procedure B* using 3–(chloro(4–chloro–2–fluorophenyl)methyl)pyridine (**Int2**, 219 mg, 1.0 equiv.), isopentylamine (0.15 mL, 1.5 equiv.), $Et_3N$ (0.24 mL, 2.0 equiv.) and KI catalyst (7.0 mg, 0.05 equiv.) to give the crude product (253 mg) which was purified by Biotage Selekt chromatography (0–85%, EtOAc in hexanes) to afford *the title compound* as a yellow

oil (150 mg, 57%). $^1$H NMR (500 MHz, DMSO-$d_6$) δ 8.66 – 8.62 (m, 1H), 8.48 (d, $J$ = 5.7 Hz, 1H), 7.70 (app dt, $J$ = 8.0, 2.0 Hz, 1H), 7.54 (app t, $J$ = 8.2 Hz, 1H), 7.23 (dd, $J$ = 8.0, 5.1 Hz, 1H), 7.14 (dd, $J$ = 8.3, 2.2 Hz, 1H), 7.04 (dd, $J$ = 10.0, 2.1 Hz, 1H), 5.14 (s, 1H), 2.61 (br s, 1H), 2.63 – 2.51 (m, 2H), 1.64 (hept, $J$ = 13.3, 6.7 Hz, 1H), 1.42 (q, $J$ = 7.2 Hz, 2H), 0.86 (d, $J$ = 6.6 Hz, 6H). $^{13}$C NMR (101 MHz, DMSO–$d_6$) δ 159.6 (d, $^1J_{CF}$ = 248.1 Hz), 148.7 (d, $J$ = 1.1 Hz), 148.3, 138.4, 134.6, 132.2 (d, $^3J_{CF}$ = 10.7 Hz), 129.8 (d, $^2J_{CF}$ = 13.5 Hz), 129.4 (d, $^3J_{CF}$ = 5.3 Hz), 124.9 (d, $^4J_{CF}$ = 3.4 Hz), 123.5, 115.9 (d, $^2J_{CF}$ = 25.9 Hz), 56.7 (d, $J$ = 2.2 Hz), 45.5, 38.5, 25.4, 22.5 (d, $J$ = 3.1 Hz). LRMS *m/z* (ESI+) 307.1 ([M+H]$^+$, 100%). HRMS (ESI+) calcd. for 307.1372 [$C_{17}H_{21}{}^{35}ClFN_2$]$^+$, found 307.1375 [M+H]$^+$. IR (film): $\nu_{max}$ 2955, 2870, 1576, 1479, 1423, 1211, 1099, 1025, 894, 812, 711 cm$^{-1}$.

*N*–((4–Chloro–2–fluorophenyl)(pyridin–3–yl)methyl)–2–(pyrrolidin–1–yl)ethan–1–amine, **MYOS_00206**

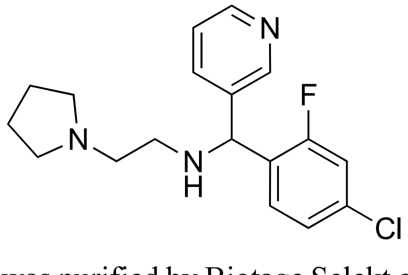


Prepared according to *General Synthetic Procedure B* using 3–(chloro(4–chloro–2–fluorophenyl)methyl)pyridine (**Int2**, 200 mg, 1.0 equiv.), 1–(2–Aminoethyl)pyrrolidine (0.15 mL, 1.5 equiv.), $Et_3N$ (0.22 mL, 2.0 equiv.) and KI catalyst (7.0 mg, 0.05 equiv.) to give the crude product (271 mg) which was purified by Biotage Selekt chromatography (0–100%, EtOAc in hexanes) to afford *the title compound* as an orange oil (225 mg, 86%). $^1$H NMR (500 MHz, Chloroform–$d$) δ 8.63 (d, $J$ = 2.2 Hz, 1H), 8.46 (dd, $J$ = 4.8, 1.7 Hz, 1H), 7.68 (app dt, $J$ = 7.9, 2.1 Hz, 1H), 7.52 (app t, $J$ = 8.1 Hz, 1H), 7.21 (dd, $J$ = 7.9, 4.8 Hz, 1H), 7.12 (dd, $J$ = 8.4, 2.1 Hz, 1H), 7.02 (dd, $J$ = 10.0, 2.1 Hz, 1H), 5.13 (s, 1H), 2.86 – 2.54 (m, 4H), 2.50 – 2.26 (m, 4H), 2.35 – 2.21 (m, 1H), 1.85 – 1.61 (m, 4H). $^{13}$C NMR (101 MHz, DMSO–$d_6$) δ 160.9, 148.7, 148.3, 138.2, 134.6, 132.3 (d, $^3J_{CF}$ = 10.8 Hz), 129.6 (d, $^2J_{CF}$ = 13.5 Hz), 129.5 (d, $^3J_{CF}$ = 5.1 Hz), 124.9 (d, $^4J_{CF}$ = 3.3 Hz), 123.6, 115.9 (d, $^2J_{CF}$ = 25.9 Hz), 56.7, 55.3, 53.6, 46.2, 23.1. LRMS *m/z* (ESI+) 334.0 ([M+H]$^+$, 100%). HRMS (ESI+) calcd. for 334.1481 [$C_{18}H_{22}{}^{35}ClFN_3$]$^+$, found 334.1486 [M+H]$^+$. IR (film): $\nu_{max}$ 3436 (N–H), 2251, 1654, 1051, 1025, 820, 760 cm$^{-1}$.

4–((((4–Chloro–2–fluorophenyl)(pyridin–3–yl)methyl)amino)methyl)phenol, **MYOS_00310**[14]

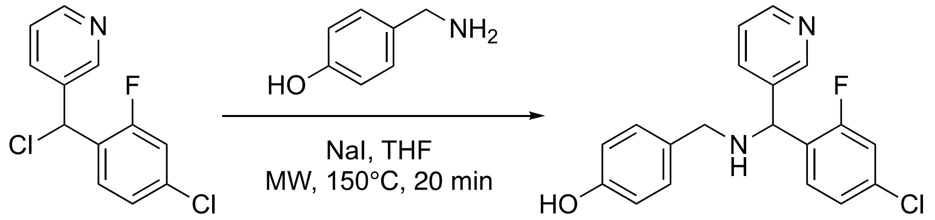


3–(Chloro(4–chloro–2–fluorophenyl)methyl)pyridine (**Int2**, 130 mg, 1.0 equiv.), 4–(aminomethyl)phenol (281 mg, 4.5 equiv.) and NaI (380 mg, 5.0 equiv.) were dissolved in dry THF (9 mL, 55 mM) and charged to a 20 mL microwave vial. The reaction vessel closed with a crimp seal was purged with Ar for 10 min and then heated under microwave irradiation at 150°C for 20 min. The crude mixture was cooled with pressurized air inside the reactor, concentrated *in vacuo* and forwarded to Biotage Selekt normal phase column chromatography purification (0–10%, DCM in MeOH) to afford the *title* organic product as a yellow liquid (146 mg, 84%). $^1$H NMR (400 MHz, DMSO–$d_6$) δ 9.25 (br s, 1H), 8.52 (d, $J$ = 2.2 Hz, 1H), 8.43 (dd, $J$ = 4.8, 1.6 Hz, 1H), 7.85 – 7.62 (m, 2H), 7.57 – 7.27 (m, 3H), 7.08 (d, $J$ = 8.3 Hz, 2H), 6.69 (d, $J$ = 8.4 Hz, 2H), 5.03 (d, $J$ = 6.2 Hz, 1H), 4.42 (s, 1H), 3.63 – 3.40 (m, 2H). $^{13}$C NMR (101 MHz, DMSO–$d_6$), weak signals, δ 156.2, 148.7, 148.3, 138.1, 134.7, 132.3, 130.1, 129.6, 129.2, 128.8, 125.0, 123.6, 116.0, 115.1, 114.9, 55.2, 50.1. LRMS *m/z* (ESI+) 343.1 ([M+H]$^+$, 100%). HRMS (ESI) calcd. for 343.1008 [$C_{19}H_{17}{}^{35}ClFN_2O$]$^+$, found 343.1013. IR (film): $\nu_{max}$ 3421, 2251, 2124, 1654, 1021, 823, 760 cm$^{-1}$.

1–(4–Chloro–2–fluorophenyl)–*N*–(4–methoxybenzyl)–1–(pyridin–3–yl)methanamine, **MYOS_00311**

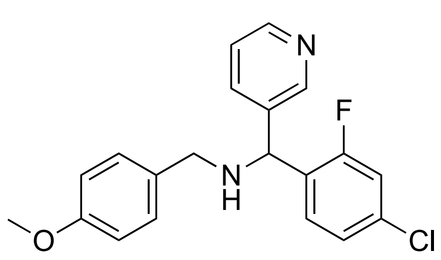


Prepared according to *General Synthetic Procedure B* (140 $^o$C, 20 min) using 3–(chloro(4–chloro–2–fluorophenyl)methyl)pyridine (**Int2**, 130 mg, 1.0 equiv.), 4–methoxybenzylamine (0.10 mL, 1.5 equiv.), anhydrous $Et_3N$ (0.14 mL, 2.0 equiv.) and a KI catalyst (4.5 mg, 0.05 equiv.) to give the crude product (201 mg) which was purified by flash chromatography (0–95%, EtOAc in hexanes) to afford *the title compound* as a yellow oil (149 mg, 82%). $^1$H NMR (400 MHz, Chloroform–$d$) δ 8.68 (d, $J$ = 2.3 Hz, 1H), 8.51 (dd, $J$ = 4.8, 1.7 Hz, 1H), 7.74 (app dt, $J$ = 7.9, 2.0 Hz, 1H), 7.60 (app

d, $J$ = 8.1 Hz, 1H), 7.28 – 7.21 (m, 3H), 7.22 – 7.13 (m, 1H), 7.07 (dd, $J$ = 10.0, 2.0 Hz, 1H), 6.96 – 6.79 (m, 2H), 5.19 (s, 1H), 3.83 (s, 3H), 3.76 – 3.62 (m, 2H), 2.05 – 1.87 (m, 1H). $^{13}$C NMR (101 MHz, Chloroform–*d*) δ 160.4 (d, $^{1}J_{CF}$ = 249.9 Hz), 159.0, 149.4, 149.0, 137.9, 135.0, 134.0 (d, $^{3}J_{CF}$ = 10.5 Hz), 131.7, 129.5, 129.1 (d, $^{3}J_{CF}$ = 5.1 Hz), 128.7 (d, $^{2}J_{CF}$ = 13.3 Hz), 125.1 (d, $^{4}J_{CF}$ = 3.5 Hz), 123.7, 116.6 (d, $^{2}J_{CF}$ = 25.4 Hz), 114.1, 56.7 (d, $J$ = 2.6 Hz), 55.4, 51.4. LRMS *m/z* (ESI+) 357.0 ([M+H]$^{+}$, 100%).

1–((4–Chloro–2–fluorophenyl)(pyridin–3–yl)methyl)indoline, **MYOS_00321**

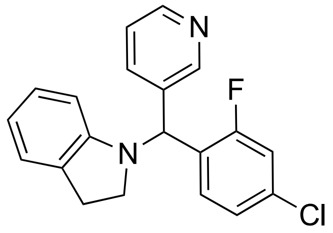


Prepared according to *General Synthetic Procedure B* using 3–(chloro(4–chloro–2–fluorophenyl)methyl)pyridine (**Int2**, 160 mg, 1.0 equiv.), indoline (112 mg, 1.5 equiv.), $Et_3N$ (0.17 mL, 2.0 equiv.) and KI catalyst (5.0 mg, 0.05 equiv.) to give the crude product (221 mg) which was purified by Biotage Selekt chromatography (0–100%, EtOAc in hexanes) to afford *the title compound* as a yellow viscous liquid (163 mg, 77%). $^{1}$H NMR (500 MHz, Chloroform–*d*) δ 8.61 (d, $J$ = 2.2 Hz, 1H), 8.56 (dd, $J$ = 4.9, 1.6 Hz, 1H), 7.72 (app dt, $J$ = 7.9, 2.0 Hz, 1H), 7.43 – 7.29 (m, 2H), 7.17 – 7.07 (m, 3H), 6.96 (app t, $J$= 7.7 Hz, 1H), 6.71 (app t, $J$ = 7.4 Hz, 1H), 6.16 (d, $J$ = 7.9 Hz, 1H), 5.79 (s, 1H), 3.18 (app t, $J$ = 8.6 Hz, 2H), 2.98 (app t, $J$ = 8.2 Hz, 2H). $^{13}$C NMR (126 MHz, Chloroform–*d*) δ 160.3 (d, $^{1}J_{CF}$ = 251.1 Hz), 151.0, 148.9, 148.1, 136.9, 136.3, 134.8 (d, $^{3}J_{CF}$ = 10.2 Hz), 130.6 (d, $^{3}J_{CF}$ = 4.6 Hz), 130.5, 127.4, 125.9 (d, $^{2}J_{CF}$ = 13.7 Hz), 125.1 (d, $^{4}J_{CF}$ = 3.6 Hz), 124.7, 124.0, 118.9, 117.0 (d, $^{2}J_{CF}$ = 24.9 Hz), 108.3, 57.9, 51.9, 28.5. LRMS *m/z* (ESI+) 338.9 ([M+H]$^{+}$, 100%). HRMS (ESI) calcd. for $[C_{20}H_{17}{}^{35}ClFN_2]^{+}$ 339.1059, found 339.1061 [M+H]$^{+}$. IR (film): $\nu_{max}$ 3026, 2844, 1736, 1602, 1479, 1405, 1244, 1025, 898, 745, 711 cm$^{-1}$.

(4-Bromo-2-fluorophenyl)(cyclohexyl)(pyridin-3-yl)methanol, **MYOS_00446**

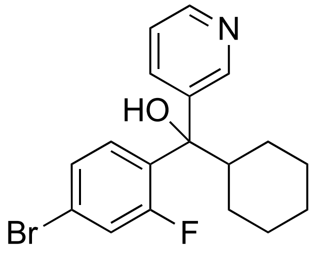


Prepared according to *General Synthetic Procedure C,* using cyclohexyl magnesium chloride (2 M in THF, 0.3 mL, 0.6 mmol) and intermediate **Int10** (124 mg, 0.443 mmol). The crude product was purified by column chromatography (hexane 20–40% in ethyl acetate) to give three fractions. Fraction A gave the starting ketone **Int10** (11 mg, 9.1%, $R_f$ = 0.5), fraction B gave *the title compound* as a straw-coloured oil (31

mg, 19%, $R_f$ = 0.3), and fraction C gave the starting ketone **Int10** reduced to alcohol (96 mg, 77%, $R_f$ = 0.1). $^1$H NMR (500 MHz, Acetone-$d_6$) $\delta$ 8.73 (s, 1H), 8.38 (d, $J$ = 4.7 Hz, 1H), 8.03 – 7.67 (m, 2H), 7.39 (dd, $J$ = 8.6, 2.0 Hz, 1H), 7.28 (dd, $J$ = 8.1, 4.7 Hz, 1H), 7.23 (dd, $J$ = 11.6, 2.0 Hz, 1H), 4.90 (s, 1H), 2.75 (tt, $J$ = 11.5, 2.7 Hz, 1H), 1.80 – 1.59 (m, 4H), 1.46 – 1.20 (m, 6H); $^{13}$C NMR (126 MHz, Acetone-$d_6$) $\delta$ 159.6 (d, $^1J_{CF}$ = 250.2 Hz), 148.9 (d, $^5J_{CF}$ = 3.3 Hz), 148.3, 141.3, 134.6 (d, $^4J_{CF}$ = 3.0 Hz), 134.2 (d, $^2J_{CF}$ = 11.6 Hz), 130.5 (d, $^3J_{CF}$ = 5.0 Hz), 128.1 (d, $^5J_{CF}$ = 3.4 Hz), 123.4, 121.2 (d, $^3J_{CF}$ = 10.0 Hz), 119.9 (d, $^2J_{CF}$ = 27.7 Hz), 78.4 (d, $^3J_{CF}$ = 3.9 Hz), 44.1 (d, $^4J_{CF}$ = 4.8 Hz), 28.0, 27.6, 27.2, 26.9, 26.9; $^{19}$F NMR (471 MHz, Acetone-$d_6$) $\delta$ –109.0; LRMS *m/z* (APCI) 366 ([M+H]$^+$, 100%) and 364 ([M+H]$^+$, 100%); HRMS (APCI) calcd. for $[C_{18}H_{20}{}^{81}BrFNO]^+$ 366.06863 and $[C_{18}H_{20}{}^{79}BrFNO]^+$ 364.07068 ([M+H]$^+$), found 366.06870 and 364.07075; IR (film) $n_{max}$ 3165 (br), 2931, 2852, 1598, 1568, 1477, 1206, 1089, 881 and 711 cm$^{-1}$.

(4-Chloro-2-fluorophenyl)(cyclohexyl)(pyridin-3-yl)methanol, **MYOS_00447**

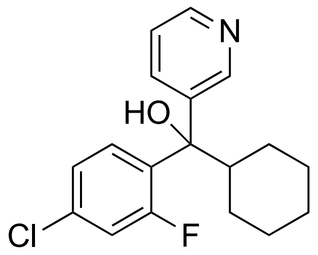


Prepared according to *General Synthetic Procedure C,* using cyclohexyl magnesium chloride (2 M in THF, 0.5 mL, 1.0 mmol) and intermediate **Int11** (124 mg, 0.526 mmol). The crude product was purified by column chromatography (hexane 20–40% in ethyl acetate) to give three fractions. Fraction A gave the starting ketone **Int11** (7 mg, 5.6%, $R_f$ = 0.5), fraction B gave *the title compound* as a straw-coloured oil (66 mg, 40%, $R_f$ = 0.3), and fraction C gave the starting ketone **Int11** reduced to alcohol (37 mg, 31%, $R_f$ = 0.1). $^1$H NMR (500 MHz, Acetone-$d_6$) $\delta$ 8.73 (s, 1H), 8.37 (s, 1H), 8.03 – 7.71 (m, 2H), 7.37 – 7.14 (m, 2H), 7.06 (d, $J$ = 11.8 Hz, 1H), 4.94 (s, 1H), 2.73 (t, $J$ = 11.5 Hz, 1H), 1.82 – 1.53 (m, 4H), 1.51 – 1.05 (m, 6H); $^{13}$C NMR (126 MHz, Acetone-$d_6$) $\delta$ 158.8 (d, $^1J_{CF}$ = 249.1 Hz), 148.1 (d, $^5J_{CF}$ = 3.3 Hz), 147.6, 140.8, 133.9 (d, $^4J_{CF}$ = 3.0 Hz), 133.1 (d, $^3J_{CF}$ = 10.9 Hz), 133.0 (d, $^2J_{CF}$ = 11.4 Hz), 129.5 (d, $^3J_{CF}$ = 5.4 Hz), 124.4, 122.8, 116.3 (d, $^2J_{CF}$ = 28.1 Hz), 77.6, 43.5 (d, $^4J_{CF}$ = 4.9 Hz), 27.3, 27.0, 26.5, 26.3, 26.2; $^{19}$F NMR (471 MHz, Acetone-$d_6$) $\delta$ –109.1; LRMS *m/z* (APCI) 322 ([M+H]$^+$, 33%) and 320 ([M+H]$^+$, 100%); HRMS (APCI) calcd. for $[C_{18}H_{20}{}^{37}ClFNO]^+$ 322.11825 and $[C_{18}H_{20}{}^{35}ClFNO]^+$ 320.12120 ([M+H]$^+$), found

322.11833 and 320.12128; IR (film) $n_{max}$ 3177 (br), 2931, 2853, 1603,1573, 1478, 1081, 893 and 711 cm$^{-1}$.

Cyclohexyl(phenyl)(pyridin-3-yl)methanol, **MYOS_00448**

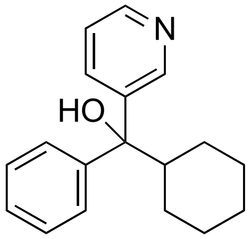


Prepared according to *General Synthetic Procedure C,* using cyclohexyl magnesium chloride (2 M in THF, 0.45 mL, 0.9 mmol) and phenyl(pyridin-3-yl)methanone (110 mg, 0.600 mmol). The crude product was purified by column chromatography (hexane 20–40% in ethyl acetate) to give two fractions. Fraction A gave the starting ketone (10 mg, 9.0%, $R_f$ = 0.5) and fraction B gave *the title compound* as a straw-coloured oil (64 mg, 40%, $R_f$ = 0.3). $^{1}$H NMR (500 MHz, Chloroform-*d*) $\delta$ 8.71 (s, 1H), 8.39 (d, $J$ = 4.8 Hz, 1H), 7.79 (dt, $J$ = 8.2, 2.1 Hz, 1H), 7.47 (d, $J$ = 7.7 Hz, 2H), 7.31 (t, $J$ = 7.7 Hz, 2H), 7.20 (dd, $J$ = 8.3, 5.6 Hz, 2H), 2.43 (tt, $J$ = 11.9, 2.9 Hz, 1H), 1.84 (s, 1H), 1.82 – 1.44 (m, 4H), 1.40 – 1.22 (m, 2H), 1.20 – 1.00 (m, 4H); $^{13}$C NMR (126 MHz, Chloroform-*d*) $\delta$ 147.8, 147.6, 145.7, 141.9, 133.9, 128.5, 126.9, 125.9, 123.1, 79.5, 45.7, 27.3, 27.2, 26.7, 26.5; LRMS *m/z* (APCI) 268 ([M+H]$^+$, 100%); HRMS (APCI) calcd. for $[C_{18}H_{22}NO]^+$ 268.16959 ([M+H]$^+$), found 268.16966; IR (film) $\nu_{max}$ 3124 (br), 2931, 2848, 1445, 1422, 906, 732 and 712 cm$^{-1}$.

Cyclohexyl(2-fluorophenyl)(pyridin-3-yl)methanol, **MYOS_00449**

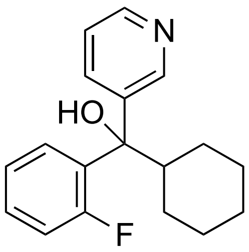


Prepared according to *General Synthetic Procedure C,* using cyclohexyl magnesium chloride (2 M in THF, 0.5 mL, 1.0 mmol) and intermediate **Int14** (124 mg, 0.616 mmol). The crude product was purified by column chromatography (hexane 20–40% in ethyl acetate) to give three fractions. Fraction A gave the starting ketone **Int14** (7 mg, 5.6%, $R_f$ = 0.5), fraction B gave *the title compound* as a straw-coloured oil (28 mg, 14%, $R_f$ = 0.3), and fraction C gave the starting ketone **Int14** reduced to alcohol (70 mg, 49%, $R_f$ = 0.1). $^{1}$H NMR (500 MHz, Acetone-*d*$_6$) $\delta$ 8.73 (s, 1H), 8.33 (d, $J$ = 4.6 Hz, 1H), 7.97 – 7.74 (m, 2H), 7.26 – 7.20 (m, 2H), 7.15 (q, $J$ = 8.8, 8.2 Hz, 1H), 6.94 (dd, $J$ = 12.6, 8.1 Hz, 1H), 4.58 (s, 1H), 2.80 – 2.62 (m, 1H), 1.80 – 1.54 (m, 4H), 1.41 – 1.16 (m, 6H); $^{13}$C NMR (126 MHz, Acetone-*d*$_6$) $\delta$ 159.1 (d, $^{1}J_{CF}$ = 244.9 Hz), 148.3 (d, $^{5}J_{CF}$ = 3.5 Hz), 147.5, 141.1, 133.7 (d, $^{3}J_{CF}$ = 3.1 Hz), 129.0 (d, $^{2}J_{CF}$ = 8.7 Hz), 128.0 (d, $^{3}J_{CF}$ = 4.5 Hz), 124.1 (d, $^{4}J_{CF}$ = 3.2 Hz), 122.6, 115.8 (d, $^{2}J_{CF}$ = 24.2 Hz), 77.7 (d, $^{3}J_{CF}$ = 11.9 Hz), 43.7 (d, $^{4}J_{CF}$ = 4.9 Hz), 27.3, 27.0, 26.5, 26.3, 26.3; $^{19}$F NMR (471 MHz,

Acetone-$d_6$) $\delta$ –112.1; LRMS $m/z$ (ESI) 308 ([M+Na]$^+$, 68%) and 286 ([M+H]$^+$, 100%); HRMS (ESI) calcd. for [$C_{18}H_{21}FNO$]$^+$ 286.16017 ([M+H]$^+$), found 286.16020; IR (film) $\nu_{max}$ 3181 (br), 2931, 2852, 1481, 1450, 1208, 757 and 712 cm$^{-1}$.

(4-Bromo-2-fluorophenyl)(cyclopentyl)(pyridin-3-yl)methanol, **MYOS_00450**

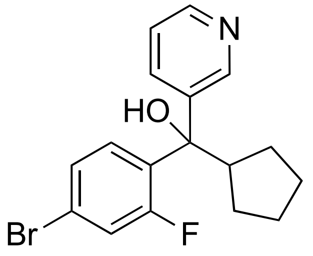


Prepared according to *General Synthetic Procedure C,* using cyclopentyl magnesium chloride (2 M in THF, 0.9 mL, 1.8 mmol) and intermediate **Int10** (180 mg, 0.642 mmol). The crude product was purified by column chromatography (hexane 20→40% in ethyl acetate) to give three fractions. Fraction A gave *the title compound* as a straw-coloured oil (14 mg, 6.5%, $R_f$ = 0.3) and fraction B gave the starting ketone **Int10** reduced to alcohol (114 mg, 63%, $R_f$ = 0.2). $^1$H NMR (500 MHz, Acetone-$d_6$) $\delta$ 8.71 (d, $J$ = 2.5 Hz, 1H), 8.38 (d, $J$ = 4.7 Hz, 1H), 8.03 – 7.72 (m, 2H), 7.41 (dd, $J$ = 8.5, 2.1 Hz, 1H), 7.27 (dd, $J$ = 8.1, 4.7 Hz, 1H), 7.20 (dd, $J$ = 11.5, 2.1 Hz, 1H), 4.91 (s, 1H), 3.40 (p, $J$ = 8.3 Hz, 1H), 1.77 – 1.38 (m, 8H); $^{13}$C NMR (126 MHz, Acetone-$d_6$) $\delta$ 160.1 (d, $^1J_{CF}$ = 251.1 Hz), 149.3 (d, $^5J_{CF}$ = 2.6 Hz), 148.7, 142.5, 135.4 (d, $^2J_{CF}$ = 11.2 Hz), 134.9 (d, $^4J_{CF}$ = 2.4 Hz), 130.3 (d, $^3J_{CF}$ = 4.9 Hz), 128.0 (d, $^5J_{CF}$ = 3.6 Hz), 123.5, 121.5 (d, $^3J_{CF}$ = 9.9 Hz), 120.1 (d, $^2J_{CF}$ = 27.1 Hz), 77.5 (d, $^3J_{CF}$ = 3.5 Hz), 45.8 (d, $^4J_{CF}$ = 3.9 Hz), 28.5, 28.0, 27.2, 27.1; $^{19}$F NMR (471 MHz, Acetone-$d_6$) $\delta$ –109.5; LRMS $m/z$ (ESI) 352 ([M+H]$^+$, 90%) and 350 ([M+H]$^+$, 100%); HRMS (ESI) calcd. for [$C_{17}H_{18}{}^{81}BrFNO$]$^+$ 352.05298 and [$C_{17}H_{18}{}^{79}BrFNO$]$^+$ 350.05503 ([M+H]$^+$), found 352.05276 and 350.05477; IR (film) $\nu_{max}$ 3192 (br), 2952, 2866, 1599, 1569, 1477, 1398, 855 and 711 cm$^{-1}$.

(4-Bromophenyl)(cyclopentyl)(pyridin-3-yl)methanol, **MYOS_00451**

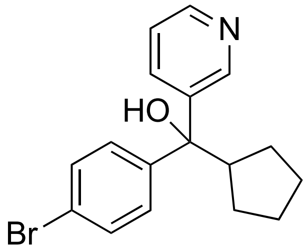


Prepared according to *General Synthetic Procedure C,* using cyclopentyl magnesium chloride (2 M in THF, 0.3 mL, 0.6 mmol) and intermediate **Int8** (105 mg, 0.401 mmol). The crude product was purified by column chromatography (hexane 20–40% in ethyl acetate) to give three fractions. Fraction A gave the starting ketone **Int8** (3.7 mg, 3.5%, $R_f$ = 0.5), fraction B gave *the title compound* as a straw-coloured oil (64 mg, 40%, $R_f$ = 0.3), and fraction C gave the starting ketone **Int8** reduced to alcohol (28 mg, 26%, $R_f$ = 0.1). $^1$H NMR (500 MHz, Acetone-$d_6$) $\delta$ 8.81 (s, 1H), 8.42 (s, 1H), 7.97 (dd, $J$ = 7.9, 2.5 Hz,

1H), 7.45 (d, $J$ = 8.3 Hz, 2H), 7.51 (d, $J$ = 8.4 Hz, 2H), 7.33 (dd, $J$ = 8.0, 4.7 Hz, 1H), 4.67 (s, 1H), 3.6 – 3.1 (m, 1H), 1.71 – 1.43 (m, 8H); $^{13}$C NMR (126 MHz, Acetone-$d_6$) $\delta$ 148.7, 148.4, 148.3, 144.1, 134.2, 131.9, 129.1, 123.7, 120.7, 78.7, 48.3, 28.0, 27.9, 27.2, 27.1; LRMS *m/z* (ESI) 334 ([M+H]$^+$, 90%) and 332 ([M+H]$^+$, 100%); HRMS (ESI) calcd. for [$C_{17}H_{19}{}^{81}BrNO$]$^+$ 334.06241 and [$C_{17}H_{19}{}^{79}BrNO$]$^+$ 332.06445 ([M+H]$^+$), found 334.06232 and 332.06437; IR (film) $\nu_{max}$ 3177 (br), 2951, 2866, 1485, 1418, 1008, 802 and 712 cm$^{-1}$.

(4-Chloro-2-fluorophenyl)(cyclopentyl)(pyridin-3-yl)methanol, **MYOS_00452**

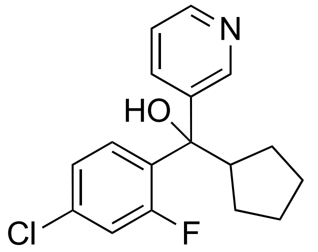


Prepared according to *General Synthetic Procedure C,* using cyclopentyl magnesium chloride (2 M in THF, 0.6 mL, 1.2 mmol) and intermediate **Int11** (182 mg, 0.772 mmol). The crude product was purified by column chromatography (hexane 20–40% in ethyl acetate) to give three fractions. Fraction A gave the starting ketone **Int11** (11 mg, 6.1%, $R_f$ = 0.5), fraction B gave *the title compound* as a straw-coloured oil (22 mg, 10%, $R_f$ = 0.3), and fraction C gave the starting ketone **Int11** reduced to alcohol (38 mg, 21%, $R_f$ = 0.1). $^1$H NMR (500 MHz, Acetone-$d_6$) $\delta$ 8.72 (s, 1H), 8.38 (d, $J$ = 4.7 Hz, 1H), 7.96 (t, $J$ = 8.6 Hz, 1H), 7.86 (d, $J$ = 7.3 Hz, 1H), 7.31 – 7.21 (m, 2H), 7.07 (dd, $J$ = 11.7, 2.1 Hz, 1H), 4.88 (s, 1H), 3.40 (p, $J$ = 8.1 Hz, 1H), 1.87 – 1.18 (m, 8H); $^{13}$C NMR (126 MHz, Acetone-$d_6$) $\delta$ 160.1 (d, $^1J_{CF}$ = 250.1 Hz), 149.3 (d, $^5J_{CF}$ = 2.6 Hz), 148.7, 142.6, 135.0 (d, $^4J_{CF}$ = 2.3 Hz), 134.0 (d, $^3J_{CF}$ = 10.6 Hz), 130.0 (d, $^2J_{CF}$ = 5.2 Hz), 125.0 (d, $^3J_{CF}$ = 3.5 Hz), 123.6, 117.2 (d, $^2J_{CF}$ = 27.4 Hz), 77.4, 45.9 (d, $^4J_{CF}$ = 4.0 Hz), 28.5, 28.1, 27.3, 27.1; $^{19}$F NMR (471 MHz, Acetone-$d_6$) $\delta$ –109.7; LRMS *m/z* (APCI) 308 ([M+H]$^+$, 31%) and 306 ([M+H]$^+$, 100%); HRMS (APCI) calcd. [$C_{17}H_{18}{}^{37}ClFNO$]$^+$ 308.10260 and [$C_{17}H_{18}{}^{35}ClFNO$]$^+$ 306.10555 ([M+H]$^+$), found 308.10266 and 306.10562; IR (film) $\nu_{max}$ 3159 (br), 2952, 2867, 1603, 1573, 1479,1402, 1078, 902 and 711 cm$^{-1}$.

Cyclopentyl(2-fluorophenyl)(pyridin-3-yl)methanol, **MYOS_00453**

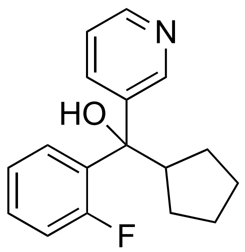


Prepared according to *General Synthetic Procedure C,* using cyclopentyl magnesium chloride (2 M in THF, 1.2 mL, 2.4 mmol) and intermediate **Int14** (163 mg, 0.810 mmol). The crude product was purified by column chromatography (hexane 20–40% in ethyl acetate) to give two fractions. Fraction A gave *the title compound* as a straw-coloured oil (12 mg, 5.0%,

$R_f$ = 0.3) and fraction B starting ketone **Int14** reduced to alcohol (107 mg, 66%, $R_f$ = 0.2). $^1$H NMR (500 MHz, Acetone-$d_6$) $\delta$ 8.73 (d, $J$ = 2.4 Hz, 1H), 8.54 – 8.19 (m, 1H), 7.94 (td, $J$ = 8.1, 1.9 Hz, 1H), 7.87 (d, $J$ = 8.2 Hz, 1H), 7.35 – 7.14 (m, 3H), 6.95 (dd, $J$ = 12.4, 8.0 Hz, 1H), 4.69 (s, 1H), 3.44 (p, $J$ = 8.2 Hz, 1H), 1.84 – 1.31 (m, 8H); $^{13}$C NMR (126 MHz, Acetone-$d_6$) $\delta$ 160.4 (d, $^1J_{CF}$ = 245.7 Hz), 149.4 (d, $^5J_{CF}$ = 2.6 Hz), 148.4, 143.1, 134.8 (d, $^3J_{CF}$ = 2.5 Hz), 129.9 (d, $^2J_{CF}$ = 8.7 Hz), 128.6 (d, $^3J_{CF}$ = 4.3 Hz), 124.8 (d, $^4J_{CF}$ = 3.2 Hz), 123.4, 116.7 (d, $^2J_{CF}$ = 23.7 Hz), 77.6, 46.2 (d, $^4J_{CF}$ = 3.9 Hz), 28.6, 28.1, 27.3, 27.1; $^{19}$F NMR (471 MHz, Acetone-$d_6$) $\delta$ –112.6; LRMS *m/z* (ESI) 272 ([M+H]$^+$, 100%); HRMS (ESI) calcd. for [$C_{17}H_{19}FNO$]$^+$ 272.14452 ([M+H]$^+$), found 272.14456; IR (film) $\nu_{max}$ 3205 (br), 2950, 2866, 1579, 1481, 1451, 1418, 1210, 1027, 756 and 712 cm$^{-1}$.

Cyclopentyl(phenyl)(pyridin-3-yl)methanol, **MYOS_00454**

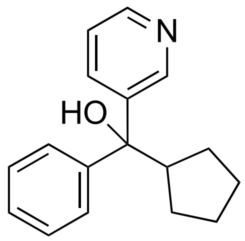

Prepared according to *General Synthetic Procedure C*, using cyclopentyl magnesium chloride (2 M in THF, 0.6 mL, 1.2 mmol) and phenyl(pyridin-3-yl)methanone (112 mg, 0.611 mmol). The crude product was purified by column chromatography (hexane 20–40% in ethyl acetate) to give three fractions. Fraction A gave the starting ketone (2.6 mg, 2.3%, $R_f$ = 0.5), fraction B gave *the title compound* as a straw-coloured oil (20 mg, 13%, $R_f$ = 0.3), and fraction C gave the starting ketone reduced to alcohol (21 mg, 19%, $R_f$ = 0.1). $^1$H NMR (500 MHz, Acetone-$d_6$) $\delta$ 8.9 (s, 1H), 8.5 (d, $J$ = 5.2 Hz, 1H), 8.4 (dd, $J$ = 8.1, 1.8 Hz, 1H), 7.8 – 7.6 (m, 3H), 7.3 (t, $J$ = 7.7 Hz, 2H), 7.2 (t, $J$ = 7.3 Hz, 2H), 5.1 (s, 1H), 1.8 – 1.3 (m, 8H); $^{13}$C NMR (126 MHz, Acetone-$d_6$) $\delta$ 147.5, 143.4, 143.3, 139.6, 129.0, 127.5, 126.8, 125.7, 78.7, 48.2, 27.8, 27.8, 27.0, 26.9; LRMS *m/z* (ESI) 254 ([M+H]$^+$, 100%); HRMS (ESI) calcd. for [$C_{17}H_{20}NO$]$^+$ 254.15394 ([M+H]$^+$), found 254.15378; IR (film) $\nu_{max}$ 3340 (br), 2951, 2866, 1446, 1173, 1028, 757 and 701 cm$^{-1}$.

(2-Fluorophenyl)(phenyl)(pyridin-3-yl)methanol, **MYOS_00455**

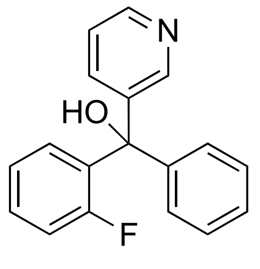

Prepared according to *General Synthetic Procedure C,* using phenylmagnesium bromide (1 M in THF, 1.8 mL, 1.8 mmol) and intermediate **Int14** (126 mg, 0.63 mmol). The crude mixture was purified by column chromatography (hexane 20–40% in ethyl acetate) to give two fractions. Fraction A gave the starting ketone **Int14** (19 mg, 15%, $R_f$ = 0.5) and fraction B gave *the title compound* as a straw-coloured oil (88 mg, 50%, $R_f$ = 0.3). $^1$H NMR (500 MHz, Acetone-$d_6$) $\delta$ 8.5 (d, $J$ = 2.4 Hz, 1H), 8.5 (dd, $J$ = 4.7, 1.5 Hz, 1H), 7.7 (dt, $J$ = 8.1, 2.1 Hz, 1H), 7.5 – 7.3 (m, 8H), 7.2 (td, $J$ = 7.6, 1.2 Hz, 1H), 7.1 (dd, $J$ = 11.7, 8.0 Hz, 1H), 5.6 (s, 1H); $^{13}$C NMR (126 MHz, Acetone-$d_6$) $\delta$ 161.0 (d, $^1J_{CF}$ = 247.2 Hz), 150.0 (d, $^5J_{CF}$ = 2.2 Hz), 149.1, 146.4, 142.8, 135.7 (d, $^5J_{CF}$ = 1.8 Hz), 134.8 (d, $^3J_{CF}$ = 11.2 Hz), 130.9 (d, $^2J_{CF}$ = 8.6 Hz), 130.1 (d, $^3J_{CF}$ = 3.4 Hz), 128.9, 128.4 (d, $^5J_{CF}$ = 1.7 Hz), 128.3, 124.9 (d, $^4J_{CF}$ = 3.5 Hz), 123.5, 117.0 (d, $^2J_{CF}$ = 22.8 Hz), 79.2; $^{19}$F NMR (471 MHz, Acetone-$d_6$) $\delta$ –107.4; LRMS *m/z* (ESI) 302 ([M+Na]$^+$, 83%) and 280 ([M+H]$^+$, 100%); HRMS (ESI) calcd. for [$C_{18}H_{15}FNO$]$^+$ 280.11322 ([M+H]$^+$), found 280.11325; IR (film) $\nu_{max}$ 3059 (br), 2866, 2779, 1579, 1481, 1448, 1022, 757 and 699 cm$^{-1}$.

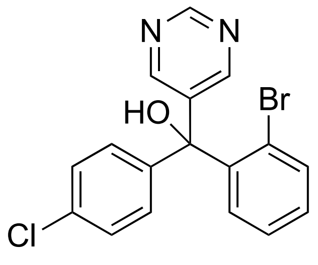

(2-Bromophenyl)(4-chlorophenyl)(pyrimidin-5-yl)methanol, **MYOS_00510**

Prepared according to *General Synthetic Procedure A*, using **Int12** (150 mg, 0.50 mmol) and 5-bromopyrimidine (0.56 µL, 0.50 mmol). The crude mixture was purified by column chromatography ($CH_2Cl_2$ 0–5% in methanol) to give two fractions. Fraction A gave the starting ketone **Int12** (46 mg, 31%, $R_f$ = 0.4) and fraction B gave *the title compound* as a straw-coloured oil (72 mg, 14%, $R_f$ = 0.3). $^1$H NMR (500 MHz, Acetone-$d_6$) $\delta$ 9.07 (d, $J$ = 1.2 Hz, 1H), 8.68 (d, $J$ = 1.2 Hz, 2H), 7.69 (d, $J$ = 7.8 Hz, 1H), 7.48 – 7.36 (m, 6H), 7.35 – 7.21 (m, 2H), 5.94 (s, 1H); $^{13}$C NMR (126 MHz, Acetone-$d_6$) $\delta$ 158.0, 157.1, 144.0, 143.9, 139.8, 136.4, 134.0, 131.7, 131.1, 130.6, 129.1, 128.4, 123.8, 80.3; LRMS *m/z* (ESI) 377 ([M–H]$^-$, 28%), 375 ([M–H]$^-$, 100%) and 373 ([M–H]$^-$, 69%); HRMS (ESI) calcd. for [$C_{17}H_{12}{}^{81}Br^{37}ClN_2ONa$]$^+$ 400.96638, [$C_{17}H_{12}{}^{79}Br^{37}ClN_2ONa$]$^+$ and [$C_{17}H_{12}{}^{81}Br^{35}ClN_2ONa$]$^+$ 398.96933, and [$C_{17}H_{12}{}^{79}Br^{35}ClN_2ONa$]$^+$ 396.97137 ([M+Na]$^+$), found 400.96641, 398.96935 and 396.97140; IR (film) $\nu_{max}$ 3212 (br), 2972, 1562, 1409, 1093, 1012, 823, 728 and 637 cm$^{-1}$.

(4-Bromophenyl)(4-chlorophenyl)(pyrimidin-5-yl)methanol, **MYOS_00511**

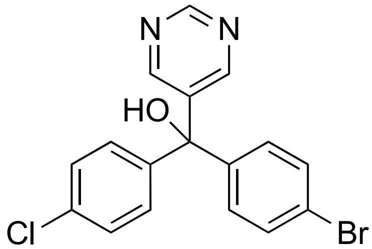

Prepared according to *General Synthetic Procedure A*, using **MYOS_00134** (150 mg, 0.50 mmol) and 5-bromopyrimidine (0.56 μL, 0.60 mmol). The crude mixture was purified by column chromatography ($CH_2Cl_2$ 0→5% in methanol) to give three fractions. Fraction A gave the starting ketone **MYOS_00134** (31 mg, 21%, $R_f$ = 0.4), fraction B gave *the title compound* as a straw-coloured oil (72 mg, 38%, $R_f$ = 0.3), and the starting ketone **MYOS_00134** reduced to alcohol (20 mg, 13%, $R_f$ = 0.2). $^1$H NMR (500 MHz, Chloroform-*d*) $\delta$ 9.01 (s, 1H), 8.60 (s, 2H), 7.50 – 7.45 (m, 2H), 7.35 – 7.30 (m, 2H), 7.18 – 7.15 (m, 2H), 7.13 – 7.09 (m, 2H), 4.15 (s, 1H); $^{13}$C NMR (126 MHz, Chloroform-*d*) $\delta$ 157.4, 156.1, 143.8, 143.2, 139.6, 134.6, 131.9, 129.4, 129.1, 122.8, 78.9; LRMS *m/z* (ESI) 377 ([M–H]$^-$, 30%), 375 ([M–H]$^-$, 100%) and 373 ([M–H]$^-$, 80%); HRMS (APCI) calcd. for $[C_{17}H_{12}{}^{81}Br^{37}ClIN_2O_3]^-$ 409.96755, $[C_{17}H_{12}{}^{79}Br^{37}ClN_2O_3]^-$ and $[C_{17}H_{12}{}^{81}Br^{35}ClN_2O_3]^-$ 407.97026, and $[C_{17}H_{12}{}^{79}Br^{35}ClN_2O_3]^-$ 405.97253 ([M+O$_2$]$^-$), found 409.96743, 407.97027 and 405.97246; IR (film) $\nu_{max}$ 3178 (br), 1568, 1487, 1408, 1037, 915 and 816 cm$^{-1}$.[5]

(4-Chlorophenyl)(pyrimidin-5-yl)(*p*-tolyl)methanol, **MYOS_00512**

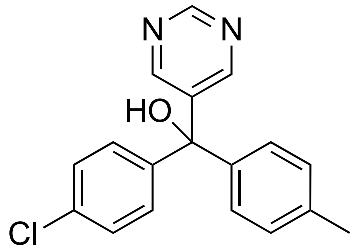

Prepared according to *General Synthetic Procedure A*, using **Int13** (115 mg, 0.50 mmol) and 5-bromopyrimidine (0.46 μL, 0.50 mmol). The crude mixture was purified by column chromatography ($CH_2Cl_2$ 0–5% in methanol) to give three fractions. Fraction A gave the starting ketone **Int13** (32 mg, 21%, $R_f$ = 0.4), fraction B gave *the title compound* as a straw-coloured oil (56 mg, 36%, $R_f$ = 0.3), and the starting ketone **Int13** reduced to alcohol (24 mg, 16%, $R_f$ = 0.2). $^1$H NMR (300 MHz, Acetone-*d$_6$*) $\delta$ 9.05 (s, 1H), 8.67 (s, 2H), 7.48 – 7.30 (m, 4H), 7.20 (s, 4H), 5.87 (s, 1H), 2.33 (s, 3H); $^{13}$C NMR (126 MHz, Acetone-*d$_6$*) $\delta$ 158.0, 156.9, 146.0, 143.6, 141.5, 138.2, 133.8, 130.4, 129.7, 129.0, 128.5, 79.3, 21.0; LRMS *m/z* (ESI) 311 ([M–H]$^-$, 34%), 309 ([M–H]$^-$, 100%); HRMS (ESI) calcd. for $[C_{18}H_{15}{}^{37}ClN_2O]^-$ 311.07706 and $[C_{18}H_{15}{}^{37}ClN_2O]^-$ 309.08001 ([M–H]$^-$), found 311.07734 and 309.08027; IR (film) $\nu_{max}$ 3179 (br), 2970, 1561, 1488, 1407, 1092, 1012, 812 and 724 cm$^{-1}$.[5]

(4-Bromo-2-fluorophenyl)(pyridin-3-yl)(pyrimidin-5-yl)methanol, **MYOS_00513**

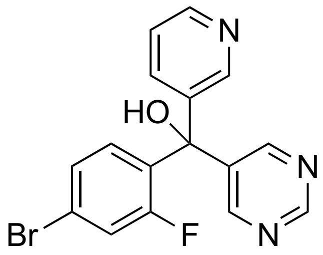

Prepared according to *General Synthetic Procedure A*, using **Int10** (140 mg, 0.50 mmol) and 5-bromopyrimidine (0.46 µL, 0.50 mmol). The crude mixture was purified by column chromatography ($CH_2Cl_2$ 0–5% in methanol) to give three fractions. Fraction A gave the starting ketone **Int10** (31 mg, 22%, $R_f = 0.4$), fraction B gave *the title compound* as a straw-coloured oil (42 mg, 26%, $R_f = 0.3$) and fraction C gave starting ketone **Int10** reduced to alcohol (20 mg, 14%, $R_f = 0.2$). $^1$H NMR (500 MHz, Chloroform-*d*) $\delta$ 9.15 (d, $J = 1.5$ Hz, 1H), 8.66 (d, $J = 1.4$ Hz, 2H), 8.53 (d, $J = 4.8$ Hz, 1H), 8.43 (s, 1H), 7.61 (dd, $J = 8.1, 2.0$ Hz, 1H), 7.31 (ddt, $J = 12.6, 7.4, 1.8$ Hz, 3H), 7.05 (td, $J = 8.5, 1.5$ Hz, 1H), 4.61 (s, 1H); $^{13}$C NMR (126 MHz, Chloroform-*d*) $\delta$ 159.7 (d, $^1J_{CF} = 251.1$ Hz), 158.2, 156.0 (d, $^5J_{CF} = 1.8$ Hz), 149.6, 148.6, 139.0, 137.8, 135.1, 130.6 (d, $^2J_{CF} = 10.8$ Hz), 130.1 (d, $^3J_{CF} = 3.5$ Hz), 128.2 (d, $^4J_{CF} = 3.5$ Hz), 123.8 (d, $^3J_{CF} = 9.8$ Hz), 123.6, 120.6 (d, $^2J_{CF} = 25.7$ Hz), 76.6; $^{19}$F NMR (471 MHz, Chloroform-*d*) $\delta$ –106.2; LRMS *m/z* (ESI) 360 ([M–H]$^-$, 100%), 358 ([M–H]$^-$, 90%); HRMS (ESI) calcd. for $[C_{16}H_{12}{}^{81}BrFN3O]^+$ 362.01218 and $[C_{16}H_{12}{}^{79}BrFN3O]^+$ 360.01423 ([M+H]$^+$), found 362.01255 and 360.01457; IR (film) $\nu_{max}$ 3065 (br), 2770, 1568, 1478, 1409, 1211, 809, 711 and 632 cm$^{-1}$.

(4-Bromophenyl)(pyridin-3-yl)(pyrimidin-5-yl)methanol, **MYOS_00514**

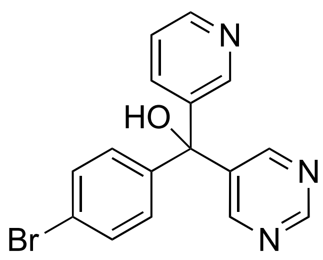

Prepared according to *General Synthetic Procedure A*, using **Int8** (132 mg, 0.50 mmol) and 5-bromopyrimidine (0.46 µL, 0.50 mmol). The crude mixture was purified by column chromatography ($CH_2Cl_2$ 0–5% in methanol) to give two fractions. Fraction A gave the starting ketone **Int8** (13 mg, 8.9%, $R_f = 0.4$) and fraction B gave *the title compound* as a straw-coloured oil (64 mg, 37%, $R_f = 0.3$). $^1$H NMR (500 MHz, Acetone-*d*$_6$) $\delta$ 9.09 (d, $J = 1.3$ Hz, 1H), 8.72 (d, $J = 1.3$ Hz, 2H), 8.61 – 8.44 (m, 2H), 7.75 (dd, $J = 8.1, 2.1$ Hz, 1H), 7.65 – 7.52 (m, 2H), 7.37 (ddd, $J = 18.7, 8.3, 3.2$ Hz, 3H), 6.39 (s, 1H); $^{13}$C NMR (126 MHz, Acetone-*d*$_6$) $\delta$ 158.3, 156.9, 149.8, 149.7, 145.5, 141.8, 140.5, 136.0, 132.3, 130.6, 124.0, 122.4, 78.3; LRMS *m/z* (ESI) 342 ([M–H]$^-$, 92%) and 340 ([M–H]$^-$, 100%); HRMS (ESI) calcd. for $[C_{16}H_{11}{}^{81}BrN_3O]^-$ 342.00705 and $[C_{16}H_{11}{}^{79}BrN_3O]^-$ 340.00910

([M–H]$^-$), found 342.00753 and 340.00959; IR (film) $\nu_{max}$ 3051 (br), 2779, 1567, 1478, 1404, 1205, 1049, 859, 809 and 711 cm$^{-1}$.

(4-Chloro-2-fluorophenyl)(pyridin-3-yl)(pyrimidin-5-yl)methanol, **MYOS_00515**

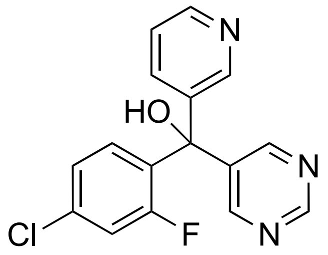


Prepared according to *General Synthetic Procedure A*, using **Int11** (115 mg, 0.49 mmol) and 5-bromopyrimidine (0.46 μL, 0.50 mmol). The crude mixture was purified by column chromatography ($CH_2Cl_2$ 0–5% in methanol) to give three fractions. Fraction A gave the starting ketone **Int11** (33 mg, 29%, $R_f$ = 0.4), fraction B gave *the title compound* as a straw-coloured oil (29 mg, 18%, $R_f$ = 0.3) and fraction C gave starting ketone **Int11** reduced to alcohol (24 mg, 21%, $R_f$ = 0.2). $^1$H NMR (500 MHz, Chloroform-*d*) δ 9.12 (d, *J* = 1.5 Hz, 1H), 8.65 (d, *J* = 1.5 Hz, 2H), 8.48 (d, *J* = 4.4 Hz, 1H), 8.38 (d, *J* = 2.4 Hz, 1H), 7.60 (dd, *J* = 8.1, 2.1 Hz, 1H), 7.29 (dd, *J* = 8.2, 4.9 Hz, 1H), 7.23 – 7.06 (m, 3H), 4.99 (s, 1H); $^{13}$C NMR (126 MHz, Chloroform-*d*) δ 159.7 (d, $^1J_{CF}$ = 250.2 Hz), 158.1, 156.0, 149.4, 148.5, 139.2, 137.9, 136.3 (d, $^3J_{CF}$ = 10.6 Hz), 135.2, 130.1 (d, $^3J_{CF}$ = 10.8 Hz), 129.7 (d, $^2J_{CF}$ = 3.7 Hz), 125.2 (d, $^4J_{CF}$ = 3.4 Hz), 123.6, 117.7 (d, $^2J_{CF}$ = 25.9 Hz), 76.4; $^{19}$F NMR (471 MHz, Chloroform-*d*) δ –106.1; LRMS *m/z* (ESI) 316 ([M–H]$^-$, 29%), 314 ([M–H]$^-$, 100%); HRMS calcd. for $[C_{16}H_{12}{}^{37}ClFN_3O]^-$ 316.04724 and $[C_{16}H_{12}{}^{35}ClFN_3O]^-$ 314.05019 ([M–H]$^-$), found 316.04726 and 314.05002; IR (film) $\nu_{max}$ 3062 (br), 2807, 1558, 1479, 1402, 1026, 906, 817, 713 and 630 cm$^{-1}$.

(2-Fluorophenyl)(pyridin-3-yl)(pyrimidin-5-yl)methanol, **MYOS_00516**

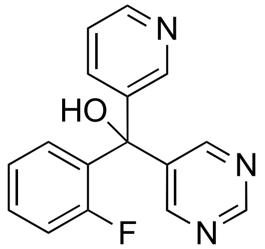


Prepared according to *General Synthetic Procedure A*, using **Int14** (101 mg, 0.50 mmol) and 5-bromopyrimidine (0.46 μL, 0.50 mmol). The crude mixture was purified by column chromatography ($CH_2Cl_2$ 0→5% in methanol) to give three fractions. Fraction A gave the starting ketone **Int14** (36 mg, 25%, $R_f$ = 0.4), fraction B gave *the title compound* as a straw-coloured oil (36 mg, 25%, $R_f$ = 0.3) and fraction C gave starting ketone **Int14** reduced to alcohol (24 mg, 21%, $R_f$ = 0.2). $^1$H NMR (500 MHz, Chloroform-*d*) δ 9.10 (d, *J* = 2.6 Hz, 1H), 8.65 (s, 3H), 8.58 – 8.27 (m, 4H), 7.63 (dd, *J* = 8.1, 2.2 Hz, 2H), 7.39 (dq, *J* = 7.8, 4.1 Hz, 2H), 7.28 (dd, *J* = 6.1, 3.0 Hz, 2H), 7.20 – 7.12 (m, 4H), 7.08 (dd, *J* = 11.9, 8.1 Hz, 2H), 5.30 (s, 1H); $^{13}$C NMR (126 MHz, Chloroform-*d*) δ 160.1 (d, $^1J_{CF}$ = 246.6 Hz), 157.8, 156.1, 149.1,

148.6, 139.7, 138.4, 135.3, 131.3 (d, $^{2}J_{CF}$ = 10.6 Hz), 131.1 (d, $^{3}J_{CF}$ = 8.8 Hz), 128.9, 124.7 (d, $^{3}J_{CF}$ = 3.4 Hz), 123.5, 116.9 (d, $^{2}J_{CF}$ = 22.3 Hz), 76.6; $^{19}$F NMR (471 MHz, Chloroform-*d*) $\delta$ –108.6; LRMS *m/z* (ESI) 280 ([M–H]$^{-}$, 100%); HRMS calcd. for $[C_{16}H_{11}FN_3O]^{-}$ 280.08916 ([M–H]$^{-}$), found 280.08914; IR (film) $\nu_{max}$ 3075 (br), 2797, 1578, 1403, 1207, 915, 822, 753, 714 and 631 cm$^{-1}$.

Ethyl 4-((2-fluoro-4-morpholinophenyl)(pyridin-3-yl)methyl)piperazine-1-carboxylate, **MYOS_00517**

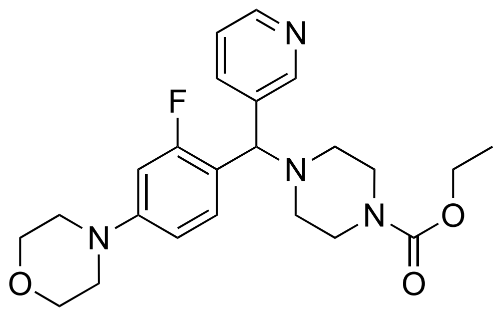

Prepared according to *General Synthetic Procedure E*, using **MYOS_00001** (184 mg, 0.49 mmol) and morpholine (0.45 mL, 0.58 mmol). The crude product was purified by column chromatography (hexane/ethyl acetate) to obtain *the title compound* as an orange-coloured gel (161 mg, 77%). $^{1}$H NMR (500 MHz, ) $\delta$ 8.63 (d, *J* = 2.3 Hz, 1H), 8.43 (dd, *J* = 4.7, 1.7 Hz, 1H), 7.81 (dt, *J* = 7.9, 2.0 Hz, 1H), 7.43 (t, *J* = 8.7 Hz, 1H), 7.30 (ddd, *J* = 7.9, 4.7, 0.9 Hz, 1H), 6.79 (dd, *J* = 8.7, 2.5 Hz, 1H), 6.62 (dd, *J* = 13.9, 2.5 Hz, 1H), 4.69 (s, 1H), 4.06 (q, *J* = 7.1 Hz, 2H), 3.95 – 3.64 (m, 5H), 3.47 (t, *J* = 5.1 Hz, 5H), 3.29 – 2.95 (m, 5H), 2.47 – 2.39 (m, 2H), 2.36 – 2.29 (m, 2H), 1.19 (t, *J* = 7.0 Hz, 3H); $^{13}$C NMR (126 MHz, Acetone-$d_6$) $\delta$ 162.3 (d, $^{1}J_{CF}$ = 242.4 Hz), 155.7, 153.2 (d, $^{3}J_{CF}$ = 10.3 Hz), 150.4, 149.3, 138.5, 136.0, 130.2 (d, $^{3}J_{CF}$ = 5.9 Hz), 124.3, 118.6 (d, $^{2}J_{CF}$ = 13.4 Hz), 112.0, 102.4 (d, $^{2}J_{CF}$ = 26.9 Hz), 67.2, 65.3, 61.5, 52.2, 49.1, 44.5, 15.0; $^{19}$F NMR (471 MHz, Acetone-$d_6$) $\delta$ –118.2; LRMS *m/z* (ESI) 451 ([M+Na]$^{+}$, 100%); HRMS calcd. for $[C_{23}H_{29}FN_4O_3Na]^{+}$ 451.21159 ([M+Na]$^{+}$), found 451.21201; IR (film) $\nu_{max}$ 2964, 2856, 2825, 1691 (s), 1624, 1427, 1236, 1119, 974, 830, 766 and 713 cm$^{-1}$.

1-(2-Fluorophenyl)-2-phenyl-1-(pyridin-3-yl)ethan-1-ol, **MYOS_00518**

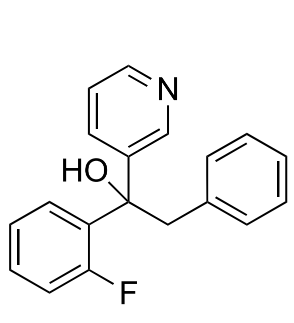

Prepared according to *General Synthetic Procedure C,* using benzylmagnesium chloride (1 M in THF, 1.5 mL, 1.5 mmol) and intermediate **Int14** (100 mg, 0.50 mmol). The crude mixture was purified by column chromatography (hexane/ethyl acetate) to give *the title compound* a light straw-coloured oil (62 mg, 44%) and starting ketone recovery (14 mg,

14%). $^{1}$H NMR (500 MHz, Acetone-$d_6$) $\delta$ 8.65 (d, $J$ = 2.8 Hz, 1H), 8.41 (d, $J$ = 4.7 Hz, 1H), 7.80 (dd, $J$ = 8.1, 2.1 Hz, 1H), 7.60 (td, $J$ = 8.1, 1.8 Hz, 1H), 7.27 (dd, $J$ = 8.0, 5.0 Hz, 2H), 7.12 – 6.96 (m, 7H), 5.07 (s, 1H), 3.77 (dd, $J$ = 91.6, 13.3 Hz, 2H); $^{13}$C NMR (126 MHz, Acetone-$d_6$) $\delta$ 160.2 (d, $^{1}J_{CF}$ = 245.2 Hz), 149.0 (d, $^{4}J_{CF}$ = 2.1 Hz), 148.8, 143.3, 137.7, 134.5 (d, $^{5}J_{CF}$ = 1.9 Hz), 133.8 (d, $^{2}J_{CF}$ = 11.1 Hz), 131.7, 130.1 (d, $^{3}J_{CF}$ = 8.7 Hz), 129.1 (d, $^{4}J_{CF}$ = 4.0 Hz), 128.2, 126.9, 124.6 (d, $^{3}J_{CF}$ = 3.4 Hz), 123.6, 116.4 (d, $^{2}J_{CF}$ = 23.0 Hz), 75.8, 45.8; $^{19}$F NMR (471 MHz, Acetone-$d_6$) $\delta$ –111.9; LRMS *m/z* (ESI) 292 ([M–H]$^-$, 100%); HRMS (ESI) calcd. for $[C_{19}H_{15}FNO]^-$ 292.11432 ([M–H]$^-$, found 292.11426; IR (film) $\nu_{max}$ 3168 (br), 2925, 1452, 1210, 1029, 819, 760 and 696 cm$^{-1}$.

Ethyl 4-((2-fluoro-4-(piperidin-1-yl)phenyl)(pyridin-3-yl)methyl)piperazine-1-carboxylate, **MYOS_00519**

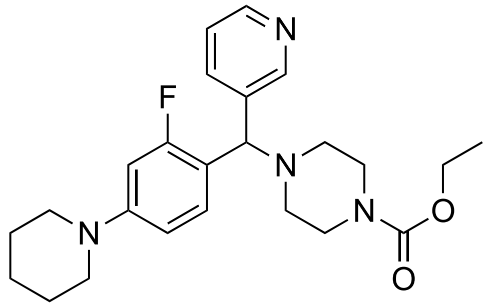


Prepared according to *General Synthetic Procedure E*, using **MYOS_00001** (188 mg, 0.50 mmol) and piperidine (0.60 mL, 0.60 mmol). The crude product was purified by column chromatography (hexane/ethyl acetate) to obtain *the title compound* as an orange-coloured gel (136 mg, 64%). $^{1}$H NMR (500 MHz, ) $\delta$ 8.63 (d, $J$ = 2.2 Hz, 1H), 8.42 (dd, $J$ = 4.8, 1.7 Hz, 1H), 7.81 (dt, $J$ = 7.9, 2.0 Hz, 1H), 7.37 (t, $J$ = 8.7 Hz, 1H), 7.32 – 7.28 (m, 1H), 6.76 (dd, $J$ = 8.8, 2.6 Hz, 1H), 6.58 (dd, $J$ = 14.3, 2.5 Hz, 1H), 4.67 (s, 1H), 4.06 (q, $J$ = 7.1 Hz, 2H), 3.47 (t, $J$ = 5.5 Hz, 5H), 3.27 – 3.09 (m, 4H), 2.42 (dt, $J$ = 11.0, 5.2 Hz, 3H), 2.32 (dt, $J$ = 11.0, 5.1 Hz, 2H), 1.66 – 1.51 (m, 6H), 1.19 (t, $J$ = 7.1 Hz, 3H); $^{13}$C NMR (126 MHz, Acetone-$d_6$) $\delta$ 162.3 (d, $^{1}J_{CF}$ = 242.2 Hz), 155.8, 153.6 (d, $^{3}J_{CF}$ = 10.2 Hz), 150.4, 149.2, 138.6, 136.0, 130.1 (d, $^{3}J_{CF}$ = 6.0 Hz), 124.4, 117.4 (d, $^{2}J_{CF}$ = 13.4 Hz), 112.6, 102.6 (d, $^{2}J_{CF}$ = 26.4 Hz), 65.3, 61.5, 52.2, 50.0, 44.5, 26.2, 24.9, 15.0; $^{19}$F NMR (471 MHz, Acetone-$d_6$) $\delta$ –118.4; LRMS *m/z* (ESI) 449 ([M+Na]$^+$, 100%); HRMS (ESI) calcd. for $[C_{24}H_{31}FN_4O_2Na]^+$ 449.23233 ([M+Na]$^+$), found 449.23163; IR (film) $\nu_{max}$ 2931, 2854, 2812, 1694 (s), 1623, 1425, 1238, 1118, 996, 764 and 712 cm$^{-1}$.

*tert*-Butyl 4-(4-((4-(ethoxycarbonyl)piperazin-1-yl)(pyridin-3-yl)methyl)-3-fluorophenyl)piperazine-1-carboxylate, **MYOS_00520**

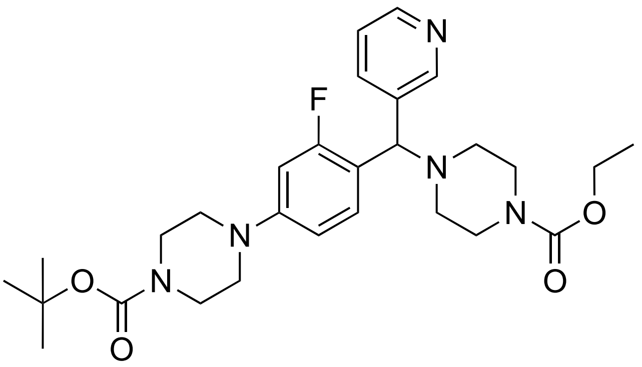

Prepared according to *General Synthetic Procedure E*, using **MYOS_00001** (173 mg, 0.46 mmol) and *tert*-butyl piperazine-1-carboxylate (102 mg, 0.55 mmol). The crude product was purified by column chromatography (hexane/ethyl acetate) to obtain *the title compound* as an orange-coloured gel (186 mg, 77%). $^{1}$H NMR (500 MHz, Acetone-$d_6$) $\delta$ 8.63 (d, $J$ = 2.2 Hz, 1H), 8.43 (dd, $J$ = 4.7, 1.7 Hz, 1H), 7.81 (app dt, $J$ = 7.9, 2.0 Hz, 1H), 7.43 (app t, $J$ = 8.7 Hz, 1H), 7.30 (ddd, $J$ = 7.9, 4.8, 0.9 Hz, 1H), 6.81 (dd, $J$ = 8.8, 2.5 Hz, 1H), 6.65 (dd, $J$ = 13.9, 2.5 Hz, 1H), 5.62 (s, 1H), 4.06 (q, $J$ = 7.1 Hz, 2H), 3.49 (dt, $J$ = 19.6, 5.3 Hz, 9H), 3.25 – 3.10 (m, 5H), 2.83 (s, 1H), 2.43 (dt, $J$ = 10.7, 5.2 Hz, 2H), 2.38 – 2.28 (m, 2H), 2.05 (p, $J$ = 2.2 Hz, 1H), 1.45 (s, 9H), 1.19 (t, $J$ = 7.1 Hz, 3H); $^{13}$C NMR (126 MHz, Acetone-$d_6$) $\delta$ 162.3 (d, $^{1}J_{CF}$ = 242.4 Hz), 155.7, 155.0, 153.0 (d, $^{3}J_{CF}$ = 10.4 Hz), 150.4, 149.3, 138.5, 136.0, 130.2 (d, $^{3}J_{CF}$ = 5.8 Hz), 124.3, 118.8 (d, $^{2}J_{CF}$ = 13.7 Hz), 112.8, 103.2 (d, $^{2}J_{CF}$ = 26.7 Hz), 79.8, 65.3, 61.5, 55.0, 52.2, 49.0, 44.5, 28.5, 15.0; $^{19}$F NMR (471 MHz, Acetone-$d_6$) $\delta$ –118.1; LRMS *m/z* (ESI) 550 ([M+Na]$^+$, 100%); HRMS (ESI) calcd. for [$C_{28}H_{38}FN_5O_4Na$]$^+$ 550.28000 ([M+Na]$^+$), found 550.27949; IR (film) $\nu_{max}$ 2975, 2859, 2824, 1690 (s), 1623, 1422, 1238, 1165, 1120, 996, 970 and 766 cm$^{-1}$.

### *Madurella mycetomatis* isolates

*M. mycetomatis* isolate MM55 was used to determine the $IC_{50}$ and $IC_{90}$ of each fenarimol analogue. To determine the minimal inhibitory concentration (MIC) of the fenarimol analogues, ten *M. mycetomatis* isolates with different genetic and geographical backgrounds were used [16]. All isolates were identified to the species level on the basis of morphology, and sequencing of the internally transcribed spacer (ITS) regions [16]. These fungal isolates were obtained from both the Mycetoma Research Center in Sudan and the Westerdijk Fungal Biodiversity Institute in the Netherlands and maintained in Erasmus Medical Centre, Rotterdam, The Netherlands.

### *In vitro* screening of fenarimol analogues

Screening of the fenarimol analogues on *M. mycetomatis* hyphal suspension was performed as previously described [1, 17, 18]. The screening procedure was performed in 96 well microplates

using 2,3-bis(2-methoxy-4-nitro-5-sulfophenyl)-5-[(phenylamino)carbonyl]-2H-tetrazolium hydroxide (XTT) to facilitate end-point reading [1, 17]. Initial screening was performed at 100 and 25 μM. Next, to establish the hit compounds that were most potent in inhibiting *M. mycetomatis* growth, the concentration of the compound at which a 50% reduction in growth was obtained ($IC_{50}$) was determined. For $IC_{50}$, fenarimol analogues that exhibit potency at 25 μM were selected and tested on *M. mycetomatis* isolate MM55 at 2-fold dilutions ranging from 16 μM to 0.03125 μM. The $IC_{50}$ was determined by plotting the growth percentage at fixed concentrations and determining the concentration at which 50% reduction of growth was obtained. Next, to determine if analogue activity was not isolate dependent, MICs for analogues with $IC_{50}$ value under 9 μM were determined on nine other *M. mycetomatis* isolates at concentrations ranging from 16 μM to 0.03125 μM. MIC was defined as the concentration at which 80% or more reduction in fungi metabolic activity was observed [1, 17]. The median MIC value of the ten *M. mycetomatis* isolates was defined as $MIC_{50}$. Metabolic activity was calculated using the formula $(E_{sample}-E_{nc})/(E_{gc}-E_{nc})*100\%$, where nc is the negative control and gc is the growth control.

**Toxicity in *Galleria mellonella* larvae**

Fenarimol analogues showing *in vitro* potency below 9 μM were considered for *in vivo* screening. First, to determine the toxicity of the fenarimol analogues *in vivo,* a single dose of each compound at a final concentration of 20 μM was injected into 15 larvae as described previously [1, 9]. As a control, we also injected a group of 15 larvae with distilled water alone. Survival of the larvae was then monitored over 10 days. A lack of toxicity was determined when no significant difference in larval survival between the treated and control group was observed.

***In vivo* Infection of *Galleria mellonella* larvae with fenarimol analogues**

Infection of *G. mellonella* with *M. mycetomatis* isolate MM55 was performed as previously described [1, 9, 19]. In short, *M. mycetomatis* mycelia was cultured for 2 weeks at 37 °C and then sonicated for 2 min at 28 μm. The resulting homogeneous suspension was washed once in PBS and then diluted with PBS to an inoculum size of 4 mg wet weight per larva. Inoculation was performed by injecting 40 μL of the fungal suspension into the last left pro-leg with an insulin 29G U-100 needle (BD diagnostics, Sparks, USA). Controls were injected with PBS. Fenarimol analogues showing no toxicity in *G. mellonella* larvae were used for treatment. Larvae were treated with fenarimol analogues at 4-, 28- and 52-hours post-infection at an end

concentration of 20 μM. To monitor the course of infection, larvae were checked daily for survival for 10 days. Infection experiments were performed in triplicates of 15 larvae.

**Statistical analysis**

To compare survival curves, the Log-rank test was performed with GraphPad Prism 7 (GraphPad Inc.).

**Method employed for computational modelling of Cyp51**

The sequence of eburicol 14-alpha-demethylase was obtained from Uniprot (https://www.uniprot.org/uniprotkb/A0A175W9Q1/entry). The sequence was used as input in AlphaFold 3, "Hem" was chosen as the ligand, and the complex was co-folded to obtain the protein-haem complex. This complex was used as input at the Protenix Server (https://protenix-server.com/) along with the SMILES of compound **4**. The resulting complex had plddt, ptm, and iptm values of 0.95, 0.93, and 0.95, respectively. The complex was downloaded and visualized through PyMol.

**NMR Data for Novel Compounds**

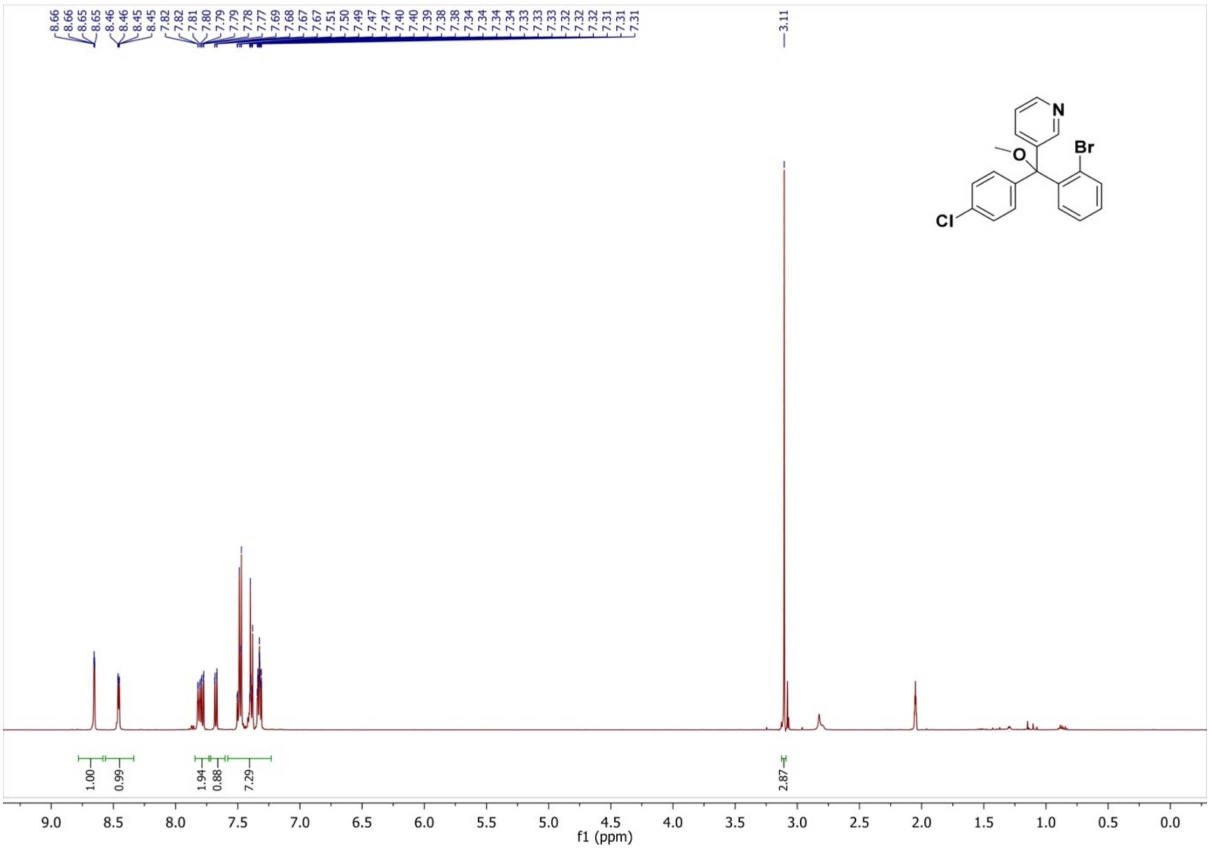

Figure S1. ¹H NMR spectrum of 3-((2-bromophenyl)(4-chlorophenyl)(methoxy)methyl)pyridine (**MYOS_00009**, 500 MHz, Acetone-$d_6$).

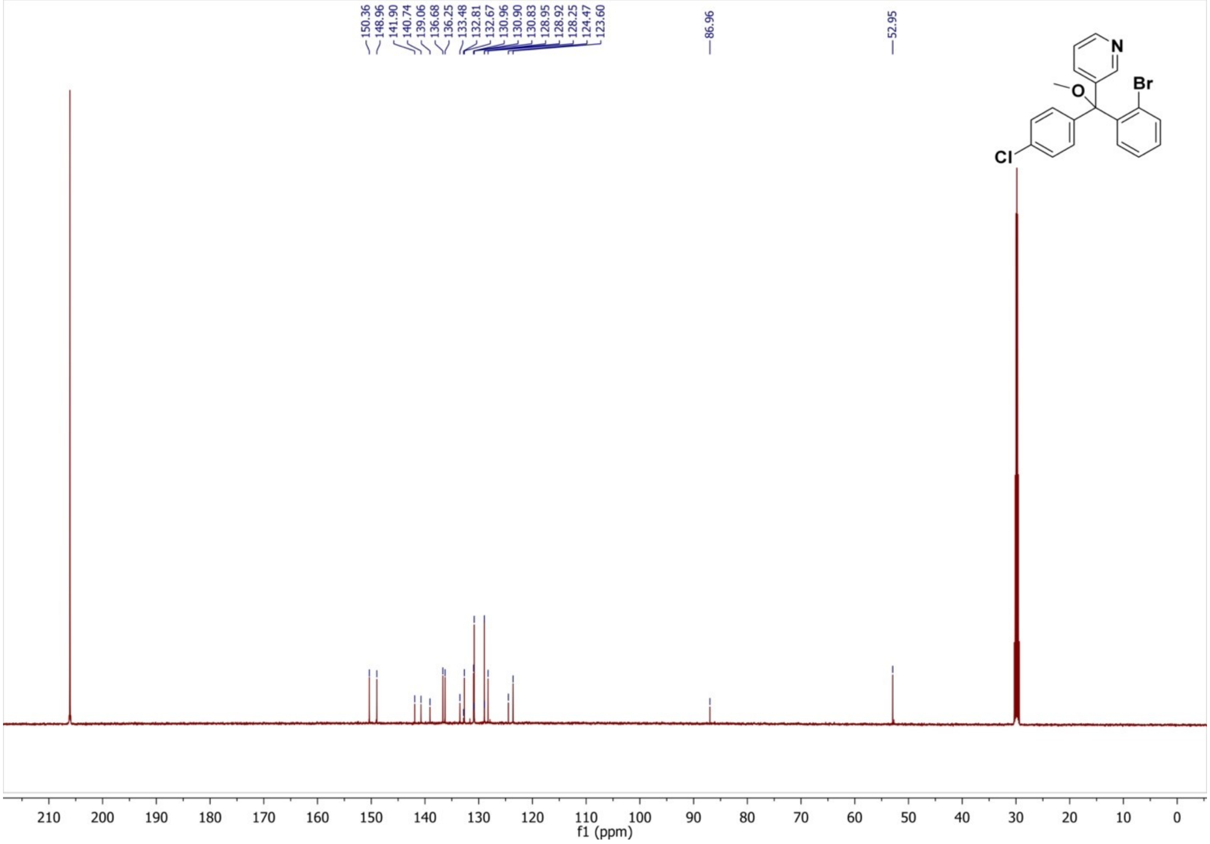

Figure S2. ¹³C NMR spectrum of 3-((2-bromophenyl)(4-chlorophenyl)(methoxy)methyl)pyridine (**MYOS_00009**, 125 MHz, Acetone-$d_6$).

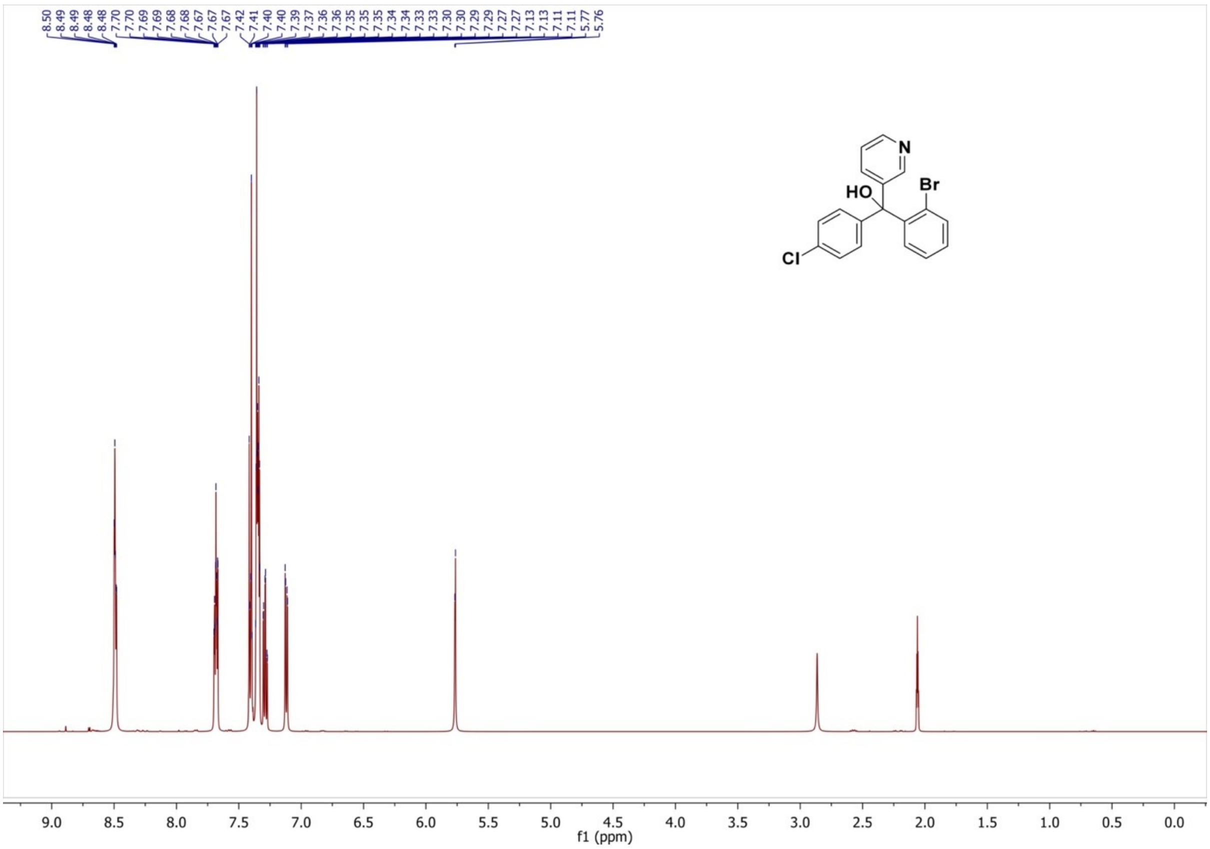

Figure S3. $^{1}$H NMR spectrum of (2-bromophenyl)(4-chlorophenyl)(pyridin-3-yl)methanol (**MYOS_00012**, 500 MHz, Acetone-$d_6$).

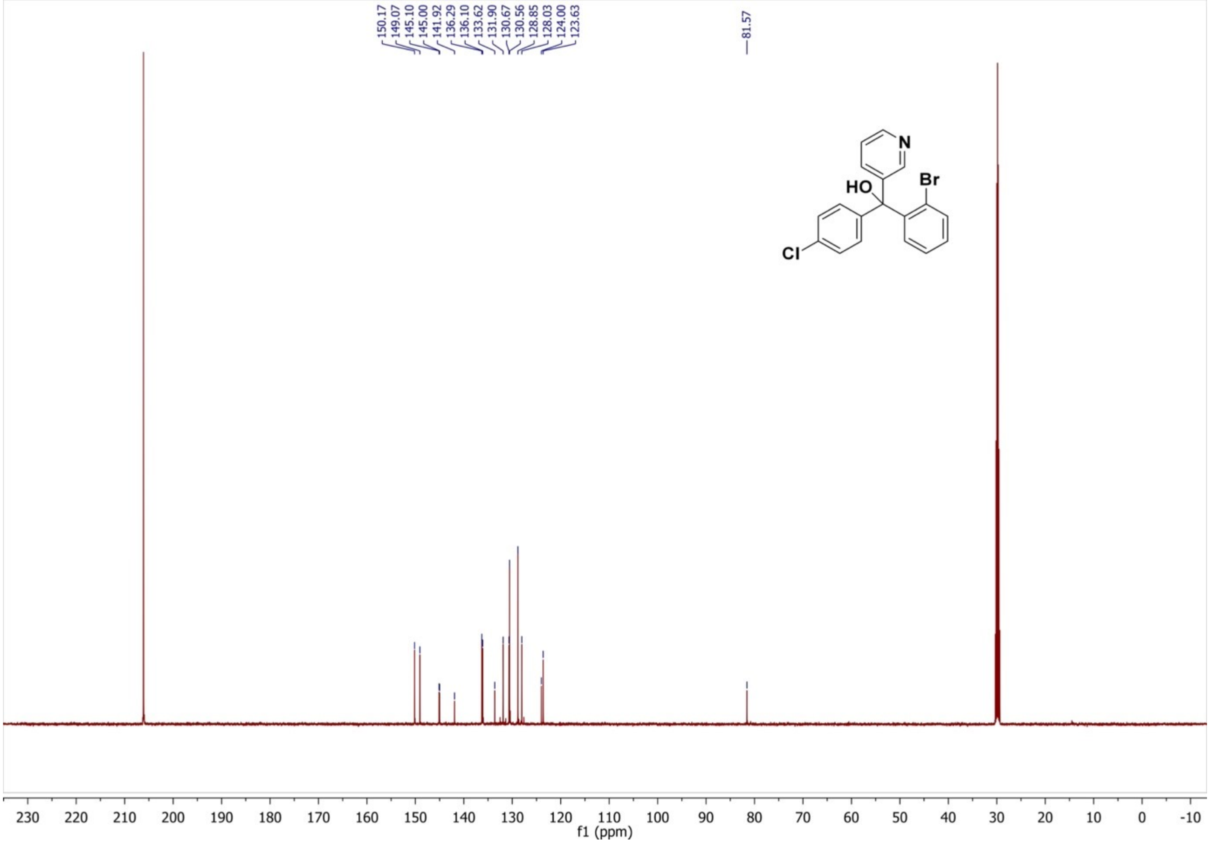

Figure S4. $^{13}$C NMR spectrum of (2-bromophenyl)(4-chlorophenyl)(pyridin-3-yl)methanol (**MYOS_00012**, 125 MHz, Acetone-$d_6$).

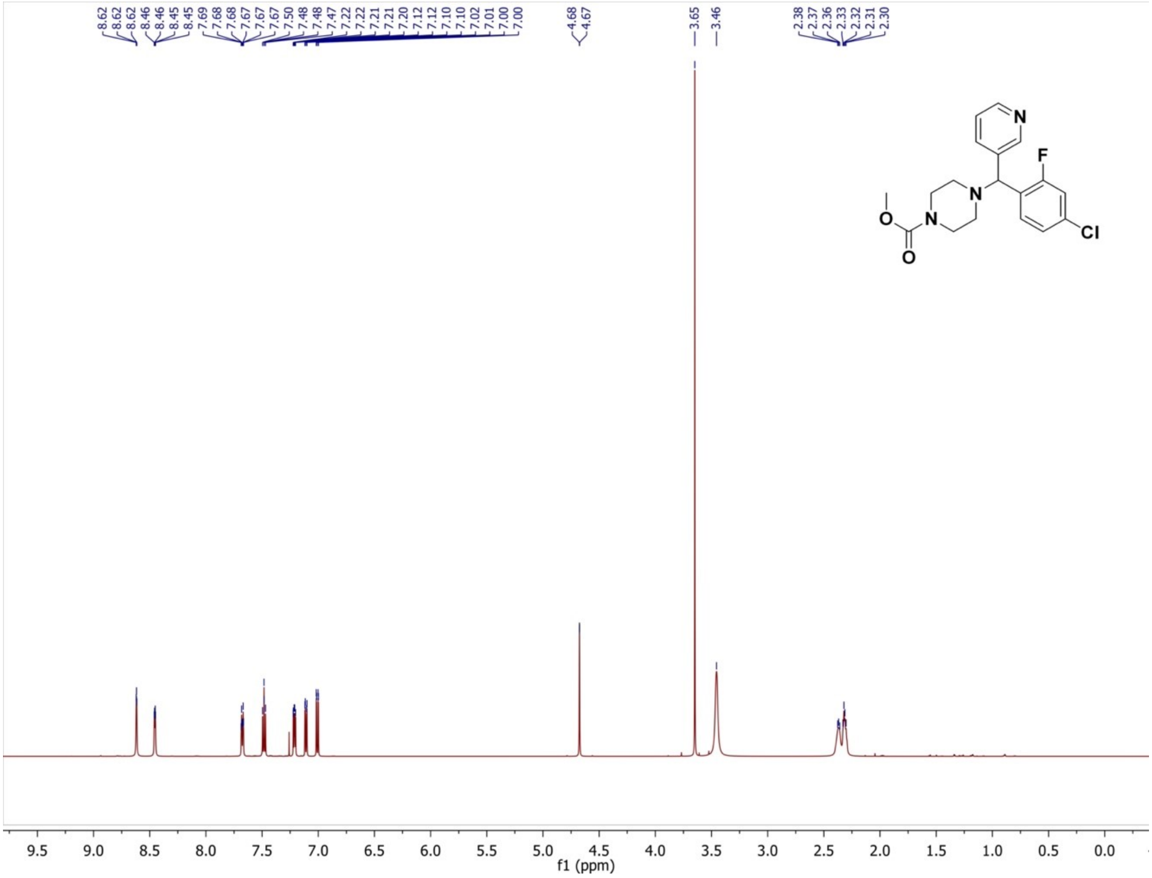


Figure S5. $^{1}$H NMR spectrum of methyl 4-((4-chloro-2-fluorophenyl)(pyridin-3-yl)methyl)piperazine-1-carboxylate (**MYOS_00015**, 600 MHz, $CDCl_3$).

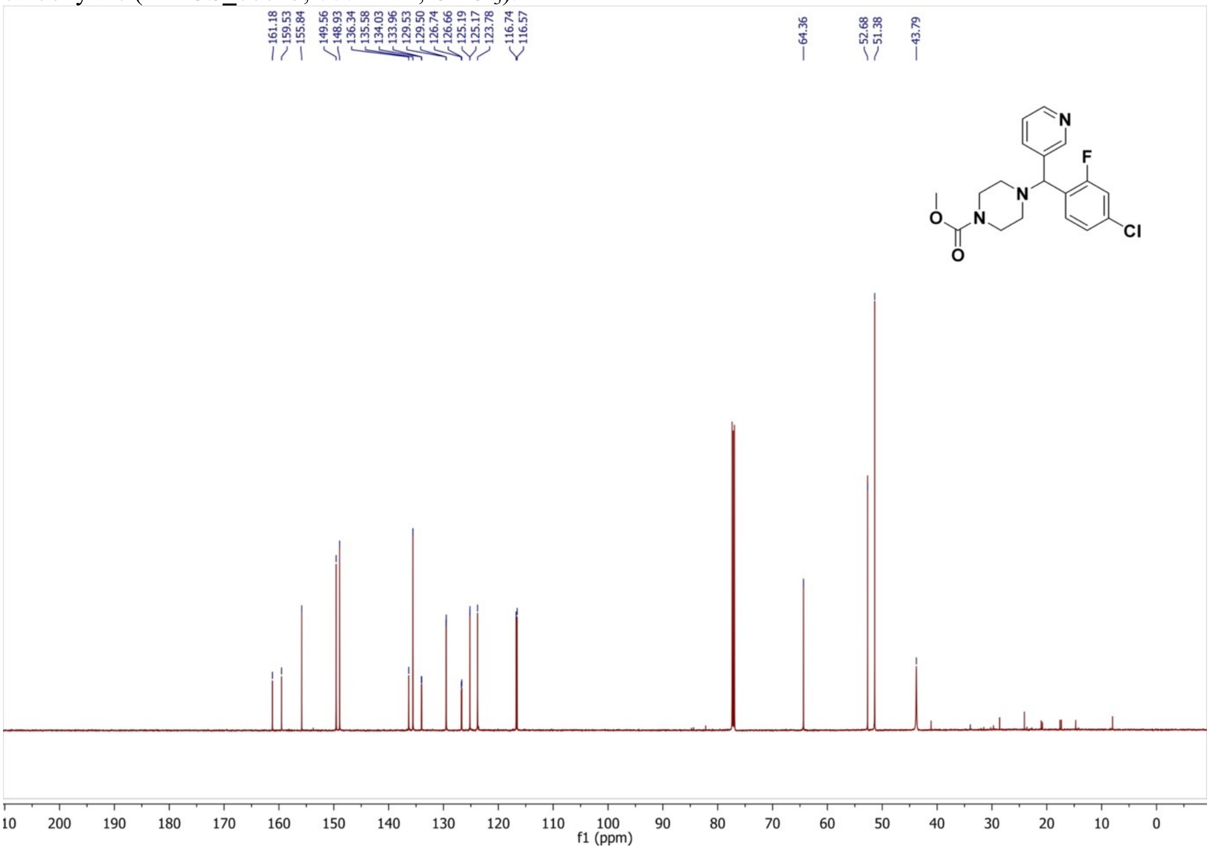


Figure S6. $^{13}$C NMR spectrum of methyl 4-((4-chloro-2-fluorophenyl)(pyridin-3-yl)methyl)piperazine-1-carboxylate (**MYOS_00015**, 151 MHz, $CDCl_3$).

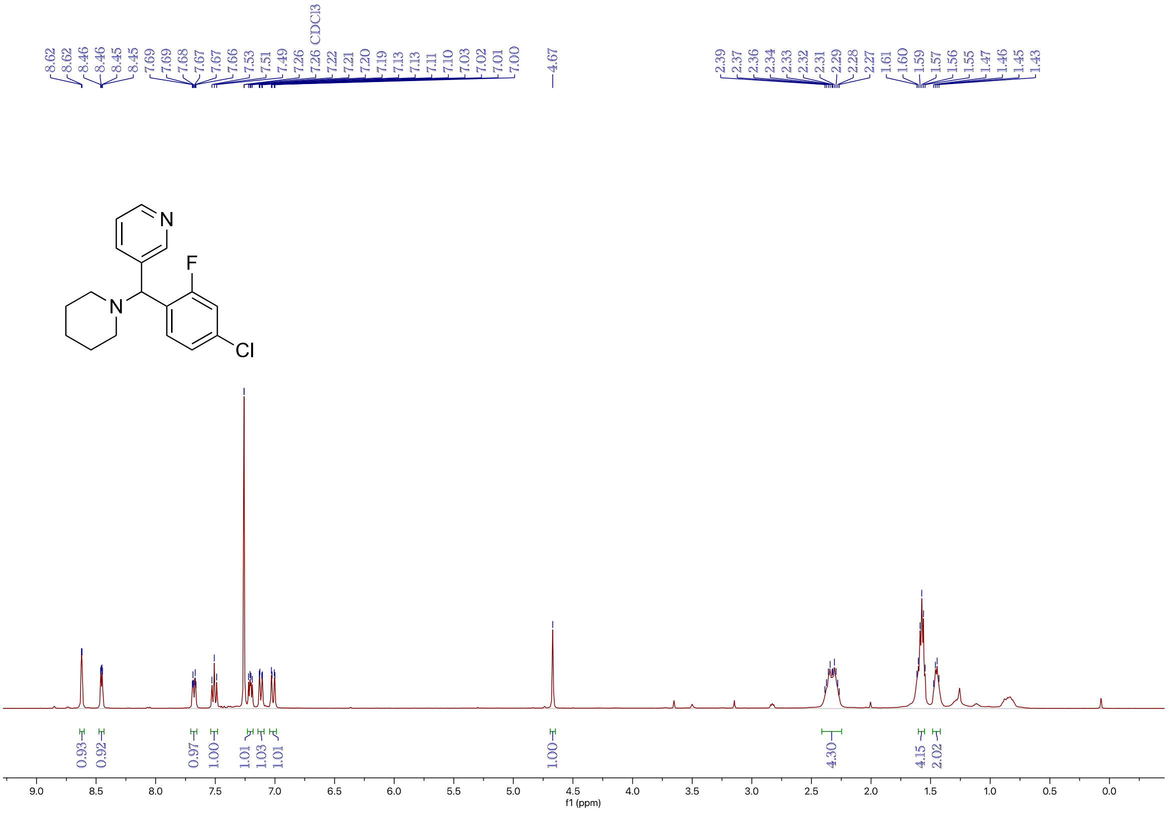


Figure S7. $^{1}H$ NMR spectrum of 3-((4-chloro-2-fluorophenyl)(piperidin-1-yl)methyl)pyridine (**MYOS_00016**, 400 MHz, $CDCl_3$).

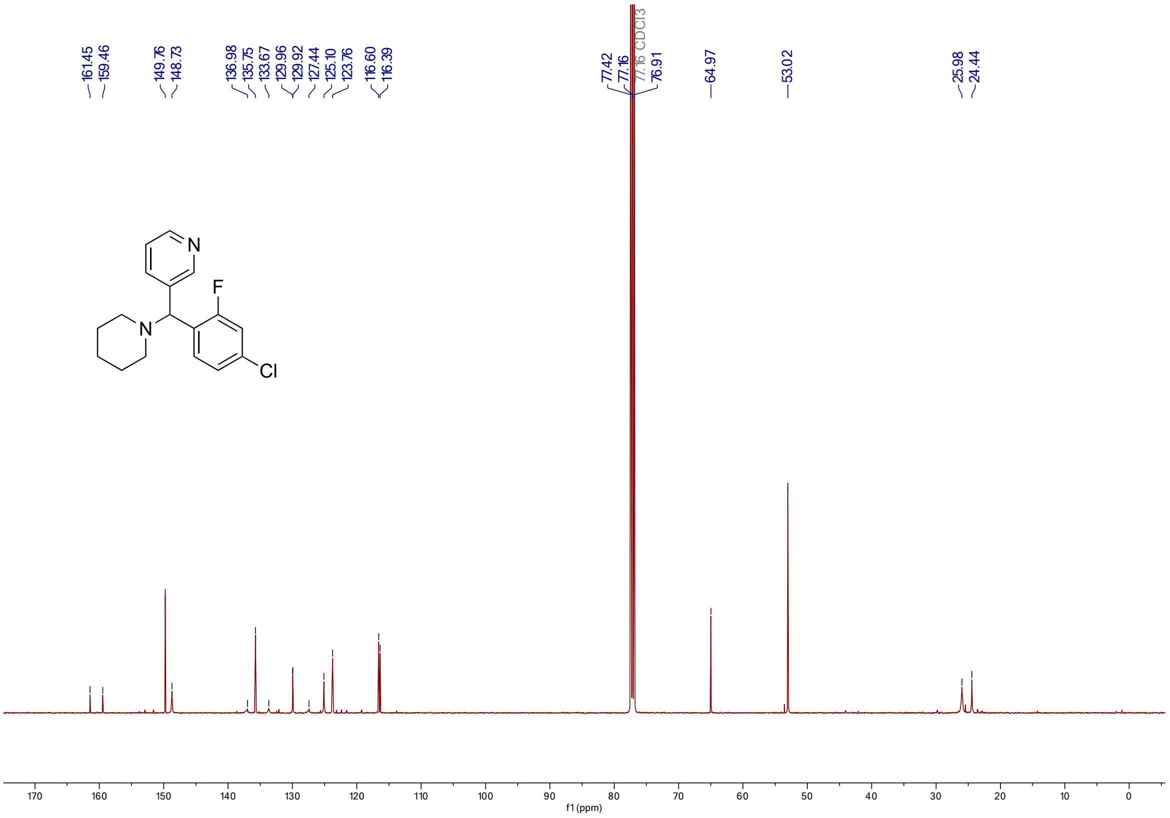


Figure S8. $^{13}C$ NMR spectrum of 3-((4-chloro-2-fluorophenyl)(piperidin-1-yl)methyl)pyridine (**MYOS_00016**, 126 MHz, $CDCl_3$).

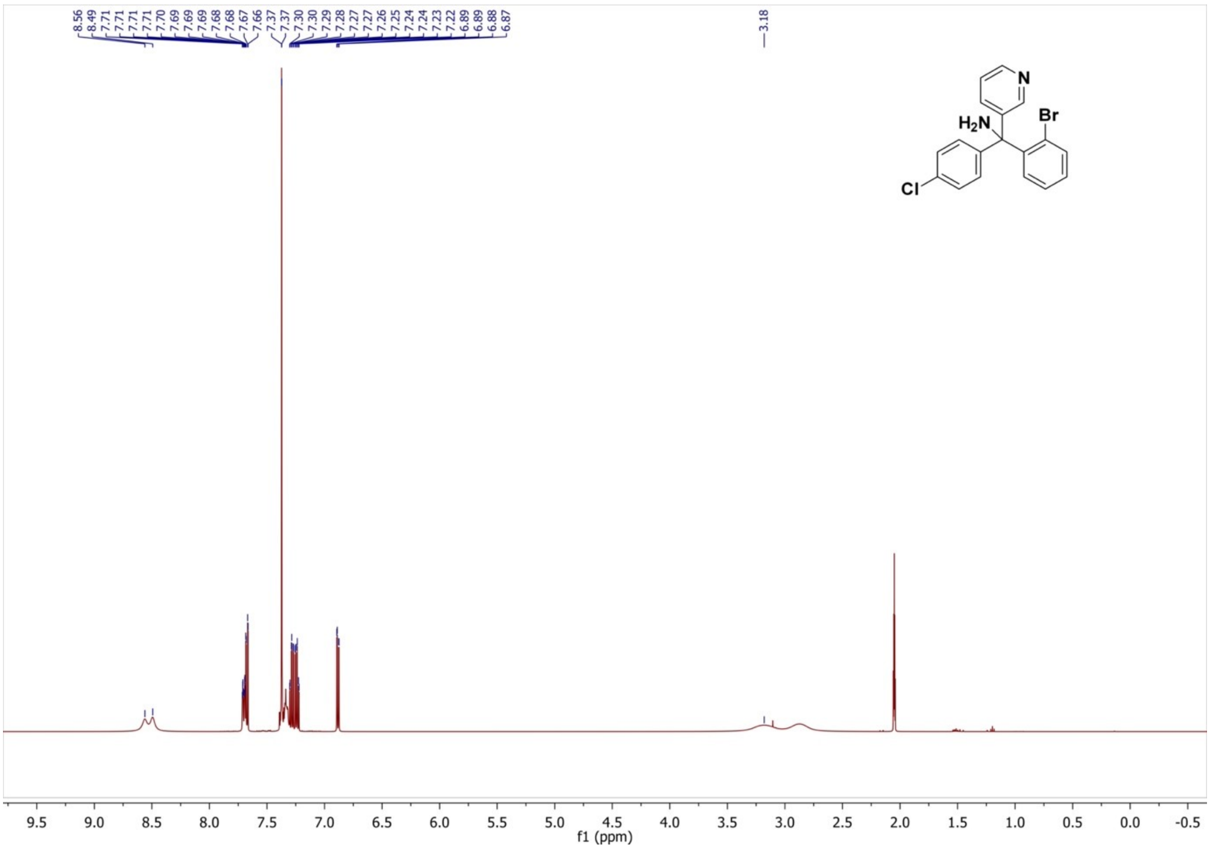


Figure S9. $^{1}H$ NMR spectrum of (2-bromophenyl)(4-chlorophenyl)(pyridin-3-yl)methanamine (**MYOS_00017**, 500 MHz, Acetone-$d_6$).

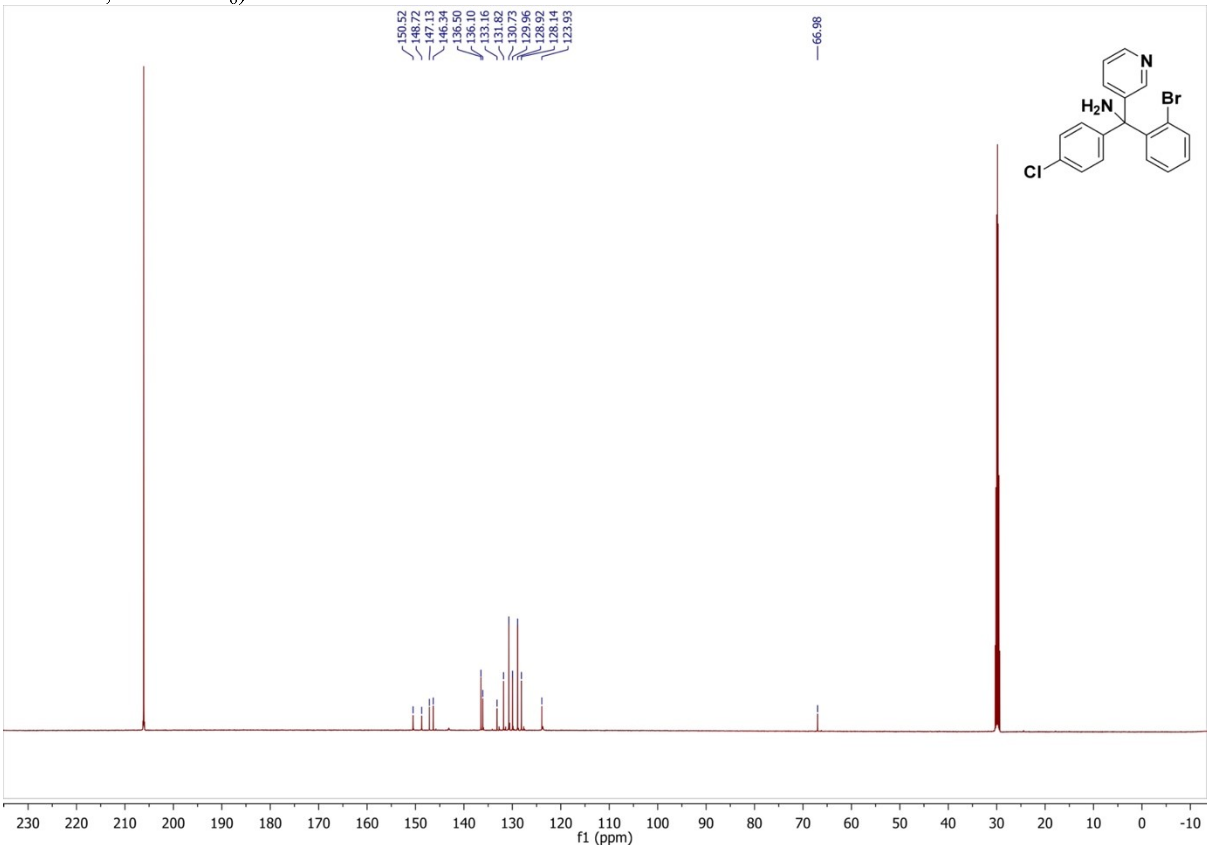


Figure S10. $^{13}C$ NMR spectrum of (2-bromophenyl)(4-chlorophenyl)(pyridin-3-yl)methanamine (**MYOS_00017**, 125 MHz, Acetone-$d_6$).

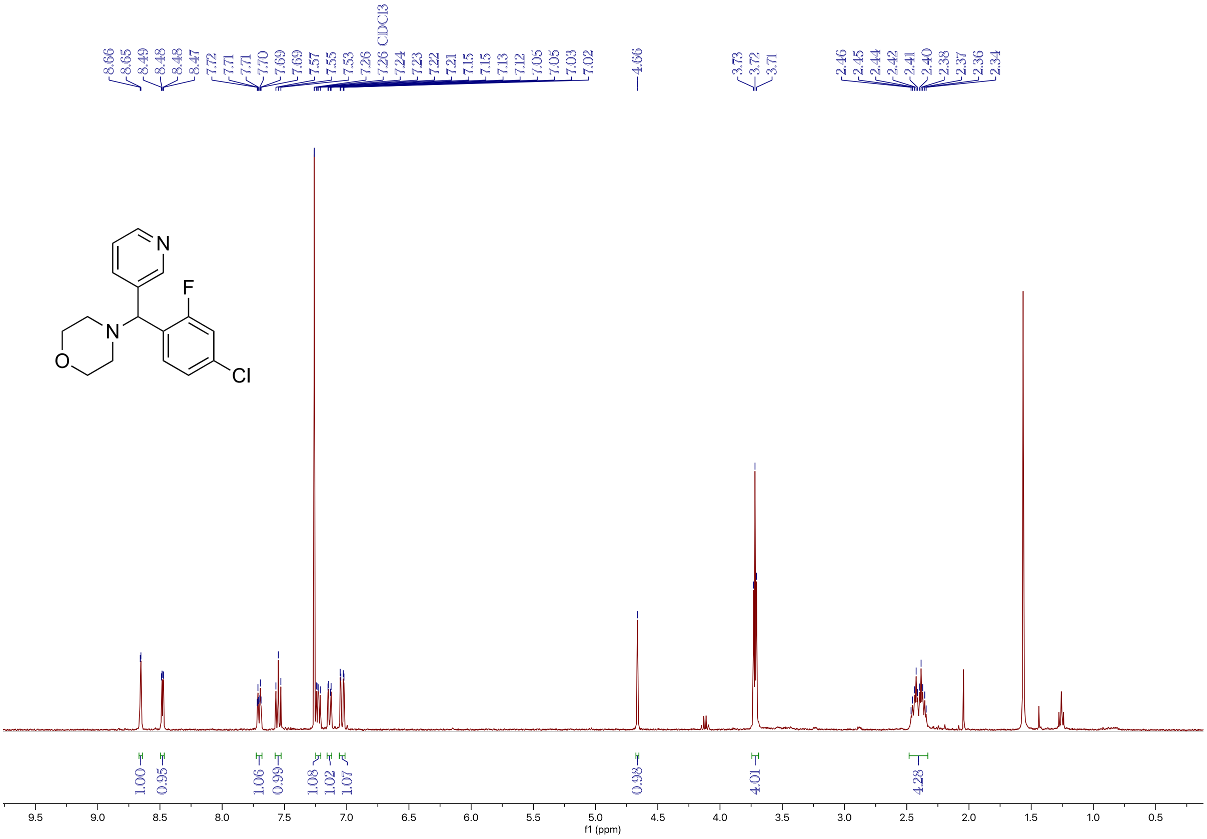


Figure S11. $^{1}$H NMR spectrum of 4-((4-chloro-2-fluorophenyl)(pyridin-3-yl)methyl)morpholine (**MYOS_00024**, 400 MHz, $CDCl_3$).

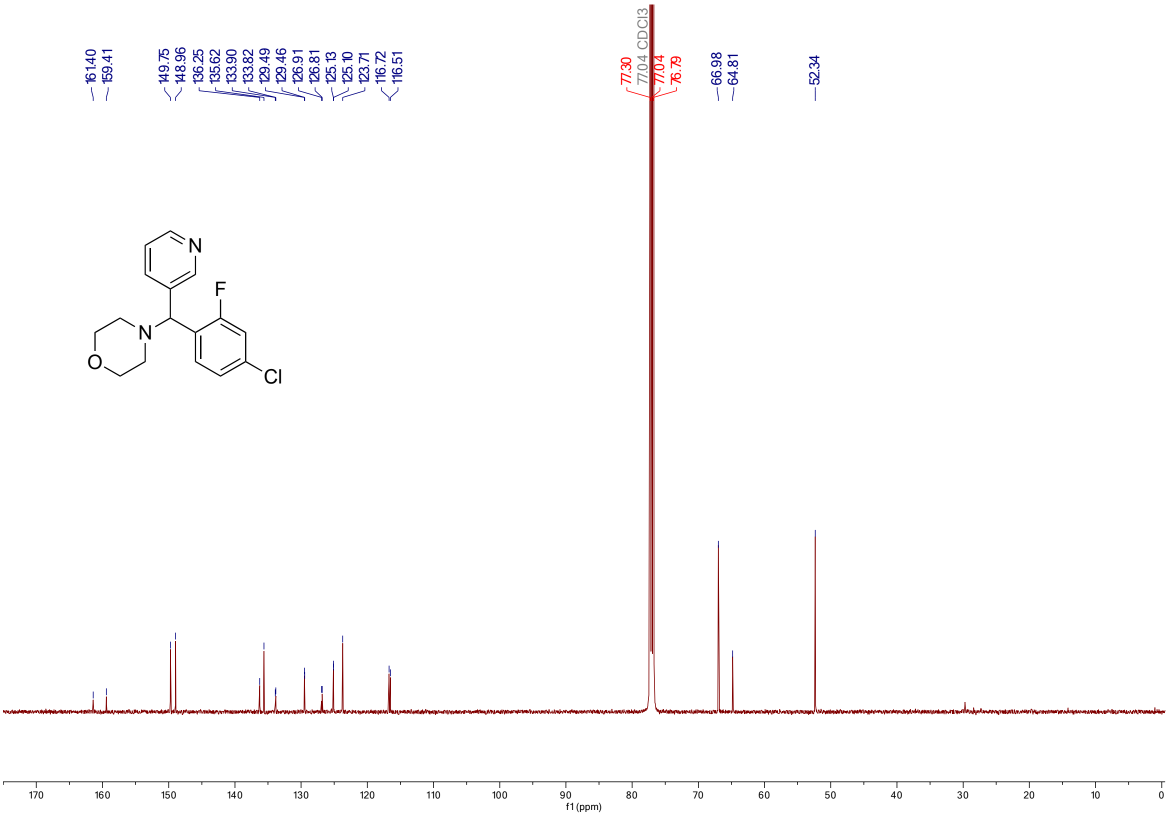


Figure S12. $^{13}$C NMR spectrum of 4-((4-chloro-2-fluorophenyl)(pyridin-3-yl)methyl)morpholine (**MYOS_00024**, 126 MHz, $CDCl_3$).

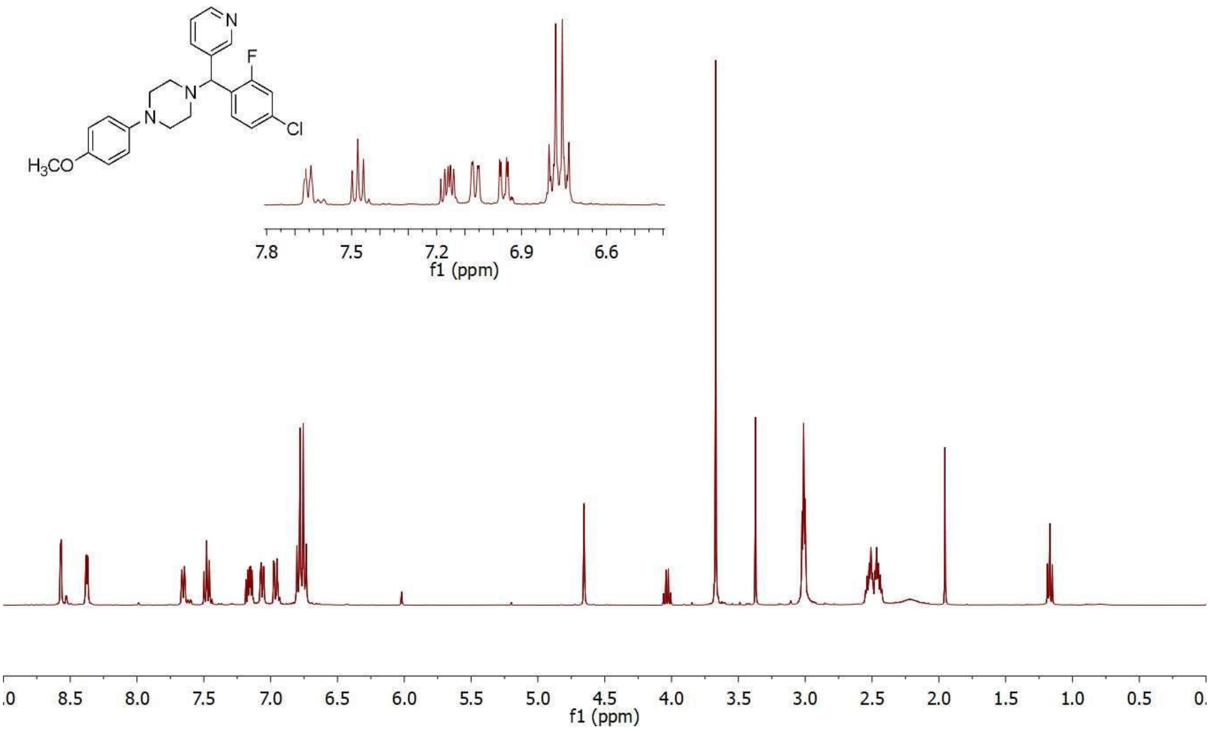

Figure S13. $^{1}H$ NMR spectrum of 1-((4-chloro-2-fluorophenyl)(pyridine-3-yl)methyl)-4-(4-methoxyphenyl)piperazine (**MYOS_00025**, 400 MHz, $CDCl_3$).

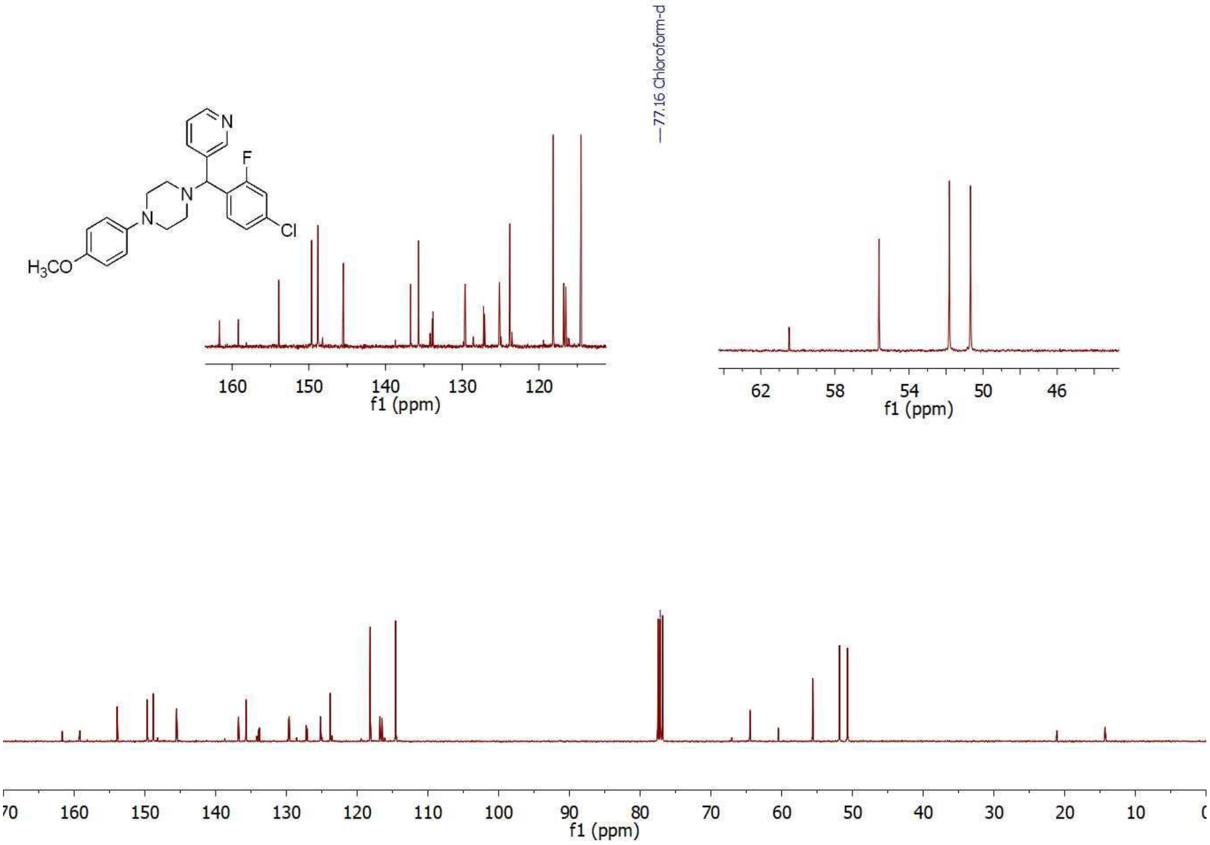

Figure S14. $^{13}C$ NMR spectrum of 1-((4-chloro-2-fluorophenyl)(pyridine-3-yl)methyl)-4-(4-methoxyphenyl)piperazine (**MYOS_00025**, 101 MHz, $CDCl_3$).

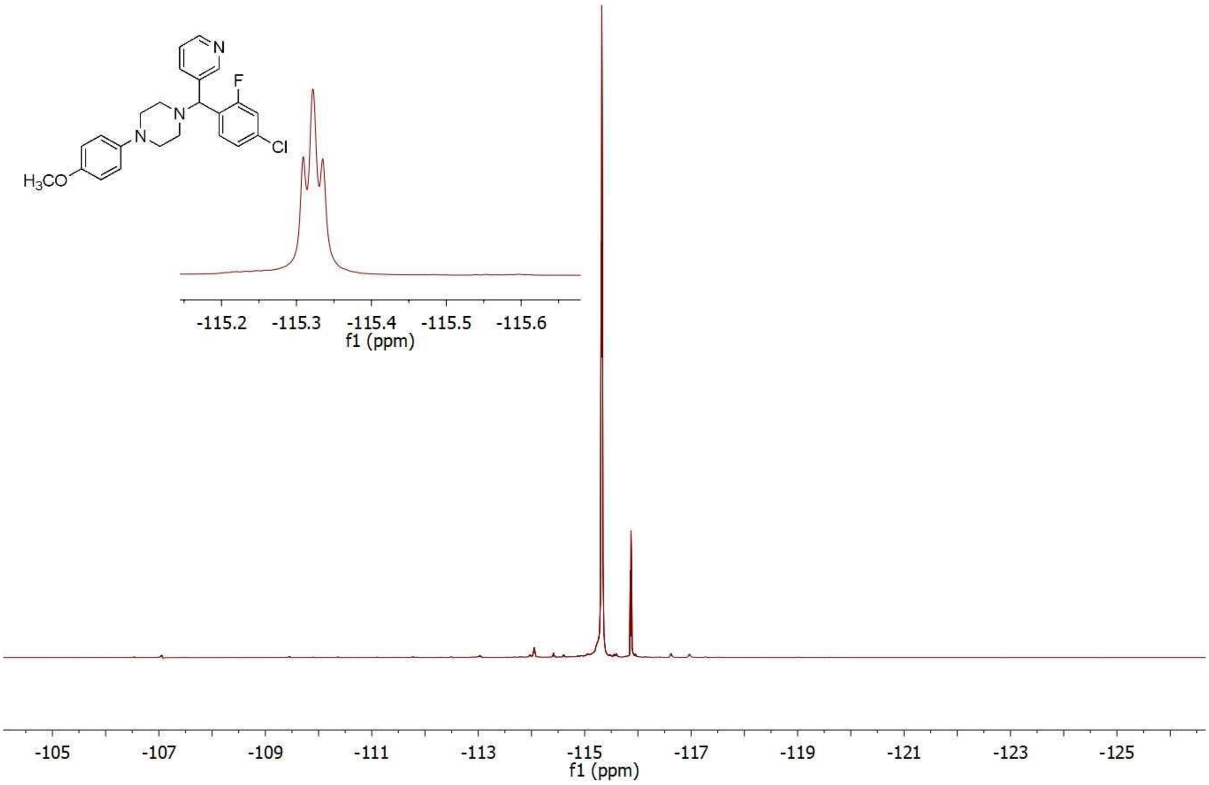


Figure S15. $^{19}F$ NMR spectrum of 1-((4-chloro-2-fluorophenyl)(pyridine-3-yl)methyl)-4-(4-methoxyphenyl)piperazine (**MYOS_00025**, 659 MHz, $CDCl_3$)

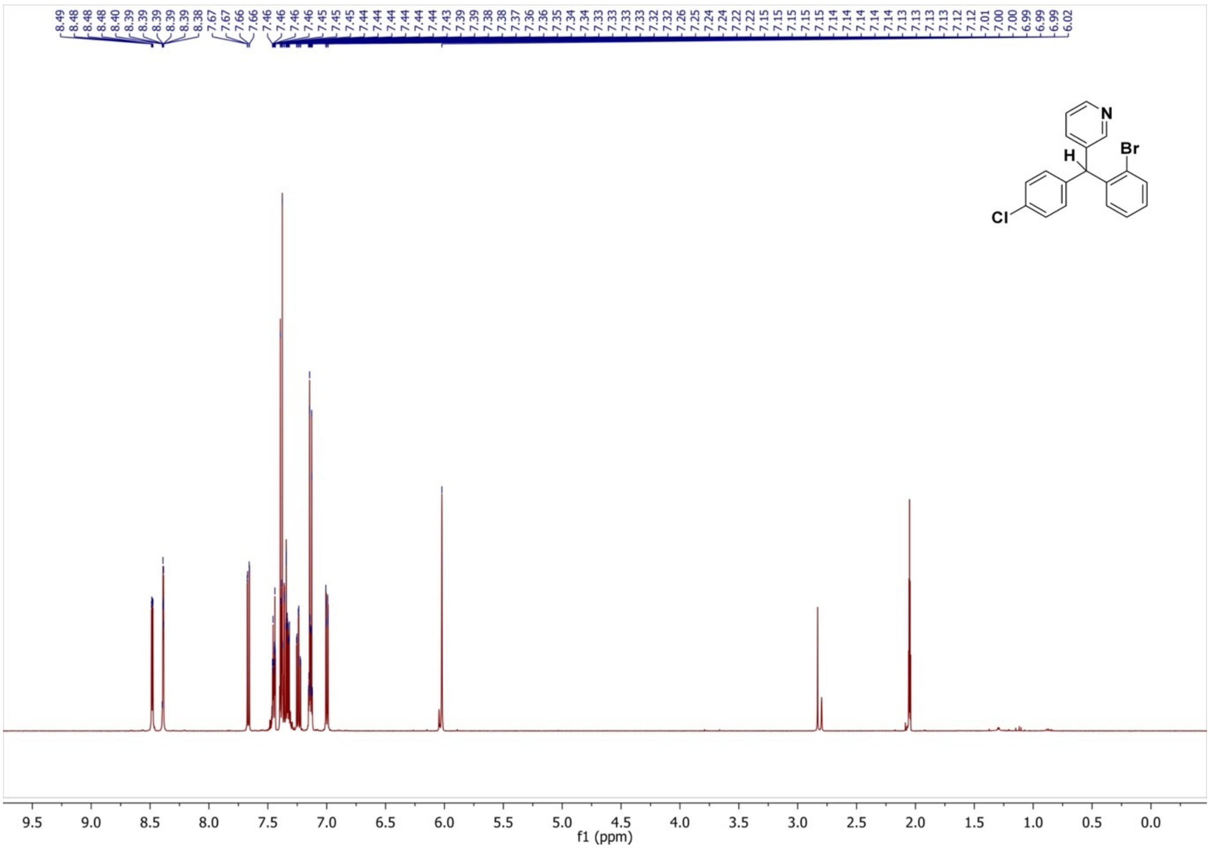

Figure S16. $^{1}$H NMR spectrum of 3-((2-bromophenyl)(4-chlorophenyl)methyl)pyridine (**MYOS_00027**, 500 MHz, Acetone-$d_6$).

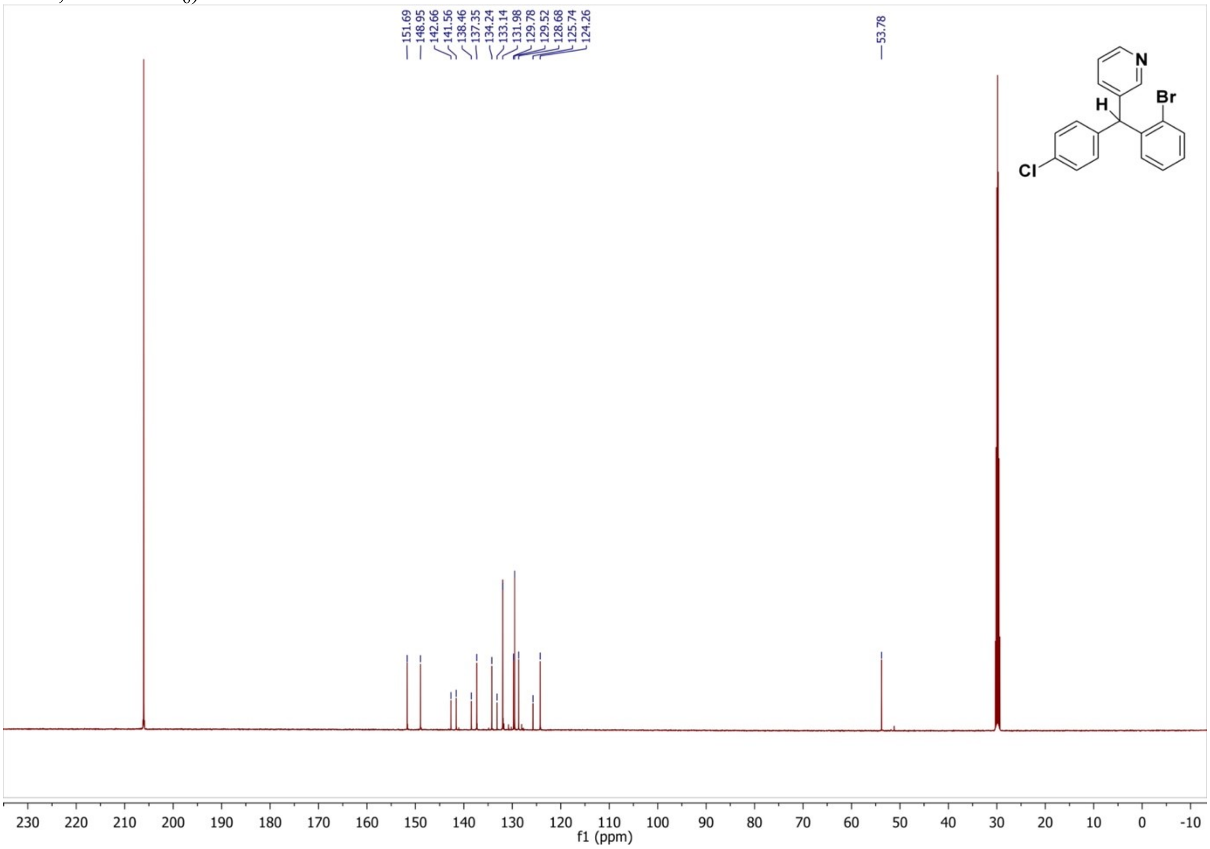

Figure S17. $^{13}$C NMR spectrum of 3-((2-bromophenyl)(4-chlorophenyl)methyl)pyridine (**MYOS_00027**, 125 MHz, Acetone-$d_6$).

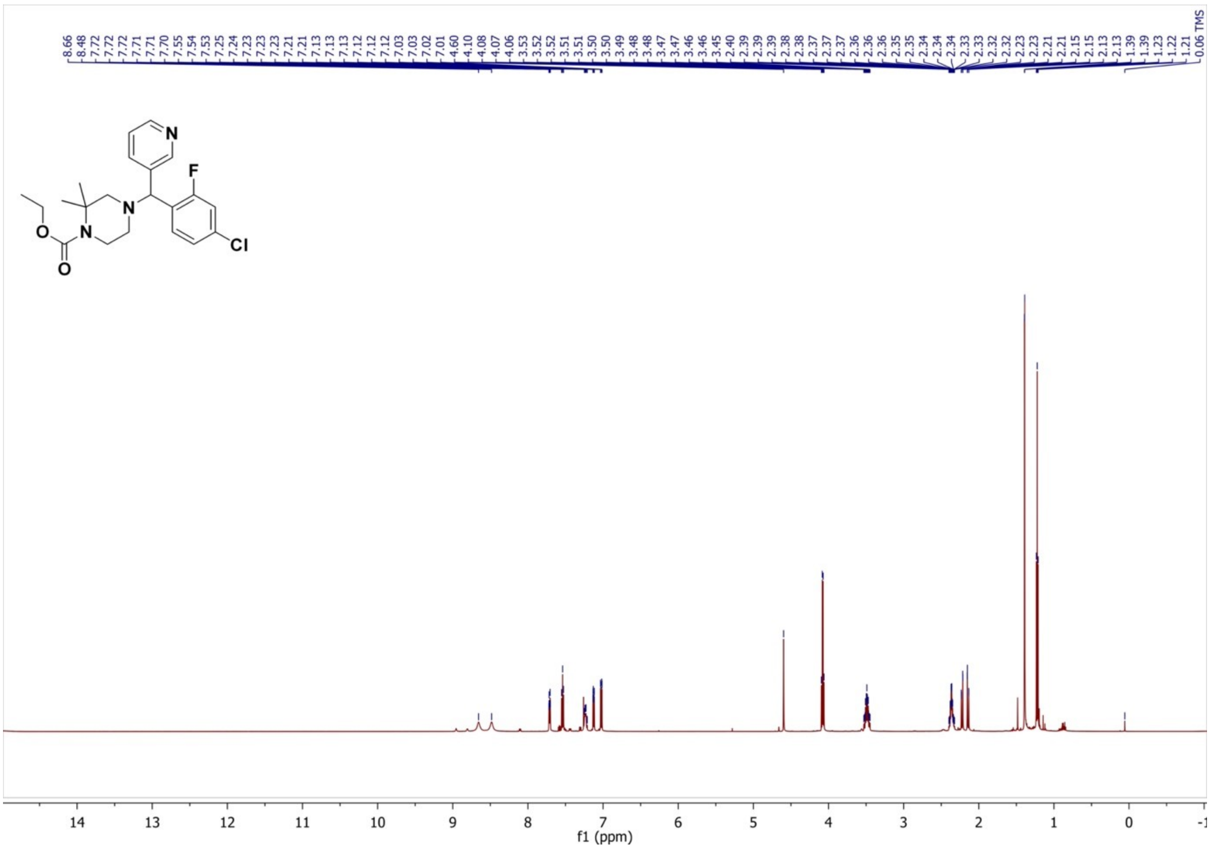


Figure S18. ¹H NMR spectrum of ethyl 4-((4-chloro-2-fluorophenyl)(pyridin-3-yl)methyl)-2,2-dimethylpiperazine-1-carboxylate (**MYOS_00028**, 600 MHz, $CDCl_3$).

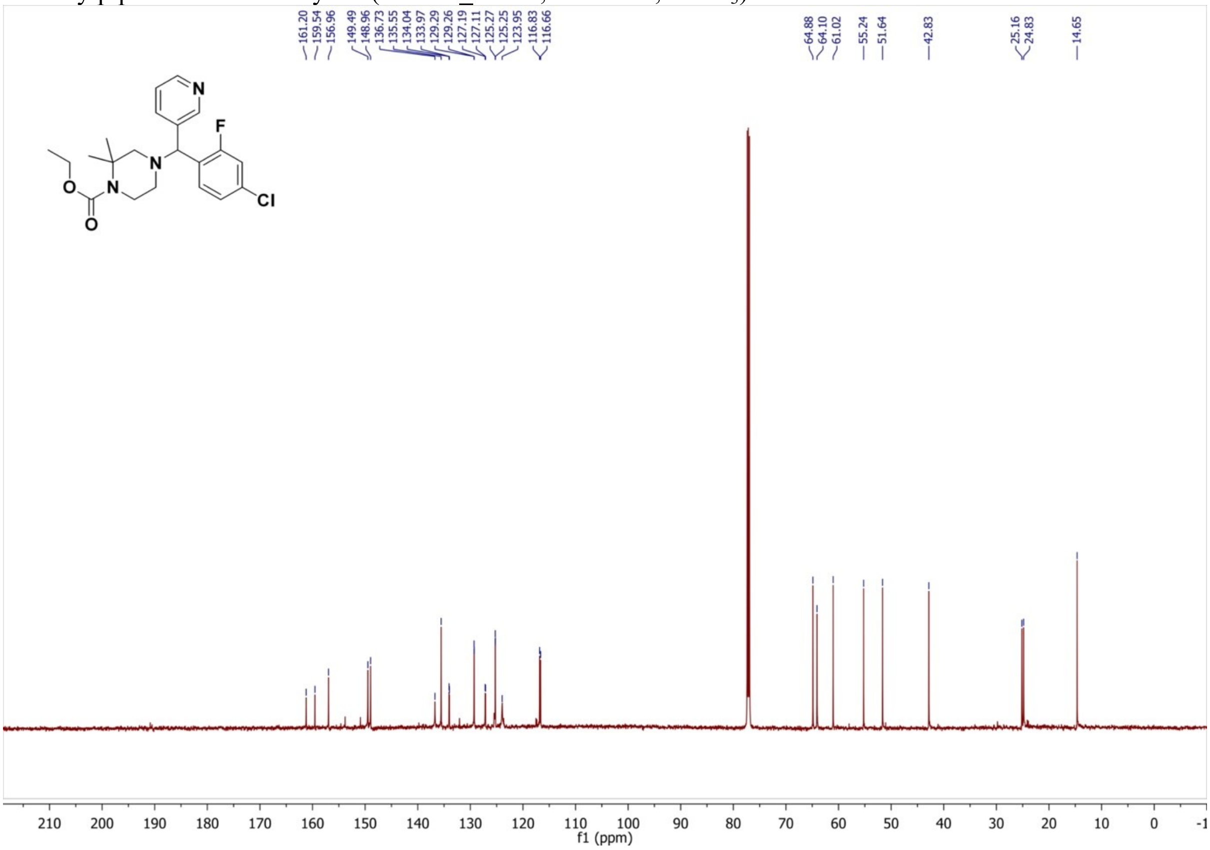


Figure S19. ¹³C NMR spectrum of ethyl 4-((4-chloro-2-fluorophenyl)(pyridin-3-yl)methyl)-2,2-dimethylpiperazine-1-carboxylate (**MYOS_00028**, 151 MHz, $CDCl_3$).

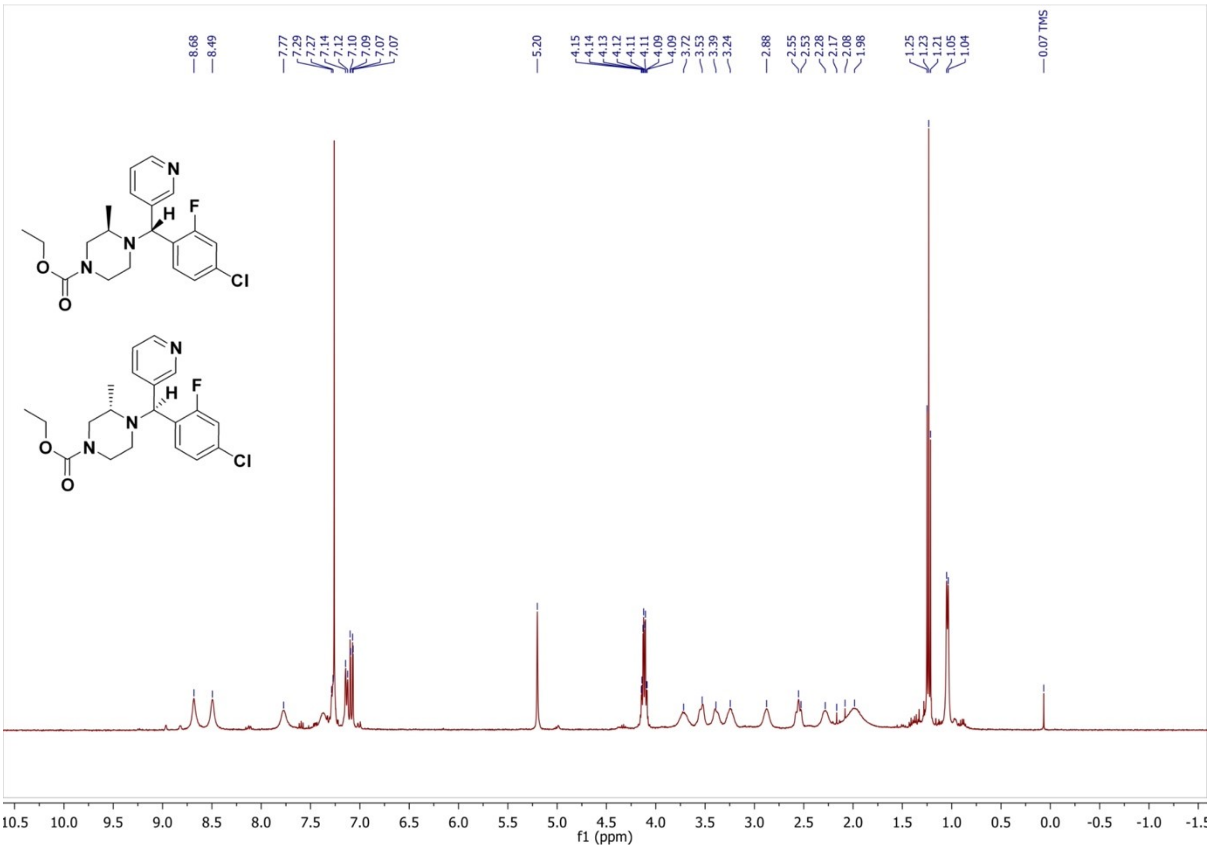


Figure S20. $^{1}$H NMR spectrum of ethyl 4-((4-chloro-2-fluorophenyl)(pyridin-3-yl)methyl)-3-methylpiperazine-1-carboxylate ((*R*,*R*) and (*S*,*S*)) (**MYOS_00029**, 400 MHz, $CDCl_3$).

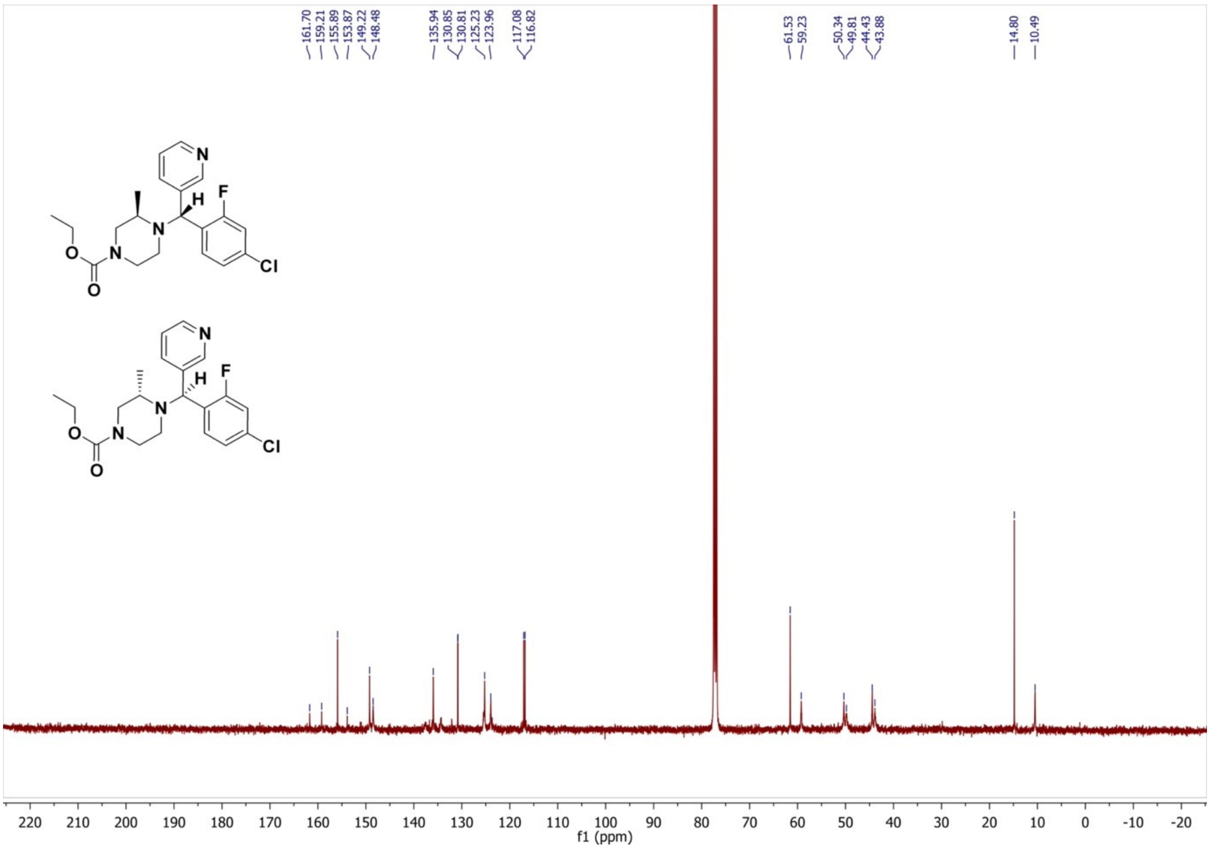


Figure S21. $^{13}$C NMR spectrum of ethyl 4-((4-chloro-2-fluorophenyl)(pyridin-3-yl)methyl)-3-methylpiperazine-1-carboxylate ((*R*,*R*) and (*S*,*S*)) (**MYOS_00029**, 101 MHz, $CDCl_3$).

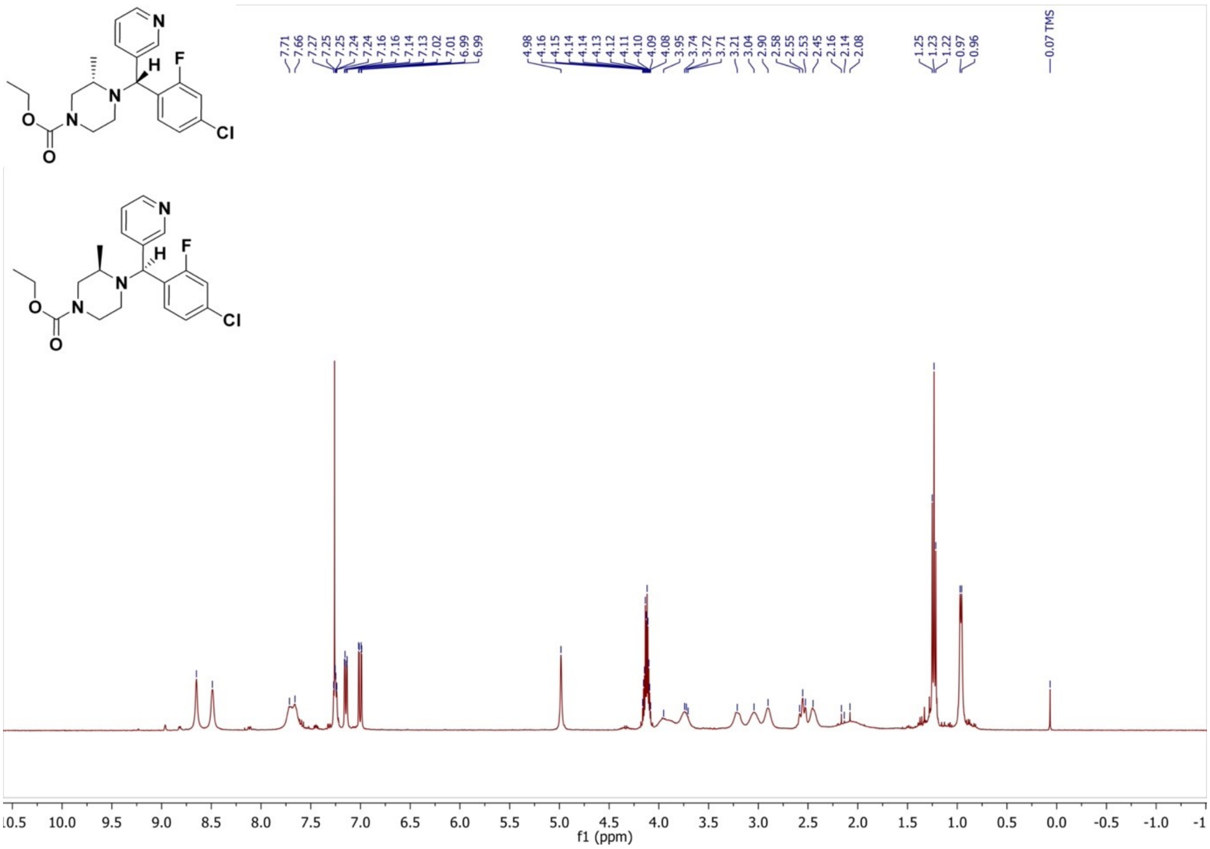


Figure S22. $^{1}$H NMR spectrum of ethyl 4-((4-chloro-2-fluorophenyl)(pyridin-3-yl)methyl)-3-methylpiperazine-1-carboxylate ((*R*,*S*) and (*S*,*R*)) (**MYOS_00030**, 400 MHz, $CDCl_3$).

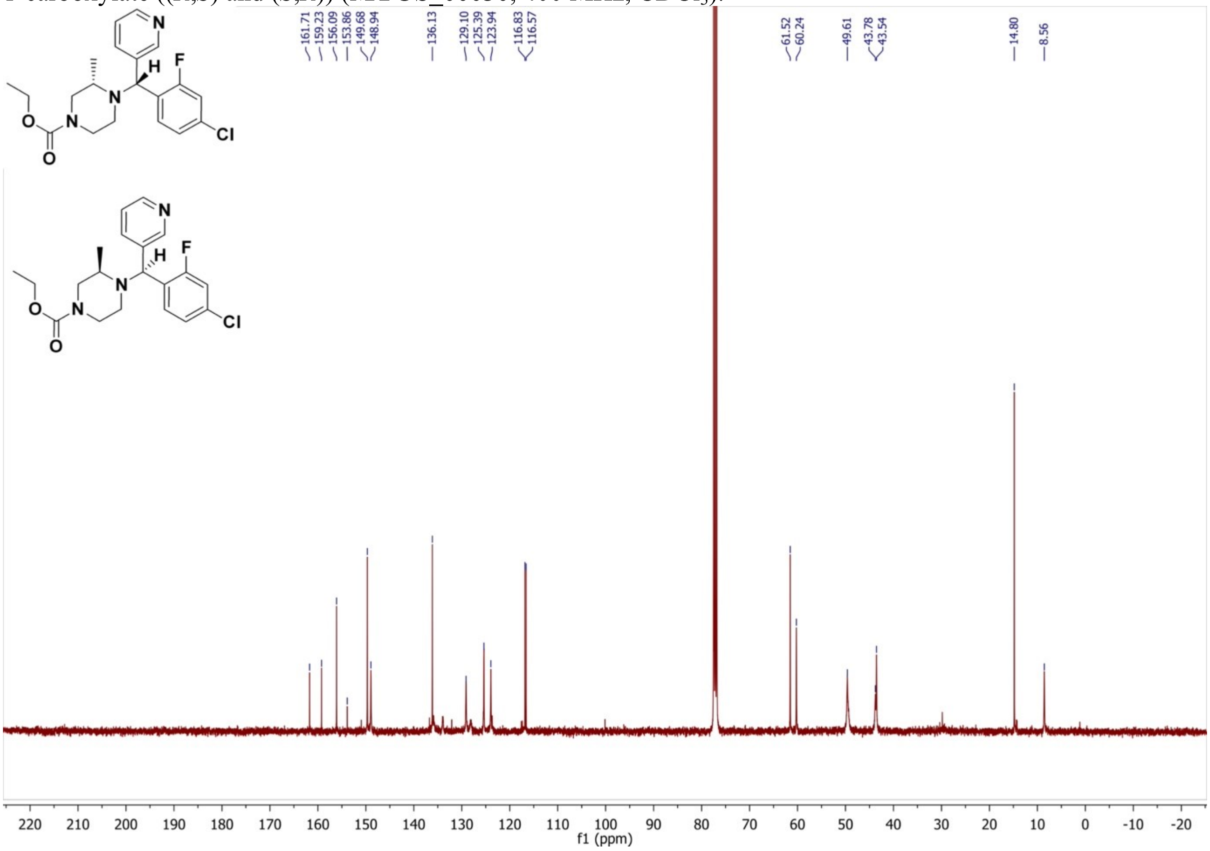


Figure S23. $^{13}$C NMR spectrum of ethyl 4-((4-chloro-2-fluorophenyl)(pyridin-3-yl)methyl)-3-methylpiperazine-1-carboxylate ((*R*,*S*) and (*S*,*R*)) (**MYOS_00030**, 101 MHz, $CDCl_3$).

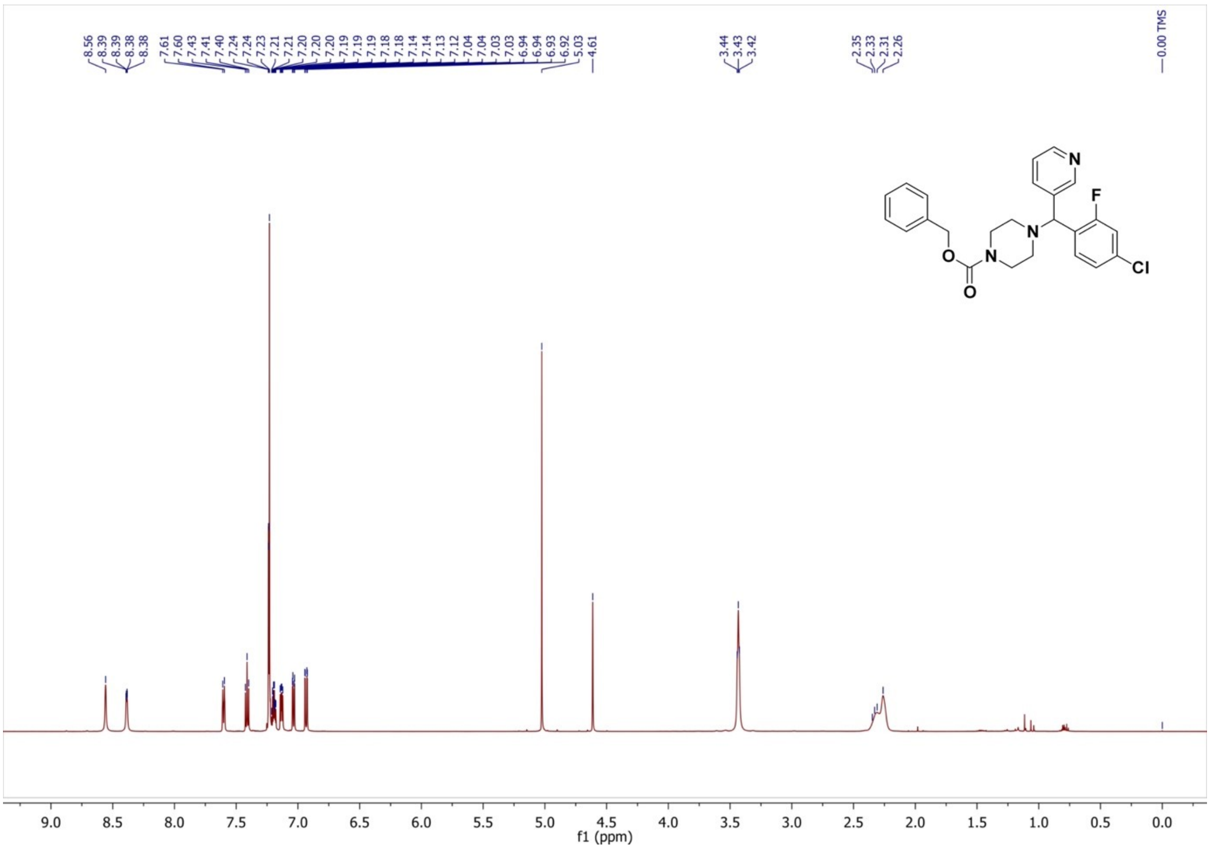


Figure S24. $^{1}$H NMR spectrum of benzyl 4-((4-chloro-2-fluorophenyl)(pyridin-3-yl)methyl)piperazine-1-carboxylate (**MYOS_00031**, 600 MHz, $CDCl_3$).

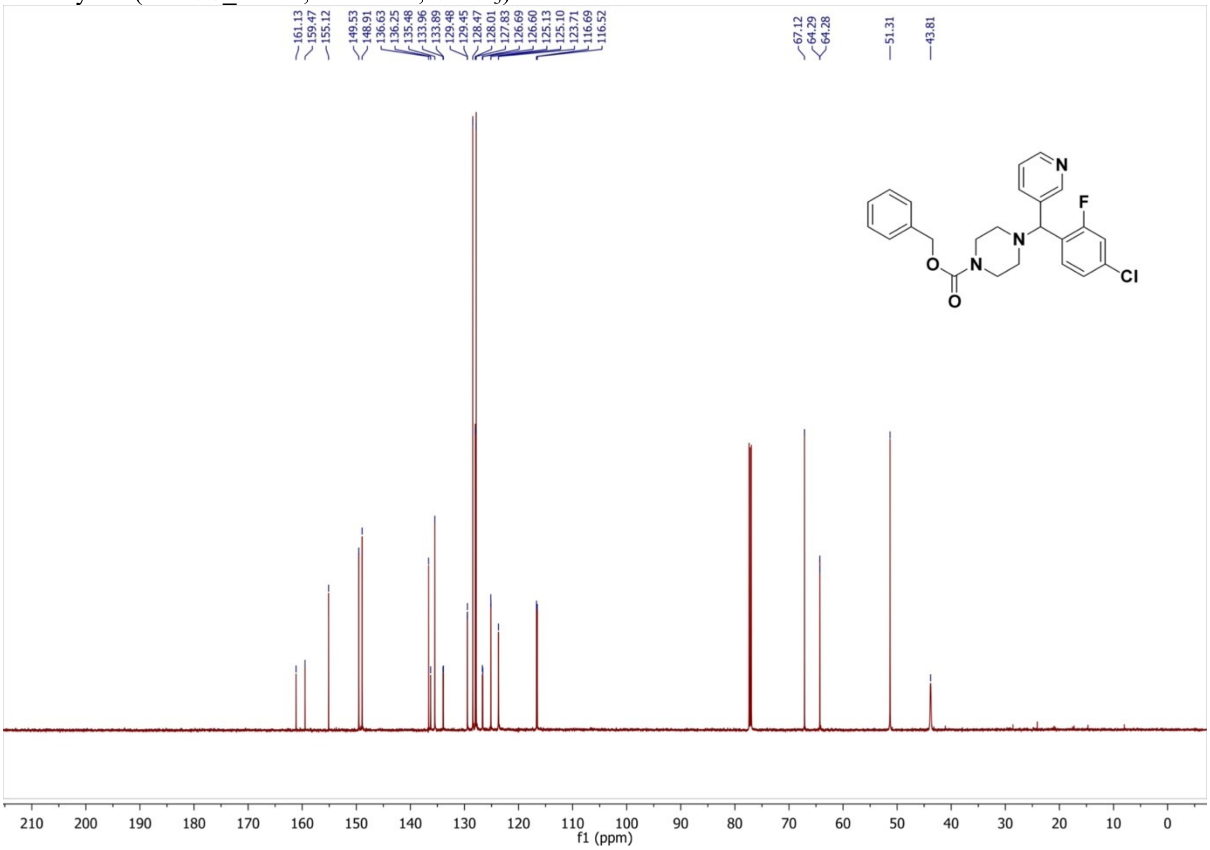


Figure S25. $^{13}$C NMR spectrum of benzyl 4-((4-chloro-2-fluorophenyl)(pyridin-3-yl)methyl)piperazine-1-carboxylate (**MYOS_00031**, 151 MHz, $CDCl_3$).

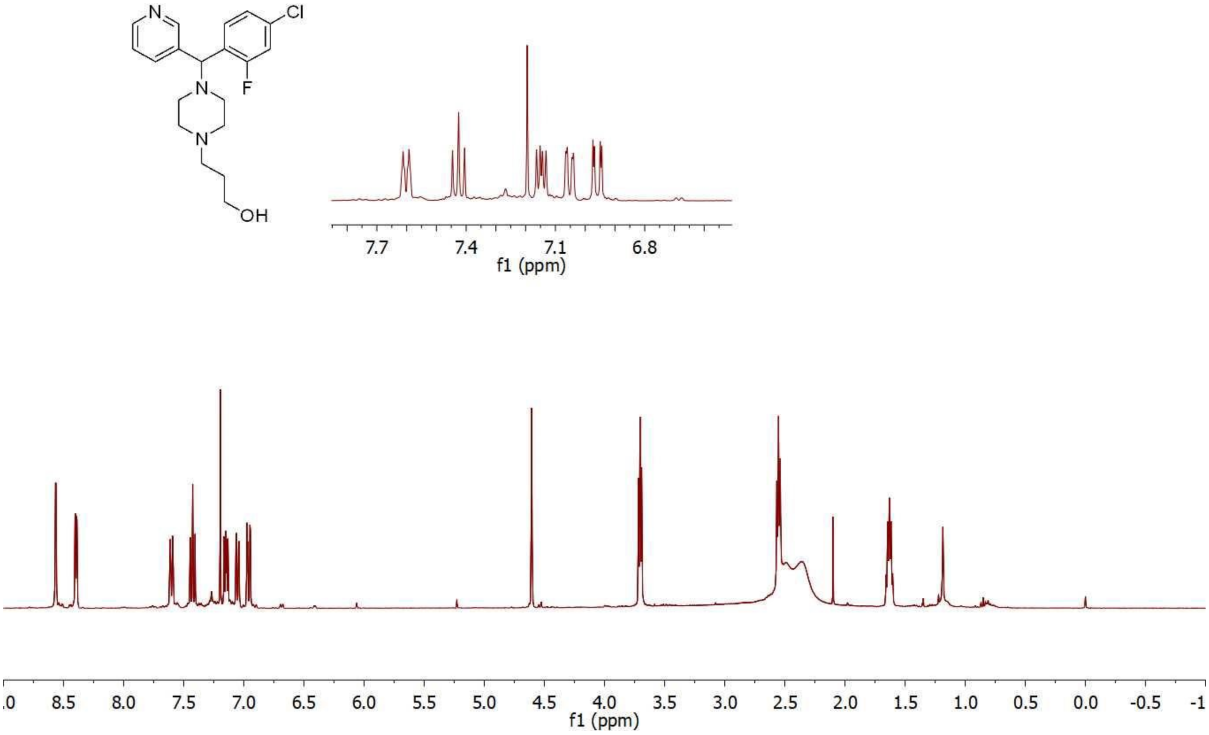


Figure S26. $^{1}$H NMR spectrum of (3-(4-((4-chloro-2-fluorophenyl)(pyridin-3-yl)methyl)piperazin-1-yl)propan-1-ol (**MYOS_00033**, 400 MHz, $CDCl_3$).

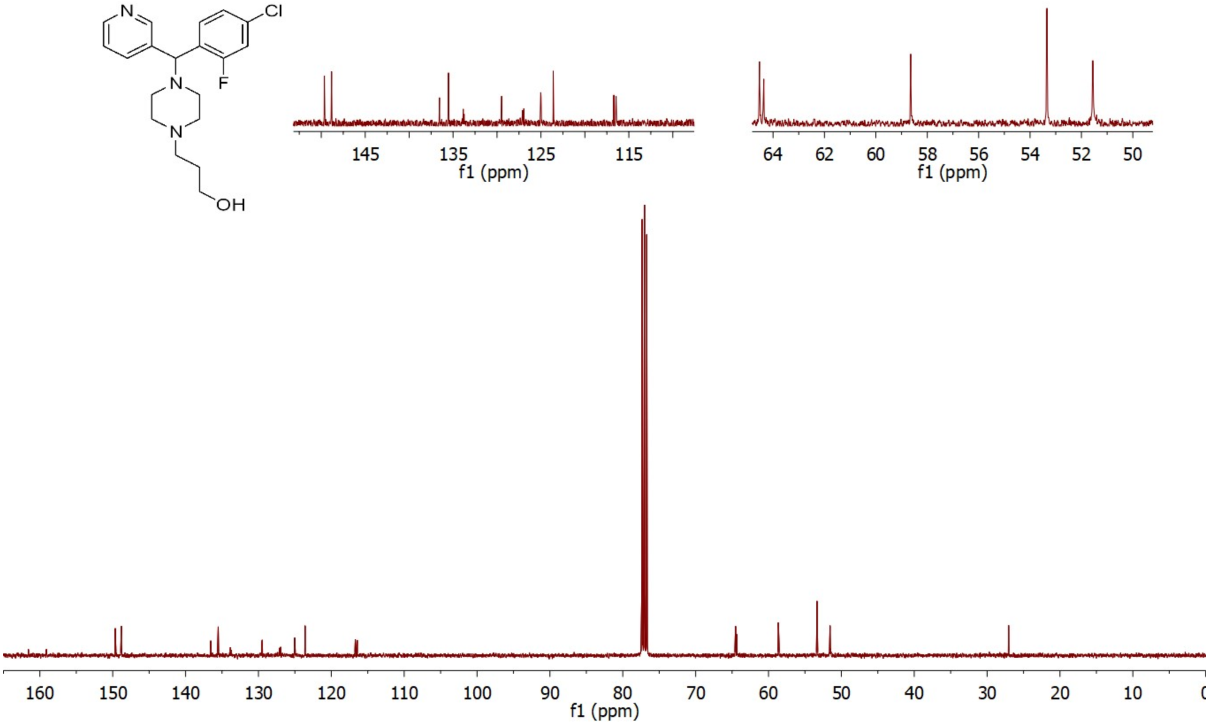


Figure S27. $^{13}$C NMR spectrum of (3-(4-((4-chloro-2-fluorophenyl)(pyridin-3-yl)methyl)piperazin-1-yl)propan-1-ol (**MYOS_00033**, 101 MHz, $CDCl_3$).

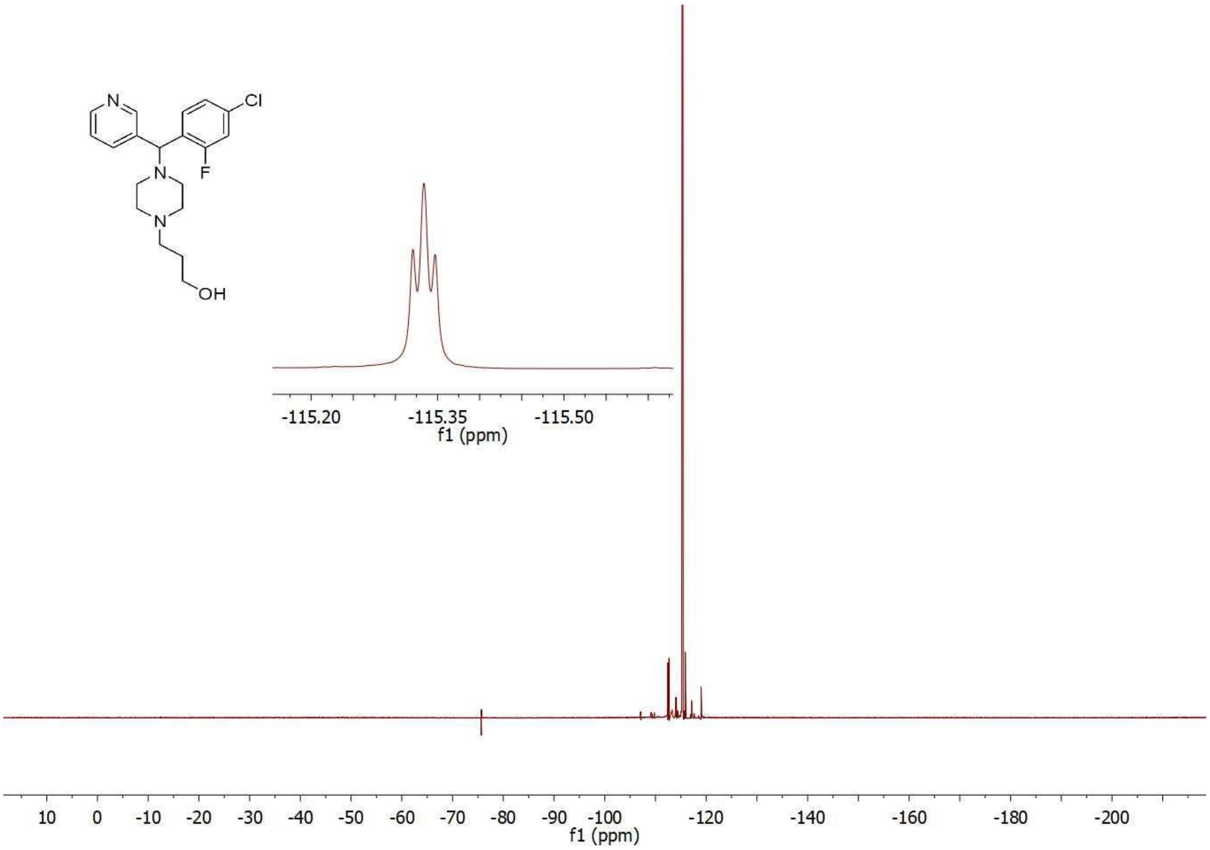


Figure S28. $^{19}$F NMR spectrum of (3-(4-((4-chloro-2-fluorophenyl)(pyridin-3-yl)methyl)piperazin-1-yl)propan-1-ol (**MYOS_00033**, 659 MHz, $CDCl_3$)

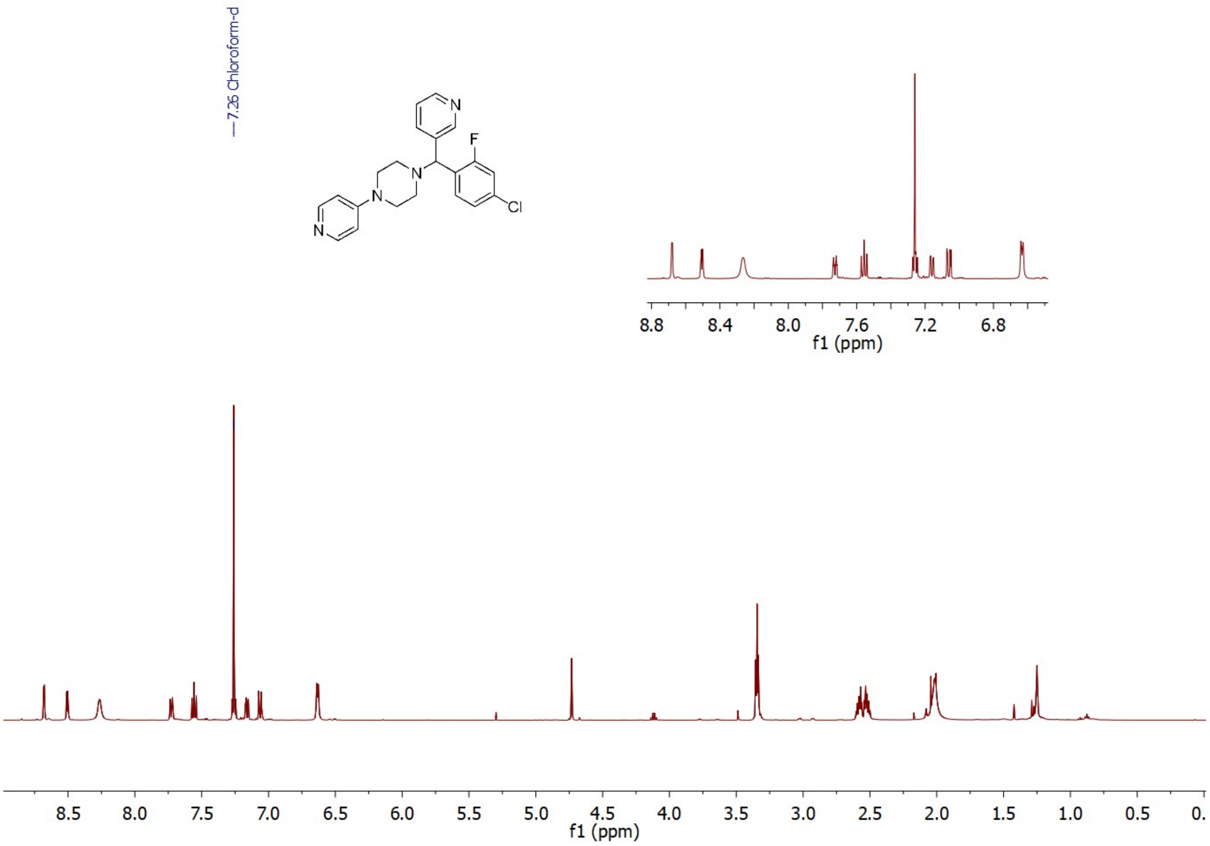


Figure S29. $^{1}$H NMR spectrum of 1-((4-chloro-2-fluorophenyl)(pyridin-3-yl)methyl)-4-(pyridin-4-yl)piperzine, (**MYOS_00034**, 500 MHz, $CDCl_3$).

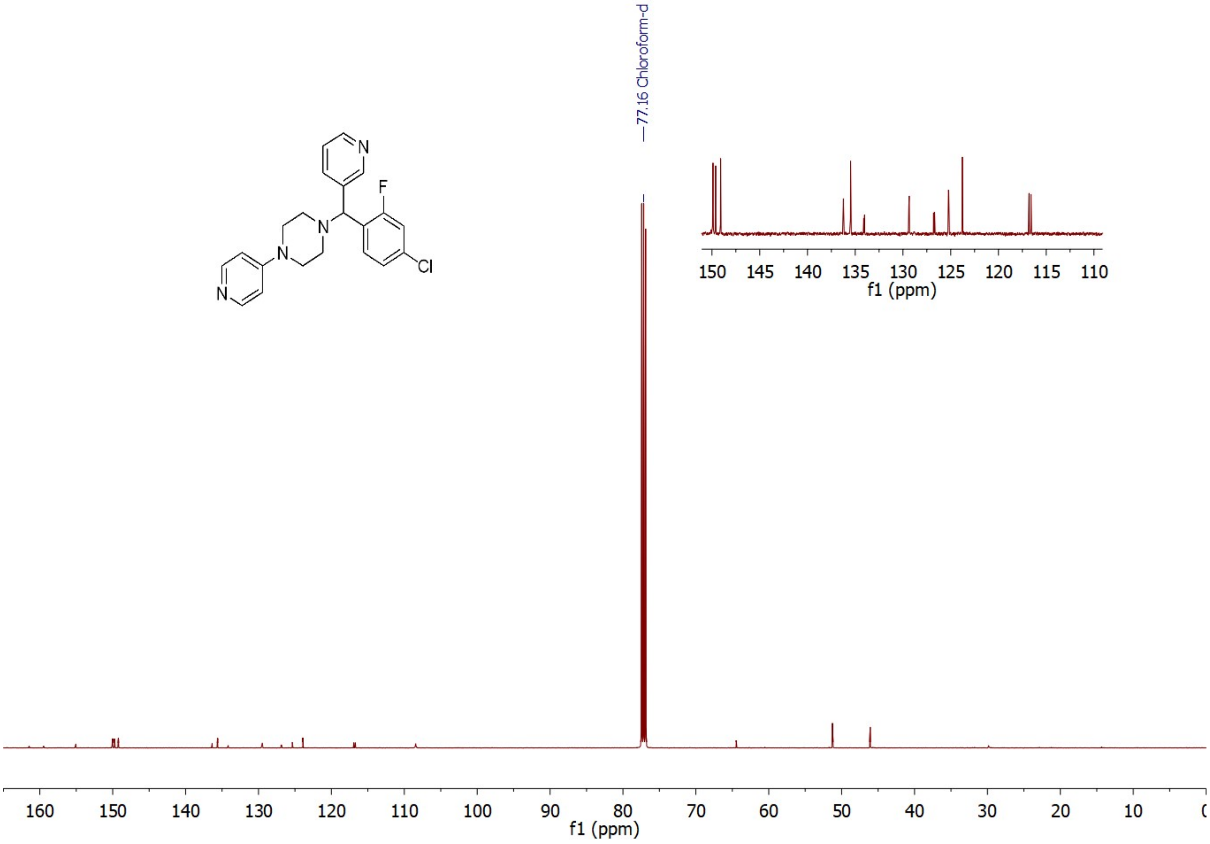


Figure S30. $^{13}$C NMR spectrum of 1-((4-chloro-2-fluorophenyl)(pyridin-3-yl)methyl)-4-(pyridin-4-yl)piperzine, (**MYOS_00034**, 126 MHz, $CDCl_3$).

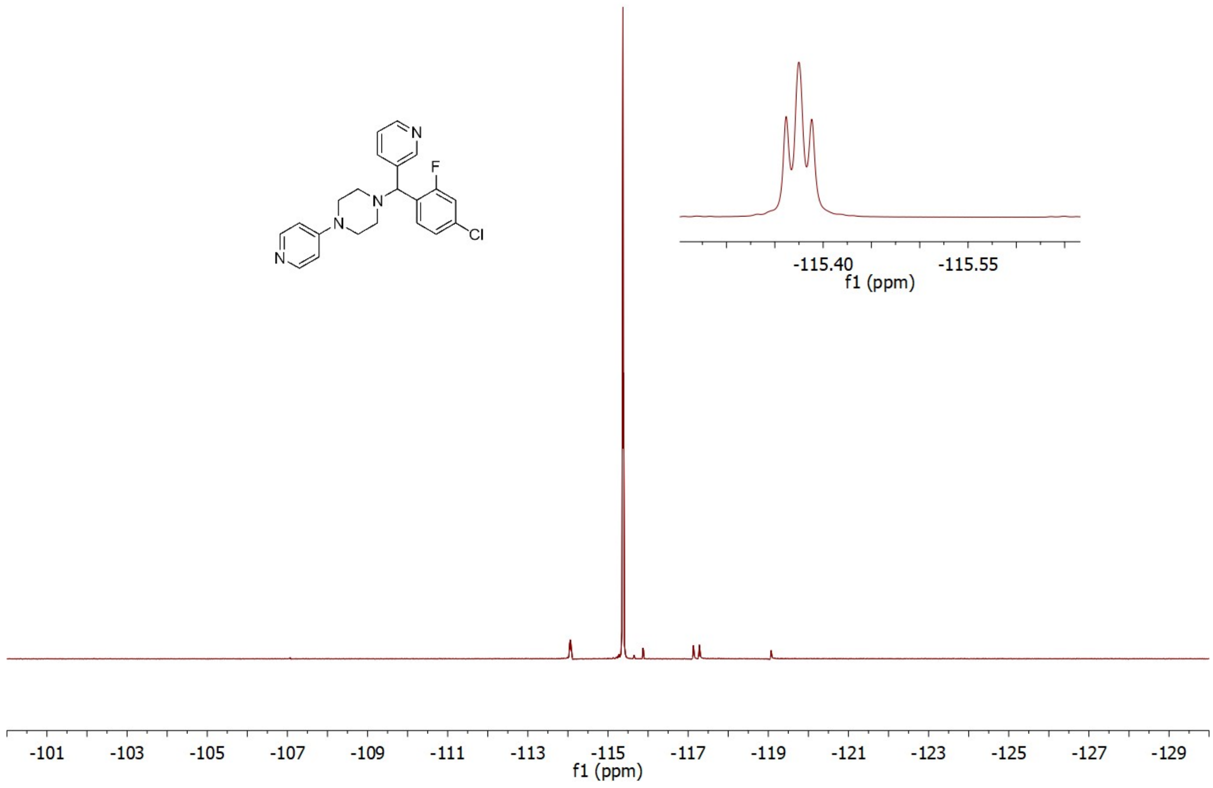


Figure S31. $^{19}F$ NMR spectrum of 1-((4-chloro-2-fluorophenyl)(pyridin-3-yl)methyl)-4-(pyridin-4-yl)piperzine, (**MYOS_00034**, 659 MHz, $CDCl_3$)

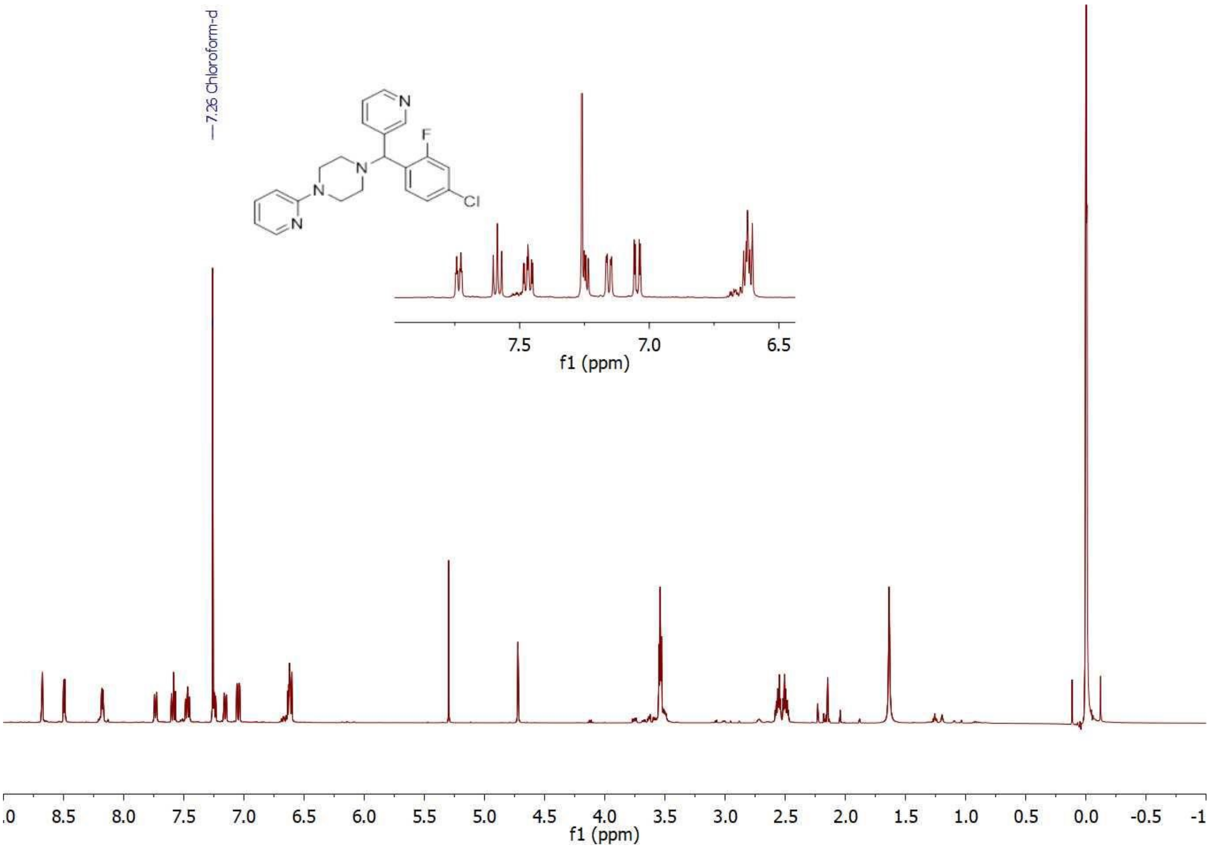


Figure S32. $^{1}$H NMR spectrum of 1-4((4-chloro-2-fluorophenyl)(pyridine-3-yl)methyl)-4-(pyridine-2-yl)piperazine (**MYOS_00035**, 500 MHz, $CDCl_3$).

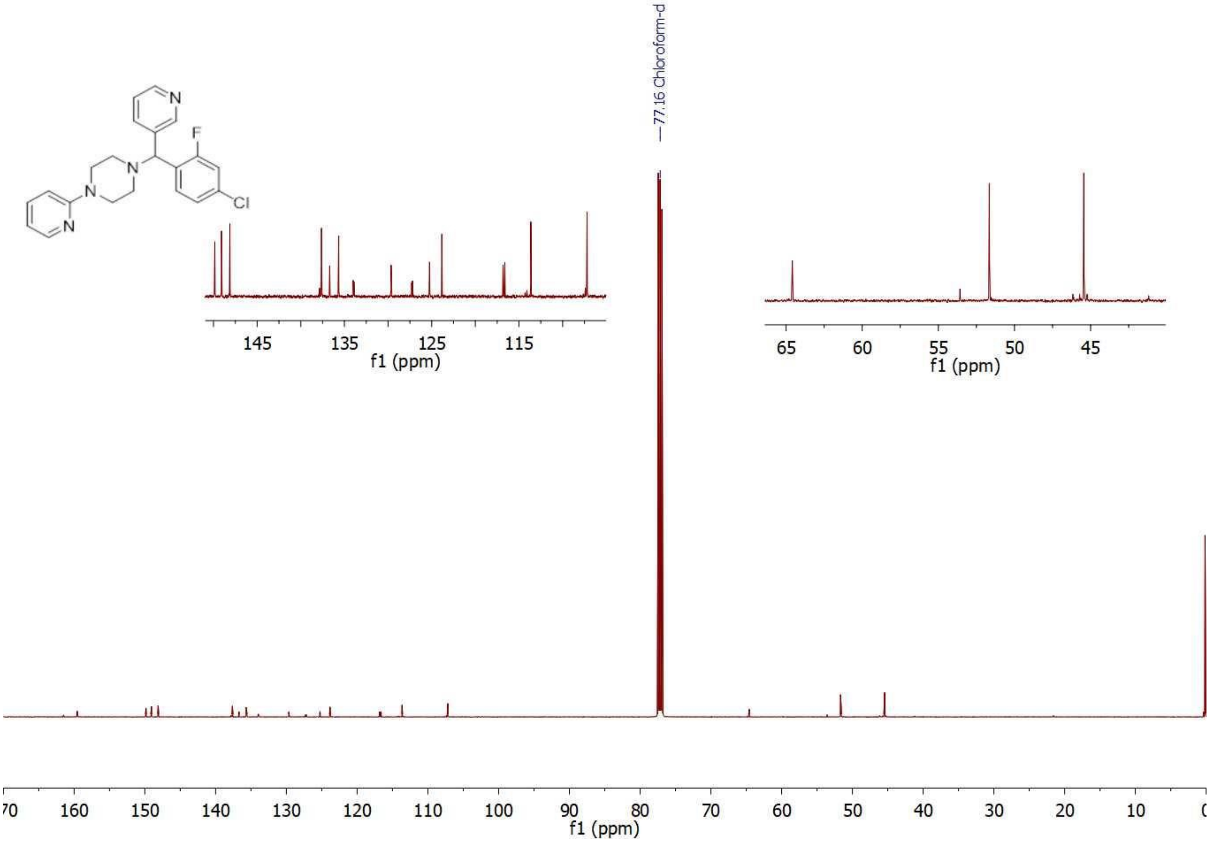


Figure S33. $^{13}$C NMR spectrum of 1-4((4-chloro-2-fluorophenyl)(pyridine-3-yl)methyl)-4-(pyridine-2-yl)piperazine (**MYOS_00035**, 126 MHz, $CDCl_3$).

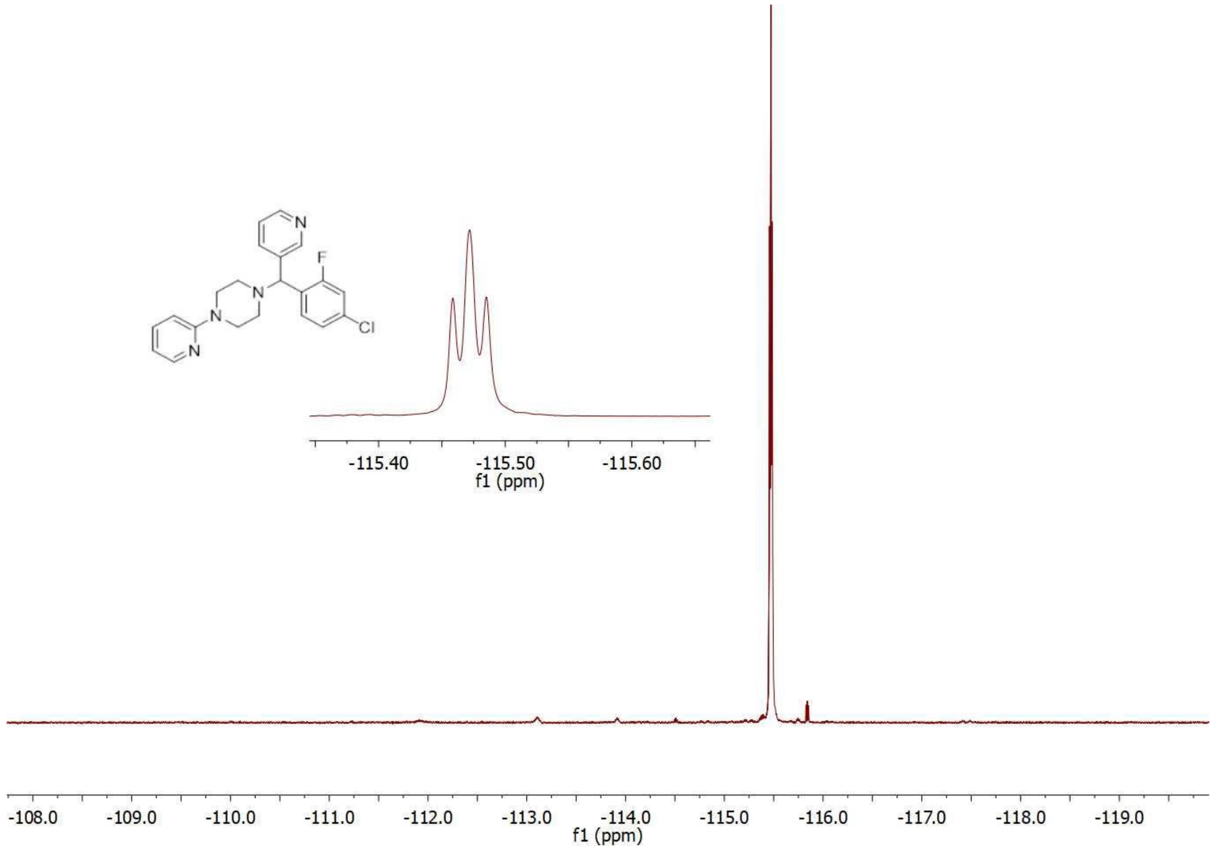


Figure S34. $^{19}$F NMR spectrum of 1-4((4-chloro-2-fluorophenyl)(pyridine-3-yl)methyl)-4-(pyridine-2-yl)piperazine (**MYOS_00035**, 659 MHz, $CDCl_3$)

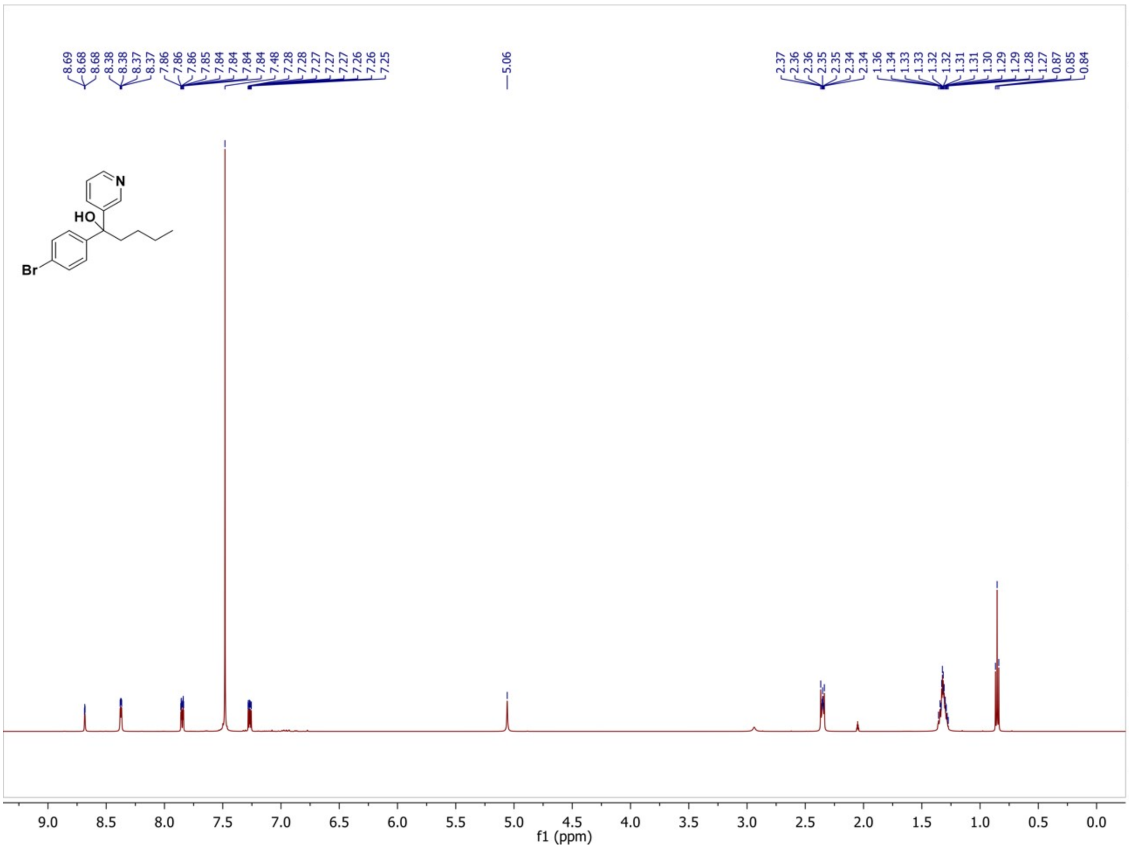


Figure S35. $^{1}$H NMR spectrum of 1-(4-bromophenyl)-1-(pyridin-3-yl)pentan-1-ol (**MYOS_00130**, 500 MHz, Acetone-$d_6$).

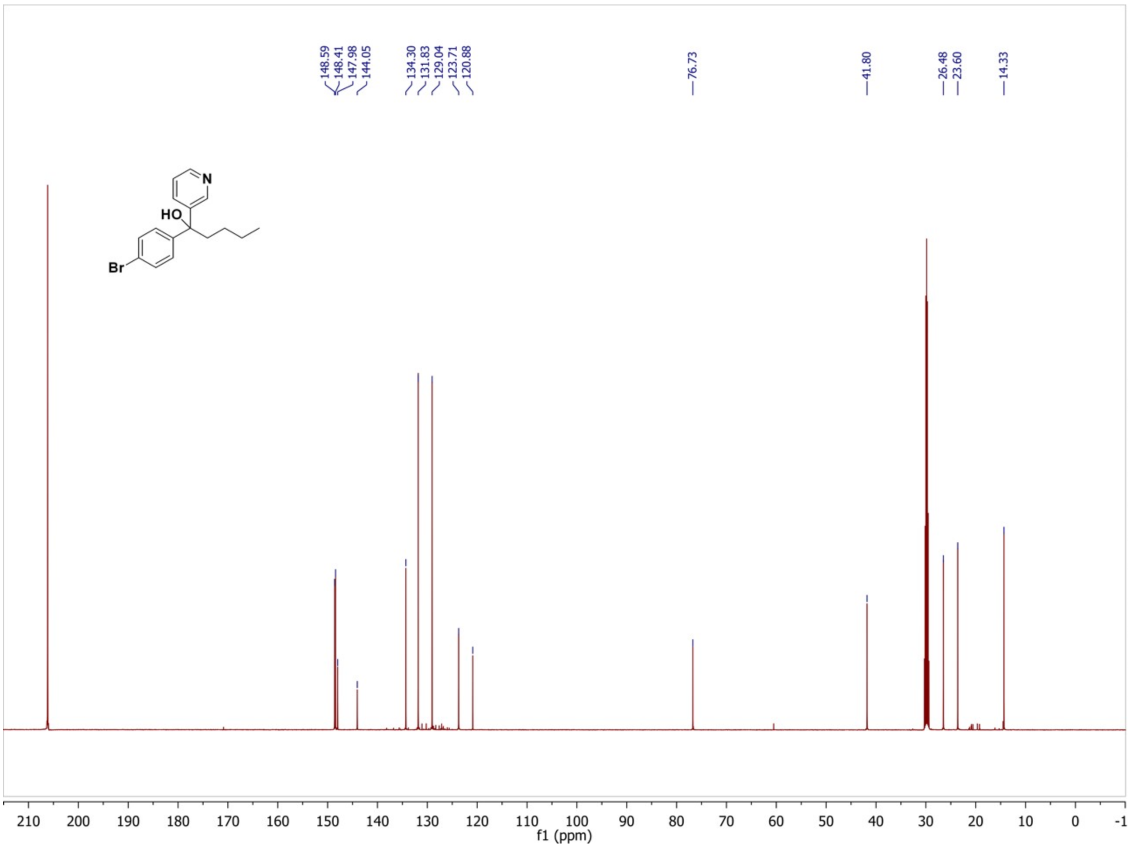


Figure S36. $^{13}$C NMR spectrum of 1-(4-bromophenyl)-1-(pyridin-3-yl)pentan-1-ol (**MYOS_00130**, 126 MHz, Acetone-$d_6$).

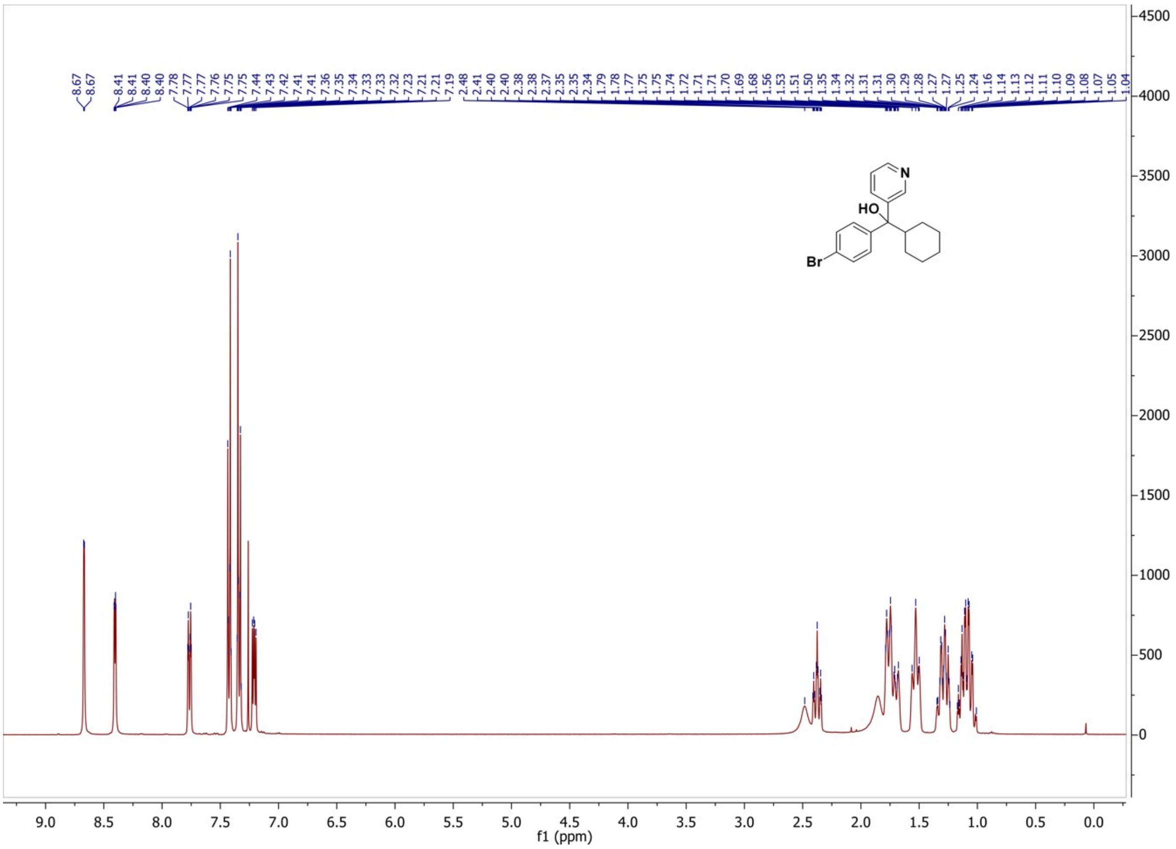

Figure S37. $^{1}$H NMR spectrum of (4-bromophenyl)(cyclohexyl)(pyridin-3-yl)methanol (**MYOS_00131**, 400 MHz, $CDCl_3$).

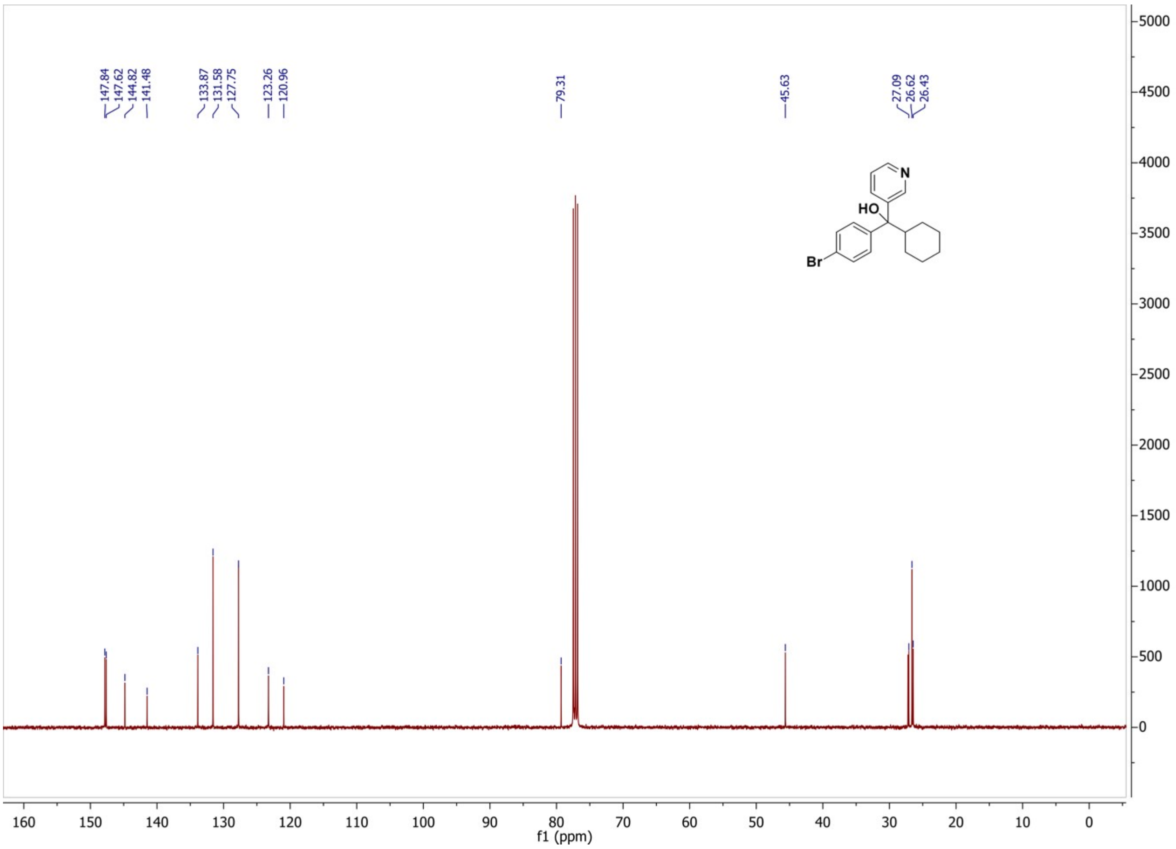

Figure S38. $^{13}$C NMR spectrum of (4-bromophenyl)(cyclohexyl)(pyridin-3-yl)methanol (**MYOS_00131**, 101 MHz, $CDCl_3$).

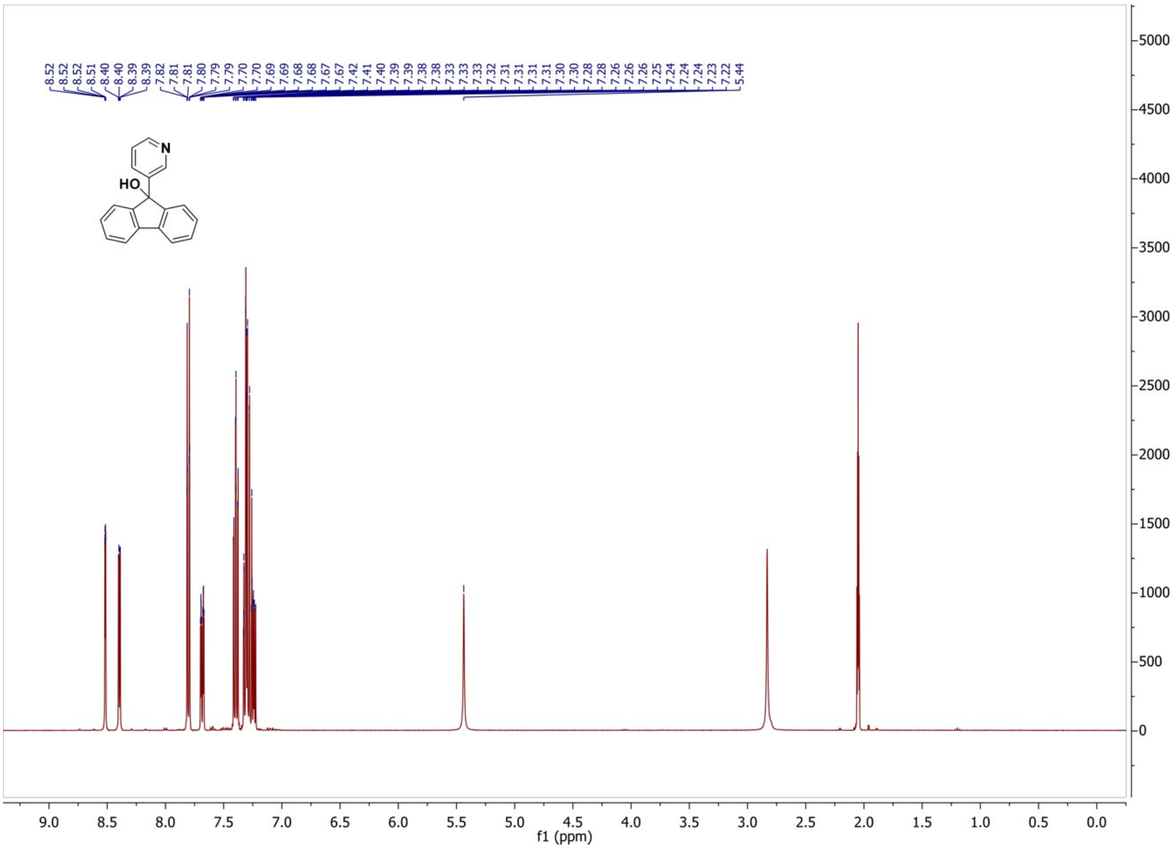

Figure S39. $^{1}$H NMR spectrum of 9-(pyridin-3-yl)-9*H*-fluoren-9-ol (**MYOS_00132**, 400 MHz, Acetone-$d_6$).

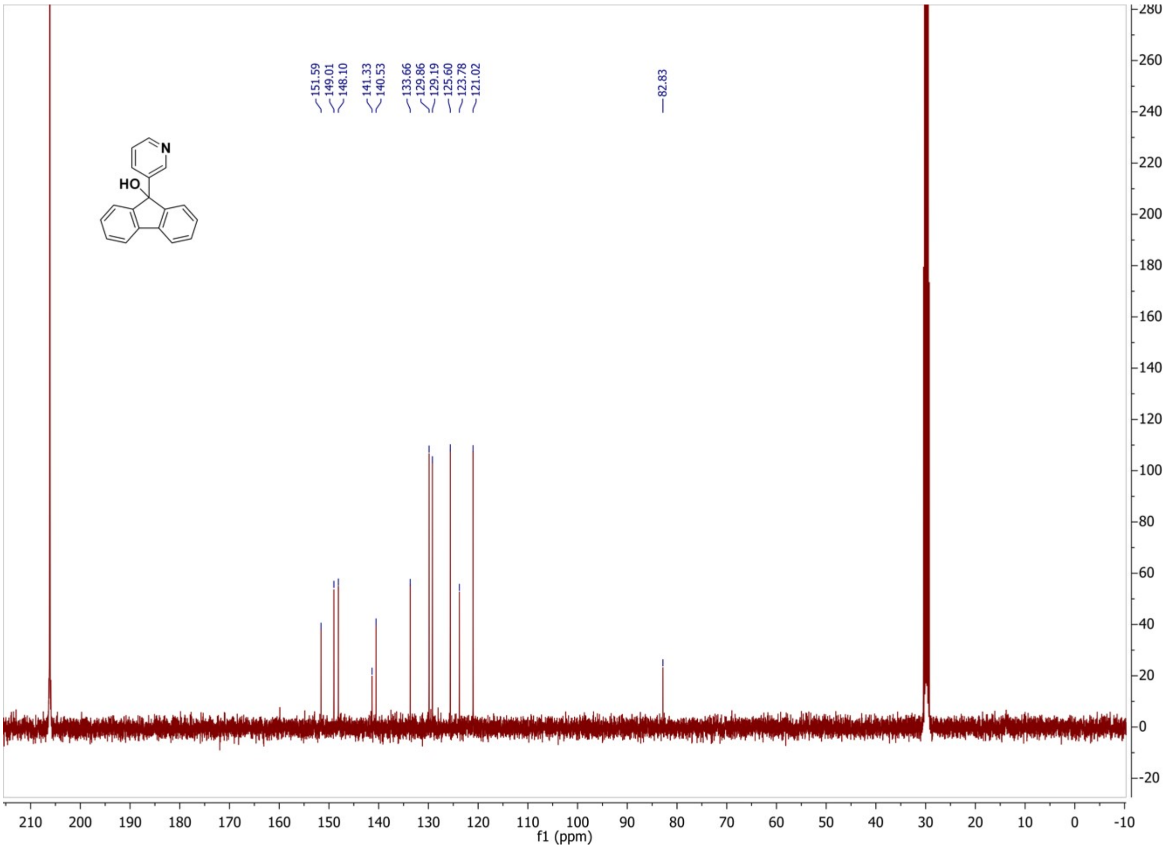

Figure S40. $^{13}$C NMR spectrum of 9-(pyridin-3-yl)-9*H*-fluoren-9-ol (**MYOS_00132**, 101 MHz, Acetone-$d_6$).

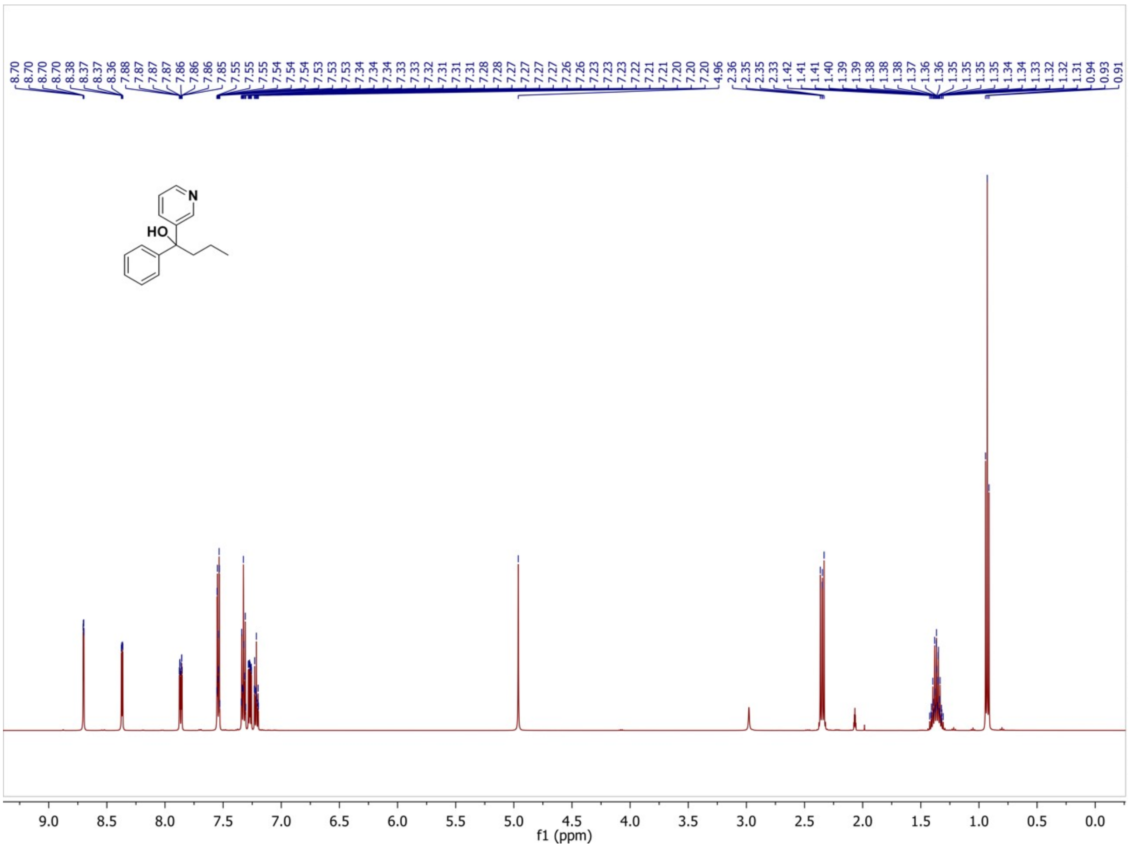


Figure S41. $^{1}$H NMR spectrum of 1-phenyl-1-(pyridin-3-yl)butan-1-ol (**MYOS_00133**, 500 MHz, Acetone-$d_6$).

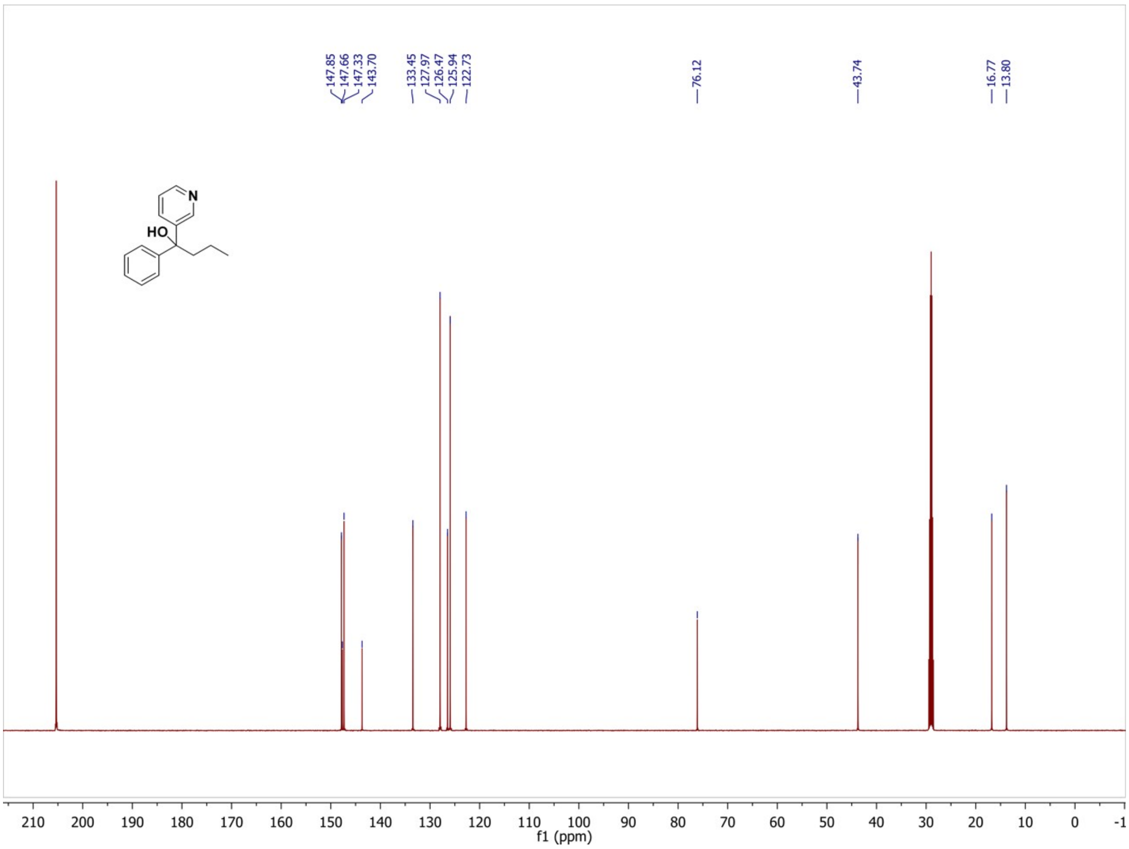


Figure S42. $^{13}$C NMR spectrum of 1-phenyl-1-(pyridin-3-yl)butan-1-ol (**MYOS_00133**, 126 MHz, Acetone-$d_6$).

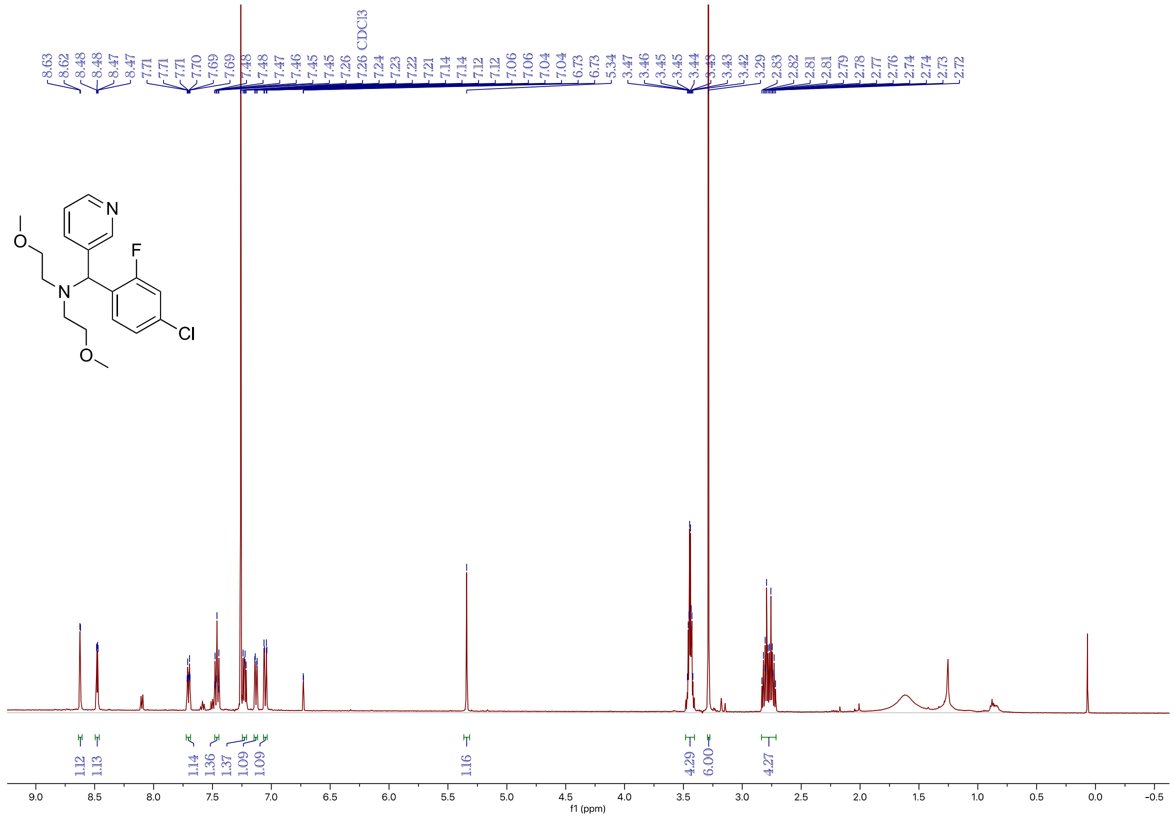

Figure S43. $^{1}$H NMR spectrum of *N*-((4-chloro-2-fluorophenyl)(pyridin-3-yl)methyl)-2-methoxy-*N*-(2-methoxyethyl)ethan-1-amine (**MYOS_00135**, 500 MHz, $CDCl_3$).

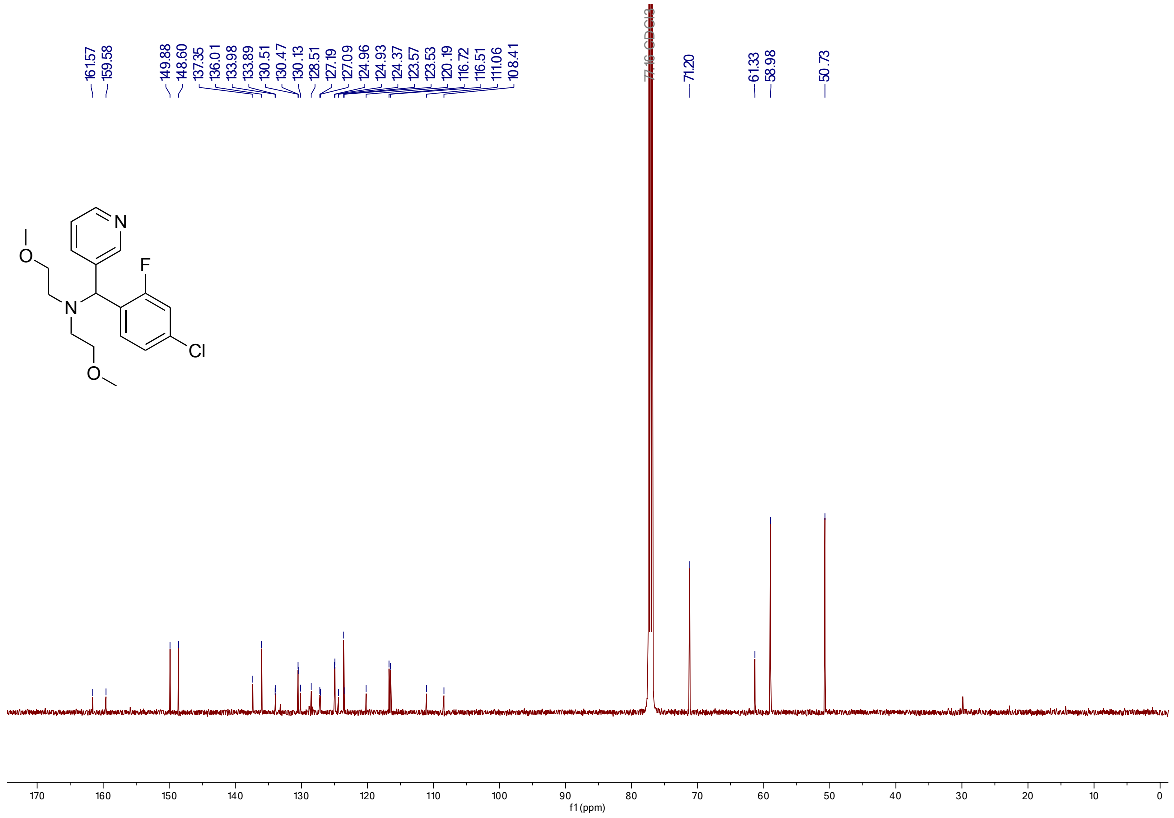

Figure S44. $^{13}$C NMR spectrum of *N*-((4-chloro-2-fluorophenyl)(pyridin-3-yl)methyl)-2-methoxy-*N*-(2-methoxyethyl)ethan-1-amine (**MYOS_00135**, 126 MHz, $CDCl_3$).

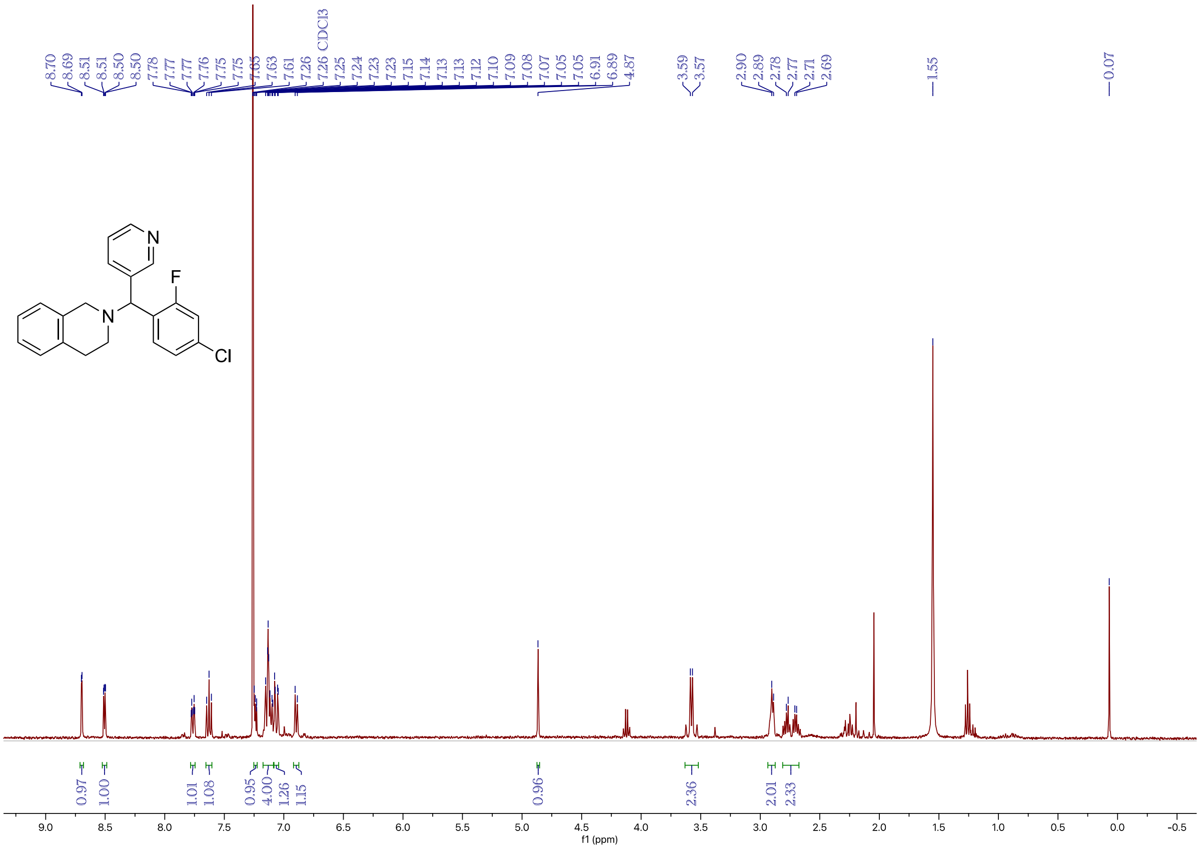

Figure S45. $^{1}$H NMR spectrum of 2-((4-chloro-2-fluorophenyl)(pyridin-3-yl)methyl)-1,2,3,4-tetrahydroisoquinoline (**MYOS_00136**, 400 MHz, $CDCl_3$)

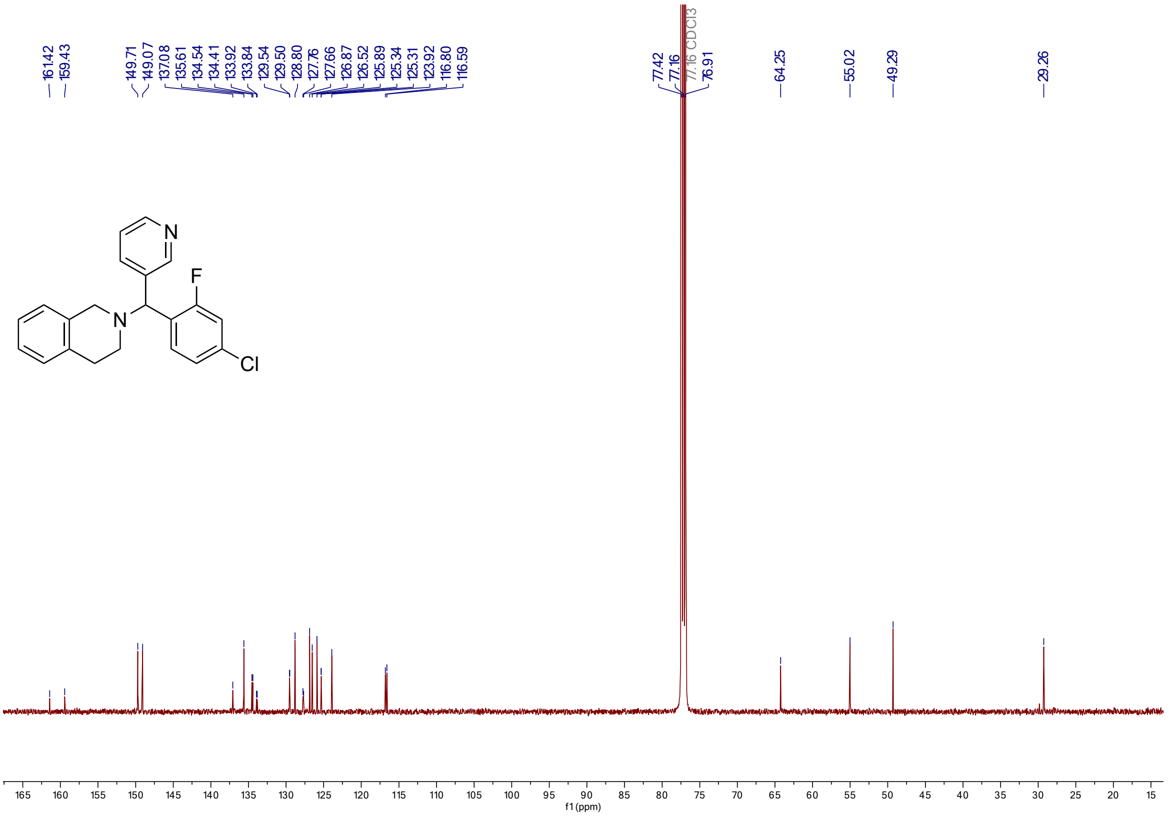

Figure S46. $^{13}$C NMR spectrum of 2-((4-chloro-2-fluorophenyl)(pyridin-3-yl)methyl)-1,2,3,4-tetrahydroisoquinoline (**MYOS_00136**, 126 MHz, $CDCl_3$).

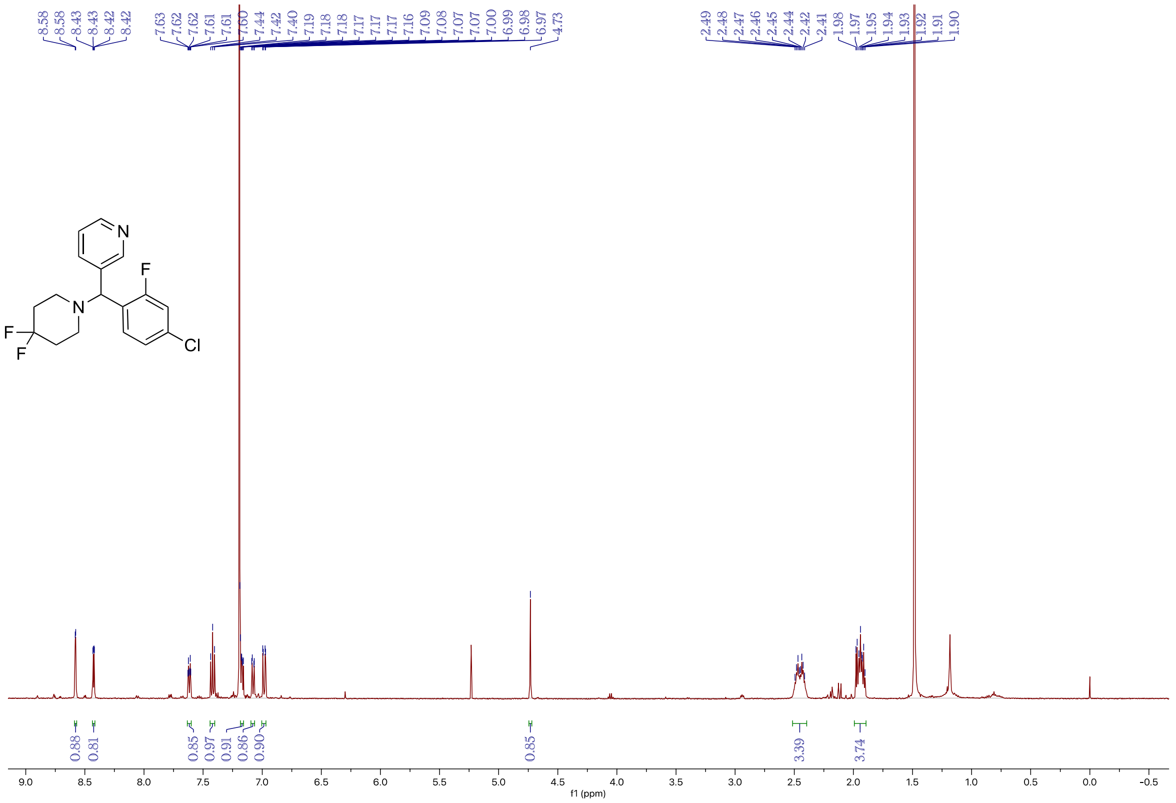


Figure S47. $^{1}$H NMR spectrum of 3-((4-chloro-2-fluorophenyl)(4,4-difluoropiperidin-1-yl)methyl)pyridine (**MYOS_00137**, 500 MHz, $CDCl_3$).

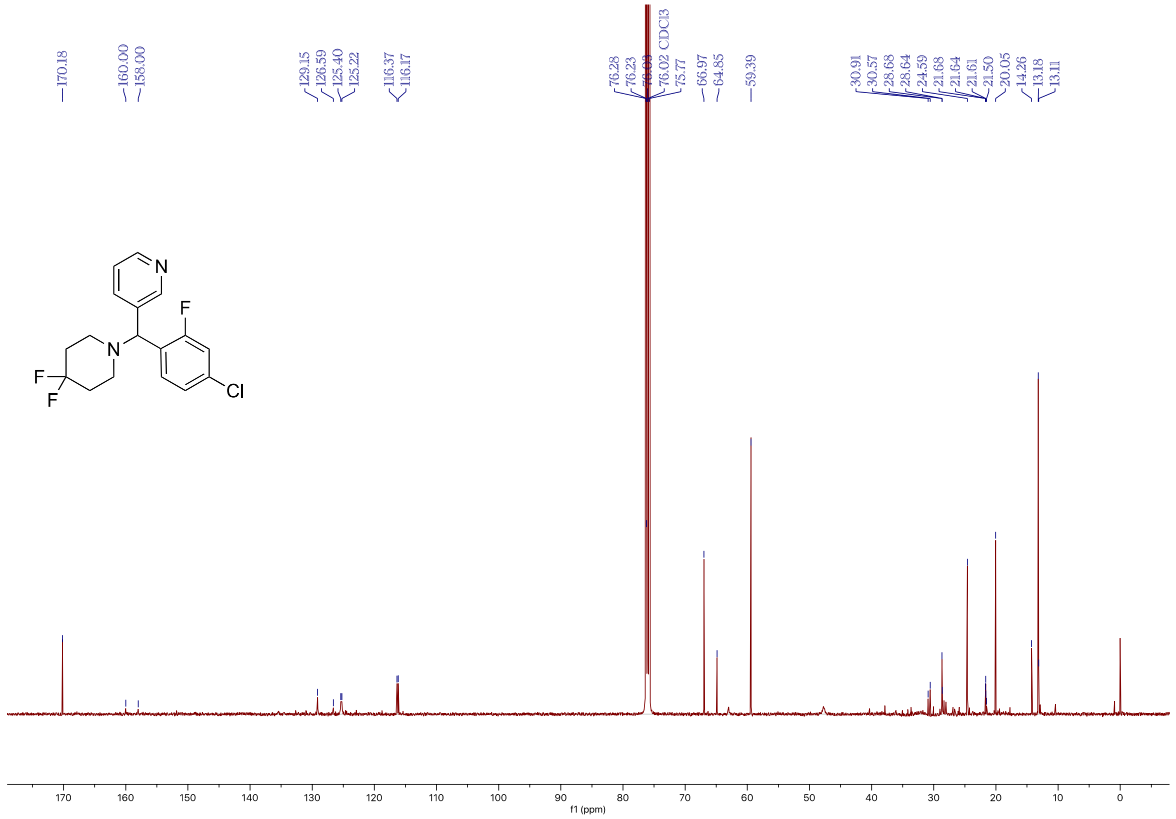


Figure S48. $^{13}$C NMR spectrum of 3-((4-chloro-2-fluorophenyl)(4,4-difluoropiperidin-1-yl)methyl)pyridine (**MYOS_00137**, 126 MHz, $CDCl_3$).

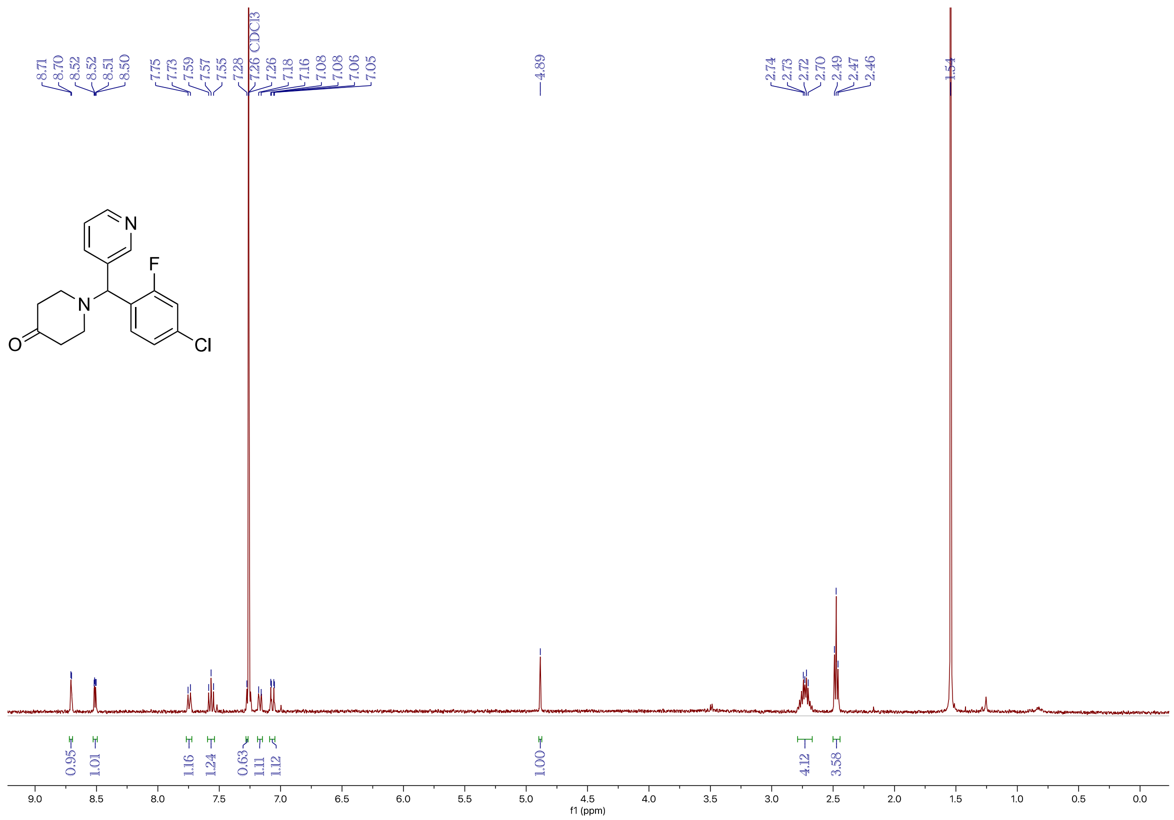

Figure S49. $^{1}$H NMR spectrum of 1-((4-chloro-2-fluorophenyl)(pyridin-3-yl)methyl)piperidin-4-one (**MYOS_00139**, 400 MHz, $CDCl_3$).

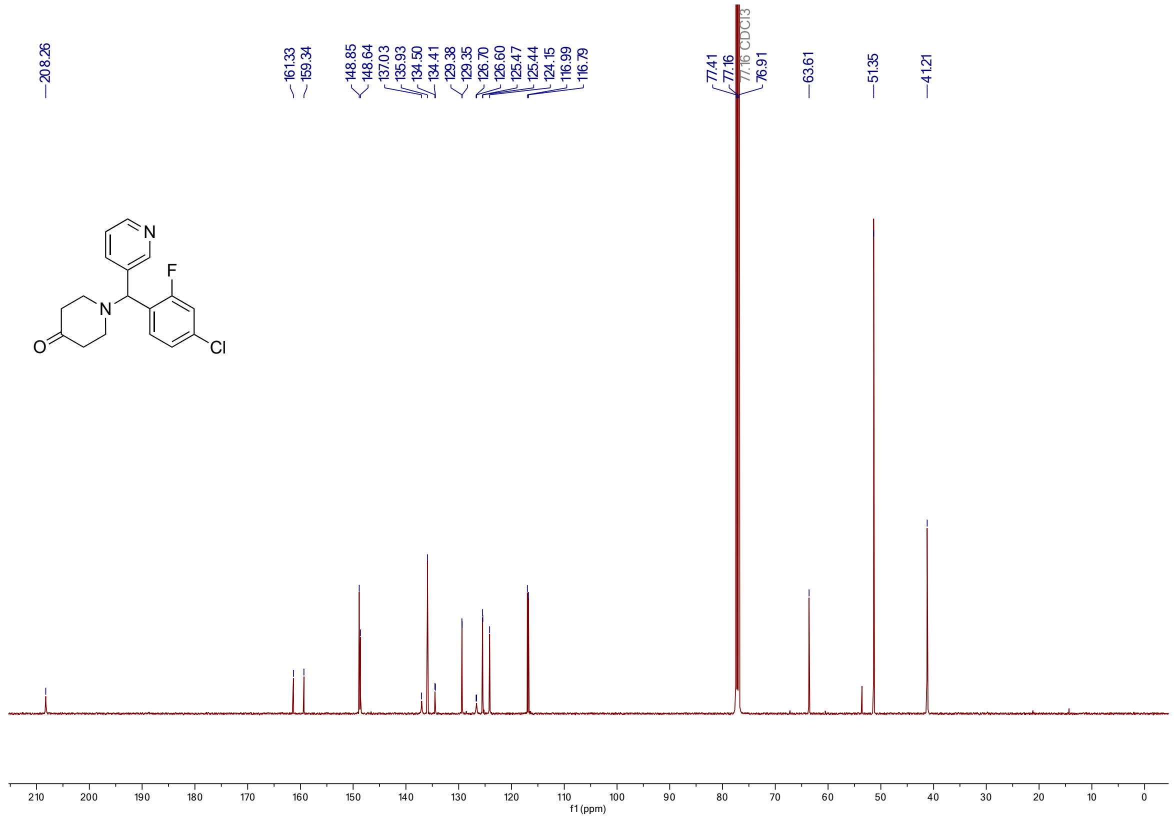

Figure S50. $^{13}$C NMR spectrum of 1-((4-chloro-2-fluorophenyl)(pyridin-3-yl)methyl)piperidin-4-one (**MYOS_00139**, 126 MHz, $CDCl_3$).

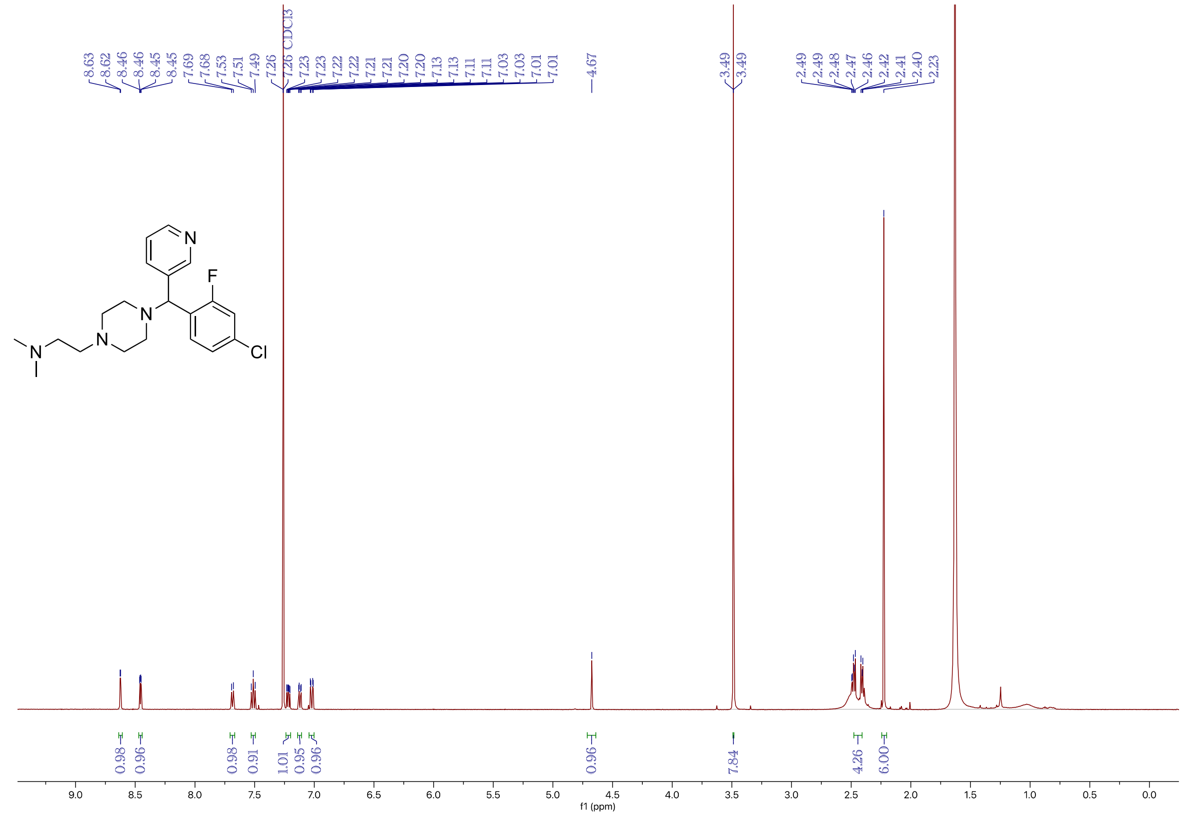

Figure S51. $^{1}$H NMR spectrum of 2-(4-((4-chloro-2-fluorophenyl)(pyridin-3-yl)methyl)piperazin-1-yl)-*N*,*N*-dimethylethan-1-amine (**MYOS_00140**, 500 MHz, $CDCl_3$).

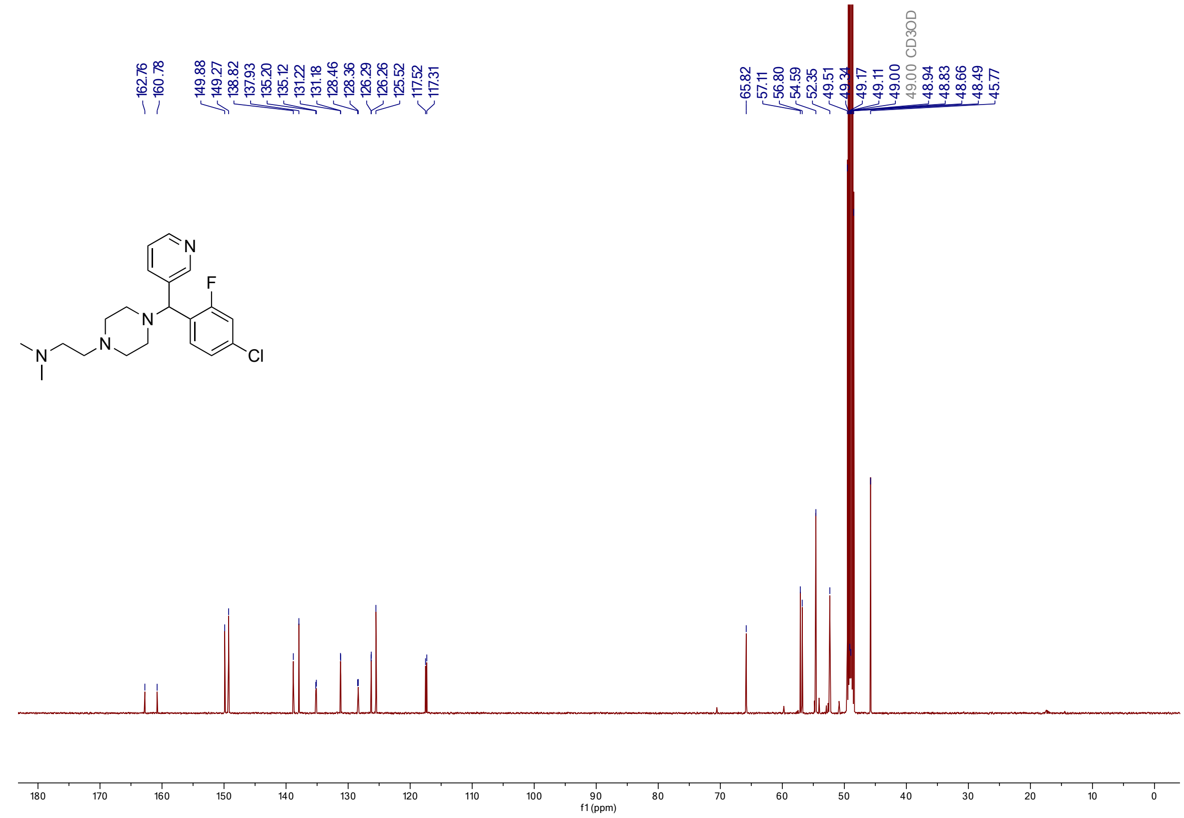

Figure S52. $^{13}$C NMR spectrum of 2-(4-((4-chloro-2-fluorophenyl)(pyridin-3-yl)methyl)piperazin-1-yl)-*N*,*N*-dimethylethan-1-amine (**MYOS_00140**, 126 MHz, Methanol-$d_4$).

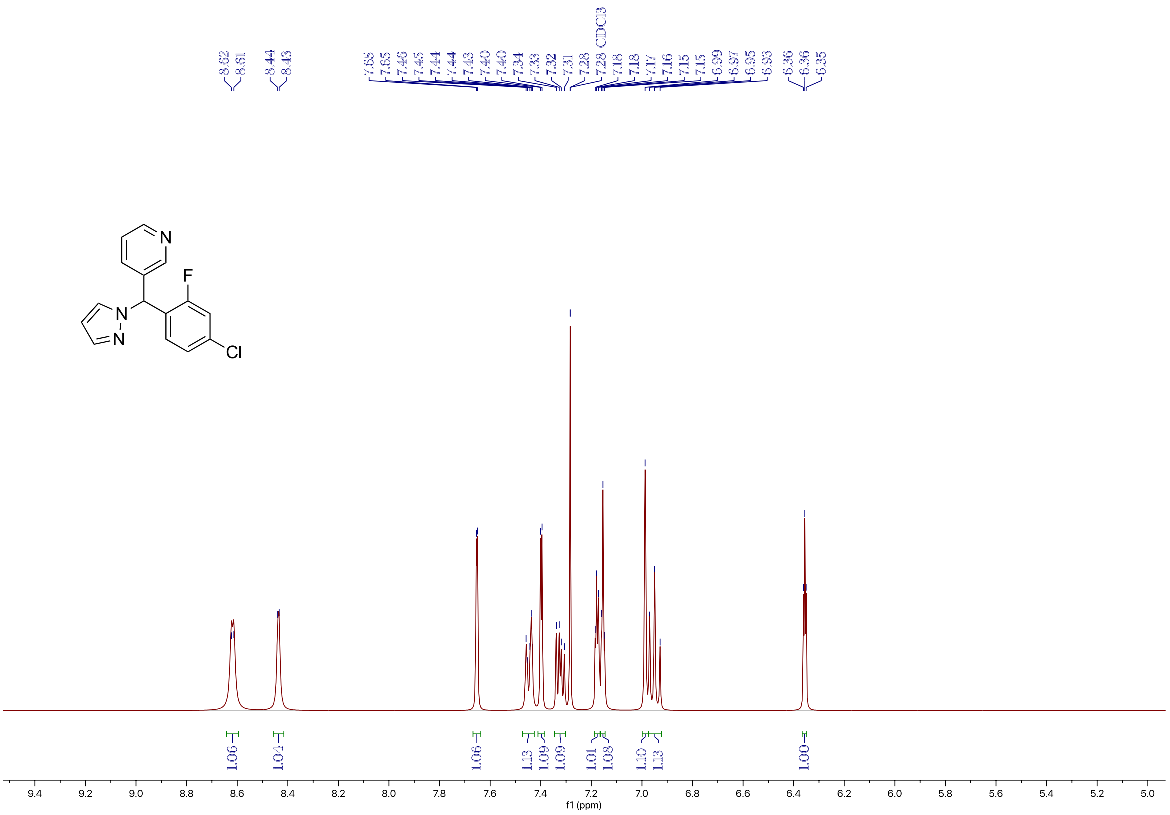

Figure S53. $^{1}$H NMR spectrum of 3-((4-chloro-2-fluorophenyl)(1H-pyrazol-1-yl)methyl)pyridine (**MYOS_00141**, 400 MHz, $CDCl_3$).

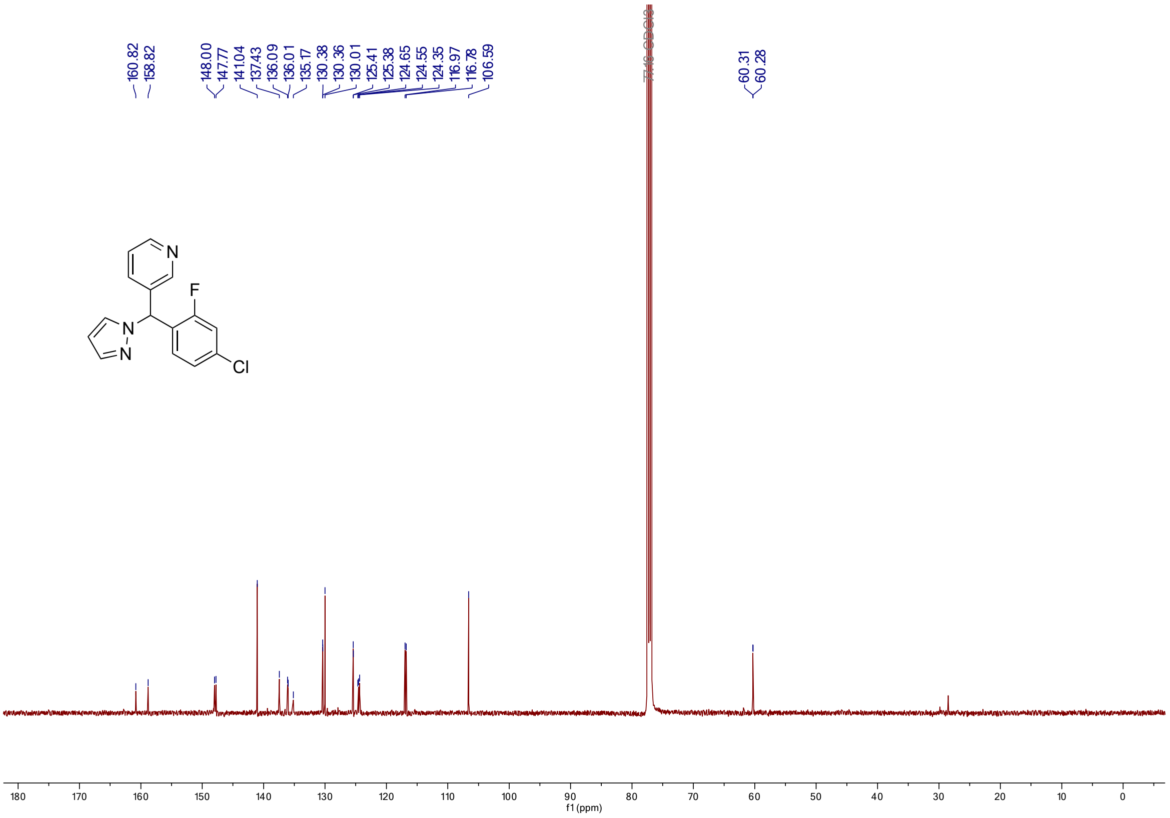

Figure S54. $^{13}$C NMR spectrum of 3-((4-chloro-2-fluorophenyl)(1H-pyrazol-1-yl)methyl)pyridine (**MYOS_00141**, 126 MHz, $CDCl_3$).

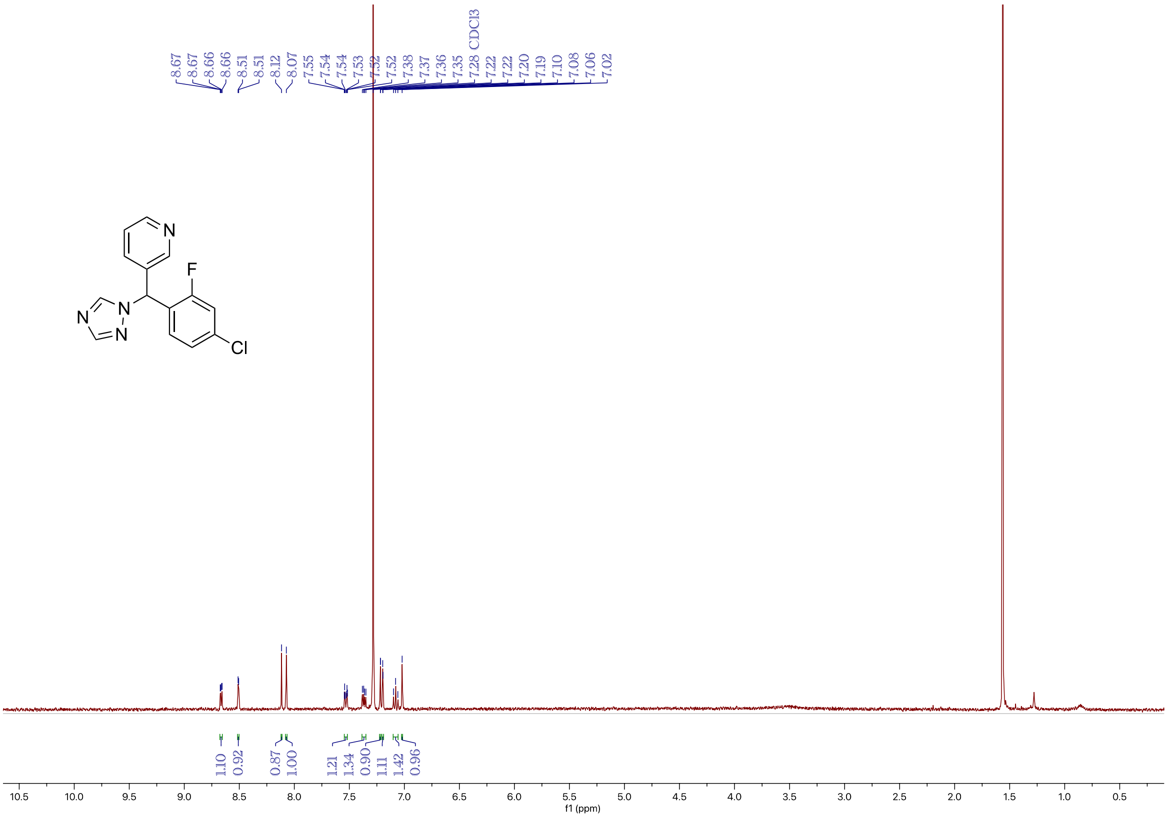


Figure S55. $^{1}$H NMR spectrum of 3-((4-chloro-2-fluorophenyl)(1H-1,2,4-triazol-1-yl)methyl)pyridine (**MYOS_00142**, 400 MHz, $CDCl_3$).

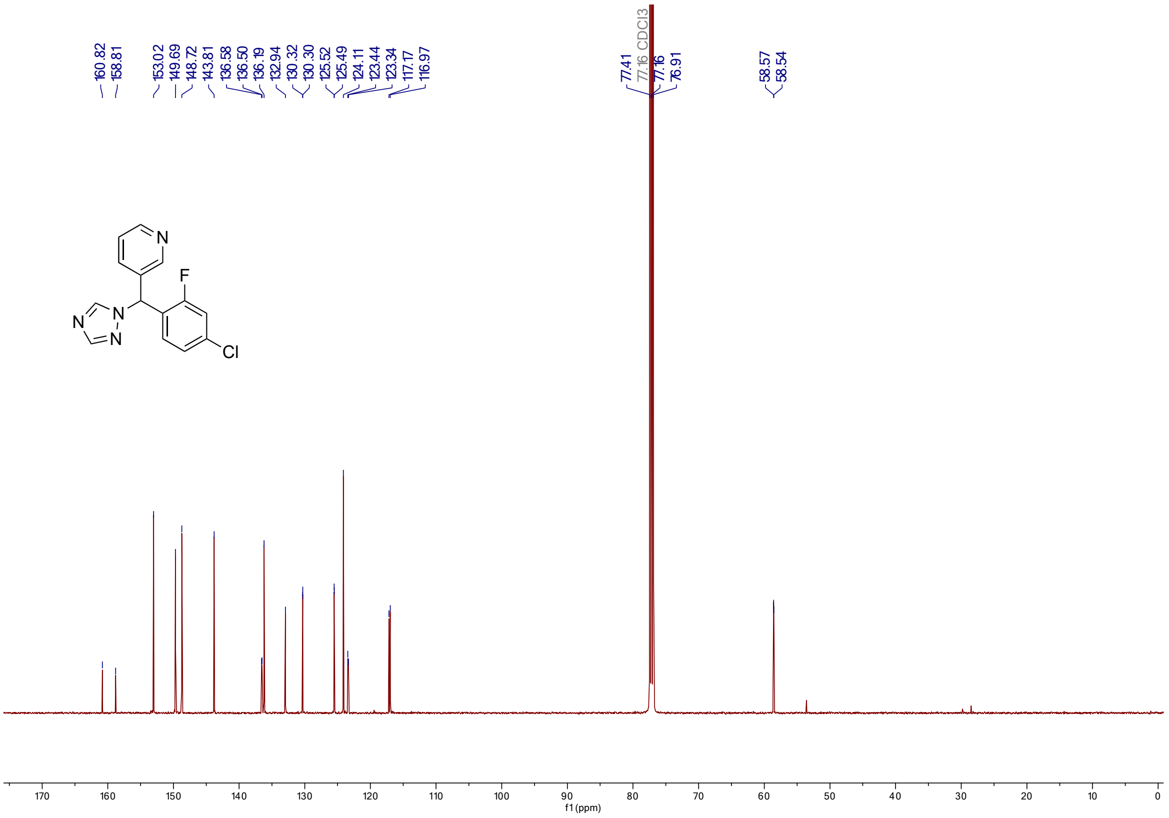


Figure S56. $^{13}$C NMR spectrum of 3-((4-chloro-2-fluorophenyl)(1H-1,2,4-triazol-1-yl)methyl)pyridine (**MYOS_00142**, 126 MHz, $CDCl_3$).

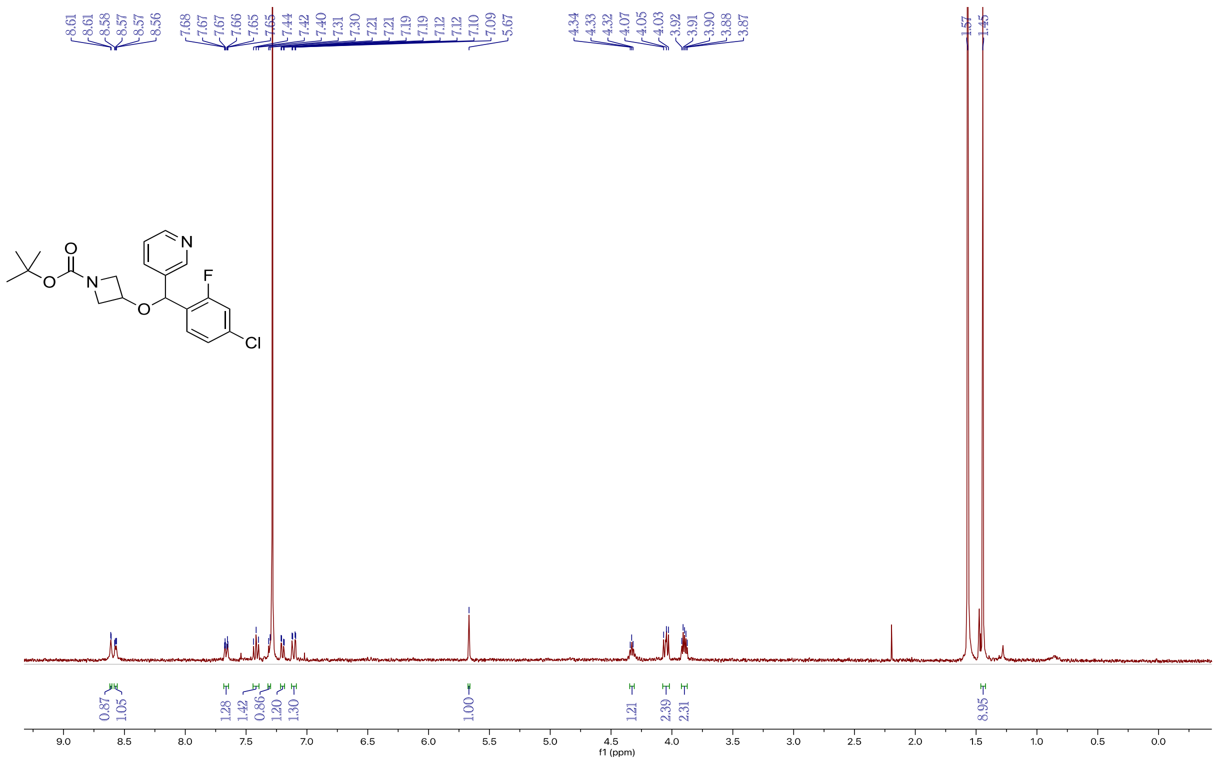

Figure S57. $^{1}H$ NMR spectrum of *tert*-butyl 3-((4-chloro-2-fluorophenyl)(pyridin-3-yl)methoxy)azetidine-1-carboxylate (**MYOS_00143**, 400 MHz, $CDCl_3$).

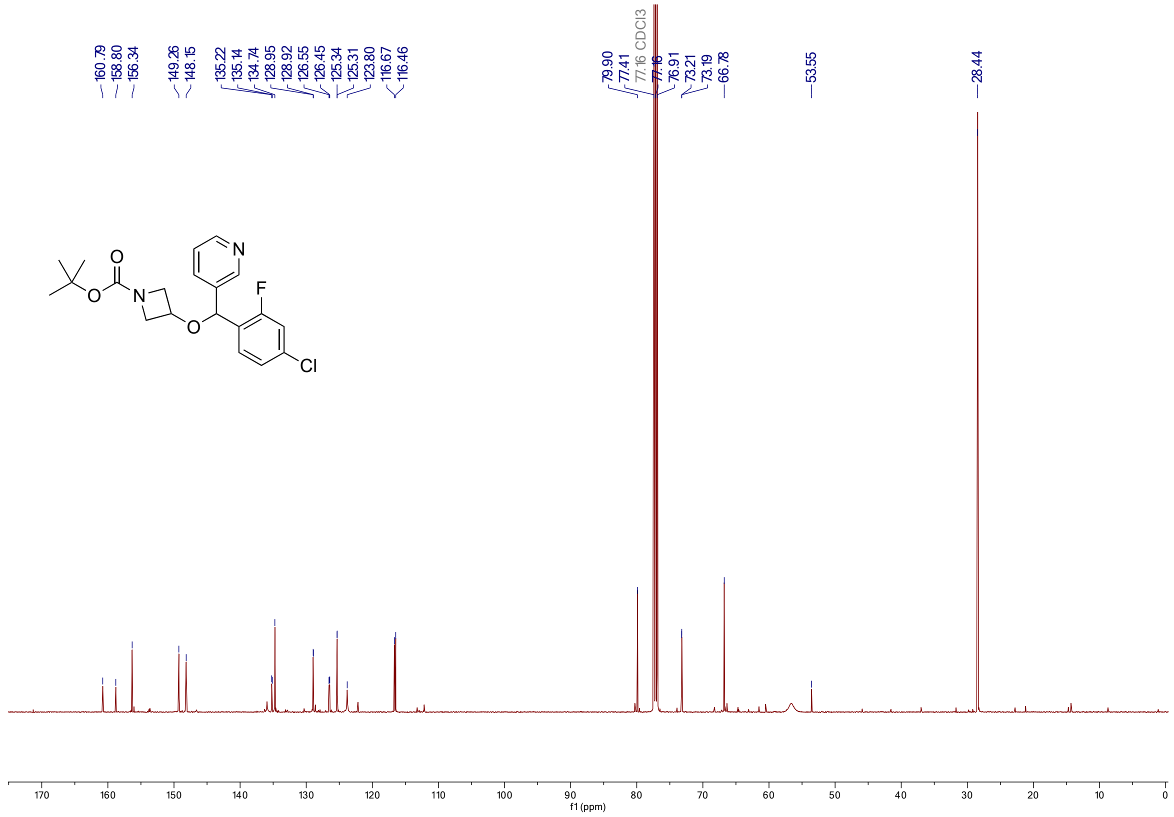

Figure S58. $^{13}C$ NMR spectrum of *tert*-butyl 3-((4-chloro-2-fluorophenyl)(pyridin-3-yl)methoxy)azetidine-1-carboxylate (**MYOS_00143**, 126 MHz, $CDCl_3$).

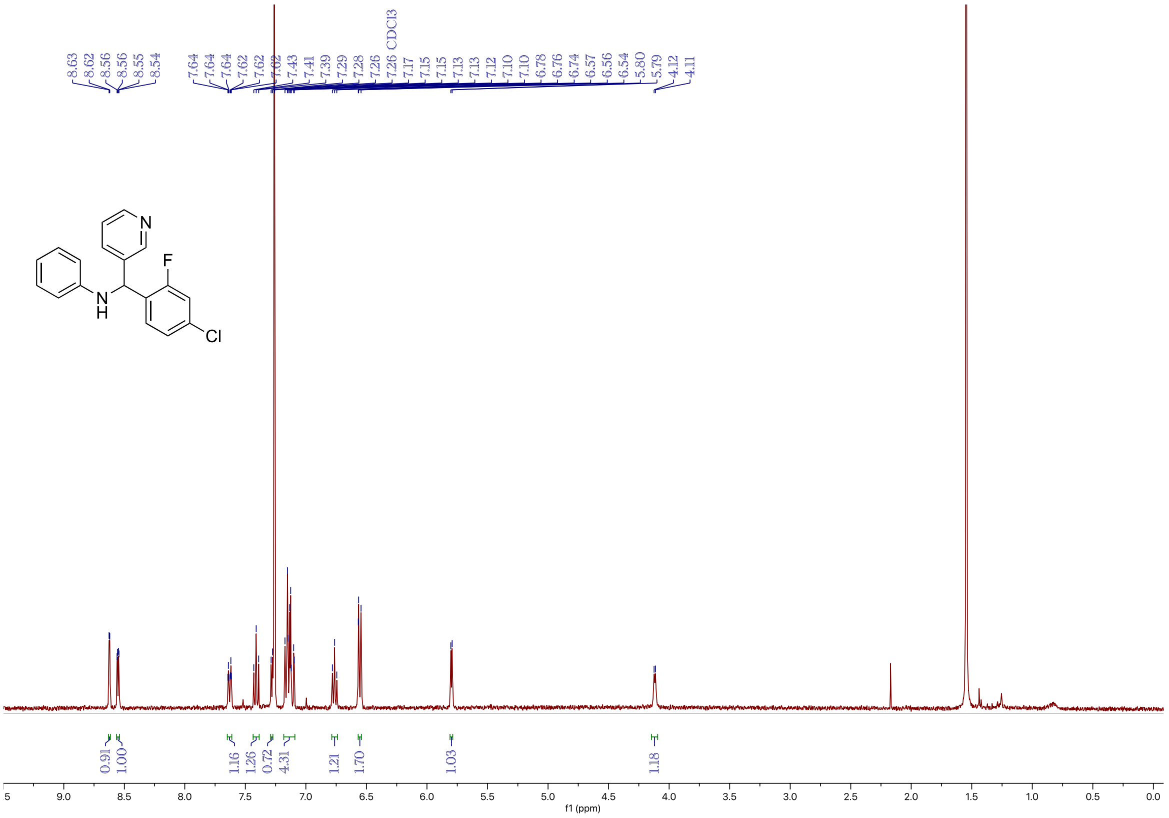


Figure S59. $^{1}$H NMR spectrum of *N*-((4-chloro-2-fluorophenyl)(pyridin-3-yl)methyl)aniline (**MYOS_00144**, 400 MHz, $CDCl_3$).

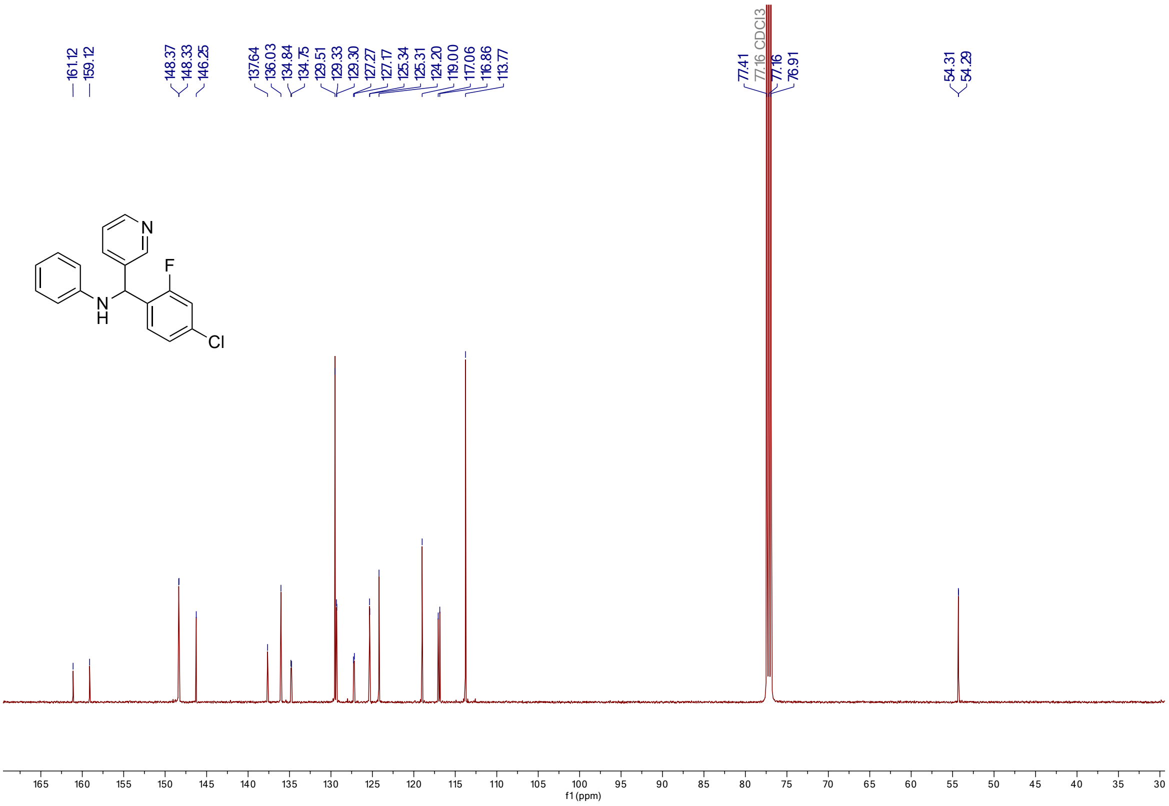


Figure S60. $^{13}$C NMR spectrum of *N*-((4-chloro-2-fluorophenyl)(pyridin-3-yl)methyl)aniline (**MYOS_00144**, 126 MHz, $CDCl_3$).

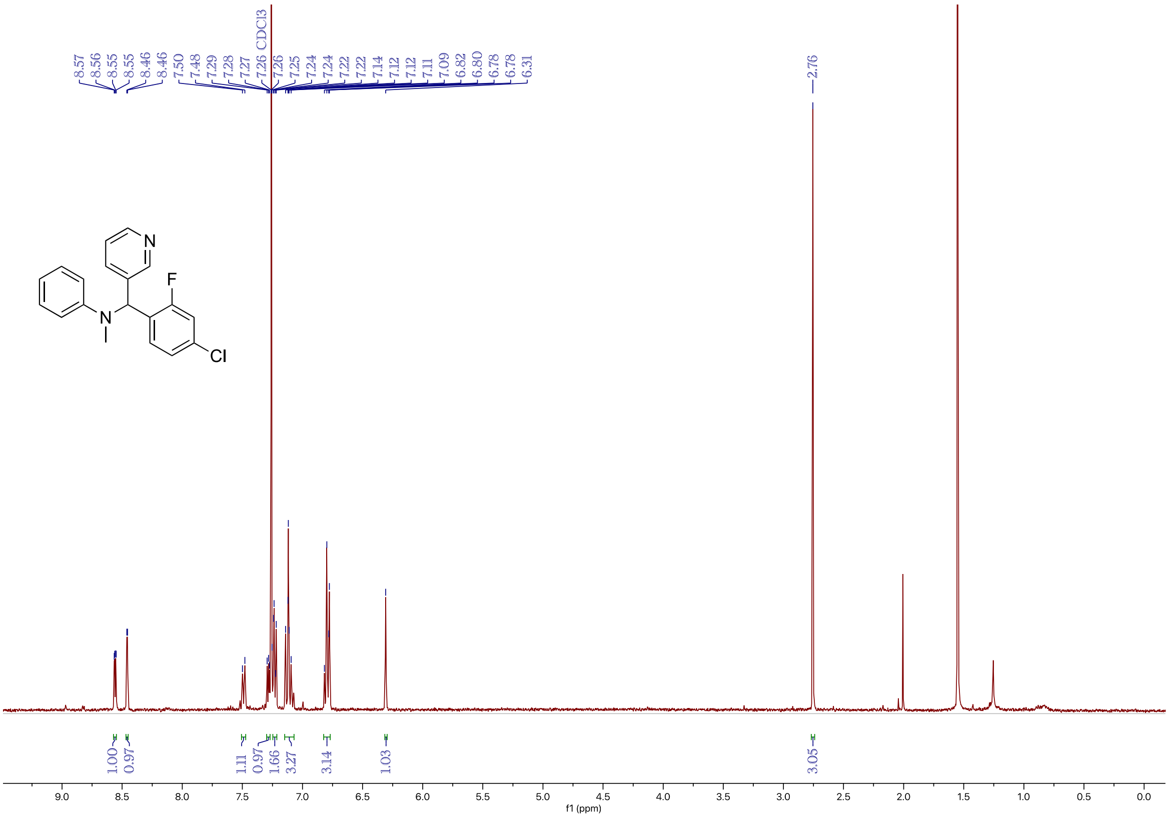

Figure S61. $^{1}$H NMR spectrum of *N*-((4-chloro-2-fluorophenyl)(pyridin-3-yl)methyl)-*N*-methylaniline (**MYOS_00145**, 400 MHz, $CDCl_3$).

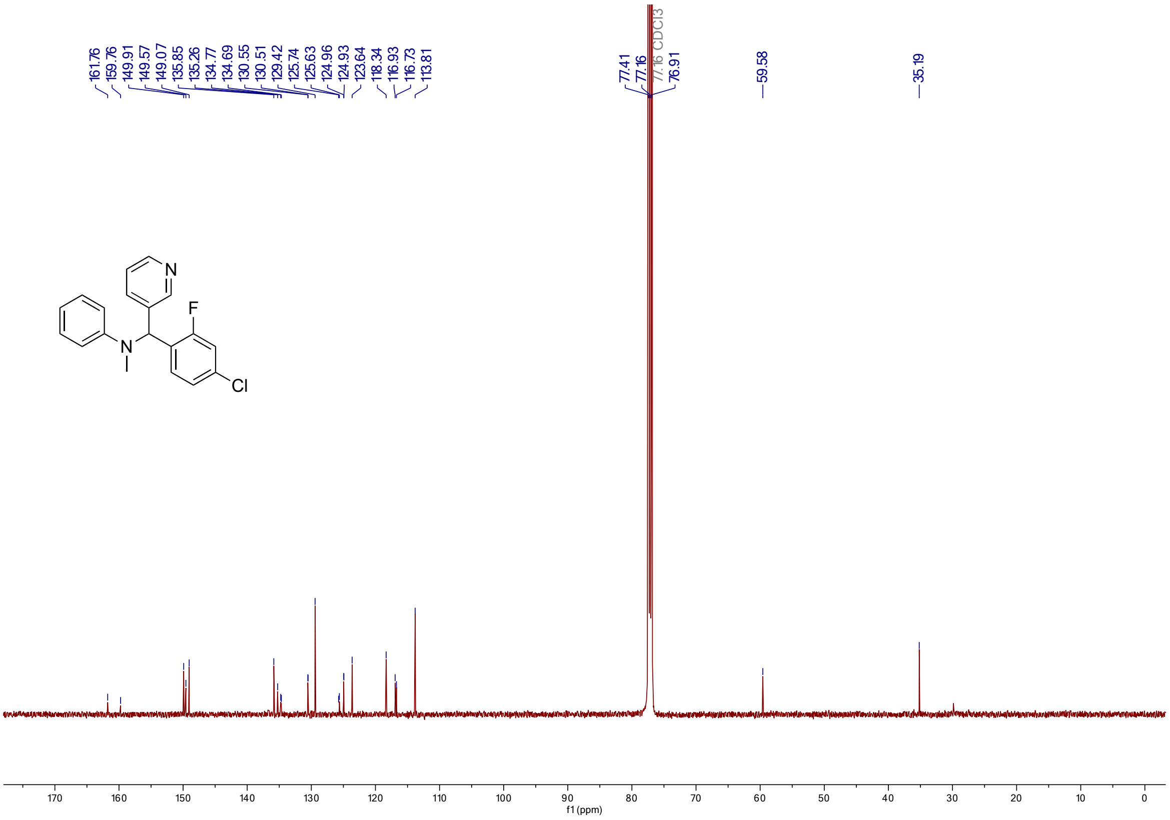

Figure S62. $^{13}$C NMR spectrum of *N*-((4-chloro-2-fluorophenyl)(pyridin-3-yl)methyl)-*N*-methylaniline (**MYOS_00145**, 126 MHz, $CDCl_3$).

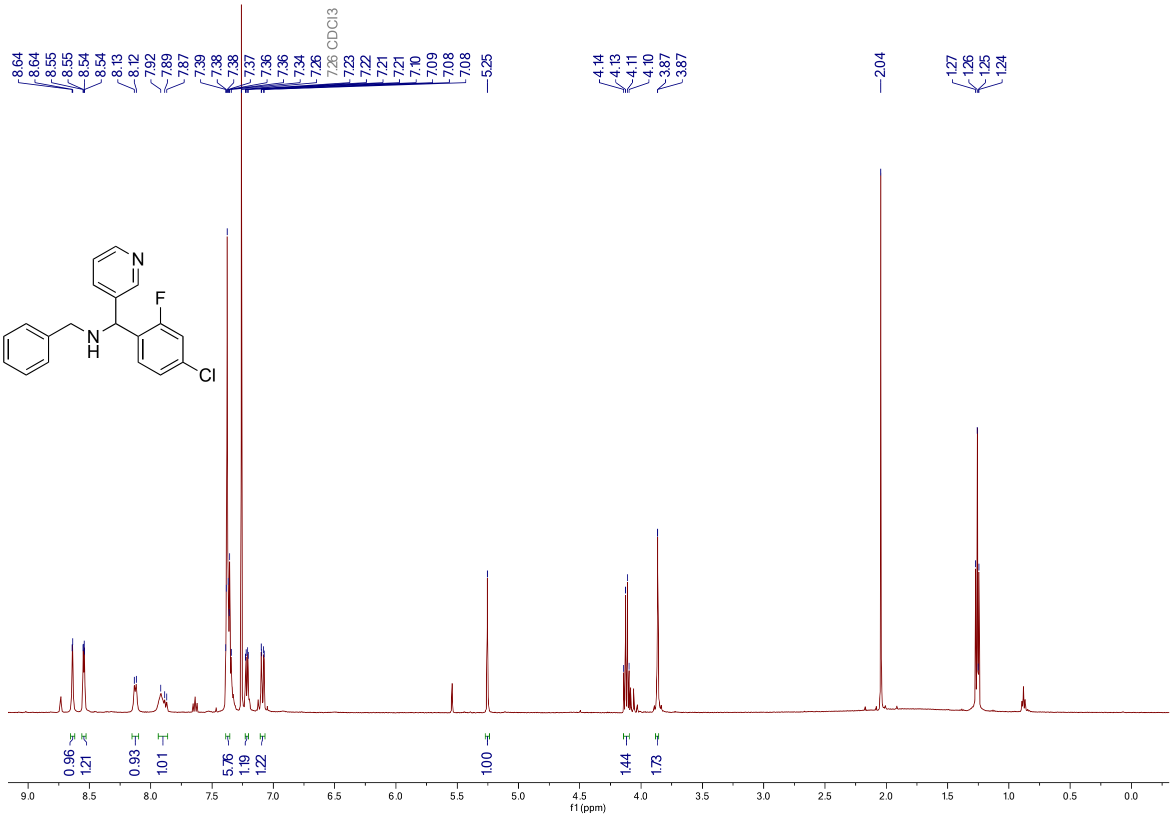


Figure S63. $^{1}$H NMR spectrum of *N*-benzyl-1-(4-chloro-2-fluorophenyl)-1-(pyridin-3-yl)methanamine (**MYOS_00147**, 500 MHz, $CDCl_3$).

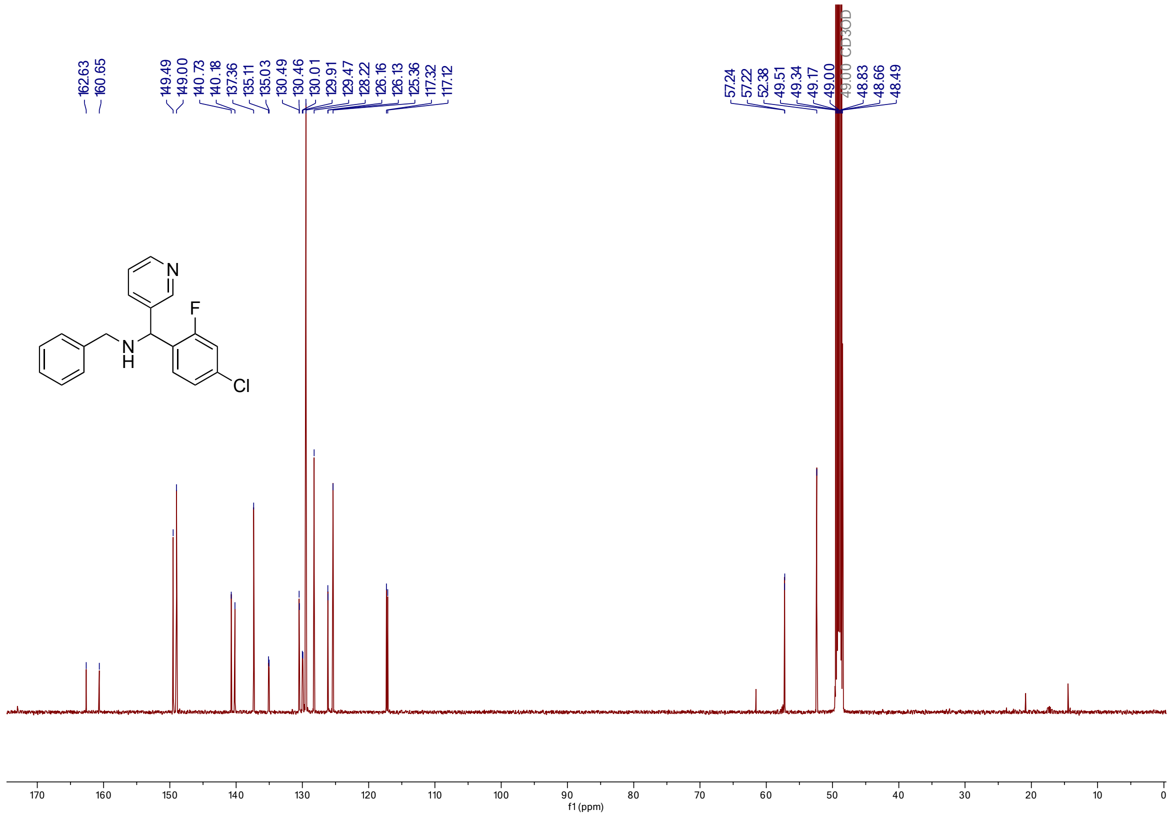


Figure S64. $^{13}$C NMR spectrum of *N*-benzyl-1-(4-chloro-2-fluorophenyl)-1-(pyridin-3-yl)methanamine (**MYOS_00147**, 126 MHz, Methanol-$d_4$).

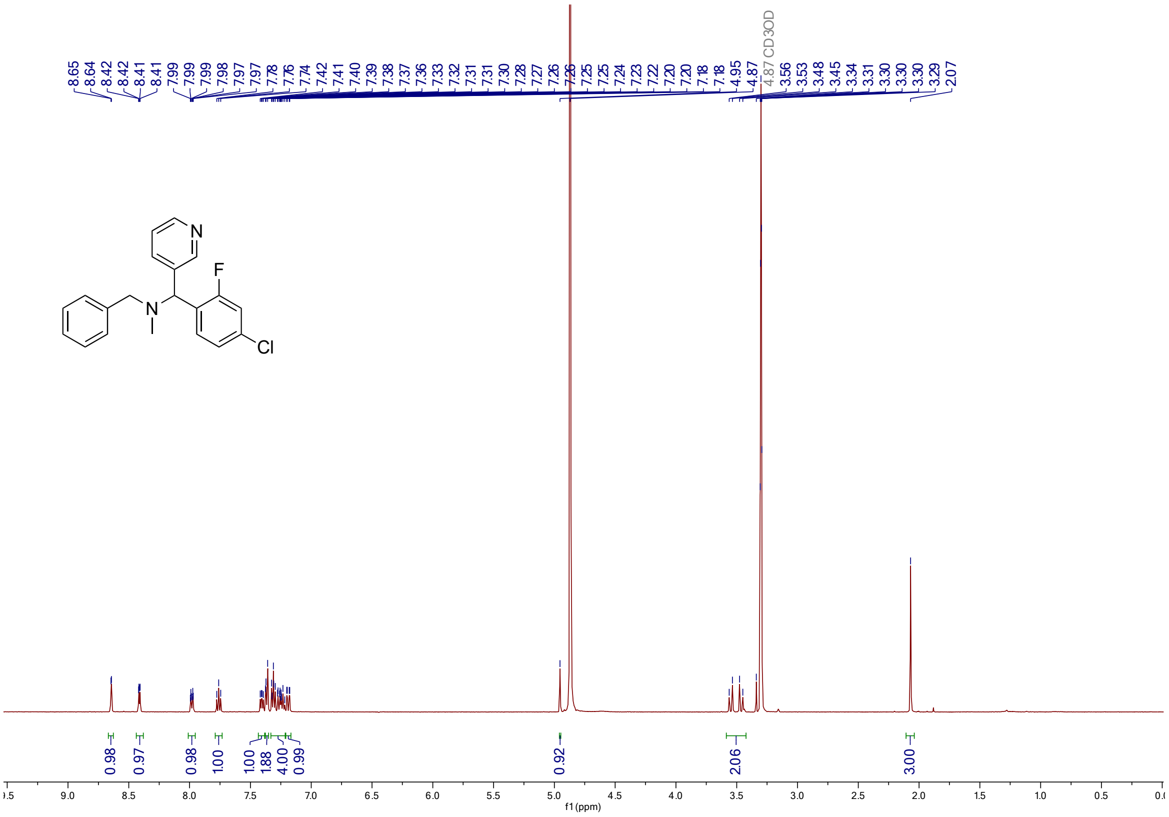


Figure S65. $^{1}$H NMR spectrum of *N*-benzyl-1-(4-chloro-2-fluorophenyl)-*N*-methyl-1-(pyridin-3-yl)methanamine (**MYOS_00148**, 500 MHz, Methanol-$d_4$).

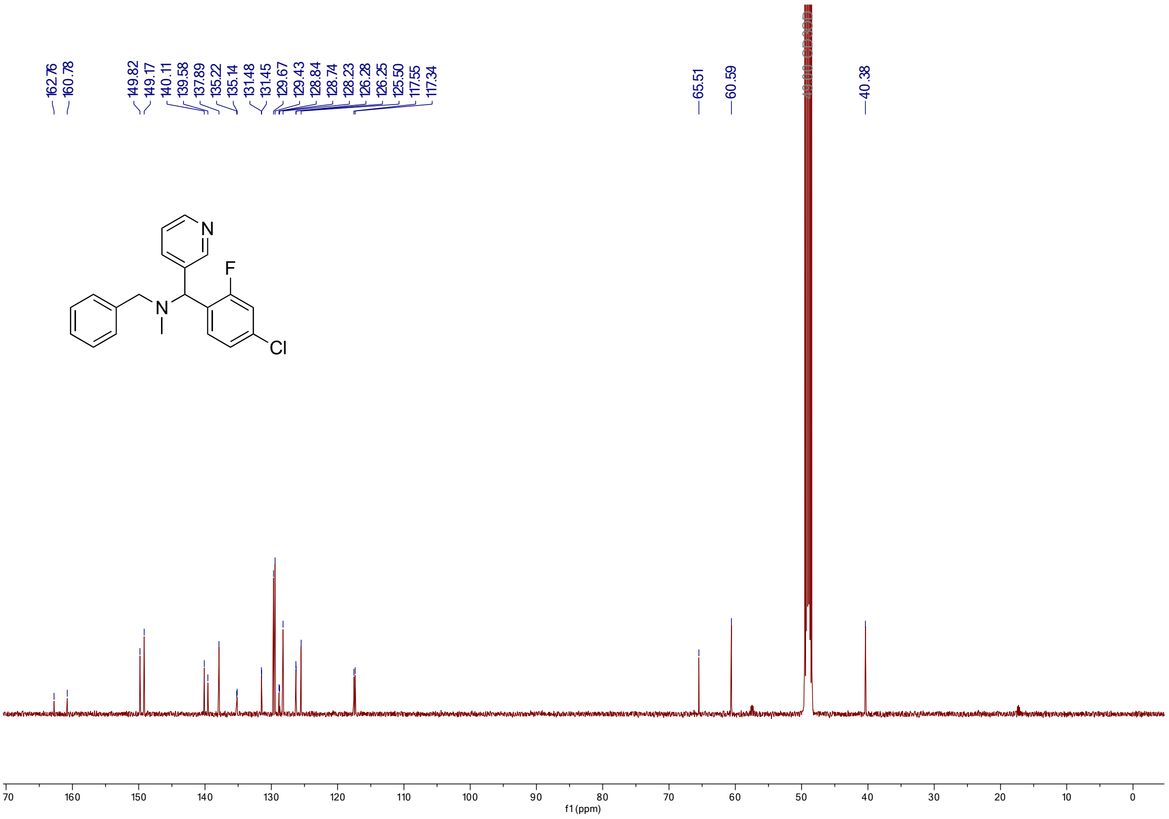


Figure S66. $^{13}$C NMR spectrum of *N*-benzyl-1-(4-chloro-2-fluorophenyl)-*N*-methyl-1-(pyridin-3-yl)methanamine (**MYOS_00148**, 126 MHz, Methanol-$d_4$).

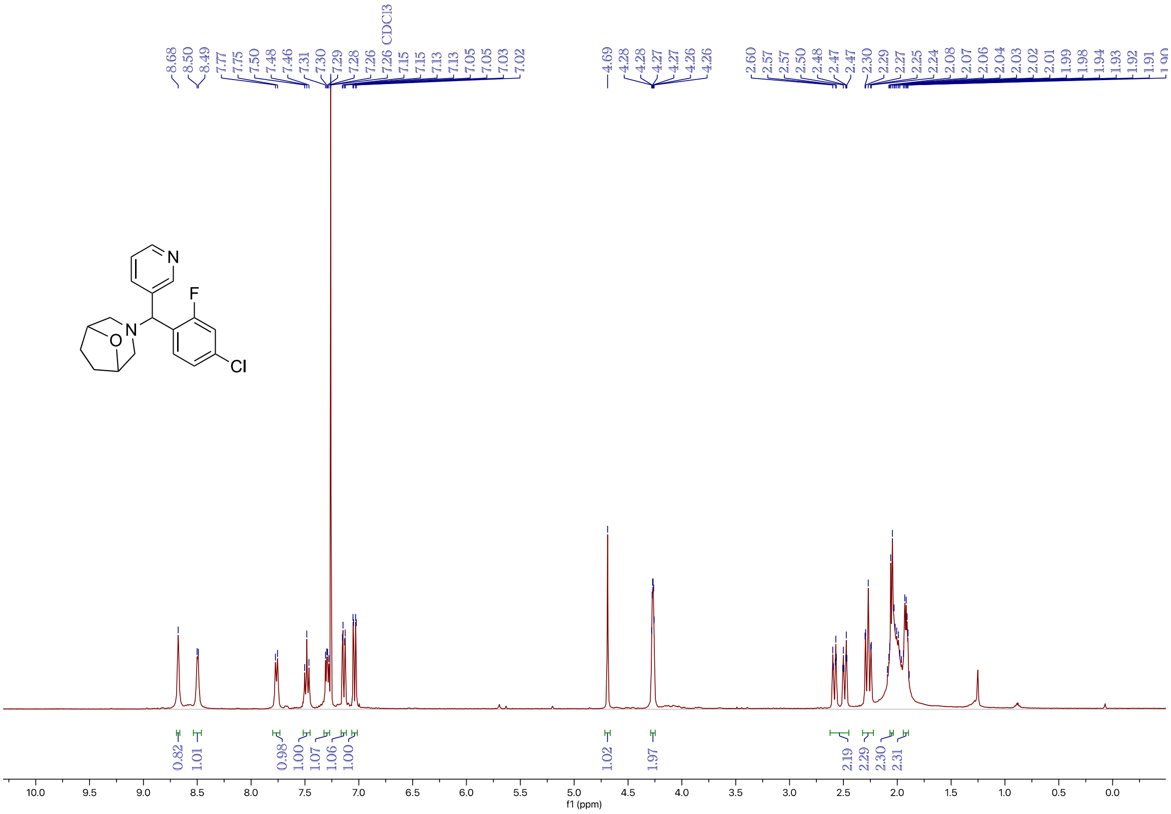


Figure S67. ¹H NMR spectrum of 3–((4–chloro–2–fluorophenyl)(pyridin–3–yl)methyl)–8–oxa–3–azabicyclo[3.2.1]octane (**MYOS_00149**, 400 MHz, $CDCl_3$).

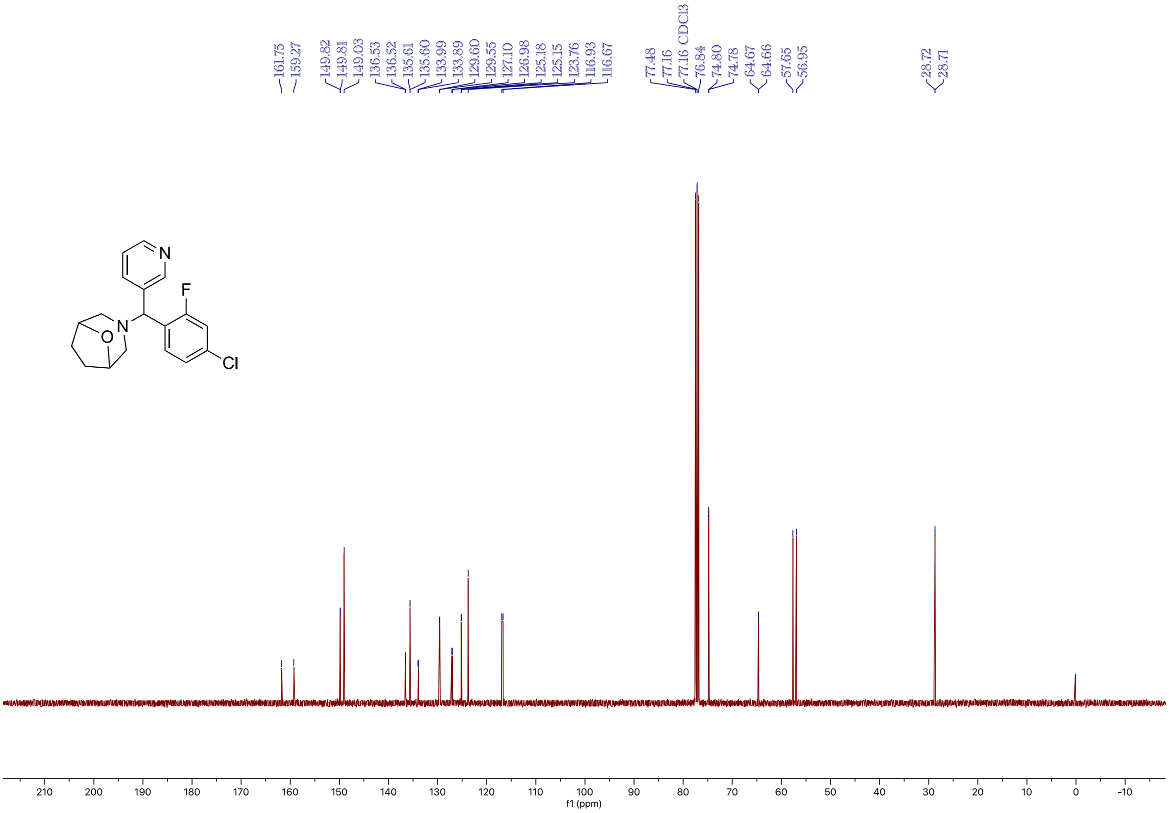


Figure S68. ¹³C NMR spectrum of 3–((4–chloro–2–fluorophenyl)(pyridin–3–yl)methyl)–8–oxa–3–azabicyclo[3.2.1]octane (**MYOS_00149**, 101 MHz, $CDCl_3$).

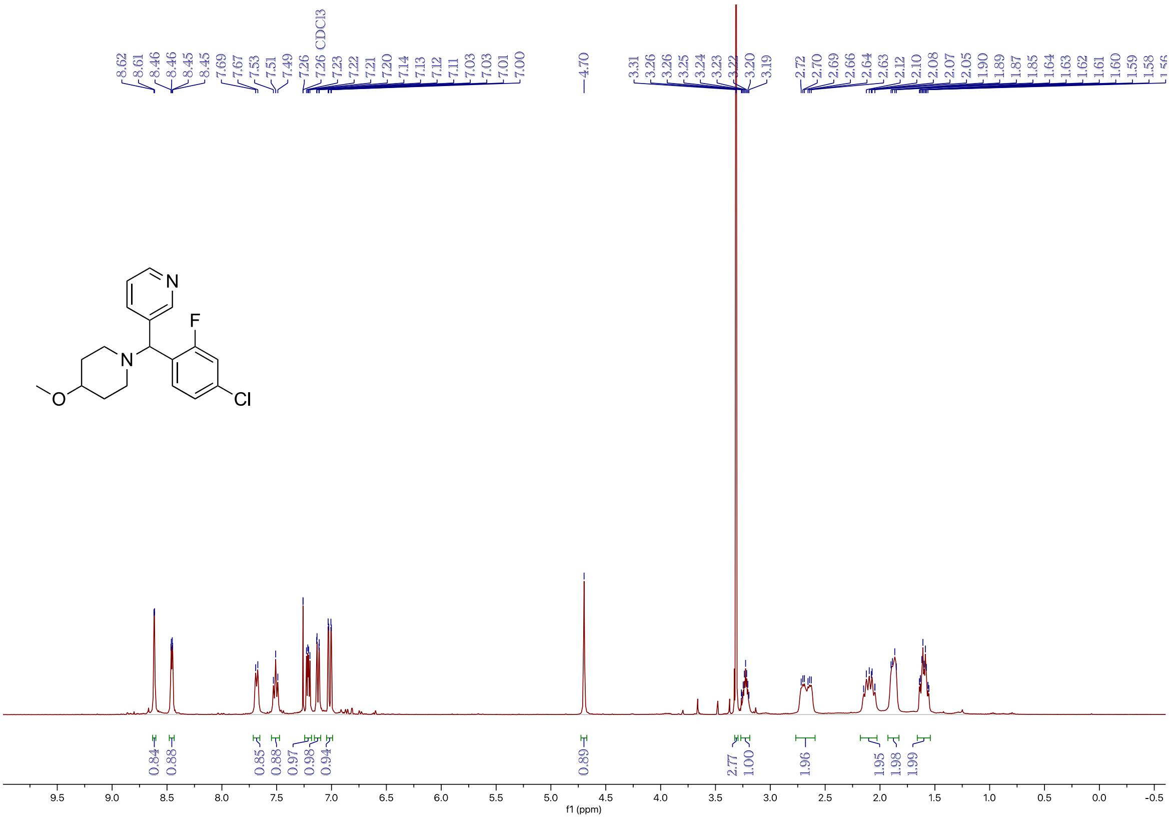


Figure S69. $^{1}$H NMR spectrum of 3–((4–chloro–2–fluorophenyl)(4–methoxypiperidin–1–yl)methyl)pyridine (**MYOS_00150**, 400 MHz, $CDCl_3$).

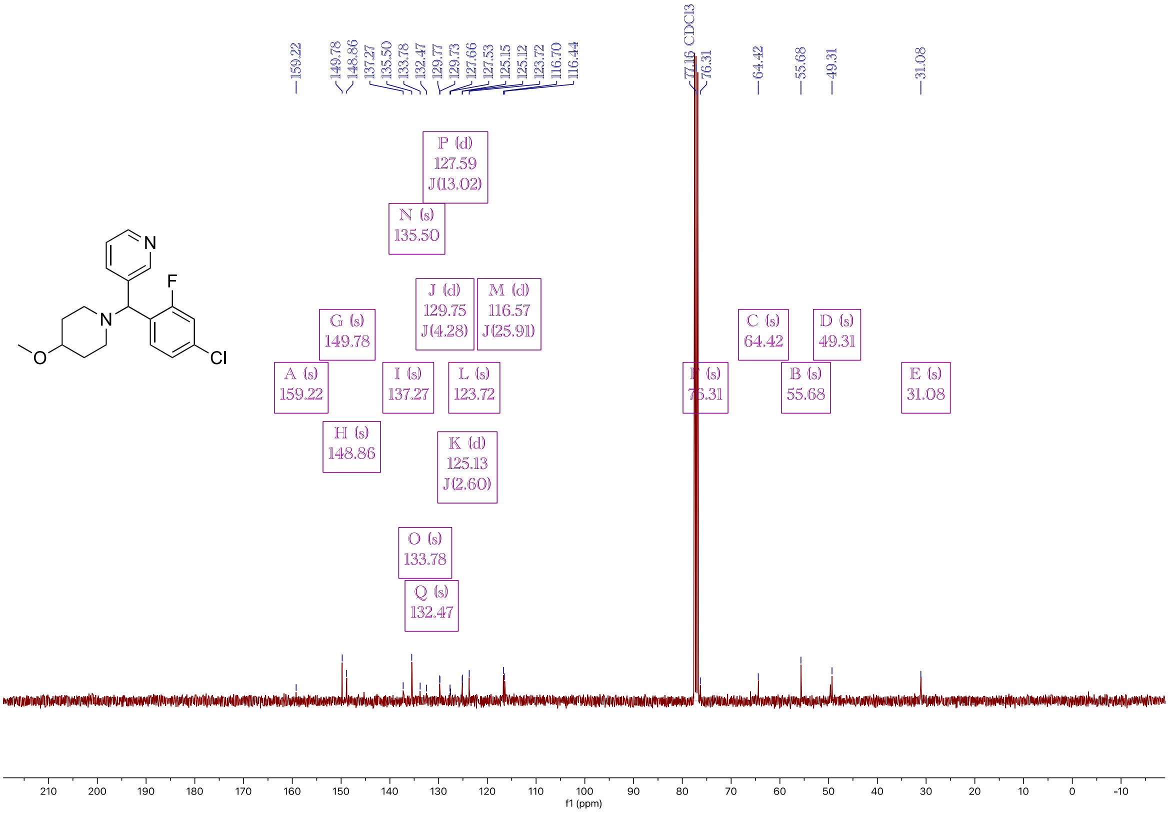


Figure S70. $^{13}$C NMR spectrum of 3–((4–chloro–2–fluorophenyl)(4–methoxypiperidin–1–yl)methyl)pyridine (**MYOS_00150**, 101 MHz, $CDCl_3$).

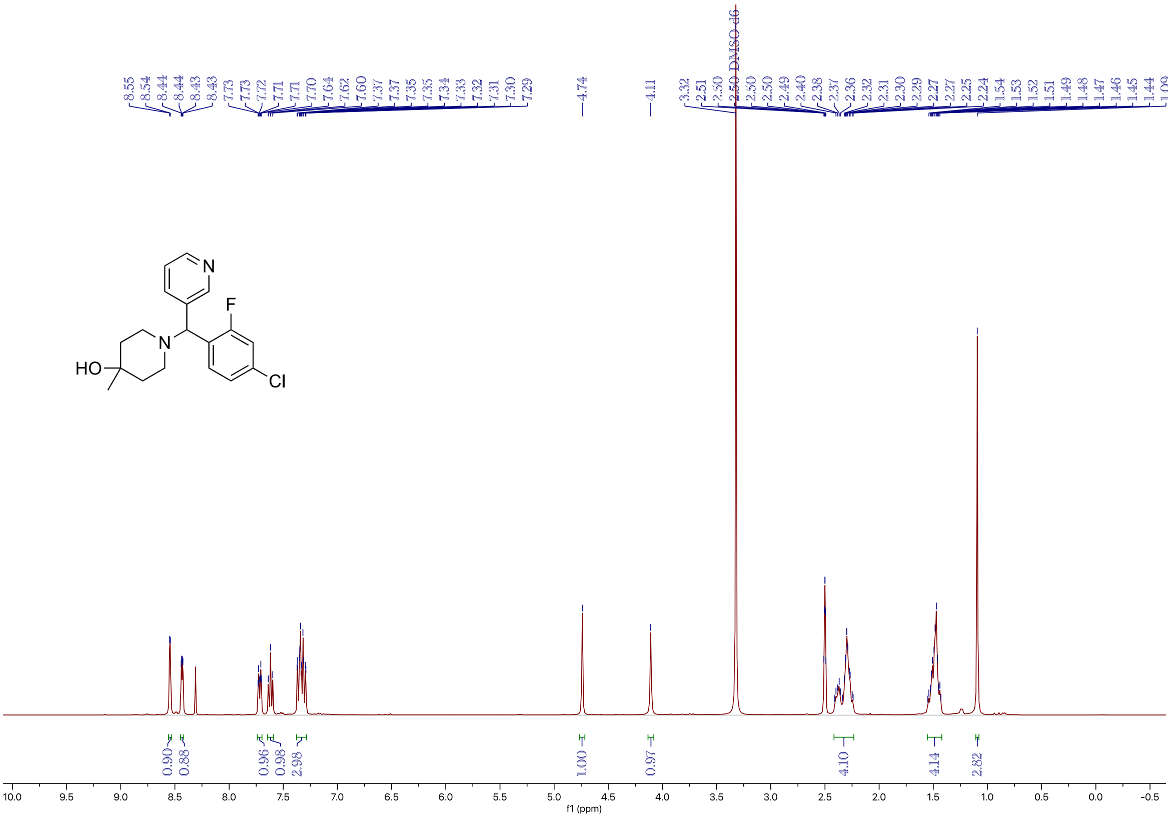


Figure S71. $^{1}$H NMR spectrum of 1–((4–chloro–2–fluorophenyl)(pyridin–3–yl)methyl)–4–methylpiperidin–4–ol (**MYOS_00151**, 400 MHz, DMSO-$d_6$).

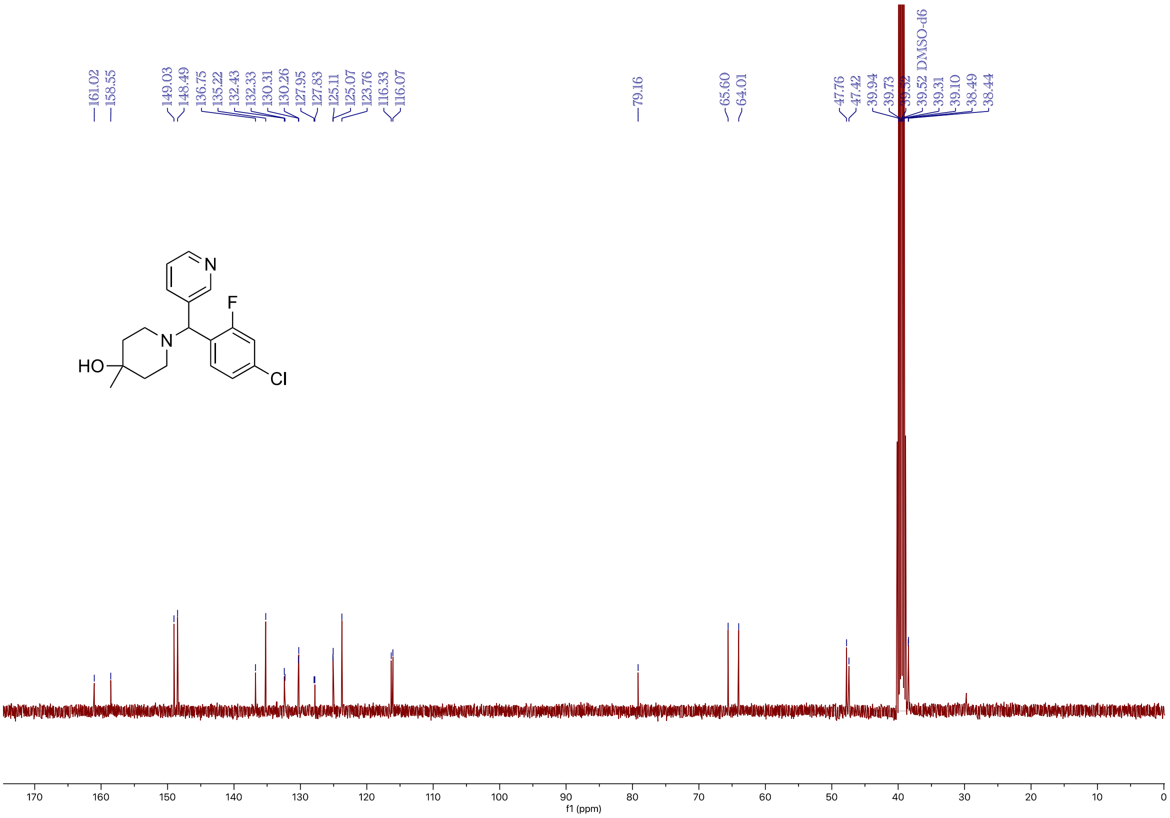


Figure S72. $^{13}$C NMR spectrum of 1–((4–chloro–2–fluorophenyl)(pyridin–3–yl)methyl)–4–methylpiperidin–4–ol (**MYOS_00151**, 101 MHz, DMSO-$d_6$).

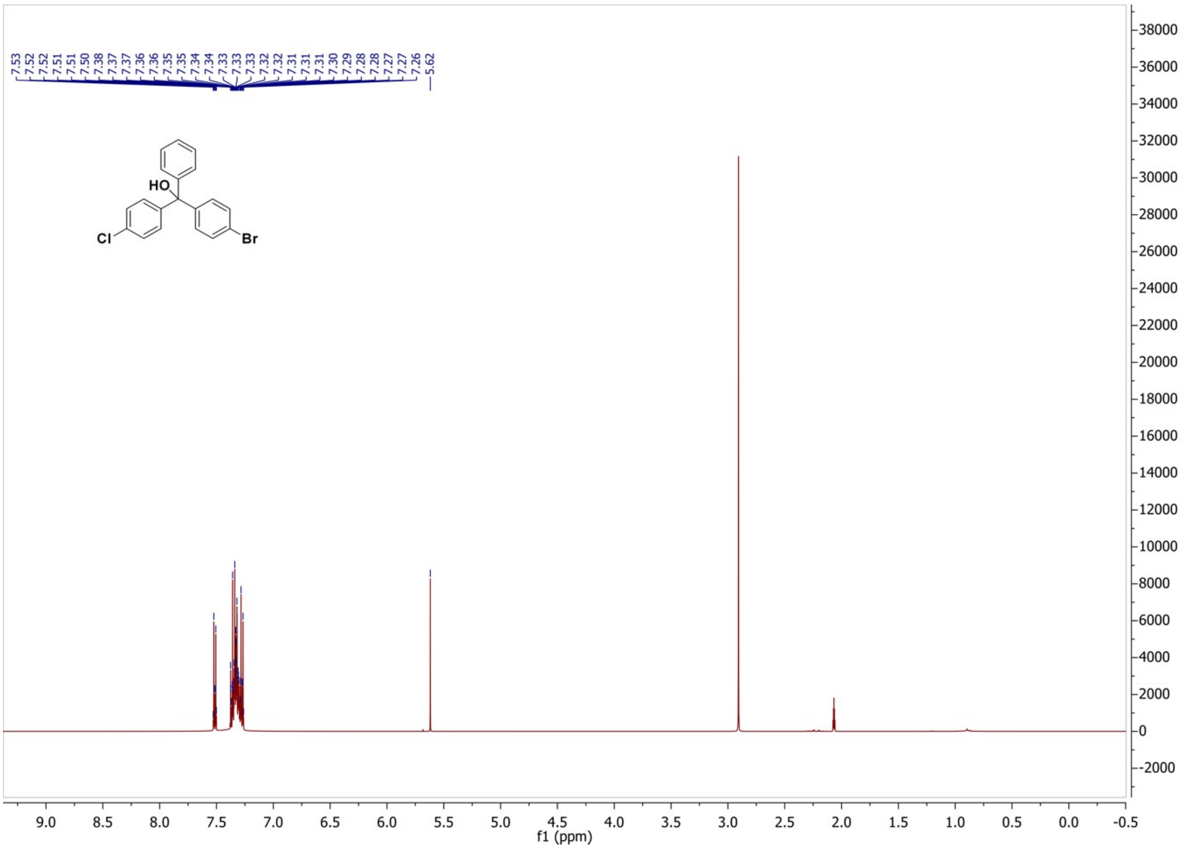


Figure S73. $^{1}$H NMR spectrum of (4-bromophenyl)(4-chlorophenyl)(phenyl)methanol (**MYOS_00163**, 500 MHz, Acetone-$d_6$).

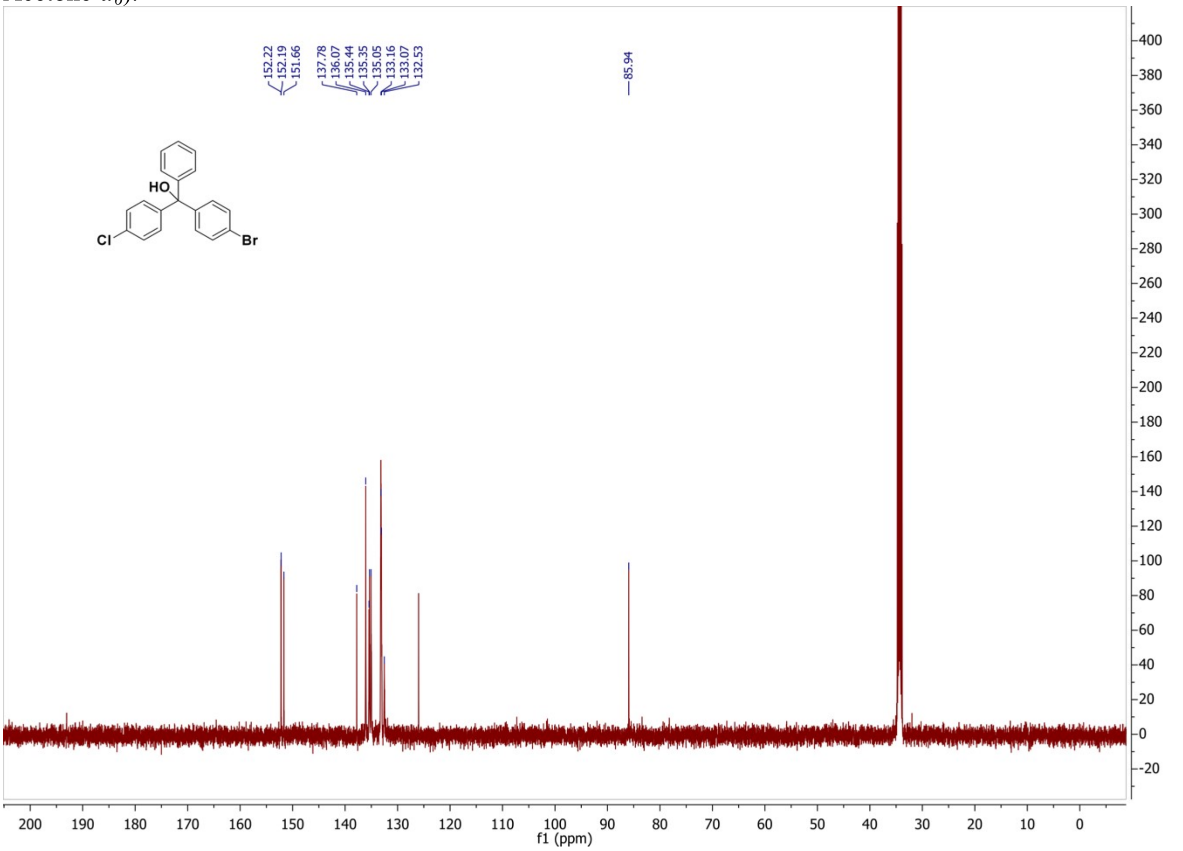


Figure S74. $^{13}$C NMR spectrum of (4-bromophenyl)(4-chlorophenyl)(phenyl)methanol (**MYOS_00163**, 126 MHz, Acetone-$d_6$).

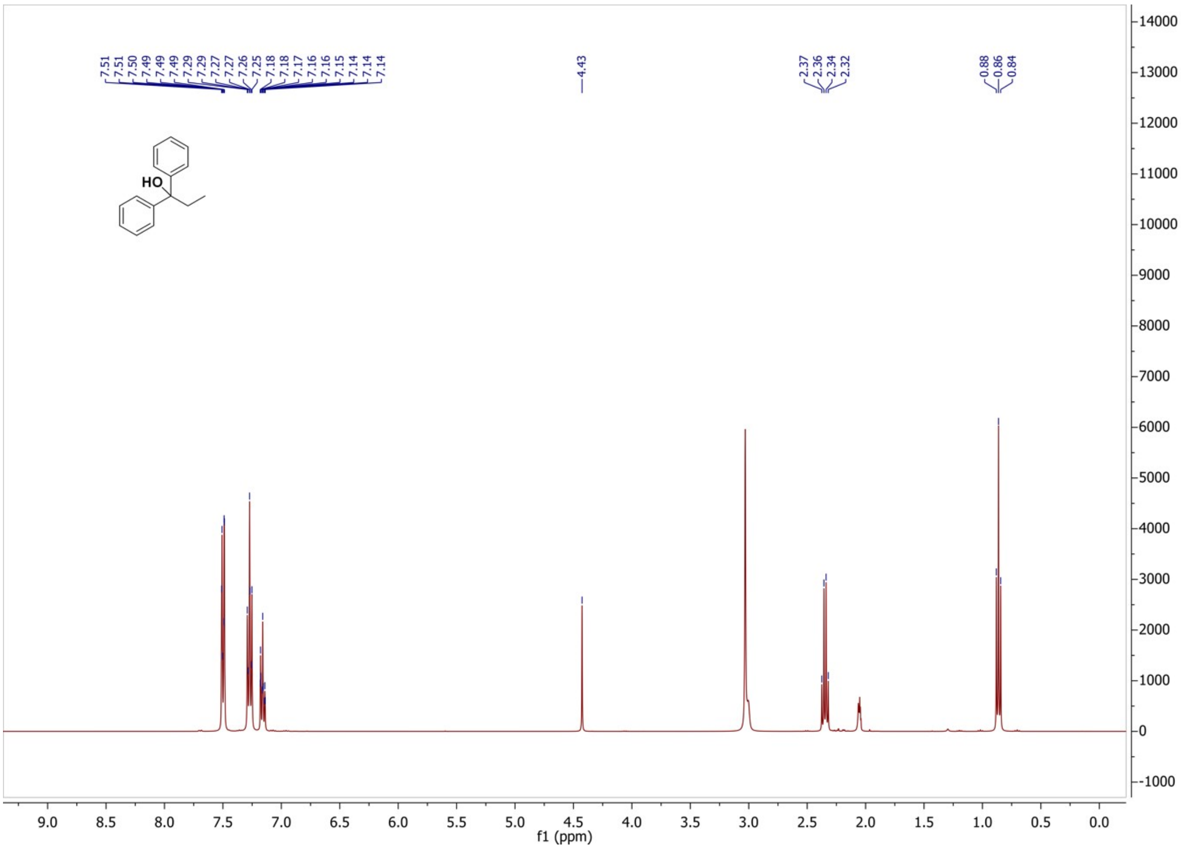

Figure S75. $^{1}$H NMR spectrum of 1,1-diphenylpropan-1-ol (**MYOS_00164**, 400 MHz, Acetone-$d_6$).

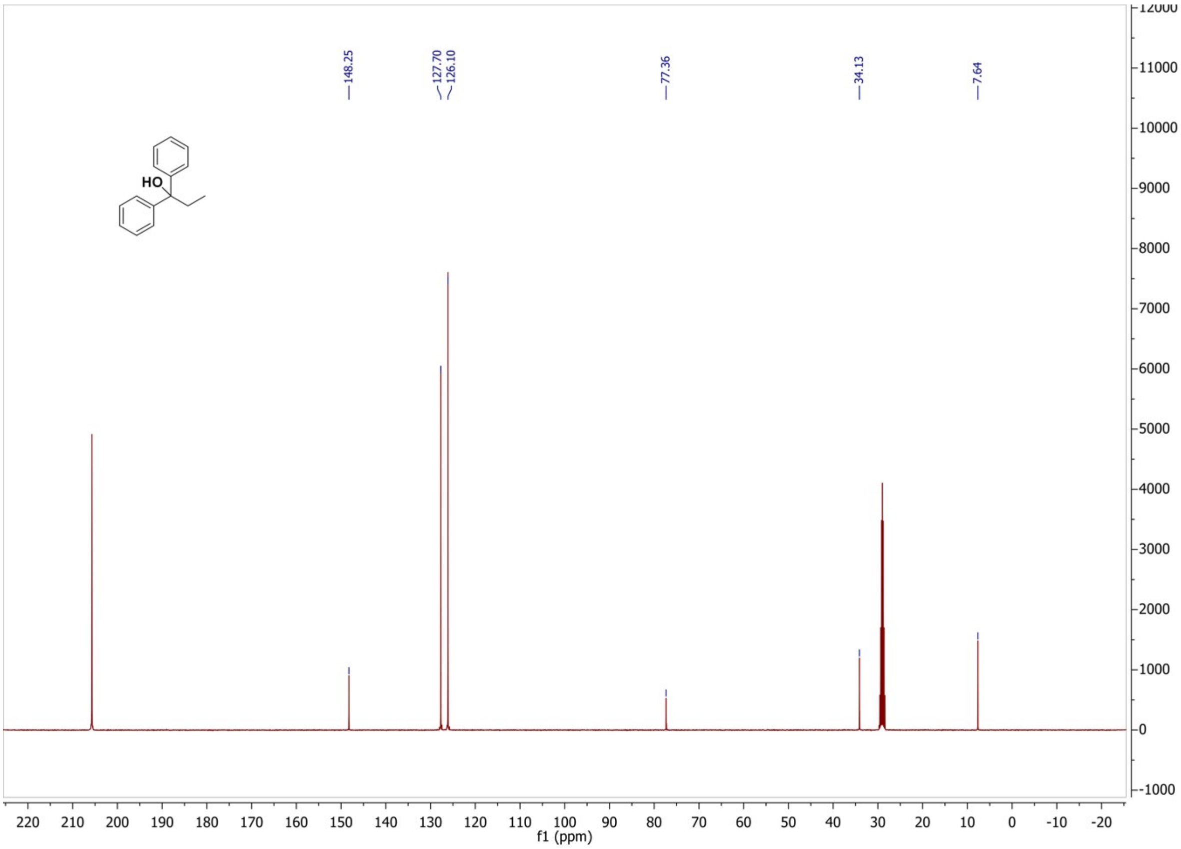

Figure S76. $^{13}$C NMR spectrum of 1,1-diphenylpropan-1-ol (**MYOS_00164**, 101 MHz, Acetone-$d_6$).

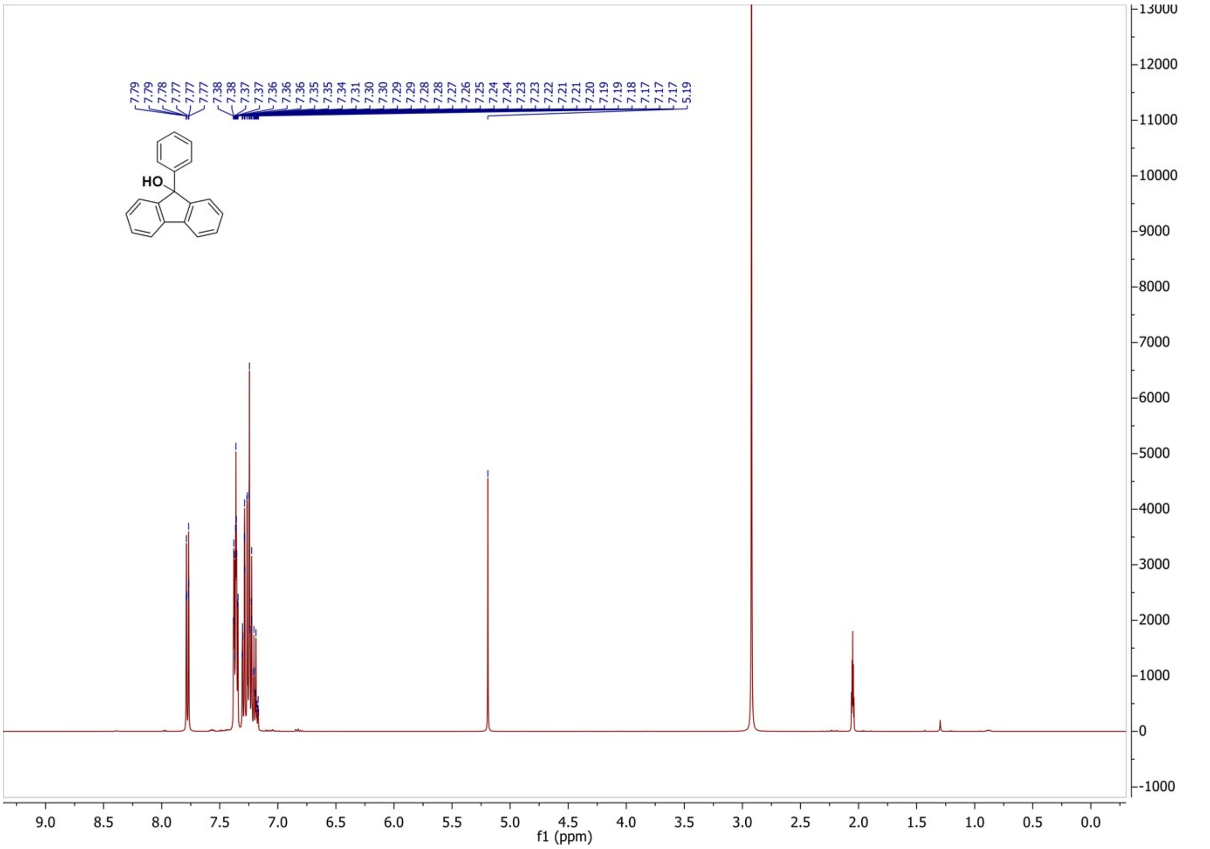


Figure S77. $^{1}$H NMR spectrum of 9-phenyl-9*H*-fluoren-9-ol (**MYOS_00165**, 400 MHz, Acetone-$d_6$).

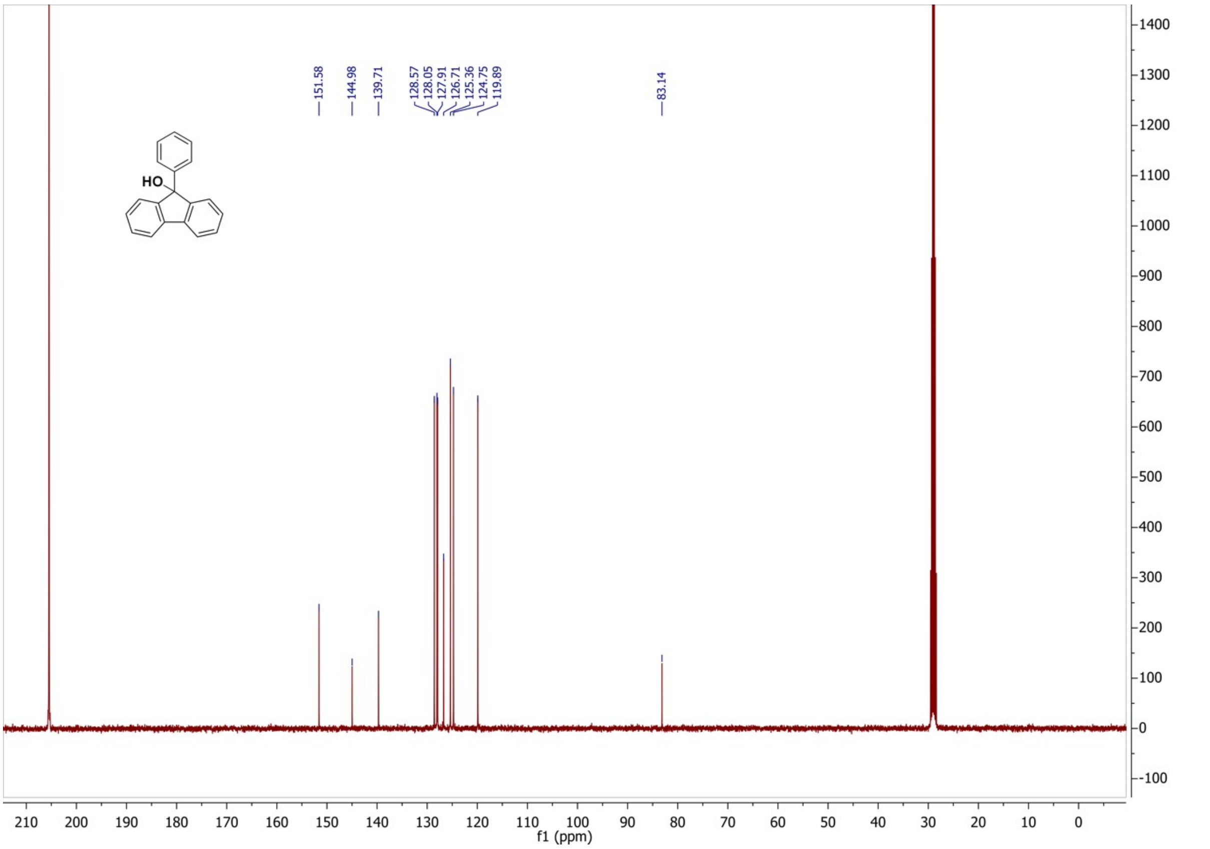


Figure S78. $^{13}$C NMR spectrum of 9-phenyl-9*H*-fluoren-9-ol (**MYOS_00165**, 101 MHz, Acetone-$d_6$).

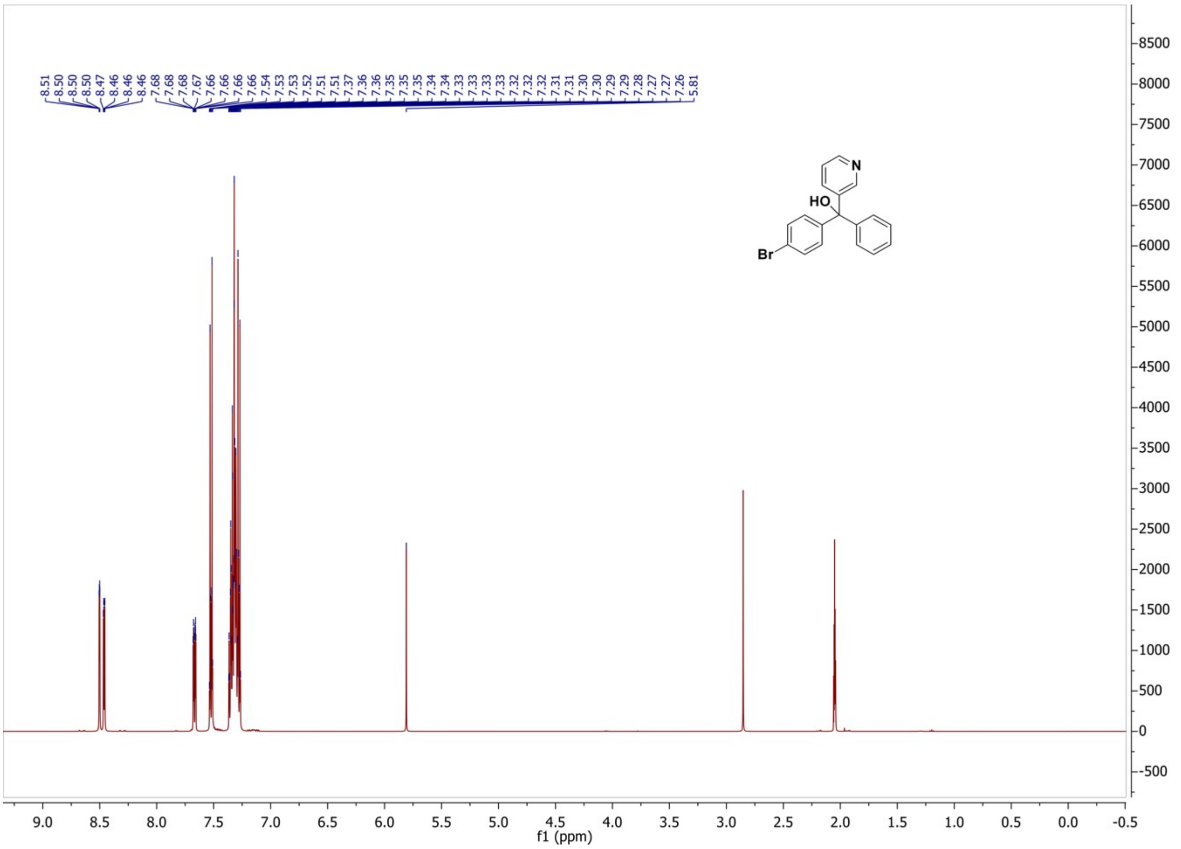

Figure S79. $^{1}H$ NMR spectrum of (4-bromophenyl)(phenyl)(pyridin-3-yl)methanol (**MYOS_00166**, 500 MHz, Acetone-$d_6$).

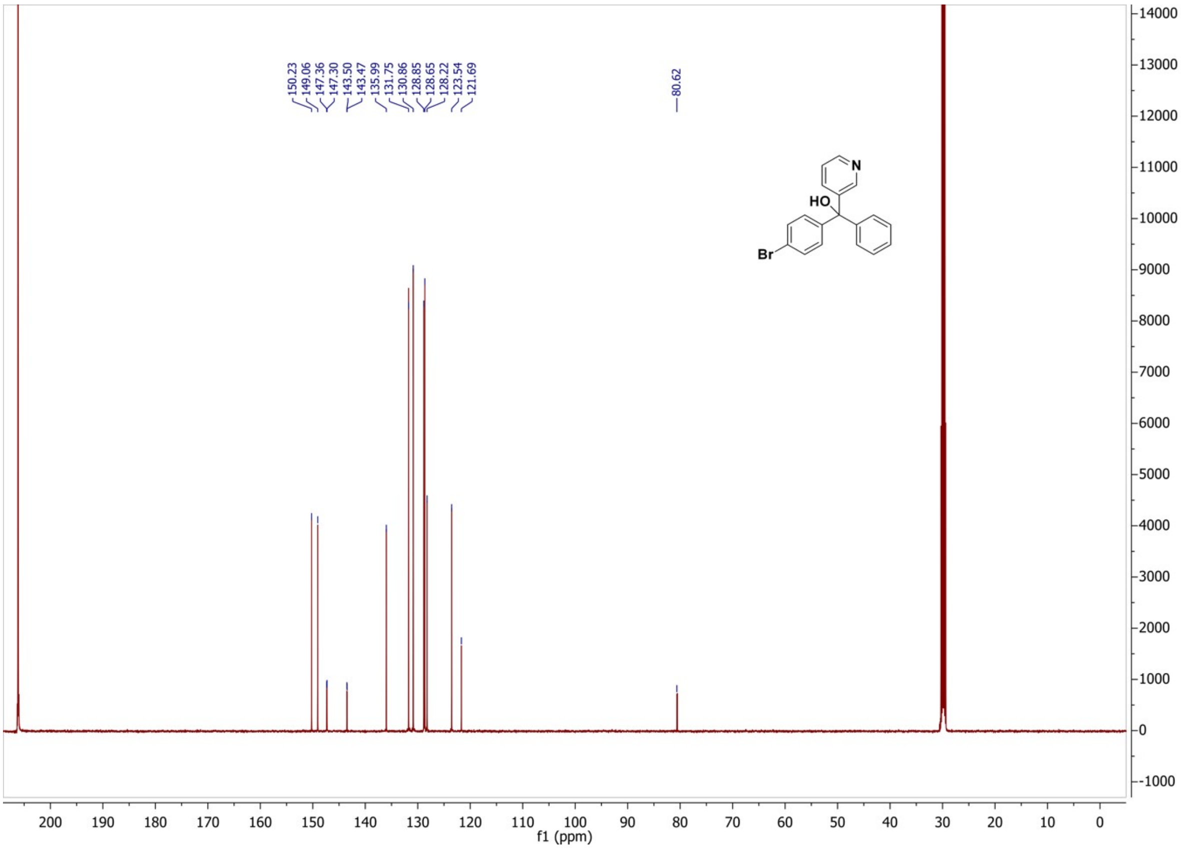

Figure S80. $^{13}C$ NMR spectrum of (4-bromophenyl)(phenyl)(pyridin-3-yl)methanol (**MYOS_00166**, 126 MHz, Acetone-$d_6$).

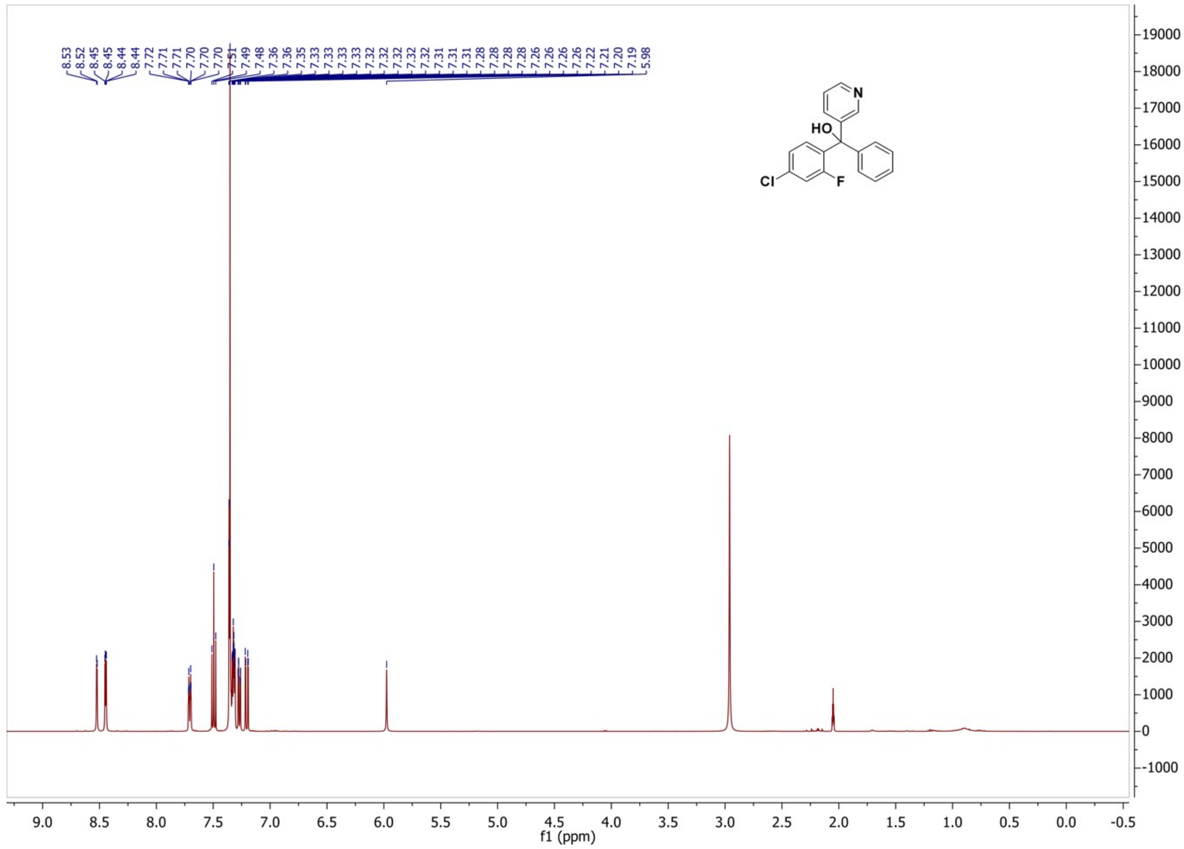

Figure S81. $^{1}$H NMR spectrum of (4-chloro-2-fluorophenyl)(phenyl)(pyridin-3-yl)methanol (**MYOS_00167**, 500 MHz, Acetone-$d_6$).

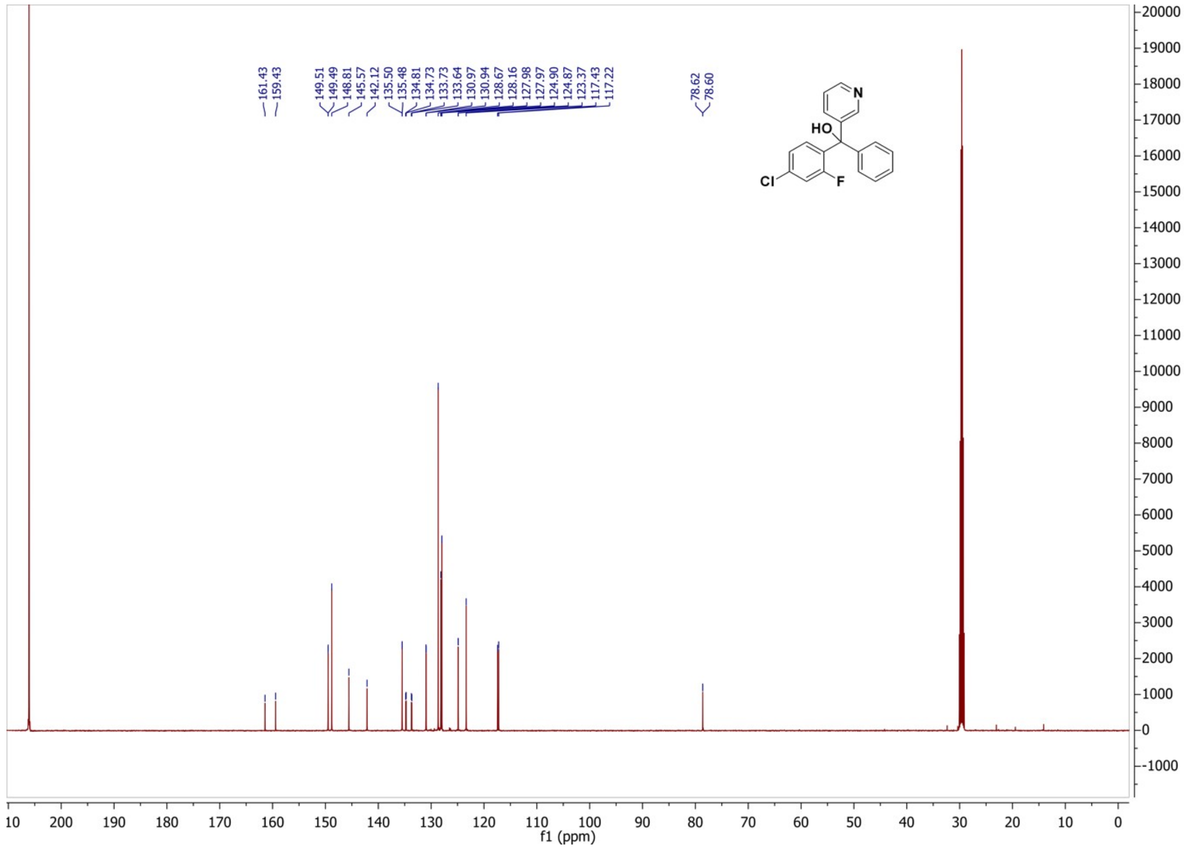

Figure S82. $^{13}$C NMR spectrum of (4-chloro-2-fluorophenyl)(phenyl)(pyridin-3-yl)methanol (**MYOS_00167**, 126 MHz, Acetone-$d_6$).

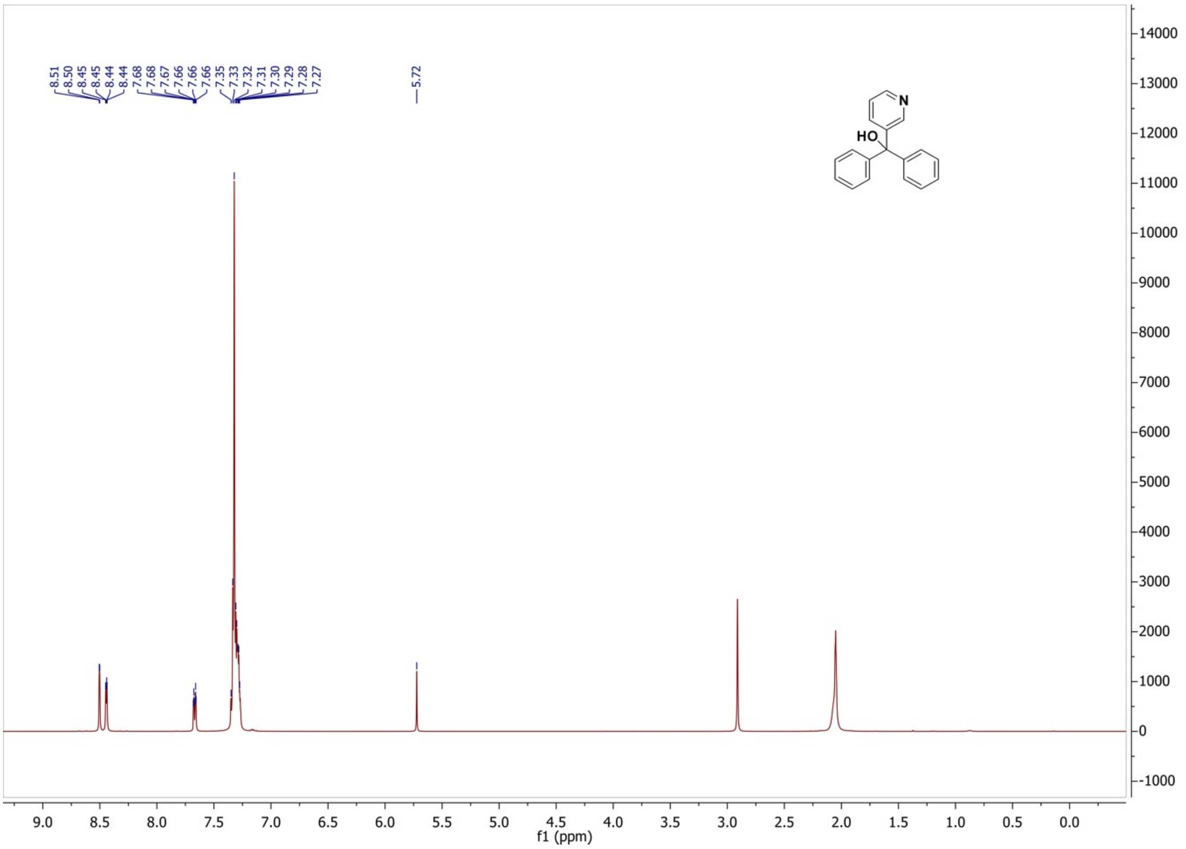


Figure S83. $^{1}$H NMR spectrum of diphenyl(pyridin-3-yl)methanol (**MYOS_00168**, 500 MHz, Acetone-$d_6$).

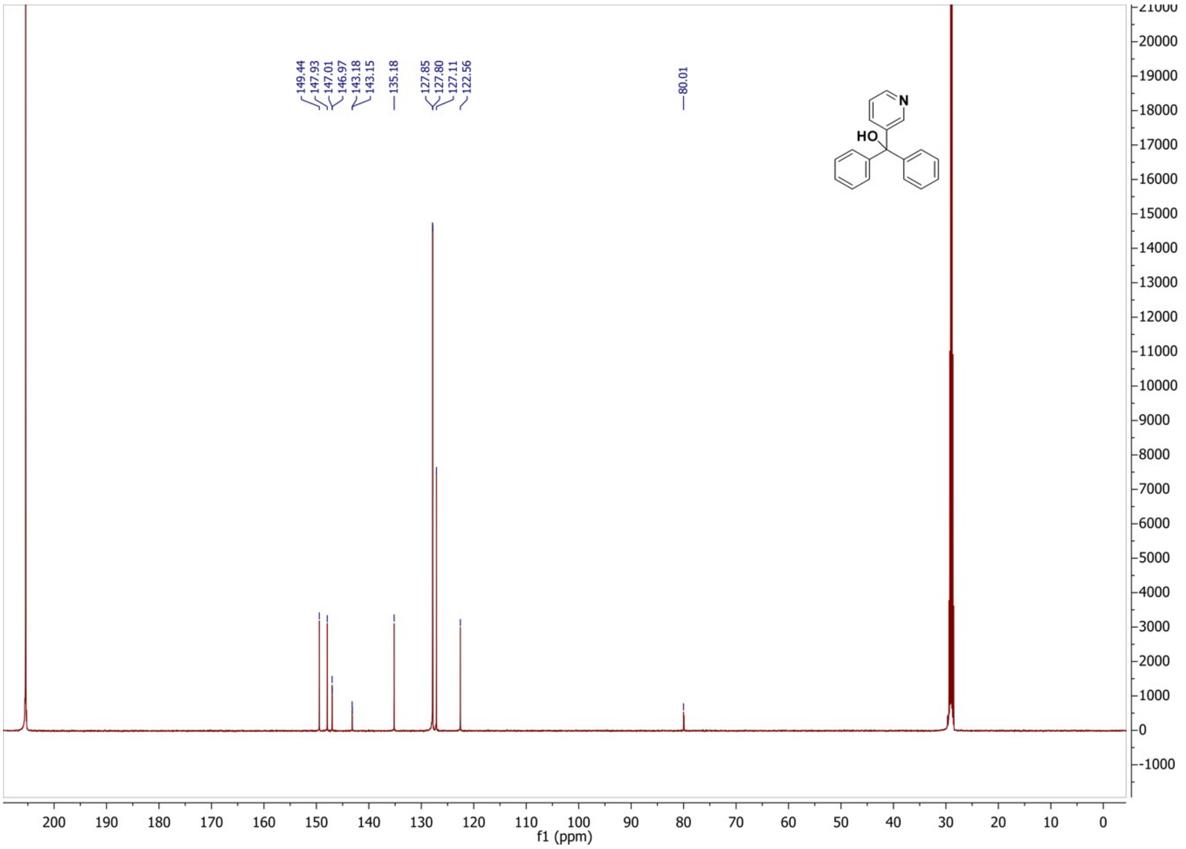


Figure S84. $^{13}$C NMR spectrum of diphenyl(pyridin-3-yl)methanol (**MYOS_00168**, 126 MHz, Acetone-$d_6$).

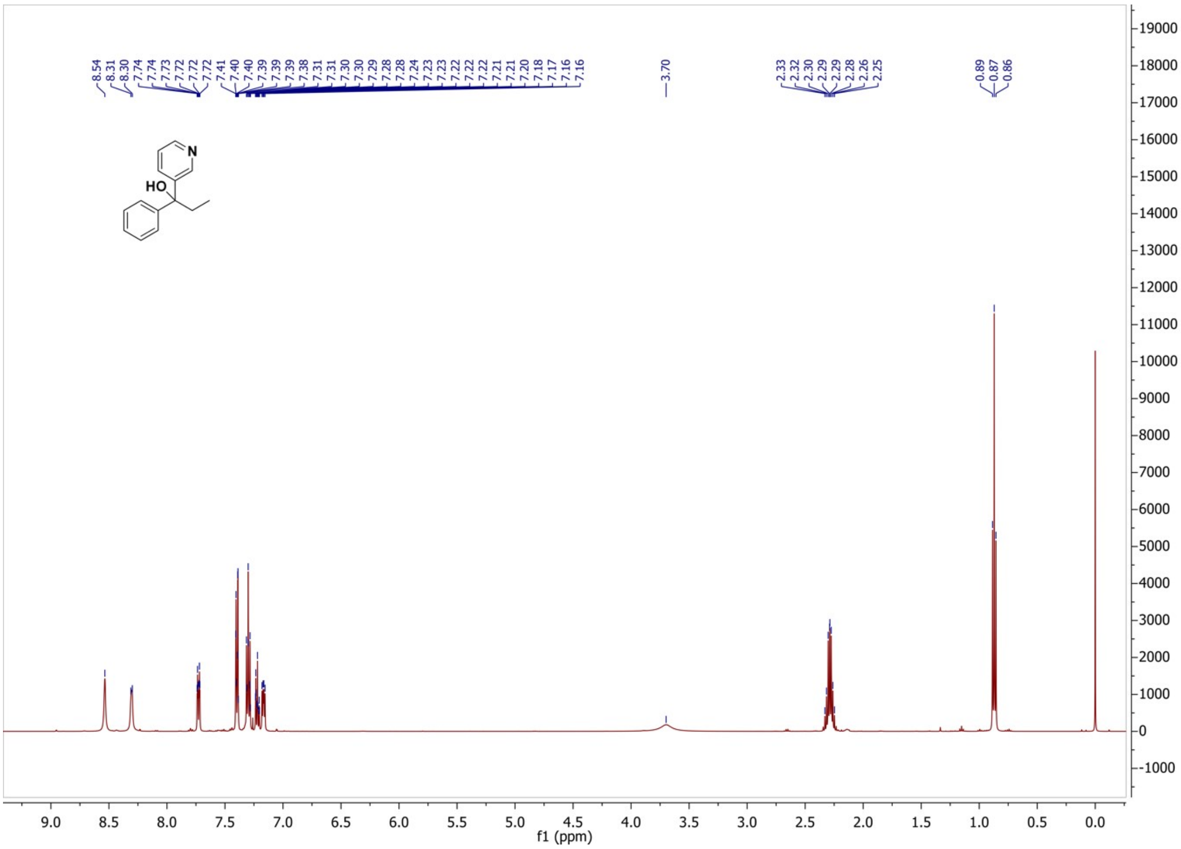

Figure S85. $^{1}$H NMR spectrum of 1-phenyl-1-(pyridin-3-yl)propan-1-ol (**MYOS_00169**, 500 MHz, $CDCl_3$).

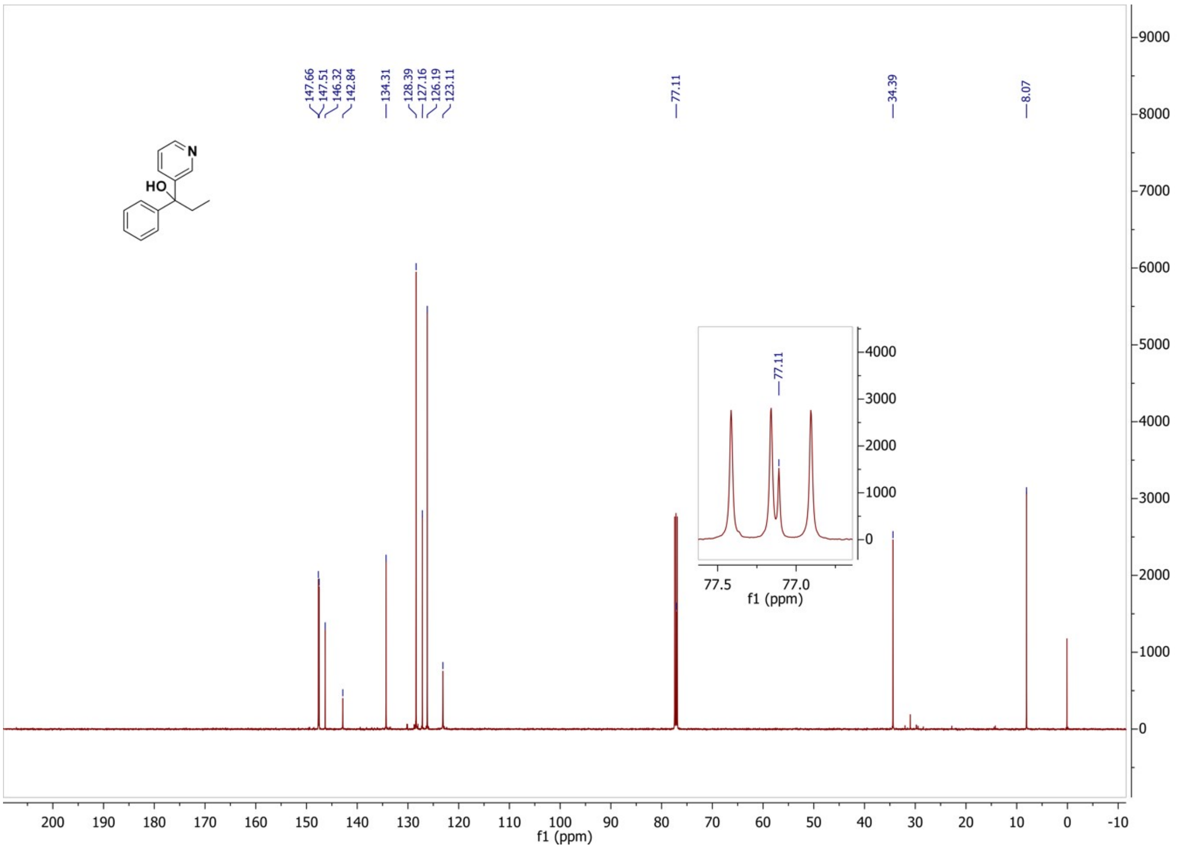

Figure S86. $^{13}$C NMR spectrum of 1-phenyl-1-(pyridin-3-yl)propan-1-ol (**MYOS_00169**, 126 MHz, $CDCl_3$).

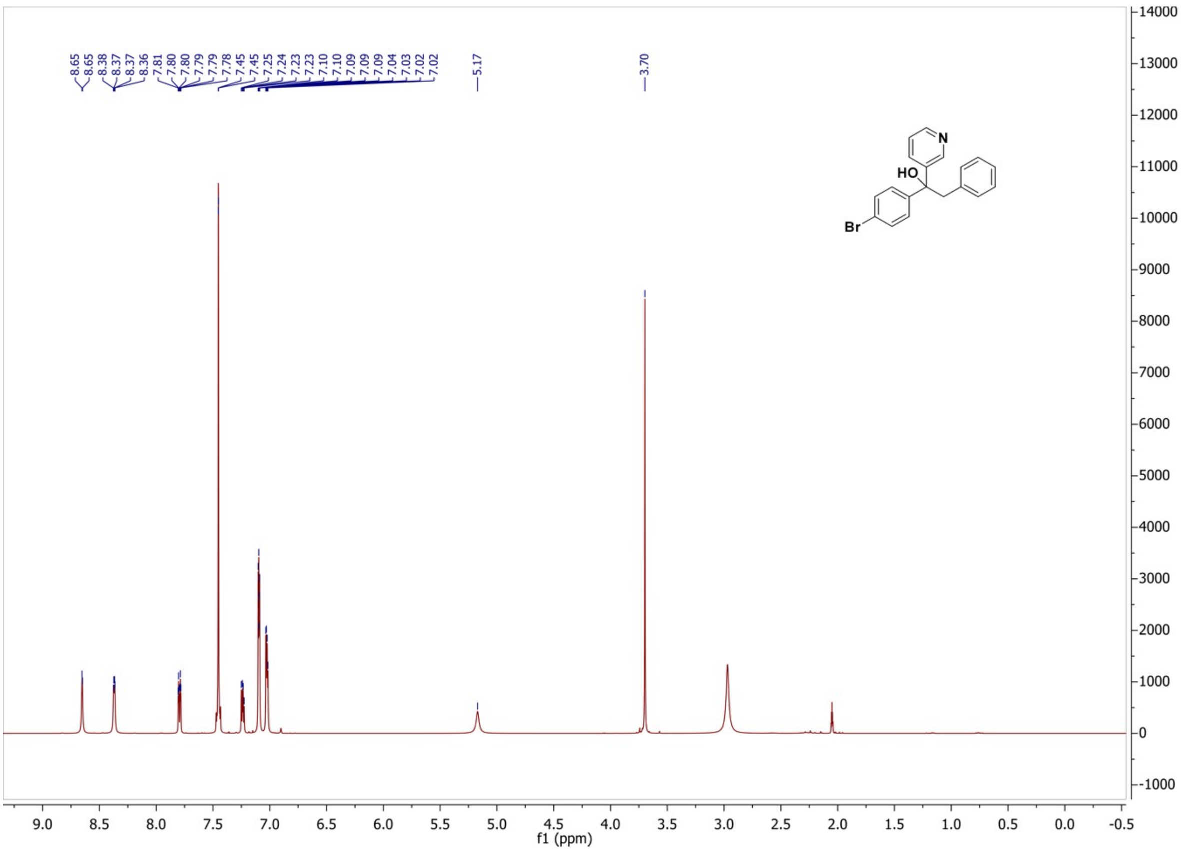


Figure S87. $^{1}H$ NMR spectrum of 1-(4-bromophenyl)-2-phenyl-1-(pyridin-3-yl)ethan-1-ol (**MYOS_00170**, 500 MHz, Acetone-$d_6$).

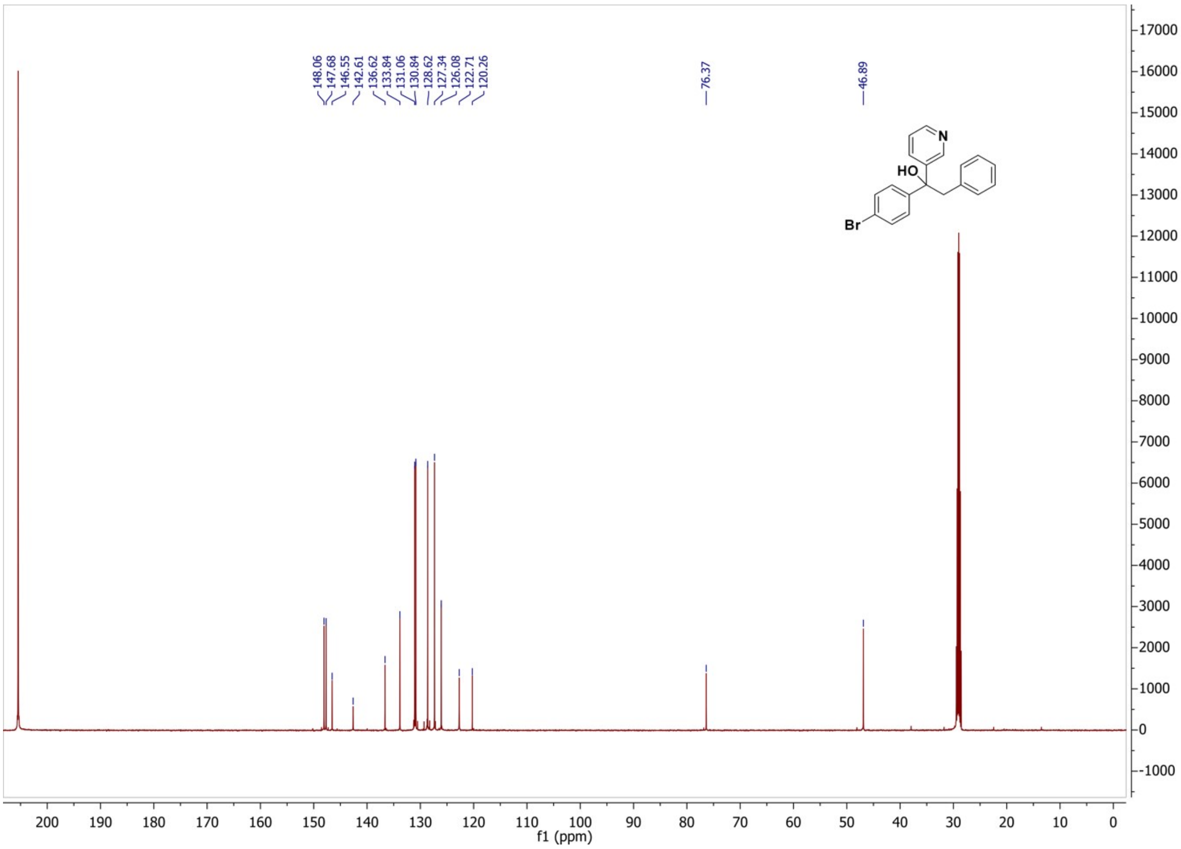


Figure S88. $^{13}C$ NMR spectrum of 1-(4-bromophenyl)-2-phenyl-1-(pyridin-3-yl)ethan-1-ol (**MYOS_00170**, 126 MHz, Acetone-$d_6$).

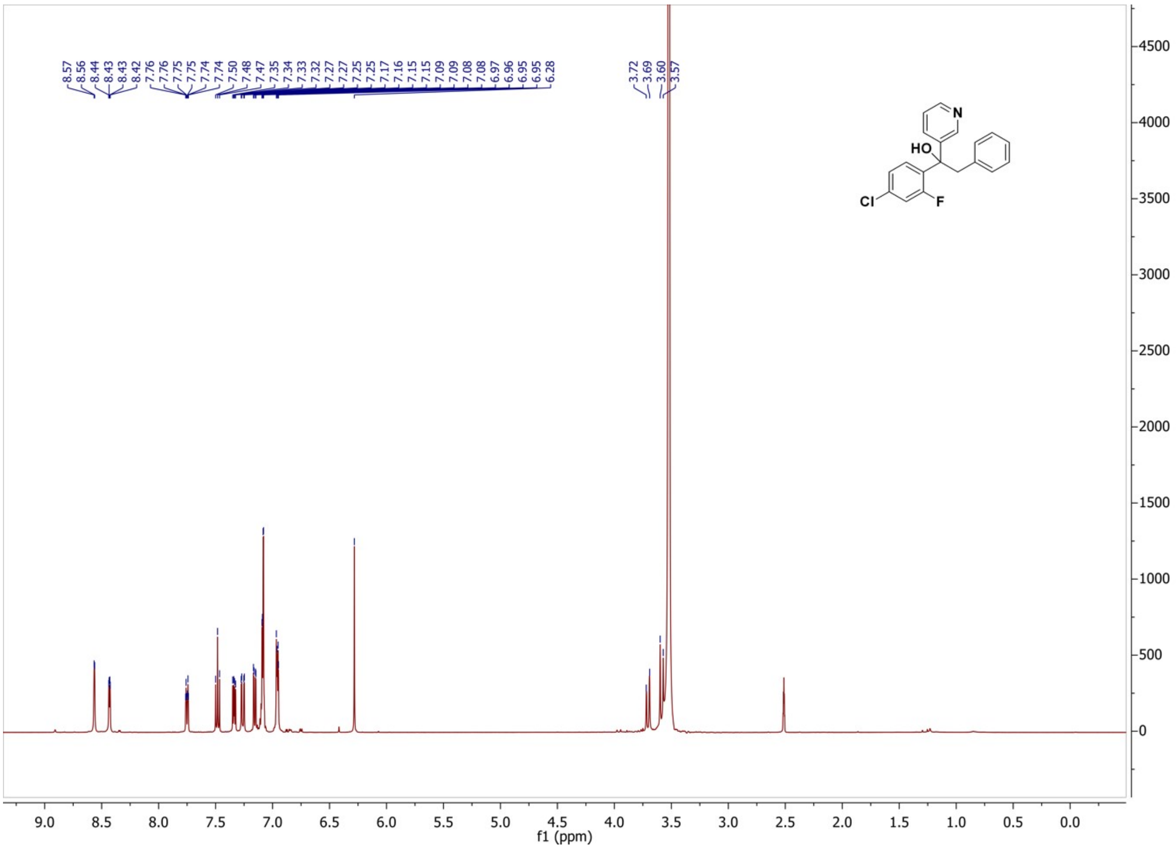


Figure S89. $^{1}$H NMR spectrum of 1-(4-chloro-2-fluorophenyl)-2-phenyl-1-(pyridin-3-yl)ethan-1-ol (**MYOS_00171**, 500 MHz, DMSO-$d_6$).

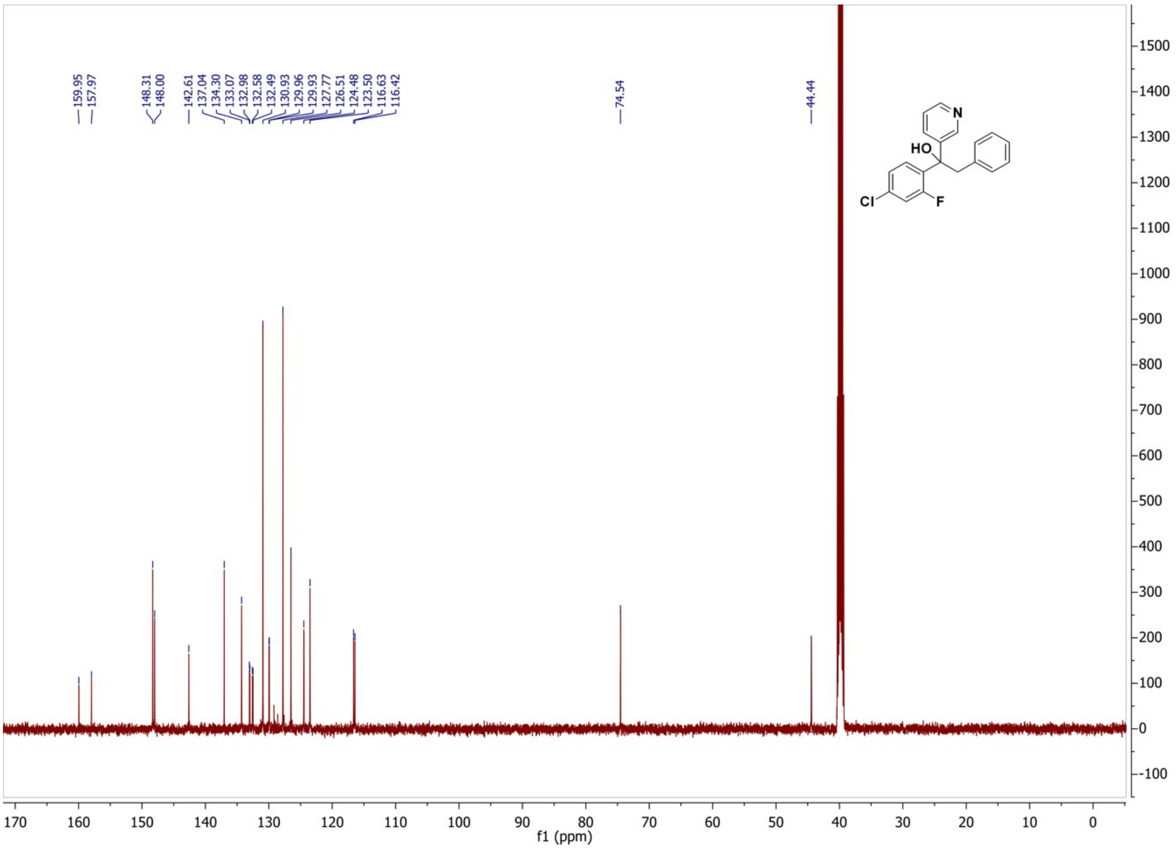


Figure S90. $^{13}$C NMR spectrum of 1-(4-chloro-2-fluorophenyl)-2-phenyl-1-(pyridin-3-yl)ethan-1-ol (**MYOS_00171**, 126 MHz, DMSO-$d_6$).

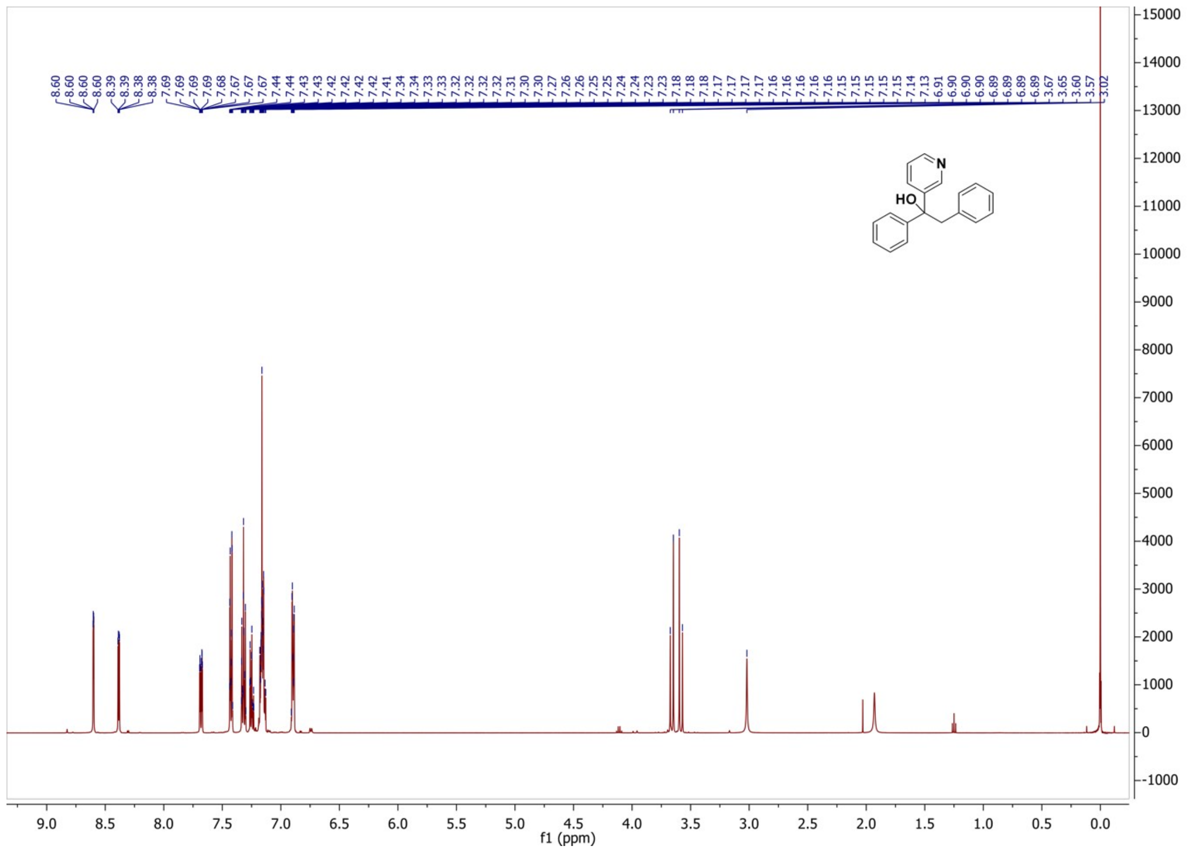

Figure S91. $^{1}$H NMR spectrum of 1,2-diphenyl-1-(pyridin-3-yl)ethan-1-ol (**MYOS_00172**, 500 MHz, $CDCl_3$).

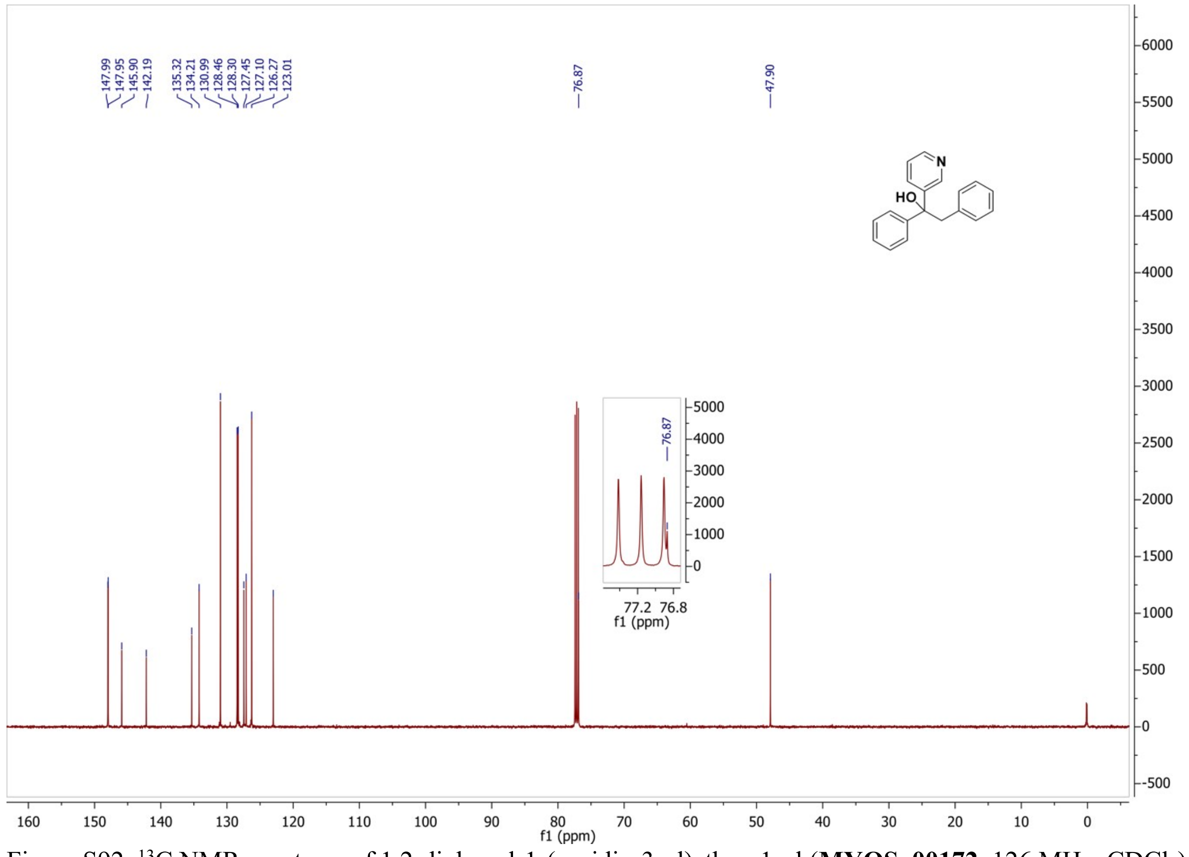

Figure S92. $^{13}$C NMR spectrum of 1,2-diphenyl-1-(pyridin-3-yl)ethan-1-ol (**MYOS_00172**, 126 MHz, $CDCl_3$).

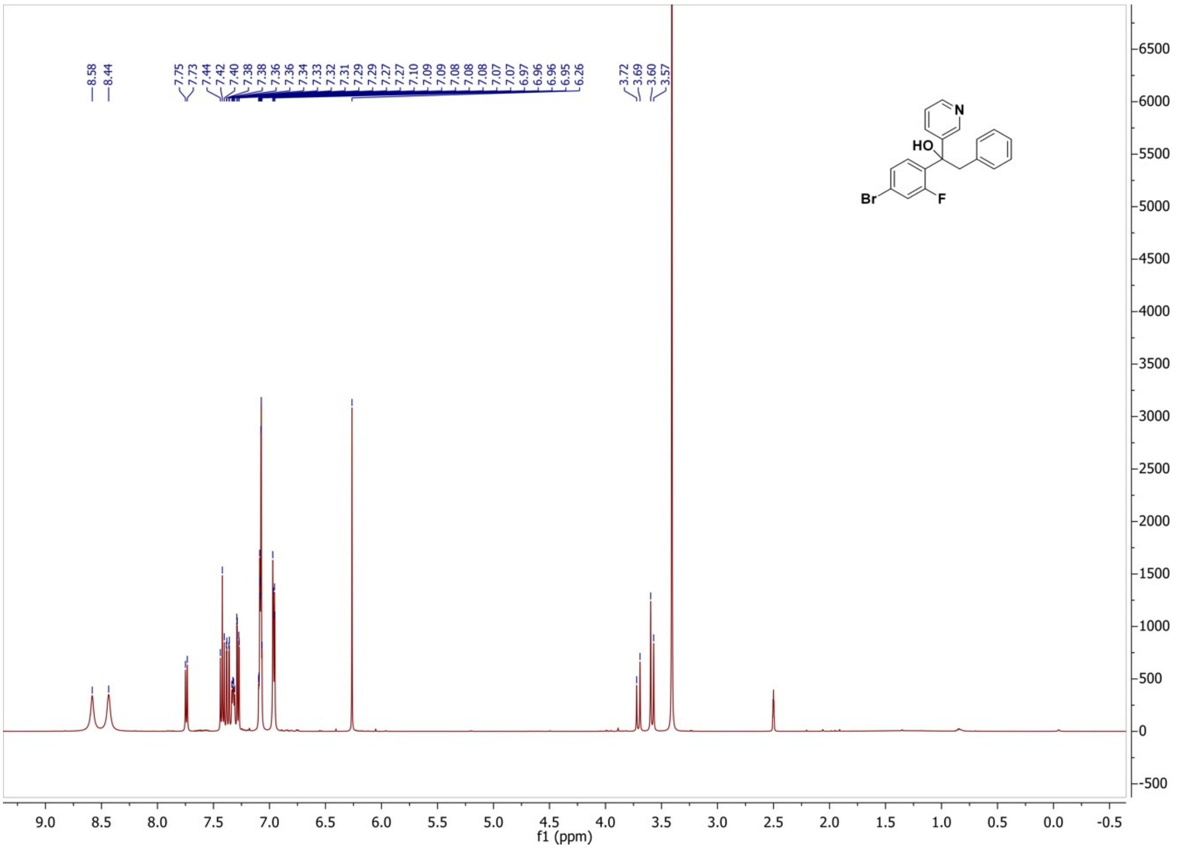


Figure S93. $^{1}H$ NMR spectrum of 1-(4-bromo-2-fluorophenyl)-2-phenyl-1-(pyridin-3-yl)ethan-1-ol (**MYOS_00173**, 500 MHz, DMSO-$d_6$).

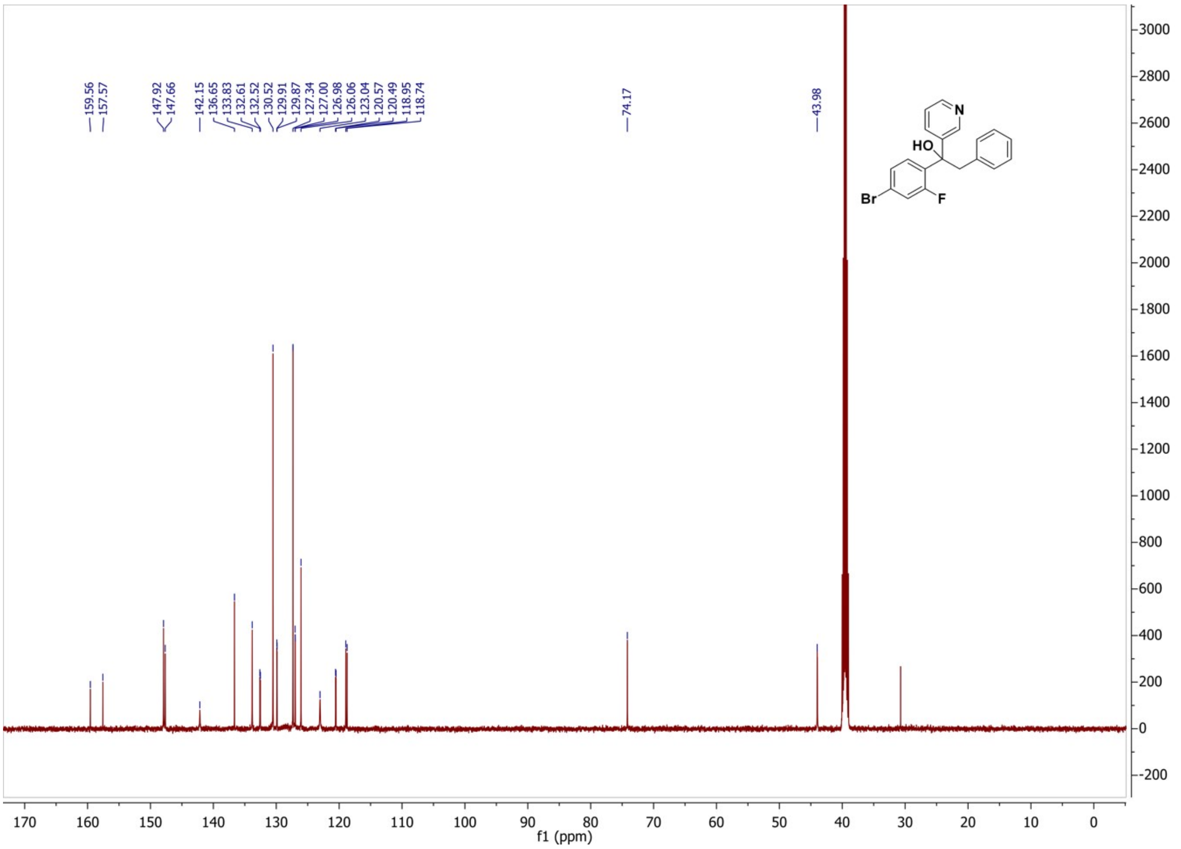


Figure S94. $^{13}C$ NMR spectrum of 1-(4-bromo-2-fluorophenyl)-2-phenyl-1-(pyridin-3-yl)ethan-1-ol (**MYOS_00173**, 126 MHz, DMSO-$d_6$).

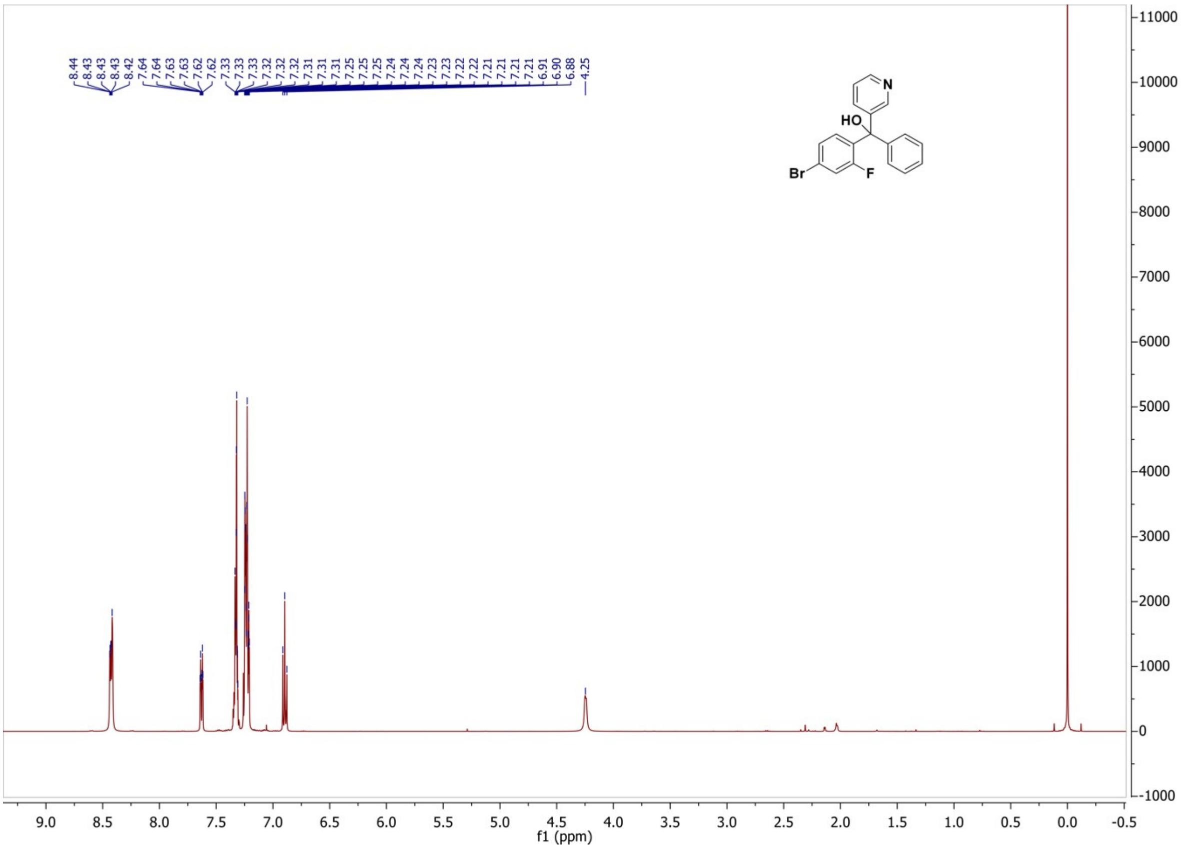

Figure S95. $^{1}H$ NMR spectrum of (4-bromo-2-fluorophenyl)(phenyl)(pyridin-3-yl)methanol (**MYOS_00174**, 500 MHz, $CDCl_3$).

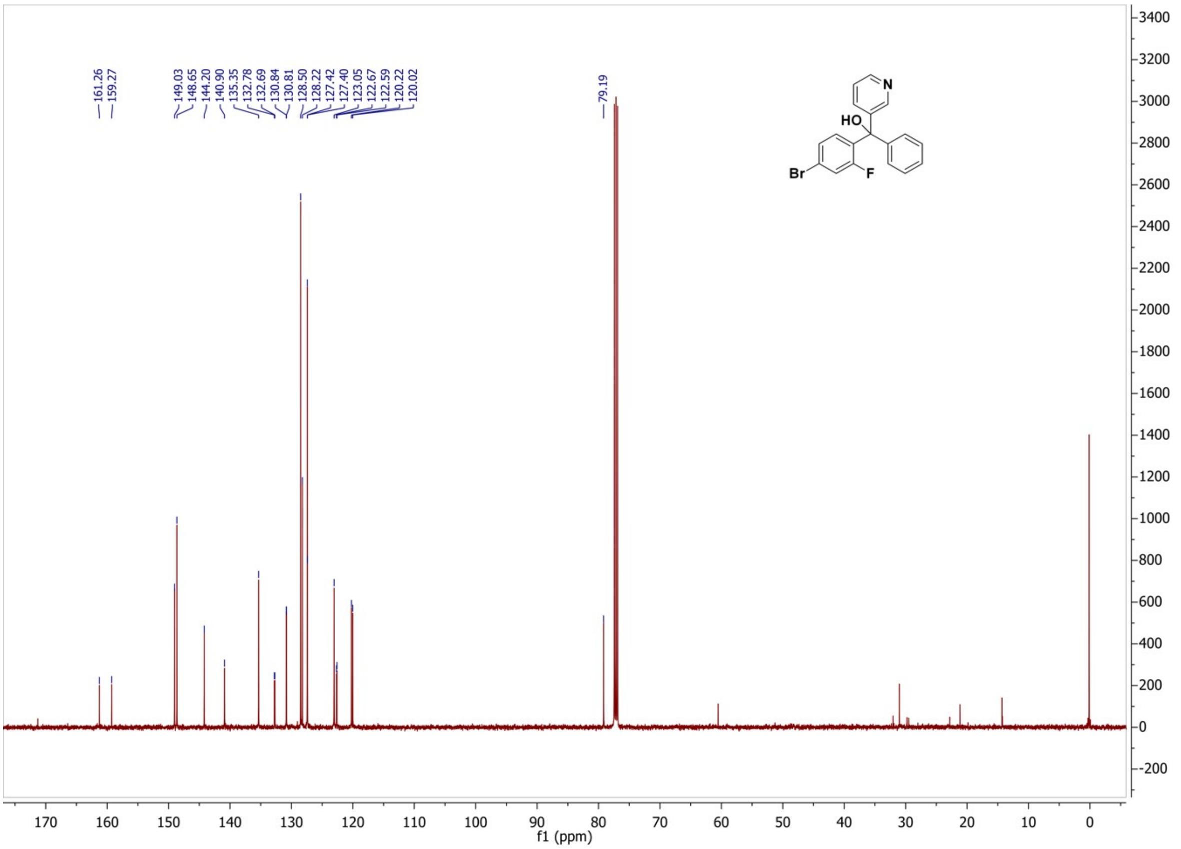

Figure S96. $^{13}C$ NMR spectrum of (4-bromo-2-fluorophenyl)(phenyl)(pyridin-3-yl)methanol (**MYOS_00174**, 126 MHz, $CDCl_3$).

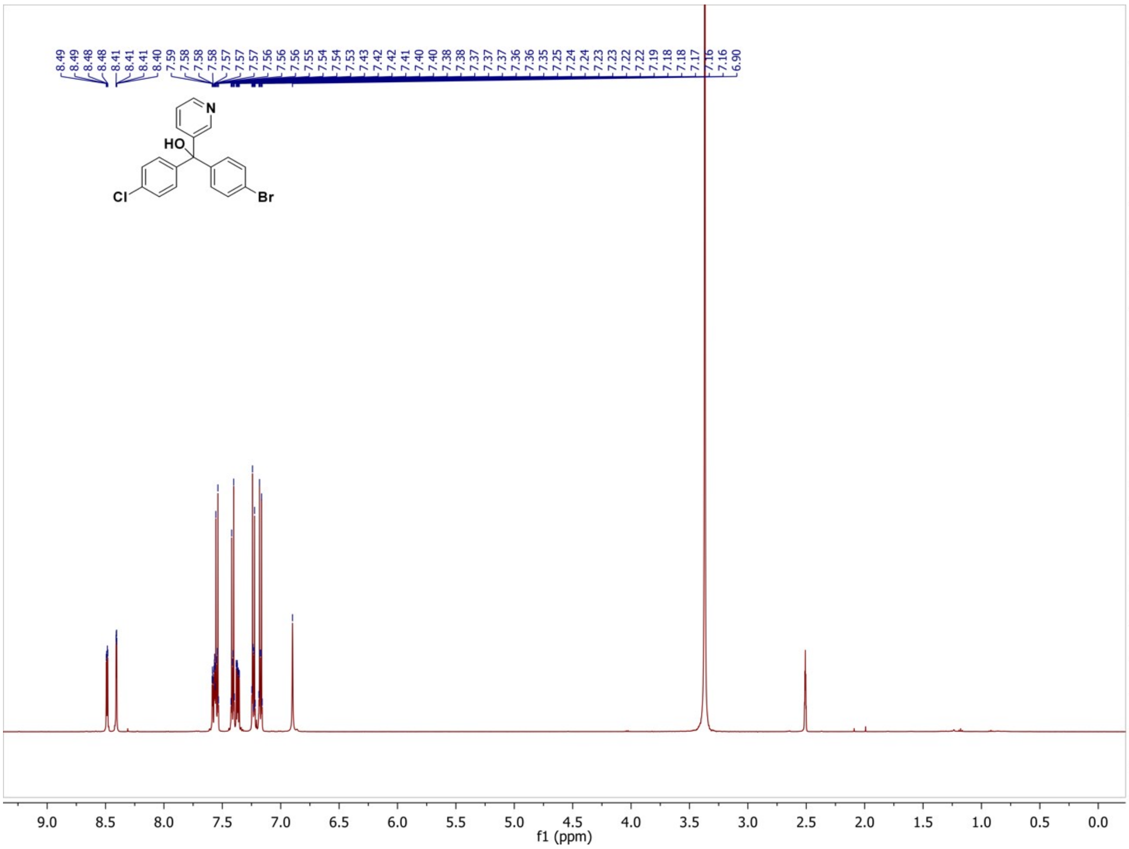

Figure S97. $^{1}$H NMR spectrum of (4-bromophenyl)(4-chlorophenyl)(pyridin-3-yl)methanol (**MYOS_00175**, 500 MHz, DMSO-$d_6$).

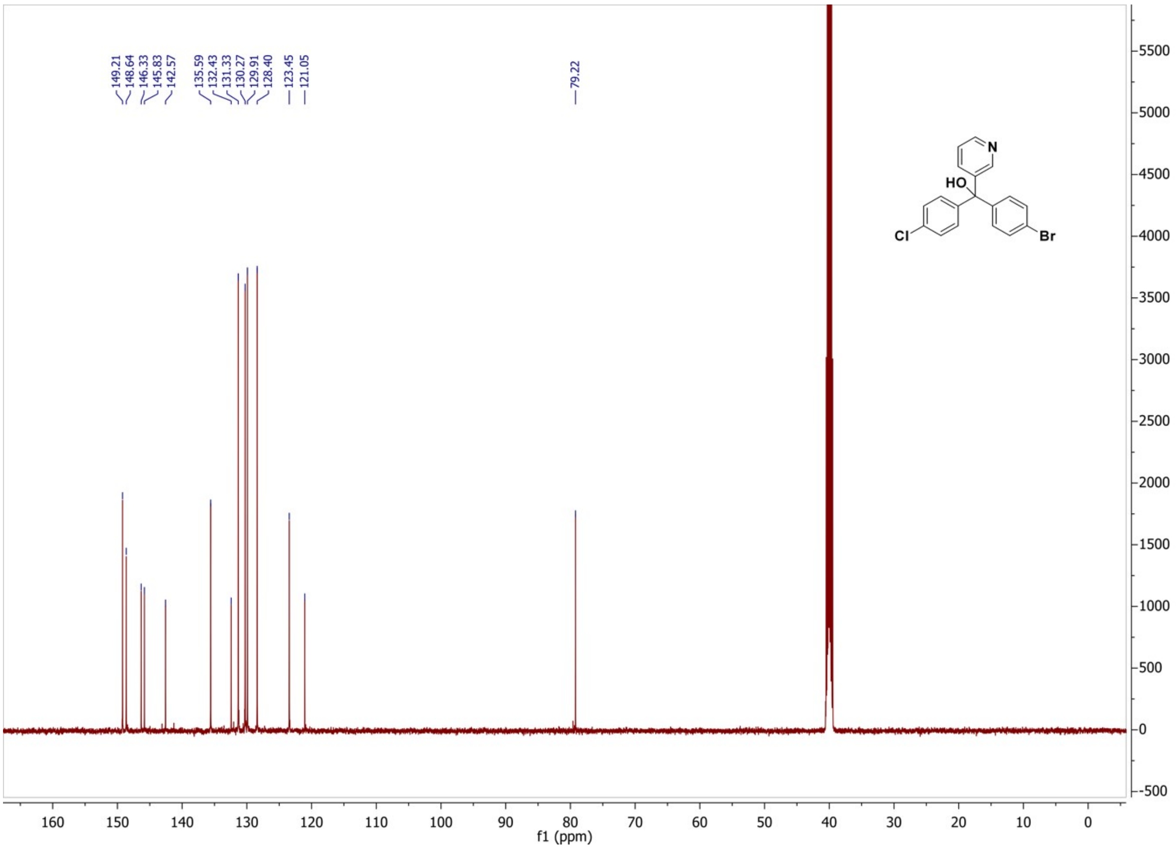

Figure S98. $^{13}$C NMR spectrum of (4-bromophenyl)(4-chlorophenyl)(pyridin-3-yl)methanol (**MYOS_00175**, 126 MHz, DMSO-$d_6$).

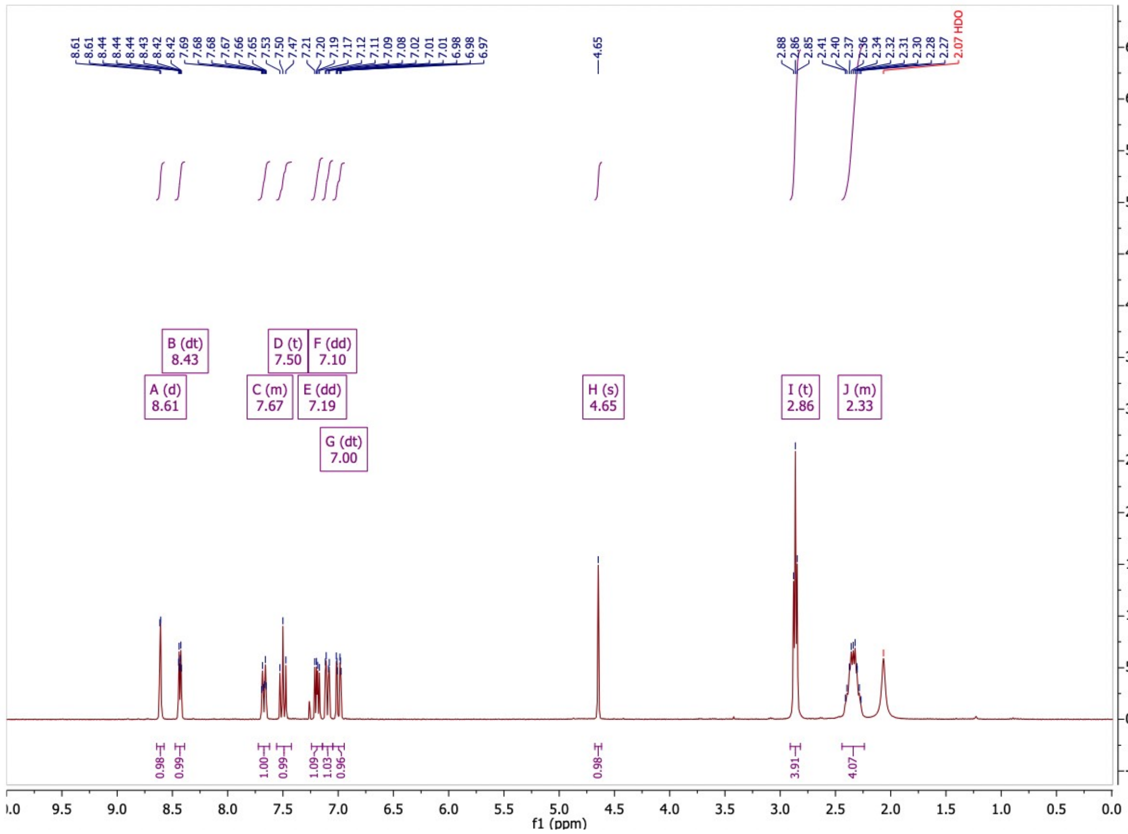


Figure S99. $^1$H NMR spectrum of 1-((4-chloro-2-fluorophenyl)(pyridin-3-yl)methyl)piperazine (**MYOS_00189**, 300 MHz, $CDCl_3$).

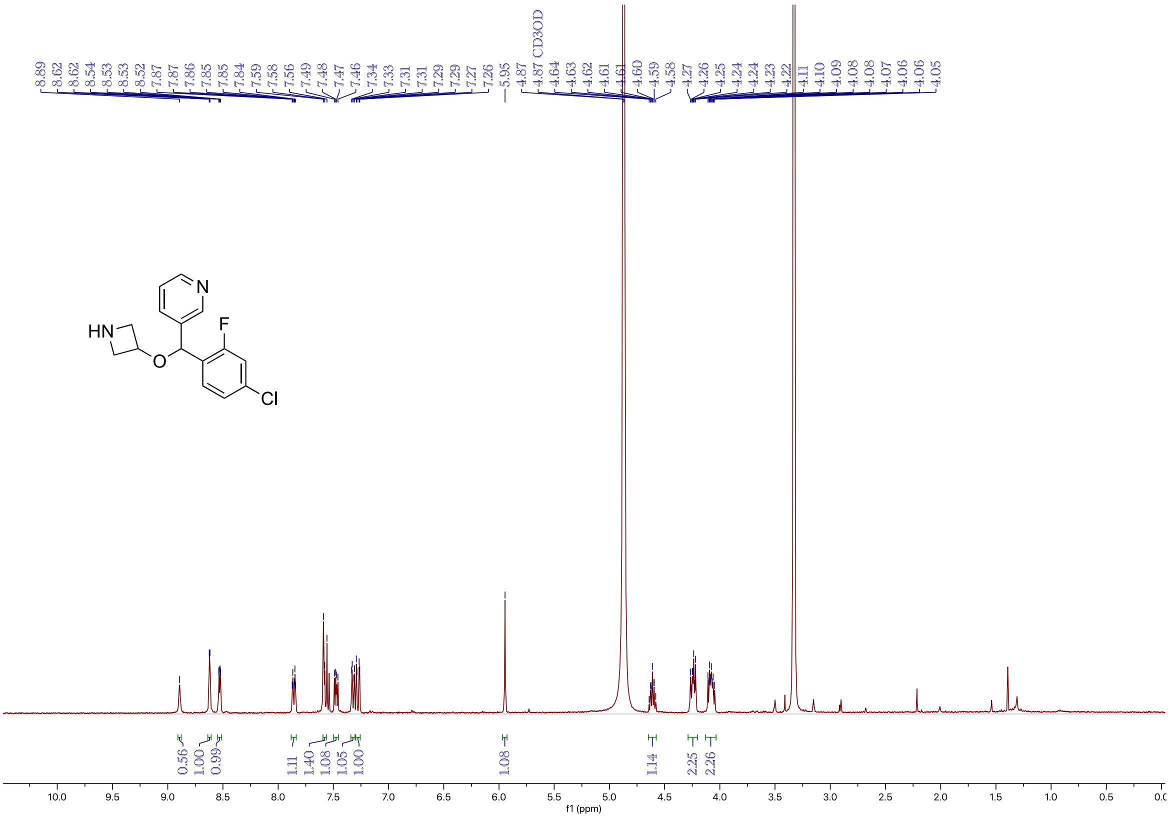


Figure S100. $^{1}H$ NMR spectrum of 3–((azetidin–3–yloxy)(4–chloro–2–fluorophenyl)methyl)pyridine (**MYOS_00190**, 400 MHz, Methanol-$d_4$).

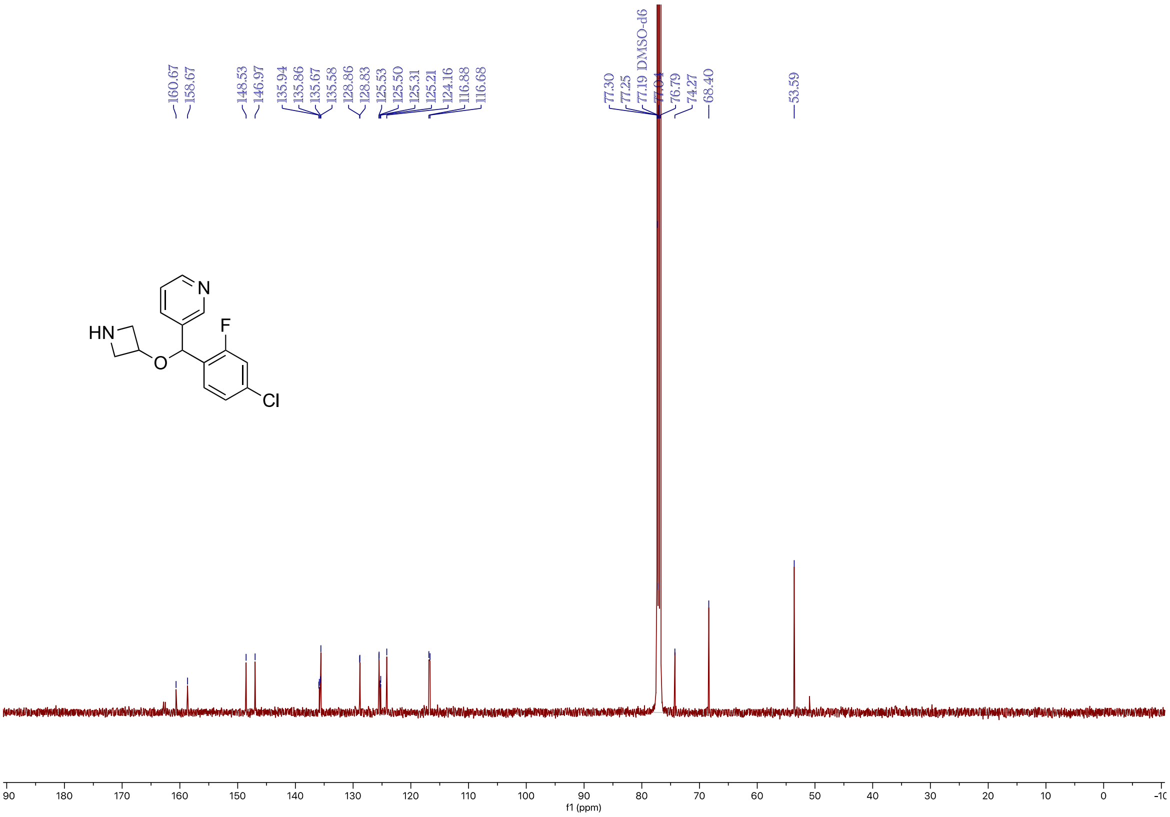


Figure S101. $^{13}C$ NMR spectrum of 3–((azetidin–3–yloxy)(4–chloro–2–fluorophenyl)methyl)pyridine (**MYOS_00190**, 126 MHz, $CDCl_3$).

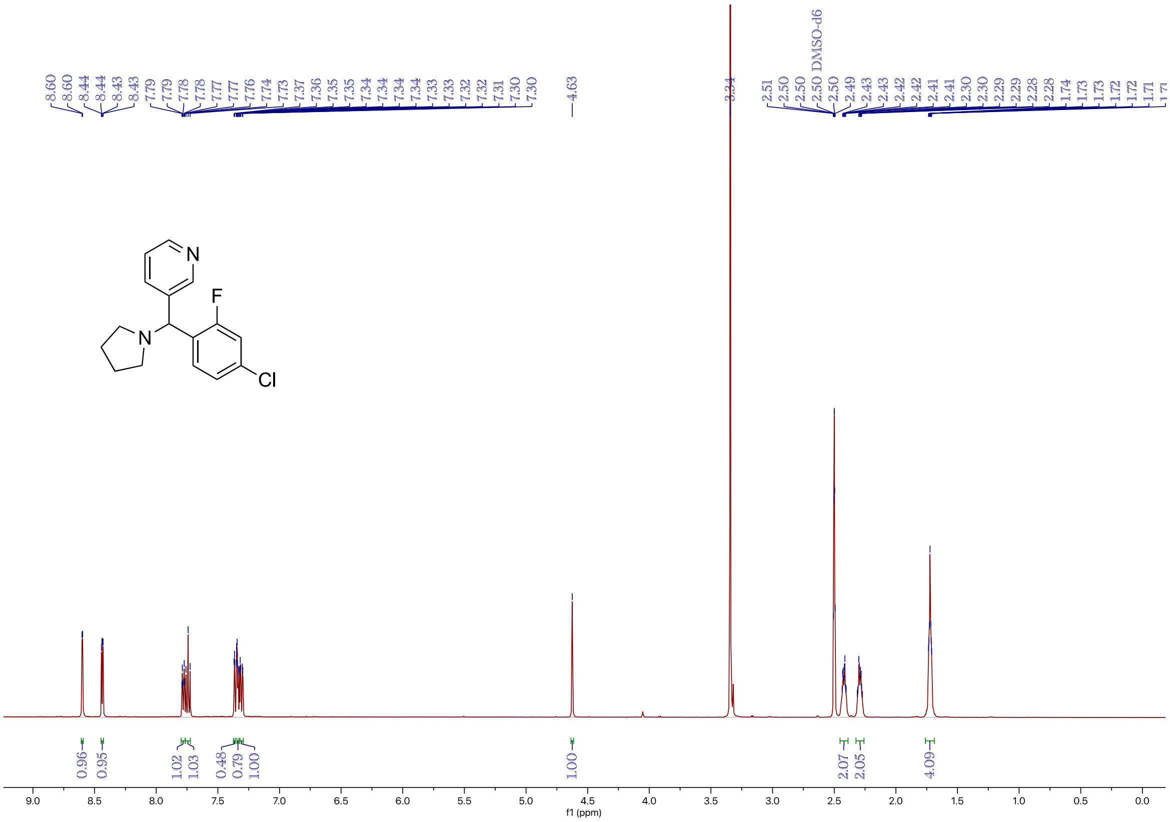

Figure S102. $^{1}H$ NMR spectrum of 3–((4–chloro–2–fluorophenyl)(pyrrolidin–1–yl)methyl)pyridine (**MYOS_00191**, 500 MHz, DMSO-$d_6$).

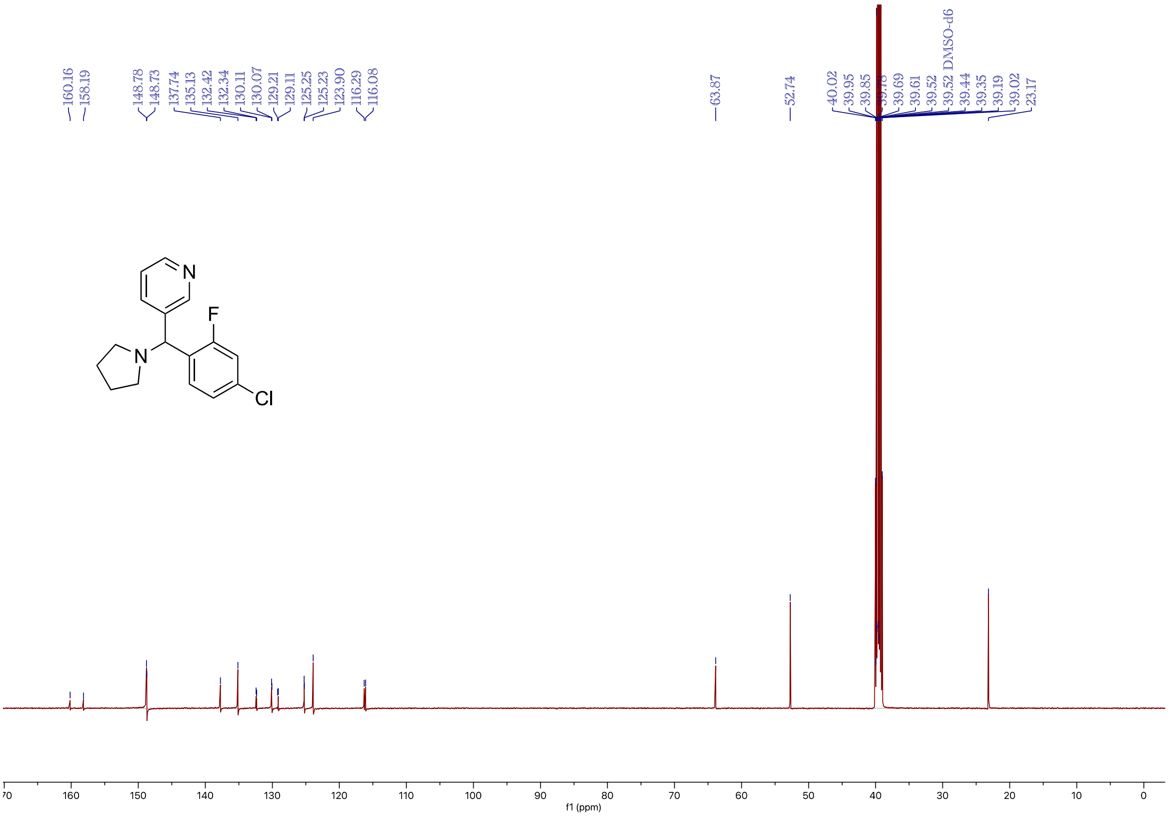

Figure S103. $^{13}C$ NMR spectrum of 3–((4–chloro–2–fluorophenyl)(pyrrolidin–1–yl)methyl)pyridine (**MYOS_00191,** 126 MHz, DMSO-$d_6$).

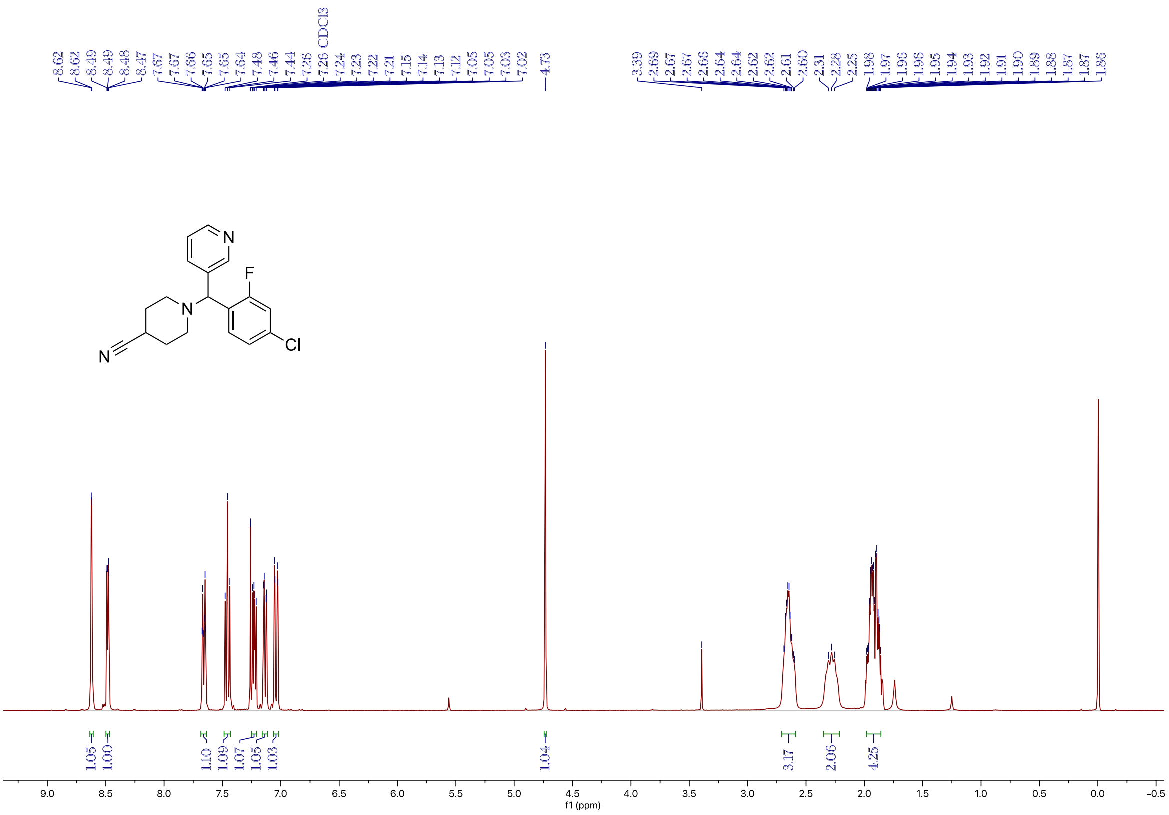


Figure S104. $^{1}$H NMR spectrum of 1–((4–chloro–2–fluorophenyl)(pyridin–3–yl)methyl)piperidine–4–carbonitrile (**MYOS_00192**, 400 MHz, $CDCl_3$).

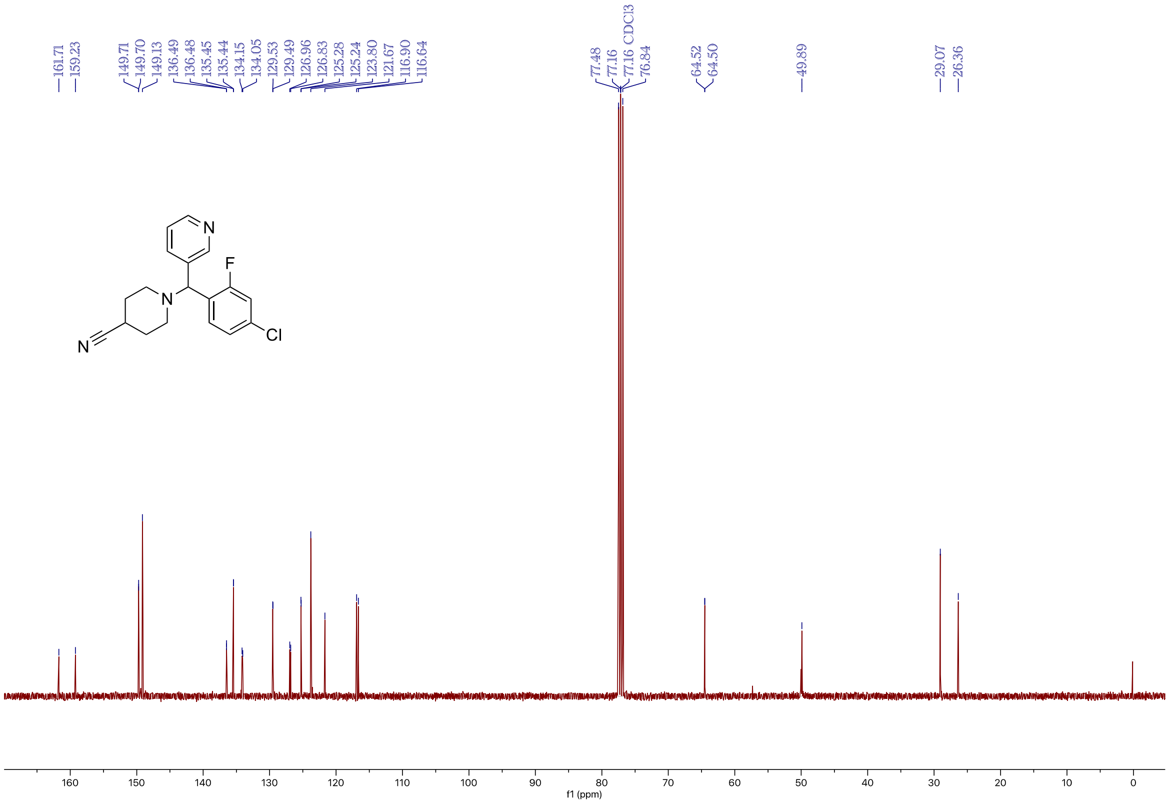


Figure S105. $^{13}$C NMR spectrum of 1–((4–chloro–2–fluorophenyl)(pyridin–3–yl)methyl)piperidine–4–carbonitrile (**MYOS_00192**, 101 MHz, $CDCl_3$).

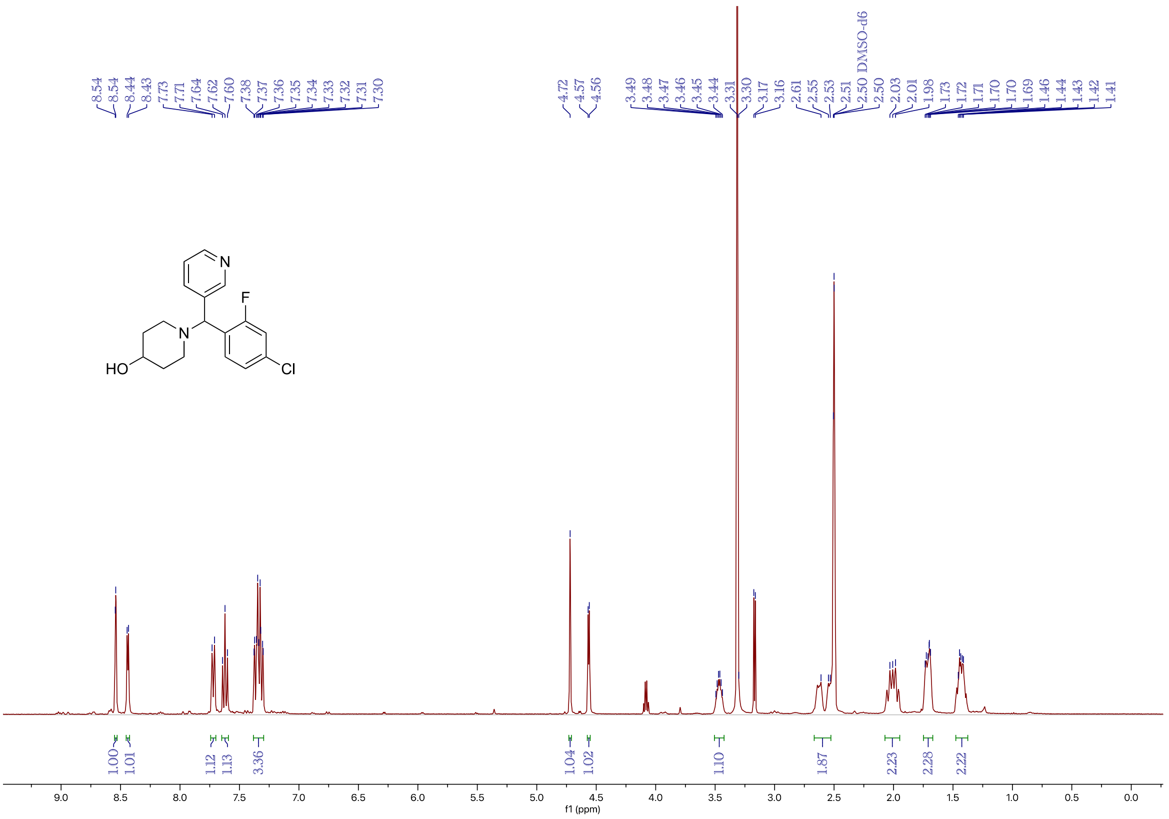


Figure S106. $^{1}$H NMR spectrum of 1–((4–chloro–2–fluorophenyl)(pyridin–3–yl)methyl)piperidin–4–ol (**MYOS_00195**, 400 MHz, DMSO-$d_6$).

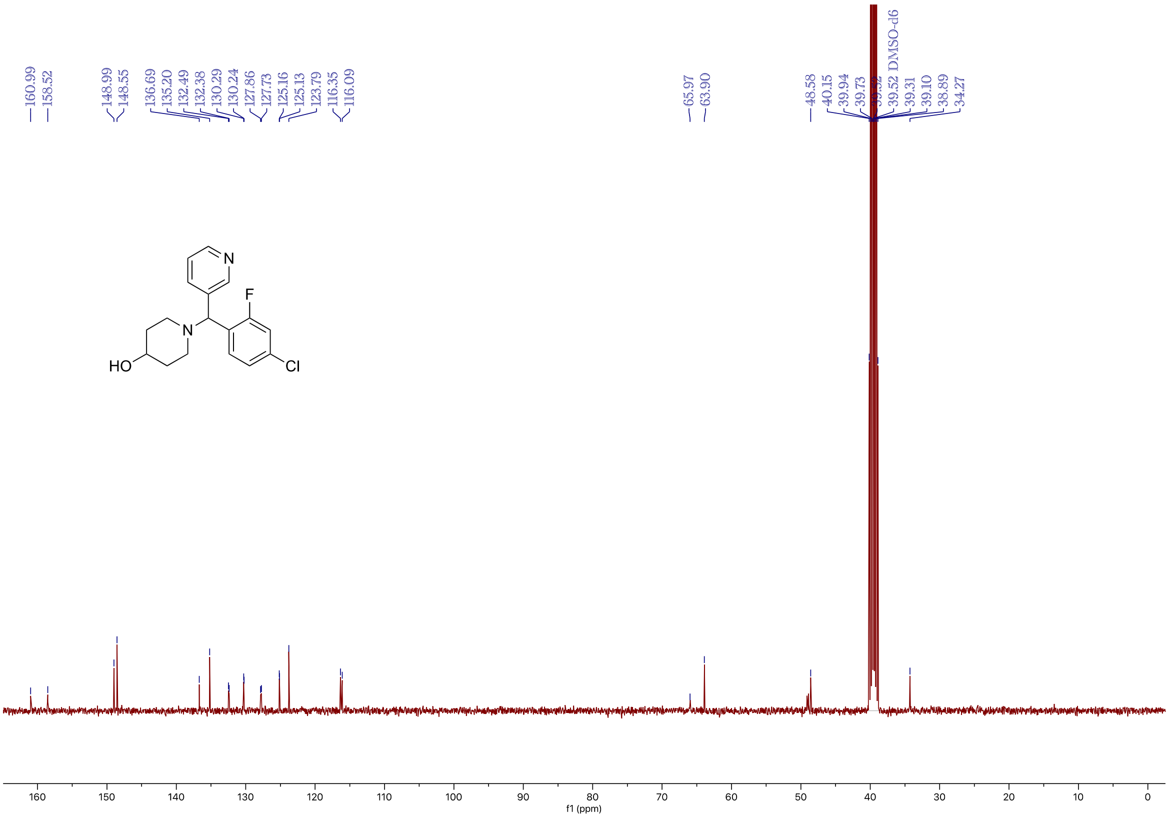


Figure S107. $^{13}$C NMR spectrum of 1–((4–chloro–2–fluorophenyl)(pyridin–3–yl)methyl)piperidin–4–ol (**MYOS_00195**, 101 MHz, DMSO-$d_6$).

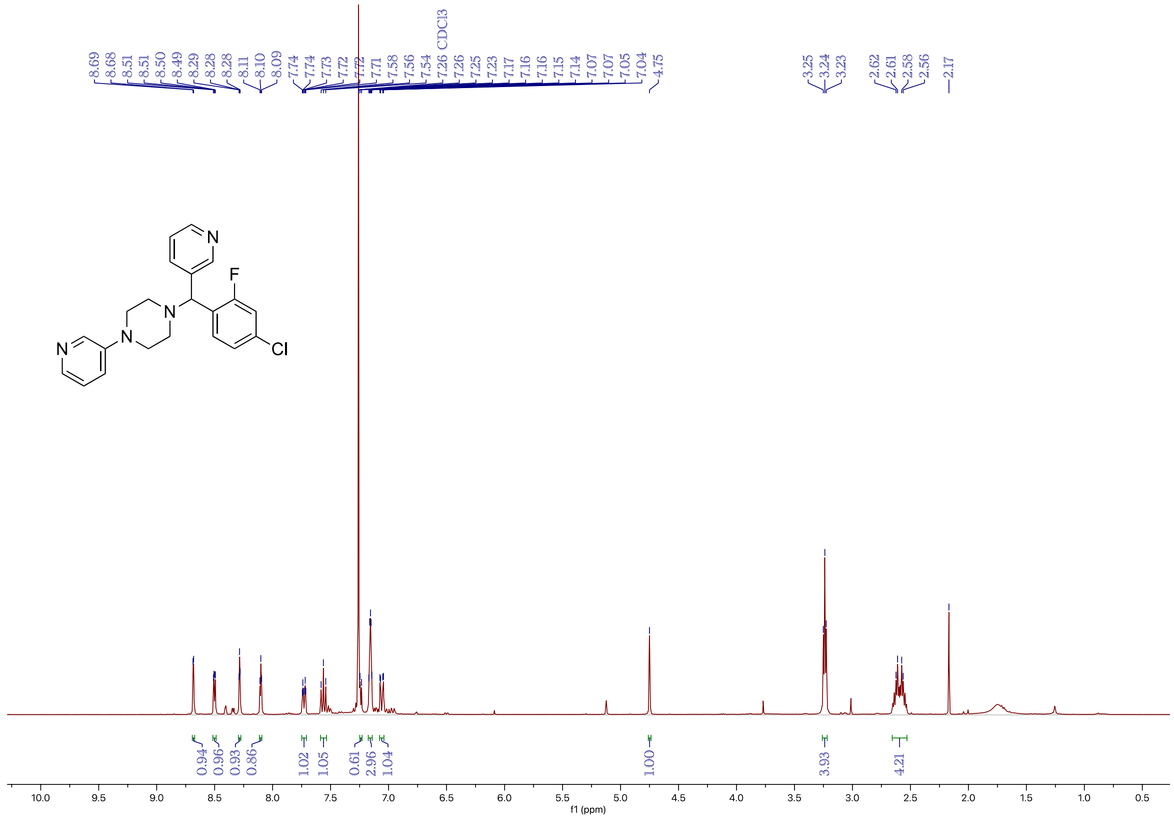

Figure S108. $^{1}H$ NMR spectrum of 1–((4–chloro–2–fluorophenyl)(pyridin–3–yl)methyl)–4–(pyridin–3–yl)piperazine (**MYOS_00196**, 400 MHz, $CDCl_3$).

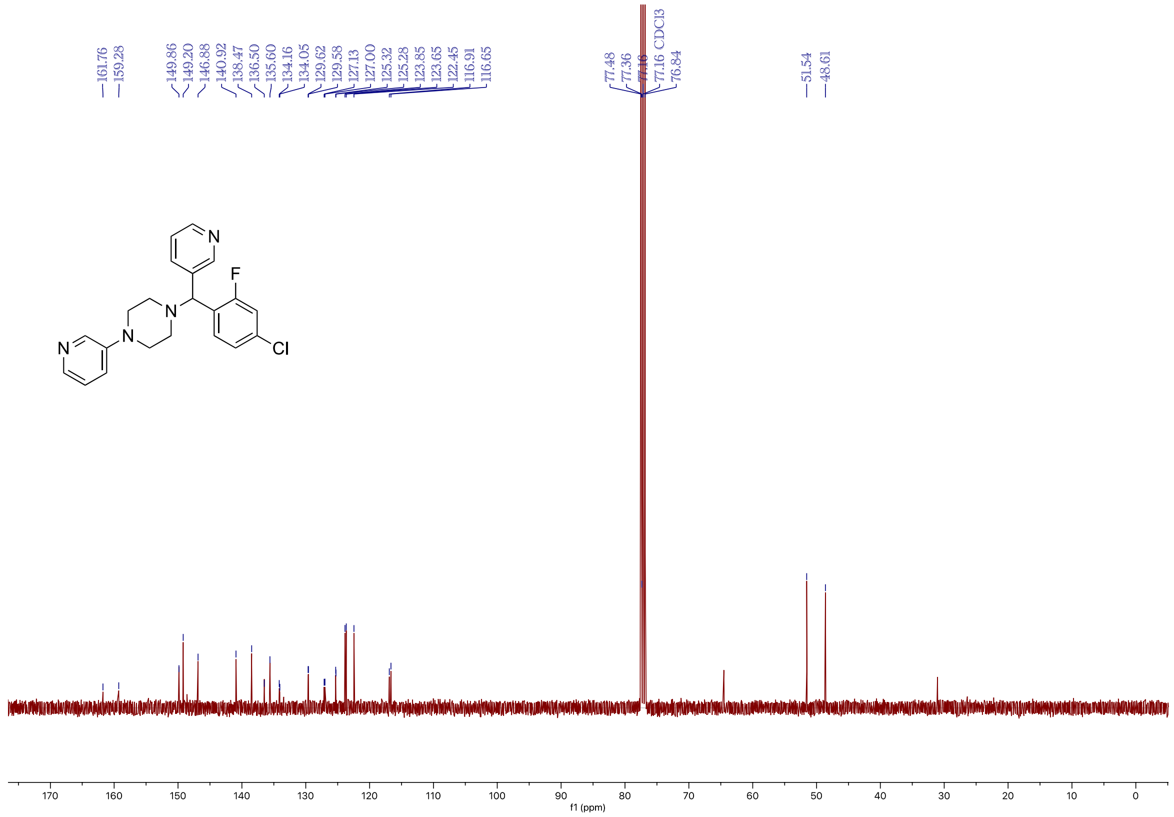

Figure S109. $^{13}C$ NMR spectrum of 1–((4–chloro–2–fluorophenyl)(pyridin–3–yl)methyl)–4–(pyridin–3–yl)piperazine (**MYOS_00196**, 101 MHz, $CDCl_3$).

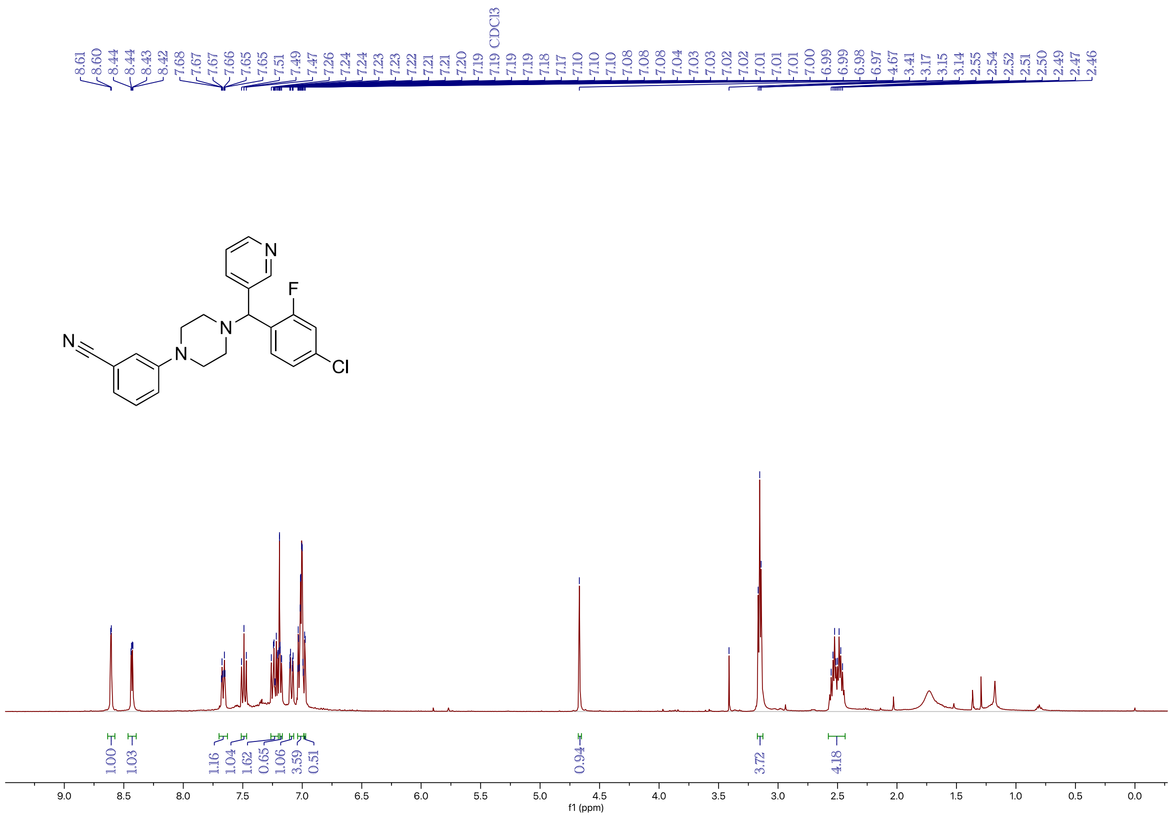


Figure S110. $^{1}$H NMR spectrum of 3–(4–((4–chloro–2–fluorophenyl)(pyridin–3–yl)methyl)piperazin–1–yl)benzonitrile (**MYOS_00197**, 400 MHz, $CDCl_3$).

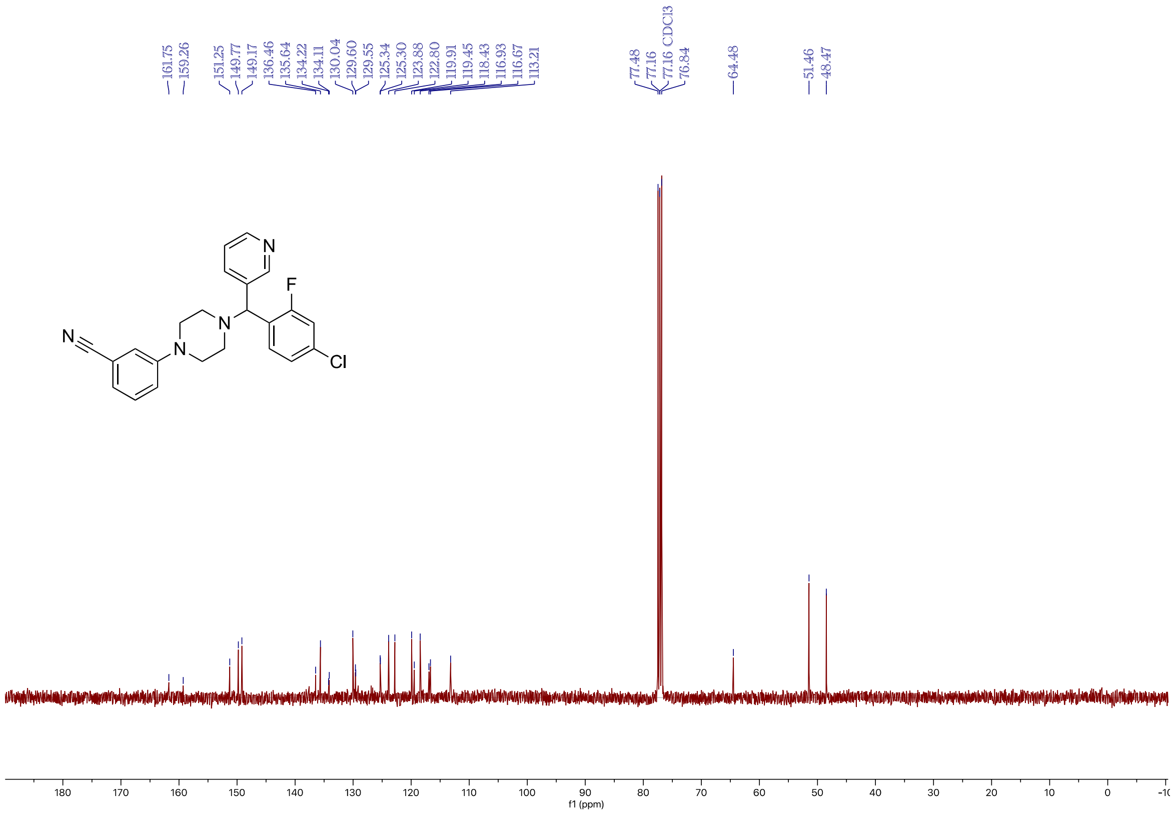


Figure S111. $^{13}$C NMR spectrum of 3–(4–((4–chloro–2–fluorophenyl)(pyridin–3–yl)methyl)piperazin–1–yl)benzonitrile (**MYOS_00197**, 101 MHz, $CDCl_3$).

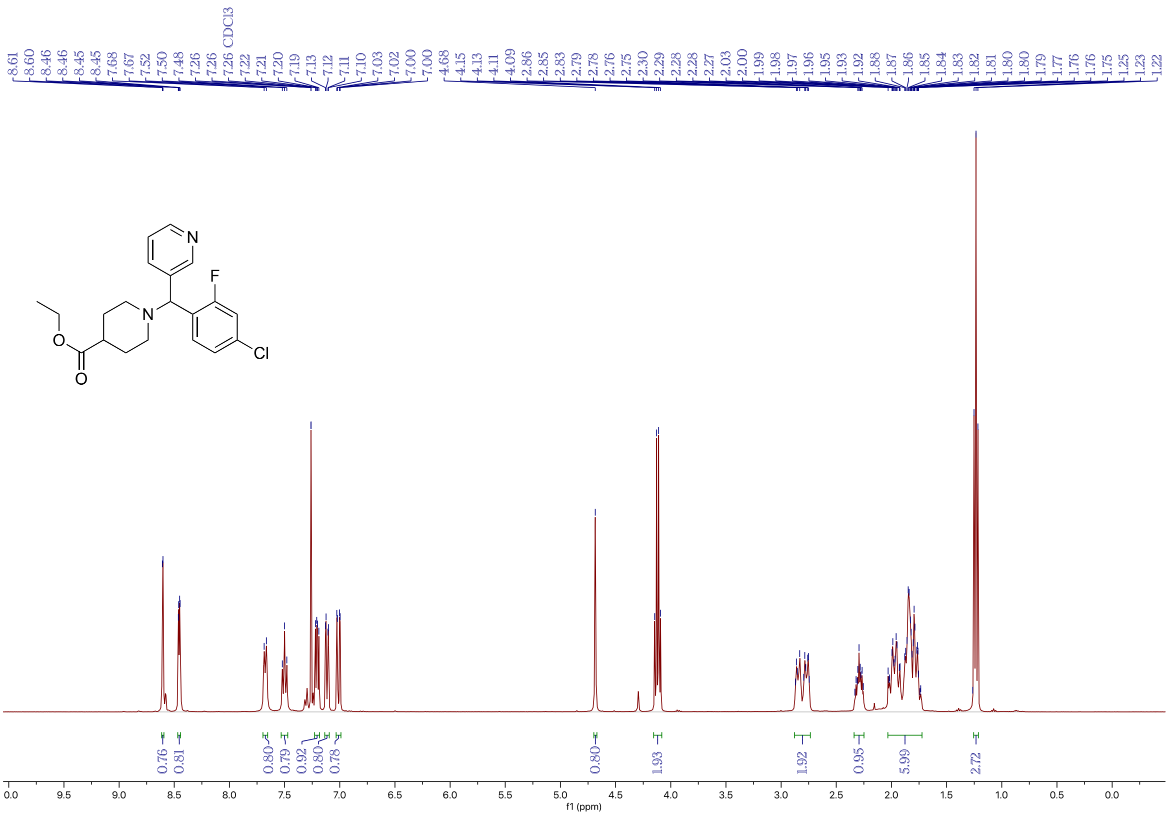


Figure S112. $^{1}$H NMR spectrum of ethyl 1–((4–chloro–2–fluorophenyl)(pyridin–3–yl)methyl)piperidine–4–carboxylate (**MYOS_00203**, 400 MHz, $CDCl_3$).

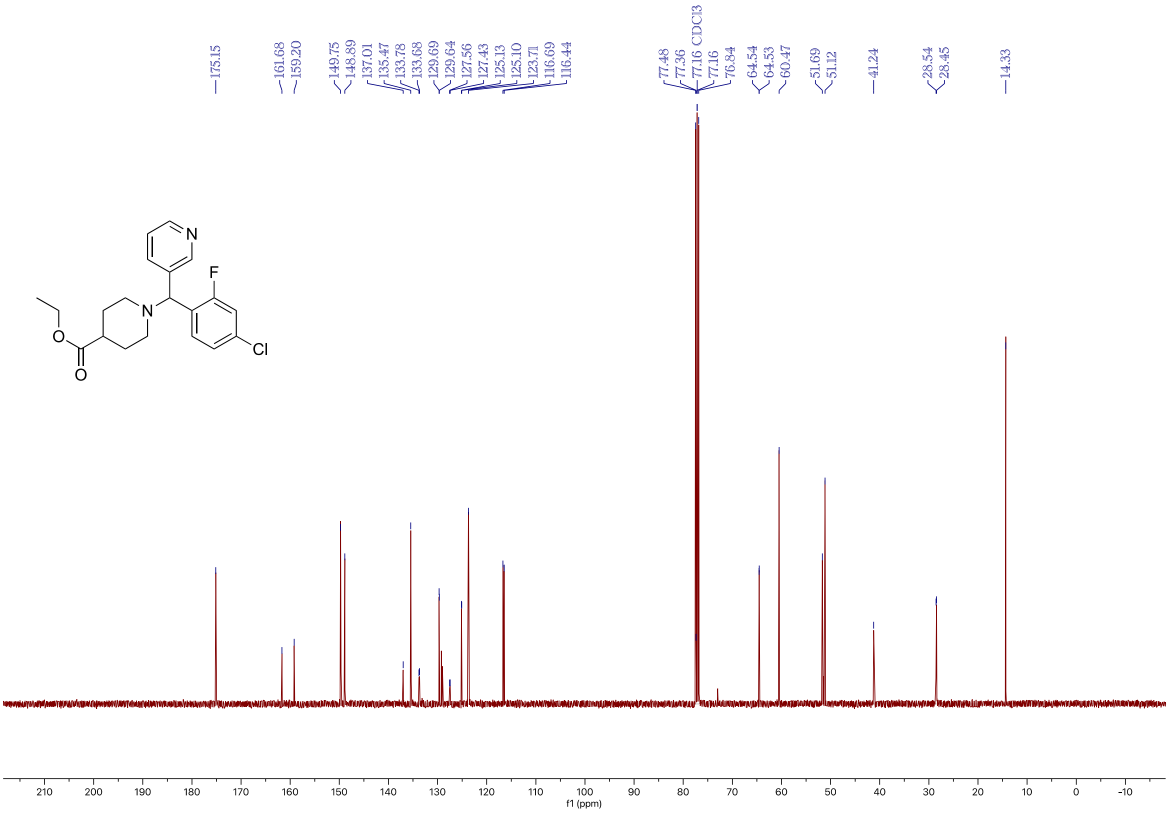


Figure S113. $^{13}$C NMR spectrum of ethyl 1–((4–chloro–2–fluorophenyl)(pyridin–3–yl)methyl)piperidine–4–carboxylate (**MYOS_00203**, 101 MHz, $CDCl_3$).

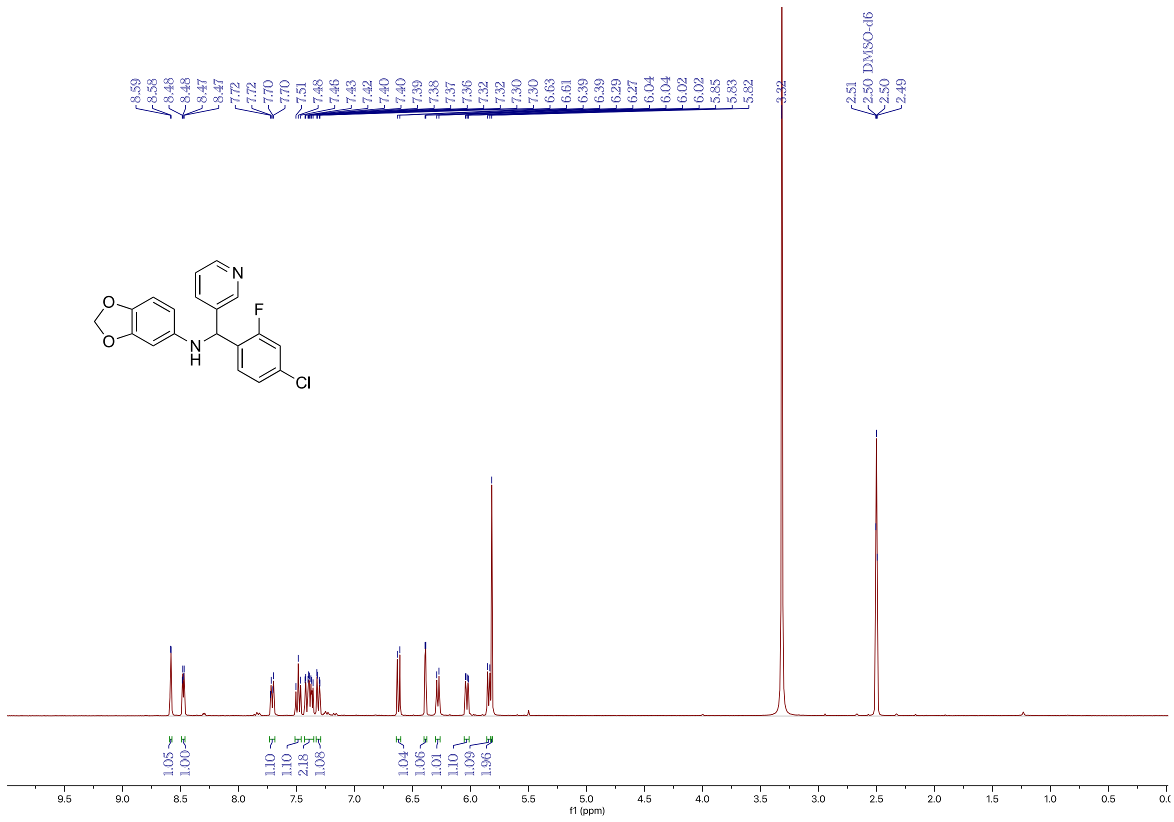

Figure S114. $^{1}$H NMR spectrum of *N*–((4–chloro–2–fluorophenyl)(pyridin–3–yl)methyl)benzo[d][1,3]dioxol–5–amine (**MYOS_00204**, 400 MHz, DMSO-$d_6$).

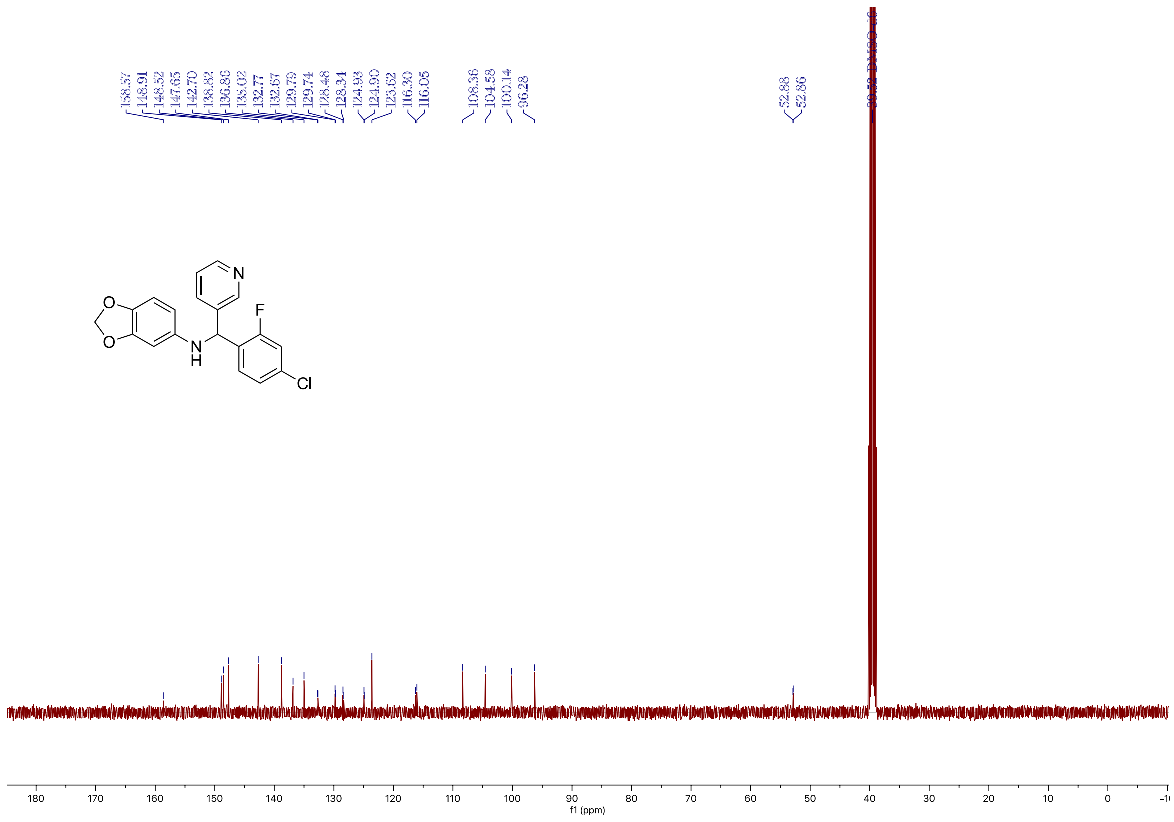

Figure S115. $^{13}$C NMR spectrum of *N*–((4–chloro–2–fluorophenyl)(pyridin–3–yl)methyl)benzo[d][1,3]dioxol–5–amine (**MYOS_00204**, 101 MHz, DMSO-$d_6$).

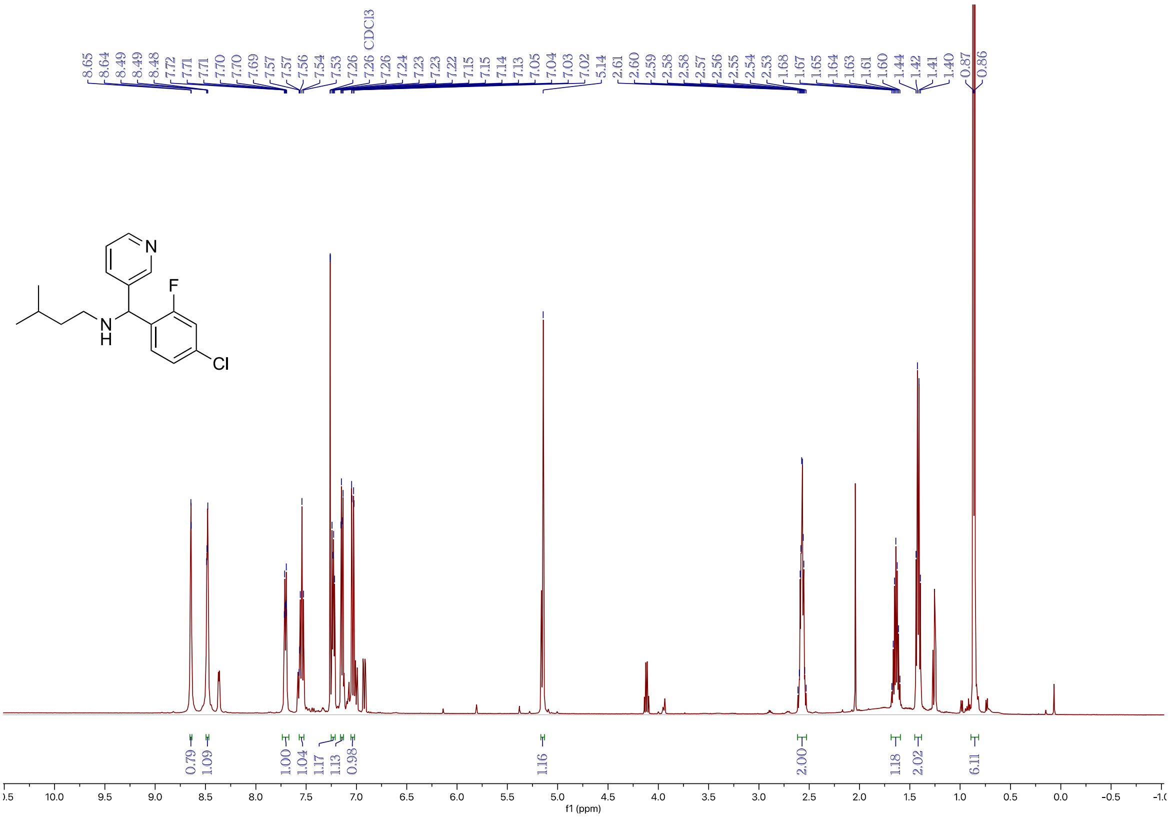


Figure S116. $^{1}H$ NMR spectrum of *N*–((4–chloro–2–fluorophenyl)(pyridin–3–yl)methyl)–3–methylbutan–1–amine (**MYOS_00205**, 500 MHz, DMSO-$d_6$).

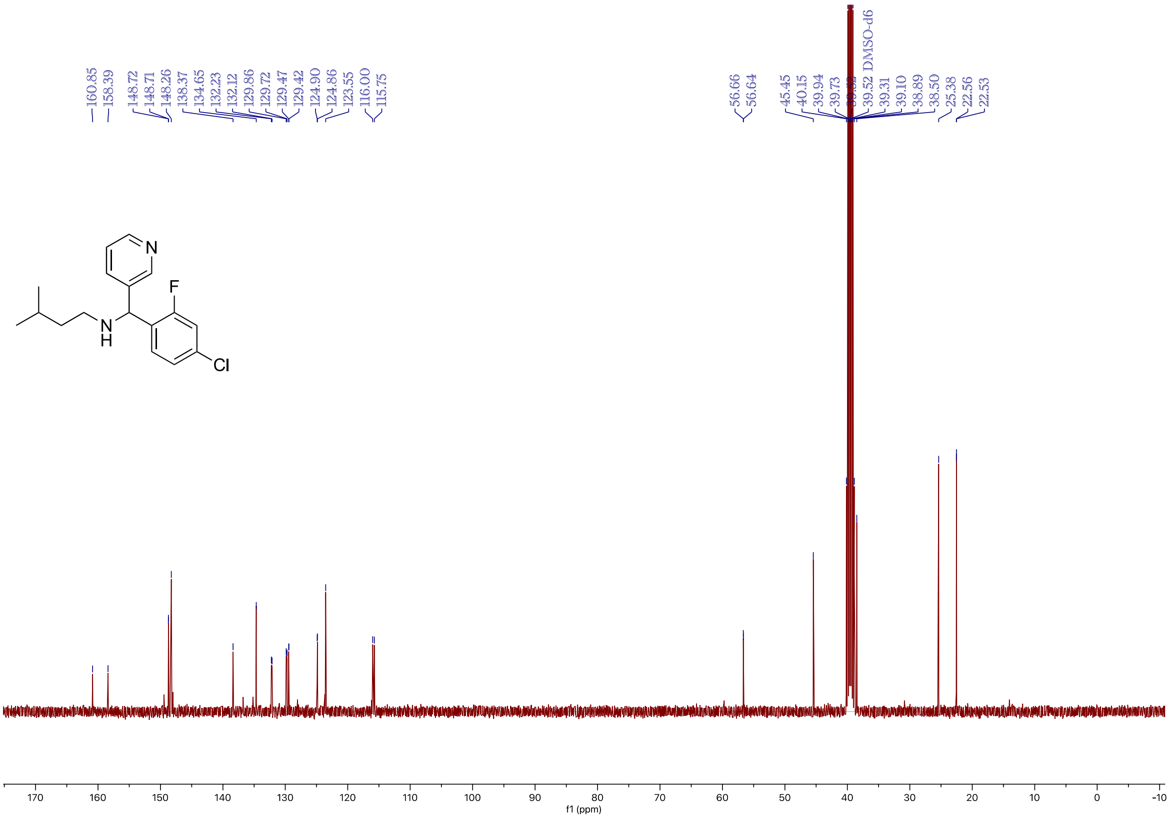


Figure S117. $^{13}C$ NMR spectrum of *N*–((4–chloro–2–fluorophenyl)(pyridin–3–yl)methyl)–3–methylbutan–1–amine (**MYOS_00205**, 101 MHz, DMSO-$d_6$).

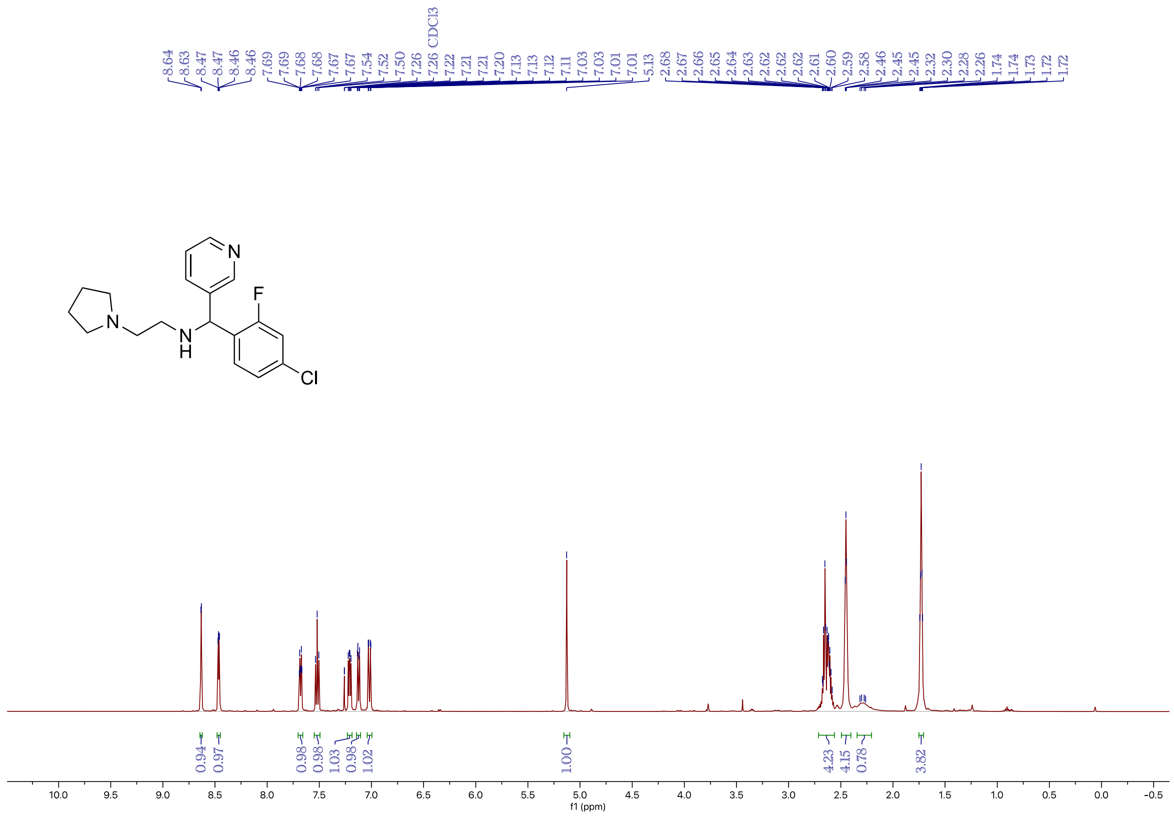

Figure S118. $^{1}$H NMR spectrum of *N*–((4–chloro–2–fluorophenyl)(pyridin–3–yl)methyl)–2–(pyrrolidin–1–yl)ethan–1–amine (**MYOS_00206**, 400 MHz, DMSO-$d_6$).

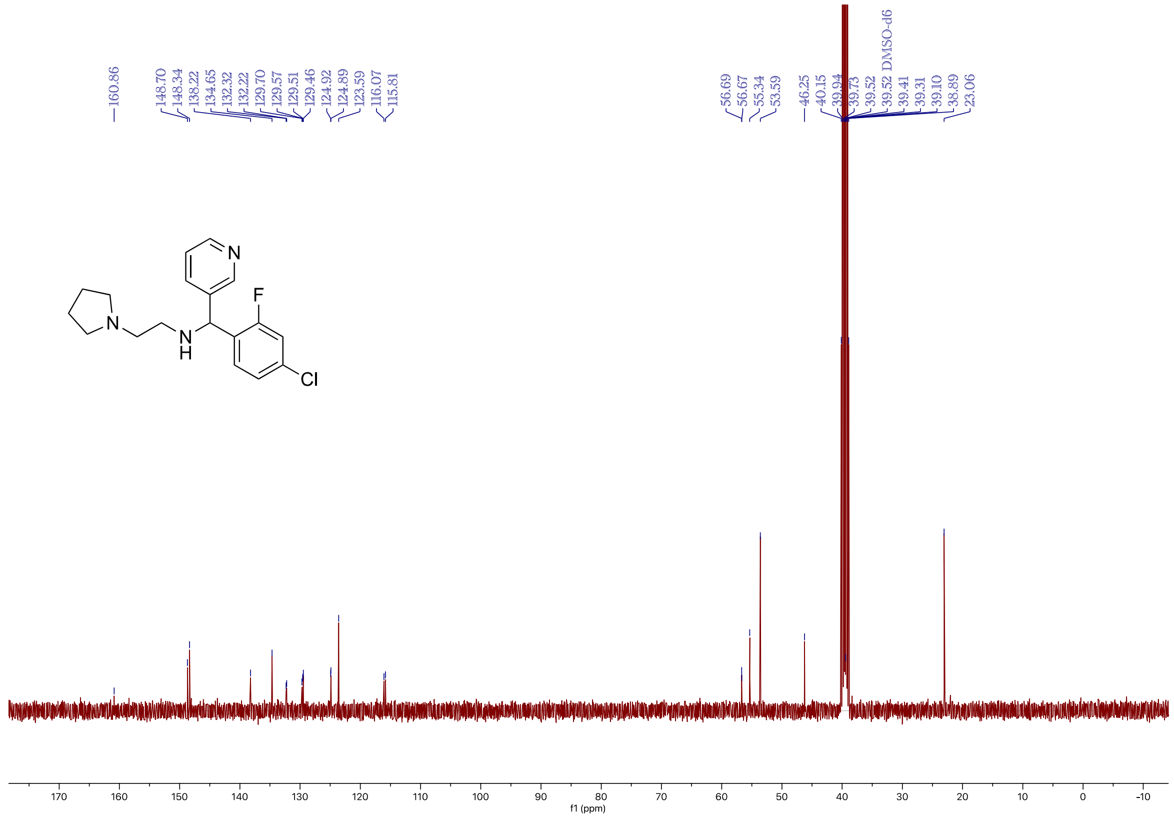

Figure S119. $^{13}$C NMR spectrum of *N*–((4–chloro–2–fluorophenyl)(pyridin–3–yl)methyl)–2–(pyrrolidin–1–yl)ethan–1–amine (**MYOS_00206**, 101 MHz, DMSO-$d_6$).

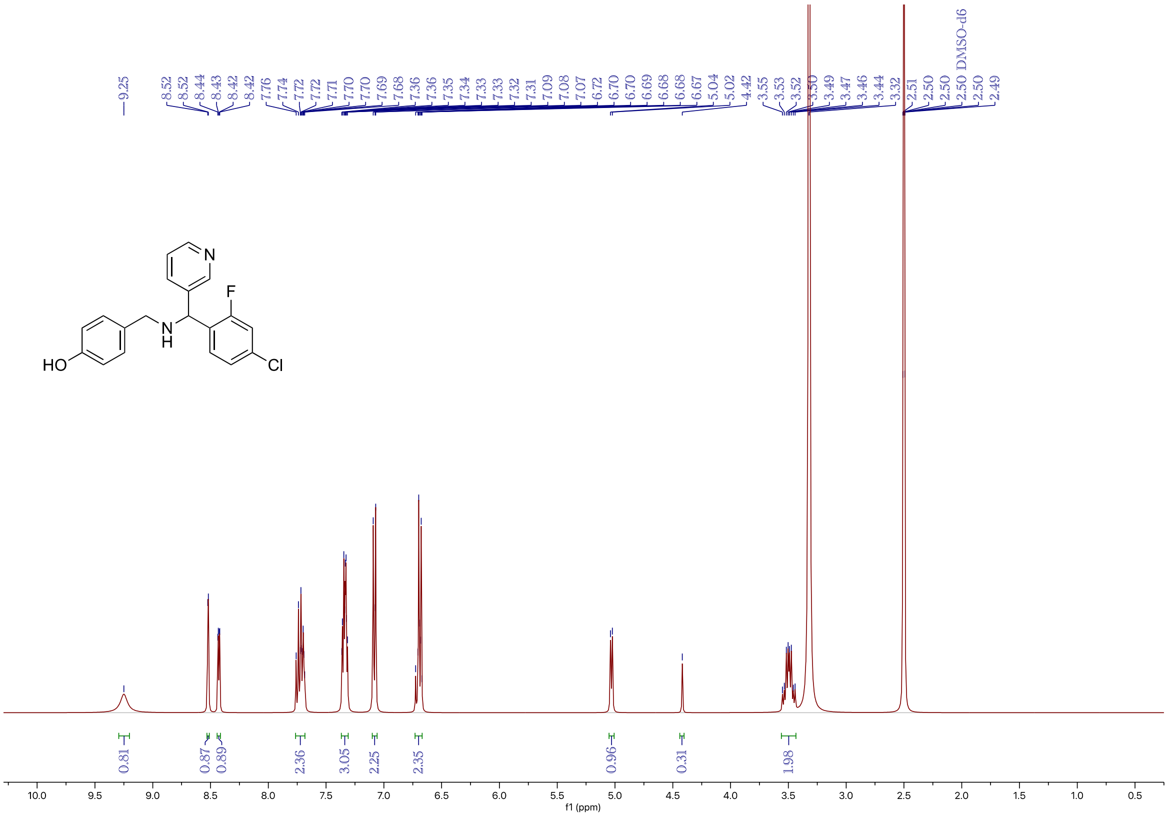

Figure S120. $^{1}$H NMR spectrum of 4–((((4–chloro–2–fluorophenyl)(pyridin–3–yl)methyl)amino)methyl)phenol (**MYOS_00310**, 400 MHz, DMSO-$d_6$).

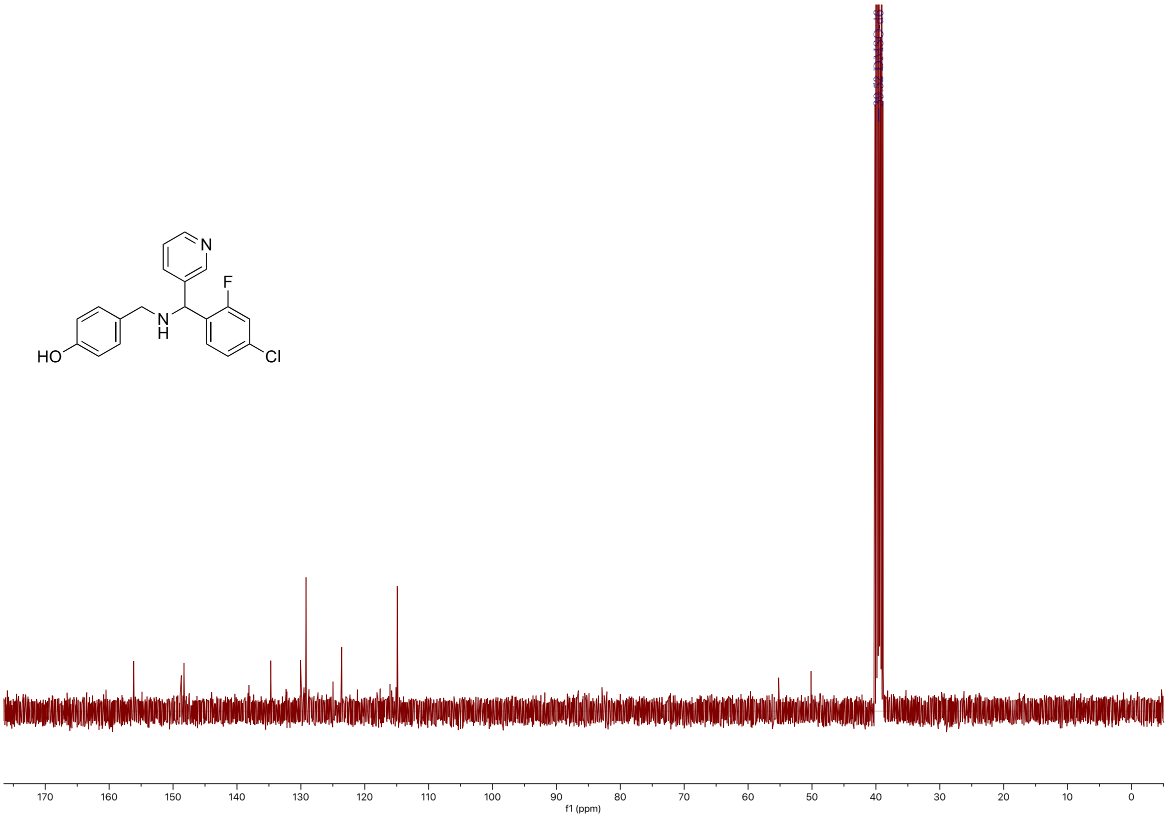

Figure S121. $^{13}$C NMR spectrum of 4–((((4–chloro–2–fluorophenyl)(pyridin–3–yl)methyl)amino)methyl)phenol (**MYOS_00310**, 101 MHz, DMSO-$d_6$).

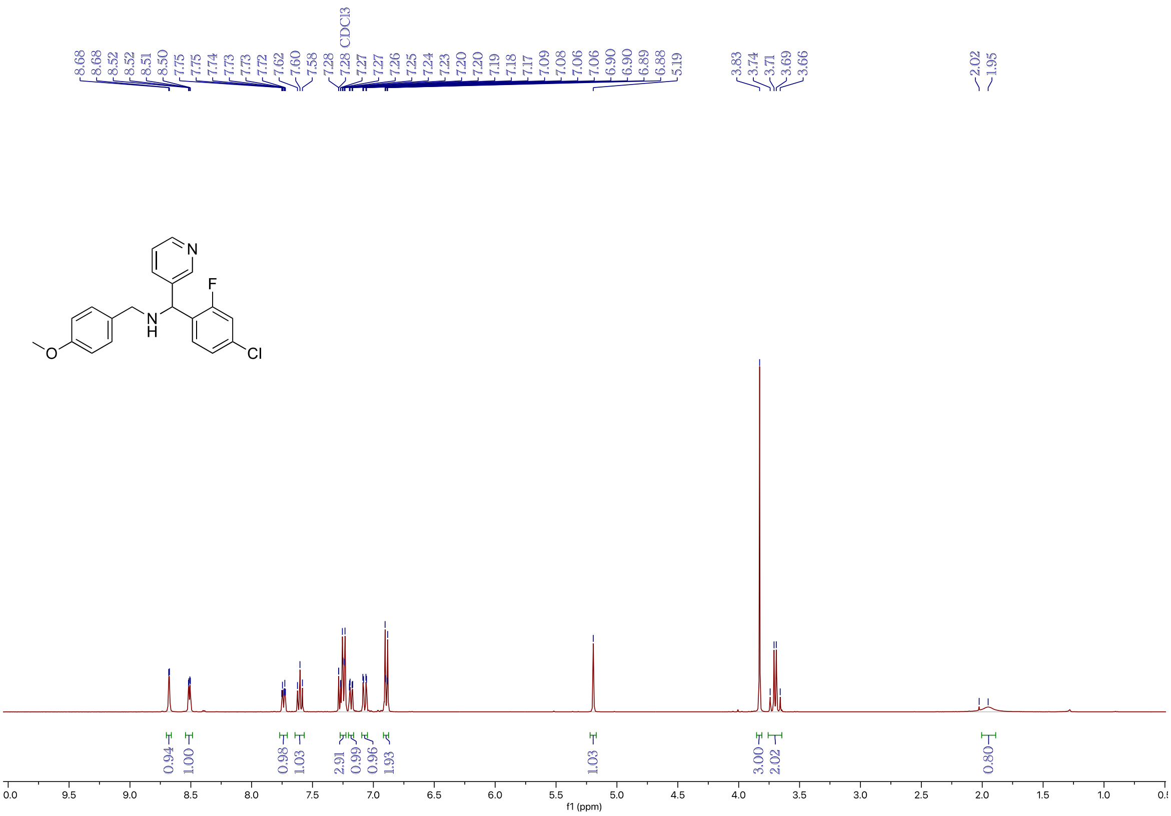

Figure S122. $^{1}$H NMR spectrum of 1–(4–chloro–2–fluorophenyl)–*N*–(4–methoxybenzyl)–1–(pyridin–3–yl)methanamine (**MYOS_00311**, 400 MHz, $CDCl_3$).

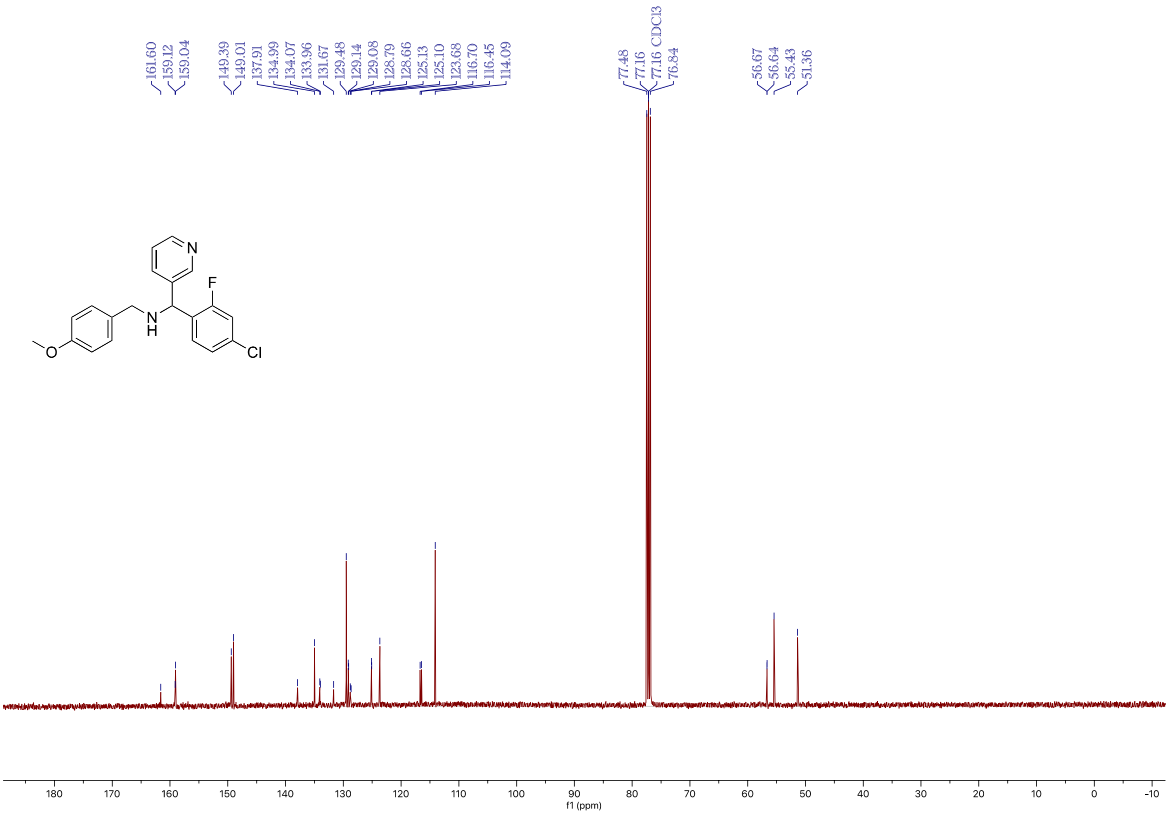

Figure S123. $^{13}$C NMR spectrum of 1–(4–chloro–2–fluorophenyl)–*N*–(4–methoxybenzyl)–1–(pyridin–3–yl)methanamine (**MYOS_00311**, 101 MHz, $CDCl_3$).

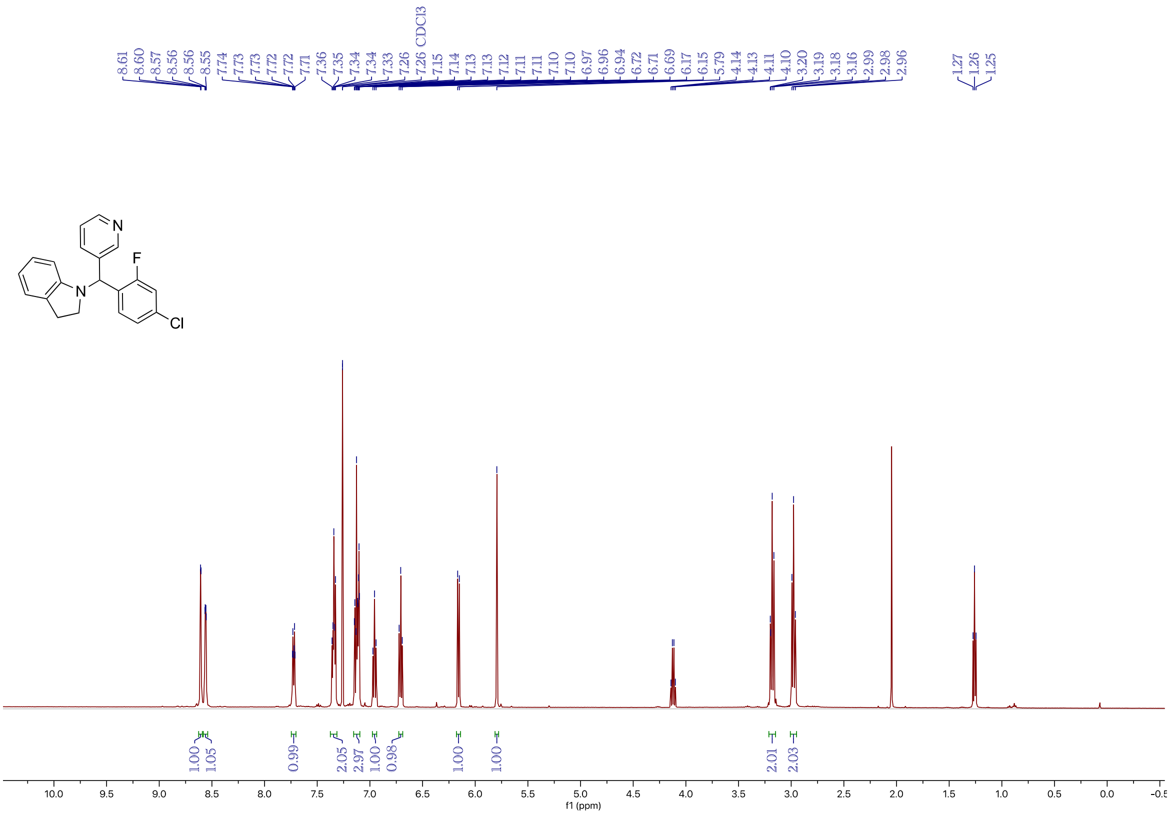


Figure S124. $^{1}$H NMR spectrum of 1–((4–chloro–2–fluorophenyl)(pyridin–3–yl)methyl)indoline (**MYOS_00321**, 500 MHz, $CDCl_3$).

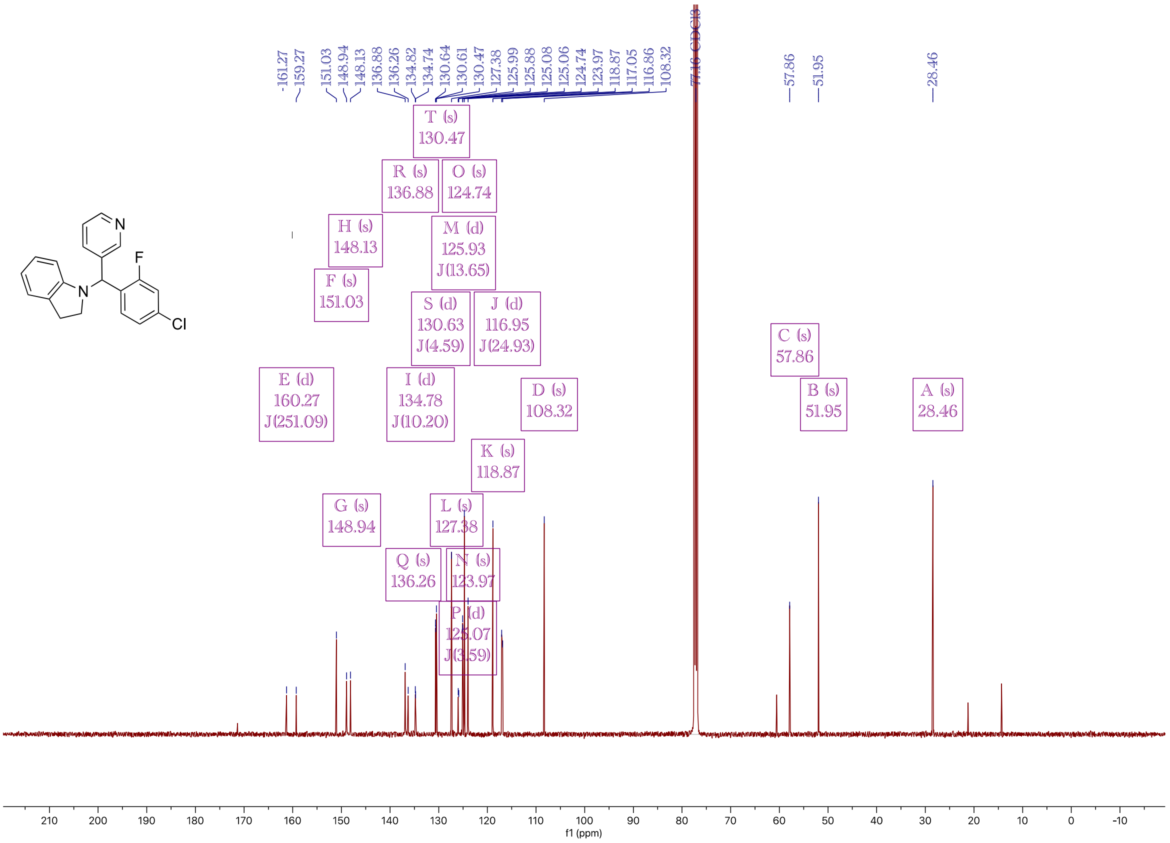


Figure S125. $^{13}$C NMR spectrum of 1–((4–chloro–2–fluorophenyl)(pyridin–3–yl)methyl)indoline (**MYOS_00321**, 126 MHz, $CDCl_3$).

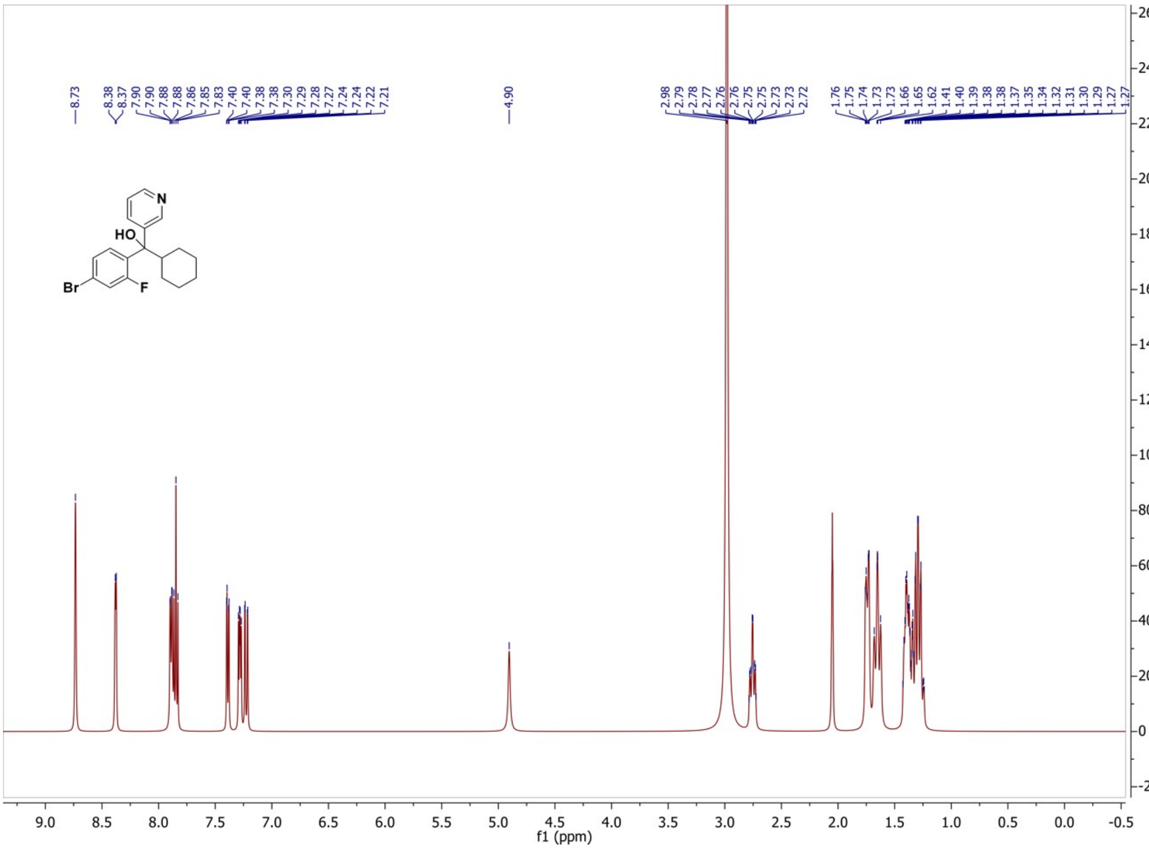

Figure S126. $^{1}$H NMR spectrum of (4-bromo-2-fluorophenyl)(cyclohexyl)(pyridin-3-yl)methanol (**MYOS_00446**, 500 MHz, Acetone-$d_6$).

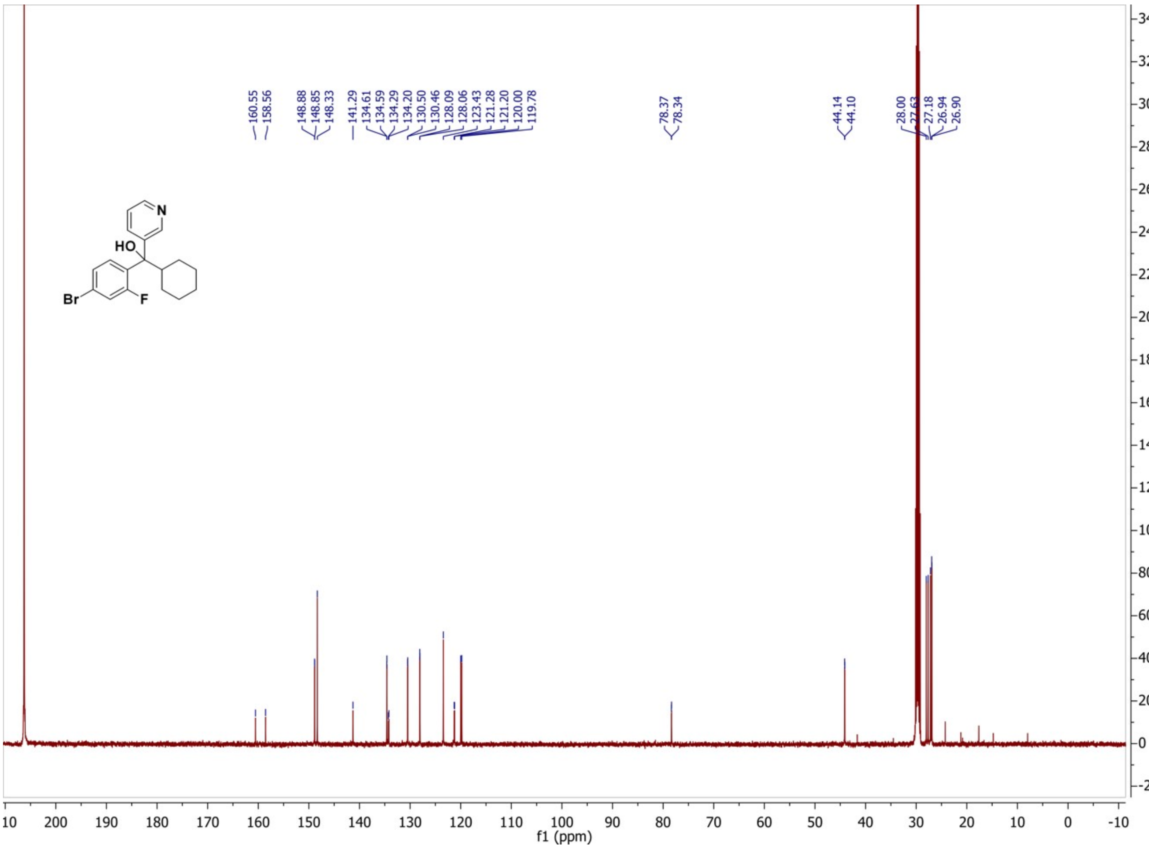

Figure S127. $^{13}$C NMR spectrum of (4-bromo-2-fluorophenyl)(cyclohexyl)(pyridin-3-yl)methanol (**MYOS_00446**, 126 MHz, Acetone-$d_6$).

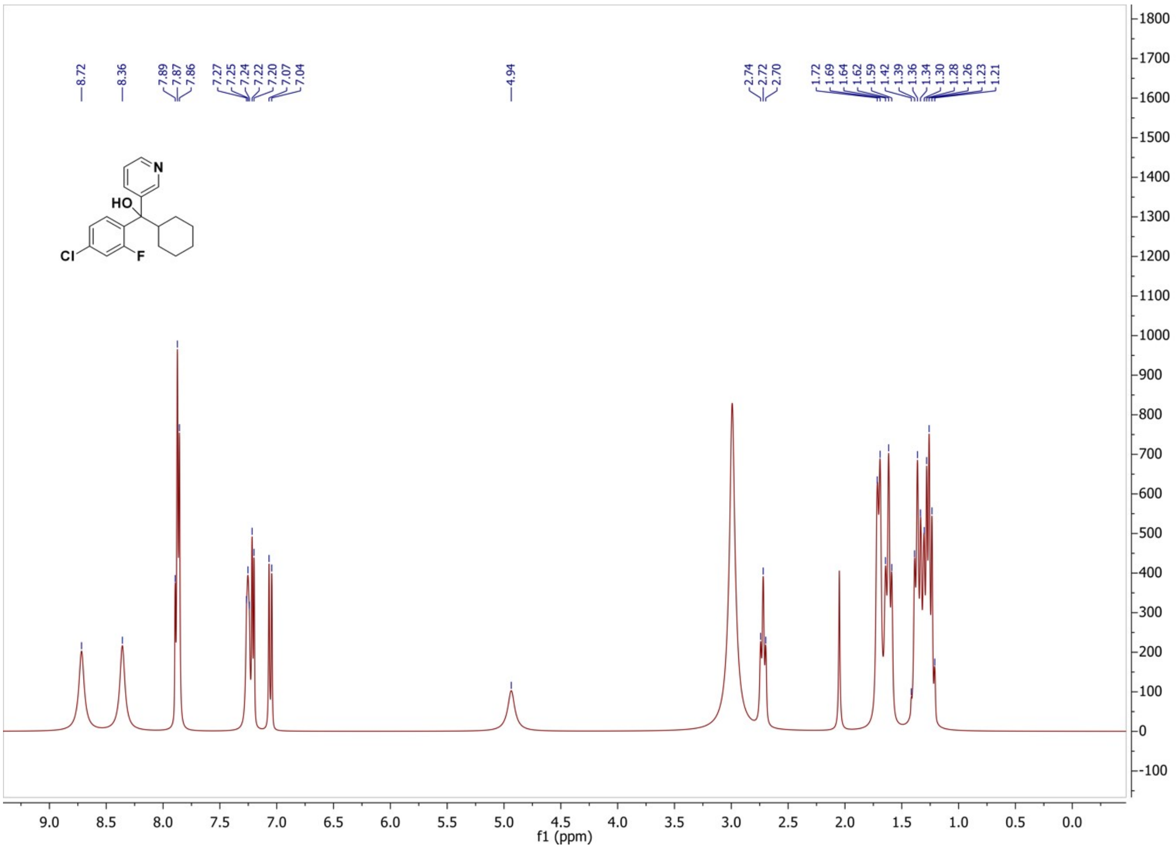


Figure S128. $^{1}$H NMR spectrum of (4-chloro-2-fluorophenyl)(cyclohexyl)(pyridin-3-yl)methanol (**MYOS_00447**, 500 MHz, Acetone-$d_6$).

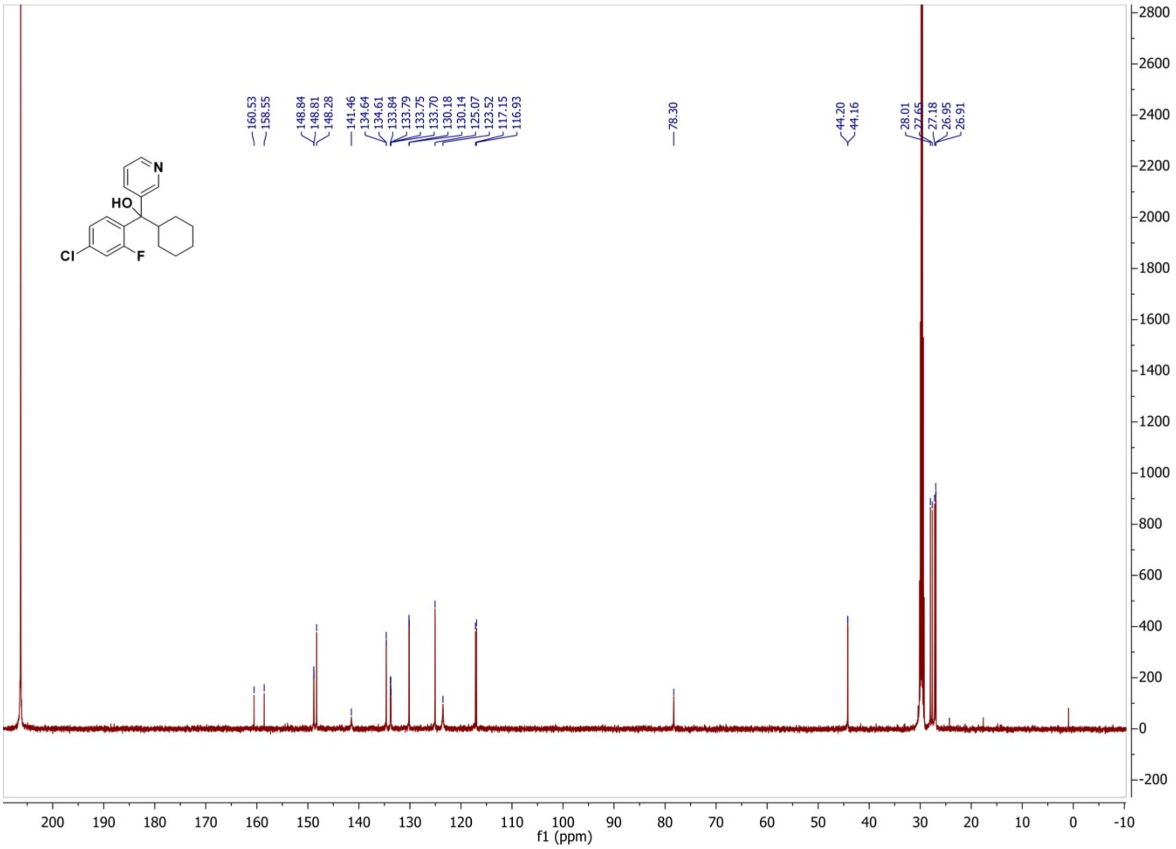


Figure S129. $^{13}$C NMR spectrum of (4-chloro-2-fluorophenyl)(cyclohexyl)(pyridin-3-yl)methanol (**MYOS_00447**, 126 MHz, Acetone-$d_6$).

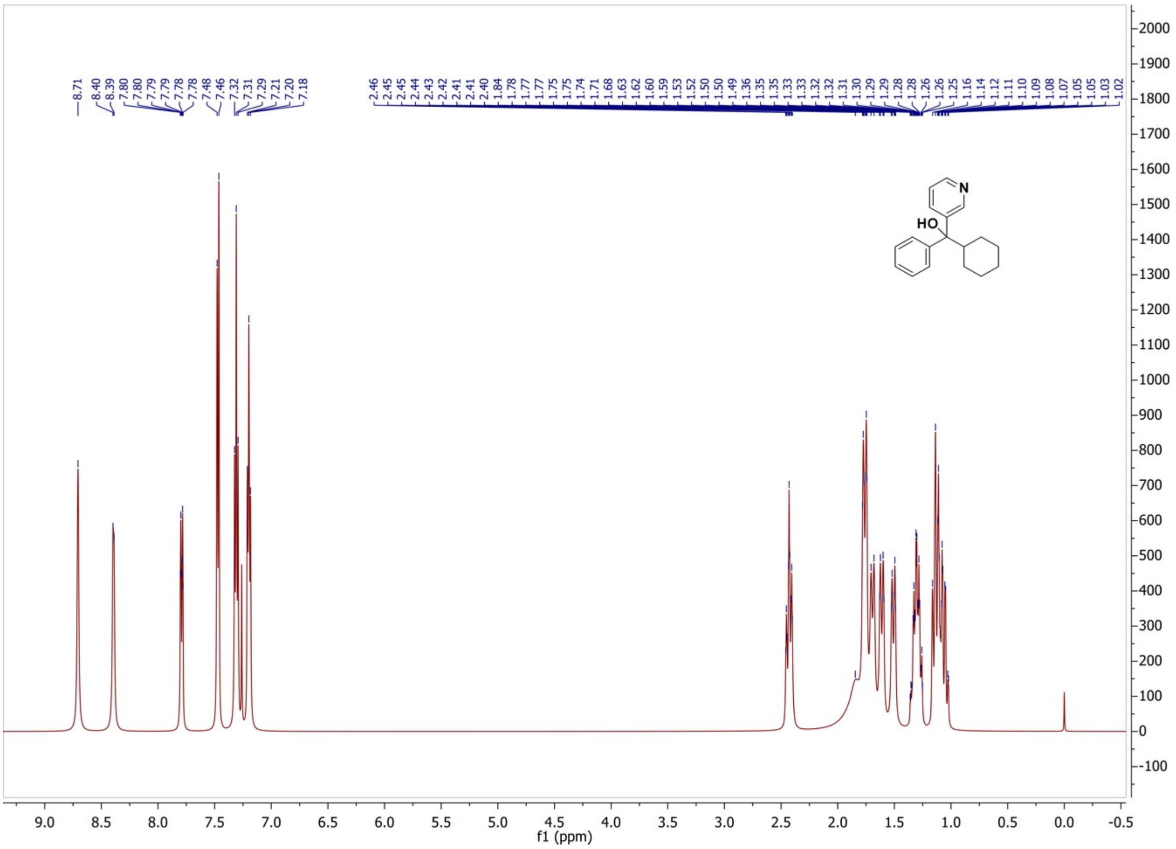

Figure S130. $^{1}H$ NMR spectrum of cyclohexyl(phenyl)(pyridin-3-yl)methanol (**MYOS_00448**, 500 MHz, $CDCl_3$).

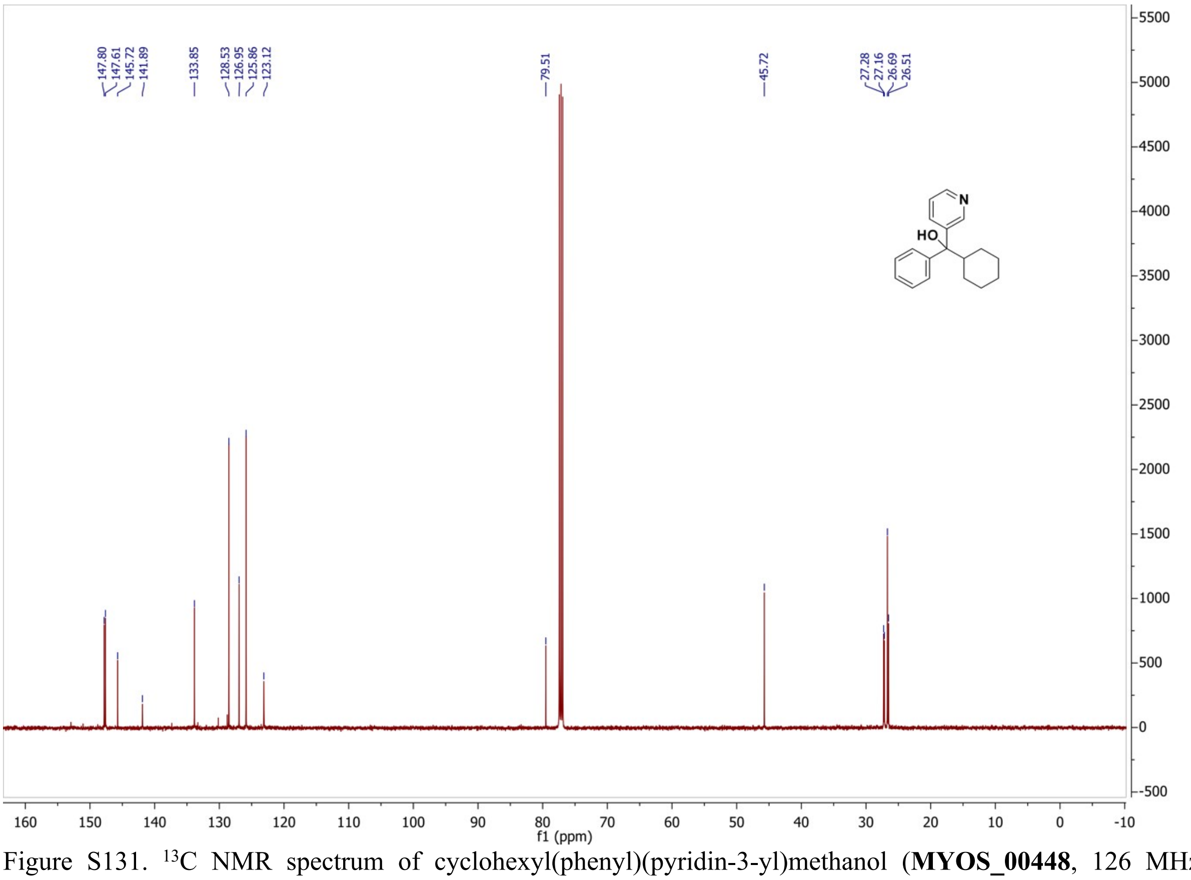

Figure S131. $^{13}C$ NMR spectrum of cyclohexyl(phenyl)(pyridin-3-yl)methanol (**MYOS_00448**, 126 MHz, $CDCl_3$).

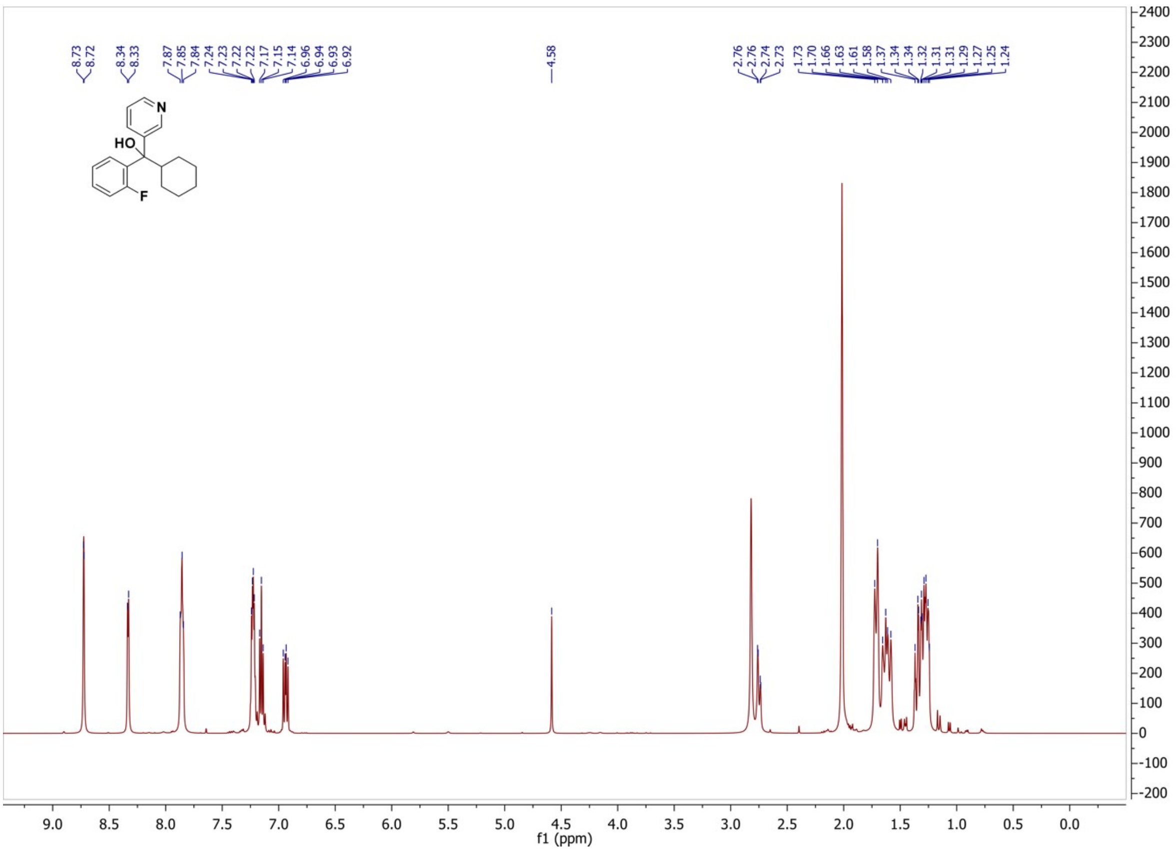

Figure S132. $^{1}H$ NMR spectrum of cyclohexyl(2-fluorophenyl)(pyridin-3-yl)methanol (**MYOS_00449**, 500 MHz, Acetone-$d_6$).

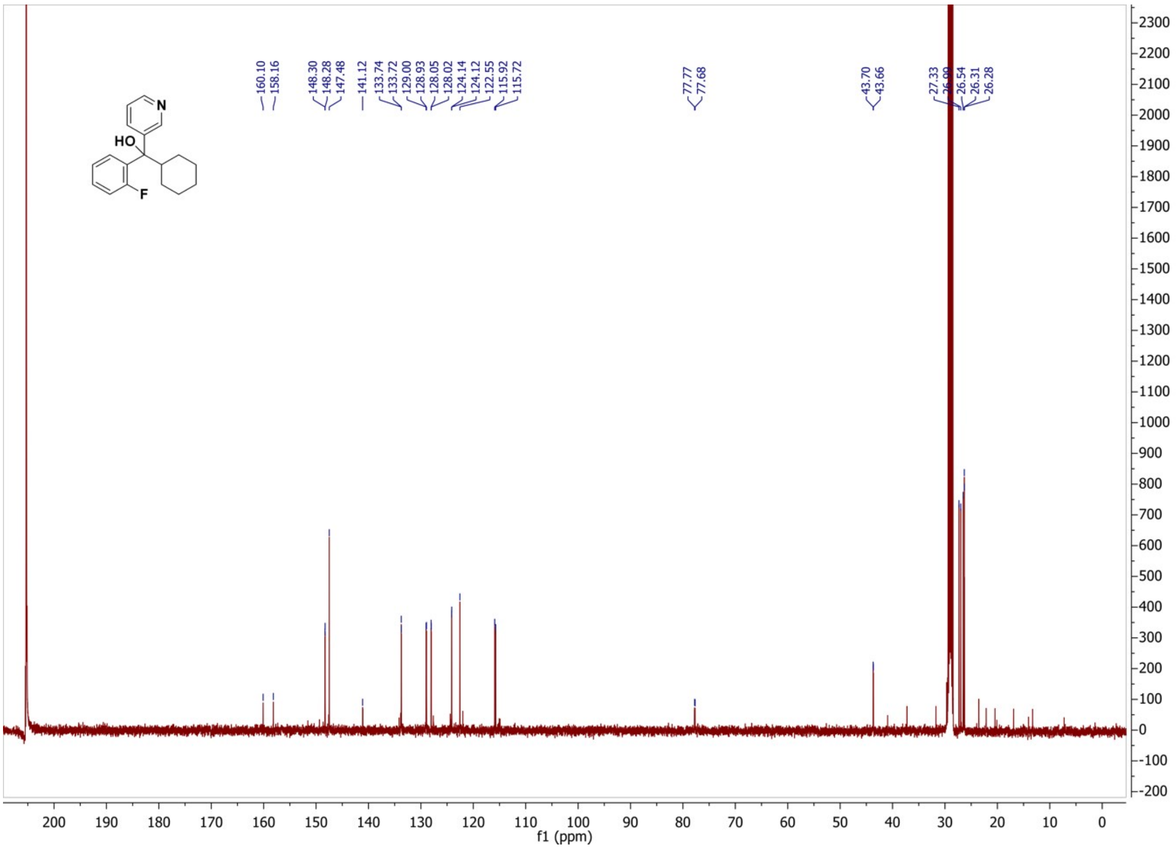

Figure S133. $^{13}C$ NMR spectrum of cyclohexyl(2-fluorophenyl)(pyridin-3-yl)methanol (**MYOS_00449**, 126 MHz, Acetone-$d_6$).

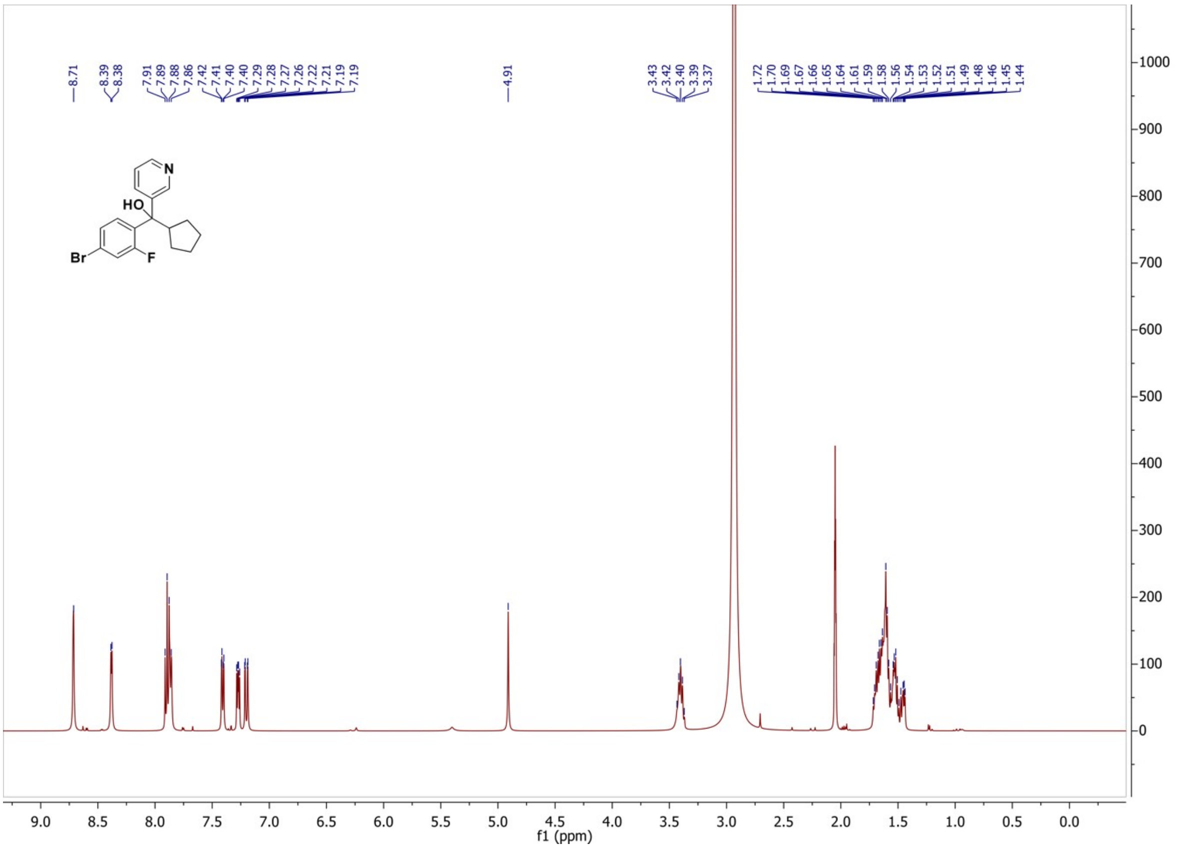

Figure S134. $^{1}H$ NMR spectrum of (4-bromo-2-fluorophenyl)(cyclopentyl)(pyridin-3-yl)methanol (**MYOS_00450**, 500 MHz, Acetone-$d_6$).

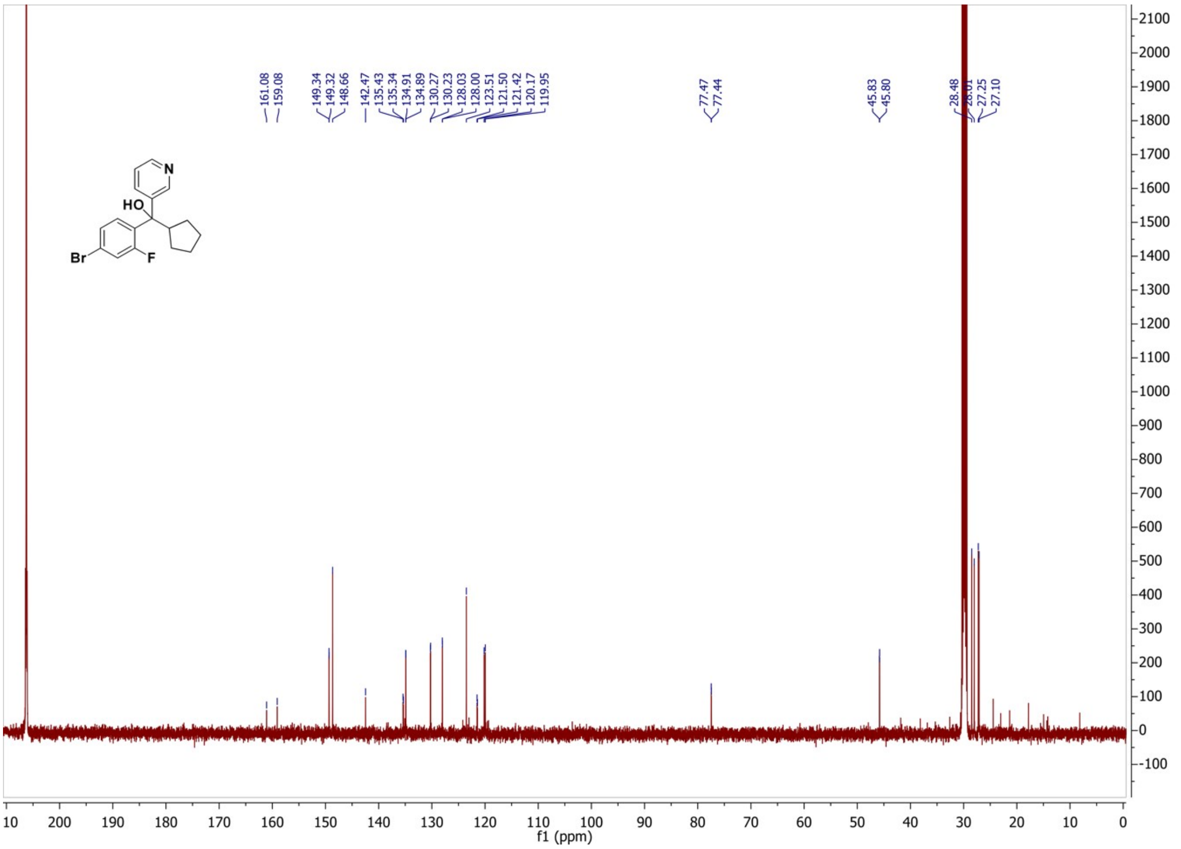

Figure S135. $^{13}C$ NMR spectrum of (4-bromo-2-fluorophenyl)(cyclopentyl)(pyridin-3-yl)methanol (**MYOS_00450**, 126 MHz, Acetone-$d_6$).

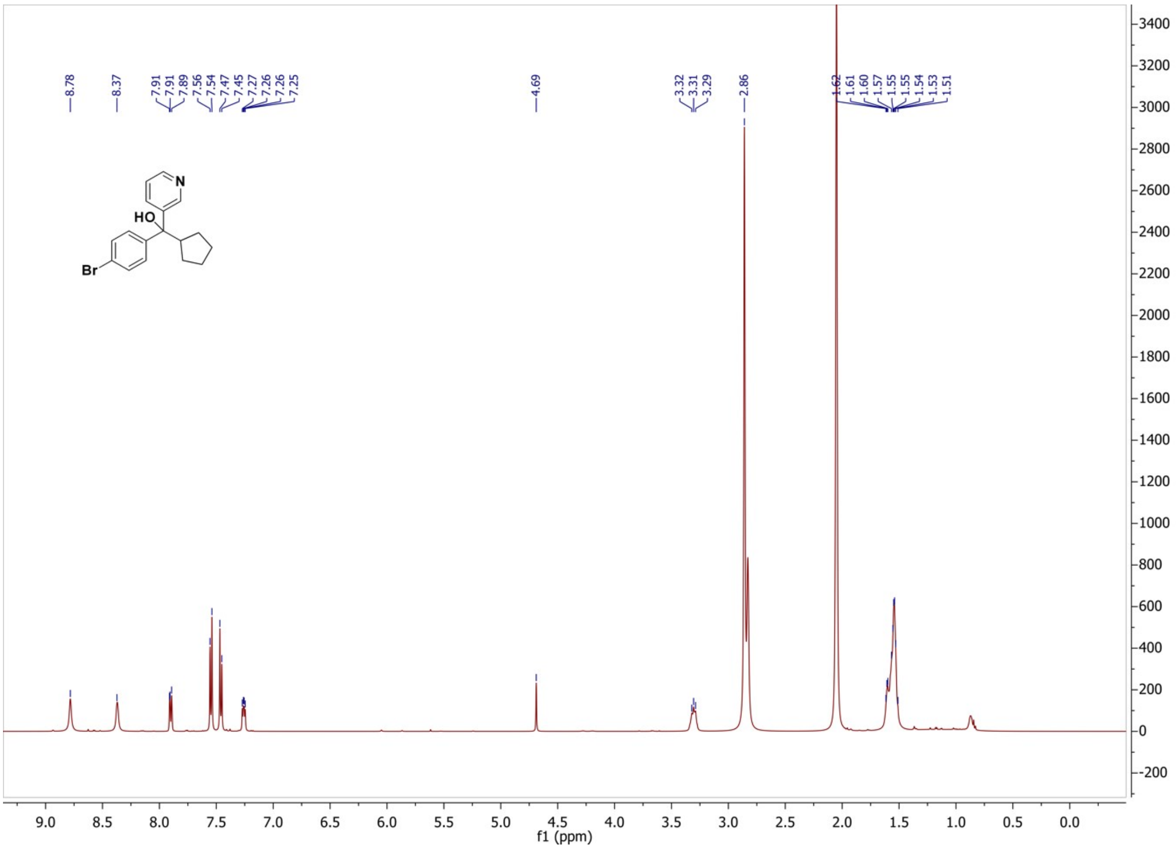


Figure S136. $^{1}$H NMR spectrum of (4-bromophenyl)(cyclopentyl)(pyridin-3-yl)methanol (**MYOS_00451**, 500 MHz, Acetone-$d_6$).

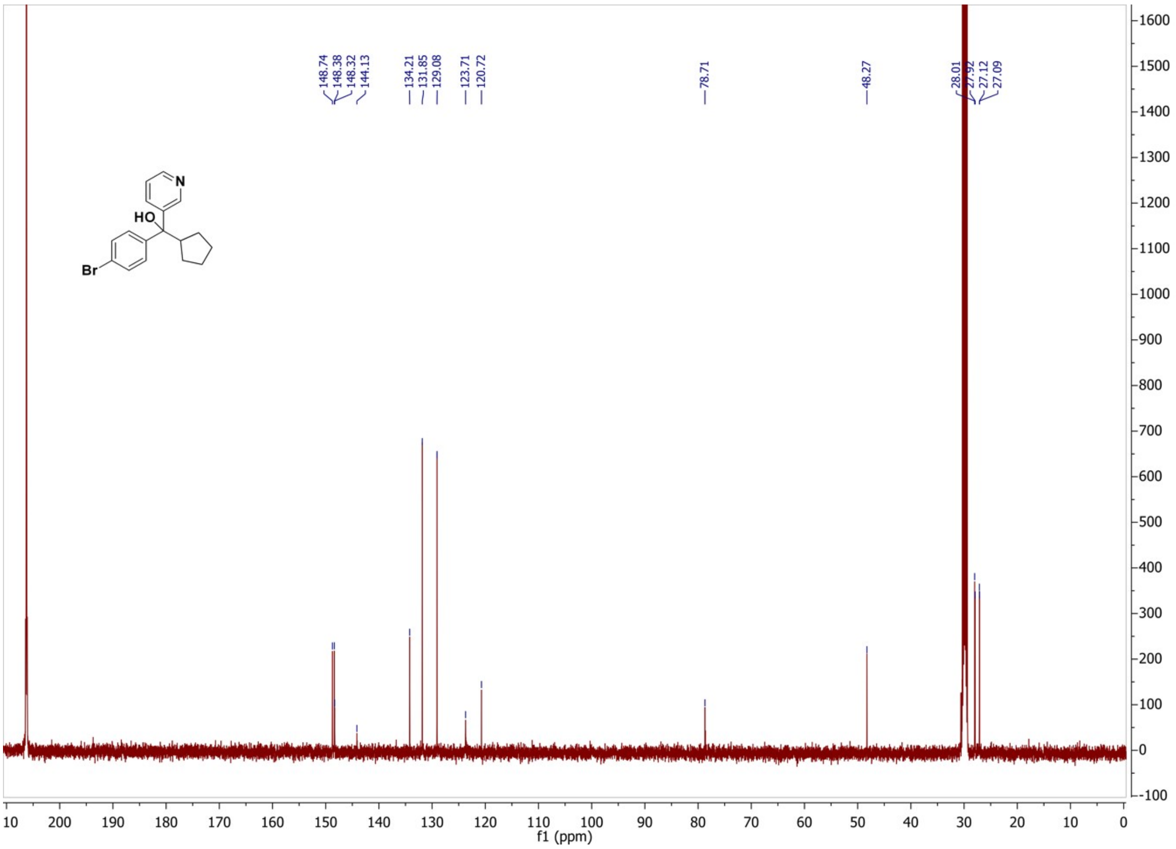


Figure S137. $^{13}$C NMR spectrum of (4-bromophenyl)(cyclopentyl)(pyridin-3-yl)methanol (**MYOS_00451**, 126 MHz, Acetone-$d_6$).

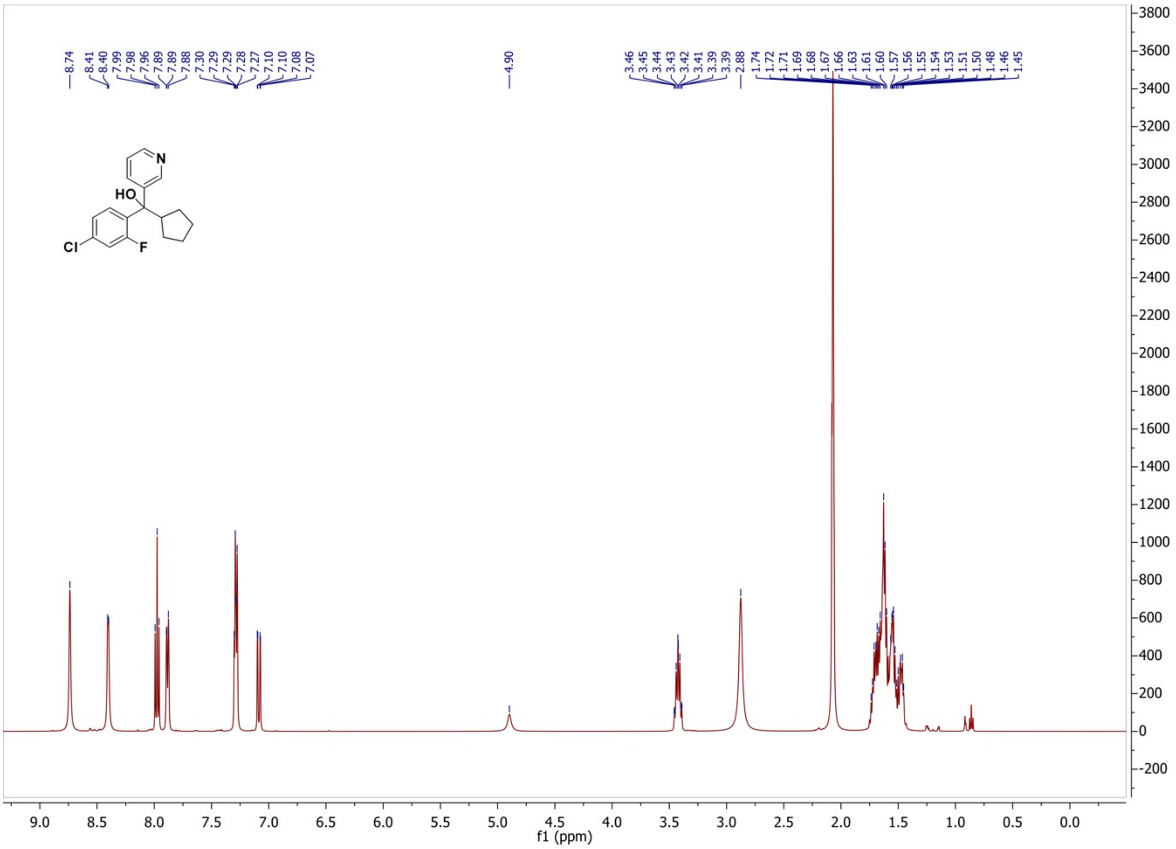

Figure S138. $^{1}H$ NMR spectrum of (4-chloro-2-fluorophenyl)(cyclopentyl)(pyridin-3-yl)methanol (**MYOS_00452**, 500 MHz, Acetone-$d_6$).

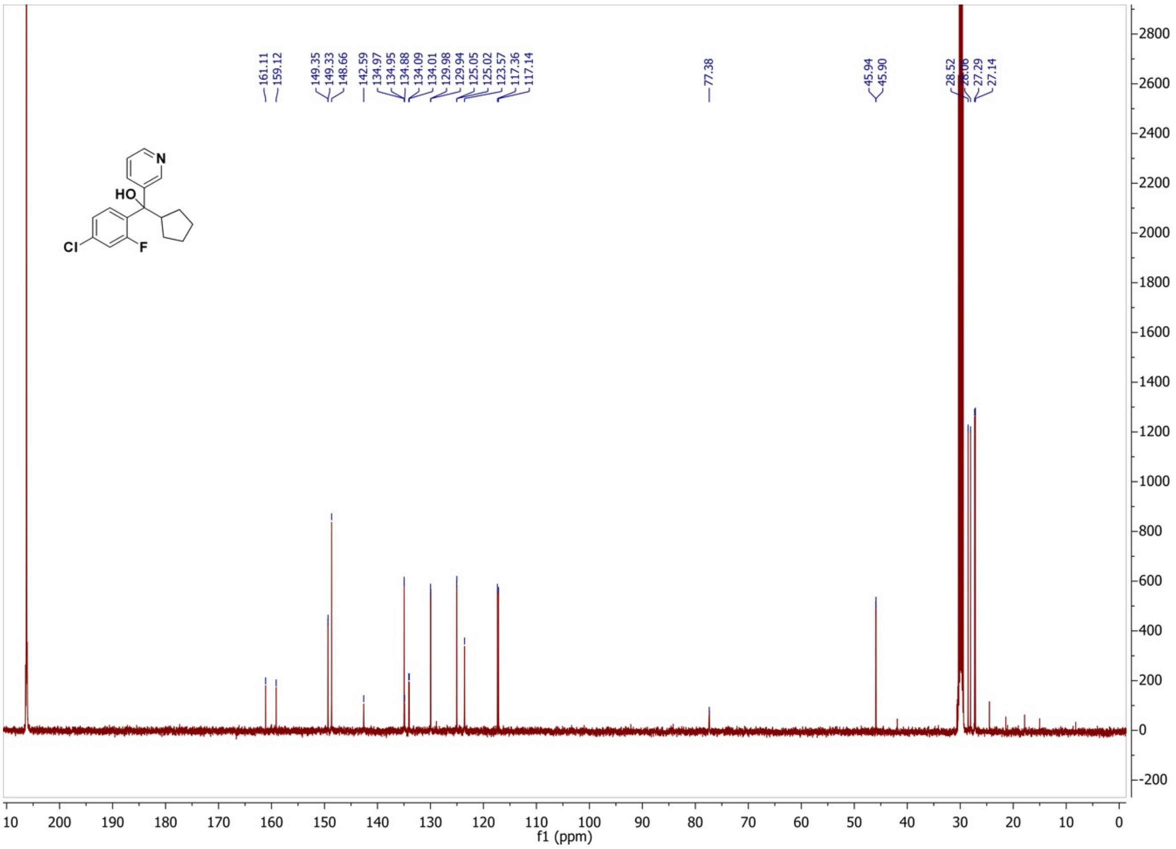

Figure S139. $^{13}C$ NMR spectrum of (4-chloro-2-fluorophenyl)(cyclopentyl)(pyridin-3-yl)methanol (**MYOS_00452**, 126 MHz, Acetone-$d_6$).

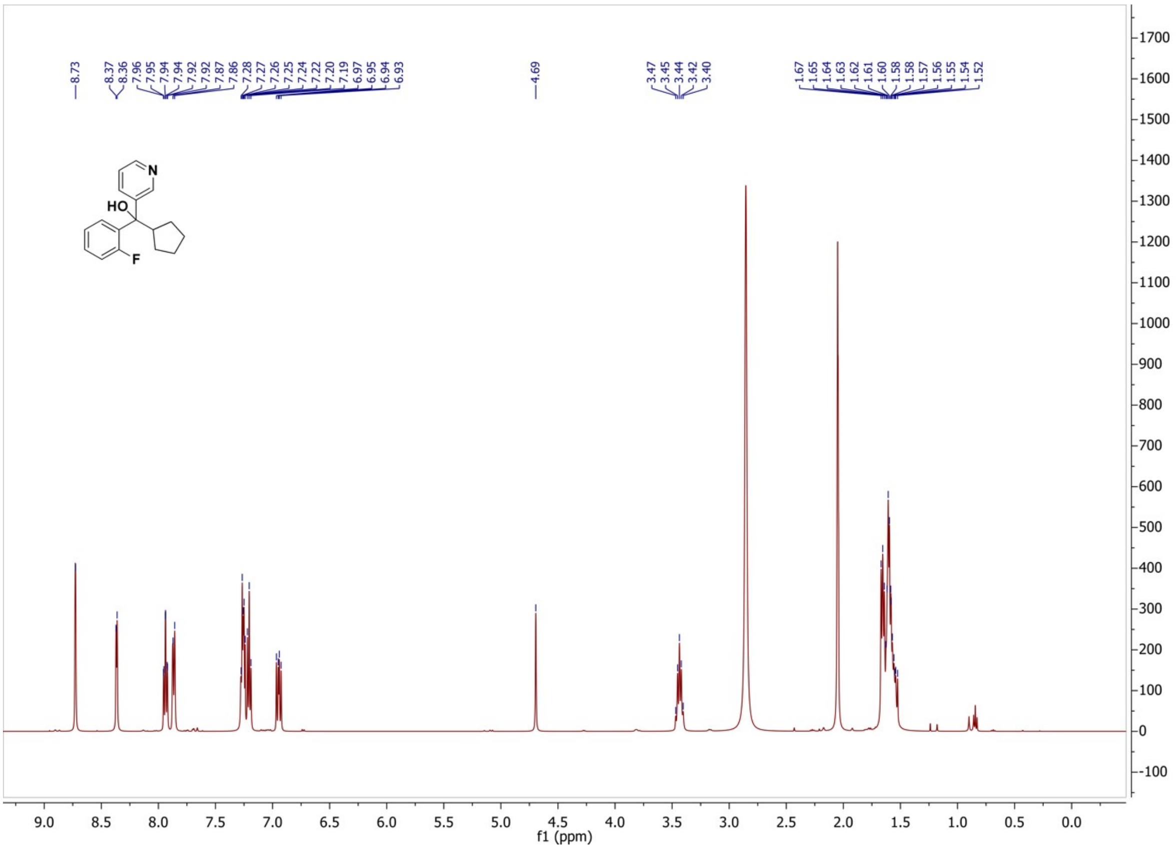


Figure S140. $^{1}H$ NMR cyclopentyl(2-fluorophenyl)(pyridin-3-yl)methanol (**MYOS_00453**, 500 MHz, Acetone-$d_6$).

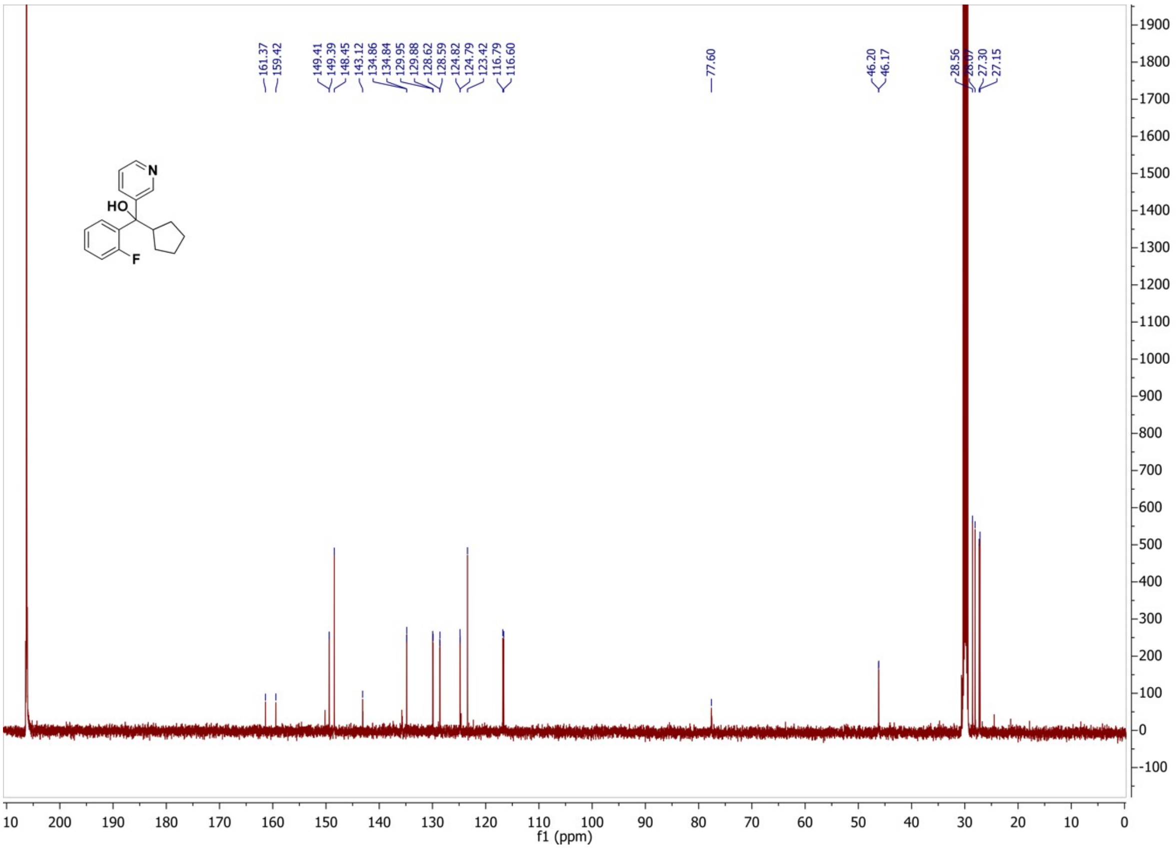


Figure S141. $^{13}C$ NMR spectrum of cyclopentyl(2-fluorophenyl)(pyridin-3-yl)methanol (**MYOS_00453**, 126 MHz, Acetone-$d_6$).

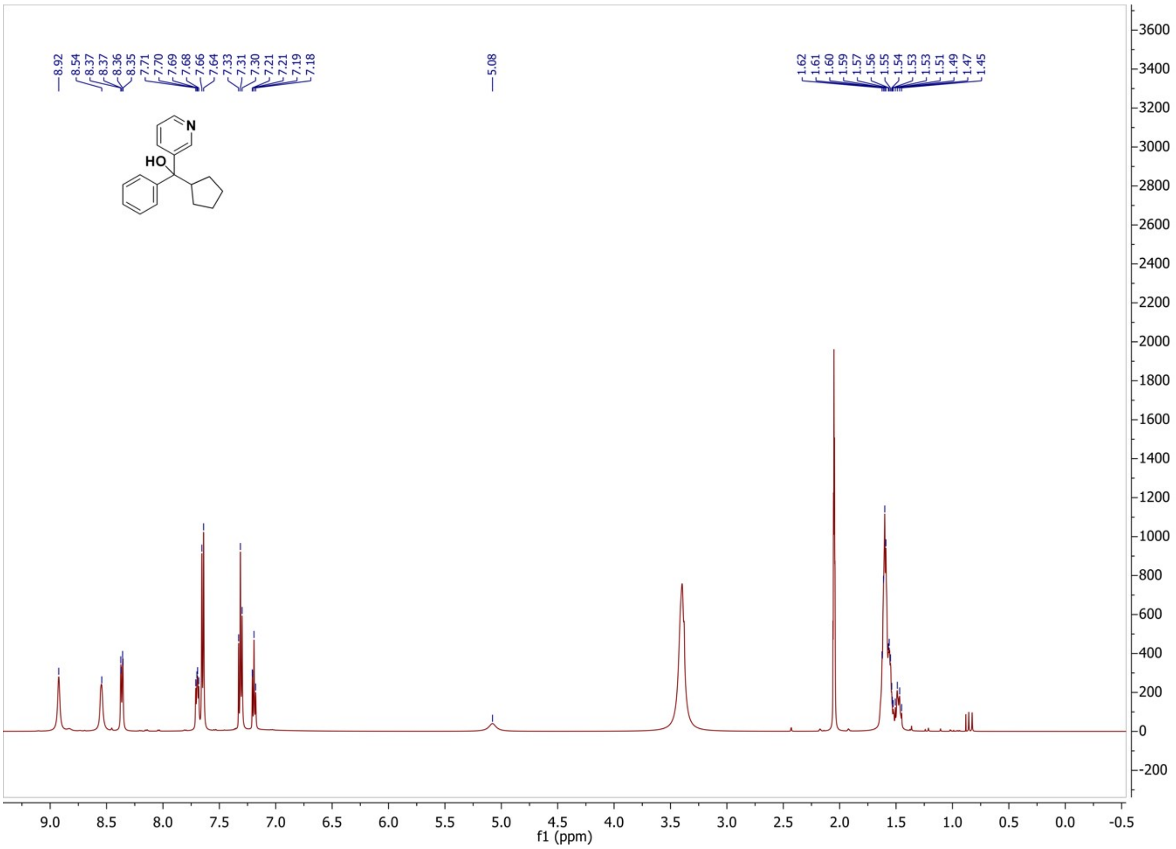

Figure S142. $^{1}H$ NMR spectrum of cyclopentyl(phenyl)(pyridin-3-yl)methanol (**MYOS_00454**, 500 MHz, Acetone-$d_6$).

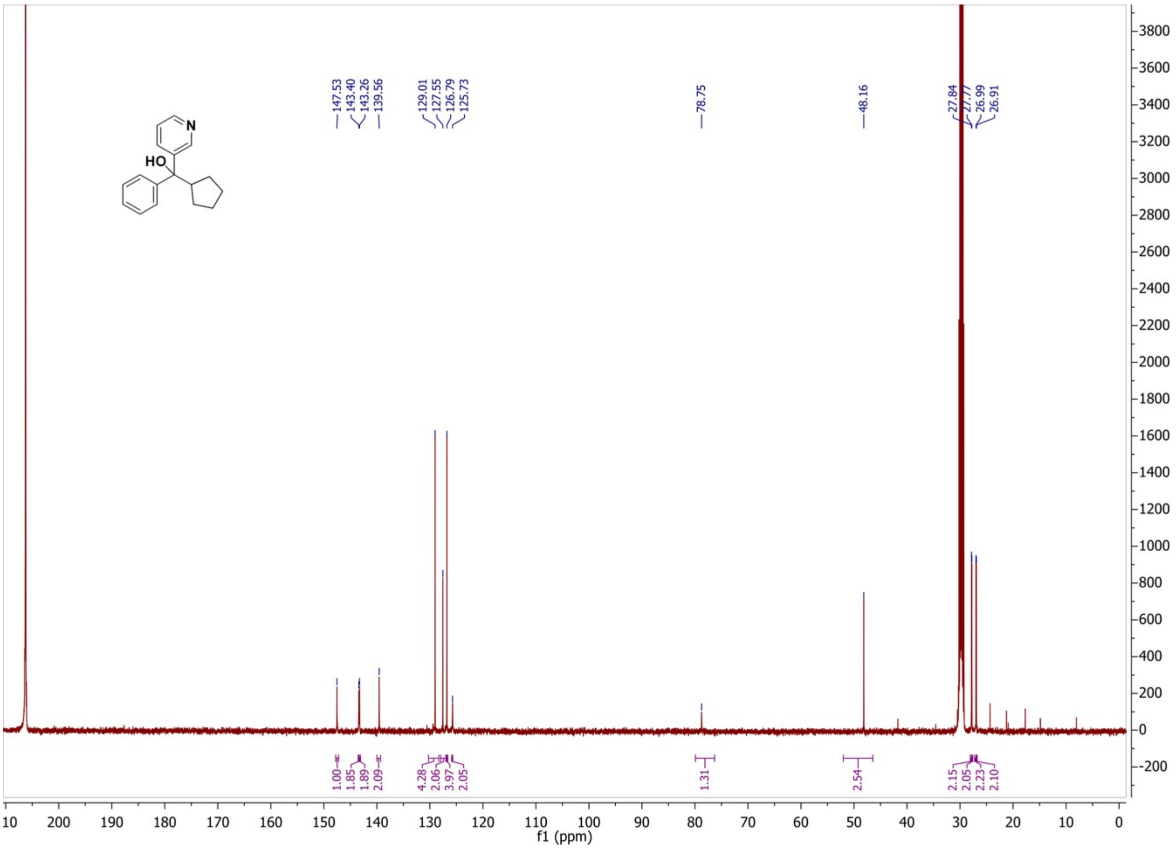

Figure S143. $^{13}C$ NMR spectrum of cyclopentyl(phenyl)(pyridin-3-yl)methanol (**MYOS_00454**, 126 MHz, Acetone-$d_6$).

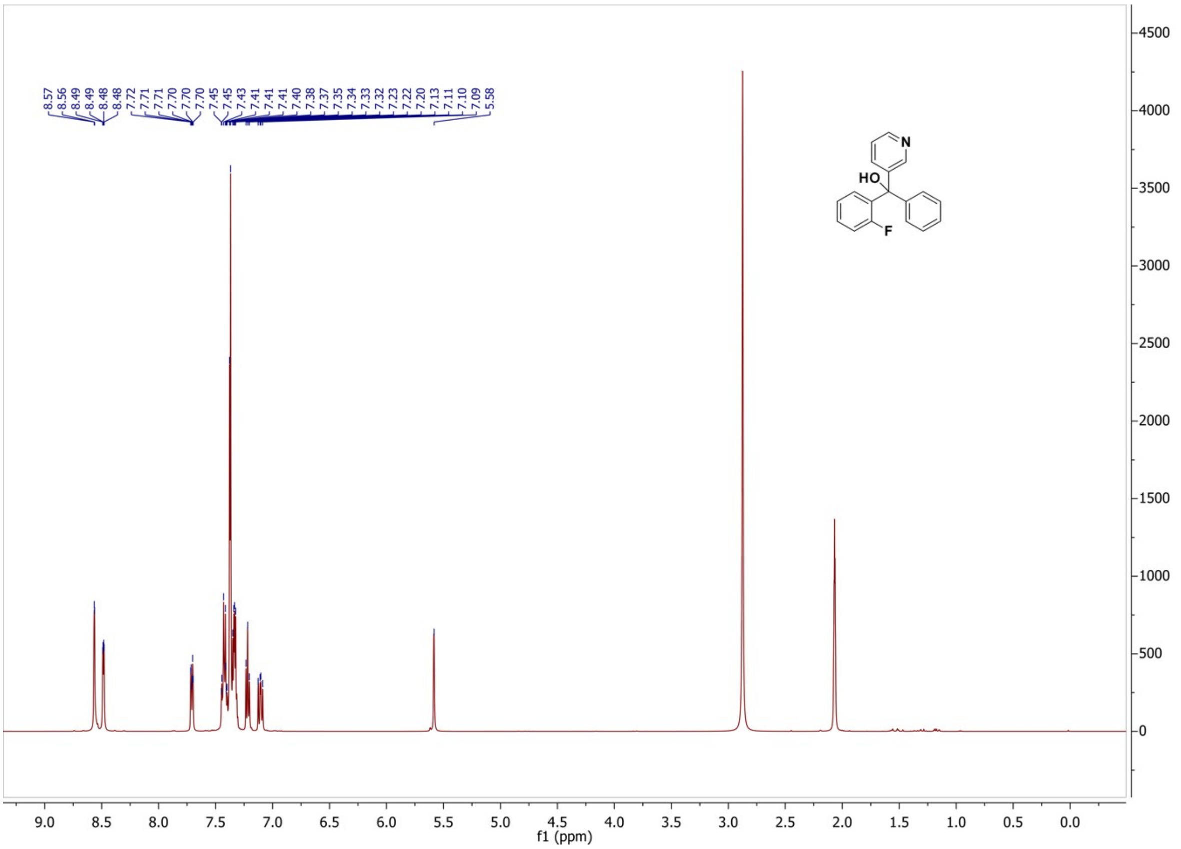

Figure S144. $^{1}$H NMR spectrum of (2-fluorophenyl)(phenyl)(pyridin-3-yl)methanol (**MYOS_00455**, 500 MHz, Acetone-$d_6$).

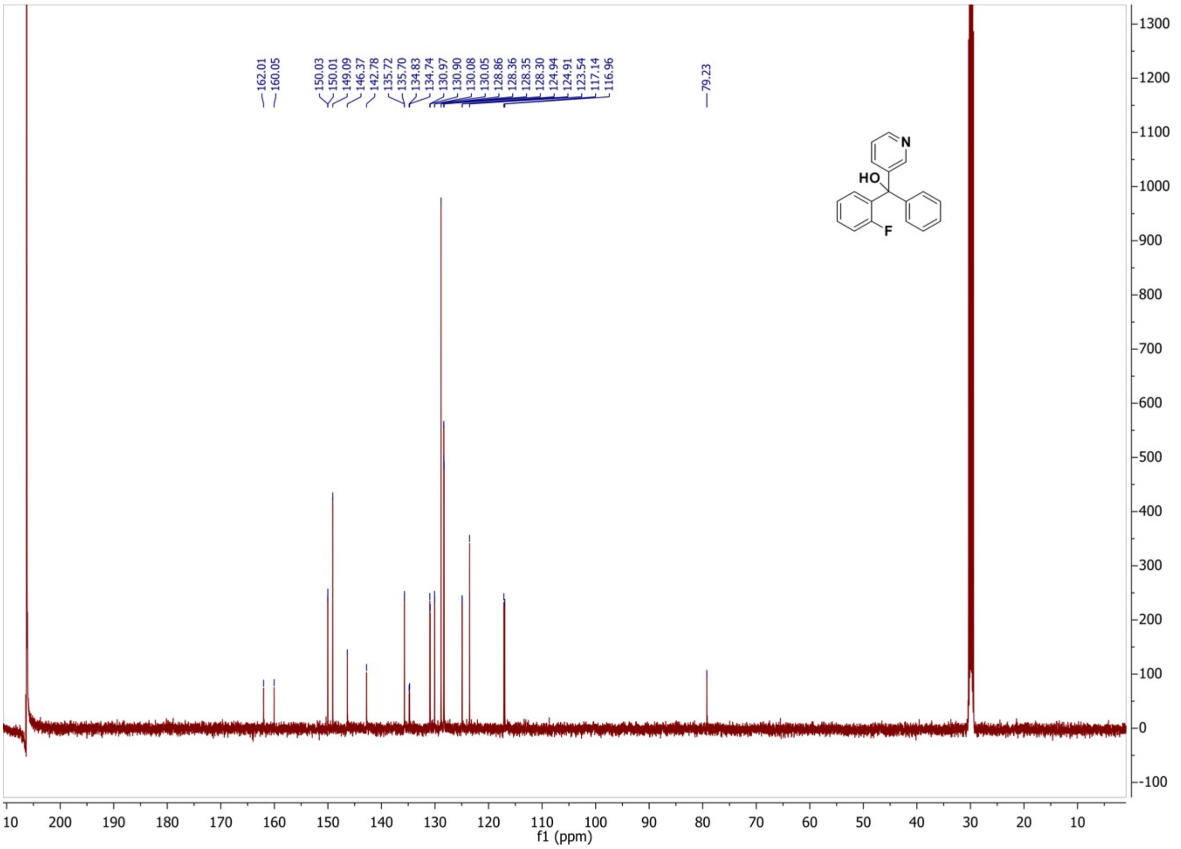

Figure S145. $^{13}$C NMR spectrum of (2-fluorophenyl)(phenyl)(pyridin-3-yl)methanol (**MYOS_00455**, 126 MHz, Acetone-$d_6$).

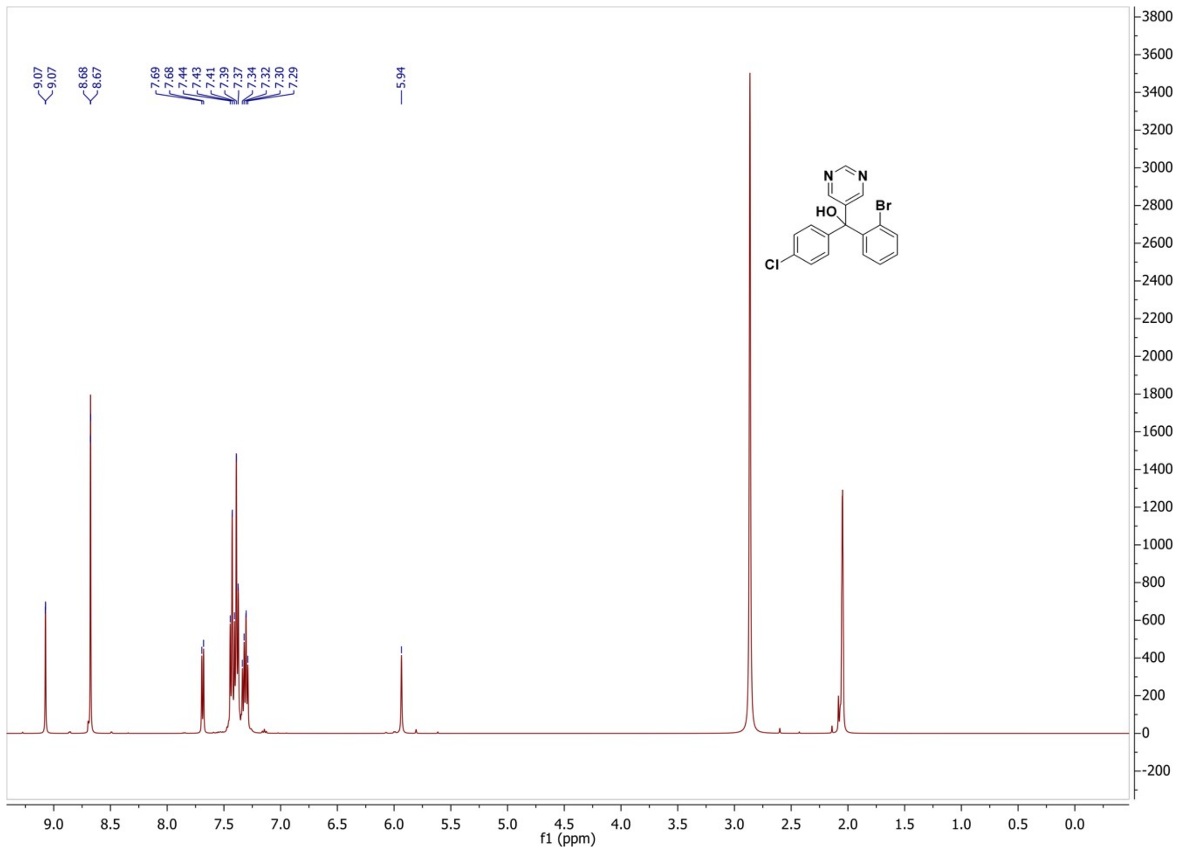

Figure S146. $^1$H NMR spectrum of (2-bromophenyl)(4-chlorophenyl)(pyrimidin-5-yl)methanol (**MYOS_00510**, 500 MHz, Acetone-$d_6$).

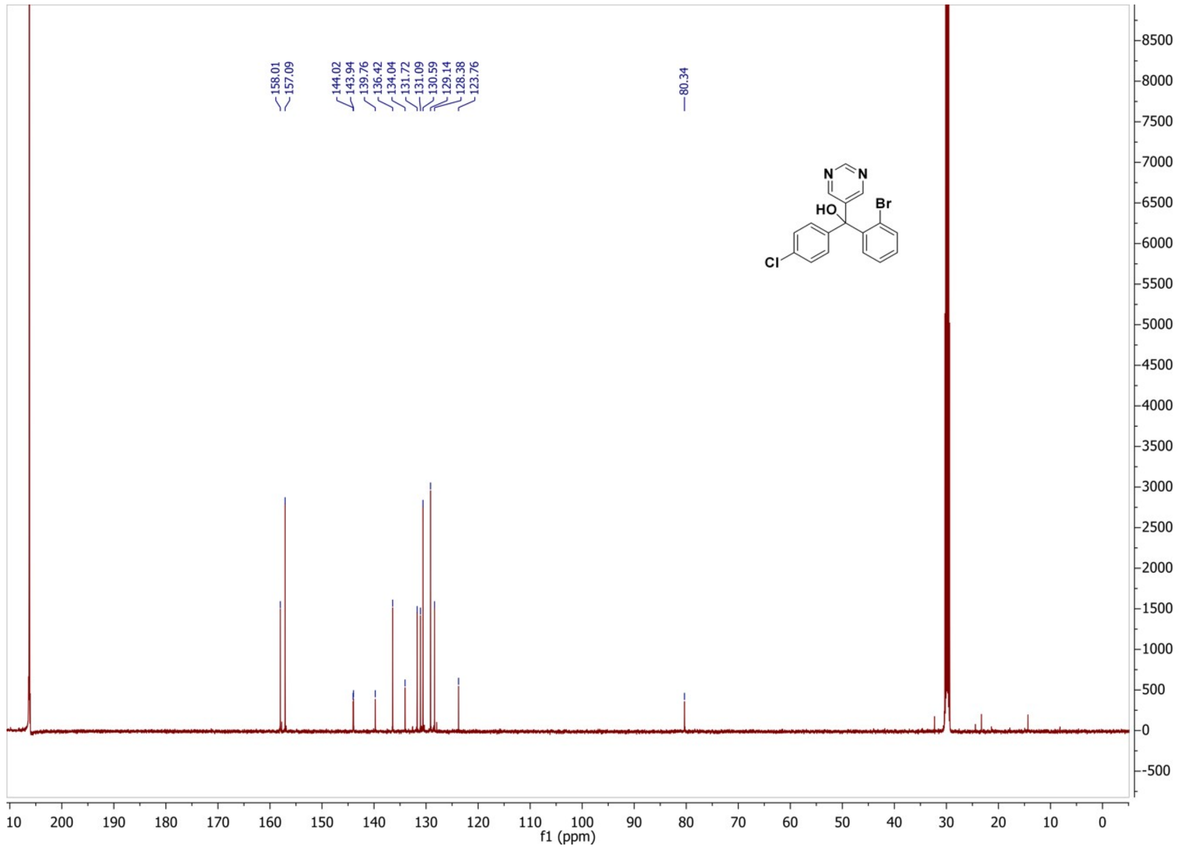

Figure S147. $^{13}$C NMR spectrum of (2-bromophenyl)(4-chlorophenyl)(pyrimidin-5-yl)methanol (**MYOS_00510**, 126 MHz, Acetone-$d_6$).

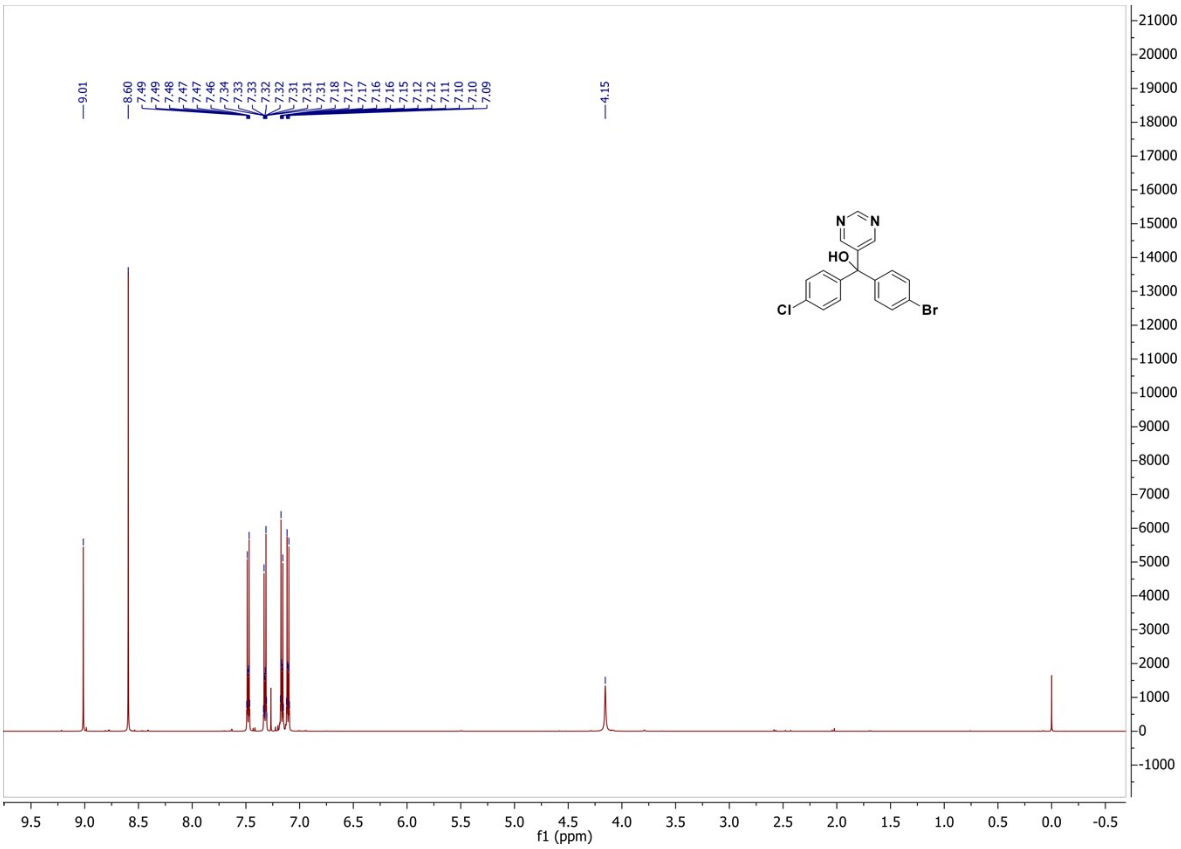

Figure S148. $^{1}$H NMR spectrum of (4-bromophenyl)(4-chlorophenyl)(pyrimidin-5-yl)methanol (**MYOS_00511**, 500 MHz, $CDCl_3$).

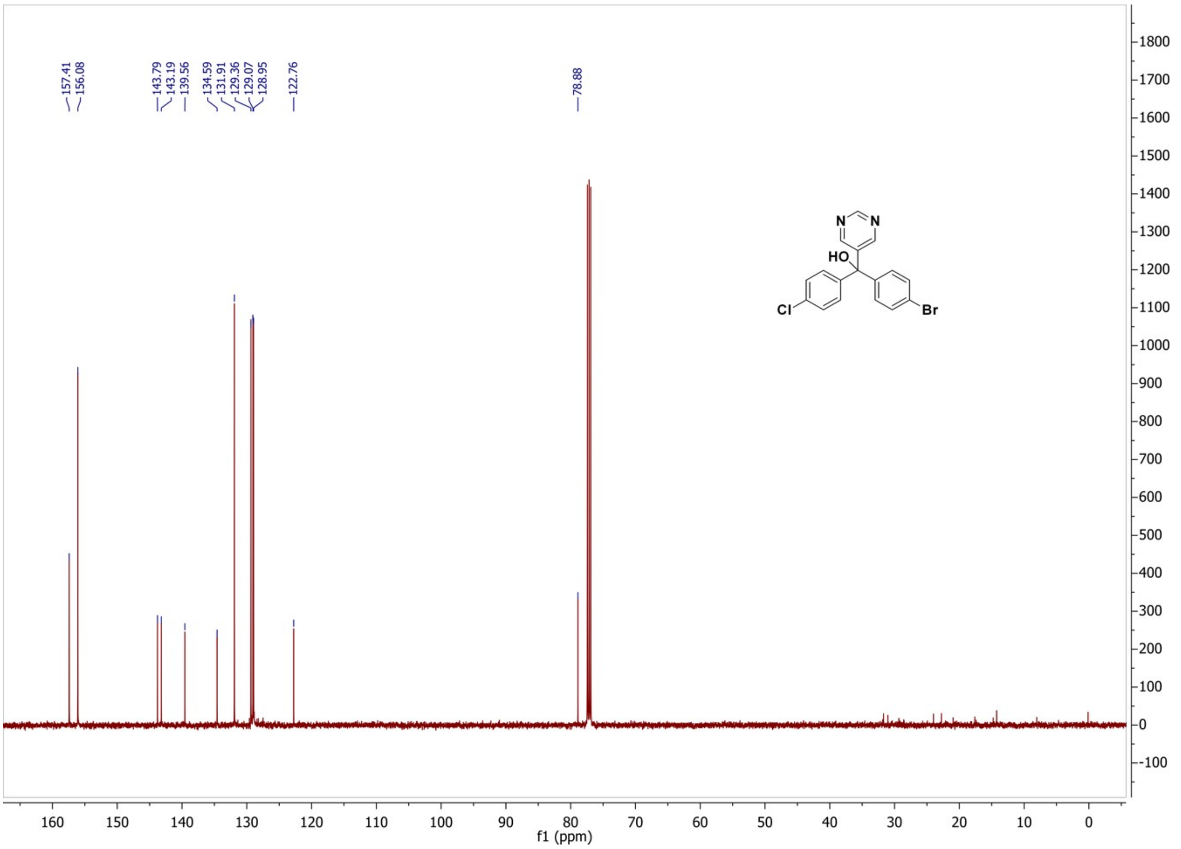

Figure S149. $^{13}$C NMR spectrum of (4-bromophenyl)(4-chlorophenyl)(pyrimidin-5-yl)methanol (**MYOS_00511**, 126 MHz, $CDCl_3$).

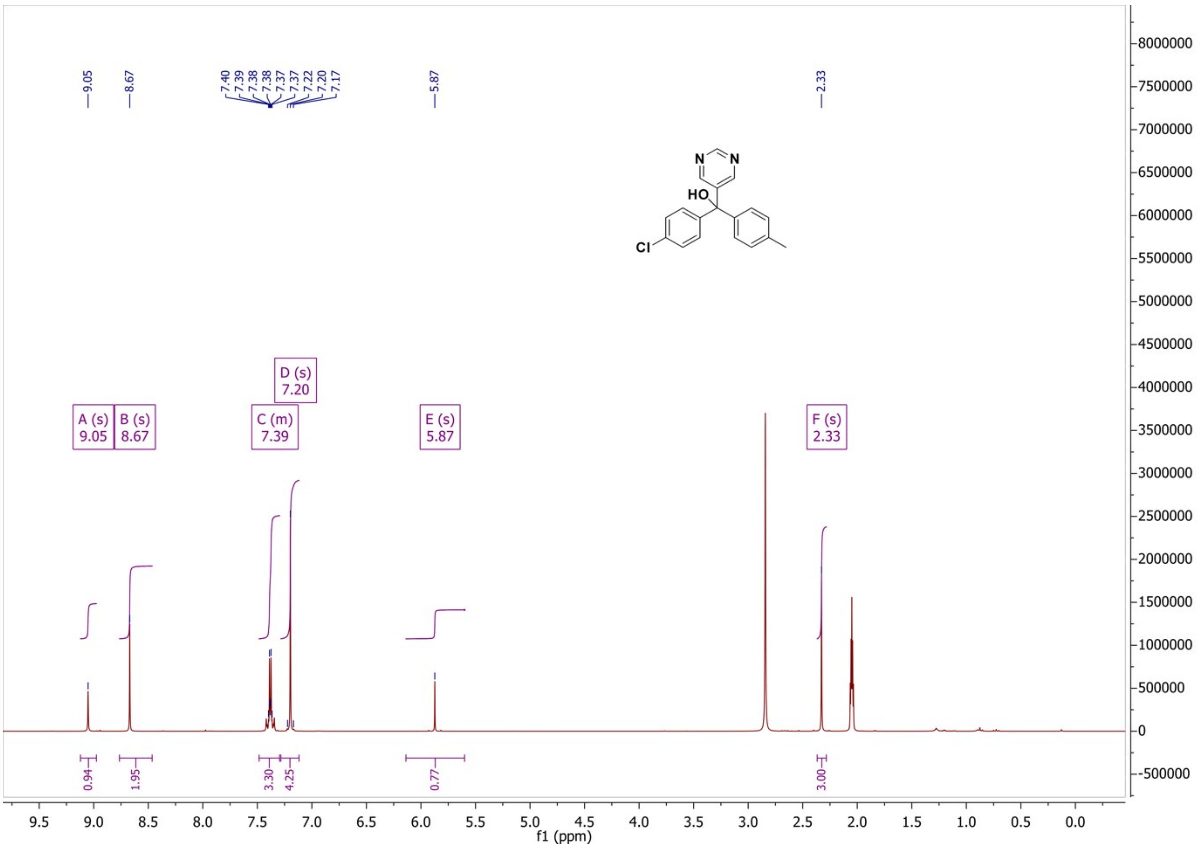

Figure S150. $^{1}H$ NMR spectrum of (4-chlorophenyl)(pyrimidin-5-yl)(*p*-tolyl)methanol (**MYOS_00512**, 500 MHz, Acetone-$d_6$).

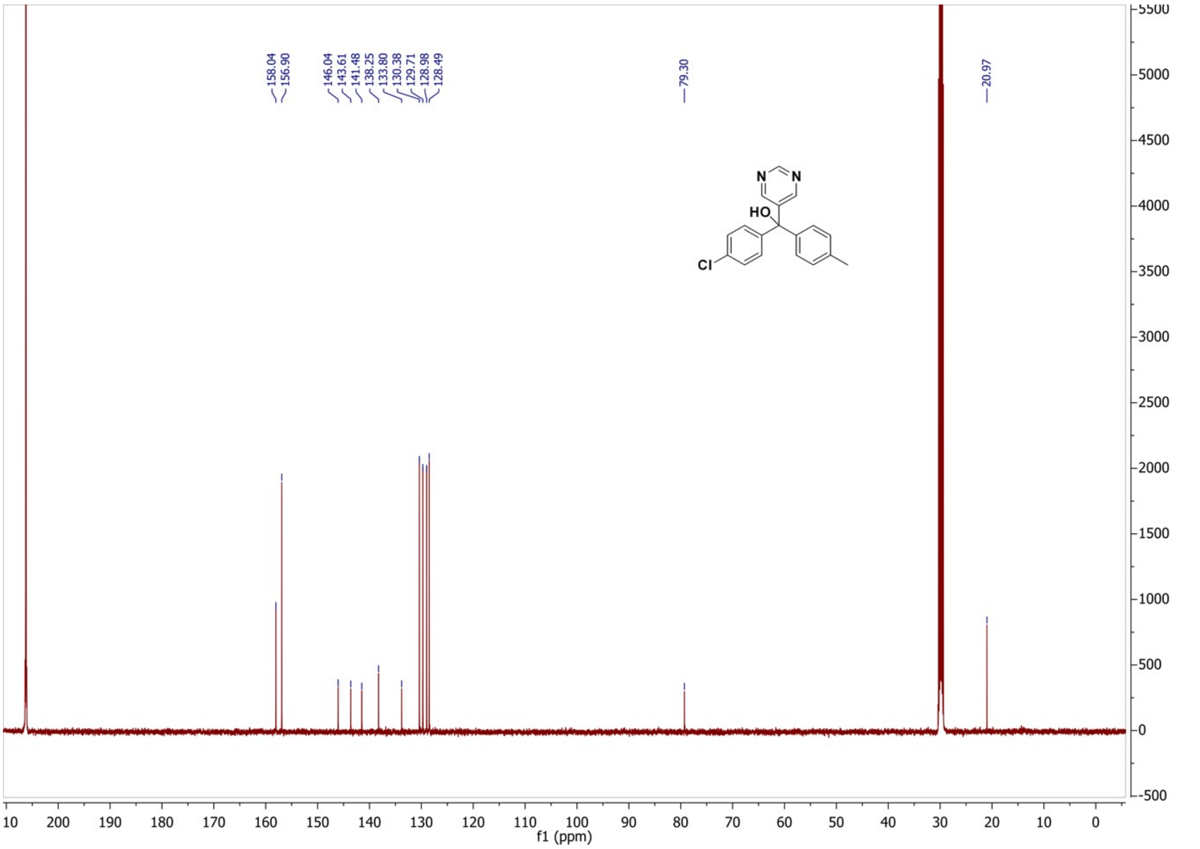

Figure S151. $^{13}C$ NMR spectrum of (4-chlorophenyl)(pyrimidin-5-yl)(*p*-tolyl)methanol (**MYOS_00512**, 126 MHz, Acetone-$d_6$).

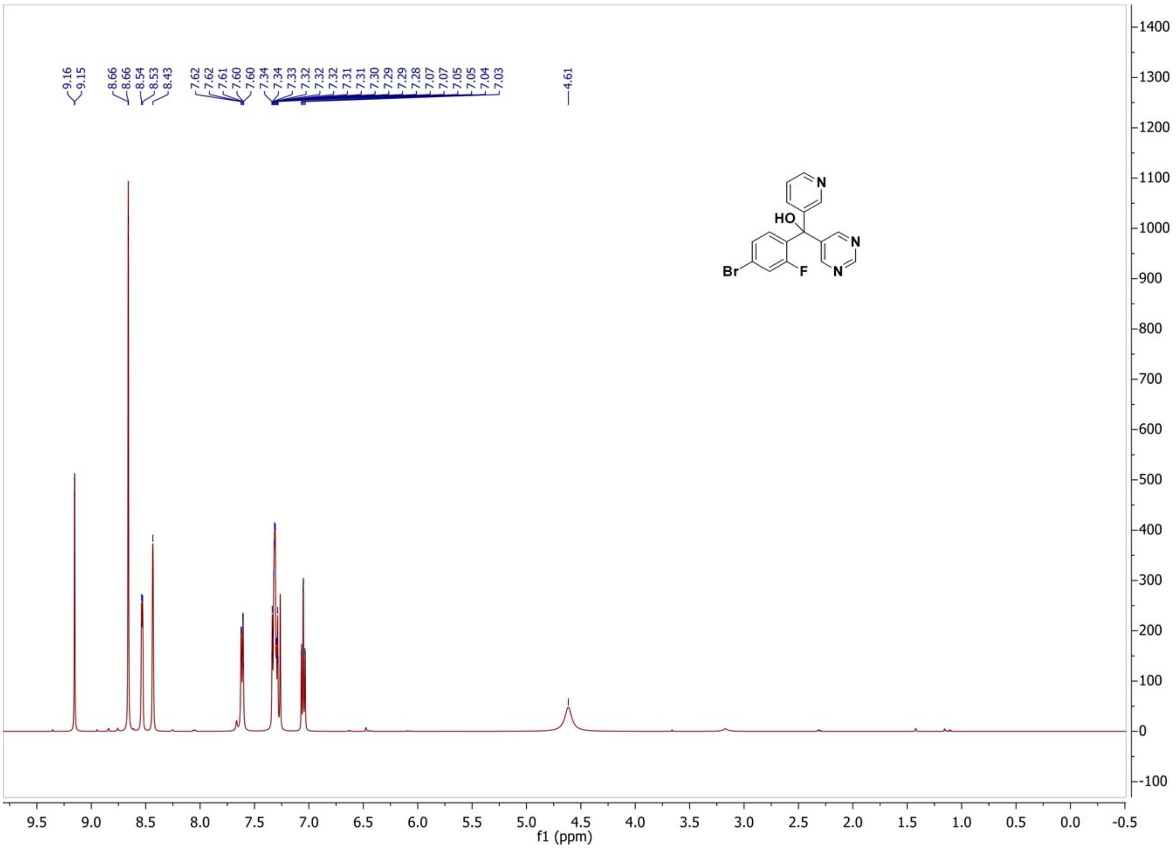


Figure S152. $^{1}H$ NMR spectrum of (4-bromo-2-fluorophenyl)(pyridin-3-yl)(pyrimidin-5-yl)methanol (**MYOS_00513**, 500 MHz, $CDCl_3$).

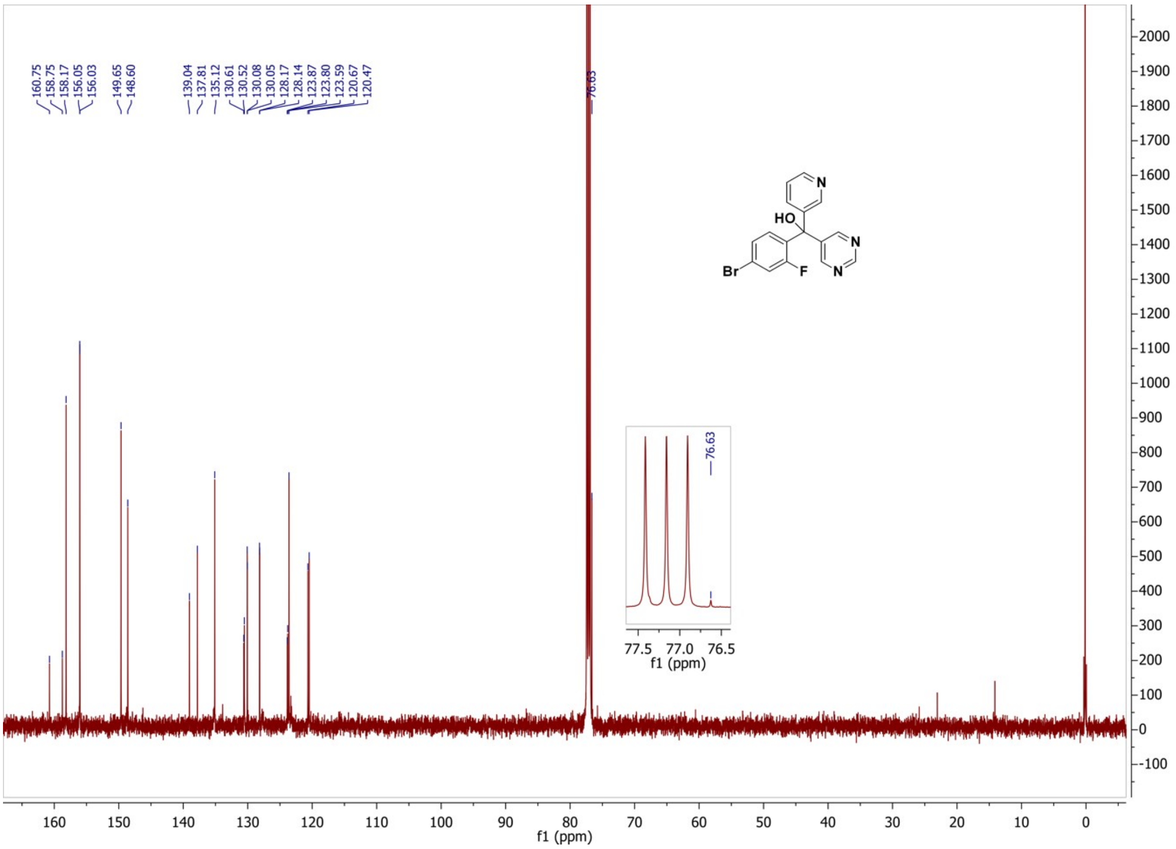


Figure S153. $^{13}C$ NMR spectrum of (4-bromo-2-fluorophenyl)(pyridin-3-yl)(pyrimidin-5-yl)methanol (**MYOS_00513**, 126 MHz, $CDCl_3$).

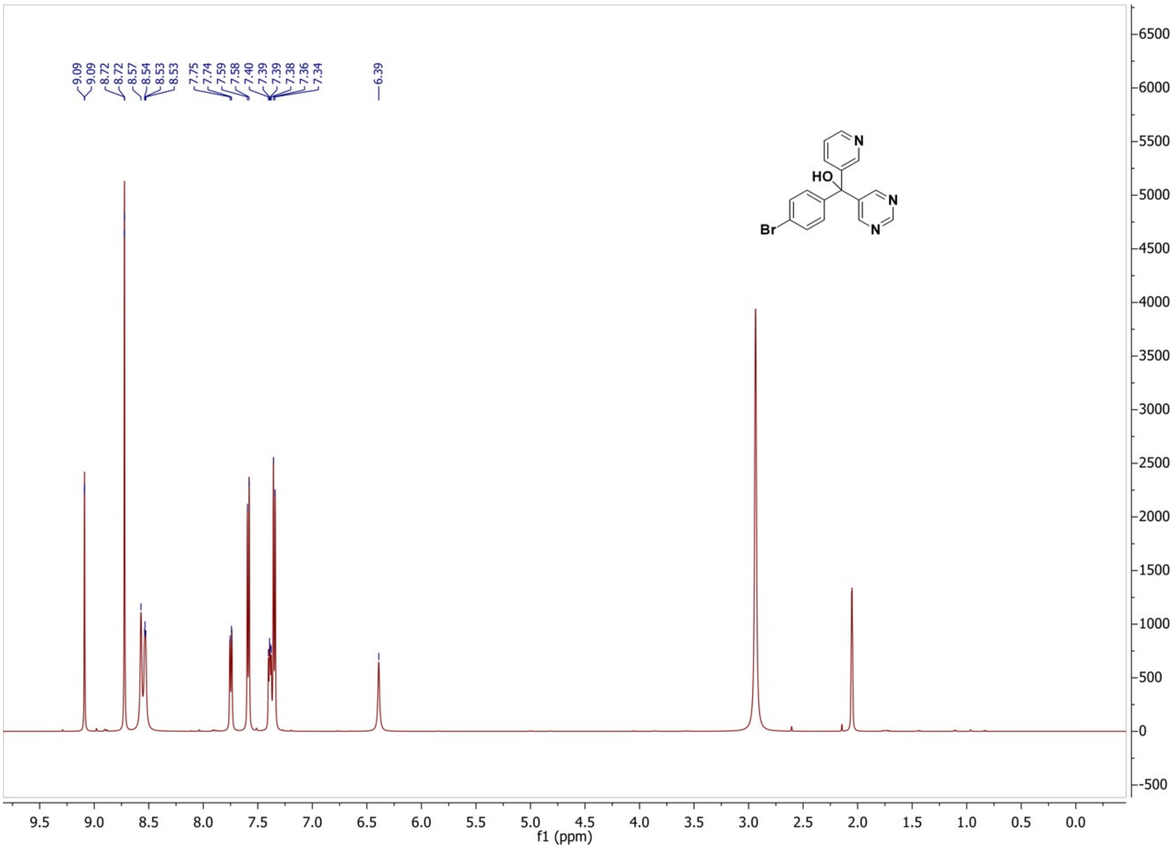


Figure S154. ¹H NMR spectrum of (4-bromophenyl)(pyridin-3-yl)(pyrimidin-5-yl)methanol (**MYOS_00514**, 500 MHz, Acetone-$d_6$).

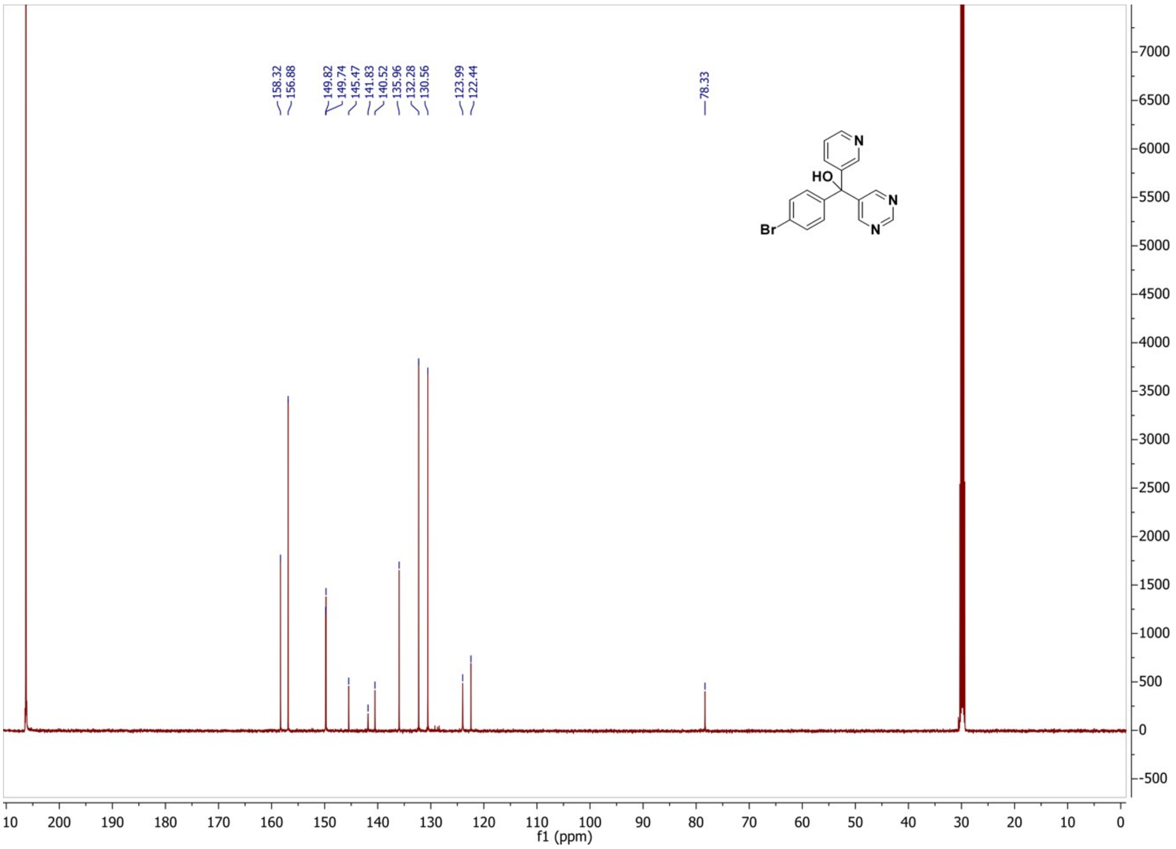


Figure S155. ¹³C NMR spectrum of (4-bromophenyl)(pyridin-3-yl)(pyrimidin-5-yl)methanol (**MYOS_00514**, 126 MHz, Acetone-$d_6$).

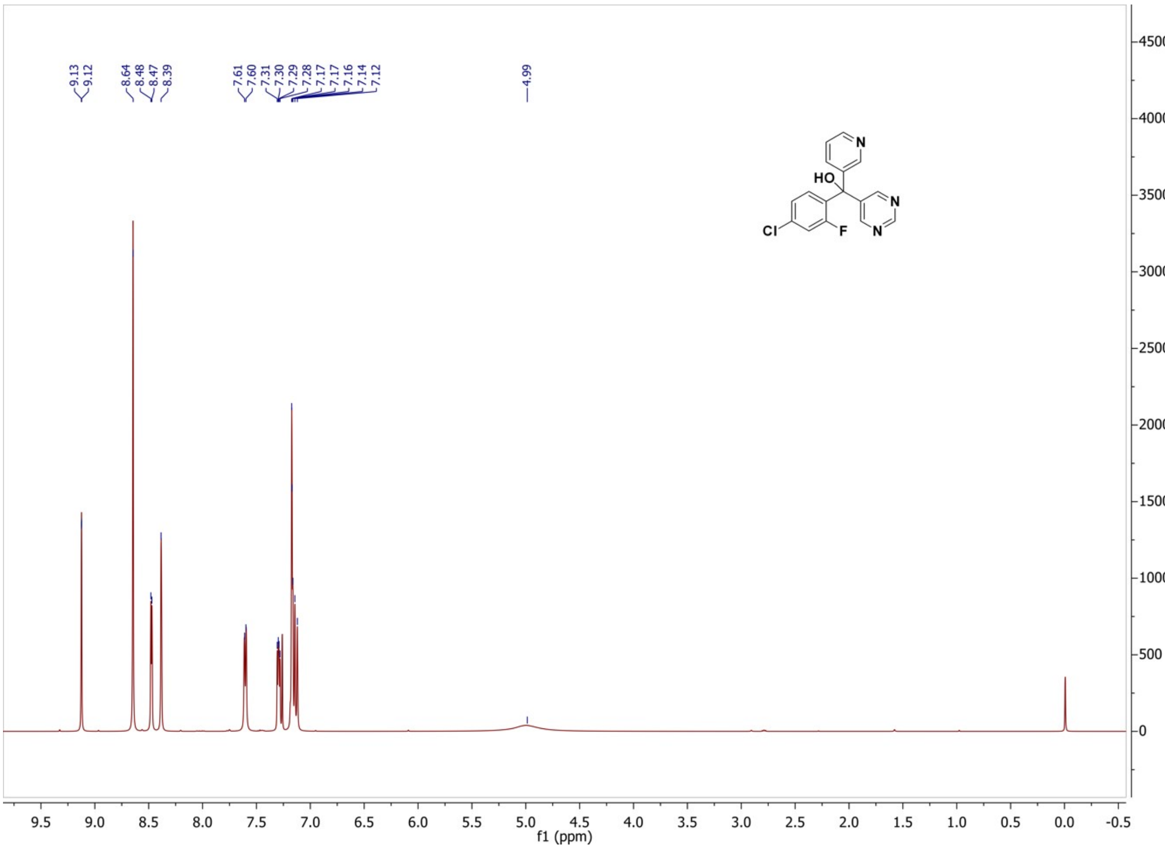

Figure S156. $^{1}H$ NMR (4-chloro-2-fluorophenyl)(pyridin-3-yl)(pyrimidin-5-yl)methanol (**MYOS_00515**, 500 MHz, $CDCl_3$).

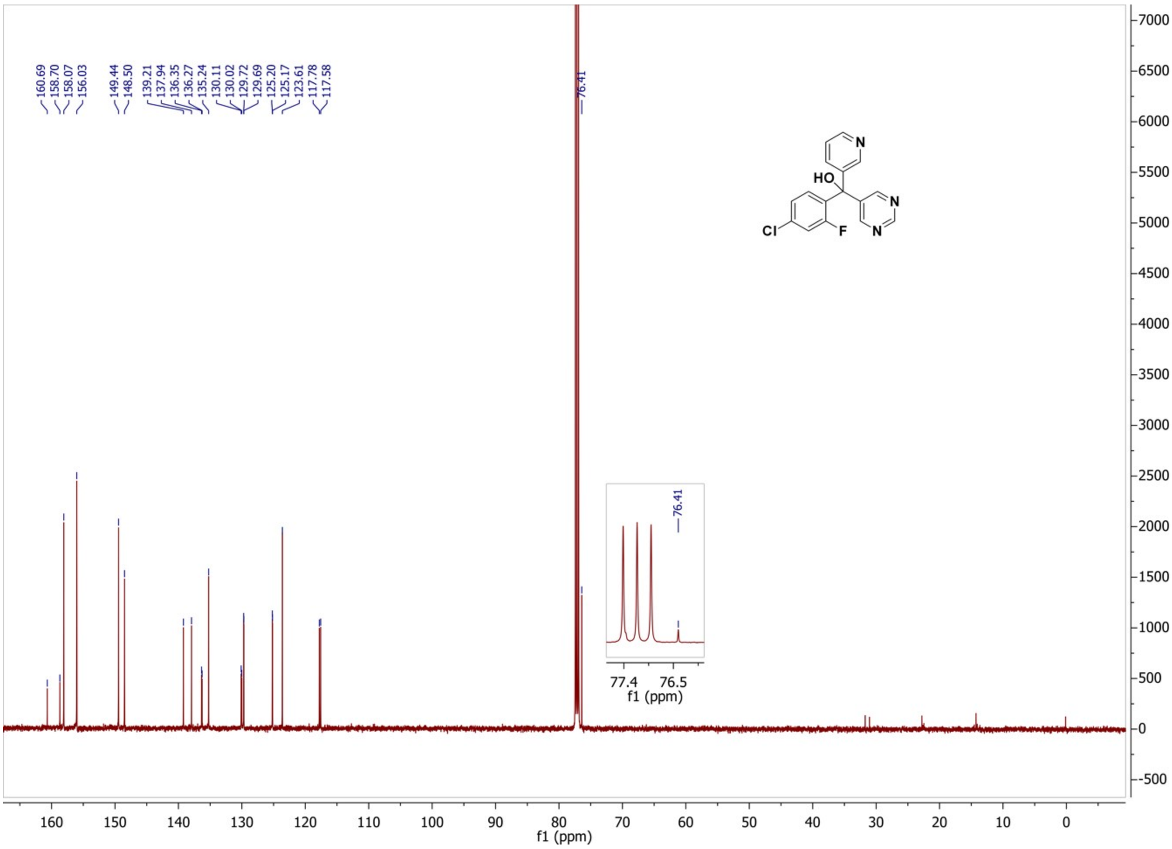

Figure S157. $^{13}C$ NMR spectrum of (4-chloro-2-fluorophenyl)(pyridin-3-yl)(pyrimidin-5-yl)methanol (**MYOS_00515**, 126 MHz, $CDCl_3$).

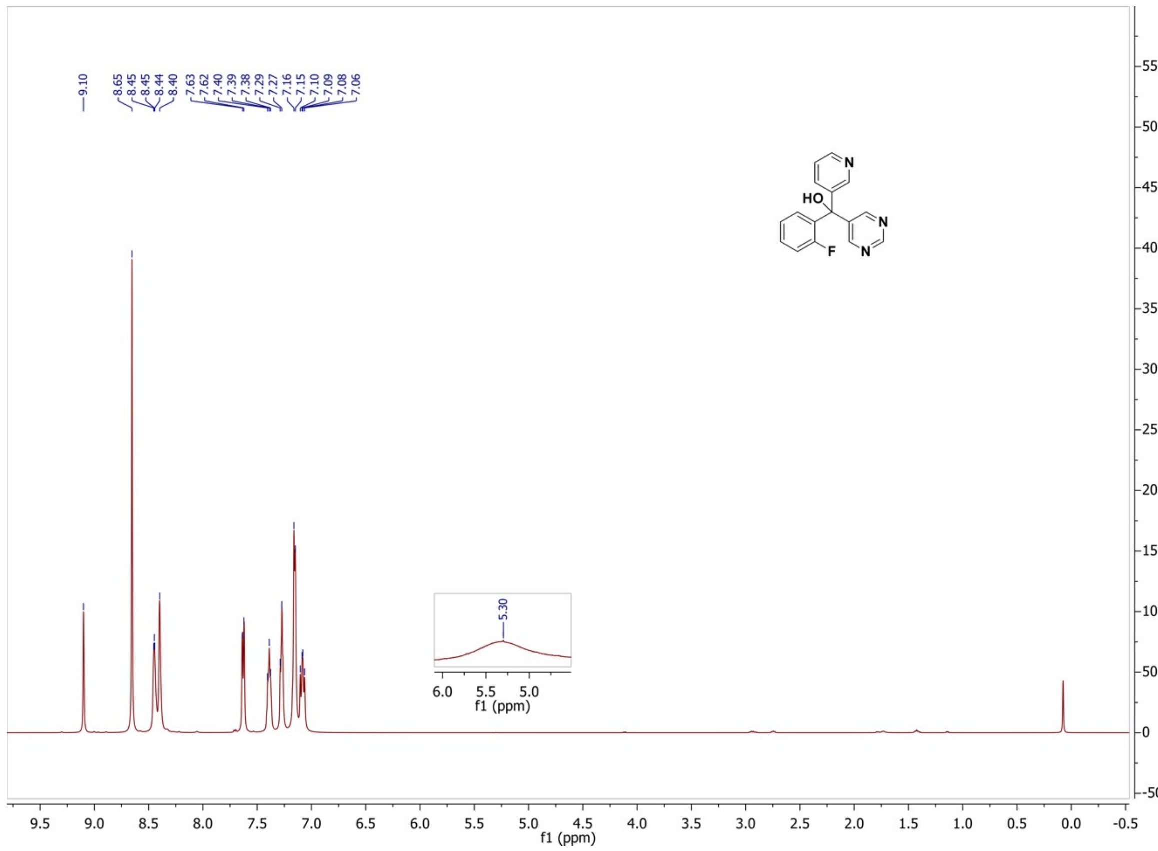


Figure S158. $^{1}$H NMR spectrum of (2-fluorophenyl)(pyridin-3-yl)(pyrimidin-5-yl)methanol (**MYOS_00516**, 500 MHz, $CDCl_3$).

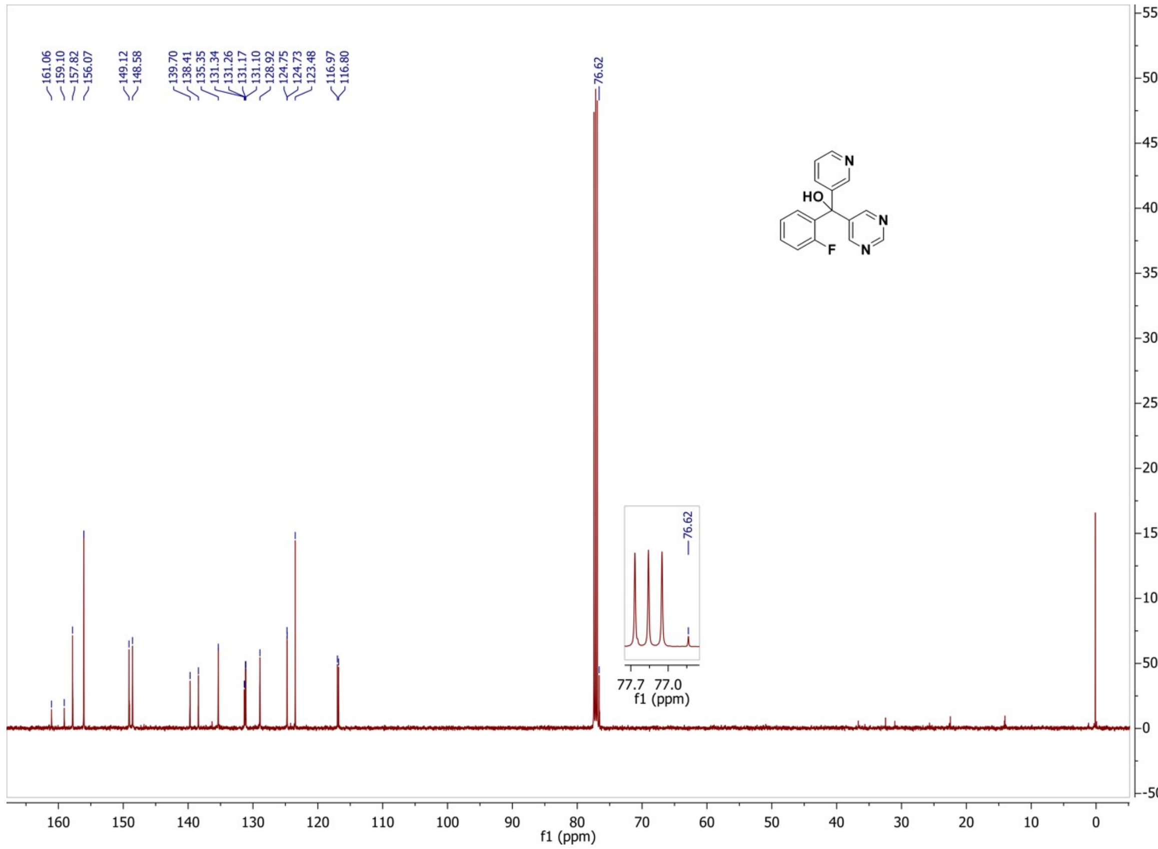


Figure S159. $^{13}$C NMR spectrum of (2-fluorophenyl)(pyridin-3-yl)(pyrimidin-5-yl)methanol (**MYOS_00516**, 126 MHz, $CDCl_3$).

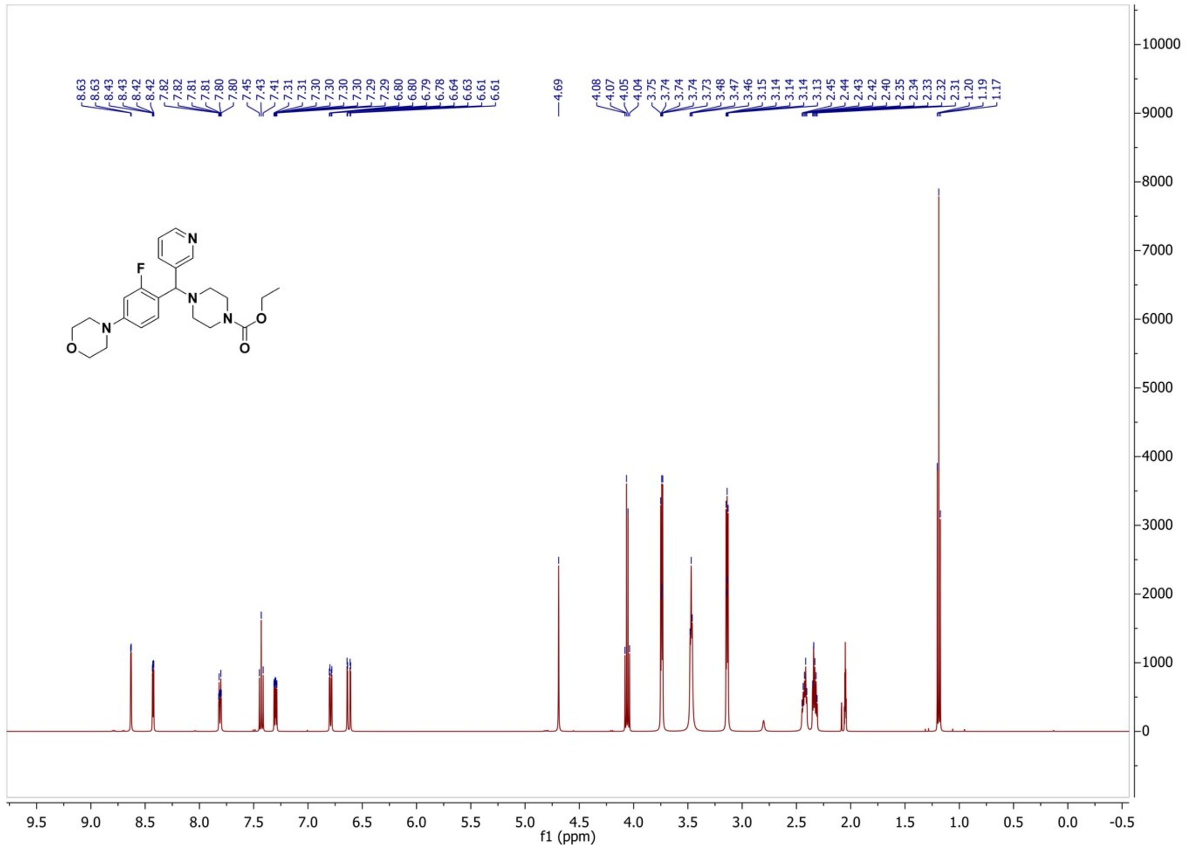

Figure S160. $^{1}H$ NMR spectrum of ethyl 4-((2-fluoro-4-morpholinophenyl)(pyridin-3-yl)methyl)piperazine-1-carboxylate (**MYOS_00517**, 500 MHz, Acetone-$d_6$).

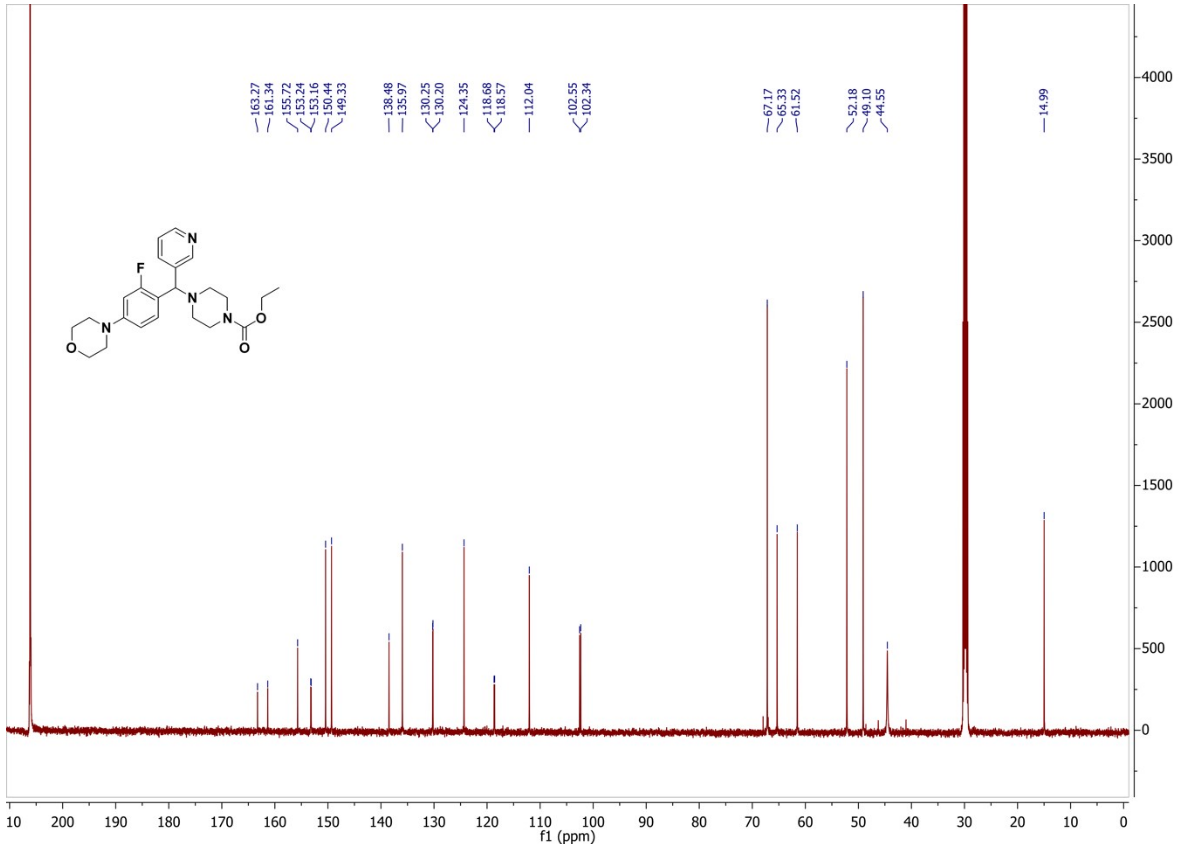

Figure S161. $^{13}C$ NMR spectrum of ethyl 4-((2-fluoro-4-morpholinophenyl)(pyridin-3-yl)methyl)piperazine-1-carboxylate (**MYOS_00517**, 126 MHz, Acetone-$d_6$).

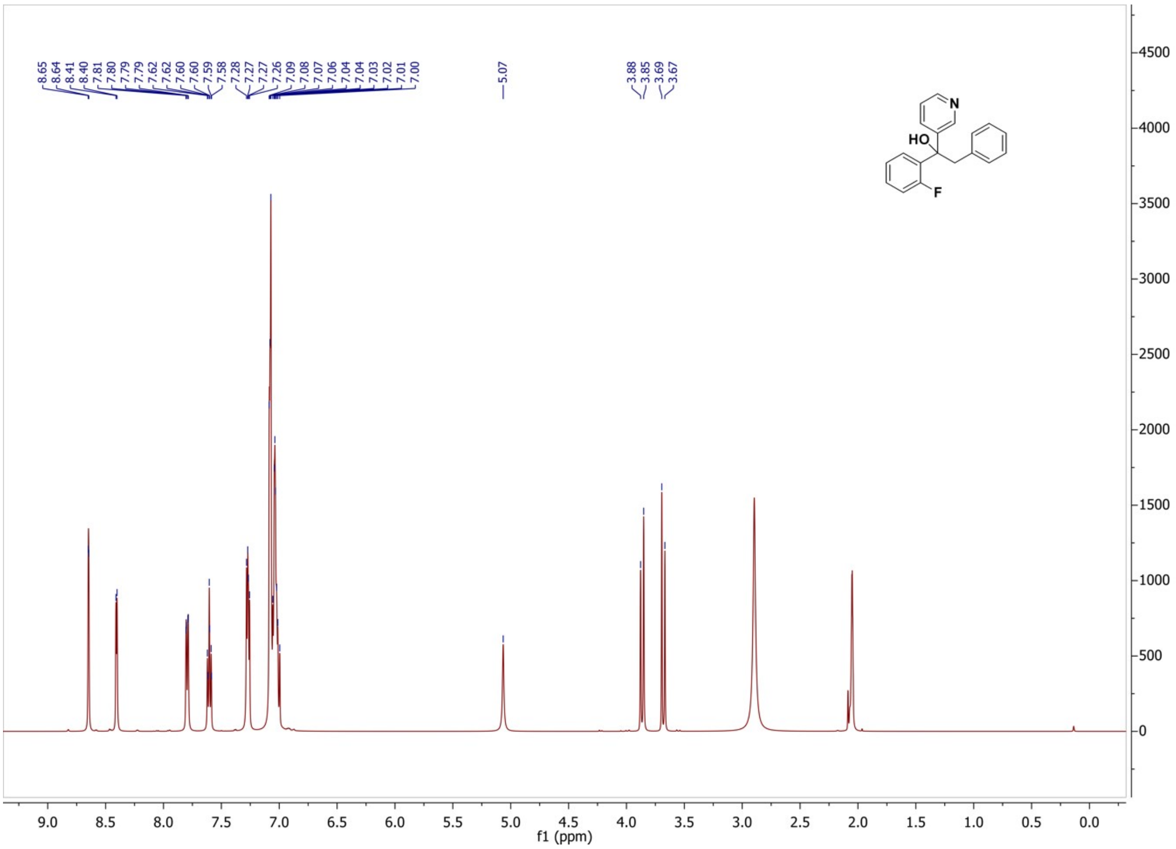

Figure S162. $^{1}H$ NMR spectrum of 1-(2-fluorophenyl)-2-phenyl-1-(pyridin-3-yl)ethan-1-ol (**MYOS_00518**, 500 MHz, Acetone-$d_6$).

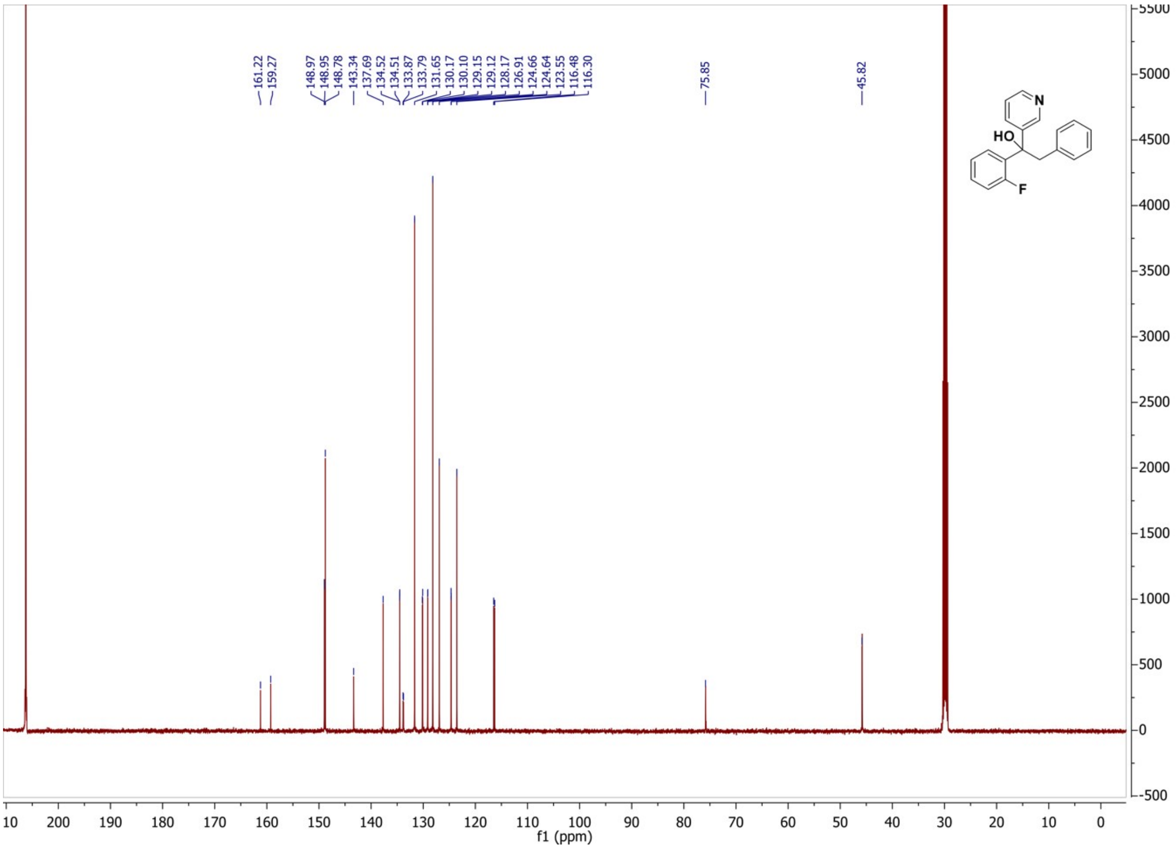

Figure S163. $^{13}C$ NMR spectrum of 1-(2-fluorophenyl)-2-phenyl-1-(pyridin-3-yl)ethan-1-ol (**MYOS_00518**, 126 MHz, Acetone-$d_6$).

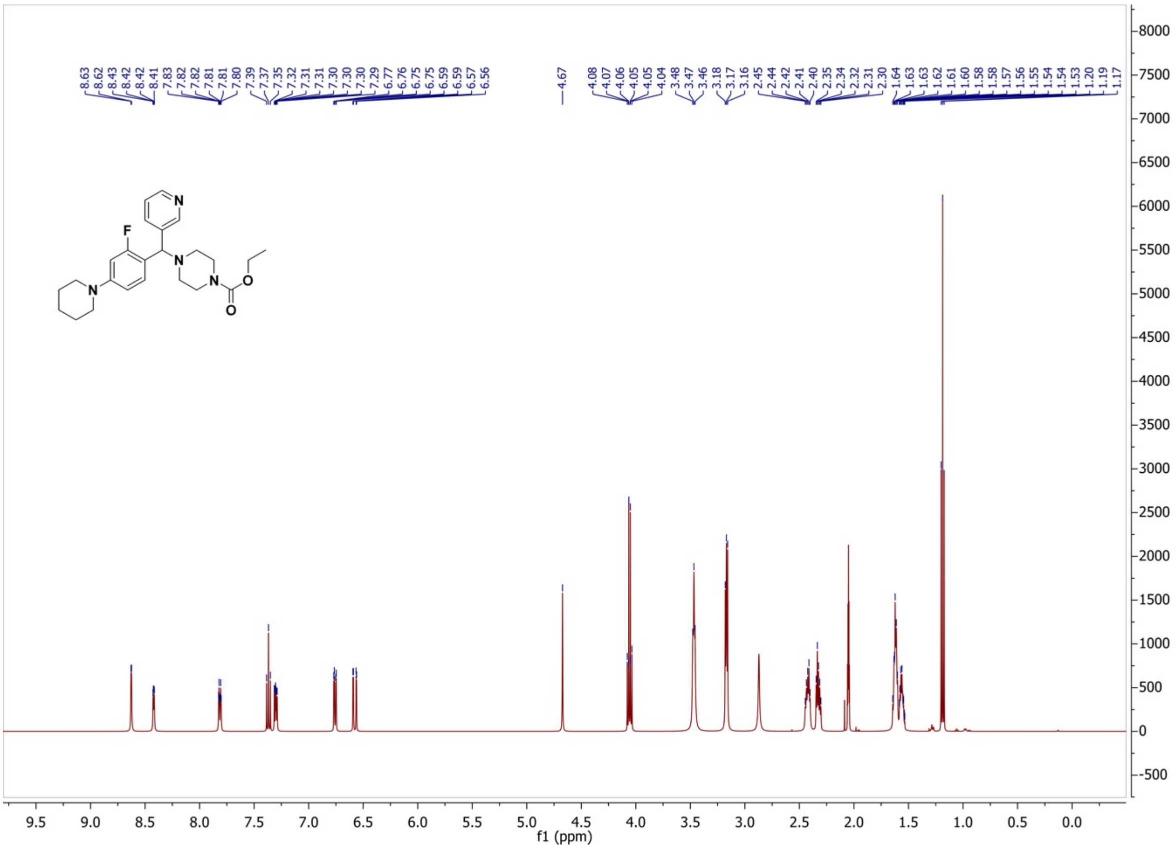

Figure S164. $^{1}H$ NMR spectrum of ethyl 4-((2-fluoro-4-(piperidin-1-yl)phenyl)(pyridin-3-yl)methyl)piperazine-1-carboxylate (**MYOS_00519**, 500 MHz, Acetone-$d_6$).

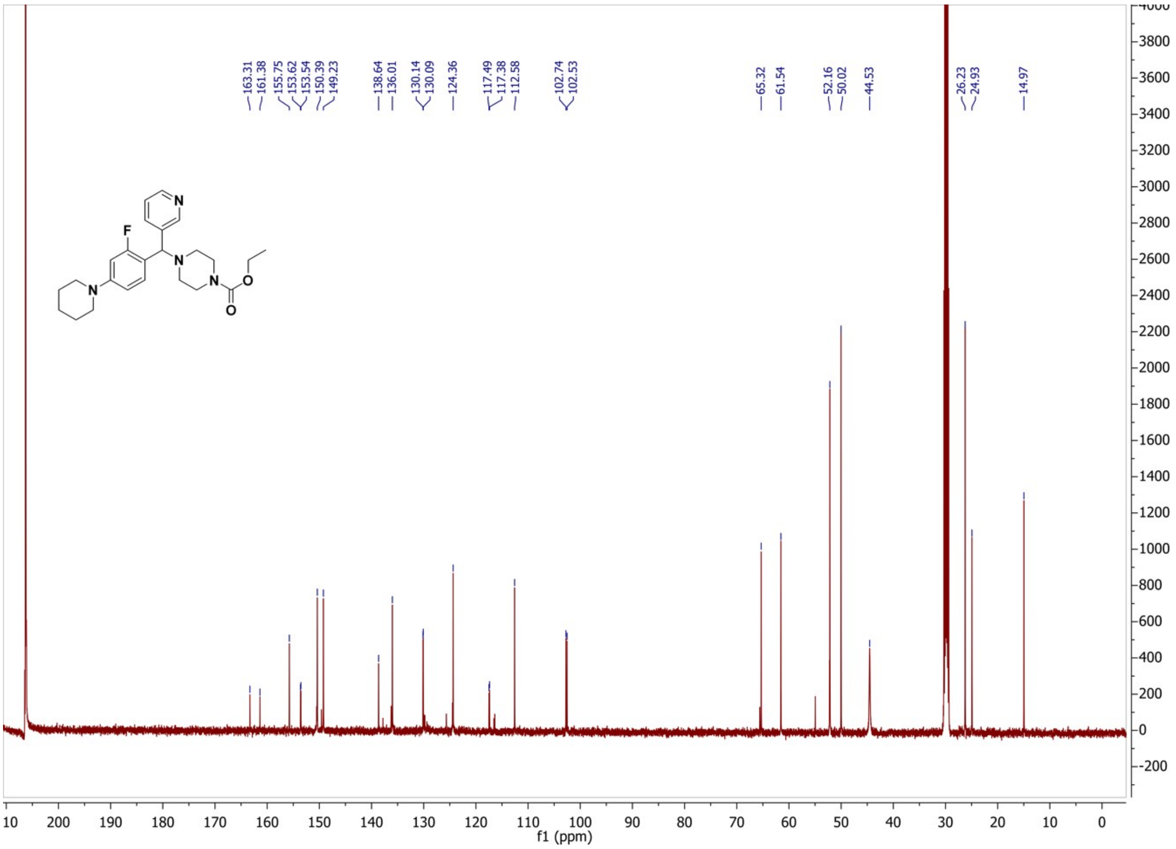

Figure S165. $^{13}C$ NMR spectrum of ethyl 4-((2-fluoro-4-(piperidin-1-yl)phenyl)(pyridin-3-yl)methyl)piperazine-1-carboxylate (**MYOS_00519**, 126 MHz, Acetone-$d_6$).

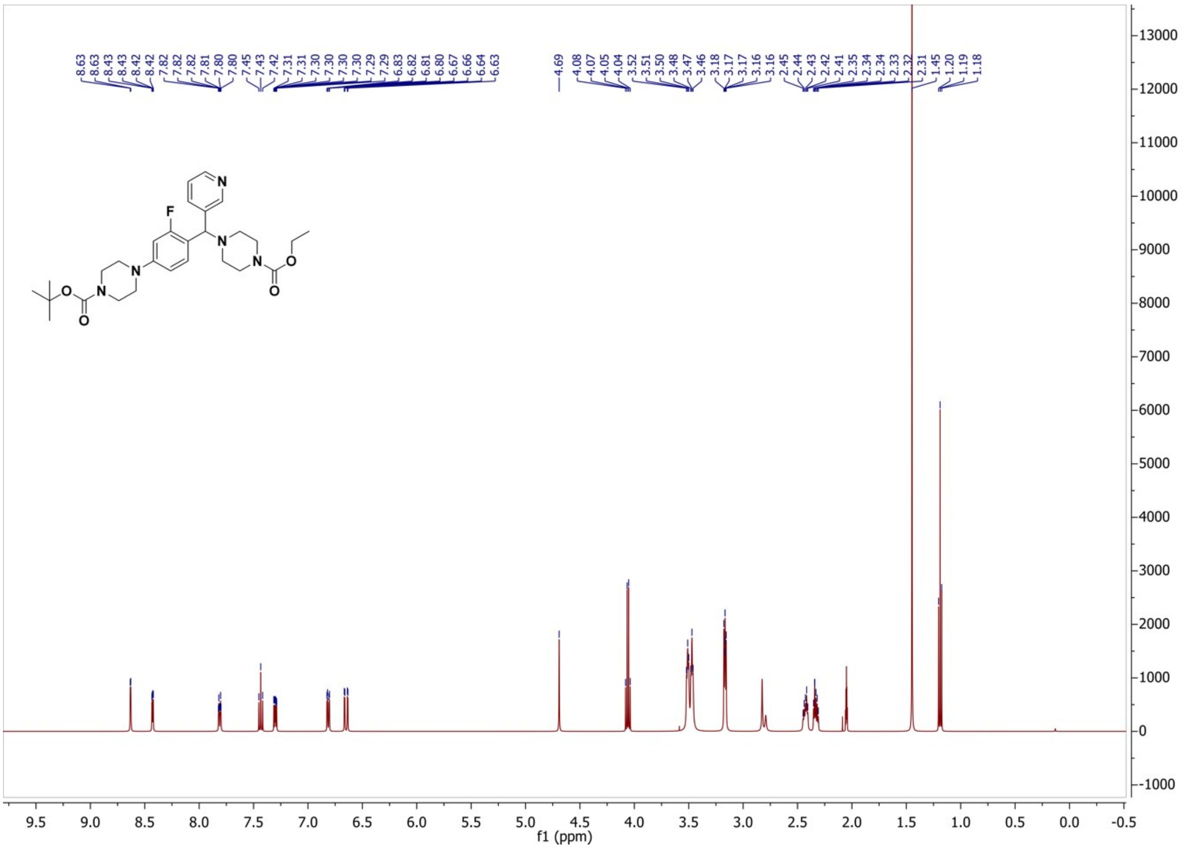


Figure S166. $^{1}$H NMR spectrum of *tert*-butyl 4-(4-((4-(ethoxycarbonyl)piperazin-1-yl)(pyridin-3-yl)methyl)-3-fluorophenyl)piperazine-1-carboxylate (**MYOS_00520**, 500 MHz, Acetone-$d_6$).

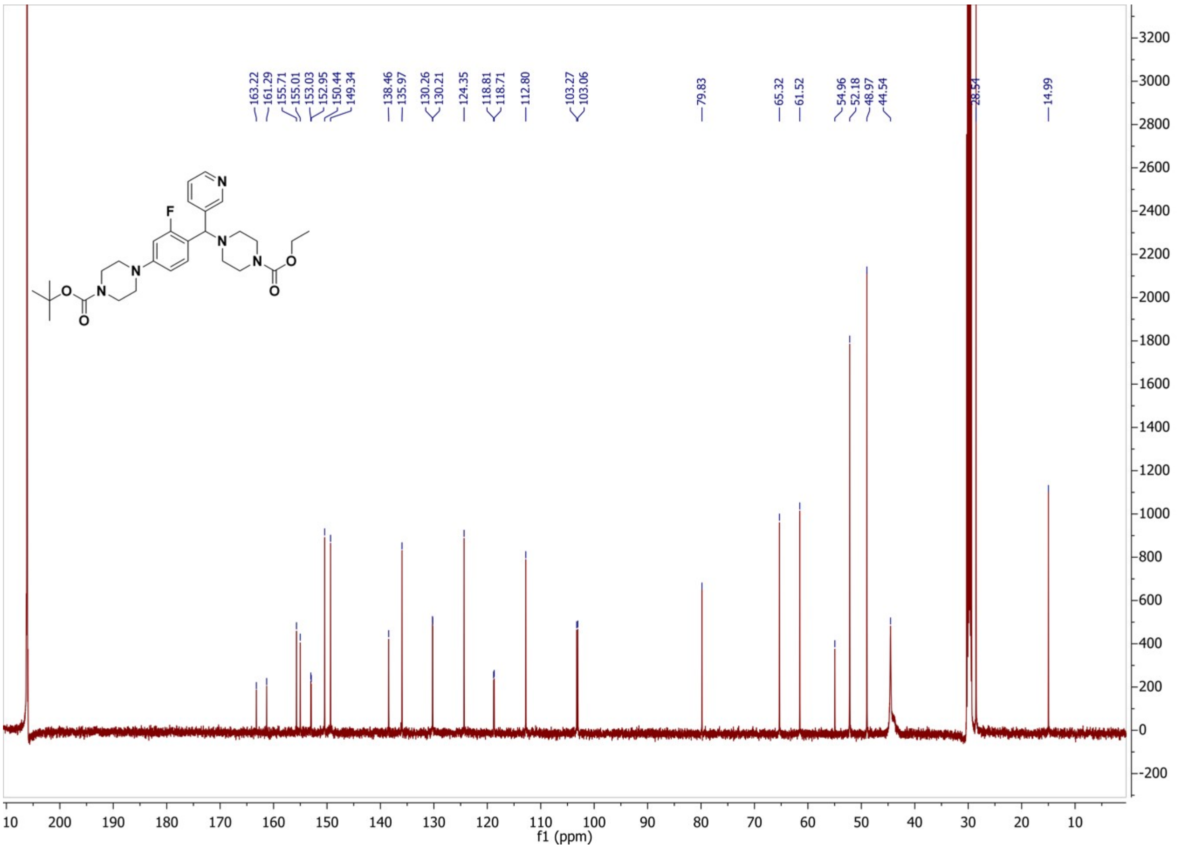


Figure S167. $^{13}$C NMR spectrum of *tert*-butyl 4-(4-((4-(ethoxycarbonyl)piperazin-1-yl)(pyridin-3-yl)methyl)-3-fluorophenyl)piperazine-1-carboxylate (**MYOS_00520**, 126 MHz, Acetone-$d_6$).

**Compounds Contributed from IMSA (Illinois Mathematics and Science Academy)**

MYOS_00312, MYOS_00313 and MYOS_00314 were contributed by students from IMSA under the guidance of John Thurmond. Quality control (LRMS and an assessment of purity) on these compounds was judged by the LCMS traces obtained at UCL (below). NMR peak listings were also provided, but it was not possible to secure reproductions of the NMR spectra.

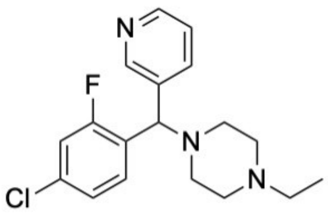

**MYOS312**
DNDI0003974625
PCOSNIMSAP4-001
P4_A_001

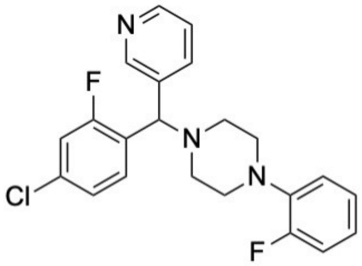

**MYOS313**
DNDI0003974627
PCOSNIMSAP4-003
P4_B_004

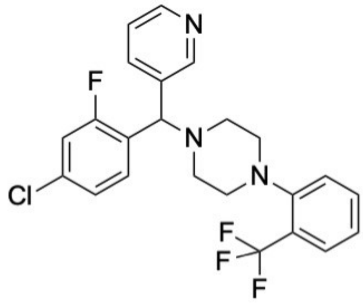

**MYOS314**
DNDI0003974626
PCOSNIMSAP4-002
P4_B_005

1-((4-Chloro-2-fluorophenyl)(pyridin-3-yl)methyl)-4-ethylpiperazine (**MYOS_00312**) (60 MHz, $CDCl_3$) δ 8.65 (d, $J$ = 3 Hz, 2H), 8.53 (d, $J$ = 2.4 Hz, 1H), 8.42 (d, $J$ = 2.4 Hz, 1H), 7.80–6.88 (m, 3H), 4.70 (s, 1H), 3.11–2.02 (m, 10H), 1.11 (t, $J$ = 10.2 Hz, 2H).

1-((4-Chloro-2-fluorophenyl)(pyridin-3-yl)methyl)-4-(2-fluorophenyl)piperazine (**MYOS_00313**) (60 MHz, $CDCl_3$) δ 8.69 (d, $J$ = 3 Hz, 2H), 8.54 (d, $J$ = 2.4 Hz, 1H), 8.44 (d, $J$ = 3 Hz, 1H), 7.88–6.71 (m, 7H), 4.77 (s, 1H), 3.43–2.46 (m, 8H)

1-((4-Chloro-2-fluorophenyl)(pyridin-3-yl)methyl)-4-(2-(trifluoromethyl)phenyl)piperazine (**MYOS_00314**) (60 MHz, $CDCl_3$) δ 8.71 (d, $J$ = 3 Hz, 2H), 8.54 (d, $J$ = 2.4 Hz, 1H), 8.43 (d, $J$ = 2.4 Hz, 1H), 7.80–6.90 (m, 7H), 4.76 (s, 1H), 3.01–2.52 (m, 8H)

LCMS (+ESI) for MYOS_00312

## Single Injection Report

Agilent Technologies

| | | | |
|---|---|---|---|
| **Data file:** | C:\Users\Public\Documents\ChemStation\1\Data\TODD\DM\Dmitrij 2021-09-24 16-05-22\002-P2-C2-P4_A_001.D | | |
| **Sample name:** | P4_A_001 | | |
| **Description:** | | | |
| **Sample amount:** | 0.000 | **Sample type:** | Sample |
| **Instrument:** | G25LCMS | **Location:** | P2-C2 |
| **Injection date:** | 9/24/2021 4:15:22 PM | **Injection:** | 1 of 1 |
| **Acq. method:** | EGT_5min pos.M | **Injection volume:** | 0.500 |
| **Analysis method:** | EGT_5min pos.M | **Acq. operator:** | Yuhang Wang |
| **Last changed:** | 9/20/2021 1:48:34 PM | | |

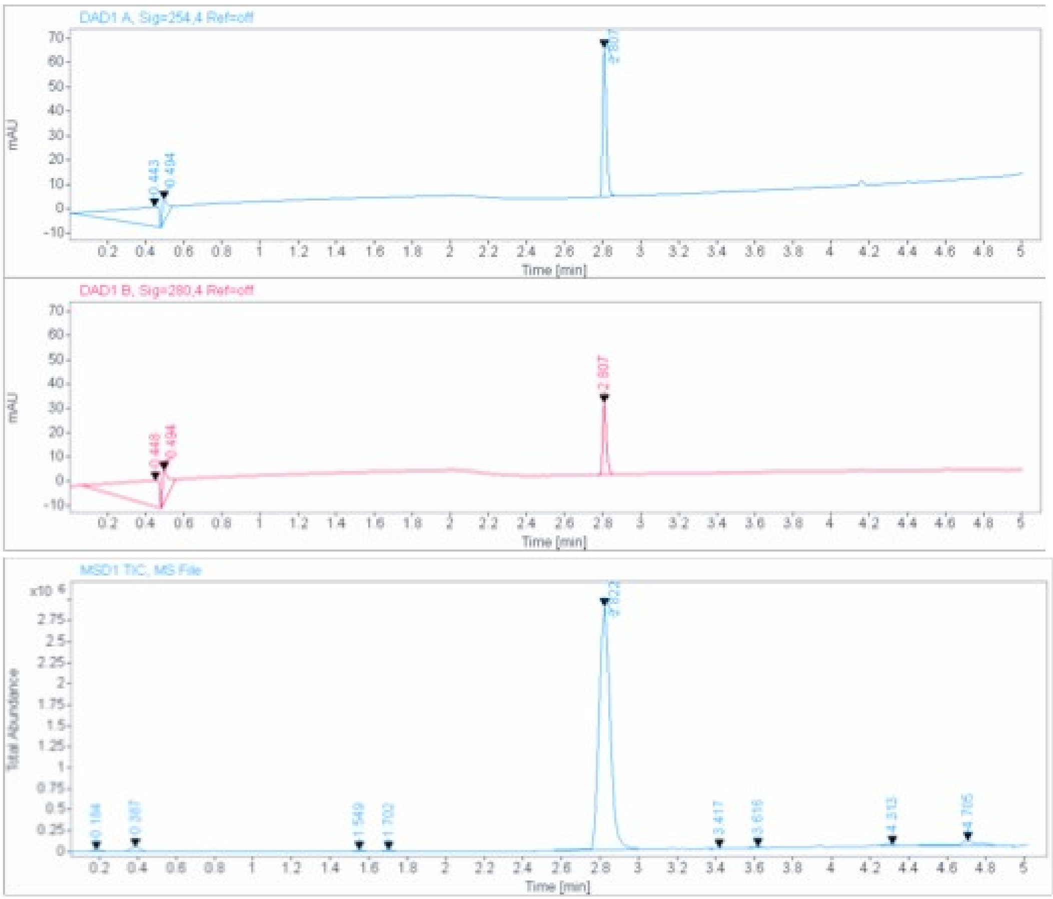

**Signal** MSD1 TIC, MS File

**Peak RT** 0.184

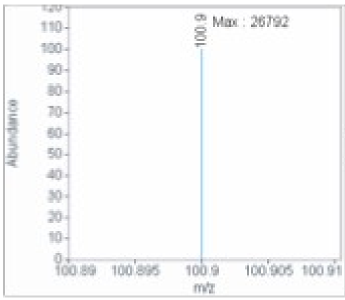


**Peak RT** 0.387

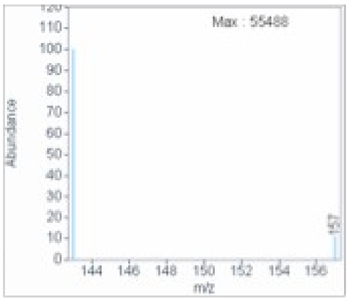


**Peak RT** 1.549

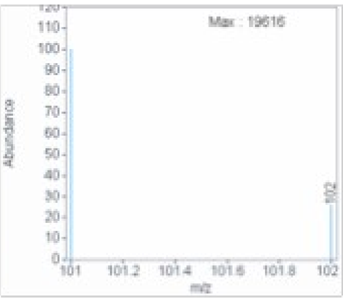


**Peak RT** 1.702

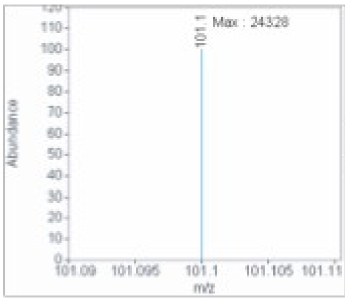


**Peak RT** 2.822

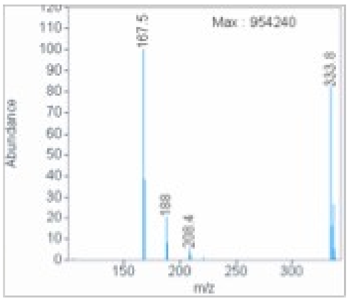


**Peak RT** 3.417

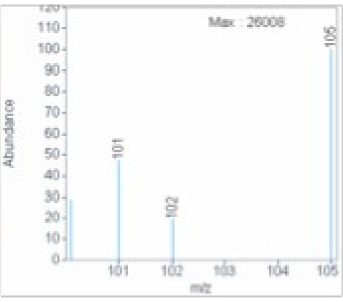


**Peak RT** 3.616

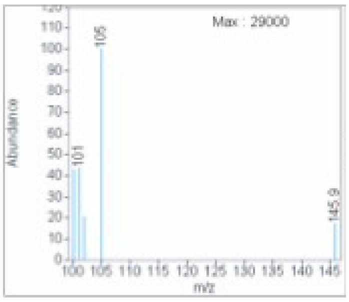


**Peak RT** 4.313

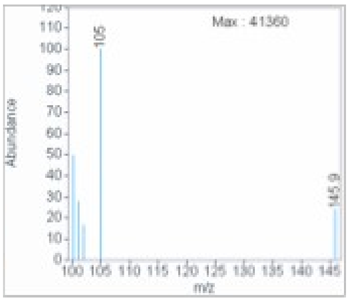


**Peak RT** 4.705

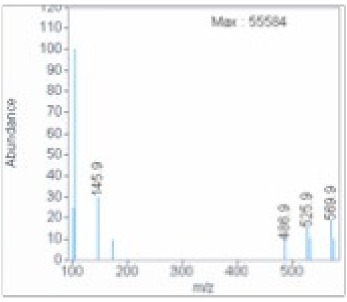

Agilent Technologies

**Signal:** DAD1 A, Sig=254,4 Ref=off

| RT [min] | Type | Width [min] | Area | Height | Area% | Name |
|---|---|---|---|---|---|---|
| 0.443 | BB | 0.1755 | 122.5459 | 8.3867 | 55.7443 | |
| 0.494 | BB | 0.0280 | 18.1070 | 9.6228 | 8.2366 | |
| 2.807 | BB | 0.0196 | 79.1827 | 61.1027 | 36.0191 | |
| | | Sum | 219.8356 | | | |

**Signal:** DAD1 B, Sig=280,4 Ref=off

| RT [min] | Type | Width [min] | Area | Height | Area% | Name |
|---|---|---|---|---|---|---|
| 0.448 | BB | 0.1549 | 142.6768 | 10.9924 | 67.9628 | |
| 0.494 | BB | 0.0311 | 28.7859 | 13.1395 | 13.7119 | |
| 2.807 | BB | 0.0197 | 38.4710 | 29.6071 | 18.3253 | |
| | | Sum | 209.9337 | | | |

**Signal:** MSD1 TIC, MS File

| RT [min] | Type | Width [min] | Area | Height | Area% | Name |
|---|---|---|---|---|---|---|
| 0.184 | BB | 0.0557 | 94196.1172 | 27195.5430 | 0.7356 | |
| 0.387 | BB | 0.0634 | 231493.0781 | 62811.8047 | 1.8079 | |
| 1.549 | BB | 0.0478 | 70800.2422 | 24688.0176 | 0.5529 | |
| 1.702 | BB | 0.0588 | 90731.2031 | 24331.2871 | 0.7086 | |
| 2.822 | BB | 0.0614 | 11498732.0000 | 2909150.5000 | 89.8010 | |
| 3.417 | BB | 0.0331 | 24715.8184 | 12589.6641 | 0.1930 | |
| 3.616 | BB | 0.0480 | 57866.6406 | 21412.9492 | 0.4519 | |
| 4.313 | BB | 0.0751 | 87520.4063 | 21501.8086 | 0.6835 | |
| 4.705 | BB | 0.1184 | 648629.8750 | 69958.4609 | 5.0656 | |
| | | Sum | 12804685.38 | | | |

LCMS (+ESI) for MYOS_00313

## Single Injection Report

Agilent Technologies

| | | | |
|---|---|---|---|
| **Data file:** | C:\Users\Public\Documents\ChemStation\1\Data\TODD\DM\Dmitrij 2021-09-24 16-05-22\003-P2-C3-P4_B_004.D | | |
| **Sample name:** | P4_B_004 | | |
| **Description:** | | | |
| **Sample amount:** | 0.000 | **Sample type:** | Sample |
| **Instrument:** | G25LCMS | **Location:** | P2-C3 |
| **Injection date:** | 9/24/2021 4:22:57 PM | **Injection:** | 1 of 1 |
| **Acq. method:** | EGT_5min pos.M | **Injection volume:** | 0.500 |
| **Analysis method:** | EGT_5min pos.M | **Acq. operator:** | Yuhang Wang |
| **Last changed:** | 9/20/2021 1:48:34 PM | | |

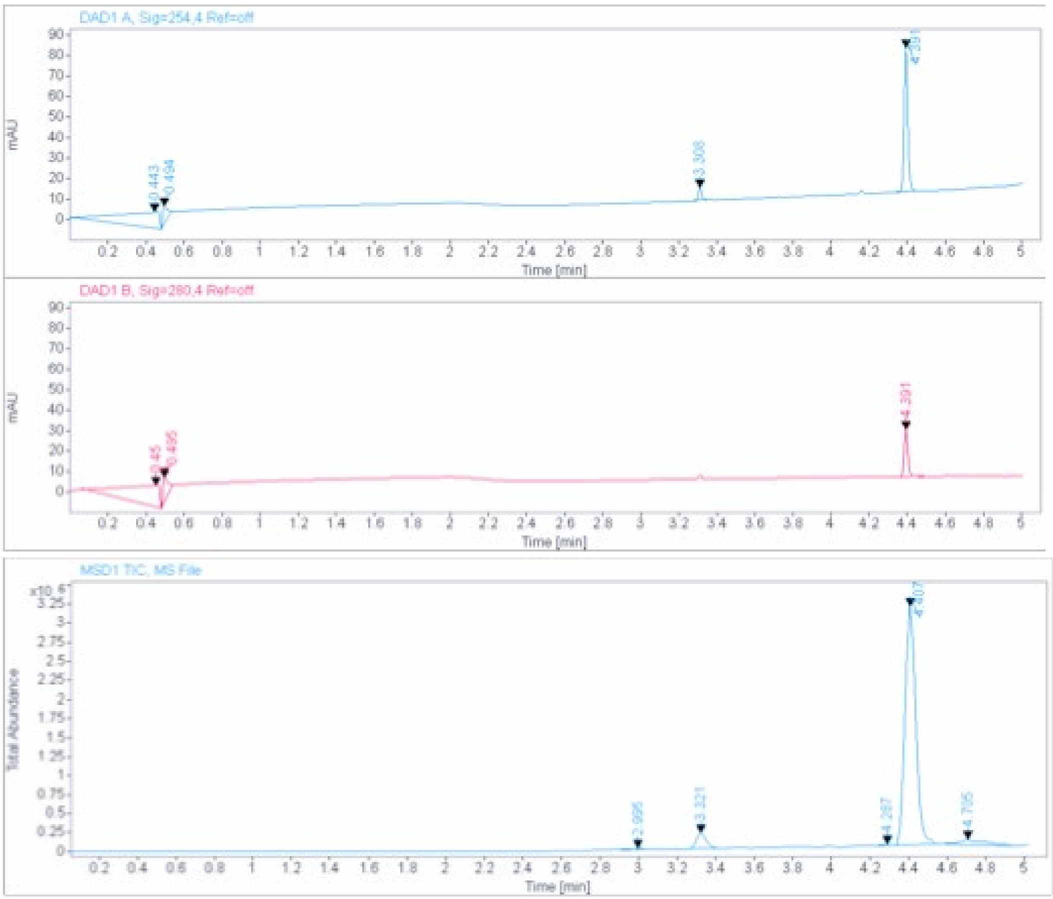

## Single Injection Report

Agilent Technologies

**Signal** MSD1 TIC, MS File

**Peak RT** 2.995

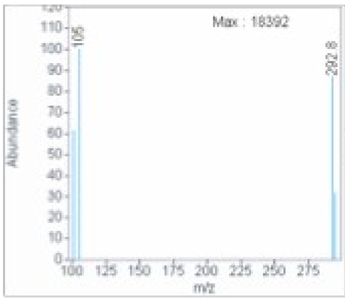


**Peak RT** 3.321

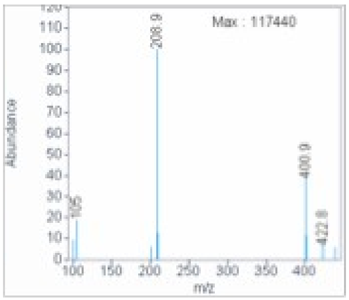


**Peak RT** 4.287

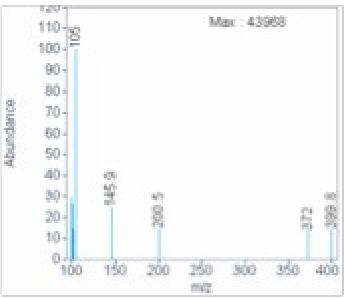


**Peak RT** 4.407

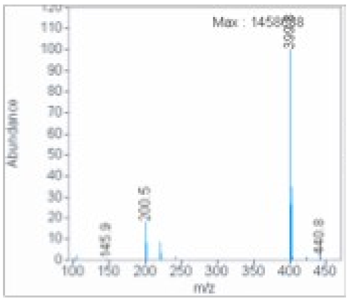


**Peak RT** 4.705

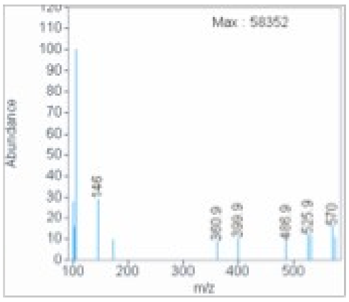

# Single Injection Report

Agilent Technologies

**Signal:** DAD1 A, Sig=254,4 Ref=off

| RT [min] | Type | Width [min] | Area | Height | Area% | Name |
|---|---|---|---|---|---|---|
| 0.443 | BB | 0.1767 | 119.9047 | 8.1012 | 50.3440 | |
| 0.494 | BB | 0.0263 | 15.6533 | 9.0159 | 6.5723 | |
| 3.308 | BB | 0.0189 | 7.2775 | 6.1295 | 3.0556 | |
| 4.391 | BB | 0.0208 | 95.3354 | 70.1767 | 40.0282 | |
| | | Sum | 238.1709 | | | |

**Signal:** DAD1 B, Sig=280,4 Ref=off

| RT [min] | Type | Width [min] | Area | Height | Area% | Name |
|---|---|---|---|---|---|---|
| 0.450 | BB | 0.1551 | 138.2326 | 10.7472 | 71.7018 | |
| 0.495 | BB | 0.0279 | 22.8505 | 12.1847 | 11.8527 | |
| 4.391 | BB | 0.0209 | 31.7051 | 23.2003 | 16.4456 | |
| | | Sum | 192.7882 | | | |

**Signal:** MSD1 TIC, MS File

| RT [min] | Type | Width [min] | Area | Height | Area% | Name |
|---|---|---|---|---|---|---|
| 2.995 | BB | 0.0448 | 65187.2148 | 21951.5371 | 0.4703 | |
| 3.321 | BB | 0.0573 | 765328.8125 | 212406.7188 | 5.5219 | |
| 4.287 | BB | 0.0400 | 50070.3789 | 20863.4648 | 0.3613 | |
| 4.407 | BB | 0.0610 | 12300361.0000 | 3138220.7500 | 88.7475 | |
| 4.705 | BB | 0.1173 | 679007.1250 | 73992.5938 | 4.8991 | |
| | | Sum | 13859954.53 | | | |

LCMS (+ESI) for MYOS_00314

## Single Injection Report

Agilent Technologies

| | | | |
|---|---|---|---|
| **Data file:** | C:\Users\Public\Documents\ChemStation\1\Data\TODD\DM\Dmitrij 2021-09-24 16-05-22\004-P2-C4-P4_B_005.D | | |
| **Sample name:** | P4_B_005 | | |
| **Description:** | | | |
| **Sample amount:** | 0.000 | **Sample type:** | Sample |
| **Instrument:** | G25LCMS | **Location:** | P2-C4 |
| **Injection date:** | 9/24/2021 4:30:33 PM | **Injection:** | 1 of 1 |
| **Acq. method:** | EGT_5min pos.M | **Injection volume:** | 0.500 |
| **Analysis method:** | EGT_5min pos.M | **Acq. operator:** | Yuhang Wang |
| **Last changed:** | 9/20/2021 1:48:34 PM | | |

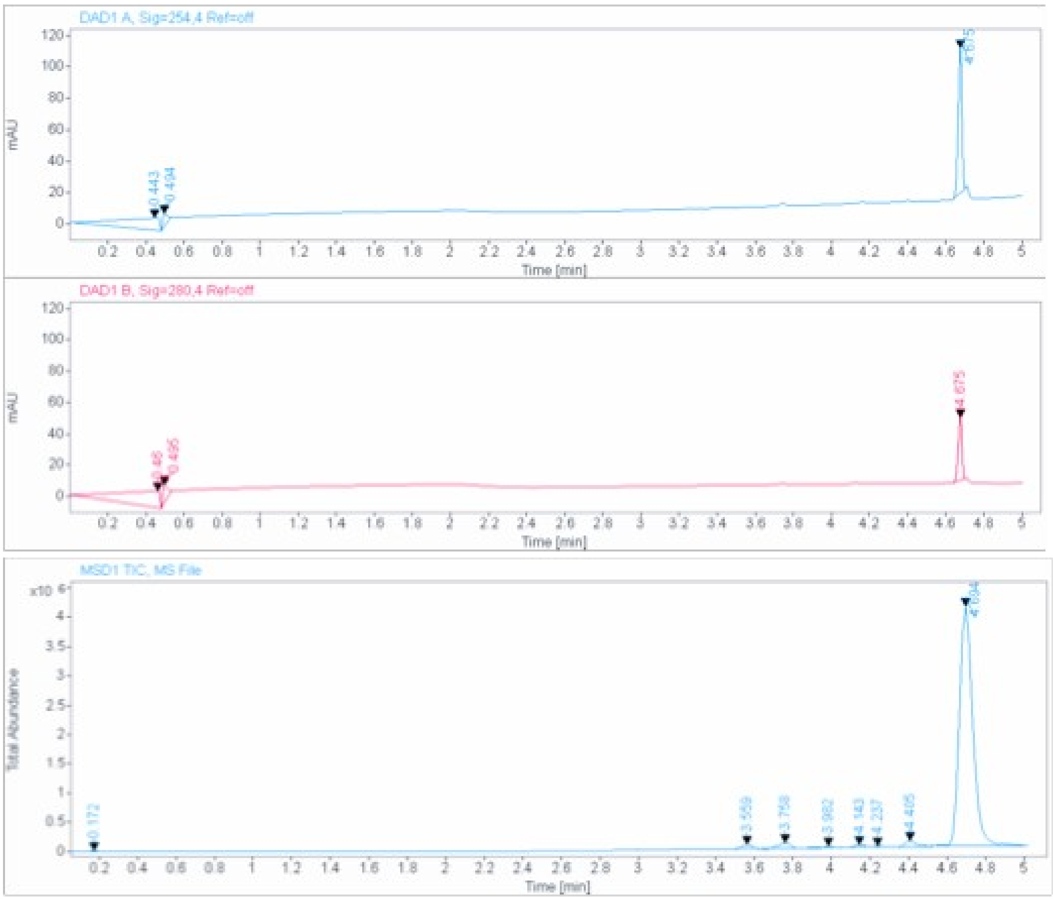

**Signal** MSD1 TIC, MS File

**Peak RT** 0.172

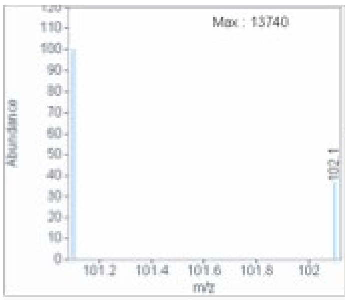


**Peak RT** 3.559

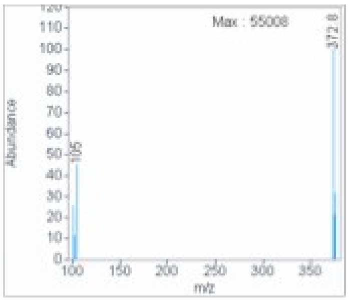


**Peak RT** 3.758

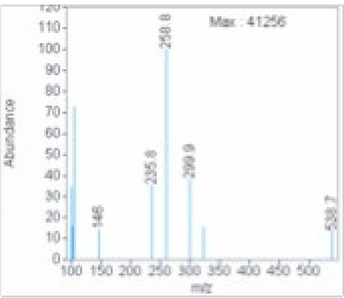


**Peak RT** 3.982

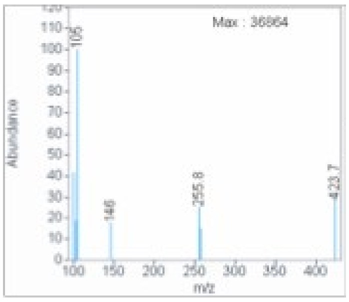


**Peak RT** 4.143

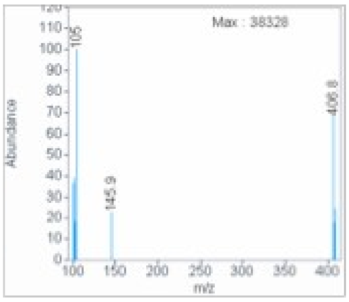


**Peak RT** 4.237

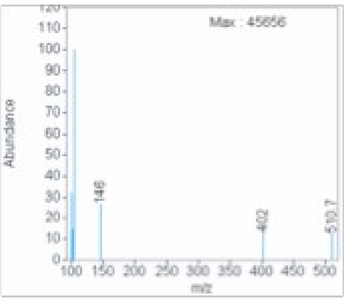


**Peak RT** 4.405

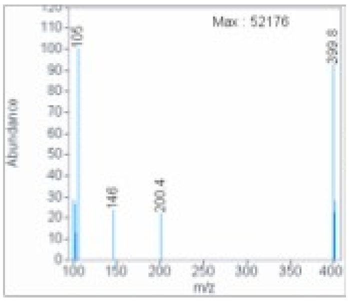


**Peak RT** 4.694

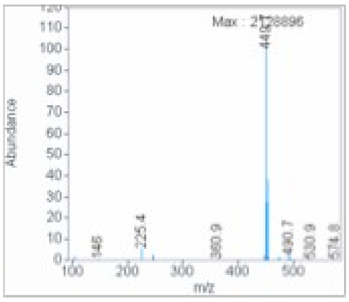

# Single Injection Report

Agilent Technologies

**Signal:** DAD1 A, Sig=254,4 Ref=off

| RT [min] | Type | Width [min] | Area | Height | Area% | Name |
|---|---|---|---|---|---|---|
| 0.443 | BB | 0.1763 | 117.9271 | 7.9866 | 45.8271 | |
| 0.494 | BB | 0.0258 | 14.8478 | 8.7576 | 5.7699 | |
| 4.675 | BB | 0.0207 | 124.5554 | 92.3550 | 48.4029 | |
| | | Sum | 257.3303 | | | |

**Signal:** DAD1 B, Sig=280,4 Ref=off

| RT [min] | Type | Width [min] | Area | Height | Area% | Name |
|---|---|---|---|---|---|---|
| 0.460 | BB | 0.1768 | 160.2784 | 10.8825 | 68.1054 | |
| 0.495 | BB | 0.0263 | 20.3541 | 11.7051 | 8.6488 | |
| 4.675 | BB | 0.0208 | 54.7063 | 40.3037 | 23.2458 | |
| | | Sum | 235.3387 | | | |

**Signal:** MSD1 TIC, MS File

| RT [min] | Type | Width [min] | Area | Height | Area% | Name |
|---|---|---|---|---|---|---|
| 0.172 | BB | 0.0487 | 53768.2813 | 18800.6875 | 0.2472 | |
| 3.559 | BB | 0.0603 | 279342.3125 | 81731.0078 | 1.2844 | |
| 3.758 | BB | 0.0714 | 487220.4688 | 101372.3828 | 2.2403 | |
| 3.982 | BB | 0.0717 | 178309.6719 | 36856.3164 | 0.8199 | |
| 4.143 | BB | 0.0531 | 171412.7813 | 46666.0117 | 0.7882 | |
| 4.237 | BB | 0.0423 | 42553.3789 | 18508.4316 | 0.1957 | |
| 4.405 | BB | 0.0640 | 448808.6875 | 107505.4063 | 2.0637 | |
| 4.694 | BBA | 0.0725 | 20086792.0000 | 4093704.0000 | 92.3607 | |
| | | Sum | 21748207.58 | | | |

**Large Scale Synthesis of Int1**



Reaction Details:

| Temperature | Time | Pressure | Main Yield | Succeed | Recommendation | Lab Room |
|---|---|---|---|---|---|---|
| 4-15 °C | 5 hr | | 67.04% | Succeeded | ★★★ | 16-415 |
| **Compound ID** | **WuXi ID** | **Compound Novelty** | **Compound Color** | **Compound state** | **Stability** | **Final Step** |
| N/A | N/A | Unknown Compound | White | Solid | | X |

Scheme:

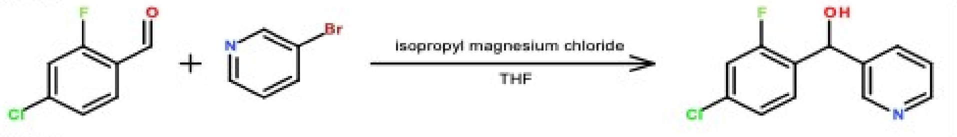


Reactants:

| Sample ID | Structure | Salt | MW | Eq | Amount | Moles | Source | [Stock] | Vol | Purity | %ee |
|---|---|---|---|---|---|---|---|---|---|---|---|
| EW618-1841-R1 | 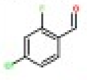 | | 158.56 | 1 | 80 g | 504.55 mmol | Domestic | | | | |
| EW618-1841-R2 | 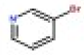 | | 158.00 | 1.13 | 90 g | 569.64 mmol | Domestic | | 54.88 mL | | |

Reagents:

| Reagent | MW | Eq | Amount | Moles | Source | [Stock] | Vol | Cat. | Purity |
|---|---|---|---|---|---|---|---|---|---|
| isopropyl magnesium chloride | 102.85 | 1.19 | 61.51 g | 598.12 mmol | | 2 M | 299.06 mL | | |

Solvents:

| Solvent | Vol | Source | Notes |
|---|---|---|---|
| THF | 800 mL | | |
| THF | 300 mL | | |

Products:

| Sample ID | Structure | MW | Amount | Moles | Theory Amount | Purity | %ee | Yld | Batches in parallel | Products state | Vol |
|---|---|---|---|---|---|---|---|---|---|---|---|
| EW618-1841-P1 | 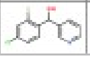 | 237.66 | 81.2 g | 338.25 mmol | 119.91 g | 99% | | 67.04% | | | |

Reference:

| Journal of Abbr. | Year | Volume | Issue | Page Range | Notes |
|---|---|---|---|---|---|
| N/A | | | | | |

Procedure:

**[Reaction Setup]**

To a solution of 3-bromopyridine (90 g, 569.64 mmol, 54.88 mL, 1.13 *eq*) in THF (800 mL) was added isopropyl magnesium chloride (2 M, 299.06 mL, 1.19 *eq*) dropwise at 4 °C. After stirred 2 h at 15°C(room temperature). A solution of 4-chloro-2-fluoro-benzaldehyde (80 g, 504.55 mmol, 1 *eq*) in THF (300 mL) was added at 4°C. The mixture was stirred at 15°C(room temperature) for 3 h.

**[Monitoring]**

TLC(PE/EA=1/1) indicated no Reactant 1 was remained, and one major new spot was detected.

**[Work-up]**

The reaction mixture was poured into sat. NH4Cl(1 L) and extracted with EA(800 mL*2). The combined organic layers were washed with brine(1 L), dried over Na2SO4, filtered and concentrated under reduced pressure to give a residue.

**[Purification]**

The residue was triturated with the mixed solvent(PE/EA=10/1, 200 mL) at room temperature for 30 min, filtered and the cake

was dried under reduced pressure.

**[Result]**

Compound (4-chloro-2-fluoro-phenyl)-(3-pyridyl)methanol (81.2 g, 338.25 mmol, 67.04% yield, 99% purity) (NMR: EW618-1841-P1A, HPLC:EW618-1841-P1H, LCMS:EW618-1841-P1L)was obtained as a white solid.

TLC:

| Plate NO | Eluent | Ratio | Color Developing Reagent | Others |
|---|---|---|---|---|
| Plate 1 | Petroleum ether : Ethyl acetate | 1:1 | 紫外(UV 254 nm) | |

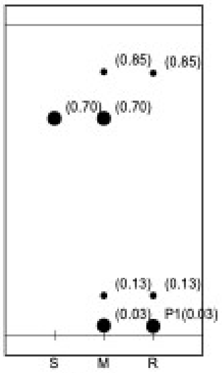

Plate 1

Confidential. For research information only

# LCMS REPORT

Compound ID : EW618-1841-P1A
Sample ID : EW618-1841-P1A
Injection Vol : 1ul
Location : vial42
Acq Method : d:\method\5-95AB_R_220&254.lcm
Org DataFile : D:\DATA\1903\190315\EW618-1841-P1A.lcd
Injection Date : 2019-03-15 11:11:42
Instrument : LCMS-Q 17-102

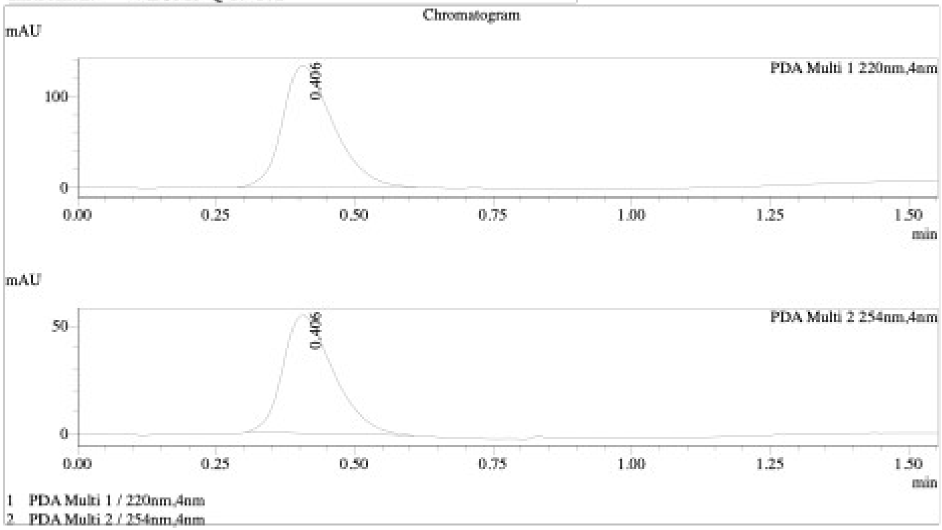


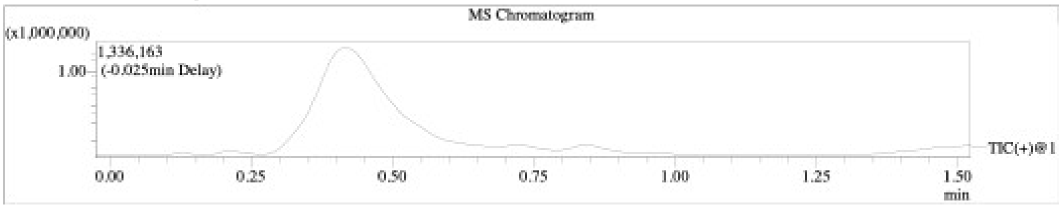


Integration Result

Peak Table

PDA Ch1 220nm

| Peak# | Ret. Time | Height | Height% | USP Width | Area | Area% |
|---|---|---|---|---|---|---|
| 1 | 0.406 | 133294 | 100.000 | 0.164 | 844153 | 100.000 |

Peak Table

PDA Ch2 254nm

| Peak# | Ret. Time | Height | Height% | USP Width | Area | Area% |
|---|---|---|---|---|---|---|
| 1 | 0.406 | 54507 | 100.000 | 0.164 | 343138 | 100.000 |

Confidential. For research information only

Operator:______________

Date:_________________

Confidential. For research information only

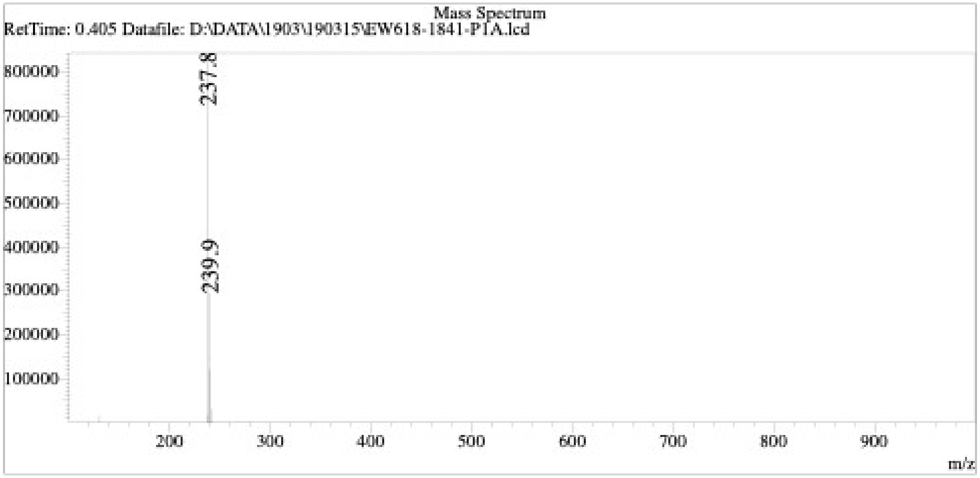

**Compound ID: EW618-1841-P1A**

EW618-1841-P1A DMSO Bruker_F_400MHz

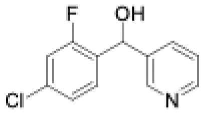


Exact Mass: 237.04

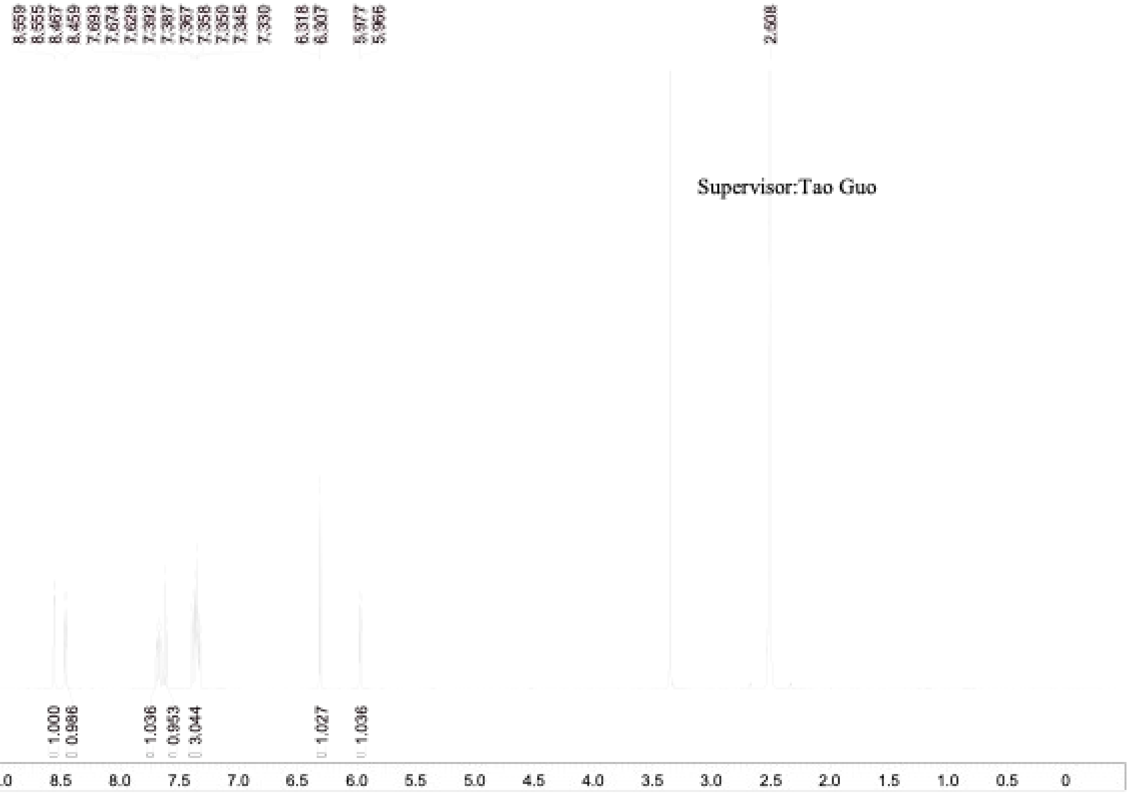


Operator: Date:

Confidential, for research only not for regulatory filing

# HPLC REPORT

| | |
|---|---|
| Compound ID | : EW618-1841-P1A |
| Sample ID | : EW618-1841-P1H |
| Vial# | : 64 |
| Injection Volumn | : 1 |
| Filename | : D:\DATA\2019\1903\190315\EW618-1841-P1H.lcd |
| Method Name | : D:\Method\10-80AB_4min.lcm |
| Instrument | : HPLC-R |
| Run time | : 2019-03-15 10:44:17 |

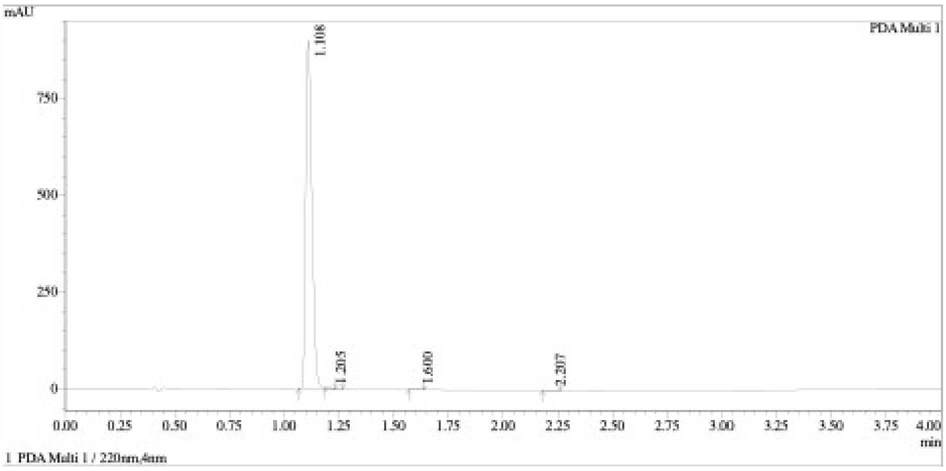


Integration result

PeakTable

PDA Ch1 220nm

| Peak# | Ret. Time | USP Width | Resolution | Height | Area | Area % |
|---|---|---|---|---|---|---|
| 1 | 1.108 | 0.054 | 0.000 | 899786 | 1796014 | 99.440 |
| 2 | 1.205 | 0.038 | 2.119 | 1508 | 1987 | 0.110 |
| 3 | 1.600 | 0.041 | 9.980 | 3493 | 5282 | 0.292 |
| 4 | 2.207 | 0.046 | 13.922 | 1715 | 2845 | 0.157 |
| Total | | | | 906501 | 1806128 | 100.000 |

Operater : ______________

Date : ______________

Confidential. For research information only

# HPLC REPORT

Compound ID : EW618-1841-P1A
Sample ID : EW618-1841-P1H
Filename : D:\DATA\2019\1903\190315\EW618-1841-P1H.lcd
Method Name : D:\Method\10-80AB_4min.lcm
Instrument &Column : HPLC-R
Run time : 2019-03-15 10:44:17

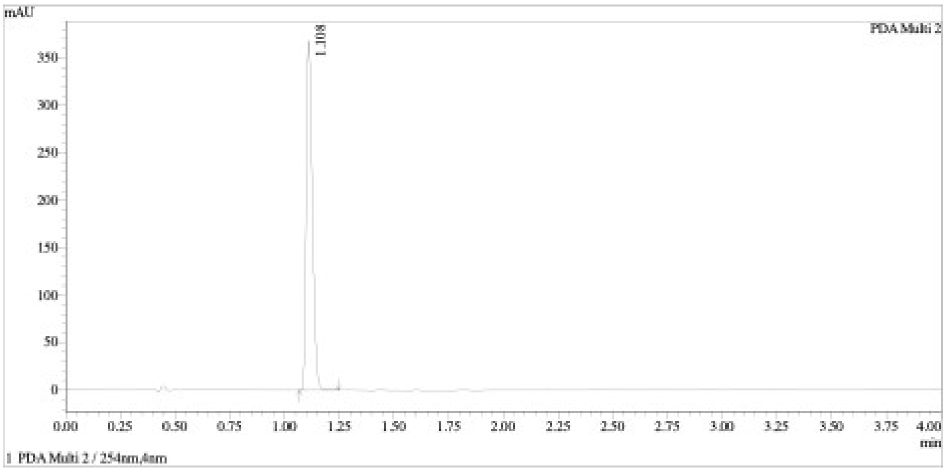


Integration result

PeakTable
PDA Ch2 254nm

| Peak# | Ret. Time | USP Width | Resolution | Height | Area | Area % |
|---|---|---|---|---|---|---|
| 1 | 1.108 | 0.053 | 0.000 | 368799 | 728870 | 100.000 |
| Total | | | | 368799 | 728870 | 100.000 |

Operater : ________________

Date : ________________

Confidential. For research information only

# HPLC REPORT

Compound ID : EW618-1841-P1A
Sample ID : EW618-1841-P1H
Filename : D:\DATA\2019\1903\190315\EW618-1841-P1H.lcd
Method Name : D:\Method\10-80AB_4min.lcm
Instrument &Column : HPLC-R
Run time : 2019-03-15 10:44:17

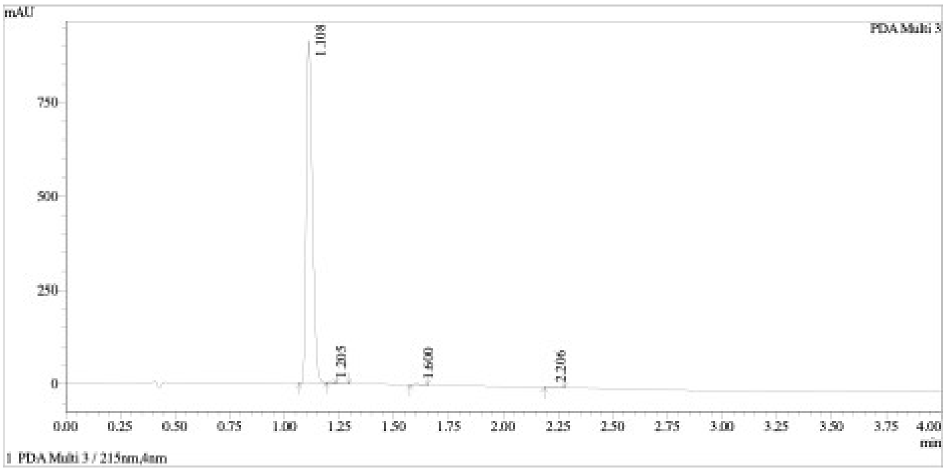


Integration result

PeakTable

PDA Ch3 215nm

| Peak# | Ret. Time | USP Width | Resolution | Height | Area | Area % |
|---|---|---|---|---|---|---|
| 1 | 1.108 | 0.054 | 0.000 | 913066 | 1811882 | 99.473 |
| 2 | 1.205 | 0.037 | 2.134 | 1466 | 1909 | 0.105 |
| 3 | 1.600 | 0.042 | 10.119 | 3360 | 4992 | 0.274 |
| 4 | 2.206 | 0.044 | 14.167 | 1556 | 2698 | 0.148 |
| Total | | | | 919448 | 1821481 | 100.000 |

Operater : ______________

Date : ______________

**References**


[1] Lim, W.; Melse, Y.; Konings, M.; Phat Duong, H.; Eadie, K.; Laleu, B.; Perry, B.; Todd, M. H.; Ioset, J. R.; van de Sande, W. W. J. Addressing the most neglected diseases through an open research model: The discovery of fenarimols as novel drug candidates for eumycetoma. *PLoS Negl Trop Dis* **2018**, *12* (4), e0006437. DOI: 10.1371/journal.pntd.0006437

[2] Burchat, A. F.; Chong, J. M.; Nielsen, N. Titration of alkyllithiums with a simple reagent to a blue endpoint. *J. Organomet. Chem.* **1997**, 542 (2), 281–283. DOI: 10.1016/S0022-328X(97)00143-5

[3] Keenan, M., *et al.*, Design, structure–activity relationship and *in vivo* efficacy of piperazine analogues of fenarimol as inhibitors of *Trypanosoma cruzi*, *Biorg. Med. Chem*. **2013**. 21(7): 1756–1763. DOI: 10.1016/j.bmc.2013.01.050

[4] Keenan, M., *et al.*, Two analogues of fenarimol show curative activity in an experimental model of Chagas disease, *J. Med. Chem*. **2013**. 56(24): 10158–10170. DOI: 10.1021/jm401610c

[5] Keenan, M., *et al.*, Analogues of fenarimol are potent inhibitors of *Trypanosoma cruzi* and are efficacious in a murine model of Chagas disease, *J. Med. Chem*. **2012**. 55(9): 4189–4204. DOI: 10.1021/jm2015809

[6] Kato, K., Ohkawa, S., Terao, S., Terashita, Z., Nishikawa, K. Thromboxane synthetase inhibitors (TXSI). Design, synthesis, and evaluation of a novel series of ω-pyridylalkenoic acids, *J. Med. Chem.* **1985**, *28*, 287–294. DOI: 10.1021/jm00381a005.

[7] Duong, H. P. Synthesis of novel fenarimol analogues as drug candidates for the neglected tropical disease mycetoma, The University of Sydney, **2017**. Available at https://github.com/OpenSourceMycetoma/Series-1-Fenarimols/tree/master/Theses%20Reports%20Docs/Hung%20Duong%20Hons%20Thesis

[8] Ohno, M., Miyamoto, M., Hoshi, K., Takeda, T., Yamada, N., Ohtake, A. Development of dual acting benzofurans for thromboxane $A_2$ receptor antagonist and prostacyclin receptor agonist: synthesis, structure−activity relationship, and evaluation of benzofuran derivatives. *J. Med. Chem.* **2005**, *48* (16), 5279–5294. DOI: 10.1021/jm050194z.

[9] Fleckenstein, C. A., Plenio, H. 9 Fluorenylphosphines for the Pd catalyzed Sonogashira, Suzuki, and Buchwald–Hartwig coupling reactions in organic solvents and water. *Chem. Eur. J.* **2007**, *13* (9), 2701–2716. DOI: 10.1002/chem.200601142

[10] Trécourt, F., Breton, G., Bonnet, V., Mongin, F., Marsais, F., Quéguiner, G. New syntheses of substituted pyridines via bromine–magnesium exchange. *Tetrahedron* **2000**, *56* (10), 1349–1360. DOI: 10.1016/S0040-4020(00)00027-2

[11] Sutherlin, D. P. *et al.*, Discovery of a potent, selective, and orally available class I phosphatidylinositol 3-kinase

(PI3K)/mammalian target of rapamycin (mTOR) kinase inhibitor (GDC-0980) for the treatment of cancer, *J. Med. Chem*. **2011**, *54*(21), 7579–7587. DOI: 10.1021/jm2009327.

[12] Masse, C. E. et al., Tyk2 inhibitors and uses thereof, W.I.P. Organization, 2015, USA, WO2015131080A1

[13] Liu, J., Eaton, J. B., Caldarone, B., Lukas, R. J., Kozikowski, A. P. Chemistry and pharmacological characterization of novel nitrogen analogues of AMOP-H-OH (Sazetidine-A, 6-[5-(azetidin-2-ylmethoxy)pyridin-3-yl]hex-5-yn-1-ol) as α4β2-nicotinic acetylcholine receptor-selective partial agonists, *J. Med. Chem*. **2010**, *53*(19): 6973–6985. DOI: 10.1021/jm100765u.

[14] Griffioen, G. *et al.*, New compounds for the treatment of neurodegenerative diseases, W.I.P. Organization, **2012**, Belgium, WO2012080221.
